# Transient Exposure to Low Levels of Insecticide Affects Metabolic Networks of Honeybee Larvae

**DOI:** 10.1371/journal.pone.0068191

**Published:** 2013-07-02

**Authors:** Kamila Derecka, Martin J. Blythe, Sunir Malla, Diane P. Genereux, Alessandro Guffanti, Paolo Pavan, Anna Moles, Charles Snart, Thomas Ryder, Catharine A. Ortori, David A. Barrett, Eugene Schuster, Reinhard Stöger

**Affiliations:** 1 School of Biosciences, University of Nottingham, Sutton Bonington Campus, Leicestershire, United Kingdom; 2 Deep Seq, Centre for Genetics and Genomics, University of Nottingham, Nottingham, United Kingdom; 3 Biology Department, Westfield State University, Westfield, Massachusetts, United States of America; 4 Genomnia srl, Lainate, Milan, Italy; 5 Parks Apiaries, Nottingham, United Kingdom; 6 Centre for Analytical Bioscience, School of Pharmacy, University of Nottingham, Nottingham, United Kingdom; 7 Institute of Healthy Ageing, Department of Genetics, Evolution and Environment, University College London, London, United Kingdom; East Carolina University, United States of America

## Abstract

The survival of a species depends on its capacity to adjust to changing environmental conditions, and new stressors. Such new, anthropogenic stressors include the neonicotinoid class of crop-protecting agents, which have been implicated in the population declines of pollinating insects, including honeybees (*Apis mellifera*). The low-dose effects of these compounds on larval development and physiological responses have remained largely unknown. Over a period of 15 days, we provided syrup tainted with low levels (2 µg/L^−1^) of the neonicotinoid insecticide imidacloprid to beehives located in the field. We measured transcript levels by RNA sequencing and established lipid profiles using liquid chromatography coupled with mass spectrometry from worker-bee larvae of imidacloprid-exposed (IE) and unexposed, control (C) hives. Within a catalogue of 300 differentially expressed transcripts in larvae from IE hives, we detect significant enrichment of genes functioning in lipid-carbohydrate-mitochondrial metabolic networks. Myc-involved transcriptional response to exposure of this neonicotinoid is indicated by overrepresentation of E-box elements in the promoter regions of genes with altered expression. RNA levels for a cluster of genes encoding detoxifying P450 enzymes are elevated, with coordinated downregulation of genes in glycolytic and sugar-metabolising pathways. Expression of the environmentally responsive *Hsp90* gene is also reduced, suggesting diminished buffering and stability of the developmental program. The multifaceted, physiological response described here may be of importance to our general understanding of pollinator health. Muscles, for instance, work at high glycolytic rates and flight performance could be impacted should low levels of this evolutionarily novel stressor likewise induce downregulation of energy metabolising genes in adult pollinators.

## Introduction

Species populating today’s Earth all derive from ancestors that prevailed in changeable environments. Waddington described the tendency of an organism to produce definitive phenotypes under variable conditions as ‘canalisation’, which implies some robustness of the genetically encoded developmental program [1]. Such canalisation is not absolute. With too great of an environmental stimulus, developmental buffering systems can fail, causing abnormities or death during development. A number of molecular mechanisms have been proposed and identified as contributing to canalisation. These mechanisms include microRNAs [2], [3], [4], [5], small non-coding RNAs that modulate networks of protein-coding genes and the heat-shock protein 90 (Hsp90), a molecular chaperone that stabilises developmental programs [6]. A characteristic property for both Hsp90 and microRNA buffering systems is their ability to facilitate rapid adjustments.

Taxing conditions often require energy-consuming physiological adjustments: altered metabolic demands must be coordinated with organismal growth to ensure completion of the developmental program under adverse conditions. Hormones participate in regulating metabolic aspects of the stress response [7]. In the fruit fly *Drosophila*, hormonal and nutritional signals are integrated by the transcription factor Myc (dMyc), which functions as an intermediate regulator of growth during development [8]. Insulin indirectly regulates dMyc in the larval fat body and muscle through FOXO and TOR complex 1 (TORC1) signaling pathways, causing changes in expression of a large metabolic gene network [9], [10]. Both TORC1 and FOXO, which act upstream of dMyc, also respond to additional environmental cues, including immune challenges and changes in oxygen levels [11], [12]. Thus, there is evidence for the evolution of developmental buffering systems to meet the challenges of environmental stressors recurrently experienced during life histories.

Entirely new environmental hazards, and those not encountered previously by a species present a conundrum. The organism must rapidly deal with the novel stressor in order to survive, but the genome-encoded options for responding are often limited. The synthetic class of neonicotinoid insecticides represents such a new type of environmental stressor. Registered for use in more than 120 countries and accounting for around 24% of the global market, neonicotinoids have been the most important class of agricultural pesticides in recent years [13]. The success of these synthetic insecticides in agricultural applications largely derives from two factors. First, neonicotinoids selectively target nicotinic acetylcholine receptors (nAChRs) of insects and as a result have replaced older, more hazardous organophosphate and organochlorine pesticides, which have a much higher toxicity for vertebrates [14]. Second, neonicotinoids act systemically; they are absorbed, distributed and maintained throughout the plant upon seed or soil treatment. The necessity of repeated crop spraying is reduced. Although development and application of neonicotinoids can be viewed favourably insofar as they contribute to an overall improvement to modern commercial agricultural practice and global food security, the systemic nature of their action has raised concern, as this very property may inadvertently harm insects considered beneficial to humans.

Because neonicotinoid compounds can be present in all parts of a plant, it is plausible that nectar-collecting and pollen-harvesting insects may ingest low doses of the insecticide, potentially affecting their health and behaviour [15], [16]. Indeed, residual levels of neonicotinoids, as well as other synthetic compounds, have been detected in the nectar and pollen of pesticide-treated, flowering crops (reviewed in [15]).

Recent field studies showed that even low doses of neonicotinoid-contaminated food disturb the population dynamics of bumblebee (*Bombus terrestris*) colonies [17], [18], and weaken flight and homing performance of honeybees [19], [20]. The brood of honeybees fully depends on food collected by foraging workers. It is conceivable, then, that neonicotinoids could be imported into the hive via contaminated nectar and pollen from surrounding insecticide-treated crops. The consequences of larval exposure to low levels of this novel stressor have not been studied under field-realistic conditions.

Here we present and analyse molecular profiles obtained from worker-honeybee larvae after hives in the field were given access to syrup tainted with a low dose of the widely used neonicotinoid insecticide, imidacloprid. Genome-wide RNA transcriptional responses and lipid profiles provide insight into the physiological responses of developing organisms confronted with a new, real-world environmental threat.

## Results and Discussion

### Field-realistic Feeding Trials of Control and Imidacloprid-exposed Hives

To mimic honey flow of a neonicotinoid-treated nectar source in bloom, we provided colonies of free-foraging honeybees in the field an additional, imidacloprid-tainted source of food. Over a 15-day period, three experimental, imidacloprid-exposed (IE) hives received a daily ration of 100 mls syrup containing imidacloprid, while three control (C) hives received 100 mls of untainted syrup. We provided a concentration of imidacloprid (2 µg imidacloprid/L^−1^ syrup/≈2 parts per billion) that lies within the range (0.5 ppb–10 pbb) detected in contaminated pollen and nectar of a variety of crops (reviewed in [15]). During these 15-day feeding trials, all individuals within IE colonies, including egg-laying queens and the developing brood, will have come into contact with this insecticide through communal food exchange, a behavioural trait of social insects termed trophollaxis [21]. Following the 15-day feeding trials, we collected worker bee larvae for RNA transcriptome analysis and lipid profiling (Figure S1).

### Altered Expression of 15 microRNAs

MicroRNAs have emerged as modulators of cell physiological processes. We asked whether altered microRNA levels are detectable in imidacloprid-exposed larvae. From each of the three C and the three IE hives, respectively. RNA samples were isolated and pooled from whole larvae to establish six independent libraries for deep sequencing. Using statistical methodology implemented by edgeR and DESeq analysis [22] on the tag numbers of identified microRNAs, we established a list of 15 candidate microRNAs for which expression levels differed significantly (≥0.5 log_2_-fold change/p≤0.05; [23]) between the IE groups and C groups (Table 1, Data S1). Six of these microRNAs have been detected and annotated only in honeybees; their gene targets are presently unknown. The other nine microRNAs on the list are conserved in the evolutionarily distant fruit fly *Drosophila*. One of these genes is *mir-14*, which has diminished levels in the IE data set (−0.52 log_2_-fold change) (Table 1). In *Drosophila, mir-14* deficiency is associated with a variety of phenotypes, including a lower probability of larvae to survive development to adulthood, reduced adult lifespan, stress-sensitivity and abnormal energy metabolism [24], [25].

**Table 1:** DESeq-based expression levels identified, 15 annotated, mature candidate microRNAs (miRNAs), which show subtle differences in abundance between larval samples collected from imidacloprid-exposed (IE) hives and unexposed, control (C) hives.

Differential expression of microRNAs			
miRNA	logFC (IE/C)	PValue	reported in species
ame-mir-3783*	2.24	0.00	*Apis mellifera*
ame-mir-3720	1.20	0.00	*Apis mellifera*
ame-mir-92a*	1.13	0.02	*many animal phyla*
ame-mir-971	0.97	0.02	*Apis mellifera, Drosophila*
ame-mir-3783	0.85	0.04	*Apis mellifera*
ame-mir-965*	0.77	0.05	*Apis mellifera, Drosophila*
ame-mir-282	0.70	0.01	*Apis mellifera, Drosophila*
ame-mir-307*	0.67	0.05	*Apis mellifera, Drosophila*
ame-mir-3792	−0.51	0.05	*Apis mellifera*
ame-mir-14	−0.52	0.03	*Apis mellifera, Drosophila*
ame-mir-14*	−0.52	0.03	*Apis mellifera, Drosophila*
ame-mir-279c*	−0.55	0.04	*Apis mellifera, Drosophila*
ame-mir-989	−0.58	0.02	*Apis mellifera, Drosophila*
ame-mir-3747b	−0.80	0.01	*Apis mellifera*
ame-mir-3767	−1.10	0.00	*Apis mellifera*

Eight miRNAs were upregulated in IE samples, while seven miRNAs were downregulated. Conservation of miRNAs in species is indicated. Fold-change refers to log2-transformed-normalised data. MiRNAs designated with a * (star form) indicate the originating hairpin arm.

### Identification of Differentially Expressed Genes

**Figure 1:**
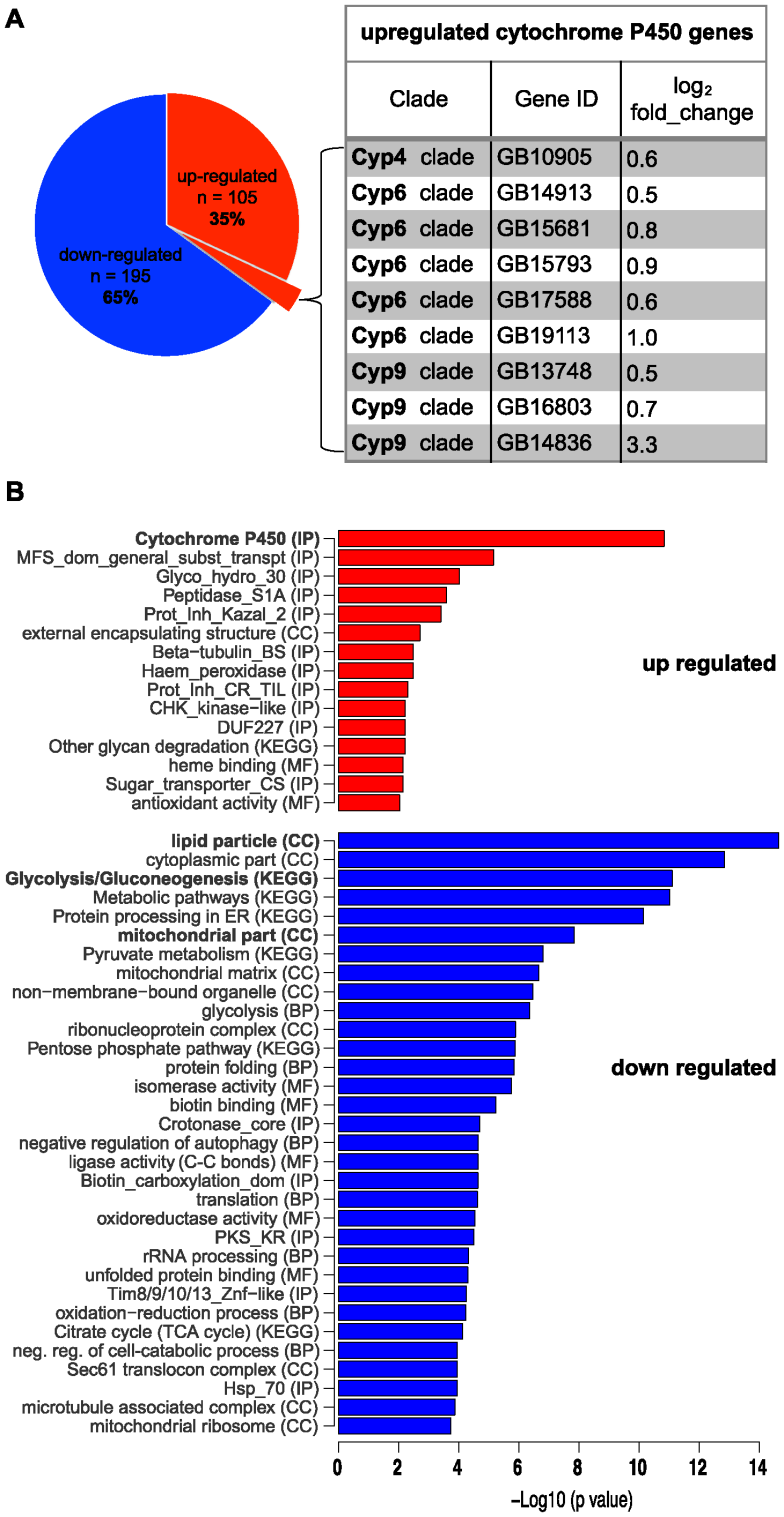
Functional annotation of differentially expressed genes. (A) Based on RNA-Seq data, expression levels for 300 genes were found to be significantly changed in imidacloprid-exposed larvae (compared with data obtained from non-exposed larvae). Expression is reduced for 195 genes (blue); expression is increased for 105 genes (red). The group of over-expressed genes includes nine cytochrome *P450s*; their gene IDs and normalised fold-change values are shown in the table. (B) Selection of non-redundant Gene Ontology (GO) terms for the three ontologies: Biological Process (BP), Molecular Function (MF) and Cellular Component (CC), and Interpro (IP) protein domains that are overrepresented in the 105 up (red) and 195 down (blue) regulated genes. Significance of enrichment is based on Fisher’s Exact Test. GO terms for the most significant enrichment groups are indicated in bold letters.

We next performed whole transcriptome sequencing (RNA-Seq) to explore whether steady-state levels of certain protein-encoding RNAs differ between worker larvae from C and IE hives. Following differential expression analysis using DEGseq (Table S1), we collated a list of 300 genes according to the DEGseq statistical tests FET, LRT, and MARS (p-value ≤0.001) and FC ≥0.5 (log_2_ normalised fold change) representing differences in RNA expression between samples C and IE (Table S2). Of this list, 65% (195/300) of IE genes have reduced RNA levels, while 35% of the IE genes (105/300) have elevated RNA levels relative to the same genes in the C group (Figure 1A, Table S2).

### Increased Expression of Lipid and Xenobiotic Metabolising Cytochrome *P450* Genes

Genes are conveniently categorised by their known functions and predicted biological roles. Such gene ontology (GO) analyses revealed that our list of differentially expressed RNAs is significantly enriched for genes operating in a lipid-carbohydrate-mitochondrial metabolic network (p<10^−5^/Figure 1B).

Within the overexpressed group of 105 transcripts, we find enrichment for nine genes of the cytochrome P450 monooxygenase superfamily (Figure 1A; Table S2). P450 (CYP) enzymes are oxidation catalysts of many cellular compounds, including lipids, steroid hormones and arachidonic acid metabolites. These enzymes also metabolise xenobiotic compounds and catalyse the breakdown of a wide range of structurally different toxins and synthetic insecticides. Overexpression of genes coding for the P450 clades (CYP4, CYP6 and CYP9) contribute considerably to insecticide-resistance [26]. In particular, filed and laboratory studies have shown a causal link between *Cyp6g1* overexpression and resistance to DTT and neonicotinoids [27]. Consistent with those results, RNAi mediated knockdown of *Cyp6g1* renders adult *Drosophila* more susceptible to imidacloprid [28].

Many insect *CYP/P450* genes appear to be under direct or indirect control of the nuclear receptor dHR96 in response to structurally diverse xenobiotics [28], [29], [30]. Knockdown of *dHR96* increases tolerance of adult *Drosophila* to imidacloprid exposure [28]. As dHR96 influences both gene activation and repression, imidacloprid tolerance could be mediated, in part, by upregulation of certain *CYP*/*P450* genes in these knockdown flies [28].

The nine upregulated honeybee *CYP/P450* genes in IE larval samples belong to the *CYP4, CYP6* and *CYP9* clades. It remains to be shown if altered transcription of these *CYP/P450* genes is a specific detoxification-response and whether the encoded enzymes are capable of metabolising imidacloprid. Further experimental studies will be required to establish whether honeybees have a functional orthologue of the *Drosophila* Cyp6g1, the CYP/P450 enzyme capable of protecting insects against the neonicotinoid insecticide. Compared with *Drosophila*, the honeybee genome carries s only around half the number of *CYP/P450* genes [31]. The shortfall of honeybee *CYP/P450* genes has been suggested to decrease the efficiency of detoxification processes, explaining the vulnerability of this pollinating insect to pesticides [32].

### Genes Involved in Lipid Metabolism

Our finding of changes in mRNA levels for fatty acid metabolising enzymes suggests that lipid homeostasis is altered in imidacloprid-exposed larvae (Figure 1B). For example, reduced transcriptional activities are seen for fatty acid synthase (*FAS*), a glycerol-3-phosphate acyltransferase (*GPAT*) and the ATP citrate lyase, *ATPCL* (Table S2; Data S3). These three genes are downregulated in adult *Drosophila* as part of an immune response to infection; their lowered expression levels reflects energy reallocation, a process proposed to be controlled by the FOXO signalling pathway [33].

FOXO might be involved in regulating the aforementioned nuclear receptor dHR96 in *Drosophila* [34]. Besides responding to xenobiotic stresses, dHR96 also plays a key role in lipid and cholesterol metabolism, in adult *Drosophila* [30], [35], [36]. We examined if some of the 300 differentially expressed genes in IE honeybee larvae could be orthologues of lipid-metabolising genes found to be dHR96-regulated in *Drosophila*. Potential dHR96-regulated genes are GB12567, GB17220 and GB10584, encoding a predicted long-chain fatty acid-CoA ligase, a Lip3-like lipase and an enzyme involved in sphingolipid metabolic processes, respectively. That is, we only observe a limited overlap between our honeybee data set of genes taking part in lipid-metabolism and that reported for dHR96-regulated lipid genes in *Drosophila*. However, there was an overrepresentation of *Drosophila* orthologues that are upregulated (P<.004) and downregulated (P<10–9) by CncC, the cap ‘n’ collar isoform-C regulator of xenobiotic detoxification responses in *Drosophila* [37].

Another transcription factor that may be involved in the response to imidacloprid-exposure is the Hepatic Nuclear Factor 4 (HNF4) as we identified 15 orthologues of transcripts that show altered expression in *Drosophila* dHNF4 mutants [38]. Amongst them are the downregulated AcetylCoenzyme A synthase (AcCoAS) and an acetyl-CoA carboxylase (ACC) and two upregulated Lip3-like lipases (GB17220, GB17745) (Table S2). Elevated levels of Lip3, encoding a predicted cholesterol ester hydrolase, have been observed in starved *Drosophila* larva [39]. The dHNF4 transcription factor is activated in response to starvation or exogenous long-chain fatty acids and it has been suggested that dHNF4 senses free fatty acid levels in the fly larva and drives lipid mobilization and catabolism through lipolysis and β-oxidation in the mitochondria [38]. Together, these findings indicate that imidacloprid-exposure triggers changes in gene expression affecting various facets of larval lipid metabolism.

### Concerted Effect on Glycolytic and Carbohydrate-metabolising genes

**Figure 2:**
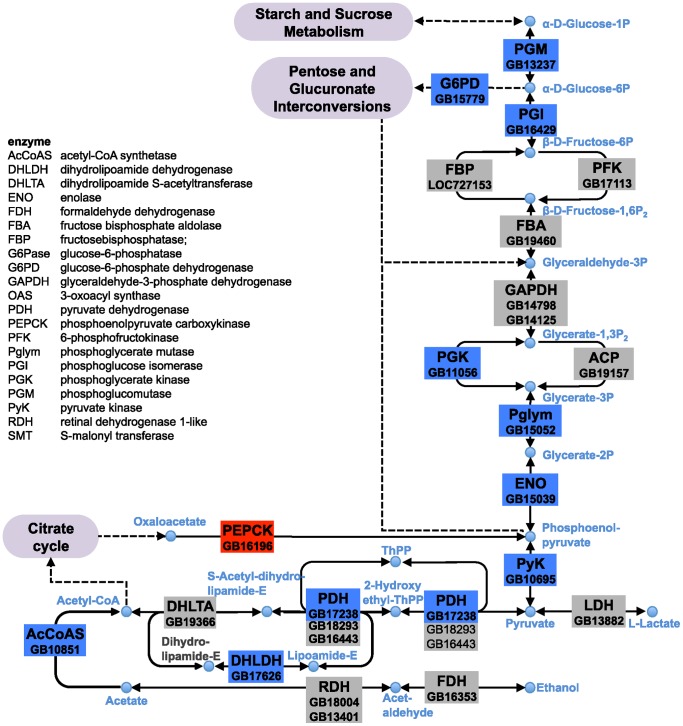
Expression of genes encoding carbohydrate-metabolising enzymes is affected in imidacloprid-exposed worker bee larvae. Genes/enzymes, including paralogues, and their positions (coloured/grey boxes) in the glycolytic and related carbohydrate pathways are placed with reference to honeybee-specific pathway variations [72], [73]. Based on DEGseq analysis, ten genes are downregulated (blue), including *PGI, PGK, PGLYM*, *ENO* and *PYK*, of the glycolytic pathway; *PEPCK* is upregulated (red). A key for gene names is provided; fold-changes in expression as determined by RNA-Seq (IE relative to C data set).

Balancing energy requirements under varying conditions necessitates coordination of lipid and glucose-metabolising pathways. We found that genes of the gluconeogenesis and glycolysis pathways have altered expression upon imidacloprid exposure (Figure 2). Increased expression is detected for the stress-responsive phosphoenolpyruvate carboxykinase (*PEPCK*), a gene that regulates glucose levels by catalysing the first step in gluconeogenesis. In *Drosophila,* upregulation of *PEPCK* is induced by xenobiotic insults [29] and under fasting conditions [39], the latter involving the FOXO signaling pathway [40].

Within the group of 195 downregulated mRNAs in the IE data set, we identified a cluster of sugar metabolising genes; five of them encode core enzymes of the glycolytic pathway (Figure 2). To better understand the potential interactions between the up- and downregulated genes, we used the gene lists as input in the online database resource Search Tool for the Retrieval of Interacting Genes (STRING) program. Analysis of the downregulated genes revealed a highly connected network with more than twice the number of putative interactions expected by chance (377 observed, 153 expected, P = 0). Our analysis showed strong links between the down regulation of genes involved in sugar metabolism and RNA translation in both the cell and the mitochondria (Figure S2). This STRING analysis further supports our discovery of a concerted, non-random down regulation of metabolic processes in IE larvae. That is, the reported transcriptional changes for the genes in our list cannot be explained by normal variation in expression found among bee colonies.

While transcriptional changes of individual glycolytic genes have little effect on glucose flux [41], several reports indicate that coordinated changes in their expression levels occur as part of the physiological response to starvation. Diminished expression of genes involved in glycolysis has been observed in nutrient deprived *Drosophila* larvae [38] and can be modulated by at least two different transcription factors: the ecdysone receptor (EcR) and the estrogen-related receptor (dERR), respectively [42], [43]. The *Drosophila ERR* is an essential regulator of carbohydrate metabolism and a deficiency in mutant *Drosophila* larvae is associated with diminished ATP and triacylglyceride (TAG) levels [43].

The overall altered transcription profile of the IE data set (Figure 1), echoes the observations reported for xenobiotic stress responses in *Drosophila* [29]. Treatment of fruit flies with the drug phenobarbital is also associated with deregulation of genes involved in energy and sugar metabolism [29].

### Possible Participation of Myc-mediated Gene Expression

Coordinated responses of metabolic gene networks to a variety of different environmental stimuli suggest genome-wide, synchronised regulation by one or more transcription factors. We applied ‘Clover’, a program developed to identify statistical overrepresentation of functional DNA sequence motives [44] to the promoters of our 300 differentially expressed protein-coding genes (Data S2). A significant proportion of genes (19% [56/300]) contain the canonical enhancer box (E-box) sequence CACGTG, a DNA element recognised by the Myc family of transcription factors [45]. For the majority of genes containing this sequence motive (73% [41/56]), expression levels are diminished in the IE group (Data S2).

We note that six of the ‘E-box genes’ have *Drosophila* orthologues that carry the same promoter DNA element; their expression has been shown to be TORC1-regulated and dMyc-dependent (Data S2) [9], [46]). One of the factors influencing the activity of dMyc in the fat body is ecdysone [47]. Elevated levels of this steroid hormone and its receptor repress dMyc; this leads to altered expression of Myc-target genes and a slowed larval growth rate [47].

Among the many potential genomic targets, Myc has been implicated in the control of nuclear-encoded genes affecting mitochondrial function and activity [48]. We find six of these genes to be downregulated in the IE-Myc data set; they encode mitochondrial membrane proteins Tim8, Tim9a, Tim13 and bor/dATAD3A, as well as the mitochondrial ribosomal proteins mRpL9 and mRpL12 (Data S2 **)**. Cells of a fruit fly lacking the protein mRpL12 have an impaired growth rate and reduced mitochondrial activity [49]. The cluster of downregulated mitochondrial genes in our ‘E-box list’ suggests that the function of this energy-producing organelle could be affected in imidacloprid-exposed larvae.

### Decreased Expression Levels of *Hsp90*

**Figure 3:**
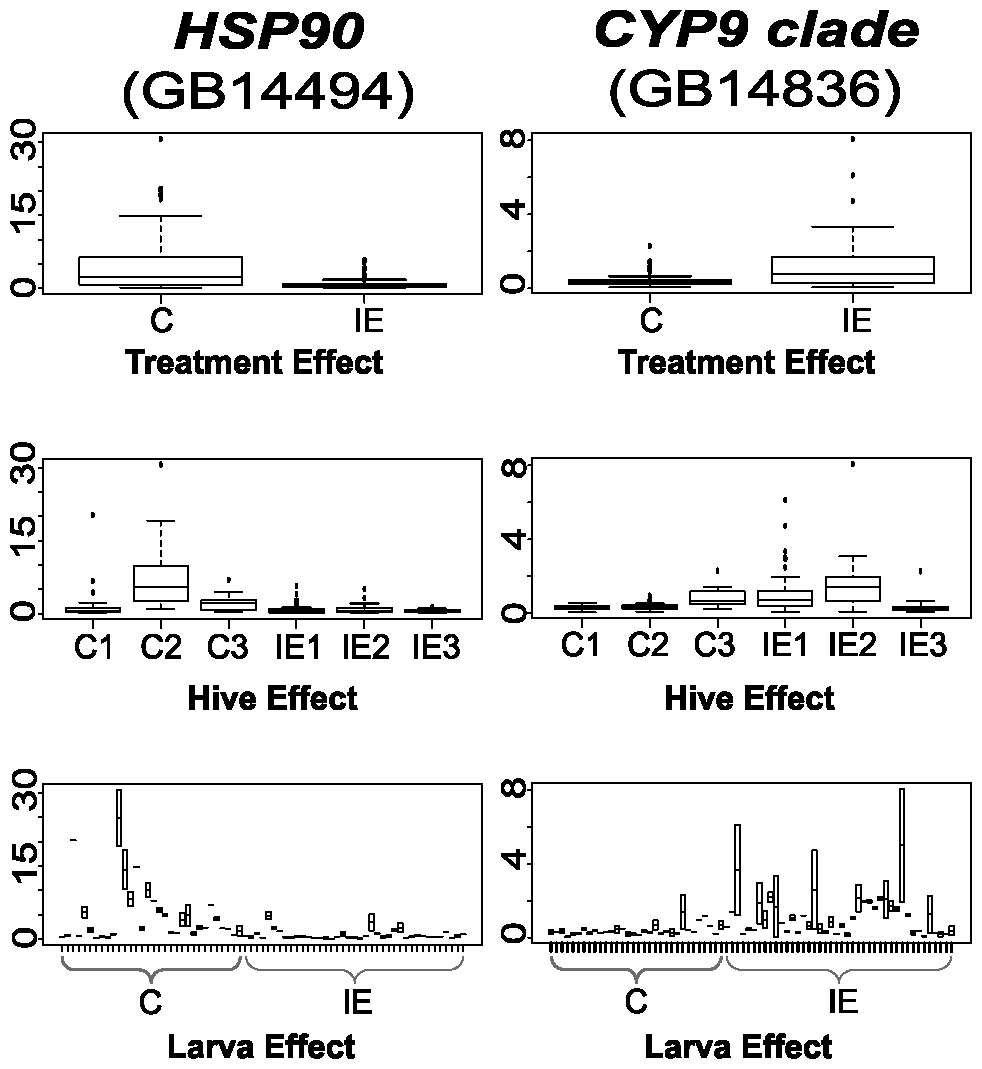
Multiplex RT-PCR validation of *HSP90* and a *P450* gene (*CYP9*-clade). Variance in measured RNA expression levels was assessed between treatment groups (left), among hives (middle) and between the two measurements taken for individual larvae (right/larval samples 1–34 came from control hives C1–C3; Larval samples 35–74 came from imidacloprid-exposed hives IE1–IE3. As two measurements were made for each larva, the height of each bar in the larva plot gives the range of values recorded; the mean value for each larva is indicated in the middle of this range. For those larvae that yielded only a single successful measurement for a given gene, only one point is shown. Box and whisker plots show 25th percentile (bottom of box), 50th percentile (middle of box), 75th percentile (top of box), median (line in middle of box), and full range of data (low bar to high bar). Relative expression levels are compared to a standardisation factor. The scale of the y-axis differs between plots for the two genes and indicates fold-difference for the expression level relative to the standardisation factor.

The developmental Hsp90 buffering system is versatile. It stabilises misfolded and metabstable protein complexes [50], suppresses the activity of transposable elements [51] and is involved in establishing altered chromatin states [52]. Levels of the ubiquitously expressed *Hsp90* gene are normally high. This ensures immediate buffering capacity if demand exceeds the required basal level [6], [53]. In *Drosophila, Hsp90* is one of the TORC1/dMyc-regulated genes that contain a canonical E-box element in the promoter region [9]. The honeybee *Hsp90* promoter carries the variant E-Box sequence CACATG, which may also be responsive to Myc. Our RNA-Seq expression data indicate reduced levels of *Hsp90* transcripts in IE larvae (Table S2). Overall diminished *Hsp90* RNA levels in IE larvae was also detected by multiplexed RT-PCR using additional larval samples (Figure 3). Reduction of Hsp90 levels would be a strategically sound response to a new environmental stressor. It allows the release from – and expression of – pre-existing cryptic genetic and epigenetic variants. Some of these variants may improve the chances of normal larval development, while others will increase imidacloprid sensitivity, potentially leading to larval death. If *Hsp90* downregulation upon imidacloprid exposure is a common response, the genetic background may prove to be an important factor in determining the fate of a bee colony. Inbred populations of fruit flies, for example, with slightly decreased *Hsp90* expression levels are less fecund and shorter-lived than populations with normal *Hsp90* levels [54]. Together, our finding of reduced *Hsp90* expression implies that imidacloprid-exposure affects the developmental buffering system.

### Measuring RNA Levels in Samples of Individual Larvae

We sought to validate results that had been obtained by RNA-Seq. From the list of 300 differentially expressed genes, a set of 17 genes was selected for analysis, including *Hsp90* and members of the cytochrome P450 clades *CYP6* and *CYP9* (Figure 3; Data S3). We employed a multiplexed, quantitative gene expression analysis system (GenomeLab GeXP/Beckman Coulter [55]. This approach allowed us to simultaneously measure relative mRNA levels of the 17 genes in a given RNA sample and perform ANOVA on expression data. RNA-expression levels were standardised by the geometric mean of three genes (*Actin, Ubiquitin* and GB19767), whose expression levels were relatively consistent. Altogether we analysed 74 RNA samples, each isolated from an individual larva. Of these, 34 larvae were collected from the three C hives, and 40 from the IE hives. We asked about the significance of variation in RNA expression levels found to exist between repeated measurements for the same larva, among hives, and between IE and C groups. Three genes, *Hsp90, FAS* and *G6PD*, respectively, were found, following the conservative Bonferroni correction [56] to exhibit marginal significance for variation among C and IE larvae. Thus, differences between measures of individual genes from C and IE groups are subtle, but overall in good agreement with the results obtained by RNA-Seq (Figure 3; Data S3). Given that even genetically identical organisms can have pronounced fluctuations in the expression of individual genes [57], we were not surprised to detect varying expression levels among individual larvae from heterogeneous, wild-type bee populations. The genetic background – reflected by hive-impact – contributes to variations in expression levels of some genes (i.e. Cyp6/GB19113 in Data S3).

### Changes in the Composition of Lipid Compounds

**Figure 4:**
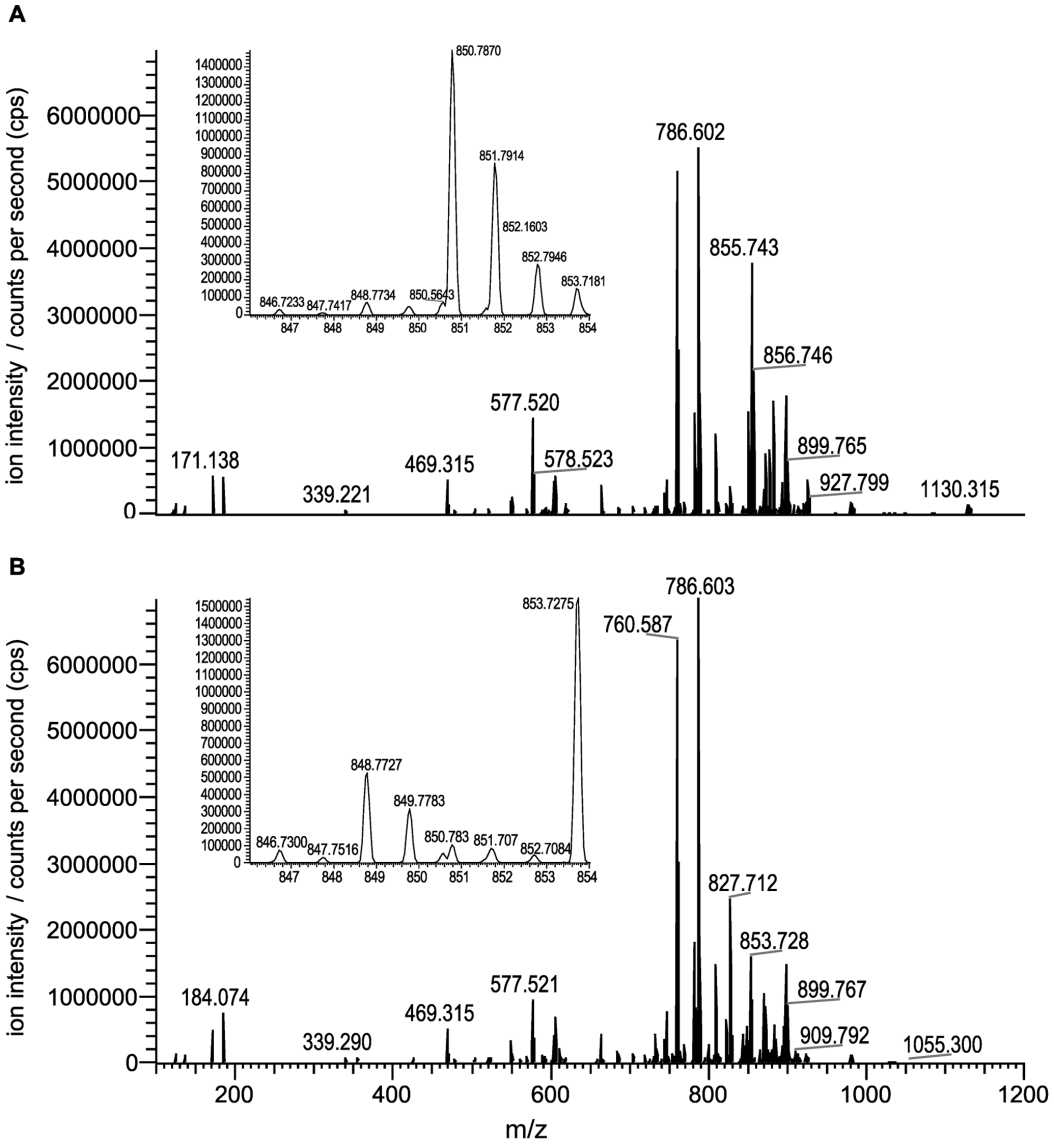
Exemplary data of high resolution LC-MS analysis of larval lipid extracts. Shown are graphic representations of summed spectra (positive electrospray mode) from a larval sample of an imidacloprid-exposed hive (A) and a larval sample of an unexposed, control hive (B). Y-axes (ion intensity) are normalised to the most intense ion species; x-axes indicate the specific mass-to-charge ratio (*m/z*). Smaller insets in A and B are zoomed-in portions of the graph axis scale (*m/z* 840–855) and provide an example of a typical change at *m/z* 850.587, which is elevated in imidacloprid-exposed samples. While some differences between the spectra can be readily observed, data processing enabled more detailed analysis.

Analysis of our RNA-Seq data point towards altered lipid metabolism in imidacloprid-exposed larvae (Figure 1). To find possible unbiased differences in lipid composition, 20 individual lipid profiles were established from extracts of ten C and IE larvae, respectively. For this, we used liquid chromatography coupled with mass spectrometry (LC-MS) analysis. We detected a total of 1638 lipid metabolites with strong MS signals (threshold >1000 cps) that were present both in C and IE samples. Of these, 247 metabolites (15%) showed significant differences in ratios between C and IE samples (P<0.001, Student’s T) (Figure 4; Table S3). While identification of lipid species on the basis of accurate mass of the ion is not definitive, we classified around 27% of the compounds (68/247) in this list. Species from many lipid classes showed altered levels, including diacylglycerols (DAG), triacylglycerols (TAG), ceramides (CE), phosphoethanolamines (PE), phosphocholines (PC), phosphoserines (PS), phosphoinositol (PI) and free fatty acids (FFA) (Table S3). Thus, altered abundance is not restricted to lipid species directly involved with energy metabolism. Constituents of cell membranes, such as PC and PE lipid species, also differ in relative quantities between IE and C larvae. Small changes in lipid composition of cell membranes could alter the structure and function of membrane-embedded proteins [58]. Functional nAChRs – the molecular target site of neonicotinoids – reside in the plasma membrane as ligand-gated ion channels, composed of five protein-subunits [59]. *In vitro* studies demonstrate that drug action at nAChRs is influenced by the composition of membrane lipids [58]. Altered abundance of certain membrane lipid species may therefore be a physiological adjustment that counters nAChR-mediated effects of imidacloprid.

### Concluding Remarks

We note that levels of protein encoded by genes reported here to be differentially expressed in IE larvae have not been measured. Interpretations of our results are therefore limited. We currently do not know how changes in RNA expression levels translate into protein levels. Nevertheless, altered lipid profiles in IE larvae are indicative that proteins – expressed by genes involved in lipid-metabolism – exert altered activity.

Given the changes we observe at the RNA and lipid level, it is reasonable so suspect that the synthetic neonicotinoid-class of insecticides are a factor driving the global decline of pollinating insects. Species risk extinction if they fail to adjust effectively to the demands of a changing or new environment. We find evidence that dietary traces of the insecticide imidacloprid impacts physiology of larvae from bee colonies located in a typical British agricultural landscape. The genomic response to this novel environmental stressor mainly affects energy metabolism pathways. A probable involvement of Myc-regulated gene networks is suggestive of an altered growth rate of imidacloprid-exposed larvae. These findings, in concert with the detected decrease of *Hsp90* expression may be interpreted as symptoms of a strained developmental buffering system. That is, larvae still grow and develop in the presence of the novel stressor although the stability of the developmental process is compromised. Depending on the genetic background, additional stressors would likely cause an increased rate of developmental failure.

Identifying that low levels of a neonicotinoid influences energy metabolism in worker bee larvae raises the question on the generality of our finding. How persistent is the effect? Recent evidence suggests that exposure in early life can influence associative ability of the adult honeybee workers [60]. Does insecticide-exposure alter expression of the same set of genes in adult pollinating insects? Downregulation of sugar metabolism in response to neonicotinoids could, for example, impact start and duration of foraging [61], [62] and impair flight performance as flight muscles work at very high glycolytic rates [63], [64]. In fact, it has already been observed that treatment of adult honey bees with imidacloprid can impair foraging and result in delayed return flights and an increase in the number of bees not returning from foraging [65]. Our study suggests that the pollinators’ struggle to adjust to new environments can be influenced by anthropogenic activities.

## Materials and Methods

### Apiary/Experimental Setting in the Field

Healthy, well-established, queenright honeybee colonies came from the Nottinghamshire region in England; the bees are likely hybrids of the Carniolan honeybee (*Apis mellifera carnica*) and the European dark bee (*Apis mellifera mellifera*). Six hives were divided into two groups: the control group (C1, C2, C3) and the experimental group (IE1, IE2, IE3), respectively. Detailed information is provided in Figure S1. Crops grown in the two fields immediately surrounding the C and IE hives had not been treated with imidacloprid. However, a variety of different agrochemicals had been applied to these fields before and during the 2010–2011 time period when the feeding trials were conducted (Table S4). The probability of exposure to these chemicals was the same for C and IE hives.

### Feeding of Sugar Syrup Spiked with Imidacloprid and Distribution of Syrup within the Hive

*Ambrosia*, commercially available beefood syrup was used as feed. Two, 15-day long feeding trials were performed: in July 2010 and in May 2011. A 20 mg/L^−1^ stock solution of imidacloprid (Sigma-Aldrich UK; dissolved in H_2_O and stored, light-protected, at 4°C) was used for the daily preparation of a syrup containing 2 µg/L^−1^ imidacloprid. The daily 100 ml syrup portions were provided afternoons between 12∶00 and 14∶00 and worker bees consistently emptied the feeders within 24 hours.

### Larval Sample Collection

15–20 worker bee larvae were collected from open (uncapped) cells. To minimise circadian rhythm-induced differences in RNA expression levels, larvae were collected between hours 13∶30–15∶00. Larvae were immediately immersed in ice-cold RNAlater solution (Sigma-Aldrich, UK) and subsequently stored at −80°C, pending RNA or lipid analysis. Since larval collection took place under field conditions, it was not possible to accurately match the developmental stage between larvae; overall we removed larvae that – by size - appeared to be at day 6–9 day of development (day 1 = laying of fertilised egg).

### RNA Isolation for Deep Sequencing

Worker bee larvae collected in July 2010 were used to isolate total RNA (MirVana kit (Ambion). We used pooled homogenates of whole larvae (n = 5–7) from each hive. Weights ranged from 70 mg –120 mg (Table S5). For micro RNA sequencing, the RNA samples represented the six individual hives. For whole transcriptome sequencing (RNA-Seq), we pooled total RNAs in equal amounts representing two hives (C1/C3 and C2/C3, respectively) and (IE1/IE3 and IE2/IE3, respectively).

### MicroRNA Sequencing and Analysis

The protocol to profile small RNAs in the larval samples essentially followed the procedure and workflow described in [66]. Six sequencing libraries were generated (SREK Rev. B, Applied Biosystems). The obtained cDNA libraries were PCR amplified, purified, and size-selected by PAGE, resulting in six libraries containing inserted small RNA (20–40 bp); verified by the Agilent 2100 Bioanalyzer (Agilent Technologies). cDNA libraries were amplified in a pool onto beads using emulsion PCR; templated beads were deposited on a full slides and analysed using the Applied Biosystems SOLiD-4 Sequencer. Each of the six RNA libraries was twice sequenced (two technical replicates), yielding a total of ∼246.5 million sequenced reads that passed quality control. A digital expression profile (DEG) was established for 342 annotated, precursor and mature microRNAs present in all C and IE data sets (Data S1). Using the edgeR Bioconductor statistical analysis package [22] on the annotated read counts generated by the mapping of the small RNA sequences, filtered for known insect non-miRNA sequences, versus the mature and precursor sections of the Mirbase database (version 18) and, separately, versus the honeybee genome (Amel 4.0) and related known *A.mellifera* miRNA mapping information, establishing a count-based, digital expression profile (DEG).

### RNA-Seq Library

For each of the four libraries, approximately 10 µg of Total RNA was used to enrich for mRNA [two rounds of enrichment with Dynalbeads Oligo(dT)_25_ (Life tech, 61005)]. SOLiD whole transcriptome libraries were made according to the SOLiD Total RNA-Seq protocol (Life tech, 4445374). Briefly, enriched mRNA was fragmented using chemical hydrolysis followed by phosphorylation with T4 PNK and column purification using Purelink RNA Micro Kit (Life tech, 12183-016). cDNA synthesis was carried out after adaptor ligation. cDNA libraries were amplified following size selection and adaptor removal. Equimolar pools of RNA-seq libraries were made following qPCR quantification using Kapa Library Quantification kit (Kapa Biosystems, KK4823). ePCR and templated bead enrichment was carried out with Solid EZ bead system according to manufacturers preparation guide. Enriched beads were sequenced on an ABi SOLiD 4 analyser according to the manufacturer’s instructions to generate 50 bp reads in colour space. We generated four libraries: two libraries represented the transcriptomes of C larvae (n = 10–14) (pooled RNA of C1/C3 and C2/C3, respectively) and the other two representing the transcriptomes of IE larvae (n = 10–14) (pooled RNA IE1/IE3 samples and IE2/IE3, respectively).

### Read Mapping and Differential Gene Expression Calculations

A total of 183 million 50 bp single reads were obtained in total for the 2 sample groups C and IE. A total of 33.4 million and 33.1 million reads for the group C replicates, and 34.8 million and 116.5 million for the group IE replicates. Read filtering and genome mapping was performed using the BioScope 1.3 (Life Technologies) whole transcriptome pipeline. Reads where initially filtered against sequences representing *A. mellifera* tRNA and rRNA and sequencing library artefacts. Filtered reads were then mapped to the *A. mellifera* genome (Amel 2.0) and guided with the Ensembl (version 13) gene annotation (GTF); a total of ∼103 million uniquely mapped SOLiD reads were identified against the honeybee reference genome. Congruency of normalised gene expression values (RPKM) between hive sample replicates through R-squared calculations yielded values≥0.95 for all comparisons; RPKMs were calculated from the uniquely aligned read counts per gene using htseq-count (http://www-huber.embl.de/users/anders/HTSeq). Differential gene expression analysis was conducted using the R package DEGseq [67]. The 4 separate differential expression tests implemented by DEGseq and the corresponding significance thresholds used were: Likelihood Ratio Test (LRT): *p*-value≤0.001, Fisher’s Exact Test (FET): *p*-value ≤0.001, MA-plot-based method with Random Sampling model (MARS): *p*-value≤0.001, and Fold-Change threshold on MA-plot (FC): log_2_ normalized fold change≥0.5. Sequencing reads used in this study are publically available through the EBI Sequence Read Archive (SRA) under accession number ERP001796 (http://www.ebi.ac.uk/ena/data/view/ERP001796).

### Functional Annotation

Gene Ontology annotation was taken from BioMart in Ensembl [68] for *Drosophila melanogaster* genes and used to annotate *Apis mellifera* genes. Annotation was taken only when there was a one-to-one orthology relationship between fly and bee genes. Interpro protein domain annotation for *Apis mellifera* genes was taken from Ensembl Metazoa [69].

### RT-PCR: Multiplex-quantitative Gene Expression Analysis (GenomeLab GeXP/Beckman Coulter

Individual bee larvae were grouped according to size (50 to 120 mg), hive and treatment. Total RNA was extracted from individual bee larvae (Qiagen kit). RNA was treated with Turbo DNaze (Ambion) and diluted to a concentration of 30 ng/ml. A multiplex PCR (GeXP Beckman Coulter) combing gene-specific and universal priming strategy was used to validate the expression of genes indicated by RNA-Seq as affected by treatment. We used 2 subsequent multiplexes. The first multiplex-PCR approach was used to assess suitability of Housekeeping (HK) genes for normalisation and to evaluate removal of – or the potential presence - of DNA contamination. Following genes had the most stable, invariable, expression levels and were used in the 2nd multiplex as normalization factor: *Actin* (GB17681), Polyubiquitin-A-like (GB16469) and an uncharacterised gene (GB19767). Gene expression data were collected according to Beckman GeXP protocol. All primer-pairs were evaluated individually; their concentrations subsequently adjusted for the multiplex RT-PCRs. 60 ng of total RNA was used in individual RT reactions with 20 reverse primers and amplified in subsequent PCRs according to instructions of the manufacturer (Beckman Coulter). As an internal control, RNA coding for *Kanamycin* was included in all reactions. Data were processed with the GeXp Profiler module.

### Statistical Analysis of the Multiplex-quantitative Gene Expression Data

The R language [70] was used to develop codes for GExP expression analysis.

### Lipid Analysis by LC-MS

Larvae collected from the 2011 feeding trial were used for lipid profiling. 3 or 4 individual larvae representing each hive (in total 10 IE and 10 C). Larvae sample preparation: The 50 mg samples were freeze-dried and stored at −80°C extraction. Larvae were ground with a micropestle in methanol and the lipid components were extracted using a chloroform:methanol method based on the Bligh and Dyer technique [71]. The lower organic layer was removed, centrifuged to remove all debris and again stored at −80C until LC-MS analysis.

Accurate mass LC-MS was performed on the extracted lipids (10 µL injection volume) using an Accela LC and Exactive mass spectrometer (ThermoFisher Scientific) in ES+ and ES– modes. The LC column used was a ThermoFisher Gold C18 (1.8 µm, 2.1×100 mm) and eluted using a water (A)-to-acetonitrile gradient (B) modified with 0.01% formic acid (25%–100%B over 8 min), flow rate 0·3 mL/min. Ions were monitored the range *m/z* 100 to 1500. The calibrated mass accuracy was 1 (ES+) and 2 (ES–) milli mass units and the resolution was 10–25,000 FWHM. A serial dilution of the pesticide imidacloprid was created from 20 ng to 200 µg/ml to check the lower limits of detection. The proprietary software Sieve 2.0 (ThermoFisher Scientific) was used to align and bin the data sets and to highlight any differences. LipidMaps (http://www.lipidmaps.org/data/standards/index.html) and Metlin databases were used to assist in identification of extracted lipids. The LipidMaps prediction tool was also used to generate the theoretical masses of product ions which in conjunction with the fatty acid residues, was used to assign identities.

## Supporting Information

Figure S1 **Flow chart.**
(PDF)

Figure S2 **STRING analysis.**
(PDF)

Table S1 **DEGseq data.**
(PDF)

Table S2 **List of 300 differentially expressed genes.**
(PDF)

Table S3 **Lipid profiles: LC/MS data.**
(PDF)

Table S4 **List of agrochemicals applied to fields.**
(PDF)

Table S5 **Larva.**
(PDF)

Data S1 **microRNA data.**
(XLSX)

Data S2 **Myc gene list.**
(XLSX)

Data S3 **Box plots of 17 genes/GeXP-based multiplex RT-PCR data.**
(PDF)
