# Supplementary material for: Transient Exposure to Low Levels of Insecticide Affects Metabolic Networks of Honeybee Larvae
Source: PLoS One. 2013 Jul 2;8(7):e68191. doi: 10.1371/journal.pone.0068191 (PMC3699529; doi:10.1371/journal.pone.0068191)
Supplement: Figure S1 — Flow chart. (PDF) [file pone.0068191.s001.pdf]

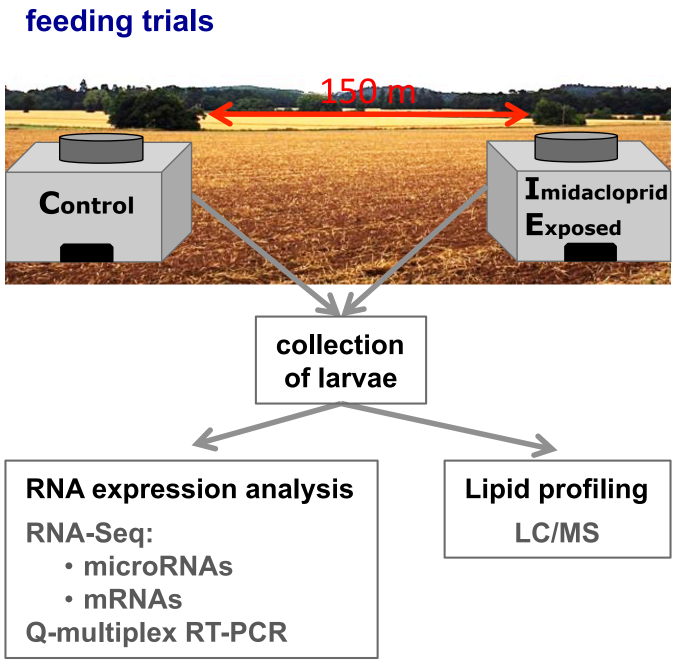

Figure S1
Schematic flow chart of field-realistic imidacloprid feeding trials and subsequent molecular profiling of worker-honeybee larvae.
Feeders (flat cylinders) were used to provide syrup (control / C) or imidacloprid-tainted syrup (Imidacloprid-Exposed / IE). The feeders were only accessible for bees from within the hives. RNA and lipid profiles were established and analysed for larvae of the 'C' and 'IE' hives. The background photo shows the actual location, where the hives were kept.
Well-established, queenright colonies were kept in hives built to the specifications issued by the British Standards Institution (the British national hive; a Langstroth-class hive). Brood chambers were covered with a queen excluder and crown board; feeders with a 17 cm diameter were placed on top of the crown board and covered by a roof. Thus, the syrup provided in the feeders was only accessible to worker bees from within the hive. Six hives were divided into two groups: the control group (C1, C2, C3) and the experimental group (IE1, IE2, IE3), respectively. The 'C' and 'IE' hives were kept 150 metres apart in two small, lightly wooded areas (~ 400 m^2), surrounded by farmland and fields. Ground cover weed fabric was placed in front of the hive entrances to facilitate detection of a possible accumulation of sick and dead bees. The feeding regime was performed two times. During spring, all hives were treated with 'ApiLifeVar®,' as a routine measure to prevent *Varroa destructor* mite infestation.

Transient exposure to low levels of insecticide affects metabolic network of honeybee larvae. Derecka et al. 2013
