# Supplementary material for: Transient Exposure to Low Levels of Insecticide Affects Metabolic Networks of Honeybee Larvae
Source: PLoS One. 2013 Jul 2;8(7):e68191. doi: 10.1371/journal.pone.0068191 (PMC3699529; doi:10.1371/journal.pone.0068191)
Supplement: Table S1 — DEGseq data. (PDF) [file pone.0068191.s003.pdf]

**TABLE S1 DEGseq data**	Table showing Poisson distribution MA-plot based method for detecting differentially expressed genes from normalized Fold-Change “FC”

Transient exposure to low levels of insecticide affects metabolic networks of honeybee larvae. Derecka et al. (2013)

Gene ID	Sample IE read count	Sample C read count	log2(Fold_change)	log2(Fold_change) normalized	Signature(abs(log2 (Fold_change)
GB10001	1	0	NA	NA	FALSE
GB10002	1109	558	0.990922338	0.12640483	FALSE
GB10003	13359	9060	0.560229062	-0.304288446	FALSE
GB10004	830	467	0.829688787	-0.034828722	FALSE
GB10005	1581	915	0.788993719	-0.075523789	FALSE
GB10006	669	539	0.311720938	-0.55279657	TRUE
GB10007	2100	906	1.212806373	0.348288864	FALSE
GB10008	1772	863	1.037946139	0.173428631	FALSE
GB10009	456	227	1.006341527	0.141824019	FALSE
GB10010	3602	1833	0.974591395	0.110073887	FALSE
GB10011	NA	NA	NA	NA	FALSE
GB10012	239	107	1.159399822	0.294882313	FALSE
GB10013	1195	600	0.993976212	0.129458704	FALSE
GB10014	243	145	0.744903414	-0.119614095	FALSE
GB10015	NA	NA	NA	NA	FALSE
GB10016	724	511	0.502666406	-0.361851102	FALSE
GB10017	280	122	1.198545679	0.334028171	FALSE
GB10018	11121	4098	1.440294632	0.575777123	TRUE
GB10019	8	2	2	1.135482492	TRUE
GB10020	67	38	0.818161677	-0.046355831	FALSE
GB10021	217	106	1.033630778	0.16911327	FALSE
GB10022	1	1	0	-0.864517508	TRUE
GB10023	267	148	0.851242566	-0.013274942	FALSE
GB10024	1370	660	1.053637964	0.189120455	FALSE
GB10025	3983	2078	0.938659825	0.074142317	FALSE
GB10026	2600	1384	0.90966768	0.045150172	FALSE
GB10027	NA	NA	NA	NA	FALSE
GB10028	2156	1114	0.952607945	0.088090437	FALSE
GB10029	7395	3618	1.031357739	0.166840231	FALSE
GB10030	5393	3468	0.636984135	-0.227533373	FALSE
GB10031	NA	NA	NA	NA	FALSE
GB10032	1486	763	0.961679154	0.097161645	FALSE
GB10033	1017	526	0.951184975	0.086667466	FALSE
GB10034	177	101	0.809394067	-0.055123441	FALSE
GB10035	838	496	0.756610123	-0.107907385	FALSE
GB10036	NA	NA	NA	NA	FALSE

GB10037	211	111	0.926683322	0.062165814	FALSE
GB10038	97	59	0.717269793	-0.147247716	FALSE
GB10039	162	112	0.532495081	-0.332022428	FALSE
GB10040	15	5	1.584962501	0.720444992	TRUE
GB10041	232	118	0.975337946	0.110820437	FALSE
GB10042	1420	829	0.776446923	-0.088070585	FALSE
GB10043	1020	481	1.084460353	0.219942845	FALSE
GB10044	4953	2690	0.880696448	0.01617894	FALSE
GB10045	902	597	0.595396502	-0.269121006	FALSE
GB10046	745	404	0.882885133	0.018367624	FALSE
GB10047	511	548	-0.100852602	-0.96537011	TRUE
GB10048	37	31	0.255257055	-0.609260453	TRUE
GB10049	538	309	0.799999335	-0.064518174	FALSE
GB10050	598	320	0.902073579	0.037556071	FALSE
GB10051	180	87	1.0489096	0.184392092	FALSE
GB10052	811	515	0.655129482	-0.209388026	FALSE
GB10053	462	285	0.696930932	-0.167586576	FALSE
GB10054	1448	942	0.620262637	-0.244254871	FALSE
GB10055	904	652	0.471450808	-0.3930667	FALSE
GB10056	463	373	0.311836563	-0.552680945	TRUE
GB10057	4	2	1	0.135482492	FALSE
GB10058	841	450	0.902180799	0.037663291	FALSE
GB10059	154	79	0.963005793	0.098488284	FALSE
GB10060	1130	974	0.214329095	-0.650188413	TRUE
GB10061	102	44	1.212993723	0.348476215	FALSE
GB10062	65	50	0.378511623	-0.486005885	FALSE
GB10063	1524	806	0.919011159	0.054493651	FALSE
GB10064	2	0	NA	NA	FALSE
GB10065	136	84	0.695145418	-0.16937209	FALSE
GB10066	54	14	1.94753258	1.083015072	TRUE
GB10067	1190	590	1.012174714	0.147657206	FALSE
GB10068	292	202	0.531613076	-0.332904432	FALSE
GB10069	1397	709	0.978474488	0.11395698	FALSE
GB10070	804	276	1.542527234	0.678009726	TRUE
GB10071	1213	641	0.920183289	0.05566578	FALSE
GB10072	3	4	-0.415037499	-1.279555008	TRUE
GB10073	7010	4534	0.628630053	-0.235887455	FALSE
GB10074	656	368	0.833990049	-0.03052746	FALSE
GB10075	525	260	1.0138058	0.149288291	FALSE
GB10076	48	38	0.337034987	-0.527482521	TRUE
GB10077	179	77	1.217029237	0.352511728	FALSE
GB10078	219	129	0.763559804	-0.100957704	FALSE
GB10079	587	310	0.921092288	0.05657478	FALSE
GB10080	0	2	NA	NA	FALSE

GB10081	888	428	1.05294888	0.188431372	FALSE
GB10082	0	2	NA	NA	FALSE
GB10083	2056	1211	0.763641399	-0.100876109	FALSE
GB10084	50	26	0.943416472	0.078898963	FALSE
GB10085	11	0	NA	NA	FALSE
GB10086	1178	593	0.990235529	0.125718021	FALSE
GB10087	24	9	1.415037499	0.550519991	TRUE
GB10088	1521	704	1.111372819	0.246855311	FALSE
GB10089	2	5	-1.321928095	-2.186445603	TRUE
GB10090	539	280	0.944858446	0.080340937	FALSE
GB10091	25	13	0.943416472	0.078898963	FALSE
GB10092	634	344	0.882074275	0.017556767	FALSE
GB10093	2460	1295	0.925706218	0.061188709	FALSE
GB10094	29614	18613	0.669968766	-0.194548742	FALSE
GB10095	133	83	0.680243004	-0.184274504	FALSE
GB10096	784	359	1.12686981	0.262352302	FALSE
GB10097	1675	862	0.958401321	0.093883813	FALSE
GB10098	345	176	0.971020933	0.106503425	FALSE
GB10099	54	19	1.506959989	0.64244248	TRUE
GB10100	2553	1390	0.877108655	0.012591146	FALSE
GB10101	770	463	0.733846252	-0.130671256	FALSE
GB10102	4090	2509	0.704988372	-0.159529136	FALSE
GB10103	575	284	1.017671026	0.153153518	FALSE
GB10104	397	252	0.655715274	-0.208802235	FALSE
GB10105	444	283	0.649757624	-0.214759885	FALSE
GB10106	216	93	1.215728691	0.351211183	FALSE
GB10107	4726	3821	0.306669372	-0.557848137	TRUE
GB10108	61	12	2.345774837	1.481257329	TRUE
GB10109	1504	1178	0.352465028	-0.51205248	TRUE
GB10110	19	10	0.925999419	0.06148191	FALSE
GB10111	2126	1226	0.794182618	-0.07033489	FALSE
GB10112	486	288	0.754887502	-0.109630006	FALSE
GB10113	471	190	1.309727641	0.445210133	FALSE
GB10114	3587	1440	1.316708934	0.452191425	FALSE
GB10115	83	44	0.915607813	0.051090304	FALSE
GB10116	411	272	0.595531742	-0.268985766	FALSE
GB10117	0	1	NA	NA	FALSE
GB10118	477	213	1.163135836	0.298618327	FALSE
GB10119	83	37	1.165586066	0.301068557	FALSE
GB10120	760	409	0.893898575	0.029381067	FALSE
GB10121	1408	878	0.681354489	-0.183163019	FALSE
GB10122	9	5	0.847996907	-0.016520602	FALSE
GB10123	3248	1956	0.731645262	-0.132872246	FALSE
GB10124	14244	6601	1.109597838	0.245080329	FALSE

GB10125	NA	NA	NA	NA	FALSE
GB10126	523	361	0.534812109	-0.329705399	FALSE
GB10127	1572	988	0.670018271	-0.194499238	FALSE
GB10128	7891	4163	0.922584581	0.058067073	FALSE
GB10130	1238	969	0.353442744	-0.511074765	TRUE
GB10131	117	71	0.7206176	-0.143899908	FALSE
GB10132	296	183	0.693753527	-0.170763981	FALSE
GB10133	24743	14857	0.735877603	-0.128639905	FALSE
GB10134	486	128	1.924812504	1.060294995	TRUE
GB10135	174	118	0.560300446	-0.304217062	FALSE
GB10136	247	86	1.522102477	0.657584969	TRUE
GB10137	124	89	0.478462879	-0.386054629	FALSE
GB10138	880	592	0.571906348	-0.29261116	FALSE
GB10139	20262	9535	1.087471742	0.222954233	FALSE
GB10140	2007	1249	0.68426714	-0.180250369	FALSE
GB10141	850	497	0.774216989	-0.090300519	FALSE
GB10142	163	145	0.168819064	-0.695698444	TRUE
GB10143	2	6	-1.584962501	-2.449480009	TRUE
GB10144	1950	1272	0.616375453	-0.248142055	FALSE
GB10145	NA	NA	NA	NA	FALSE
GB10146	312	123	1.342887714	0.478370205	FALSE
GB10147	537	389	0.465151933	-0.399365575	FALSE
GB10148	0	2	NA	NA	FALSE
GB10149	276	172	0.682259702	-0.182257806	FALSE
GB10150	130	71	0.872620694	0.008103185	FALSE
GB10151	14	0	NA	NA	FALSE
GB10152	627	273	1.199564492	0.335046984	FALSE
GB10153	154	90	0.774933444	-0.089584064	FALSE
GB10154	318	197	0.690831136	-0.173686372	FALSE
GB10155	4663	3174	0.554956302	-0.309561207	FALSE
GB10156	630	424	0.571287564	-0.293229945	FALSE
GB10157	264	128	1.044394119	0.179876611	FALSE
GB10158	2924	1752	0.738940536	-0.125576972	FALSE
GB10159	1	0	NA	NA	FALSE
GB10160	185	151	0.292976721	-0.571540787	TRUE
GB10161	46	13	1.823122238	0.95860473	TRUE
GB10162	762	431	0.822103128	-0.04241438	FALSE
GB10163	59	46	0.359081093	-0.505436415	TRUE
GB10164	993	574	0.790742981	-0.073774527	FALSE
GB10165	266	132	1.010888316	0.146370808	FALSE
GB10166	43	22	0.966833136	0.102315628	FALSE
GB10167	10	4	1.321928095	0.457410587	FALSE
GB10168	2110	1020	1.048673847	0.184156338	FALSE
GB10169	175	139	0.332270039	-0.532247469	TRUE

GB10170	1008	630	0.678071905	-0.186445603	FALSE
GB10171	9	6	0.584962501	-0.279555008	FALSE
GB10172	930	567	0.713881981	-0.150635527	FALSE
GB10173	698	386	0.854626189	-0.009891319	FALSE
GB10174	685	453	0.596592938	-0.267924571	FALSE
GB10175	NA	NA	NA	NA	FALSE
GB10176	4480	3515	0.349974043	-0.514543465	TRUE
GB10177	464	311	0.577210225	-0.287307283	FALSE
GB10178	502	211	1.250444365	0.385926857	FALSE
GB10179	824	415	0.989533001	0.125015493	FALSE
GB10180	16	7	1.192645078	0.32812757	FALSE
GB10181	947	503	0.912806026	0.048288517	FALSE
GB10182	837	576	0.539158811	-0.325358697	FALSE
GB10183	6	6	0	-0.864517508	TRUE
GB10184	1942	1028	0.917702936	0.053185428	FALSE
GB10185	1699	978	0.796779482	-0.067738026	FALSE
GB10186	12	7	0.777607579	-0.08690993	FALSE
GB10187	72	31	1.215728691	0.351211183	FALSE
GB10188	164	73	1.167727446	0.303209937	FALSE
GB10189	2180	1091	0.998677033	0.134159525	FALSE
GB10190	188	93	1.015430041	0.150912532	FALSE
GB10191	1025	532	0.946125759	0.081608251	FALSE
GB10192	551	283	0.961250266	0.096732757	FALSE
GB10193	304	208	0.547487795	-0.317029713	FALSE
GB10194	419	245	0.774168495	-0.090349014	FALSE
GB10195	1232	619	0.992990941	0.128473433	FALSE
GB10196	914	556	0.717109282	-0.147408226	FALSE
GB10197	603	306	0.978626349	0.114108841	FALSE
GB10198	882	402	1.133583154	0.269065646	FALSE
GB10199	23	3	2.938599455	2.074081947	TRUE
GB10200	51	15	1.765534746	0.901017238	TRUE
GB10201	509	220	1.210162133	0.345644624	FALSE
GB10202	230	145	0.665580961	-0.198936547	FALSE
GB10203	226	130	0.797811149	-0.066706359	FALSE
GB10204	225	142	0.664034072	-0.200483437	FALSE
GB10205	234	103	1.183864192	0.319346684	FALSE
GB10206	340	208	0.708951218	-0.15556629	FALSE
GB10207	860	503	0.77377826	-0.090739249	FALSE
GB10208	3547	1884	0.912800365	0.048282857	FALSE
GB10209	390	190	1.037474705	0.172957197	FALSE
GB10210	24	18	0.415037499	-0.449480009	FALSE
GB10211	3364	2191	0.618588221	-0.245929287	FALSE
GB10212	1019	496	1.038742026	0.174224517	FALSE
GB10213	3	2	0.584962501	-0.279555008	FALSE

GB10214	1326	670	0.984847775	0.120330266	FALSE
GB10215	189	123	0.619727919	-0.244789589	FALSE
GB10216	2863	1770	0.69377831	-0.170739198	FALSE
GB10217	2844	1390	1.032836582	0.168319074	FALSE
GB10218	258	114	1.178337241	0.313819733	FALSE
GB10219	1970	1561	0.335725092	-0.528792416	TRUE
GB10220	811	415	0.966590578	0.10207307	FALSE
GB10221	470	227	1.049968459	0.185450951	FALSE
GB10222	25	3	3.058893689	2.194376181	TRUE
GB10223	43	28	0.618909833	-0.245607676	FALSE
GB10224	378	138	1.453717967	0.589200459	TRUE
GB10225	5	3	0.736965594	-0.127551914	FALSE
GB10226	70	42	0.736965594	-0.127551914	FALSE
GB10227	1541	655	1.23430005	0.369782542	FALSE
GB10228	1973	915	1.108547308	0.244029799	FALSE
GB10229	680	347	0.970599084	0.106081575	FALSE
GB10230	6	3	1	0.135482492	FALSE
GB10231	1181	594	0.991474129	0.12695662	FALSE
GB10232	550	214	1.361820822	0.497303314	FALSE
GB10233	376	178	1.078855421	0.214337912	FALSE
GB10234	707	503	0.491151815	-0.373365693	FALSE
GB10235	839	402	1.061475309	0.196957801	FALSE
GB10236	734	407	0.850751269	-0.01376624	FALSE
GB10237	603	326	0.887286038	0.022768529	FALSE
GB10238	22870	17131	0.416846996	-0.447670512	FALSE
GB10239	288	227	0.343376514	-0.521140994	TRUE
GB10240	15	4	1.906890596	1.042373087	TRUE
GB10241	834	448	0.896548651	0.032031143	FALSE
GB10242	330	255	0.371968777	-0.492548731	FALSE
GB10243	NA	NA	NA	NA	FALSE
GB10244	602	279	1.109498365	0.244980857	FALSE
GB10245	223	117	0.93053518	0.066017672	FALSE
GB10246	NA	NA	NA	NA	FALSE
GB10247	23	11	1.064130337	0.199612829	FALSE
GB10248	NA	NA	NA	NA	FALSE
GB10249	43	39	0.140862536	-0.723654972	TRUE
GB10250	714	384	0.894817763	0.030300255	FALSE
GB10251	289	167	0.79122139	-0.073296118	FALSE
GB10252	1488	657	1.179409251	0.314891742	FALSE
GB10253	5816	4053	0.521037094	-0.343480414	FALSE
GB10254	777	542	0.519621747	-0.344895761	FALSE
GB10255	211	153	0.463711346	-0.400806162	FALSE
GB10256	NA	NA	NA	NA	FALSE
GB10257	2311	1198	0.947889352	0.083371843	FALSE

GB10258	3580	2250	0.670034586	-0.194482922	FALSE
GB10259	5700	3183	0.840574762	-0.023942746	FALSE
GB10260	305	193	0.660208395	-0.204309113	FALSE
GB10261	160	68	1.234465254	0.369947745	FALSE
GB10262	384	196	0.970252657	0.105735148	FALSE
GB10263	96	73	0.395137942	-0.469379566	FALSE
GB10264	467	284	0.71753162	-0.146985888	FALSE
GB10265	746	487	0.615253858	-0.24926365	FALSE
GB10266	159	87	0.869939459	0.005421951	FALSE
GB10267	1	1	0	-0.864517508	TRUE
GB10268	504	238	1.08246216	0.217944652	FALSE
GB10269	NA	NA	NA	NA	FALSE
GB10270	269	156	0.786060144	-0.078457365	FALSE
GB10271	446	237	0.912156651	0.047639143	FALSE
GB10272	2543	1472	0.788753791	-0.075763718	FALSE
GB10273	470	270	0.79970135	-0.064816159	FALSE
GB10274	899	541	0.732692522	-0.131824987	FALSE
GB10275	98431	50527	0.962058228	0.09754072	FALSE
GB10276	1718	965	0.832129189	-0.032388319	FALSE
GB10277	714	413	0.789782293	-0.074735216	FALSE
GB10278	197	143	0.462180483	-0.402337026	FALSE
GB10279	764	433	0.819205613	-0.045311895	FALSE
GB10280	1049	612	0.77741112	-0.087106388	FALSE
GB10281	1558	873	0.835641674	-0.028875834	FALSE
GB10282	1671	1059	0.658009144	-0.206508364	FALSE
GB10283	2671	1714	0.640012867	-0.224504642	FALSE
GB10284	3019	2100	0.523681429	-0.340836079	FALSE
GB10285	NA	NA	NA	NA	FALSE
GB10286	85	75	0.180572246	-0.683945263	TRUE
GB10287	24	10	1.263034406	0.398516898	FALSE
GB10288	1799	1032	0.801752216	-0.062765292	FALSE
GB10289	16	7	1.192645078	0.32812757	FALSE
GB10290	1383	659	1.069450786	0.204933278	FALSE
GB10291	4	5	-0.321928095	-1.186445603	TRUE
GB10292	940	485	0.954676009	0.090158501	FALSE
GB10293	599	358	0.742596416	-0.121921093	FALSE
GB10294	15534	8902	0.803227965	-0.061289543	FALSE
GB10295	408	222	0.878009476	0.013491967	FALSE
GB10296	1249	655	0.931206665	0.066689157	FALSE
GB10297	1600	1064	0.588573754	-0.275943754	FALSE
GB10298	1	1	0	-0.864517508	TRUE
GB10299	35	7	2.321928095	1.457410587	TRUE
GB10300	1179	592	0.993894637	0.129377129	FALSE
GB10301	5	4	0.321928095	-0.542589413	TRUE

GB10302	59	42	0.490325627	-0.374191882	FALSE
GB10303	31	23	0.430634354	-0.433883154	FALSE
GB10304	447	299	0.580129347	-0.284388161	FALSE
GB10305	254	243	0.063872183	-0.800645325	TRUE
GB10306	288	154	0.903138461	0.038620952	FALSE
GB10307	212	123	0.785405949	-0.079111559	FALSE
GB10308	8	3	1.415037499	0.550519991	TRUE
GB10309	5505	2591	1.087233548	0.22271604	FALSE
GB10310	1534	692	1.14845454	0.283937032	FALSE
GB10311	1837	895	1.037392039	0.172874531	FALSE
GB10312	6081	3113	0.966003013	0.101485504	FALSE
GB10313	1331	626	1.088276009	0.223758501	FALSE
GB10314	146	102	0.517399217	-0.347118291	FALSE
GB10315	1348	772	0.804147744	-0.060369764	FALSE
GB10316	2678	1378	0.958580073	0.094062564	FALSE
GB10317	180	90	1	0.135482492	FALSE
GB10318	605	292	1.050966773	0.186449265	FALSE
GB10319	40	10	2	1.135482492	TRUE
GB10320	1358	984	0.464753259	-0.399764249	FALSE
GB10321	1113	618	0.848774849	-0.015742659	FALSE
GB10322	184	117	0.653197236	-0.211320272	FALSE
GB10323	537	366	0.55307844	-0.311439069	FALSE
GB10324	763	482	0.662649911	-0.201867598	FALSE
GB10325	3883	1402	1.469685358	0.60516785	TRUE
GB10326	61	33	0.886343218	0.02182571	FALSE
GB10327	430	263	0.70927386	-0.155243648	FALSE
GB10328	1	0	NA	NA	FALSE
GB10329	417	267	0.643207642	-0.221309867	FALSE
GB10330	NA	NA	NA	NA	FALSE
GB10331	489	302	0.695285916	-0.169231593	FALSE
GB10332	1790	832	1.105304154	0.240786646	FALSE
GB10333	12	7	0.777607579	-0.08690993	FALSE
GB10334	80	41	0.96437609	0.099858582	FALSE
GB10335	NA	NA	NA	NA	FALSE
GB10336	838	630	0.411598415	-0.452919093	FALSE
GB10337	6133	4208	0.543458246	-0.321059262	FALSE
GB10338	1364	735	0.892027489	0.027509981	FALSE
GB10339	10188	4031	1.337661176	0.473143668	FALSE
GB10340	3265	2343	0.478726038	-0.385791471	FALSE
GB10341	388	167	1.21620855	0.351691041	FALSE
GB10342	1186	603	0.975874103	0.111356594	FALSE
GB10343	1040	700	0.571156701	-0.293360807	FALSE
GB10344	1009	649	0.636635791	-0.227881717	FALSE
GB10345	7	8	-0.192645078	-1.057162586	TRUE

GB10346	2421	1401	0.789146124	-0.075371385	FALSE
GB10347	86	22	1.966833136	1.102315628	TRUE
GB10348	216	66	1.710493383	0.845975874	TRUE
GB10349	591	286	1.047142983	0.182625475	FALSE
GB10350	1218	663	0.877433358	0.012915849	FALSE
GB10351	2634	1802	0.547656334	-0.316861174	FALSE
GB10352	1236	754	0.713042315	-0.151475194	FALSE
GB10353	18482	14185	0.381754734	-0.482762775	FALSE
GB10354	10	3	1.736965594	0.872448086	TRUE
GB10355	2	0	NA	NA	FALSE
GB10356	35	17	1.041820176	0.177302667	FALSE
GB10357	640	361	0.826073068	-0.03844444	FALSE
GB10358	2256	1203	0.907130425	0.042612917	FALSE
GB10359	2378	1328	0.840493568	-0.02402394	FALSE
GB10360	878	624	0.492674911	-0.371842598	FALSE
GB10361	799	590	0.437480549	-0.42703696	FALSE
GB10362	14	11	0.347923303	-0.516594205	TRUE
GB10363	17	11	0.628031223	-0.236486286	FALSE
GB10364	169	102	0.728454094	-0.136063414	FALSE
GB10365	165	96	0.781359714	-0.083157795	FALSE
GB10366	751	276	1.444144641	0.579627132	TRUE
GB10367	26981	20232	0.415304878	-0.44921263	FALSE
GB10368	0	1	NA	NA	FALSE
GB10369	339	252	0.42786154	-0.436655969	FALSE
GB10370	254	130	0.966316874	0.101799365	FALSE
GB10371	3	0	NA	NA	FALSE
GB10372	720	453	0.668485856	-0.196031652	FALSE
GB10373	8	2	2	1.135482492	TRUE
GB10374	856	440	0.960107273	0.095589765	FALSE
GB10375	217	72	1.591626231	0.727108723	TRUE
GB10376	1494	761	0.973211789	0.108694281	FALSE
GB10377	1853	1043	0.829123724	-0.035393785	FALSE
GB10378	751	378	0.990426673	0.125909165	FALSE
GB10379	1180	613	0.944827881	0.080310372	FALSE
GB10380	518	293	0.822051433	-0.042466075	FALSE
GB10381	2900	1576	0.879785365	0.015267857	FALSE
GB10382	2867	1700	0.754007158	-0.11051035	FALSE
GB10383	0	2	NA	NA	FALSE
GB10384	NA	NA	NA	NA	FALSE
GB10385	23	7	1.716207034	0.851689526	TRUE
GB10386	1443	661	1.126349123	0.261831615	FALSE
GB10387	5222	1634	1.676194474	0.811676966	TRUE
GB10388	1264	571	1.146433813	0.281916304	FALSE
GB10389	10	6	0.736965594	-0.127551914	FALSE

GB10390	4423	2605	0.76374187	-0.100775639	FALSE
GB10391	1219	633	0.945420721	0.080903213	FALSE
GB10392	437	215	1.02329662	0.158779112	FALSE
GB10393	183	94	0.961110987	0.096593478	FALSE
GB10394	599	331	0.855724786	-0.008792722	FALSE
GB10395	353	155	1.187399968	0.32288246	FALSE
GB10396	1009	518	0.961902171	0.097384663	FALSE
GB10397	3206	1672	0.939199578	0.07468207	FALSE
GB10398	72	42	0.777607579	-0.08690993	FALSE
GB10399	607	324	0.905702703	0.041185195	FALSE
GB10400	593	440	0.430528581	-0.433988927	FALSE
GB10401	273	187	0.545862681	-0.318654827	FALSE
GB10402	339	221	0.617238904	-0.247278605	FALSE
GB10403	395	286	0.465837506	-0.398680002	FALSE
GB10404	NA	NA	NA	NA	FALSE
GB10405	119	84	0.502500341	-0.362017168	FALSE
GB10406	2466	1491	0.725892542	-0.138624966	FALSE
GB10407	471	293	0.684826395	-0.179691113	FALSE
GB10408	4	1	2	1.135482492	TRUE
GB10409	463	247	0.906501152	0.041983643	FALSE
GB10410	0	1	NA	NA	FALSE
GB10411	3020	2022	0.578765552	-0.285751956	FALSE
GB10412	1	0	NA	NA	FALSE
GB10413	25337	17579	0.52739271	-0.337124798	FALSE
GB10414	918	359	1.35451031	0.489992801	FALSE
GB10415	NA	NA	NA	NA	FALSE
GB10416	2	0	NA	NA	FALSE
GB10417	NA	NA	NA	NA	FALSE
GB10418	39	14	1.478047297	0.613529788	TRUE
GB10419	4	3	0.415037499	-0.449480009	FALSE
GB10420	3367	2385	0.497474455	-0.367043053	FALSE
GB10421	2262	724	1.643537327	0.779019819	TRUE
GB10422	445	333	0.418283159	-0.44623435	FALSE
GB10423	461	217	1.087071708	0.2225542	FALSE
GB10424	10	3	1.736965594	0.872448086	TRUE
GB10425	23	21	0.131244533	-0.733272975	TRUE
GB10426	420	214	0.972778531	0.108261023	FALSE
GB10427	878	423	1.053563276	0.189045768	FALSE
GB10428	57109	21858	1.385556722	0.521039213	TRUE
GB10429	37	42	-0.182864057	-1.047381565	TRUE
GB10430	136	68	1	0.135482492	FALSE
GB10431	134	69	0.957564734	0.093047225	FALSE
GB10432	3574	1761	1.021144725	0.156627217	FALSE
GB10433	2204	1180	0.901337364	0.036819856	FALSE

GB10434	646	366	0.819690516	-0.044826992	FALSE
GB10435	544	278	0.968521769	0.10400426	FALSE
GB10436	3633	2117	0.779140096	-0.085377412	FALSE
GB10437	192	104	0.884522783	0.020005274	FALSE
GB10438	1429	880	0.699430488	-0.165087021	FALSE
GB10439	6	5	0.263034406	-0.601483102	TRUE
GB10440	81	65	0.31748219	-0.547035318	TRUE
GB10441	37526	18234	1.041259437	0.176741929	FALSE
GB10442	628	320	0.972692654	0.108175146	FALSE
GB10443	NA	NA	NA	NA	FALSE
GB10444	1577	1133	0.477034799	-0.387482709	FALSE
GB10445	1042	483	1.109260183	0.244742675	FALSE
GB10446	527	269	0.970196789	0.105679281	FALSE
GB10447	1	4	-2	-2.864517508	TRUE
GB10448	0	1	NA	NA	FALSE
GB10449	505	263	0.941220588	0.07670308	FALSE
GB10450	59	22	1.423211431	0.558693922	TRUE
GB10451	84	64	0.392317423	-0.472200086	FALSE
GB10452	441	271	0.702485804	-0.162031704	FALSE
GB10453	8243	3439	1.261180379	0.396662871	FALSE
GB10454	21	9	1.222392421	0.357874913	FALSE
GB10455	161	96	0.745954377	-0.118563131	FALSE
GB10456	1648	830	0.989533001	0.125015493	FALSE
GB10457	1582	713	1.149775618	0.28525811	FALSE
GB10458	401	237	0.758715178	-0.105802331	FALSE
GB10459	748	318	1.234011505	0.369493996	FALSE
GB10460	1012	694	0.544201722	-0.320315786	FALSE
GB10461	1494	1024	0.544964433	-0.319553076	FALSE
GB10462	729	436	0.74159068	-0.122926829	FALSE
GB10463	217	83	1.386511801	0.521994293	TRUE
GB10464	251	164	0.613991549	-0.250525959	FALSE
GB10465	173	86	1.008363473	0.143845965	FALSE
GB10466	1128	645	0.806396002	-0.058121506	FALSE
GB10467	7551	4280	0.819056921	-0.045460588	FALSE
GB10468	1	1	0	-0.864517508	TRUE
GB10469	1200	559	1.102114218	0.237596709	FALSE
GB10470	497	276	0.848577585	-0.015939924	FALSE
GB10471	635	383	0.7294122	-0.135105309	FALSE
GB10472	353	195	0.85619406	-0.008323449	FALSE
GB10473	173	94	0.880039376	0.015521868	FALSE
GB10474	2105	1381	0.608106914	-0.256410595	FALSE
GB10475	177	106	0.739685096	-0.124832413	FALSE
GB10476	583	251	1.215808519	0.351291011	FALSE
GB10477	6790	4748	0.516091639	-0.348425869	FALSE

GB10478	19	7	1.440572591	0.576055083	TRUE
GB10479	NA	NA	NA	NA	FALSE
GB10480	4	0	NA	NA	FALSE
GB10481	611	253	1.272034995	0.407517487	FALSE
GB10482	523	196	1.415957292	0.551439784	TRUE
GB10483	1050	305	1.78350818	0.918990672	TRUE
GB10484	4	1	2	1.135482492	TRUE
GB10485	55	34	0.693896872	-0.170620636	FALSE
GB10486	1	0	NA	NA	FALSE
GB10487	12516	5093	1.297185941	0.432668433	FALSE
GB10488	674	361	0.900749754	0.036232246	FALSE
GB10489	240	106	1.178970141	0.314452633	FALSE
GB10490	617	389	0.665500334	-0.199017174	FALSE
GB10491	2816	1660	0.762464092	-0.102053416	FALSE
GB10492	472	265	0.8327945	-0.031723008	FALSE
GB10493	1223	792	0.626852068	-0.23766544	FALSE
GB10494	94	47	1	0.135482492	FALSE
GB10495	NA	NA	NA	NA	FALSE
GB10496	NA	NA	NA	NA	FALSE
GB10497	310	119	1.381306642	0.516789134	TRUE
GB10498	93780	55240	0.763566959	-0.100950549	FALSE
GB10499	707	336	1.073248982	0.208731474	FALSE
GB10500	877	494	0.828065801	-0.036451707	FALSE
GB10501	443	237	0.90241964	0.037902131	FALSE
GB10502	33	15	1.137503524	0.272986015	FALSE
GB10503	3251	1746	0.896829997	0.032312489	FALSE
GB10504	5244	2754	0.929139126	0.064621618	FALSE
GB10505	NA	NA	NA	NA	FALSE
GB10506	2052	912	1.169925001	0.305407493	FALSE
GB10507	157	141	0.155069396	-0.709448112	TRUE
GB10508	24	12	1	0.135482492	FALSE
GB10509	1089	574	0.923881312	0.059363804	FALSE
GB10510	5074	2083	1.28446068	0.419943172	FALSE
GB10511	3144	1711	0.877761458	0.013243949	FALSE
GB10512	1257	732	0.780069096	-0.084448412	FALSE
GB10513	138	96	0.523561956	-0.340955552	FALSE
GB10514	86674	44318	0.96770651	0.103189002	FALSE
GB10515	575	201	1.516366455	0.651848946	TRUE
GB10516	3006	1806	0.735047116	-0.129470392	FALSE
GB10517	14273	6894	1.049875399	0.18535789	FALSE
GB10518	580	281	1.04548277	0.180965261	FALSE
GB10519	1624	704	1.205904299	0.34138679	FALSE
GB10520	2925	1965	0.573907312	-0.290610196	FALSE
GB10521	508	381	0.415037499	-0.449480009	FALSE

GB10522	958	454	1.077333358	0.21281585	FALSE
GB10523	26	7	1.893084796	1.028567288	TRUE
GB10524	8145	5320	0.614488453	-0.250029055	FALSE
GB10525	18	10	0.847996907	-0.016520602	FALSE
GB10526	3	9	-1.584962501	-2.449480009	TRUE
GB10527	NA	NA	NA	NA	FALSE
GB10528	NA	NA	NA	NA	FALSE
GB10529	380	113	1.749676646	0.885159138	TRUE
GB10530	1125	691	0.703167386	-0.161350123	FALSE
GB10531	1103	681	0.695706088	-0.168811421	FALSE
GB10532	5974	3319	0.847948608	-0.0165689	FALSE
GB10533	783	356	1.137135066	0.272617558	FALSE
GB10534	7818	4224	0.88818975	0.023672242	FALSE
GB10535	1047	661	0.663539265	-0.200978243	FALSE
GB10536	8	12	-0.584962501	-1.449480009	TRUE
GB10537	298	175	0.767957409	-0.0965601	FALSE
GB10538	1155	781	0.564498398	-0.30001911	FALSE
GB10539	380	228	0.736965594	-0.127551914	FALSE
GB10540	130	79	0.718587065	-0.145930443	FALSE
GB10541	566	386	0.552201206	-0.312316303	FALSE
GB10542	146	72	1.019899557	0.155382049	FALSE
GB10543	127	70	0.85940167	-0.005115839	FALSE
GB10544	146	98	0.575114715	-0.289402794	FALSE
GB10545	9993	5257	0.92667812	0.062160611	FALSE
GB10546	47	36	0.38466385	-0.479853658	FALSE
GB10547	177	65	1.445237737	0.580720229	TRUE
GB10548	85	46	0.88582898	0.021311472	FALSE
GB10549	392	148	1.405256478	0.54073897	TRUE
GB10550	312	202	0.627190736	-0.237326772	FALSE
GB10551	61	34	0.843274496	-0.021243012	FALSE
GB10552	60	49	0.292180751	-0.572336757	TRUE
GB10553	101	72	0.488286481	-0.376231027	FALSE
GB10554	70	54	0.374395515	-0.490121994	FALSE
GB10555	753	546	0.463748914	-0.400768595	FALSE
GB10556	6	2	1.584962501	0.720444992	TRUE
GB10557	670	477	0.490171829	-0.374345679	FALSE
GB10558	227	107	1.085081501	0.220563993	FALSE
GB10559	91	48	0.922832139	0.058314631	FALSE
GB10560	1604	558	1.523337115	0.658819606	TRUE
GB10561	5619	3756	0.581116337	-0.283401171	FALSE
GB10562	644	424	0.602996424	-0.261521085	FALSE
GB10563	20	2	3.321928095	2.457410587	TRUE
GB10564	1650	952	0.793432546	-0.071084963	FALSE
GB10565	3150	1498	1.072314205	0.207796696	FALSE

GB10566	0	3	NA	NA	FALSE
GB10567	NA	NA	NA	NA	FALSE
GB10568	2672	1556	0.780077947	-0.084439561	FALSE
GB10569	32	14	1.192645078	0.32812757	FALSE
GB10570	629	402	0.645864516	-0.218652993	FALSE
GB10571	1	0	NA	NA	FALSE
GB10572	1471	788	0.900529712	0.036012203	FALSE
GB10573	1	0	NA	NA	FALSE
GB10574	1207	437	1.465720491	0.601202983	TRUE
GB10575	946	576	0.715771372	-0.148746136	FALSE
GB10576	9863	4215	1.226493903	0.361976394	FALSE
GB10577	1192	677	0.816156497	-0.048361011	FALSE
GB10578	189	138	0.453717967	-0.410799541	FALSE
GB10579	2222	1165	0.931528862	0.067011354	FALSE
GB10580	980	498	0.976636007	0.112118499	FALSE
GB10581	1545	869	0.830178756	-0.034338752	FALSE
GB10582	179	95	0.913960169	0.049442661	FALSE
GB10583	187	54	1.792006958	0.927489449	TRUE
GB10584	6946	2349	1.564135697	0.699618189	TRUE
GB10585	74	54	0.454565863	-0.409951645	FALSE
GB10586	258	145	0.831318165	-0.033199343	FALSE
GB10587	9493	5811	0.708077628	-0.15643988	FALSE
GB10588	4	0	NA	NA	FALSE
GB10589	45	13	1.791413378	0.92689587	TRUE
GB10590	2287	1508	0.600819937	-0.263697571	FALSE
GB10591	563	361	0.641136085	-0.223381423	FALSE
GB10592	84	32	1.392317423	0.527799914	TRUE
GB10593	64	27	1.245112498	0.38059499	FALSE
GB10594	1669	1243	0.425157658	-0.43935985	FALSE
GB10595	124	53	1.226275856	0.361758347	FALSE
GB10596	397	265	0.583146648	-0.281370861	FALSE
GB10597	156	88	0.8259706	-0.038546908	FALSE
GB10598	2786	1814	0.619020802	-0.245496706	FALSE
GB10599	247	172	0.522102477	-0.342415031	FALSE
GB10600	NA	NA	NA	NA	FALSE
GB10601	33	23	0.520832163	-0.343685345	FALSE
GB10602	53	19	1.479992941	0.615475433	TRUE
GB10603	1066	486	1.133179219	0.268661711	FALSE
GB10604	410	244	0.748742762	-0.115774746	FALSE
GB10605	1741	910	0.935977753	0.071460244	FALSE
GB10606	48	24	1	0.135482492	FALSE
GB10607	1850	1074	0.784531277	-0.079986231	FALSE
GB10608	1714	938	0.869707282	0.005189773	FALSE
GB10609	1024	546	0.907242859	0.042725351	FALSE

GB10610	122	60	1.023846742	0.159329234	FALSE
GB10611	229	139	0.720262715	-0.144254793	FALSE
GB10612	84	38	1.144389909	0.279872401	FALSE
GB10613	9	6	0.584962501	-0.279555008	FALSE
GB10614	4671	3437	0.442581593	-0.421935915	FALSE
GB10615	115	85	0.436099115	-0.428418394	FALSE
GB10616	NA	NA	NA	NA	FALSE
GB10617	54	18	1.584962501	0.720444992	TRUE
GB10618	82	59	0.474908955	-0.389608553	FALSE
GB10619	NA	NA	NA	NA	FALSE
GB10620	6	0	NA	NA	FALSE
GB10621	2266	1366	0.730190378	-0.134327131	FALSE
GB10622	0	1	NA	NA	FALSE
GB10623	34	25	0.443606651	-0.420910857	FALSE
GB10624	0	1	NA	NA	FALSE
GB10625	1257	694	0.856977082	-0.007540426	FALSE
GB10626	833	615	0.437730085	-0.426787423	FALSE
GB10627	1	0	NA	NA	FALSE
GB10628	215	66	1.70379873	0.839281222	TRUE
GB10629	857	494	0.794784163	-0.069733346	FALSE
GB10630	13	10	0.378511623	-0.486005885	FALSE
GB10631	655	345	0.924898545	0.060381036	FALSE
GB10632	417	276	0.595379117	-0.269138392	FALSE
GB10633	1569	910	0.785906902	-0.078610606	FALSE
GB10634	523	309	0.759204108	-0.1053134	FALSE
GB10635	15	13	0.206450877	-0.658066631	TRUE
GB10636	652	440	0.567368441	-0.297149068	FALSE
GB10637	1450	912	0.668947171	-0.195570338	FALSE
GB10638	NA	NA	NA	NA	FALSE
GB10639	NA	NA	NA	NA	FALSE
GB10640	342	172	0.99158776	0.127070252	FALSE
GB10641	570	308	0.888031568	0.02351406	FALSE
GB10642	33	15	1.137503524	0.272986015	FALSE
GB10643	638	364	0.809617974	-0.054899535	FALSE
GB10644	38	16	1.247927513	0.383410005	FALSE
GB10645	67	37	0.856635825	-0.007881683	FALSE
GB10646	254	133	0.933402251	0.068884743	FALSE
GB10647	127	77	0.721898146	-0.142619362	FALSE
GB10648	1870	1205	0.634005124	-0.230512385	FALSE
GB10649	150	115	0.38332864	-0.481188869	FALSE
GB10650	130	73	0.832543254	-0.031974254	FALSE
GB10651	637	401	0.667691136	-0.196826373	FALSE
GB10652	819	571	0.520372706	-0.344144802	FALSE
GB10653	17	11	0.628031223	-0.236486286	FALSE

GB10654	3080	1375	1.163498732	0.298981224	FALSE
GB10655	717	417	0.781925735	-0.082591773	FALSE
GB10656	110	60	0.874469118	0.00995161	FALSE
GB10657	6745	3741	0.850394477	-0.014123031	FALSE
GB10658	1490	902	0.724112992	-0.140404516	FALSE
GB10659	465	183	1.345387068	0.480869559	FALSE
GB10660	2278	1208	0.915147292	0.050629784	FALSE
GB10661	272	135	1.010647244	0.146129736	FALSE
GB10662	130	89	0.546634382	-0.317883126	FALSE
GB10663	NA	NA	NA	NA	FALSE
GB10664	2955	1631	0.857401348	-0.00711616	FALSE
GB10665	266	131	1.021859434	0.157341926	FALSE
GB10666	38	32	0.247927513	-0.616589995	TRUE
GB10667	1421	752	0.918101988	0.053584479	FALSE
GB10668	NA	NA	NA	NA	FALSE
GB10669	22071	13872	0.669976194	-0.194541315	FALSE
GB10670	725	404	0.843625702	-0.020891806	FALSE
GB10671	9	7	0.362570079	-0.501947429	TRUE
GB10672	195	83	1.232290882	0.367773374	FALSE
GB10673	9268	4975	0.897561519	0.03304401	FALSE
GB10674	NA	NA	NA	NA	FALSE
GB10675	6018	3743	0.685089058	-0.17942845	FALSE
GB10676	413	254	0.701313285	-0.163204224	FALSE
GB10677	544	268	1.021373651	0.156856142	FALSE
GB10678	6	4	0.584962501	-0.279555008	FALSE
GB10679	96	38	1.337034987	0.472517479	FALSE
GB10680	171	78	1.132450296	0.267932788	FALSE
GB10681	63	71	-0.172467196	-1.036984704	TRUE
GB10682	NA	NA	NA	NA	FALSE
GB10683	119	50	1.250961574	0.386444065	FALSE
GB10684	201	91	1.143257051	0.278739543	FALSE
GB10685	0	3	NA	NA	FALSE
GB10686	499	258	0.95166875	0.087151242	FALSE
GB10687	914	534	0.775354423	-0.089163085	FALSE
GB10688	15	3	2.321928095	1.457410587	TRUE
GB10689	1	1	0	-0.864517508	TRUE
GB10690	1074	660	0.702456064	-0.162061445	FALSE
GB10691	718	364	0.980045394	0.115527885	FALSE
GB10692	610	384	0.667702932	-0.196814577	FALSE
GB10693	145	80	0.857980995	-0.006536513	FALSE
GB10694	481	275	0.806605275	-0.057912233	FALSE
GB10695	14658	11217	0.386001392	-0.478516116	FALSE
GB10696	204	115	0.826935291	-0.037582217	FALSE
GB10697	NA	NA	NA	NA	FALSE

GB10698	890	534	0.736965594	-0.127551914	FALSE
GB10699	7537	4575	0.720218649	-0.144298859	FALSE
GB10700	613	284	1.109996144	0.245478636	FALSE
GB10701	9	7	0.362570079	-0.501947429	TRUE
GB10702	1686	865	0.962832498	0.09831499	FALSE
GB10703	0	1	NA	NA	FALSE
GB10704	3398	2094	0.69842441	-0.166093098	FALSE
GB10705	144	58	1.311944006	0.447426498	FALSE
GB10706	1331	652	1.029566702	0.165049193	FALSE
GB10707	1	0	NA	NA	FALSE
GB10708	3	12	-2	-2.864517508	TRUE
GB10709	662	344	0.944422652	0.079905144	FALSE
GB10710	379	147	1.366381693	0.501864185	TRUE
GB10711	4523	2362	0.937271031	0.072753523	FALSE
GB10712	1	0	NA	NA	FALSE
GB10713	420	253	0.731251943	-0.133265565	FALSE
GB10714	594	471	0.334735871	-0.529781637	TRUE
GB10715	1421	698	1.025607613	0.161090105	FALSE
GB10716	417	274	0.60587149	-0.258646018	FALSE
GB10717	26863	11406	1.235827493	0.371309985	FALSE
GB10718	NA	NA	NA	NA	FALSE
GB10719	201	139	0.532110618	-0.33240689	FALSE
GB10720	1728	896	0.94753258	0.083015072	FALSE
GB10721	662	453	0.547320167	-0.317197342	FALSE
GB10722	75	34	1.141355849	0.276838341	FALSE
GB10723	163	106	0.6208077	-0.243709809	FALSE
GB10724	8	2	2	1.135482492	TRUE
GB10725	291	133	1.129592907	0.265075399	FALSE
GB10726	381	241	0.660757851	-0.203759657	FALSE
GB10727	921	459	1.004707003	0.140189494	FALSE
GB10728	180	135	0.415037499	-0.449480009	FALSE
GB10729	620	391	0.665099608	-0.1994179	FALSE
GB10730	2581	1291	0.999441141	0.134923632	FALSE
GB10731	NA	NA	NA	NA	FALSE
GB10732	6500	5261	0.305102668	-0.55941484	TRUE
GB10733	361	189	0.933612603	0.069095094	FALSE
GB10734	179	83	1.108776346	0.244258838	FALSE
GB10735	8	1	3	2.135482492	TRUE
GB10736	511	167	1.613475188	0.74895768	TRUE
GB10737	81	30	1.432959407	0.568441899	TRUE
GB10738	842	499	0.754780418	-0.109737091	FALSE
GB10739	1159	675	0.779921159	-0.084596349	FALSE
GB10740	371	214	0.79380839	-0.070709118	FALSE
GB10741	80	47	0.767339243	-0.097178265	FALSE

GB10742	192	75	1.35614381	0.491626302	FALSE
GB10743	53	56	-0.079434467	-0.943951976	TRUE
GB10744	670	403	0.733381257	-0.131136252	FALSE
GB10745	716	452	0.663636815	-0.200880693	FALSE
GB10746	288	159	0.857042046	-0.007475462	FALSE
GB10747	219	145	0.59487797	-0.269639539	FALSE
GB10748	136	61	1.156725504	0.292207995	FALSE
GB10749	242	83	1.543823806	0.679306298	TRUE
GB10750	737	437	0.75403134	-0.110486169	FALSE
GB10751	3661	2128	0.782739623	-0.081777886	FALSE
GB10752	10	1	3.321928095	2.457410587	TRUE
GB10753	13	9	0.530514717	-0.334002792	FALSE
GB10754	622	300	1.05195208	0.187434571	FALSE
GB10755	705	378	0.899237023	0.034719515	FALSE
GB10756	14	8	0.807354922	-0.057162586	FALSE
GB10757	3674	2016	0.865855988	0.001338479	FALSE
GB10758	2275	1008	1.174370906	0.309853398	FALSE
GB10759	3118	1676	0.895598779	0.031081271	FALSE
GB10760	38	19	1	0.135482492	FALSE
GB10761	NA	NA	NA	NA	FALSE
GB10762	914	467	0.968771615	0.104254107	FALSE
GB10763	314	175	0.843409637	-0.021107871	FALSE
GB10764	700	140	2.321928095	1.457410587	TRUE
GB10765	NA	NA	NA	NA	FALSE
GB10766	513	267	0.942119084	0.077601576	FALSE
GB10767	164	132	0.313157885	-0.551359623	TRUE
GB10768	528	294	0.844721775	-0.019795734	FALSE
GB10769	2556	1649	0.632296438	-0.232221071	FALSE
GB10770	488	287	0.765830411	-0.098687097	FALSE
GB10771	38023	24322	0.644610498	-0.21990701	FALSE
GB10772	273	171	0.674904626	-0.189612882	FALSE
GB10773	152	97	0.648014671	-0.216502837	FALSE
GB10774	1708	905	0.916318278	0.051800769	FALSE
GB10775	629	358	0.81310043	-0.051417079	FALSE
GB10776	86	59	0.543621705	-0.320895803	FALSE
GB10777	1013	599	0.758006266	-0.106511242	FALSE
GB10778	215	139	0.629251777	-0.235265731	FALSE
GB10779	36	25	0.526068812	-0.338448697	FALSE
GB10780	222	114	0.961525852	0.097008344	FALSE
GB10781	210	98	1.099535674	0.235018165	FALSE
GB10782	877	468	0.906068313	0.041550805	FALSE
GB10783	193	99	0.963100417	0.098582909	FALSE
GB10784	19025	9074	1.068085889	0.203568381	FALSE
GB10785	539	308	0.807354922	-0.057162586	FALSE

GB10786	481	221	1.121990524	0.257473016	FALSE
GB10787	333	165	1.013056153	0.148538644	FALSE
GB10788	NA	NA	NA	NA	FALSE
GB10789	3979	2921	0.445943541	-0.418573967	FALSE
GB10790	615	401	0.616984174	-0.247533335	FALSE
GB10791	336	212	0.664396968	-0.20012054	FALSE
GB10792	995	576	0.788627714	-0.075889794	FALSE
GB10793	2366	1385	0.772564097	-0.091953411	FALSE
GB10794	2438	1300	0.907186503	0.042668994	FALSE
GB10795	3968	1799	1.141216839	0.276699331	FALSE
GB10796	2089	1539	0.440819261	-0.423698248	FALSE
GB10797	434	258	0.750323977	-0.114193531	FALSE
GB10798	1	1	0	-0.864517508	TRUE
GB10799	3538	1819	0.959788505	0.095270997	FALSE
GB10800	6567	4523	0.537954459	-0.326563049	FALSE
GB10801	2	1	1	0.135482492	FALSE
GB10802	NA	NA	NA	NA	FALSE
GB10803	1213	710	0.772688621	-0.091828888	FALSE
GB10804	489	335	0.54567337	-0.318844139	FALSE
GB10805	461	236	0.965979891	0.101462383	FALSE
GB10806	1396	650	1.102787318	0.23826981	FALSE
GB10807	1216	686	0.825862747	-0.038654761	FALSE
GB10808	552	344	0.682259702	-0.182257806	FALSE
GB10809	664	391	0.764014634	-0.100502874	FALSE
GB10810	2138	953	1.165713734	0.301196225	FALSE
GB10811	150	85	0.819427754	-0.045089754	FALSE
GB10812	1953	1152	0.761551232	-0.102966276	FALSE
GB10813	540	295	0.872244453	0.007726944	FALSE
GB10814	1	0	NA	NA	FALSE
GB10815	3620	1676	1.110967548	0.24645004	FALSE
GB10816	4	0	NA	NA	FALSE
GB10817	605	350	0.78958022	-0.074937288	FALSE
GB10818	884	476	0.893084796	0.028567288	FALSE
GB10819	1071	627	0.772421132	-0.092096376	FALSE
GB10820	5	4	0.321928095	-0.542589413	TRUE
GB10821	133	28	2.247927513	1.383410005	TRUE
GB10822	NA	NA	NA	NA	FALSE
GB10823	232	144	0.688055994	-0.176461515	FALSE
GB10824	20	19	0.074000581	-0.790516927	TRUE
GB10825	4	1	2	1.135482492	TRUE
GB10826	1172	552	1.086232398	0.221714889	FALSE
GB10827	2386	1307	0.868334902	0.003817394	FALSE
GB10828	1062	671	0.662399095	-0.202118414	FALSE
GB10829	215	151	0.50978811	-0.354729398	FALSE

GB10830	1966	1193	0.720669279	-0.14384823	FALSE
GB10831	7	5	0.485426827	-0.379090681	FALSE
GB10832	871	488	0.835791571	-0.028725937	FALSE
GB10833	1996	1048	0.929473004	0.064955495	FALSE
GB10834	1291	757	0.770123795	-0.094393713	FALSE
GB10835	633	380	0.736206081	-0.128311427	FALSE
GB10836	4125	2443	0.755740256	-0.108777253	FALSE
GB10837	NA	NA	NA	NA	FALSE
GB10838	372	230	0.69366876	-0.170848748	FALSE
GB10839	482	277	0.79914717	-0.065370338	FALSE
GB10840	0	2	NA	NA	FALSE
GB10841	77	20	1.944858446	1.080340937	TRUE
GB10842	1	2	-1	-1.864517508	TRUE
GB10843	580	300	0.9510904	0.086572891	FALSE
GB10844	22	24	-0.125530882	-0.99004839	TRUE
GB10845	1329	769	0.789285601	-0.075231907	FALSE
GB10846	990	409	1.275327682	0.410810174	FALSE
GB10847	415	217	0.935416294	0.070898785	FALSE
GB10848	266	155	0.77915803	-0.085359478	FALSE
GB10849	70	31	1.175086707	0.310569198	FALSE
GB10850	1888	1091	0.791207663	-0.073309845	FALSE
GB10851	1966	2279	-0.213137603	-1.077655111	TRUE
GB10852	636	425	0.581563924	-0.282953584	FALSE
GB10853	1027	551	0.898311958	0.033794449	FALSE
GB10854	3000	1536	0.965784285	0.101266776	FALSE
GB10855	2899	1937	0.581731379	-0.282786129	FALSE
GB10856	518	252	1.039528364	0.175010856	FALSE
GB10857	640	404	0.663716612	-0.200800896	FALSE
GB10858	23	9	1.353636955	0.489119446	FALSE
GB10859	1806	1082	0.739097394	-0.125420115	FALSE
GB10860	280	175	0.678071905	-0.186445603	FALSE
GB10861	45255	30550	0.566904817	-0.297612691	FALSE
GB10862	247	78	1.662965013	0.798447504	TRUE
GB10863	762	469	0.700203075	-0.164314433	FALSE
GB10864	643	314	1.034054178	0.16953667	FALSE
GB10865	113	59	0.937535913	0.073018405	FALSE
GB10866	3	0	NA	NA	FALSE
GB10867	112	33	1.762960803	0.898443294	TRUE
GB10868	0	1	NA	NA	FALSE
GB10869	7762859	4520133	0.780222861	-0.084294648	FALSE
GB10870	1998	1125	0.828631582	-0.035885927	FALSE
GB10871	492	289	0.767588823	-0.096928685	FALSE
GB10872	696	422	0.721844307	-0.142673201	FALSE
GB10873	265	158	0.746067801	-0.118449707	FALSE

GB10874	138	84	0.716207034	-0.148310474	FALSE
GB10875	30	9	1.736965594	0.872448086	TRUE
GB10876	1	0	NA	NA	FALSE
GB10877	500	269	0.894321922	0.029804414	FALSE
GB10878	1765	889	0.989412859	0.124895351	FALSE
GB10879	62	35	0.824913293	-0.039604215	FALSE
GB10880	140	59	1.246639968	0.382122459	FALSE
GB10881	482	353	0.449364963	-0.415152545	FALSE
GB10882	402	227	0.824503204	-0.040014304	FALSE
GB10883	916	522	0.811297792	-0.053219717	FALSE
GB10884	556	324	0.77909107	-0.085426438	FALSE
GB10885	9	5	0.847996907	-0.016520602	FALSE
GB10886	2472	1503	0.717833734	-0.146683774	FALSE
GB10887	478	292	0.711042249	-0.153475259	FALSE
GB10888	1019	525	0.956764724	0.092247215	FALSE
GB10889	1727	1066	0.696060645	-0.168456864	FALSE
GB10890	365	144	1.341827652	0.477310144	FALSE
GB10891	593	211	1.490789106	0.626271598	TRUE
GB10892	NA	NA	NA	NA	FALSE
GB10893	NA	NA	NA	NA	FALSE
GB10894	170	140	0.280107919	-0.584409589	TRUE
GB10895	135	76	0.828888084	-0.035629425	FALSE
GB10896	583	355	0.715676859	-0.14884065	FALSE
GB10897	45	22	1.032421478	0.167903969	FALSE
GB10898	473	300	0.656877683	-0.207639825	FALSE
GB10899	1087	612	0.828748382	-0.035769126	FALSE
GB10900	142	53	1.421826665	0.557309157	TRUE
GB10901	NA	NA	NA	NA	FALSE
GB10902	1483	604	1.295898143	0.431380635	FALSE
GB10903	12972	8322	0.640398735	-0.224118773	FALSE
GB10904	503	284	0.82466747	-0.039850038	FALSE
GB10905	7148	2608	1.454595765	0.590078256	TRUE
GB10906	155	134	0.210035215	-0.654482294	TRUE
GB10907	357	194	0.879867422	0.015349914	FALSE
GB10908	738	349	1.08039378	0.215876272	FALSE
GB10909	345	199	0.793827931	-0.070689577	FALSE
GB10910	10280	5473	0.909436503	0.044918995	FALSE
GB10911	97	53	0.871992388	0.007474879	FALSE
GB10912	48	21	1.192645078	0.32812757	FALSE
GB10913	27598	16774	0.718336959	-0.146180549	FALSE
GB10914	557	362	0.62168763	-0.242829878	FALSE
GB10915	583	375	0.636605288	-0.227912221	FALSE
GB10916	703	500	0.491596594	-0.372920914	FALSE
GB10917	5065	2888	0.810491527	-0.054025982	FALSE

GB10918	539	310	0.798017057	-0.066500451	FALSE
GB10919	86	72	0.256339753	-0.608177755	TRUE
GB10920	203	116	0.807354922	-0.057162586	FALSE
GB10921	27	16	0.754887502	-0.109630006	FALSE
GB10922	958	608	0.655954332	-0.208563176	FALSE
GB10923	156	93	0.746243408	-0.118274101	FALSE
GB10924	18	11	0.710493383	-0.154024126	FALSE
GB10925	NA	NA	NA	NA	FALSE
GB10926	415	337	0.300362745	-0.564154763	TRUE
GB10927	267	187	0.513801472	-0.350716037	FALSE
GB10928	1746	1004	0.79829429	-0.066223219	FALSE
GB10929	125	49	1.351074441	0.486556932	FALSE
GB10930	NA	NA	NA	NA	FALSE
GB10931	224	100	1.163498732	0.298981224	FALSE
GB10932	413	187	1.143103512	0.278586003	FALSE
GB10933	122	63	0.953457414	0.088939906	FALSE
GB10934	455	334	0.446018443	-0.418499066	FALSE
GB10935	351	193	0.862870183	-0.001647325	FALSE
GB10936	715	441	0.697164586	-0.167352922	FALSE
GB10937	1013	564	0.844867106	-0.019650402	FALSE
GB10938	662	503	0.396272817	-0.468244691	FALSE
GB10939	27513	12664	1.119380298	0.25486279	FALSE
GB10940	1890	1025	0.882762325	0.018244816	FALSE
GB10941	580	302	0.941504351	0.076986842	FALSE
GB10942	1318	905	0.542360673	-0.322156835	FALSE
GB10943	54	28	0.94753258	0.083015072	FALSE
GB10944	681	408	0.739085646	-0.125431862	FALSE
GB10945	26307	11089	1.246317466	0.381799957	FALSE
GB10946	3738	2513	0.572855898	-0.29166161	FALSE
GB10947	452	386	0.227721925	-0.636795583	TRUE
GB10948	NA	NA	NA	NA	FALSE
GB10949	950	607	0.646230997	-0.218286511	FALSE
GB10950	347	221	0.650889293	-0.213628215	FALSE
GB10951	254	129	0.977457431	0.112939923	FALSE
GB10952	194	138	0.491388385	-0.373129123	FALSE
GB10953	604	417	0.534501166	-0.330016342	FALSE
GB10954	461	238	0.953805177	0.089287669	FALSE
GB10955	892	499	0.838003895	-0.026513614	FALSE
GB10956	150	103	0.542318163	-0.322199345	FALSE
GB10957	902	513	0.814168608	-0.050348901	FALSE
GB10958	451	211	1.095884435	0.231366926	FALSE
GB10959	2660	1435	0.890375509	0.025858	FALSE
GB10960	316	207	0.610293791	-0.254223718	FALSE
GB10961	507	331	0.61515453	-0.249362978	FALSE

GB10962	NA	NA	NA	NA	FALSE
GB10963	NA	NA	NA	NA	FALSE
GB10964	63	33	0.932885804	0.068368296	FALSE
GB10965	63	25	1.333423734	0.468906225	FALSE
GB10966	928	539	0.783839532	-0.080677976	FALSE
GB10967	1126	636	0.824108157	-0.040409352	FALSE
GB10968	1059	774	0.452297118	-0.41222039	FALSE
GB10969	780	503	0.632915724	-0.231601784	FALSE
GB10970	460	209	1.138130919	0.273613411	FALSE
GB10971	1047	443	1.240882838	0.37636533	FALSE
GB10972	3858	2874	0.424793082	-0.439724427	FALSE
GB10973	69325	36992	0.906162408	0.0416449	FALSE
GB10974	682	380	0.843772321	-0.020745188	FALSE
GB10975	346	212	0.706707773	-0.157809735	FALSE
GB10976	283	88	1.685226624	0.820709116	TRUE
GB10977	1010	591	0.773125257	-0.091392251	FALSE
GB10978	1226	546	1.166986123	0.302468614	FALSE
GB10979	492	237	1.053771256	0.189253748	FALSE
GB10980	773	389	0.990698259	0.126180751	FALSE
GB10981	94	46	1.031026896	0.166509387	FALSE
GB10982	7420	3723	0.994953571	0.130436062	FALSE
GB10983	942	469	1.006139137	0.141621629	FALSE
GB10984	331	214	0.62922042	-0.235297088	FALSE
GB10985	NA	NA	NA	NA	FALSE
GB10986	944	571	0.725296114	-0.139221394	FALSE
GB10987	628	376	0.740031897	-0.124485611	FALSE
GB10988	497	245	1.020464103	0.155946594	FALSE
GB10989	6592	2930	1.169815578	0.30529807	FALSE
GB10990	NA	NA	NA	NA	FALSE
GB10991	344	169	1.025385318	0.16086781	FALSE
GB10992	15579	16633	-0.094445772	-0.95896328	TRUE
GB10993	117	70	0.741081703	-0.123435806	FALSE
GB10994	215	82	1.390640845	0.526123337	TRUE
GB10995	2419	1149	1.074031971	0.209514463	FALSE
GB10996	213	119	0.839891857	-0.024625651	FALSE
GB10997	28	16	0.807354922	-0.057162586	FALSE
GB10998	NA	NA	NA	NA	FALSE
GB10999	587	340	0.787825757	-0.076691751	FALSE
GB11000	372	261	0.511252815	-0.353264694	FALSE
GB11001	1006	756	0.412172166	-0.452345343	FALSE
GB11002	477	187	1.350950996	0.486433488	FALSE
GB11003	1147	611	0.908621106	0.044103598	FALSE
GB11004	5	5	0	-0.864517508	TRUE
GB11005	628	313	1.004601902	0.140084394	FALSE

GB11006	NA	NA	NA	NA	FALSE
GB11007	1726	927	0.896791221	0.032273712	FALSE
GB11008	369	207	0.833990049	-0.03052746	FALSE
GB11009	248	109	1.186011986	0.321494477	FALSE
GB11010	310	166	0.901084974	0.036567466	FALSE
GB11011	84	31	1.438121112	0.573603604	TRUE
GB11012	558	324	0.784271309	-0.080246199	FALSE
GB11013	3	4	-0.415037499	-1.279555008	TRUE
GB11014	98	103	-0.071790683	-0.936308191	TRUE
GB11015	7	4	0.807354922	-0.057162586	FALSE
GB11016	1726	865	0.996660427	0.132142918	FALSE
GB11017	3551	2716	0.386741881	-0.477775628	FALSE
GB11018	375	154	1.283960245	0.419442736	FALSE
GB11019	1484	883	0.749005749	-0.115511759	FALSE
GB11020	163	99	0.719371534	-0.145145974	FALSE
GB11021	313	179	0.80620307	-0.058314439	FALSE
GB11022	146	58	1.331843564	0.467326055	FALSE
GB11023	28827	16834	0.776042682	-0.088474826	FALSE
GB11024	346	205	0.755148128	-0.10936938	FALSE
GB11025	191	107	0.835961842	-0.028555667	FALSE
GB11026	38	24	0.662965013	-0.201552496	FALSE
GB11027	8	17	-1.087462841	-1.95198035	TRUE
GB11028	29299	14440	1.020780683	0.156263175	FALSE
GB11029	26544	17763	0.579510532	-0.285006977	FALSE
GB11030	NA	NA	NA	NA	FALSE
GB11031	8	13	-0.700439718	-1.564957226	TRUE
GB11032	415	220	0.915607813	0.051090304	FALSE
GB11033	387	150	1.367371066	0.502853557	TRUE
GB11034	729	423	0.785261151	-0.079256357	FALSE
GB11035	1634	875	0.901053061	0.036535553	FALSE
GB11036	28	16	0.807354922	-0.057162586	FALSE
GB11037	940	487	0.948738984	0.084221476	FALSE
GB11038	431	216	0.996656557	0.132139049	FALSE
GB11039	245	150	0.707819249	-0.15669826	FALSE
GB11040	10824	7582	0.513583383	-0.350934126	FALSE
GB11041	531	293	0.857811196	-0.006706312	FALSE
GB11042	381	179	1.08983141	0.225313902	FALSE
GB11043	203	91	1.157541277	0.293023769	FALSE
GB11044	NA	NA	NA	NA	FALSE
GB11045	288	142	1.020177882	0.155660374	FALSE
GB11046	2121	1011	1.068961624	0.204444115	FALSE
GB11047	636	355	0.841207741	-0.023309767	FALSE
GB11048	687	372	0.885007478	0.020489969	FALSE
GB11049	30	15	1	0.135482492	FALSE

GB11050	1427	744	0.939610808	0.0750933	FALSE
GB11051	88	42	1.067114196	0.202596688	FALSE
GB11052	NA	NA	NA	NA	FALSE
GB11053	141	82	0.781999348	-0.082518161	FALSE
GB11054	7560	3860	0.969785387	0.105267879	FALSE
GB11055	125660	82082	0.614387696	-0.250129812	FALSE
GB11056	6296	5538	0.185070487	-0.679447021	TRUE
GB11057	681	340	1.002120052	0.137602544	FALSE
GB11058	1089	377	1.530367525	0.665850017	TRUE
GB11059	752144	395773	0.926335702	0.061818194	FALSE
GB11060	117	53	1.142444265	0.277926757	FALSE
GB11061	765	566	0.434657695	-0.429859814	FALSE
GB11062	40	32	0.321928095	-0.542589413	TRUE
GB11063	2	0	NA	NA	FALSE
GB11064	135	47	1.522226745	0.657709237	TRUE
GB11065	85	42	1.017073513	0.152556005	FALSE
GB11066	81	25	1.695993813	0.831476305	TRUE
GB11067	151	78	0.95300252	0.088485012	FALSE
GB11068	59	28	1.075288127	0.210770619	FALSE
GB11069	217	129	0.750323977	-0.114193531	FALSE
GB11070	1166	546	1.094594932	0.230077424	FALSE
GB11071	317	218	0.540154705	-0.324362803	FALSE
GB11072	1719	888	0.952937963	0.088420455	FALSE
GB11073	162	88	0.880418384	0.015900876	FALSE
GB11074	3388	1605	1.077860577	0.213343069	FALSE
GB11075	47	13	1.854149134	0.989631625	TRUE
GB11076	30664	19299	0.668019809	-0.1964977	FALSE
GB11077	881	511	0.785818728	-0.07869878	FALSE
GB11078	420	250	0.748461233	-0.116056275	FALSE
GB11079	722	528	0.451460908	-0.413056601	FALSE
GB11080	294	136	1.112209504	0.247691995	FALSE
GB11081	223	151	0.562495161	-0.302022348	FALSE
GB11082	5827	3093	0.913746477	0.049228969	FALSE
GB11083	1	2	-1	-1.864517508	TRUE
GB11084	249	164	0.602449927	-0.262067581	FALSE
GB11085	1055	503	1.068612694	0.204095185	FALSE
GB11086	4368	2521	0.792976738	-0.07154077	FALSE
GB11087	259	179	0.53299251	-0.331524998	FALSE
GB11088	1829	763	1.261300113	0.396782605	FALSE
GB11089	11	2	2.459431619	1.59491411	TRUE
GB11090	NA	NA	NA	NA	FALSE
GB11091	151	103	0.551904212	-0.312613296	FALSE
GB11092	4	6	-0.584962501	-1.449480009	TRUE
GB11093	45	11	2.032421478	1.167903969	TRUE

GB11094	NA	NA	NA	NA	FALSE
GB11095	NA	NA	NA	NA	FALSE
GB11096	432	232	0.896906507	0.032388999	FALSE
GB11097	0	1	NA	NA	FALSE
GB11098	98	63	0.637429921	-0.227087588	FALSE
GB11099	30	8	1.906890596	1.042373087	TRUE
GB11100	655	424	0.627430642	-0.237086866	FALSE
GB11101	745	430	0.792903766	-0.071613743	FALSE
GB11102	373	173	1.108403593	0.243886084	FALSE
GB11103	925	572	0.693438219	-0.17107929	FALSE
GB11104	45	32	0.491853096	-0.372664412	FALSE
GB11105	329	282	0.222392421	-0.642125087	TRUE
GB11106	2900	1796	0.69126555	-0.173251958	FALSE
GB11107	3341	1933	0.789438345	-0.075079163	FALSE
GB11108	198	145	0.44944753	-0.415069978	FALSE
GB11109	NA	NA	NA	NA	FALSE
GB11110	155	88	0.816692787	-0.047824722	FALSE
GB11111	369	224	0.720122084	-0.144395424	FALSE
GB11112	1283	555	1.208961494	0.344443986	FALSE
GB11113	493	334	0.561739544	-0.302777964	FALSE
GB11114	2631	1480	0.830014073	-0.034503436	FALSE
GB11115	1	0	NA	NA	FALSE
GB11116	430	188	1.193603998	0.32908649	FALSE
GB11117	3592	2085	0.784739966	-0.079777542	FALSE
GB11118	411	237	0.794251335	-0.070266174	FALSE
GB11119	NA	NA	NA	NA	FALSE
GB11120	1646	883	0.898478993	0.033961484	FALSE
GB11121	36	16	1.169925001	0.305407493	FALSE
GB11122	1389	718	0.95199085	0.087473342	FALSE
GB11123	274	158	0.794251335	-0.070266174	FALSE
GB11124	337	238	0.501787018	-0.36273049	FALSE
GB11125	1912	837	1.191782995	0.327265487	FALSE
GB11126	40	5	3	2.135482492	TRUE
GB11127	364	201	0.856742949	-0.007774559	FALSE
GB11128	925	525	0.817135943	-0.047381565	FALSE
GB11129	2287	1091	1.067805264	0.203287756	FALSE
GB11130	1568	810	0.952931746	0.088414238	FALSE
GB11131	112	74	0.597901556	-0.266615952	FALSE
GB11132	35378	23719	0.576809305	-0.287708204	FALSE
GB11133	715	428	0.740332445	-0.124185063	FALSE
GB11134	1576	978	0.688361165	-0.176156344	FALSE
GB11135	20	11	0.862496476	-0.002021032	FALSE
GB11136	48	17	1.497499659	0.632982151	TRUE
GB11137	791	505	0.647394307	-0.217123201	FALSE

GB11138	NA	NA	NA	NA	FALSE
GB11139	109	93	0.229025514	-0.635491995	TRUE
GB11140	2	2	0	-0.864517508	TRUE
GB11141	711	389	0.870079405	0.005561896	FALSE
GB11142	14	5	1.485426827	0.620909319	TRUE
GB11143	547	308	0.828610482	-0.035907026	FALSE
GB11144	860	652	0.399464695	-0.465052813	FALSE
GB11145	904	503	0.845764373	-0.018753136	FALSE
GB11146	949	585	0.697971463	-0.166546046	FALSE
GB11147	12	6	1	0.135482492	FALSE
GB11148	3	0	NA	NA	FALSE
GB11149	1486	843	0.81782958	-0.046687929	FALSE
GB11150	2456	1531	0.681836278	-0.18268123	FALSE
GB11151	741	494	0.584962501	-0.279555008	FALSE
GB11152	126	31	2.023083613	1.158566105	TRUE
GB11153	271	126	1.104869118	0.24035161	FALSE
GB11154	146	52	1.489384841	0.624867332	TRUE
GB11155	2	0	NA	NA	FALSE
GB11156	1272	748	0.765988495	-0.098529013	FALSE
GB11157	671	401	0.74271053	-0.121806979	FALSE
GB11158	518	248	1.062611977	0.198094469	FALSE
GB11159	157	100	0.650764559	-0.213752949	FALSE
GB11160	350	218	0.683026787	-0.181490721	FALSE
GB11161	664	359	0.887199398	0.022681889	FALSE
GB11162	642	493	0.380985651	-0.483531858	FALSE
GB11163	70	30	1.222392421	0.357874913	FALSE
GB11164	1	0	NA	NA	FALSE
GB11165	338	265	0.351030887	-0.513486621	TRUE
GB11166	875	397	1.14014401	0.275626501	FALSE
GB11167	77	47	0.712197689	-0.152319819	FALSE
GB11168	1613	747	1.11056629	0.246048782	FALSE
GB11169	60	14	2.099535674	1.235018165	TRUE
GB11170	948	493	0.943299413	0.078781904	FALSE
GB11171	633	367	0.786425437	-0.078092072	FALSE
GB11172	148	69	1.100928909	0.236411401	FALSE
GB11173	398	253	0.653631046	-0.210886462	FALSE
GB11174	113	68	0.732716121	-0.131801387	FALSE
GB11175	546	266	1.037474705	0.172957197	FALSE
GB11176	2499	1350	0.888391494	0.023873986	FALSE
GB11177	7226	4974	0.538790747	-0.325726761	FALSE
GB11178	NA	NA	NA	NA	FALSE
GB11179	NA	NA	NA	NA	FALSE
GB11180	335	184	0.864455329	790392114629e-05	FALSE
GB11181	91	57	0.674904626	-0.189612882	FALSE

GB11182	5	4	0.321928095	-0.542589413	TRUE
GB11183	7066	4029	0.810471944	-0.054045565	FALSE
GB11184	321	187	0.779535027	-0.084982481	FALSE
GB11185	NA	NA	NA	NA	FALSE
GB11186	413	235	0.813481025	-0.051036483	FALSE
GB11187	15	6	1.321928095	0.457410587	FALSE
GB11188	53	21	1.335603032	0.471085523	FALSE
GB11189	630	299	1.075206344	0.210688836	FALSE
GB11190	219	132	0.73039294	-0.134124568	FALSE
GB11191	5596	3374	0.729935989	-0.134581519	FALSE
GB11192	568	298	0.930578599	0.066061091	FALSE
GB11193	NA	NA	NA	NA	FALSE
GB11194	468	259	0.853556432	-0.010961076	FALSE
GB11195	815	520	0.648288436	-0.216229072	FALSE
GB11196	86	61	0.495527417	-0.368990091	FALSE
GB11197	88	46	0.935869663	0.071352154	FALSE
GB11198	321	189	0.764187063	-0.100330445	FALSE
GB11199	10000	4284	1.222969615	0.358452106	FALSE
GB11200	360	227	0.665304609	-0.199212899	FALSE
GB11201	3707	2584	0.520646045	-0.343871463	FALSE
GB11202	6531	3626	0.848924983	-0.015592525	FALSE
GB11203	2502	1496	0.741971614	-0.122545894	FALSE
GB11204	1850	754	1.294888842	0.430371334	FALSE
GB11205	310	234	0.405759686	-0.458757823	FALSE
GB11206	373	149	1.3238633	0.459345791	FALSE
GB11207	379	163	1.217325884	0.352808376	FALSE
GB11208	230	108	1.090602549	0.22608504	FALSE
GB11209	633	299	1.082060015	0.217542507	FALSE
GB11210	228	138	0.724365557	-0.140151951	FALSE
GB11211	1374	517	1.410145819	0.54562831	TRUE
GB11212	1405	810	0.794576317	-0.069941191	FALSE
GB11213	1100	543	1.018479421	0.153961912	FALSE
GB11214	2461	1256	0.970408194	0.105890685	FALSE
GB11215	150	62	1.27462238	0.410104872	FALSE
GB11216	1	1	0	-0.864517508	TRUE
GB11217	4831	2618	0.883856755	0.019339247	FALSE
GB11218	1	0	NA	NA	FALSE
GB11219	614	346	0.827466618	-0.037050891	FALSE
GB11220	2660	1510	0.816877696	-0.047639812	FALSE
GB11221	642	460	0.480939436	-0.383578072	FALSE
GB11222	NA	NA	NA	NA	FALSE
GB11223	83	38	1.127111918	0.26259441	FALSE
GB11224	471	226	1.059404287	0.194886779	FALSE
GB11225	1743	851	1.034341532	0.169824024	FALSE

GB11226	229	124	0.885007478	0.020489969	FALSE
GB11227	14638	10473	0.483043688	-0.38147382	FALSE
GB11228	451	266	0.761701188	-0.102816321	FALSE
GB11229	17214	9193	0.904974728	0.04045722	FALSE
GB11230	374	293	0.352137605	-0.512379903	TRUE
GB11231	612	390	0.650057529	-0.214459979	FALSE
GB11232	644	377	0.772496165	-0.092021343	FALSE
GB11233	436	290	0.588275235	-0.276242274	FALSE
GB11234	10	7	0.514573173	-0.349944335	FALSE
GB11235	1	1	0	-0.864517508	TRUE
GB11236	NA	NA	NA	NA	FALSE
GB11237	5255	3178	0.72557164	-0.138945869	FALSE
GB11238	460	275	0.742202243	-0.122315266	FALSE
GB11239	51	36	0.502500341	-0.362017168	FALSE
GB11240	10555	8132	0.376244458	-0.48827305	FALSE
GB11241	371	163	1.186547222	0.322029714	FALSE
GB11242	194	106	0.871992388	0.007474879	FALSE
GB11243	59	46	0.359081093	-0.505436415	TRUE
GB11244	461	256	0.84862294	-0.015894568	FALSE
GB11245	17	4	2.087462841	1.222945333	TRUE
GB11246	759	386	0.975499038	0.11098153	FALSE
GB11247	669	439	0.607785271	-0.256732237	FALSE
GB11248	11	3	1.874469118	1.00995161	TRUE
GB11249	1391	698	0.994823478	0.13030597	FALSE
GB11250	997	569	0.809164852	-0.055352656	FALSE
GB11251	1668	828	1.010416616	0.145899108	FALSE
GB11252	7	1	2.807354922	1.942837414	TRUE
GB11253	12	2	2.584962501	1.720444992	TRUE
GB11254	95	57	0.736965594	-0.127551914	FALSE
GB11255	2	4	-1	-1.864517508	TRUE
GB11256	1	0	NA	NA	FALSE
GB11257	73	40	0.867896464	0.003378956	FALSE
GB11258	345	217	0.668901319	-0.195616189	FALSE
GB11259	33	18	0.874469118	0.00995161	FALSE
GB11260	4008	2274	0.817650254	-0.046867254	FALSE
GB11261	2247	1438	0.643936449	-0.22058106	FALSE
GB11262	117	48	1.285402219	0.420884711	FALSE
GB11263	4950	2925	0.7589919	-0.105525608	FALSE
GB11264	2	2	0	-0.864517508	TRUE
GB11265	278	161	0.788024195	-0.076493314	FALSE
GB11266	79	50	0.659924558	-0.20459295	FALSE
GB11267	35	18	0.959358016	0.094840507	FALSE
GB11268	1027	485	1.082379529	0.217862021	FALSE
GB11269	1778	738	1.268562603	0.404045094	FALSE

GB11270	725	414	0.808350227	-0.056167281	FALSE
GB11271	255	87	1.551409941	0.686892433	TRUE
GB11272	656	409	0.681594972	-0.182922537	FALSE
GB11273	46266	30297	0.610777428	-0.25374008	FALSE
GB11274	804	610	0.398386259	-0.46613125	FALSE
GB11275	96	45	1.093109404	0.228591896	FALSE
GB11276	963	472	1.028748938	0.16423143	FALSE
GB11277	1670	1089	0.616844149	-0.24767336	FALSE
GB11278	2959	1604	0.883435555	0.018918046	FALSE
GB11279	2352	1344	0.807354922	-0.057162586	FALSE
GB11280	11049	5756	0.940777306	0.076259798	FALSE
GB11281	19	14	0.440572591	-0.423944917	FALSE
GB11282	3285	1613	1.026146932	0.161629424	FALSE
GB11283	15963	11911	0.422437268	-0.44208024	FALSE
GB11284	4547	2884	0.65684384	-0.207673669	FALSE
GB11285	556	319	0.801528459	-0.062989049	FALSE
GB11286	15237	7837	0.959205478	0.094687969	FALSE
GB11287	59	40	0.560714954	-0.303802554	FALSE
GB11288	273	109	1.324572816	0.460055308	FALSE
GB11289	1051	717	0.551717645	-0.312799863	FALSE
GB11290	103	32	1.686500527	0.821983019	TRUE
GB11291	1213	605	1.003572503	0.139054995	FALSE
GB11292	3	2	0.584962501	-0.279555008	FALSE
GB11293	19	9	1.078002512	0.213485004	FALSE
GB11294	574	315	0.865698908	0.0011814	FALSE
GB11295	157	81	0.954770746	0.090253238	FALSE
GB11296	353	248	0.509328063	-0.355189445	FALSE
GB11297	1	0	NA	NA	FALSE
GB11298	12262	4903	1.322457643	0.457940135	FALSE
GB11299	18655	12195	0.613272602	-0.251244906	FALSE
GB11300	213	127	0.746024933	-0.118492575	FALSE
GB11301	4629	2350	0.978039806	0.113522298	FALSE
GB11302	0	1	NA	NA	FALSE
GB11303	1081	531	1.025582757	0.161065249	FALSE
GB11304	3463	1721	1.00877529	0.144257782	FALSE
GB11305	248	139	0.835255238	-0.029262271	FALSE
GB11306	449	239	0.909704827	0.045187318	FALSE
GB11307	300	215	0.480625841	-0.383891667	FALSE
GB11308	863	391	1.142191952	0.277674444	FALSE
GB11309	1074	590	0.864207134	-0.000310375	FALSE
GB11310	1	0	NA	NA	FALSE
GB11311	235	102	1.204091605	0.339574096	FALSE
GB11312	208	134	0.634350528	-0.230166981	FALSE
GB11313	12	18	-0.584962501	-1.449480009	TRUE

GB11314	1751	537	1.70518509	0.840667582	TRUE
GB11315	1874	931	1.00926788	0.144750372	FALSE
GB11316	18	4	2.169925001	1.305407493	TRUE
GB11317	3	0	NA	NA	FALSE
GB11318	3	0	NA	NA	FALSE
GB11319	73	44	0.73039294	-0.134124568	FALSE
GB11320	398	284	0.486877501	-0.377640007	FALSE
GB11321	37	18	1.039528364	0.175010856	FALSE
GB11322	943	742	0.345838584	-0.518678924	TRUE
GB11323	770	380	1.018859027	0.154341519	FALSE
GB11324	155	107	0.534657419	-0.329860089	FALSE
GB11325	4405	2519	0.806290897	-0.058226611	FALSE
GB11326	2268	1234	0.878078246	0.013560737	FALSE
GB11327	169	130	0.378511623	-0.486005885	FALSE
GB11328	1041	447	1.219623332	0.355105824	FALSE
GB11329	436	321	0.441754838	-0.422762671	FALSE
GB11330	542	338	0.681269605	-0.183247903	FALSE
GB11331	315	117	1.428843299	0.56432579	TRUE
GB11332	928	504	0.880701072	0.016183563	FALSE
GB11333	85	30	1.502500341	0.637982832	TRUE
GB11334	870	459	0.922521247	0.058003739	FALSE
GB11335	2452	1395	0.813693857	-0.050823651	FALSE
GB11336	2408	1398	0.784471031	-0.080046477	FALSE
GB11337	NA	NA	NA	NA	FALSE
GB11338	NA	NA	NA	NA	FALSE
GB11339	6	10	-0.736965594	-1.601483102	TRUE
GB11340	150	80	0.906890596	0.042373087	FALSE
GB11341	21	7	1.584962501	0.720444992	TRUE
GB11342	NA	NA	NA	NA	FALSE
GB11343	2346	1351	0.796175339	-0.06834217	FALSE
GB11344	1234	675	0.870382987	0.005865479	FALSE
GB11345	408	252	0.695145418	-0.16937209	FALSE
GB11346	NA	NA	NA	NA	FALSE
GB11347	584	308	0.923038018	0.05852051	FALSE
GB11348	1333	1062	0.327893014	-0.536624494	TRUE
GB11349	689	313	1.138341326	0.273823817	FALSE
GB11350	604	399	0.598159803	-0.266357705	FALSE
GB11351	6	4	0.584962501	-0.279555008	FALSE
GB11352	249	139	0.841060859	-0.023456649	FALSE
GB11353	2	2	0	-0.864517508	TRUE
GB11354	692	364	0.926833587	0.062316079	FALSE
GB11355	3216	1914	0.748676577	-0.115840932	FALSE
GB11356	474	269	0.817280886	-0.047236622	FALSE
GB11357	1980	1125	0.815575429	-0.048942079	FALSE

GB11358	84406	34427	1.293805088	0.42928758	FALSE
GB11359	767	385	0.994368132	0.129850624	FALSE
GB11360	1028	345	1.575171998	0.710654489	TRUE
GB11361	2	7	-1.807354922	-2.67187243	TRUE
GB11362	1632	946	0.786728969	-0.07778854	FALSE
GB11363	1268	687	0.884172741	0.019655233	FALSE
GB11364	4044	1954	1.04935253	0.184835022	FALSE
GB11365	344	185	0.894883294	0.030365786	FALSE
GB11366	559	333	0.747326106	-0.117191403	FALSE
GB11367	NA	NA	NA	NA	FALSE
GB11368	599	307	0.964317347	0.099799839	FALSE
GB11369	243	157	0.630191755	-0.234325754	FALSE
GB11370	4968	2511	0.984403145	0.119885637	FALSE
GB11371	17	10	0.765534746	-0.098982762	FALSE
GB11372	2104	1341	0.649825467	-0.214692041	FALSE
GB11373	2436	1249	0.963740656	0.099223148	FALSE
GB11374	532	360	0.563429339	-0.301088169	FALSE
GB11375	218	128	0.768184325	-0.096333184	FALSE
GB11376	158	68	1.216317907	0.351800399	FALSE
GB11377	276	152	0.860596943	-0.003920565	FALSE
GB11378	160	74	1.112474729	0.247957221	FALSE
GB11379	894	501	0.835464228	-0.02905328	FALSE
GB11380	1861	871	1.095333431	0.230815923	FALSE
GB11381	NA	NA	NA	NA	FALSE
GB11382	21	3	2.807354922	1.942837414	TRUE
GB11383	141	81	0.79970135	-0.064816159	FALSE
GB11384	22	12	0.874469118	0.00995161	FALSE
GB11385	8628	5542	0.638619466	-0.225898043	FALSE
GB11386	6167	3729	0.725780049	-0.138737459	FALSE
GB11387	295	152	0.956643631	0.092126122	FALSE
GB11388	479	250	0.938097561	0.073580053	FALSE
GB11389	47	31	0.600392541	-0.264124967	FALSE
GB11390	NA	NA	NA	NA	FALSE
GB11391	1068	627	0.768374299	-0.096143209	FALSE
GB11392	10	1	3.321928095	2.457410587	TRUE
GB11393	394	243	0.697239316	-0.167278192	FALSE
GB11394	1600	784	1.029146346	0.164628837	FALSE
GB11395	180	95	0.921997488	0.05747998	FALSE
GB11396	495	179	1.467468938	0.602951429	TRUE
GB11397	7529	4801	0.649123323	-0.215394185	FALSE
GB11398	800	433	0.885632975	0.021115467	FALSE
GB11399	415	193	1.104510489	0.239992981	FALSE
GB11400	994	527	0.91544289	0.050925382	FALSE
GB11401	12	1	3.584962501	2.720444992	TRUE

GB11402	306	118	1.374744793	0.510227285	TRUE
GB11403	254	192	0.403722186	-0.460795322	FALSE
GB11404	1919	885	1.116605351	0.252087843	FALSE
GB11405	137	73	0.908207524	0.043690016	FALSE
GB11406	334	165	1.017382078	0.15286457	FALSE
GB11407	18933	9836	0.944759389	0.080241881	FALSE
GB11408	20091	11316	0.828185292	-0.036332216	FALSE
GB11409	2509	1420	0.821221541	-0.043295967	FALSE
GB11410	16930	8323	1.024406431	0.159888923	FALSE
GB11411	285	142	1.00507099	0.140553481	FALSE
GB11412	1225	453	1.435198794	0.570681286	TRUE
GB11413	681	340	1.002120052	0.137602544	FALSE
GB11414	2693	1772	0.603835626	-0.260681882	FALSE
GB11415	411	205	1.003514484	0.138996976	FALSE
GB11416	0	1	NA	NA	FALSE
GB11417	187	85	1.137503524	0.272986015	FALSE
GB11418	7	12	-0.777607579	-1.642125087	TRUE
GB11419	103	77	0.419713986	-0.444803522	FALSE
GB11420	104	38	1.452512205	0.587994696	TRUE
GB11421	522	280	0.89862298	0.034105471	FALSE
GB11422	NA	NA	NA	NA	FALSE
GB11423	1902	1098	0.792639192	-0.071878316	FALSE
GB11424	257	135	0.928808952	0.064291444	FALSE
GB11425	106	69	0.619395998	-0.245121511	FALSE
GB11426	1418	729	0.959866813	0.095349305	FALSE
GB11427	421	288	0.547751422	-0.316766087	FALSE
GB11428	268	125	1.100304906	0.235787397	FALSE
GB11429	5856	3082	0.926048692	0.061531183	FALSE
GB11430	1	0	NA	NA	FALSE
GB11431	1499	720	1.057931572	0.193414063	FALSE
GB11432	15290	10012	0.610858211	-0.253659298	FALSE
GB11433	458	244	0.908466451	0.043948942	FALSE
GB11434	1013	559	0.857713986	-0.006803522	FALSE
GB11435	322	194	0.731004036	-0.133513472	FALSE
GB11436	8	1	3	2.135482492	TRUE
GB11437	942	492	0.937068744	0.072551236	FALSE
GB11438	23	8	1.523561956	0.659044448	TRUE
GB11439	161	68	1.243454037	0.378936529	FALSE
GB11440	1	0	NA	NA	FALSE
GB11441	312	190	0.715546611	-0.148970898	FALSE
GB11442	1	1	0	-0.864517508	TRUE
GB11443	37	14	1.402098444	0.537580935	TRUE
GB11444	174	86	1.016678741	0.152161233	FALSE
GB11445	740	413	0.841383489	-0.023134019	FALSE

GB11446	71	28	1.342392197	0.477874689	FALSE
GB11447	1	0	NA	NA	FALSE
GB11448	756	528	0.517848305	-0.346669203	FALSE
GB11449	1	3	-1.584962501	-2.449480009	TRUE
GB11450	23	7	1.716207034	0.851689526	TRUE
GB11451	3738	2110	0.82502357	-0.039493938	FALSE
GB11452	5742	2472	1.215874587	0.351357079	FALSE
GB11453	1299	678	0.938044252	0.073526744	FALSE
GB11454	1163	727	0.677823828	-0.186693681	FALSE
GB11455	164	90	0.865698908	0.0011814	FALSE
GB11456	5885	3753	0.648998125	-0.215519383	FALSE
GB11457	81	42	0.94753258	0.083015072	FALSE
GB11458	255	159	0.681470482	-0.183047027	FALSE
GB11459	485	285	0.767022828	-0.09749468	FALSE
GB11460	199	119	0.741806857	-0.122710651	FALSE
GB11461	24851	15381	0.692154603	-0.172362905	FALSE
GB11462	1005	681	0.561468798	-0.30304871	FALSE
GB11463	49	29	0.756728849	-0.107788659	FALSE
GB11464	306	203	0.592051926	-0.272465583	FALSE
GB11465	593	313	0.921869448	0.057351939	FALSE
GB11466	1013	599	0.758006266	-0.106511242	FALSE
GB11467	53	13	2.027480736	1.162963228	TRUE
GB11468	432	185	1.223506042	0.358988533	FALSE
GB11469	NA	NA	NA	NA	FALSE
GB11470	484	275	0.815575429	-0.048942079	FALSE
GB11471	1119	470	1.251477374	0.386959866	FALSE
GB11472	0	1	NA	NA	FALSE
GB11473	128	95	0.430144392	-0.434373117	FALSE
GB11474	15	6	1.321928095	0.457410587	FALSE
GB11475	337	181	0.896758894	0.032241386	FALSE
GB11476	227	116	0.968567492	0.104049984	FALSE
GB11477	264	145	0.864485029	[illegible]789838141992e-05	FALSE
GB11478	2166	1178	0.878693704	0.014176195	FALSE
GB11479	212	129	0.716693199	-0.147824309	FALSE
GB11480	857	493	0.797707558	-0.066809951	FALSE
GB11481	1231	644	0.934698168	0.07018066	FALSE
GB11482	3953	2602	0.603326993	-0.261190515	FALSE
GB11483	665	299	1.153208856	0.288691348	FALSE
GB11484	349	205	0.767603127	-0.096914382	FALSE
GB11485	2302	1471	0.646090587	-0.218426921	FALSE
GB11486	28	9	1.637429921	0.772912412	TRUE
GB11487	34	25	0.443606651	-0.420910857	FALSE
GB11488	675	311	1.117972922	0.253455413	FALSE
GB11489	2148	1568	0.454068434	-0.410449074	FALSE

GB11490	86	57	0.593374741	-0.271142768	FALSE
GB11491	0	3	NA	NA	FALSE
GB11492	1023	614	0.736495584	-0.128021924	FALSE
GB11493	88	23	1.935869663	1.071352154	TRUE
GB11494	65	37	0.812914447	-0.051603061	FALSE
GB11495	505	295	0.775568433	-0.088949075	FALSE
GB11496	1113	682	0.706609948	-0.15790756	FALSE
GB11497	4218	2315	0.865546902	0.001029393	FALSE
GB11498	2253	1425	0.660885394	-0.203632114	FALSE
GB11499	223	102	1.128474558	0.26395705	FALSE
GB11500	1221	699	0.80469884	-0.059818669	FALSE
GB11501	NA	NA	NA	NA	FALSE
GB11502	923	419	1.139380404	0.274862896	FALSE
GB11503	9013	4687	0.943342596	0.078825088	FALSE
GB11504	762	447	0.769516166	-0.095001342	FALSE
GB11505	NA	NA	NA	NA	FALSE
GB11506	2201	1053	1.063653709	0.199136201	FALSE
GB11507	6	10	-0.736965594	-1.601483102	TRUE
GB11508	39	18	1.115477217	0.250959709	FALSE
GB11509	5149	2379	1.113936998	0.24941949	FALSE
GB11510	559	286	0.966833136	0.102315628	FALSE
GB11511	128	62	1.04580369	0.181286181	FALSE
GB11512	73	41	0.832272554	-0.032244954	FALSE
GB11513	2387	1542	0.630395801	-0.234121707	FALSE
GB11514	626	332	0.914979416	0.050461907	FALSE
GB11515	700	389	0.847584767	-0.016932741	FALSE
GB11516	567	437	0.375715455	-0.488802053	FALSE
GB11517	1	0	NA	NA	FALSE
GB11518	NA	NA	NA	NA	FALSE
GB11519	226	129	0.808951707	-0.055565801	FALSE
GB11520	2142	1371	0.643729909	-0.220787599	FALSE
GB11521	2	0	NA	NA	FALSE
GB11522	1352	656	1.043327432	0.178809923	FALSE
GB11523	NA	NA	NA	NA	FALSE
GB11524	NA	NA	NA	NA	FALSE
GB11525	2217	1235	0.844097728	-0.02041978	FALSE
GB11526	642	341	0.912801558	0.04828405	FALSE
GB11527	801	378	1.083416008	0.2188985	FALSE
GB11528	452	297	0.605859842	-0.258657667	FALSE
GB11529	6	5	0.263034406	-0.601483102	TRUE
GB11530	1110	559	0.989639488	0.12512198	FALSE
GB11531	179	96	0.898853277	0.034335768	FALSE
GB11532	72	55	0.388565288	-0.47595222	FALSE
GB11533	1786	1277	0.483973555	-0.380543953	FALSE

GB11534	NA	NA	NA	NA	FALSE
GB11535	1336	747	0.83873986	-0.025777649	FALSE
GB11536	16	7	1.192645078	0.32812757	FALSE
GB11537	2169	1466	0.56514495	-0.299372559	FALSE
GB11538	20	12	0.736965594	-0.127551914	FALSE
GB11539	8	3	1.415037499	0.550519991	TRUE
GB11540	0	1	NA	NA	FALSE
GB11541	1	0	NA	NA	FALSE
GB11542	673	351	0.939135474	0.074617966	FALSE
GB11543	4	6	-0.584962501	-1.449480009	TRUE
GB11544	4503	2576	0.805753884	-0.058763624	FALSE
GB11545	165	100	0.722466024	-0.142051484	FALSE
GB11546	494	315	0.649159213	-0.215358295	FALSE
GB11547	604	293	1.043647885	0.179130377	FALSE
GB11548	23	7	1.716207034	0.851689526	TRUE
GB11549	1097	811	0.435789706	-0.428727802	FALSE
GB11550	1	0	NA	NA	FALSE
GB11551	15	2	2.906890596	2.042373087	TRUE
GB11552	2	0	NA	NA	FALSE
GB11553	5939	2936	1.016368064	0.151850556	FALSE
GB11554	989	519	0.930235982	0.065718474	FALSE
GB11555	1670	1128	0.566081035	-0.298436473	FALSE
GB11556	207	97	1.093574115	0.229056607	FALSE
GB11557	835	494	0.757265156	-0.107252353	FALSE
GB11558	1594	897	0.829471739	-0.035045769	FALSE
GB11559	622	372	0.741611959	-0.122905549	FALSE
GB11560	5017	3134	0.678819758	-0.18569775	FALSE
GB11561	942	634	0.571244219	-0.293273289	FALSE
GB11562	384	250	0.619178216	-0.245339292	FALSE
GB11563	164	93	0.818393194	-0.046124315	FALSE
GB11564	3269	1706	0.93823173	0.073714222	FALSE
GB11565	314	151	1.05621601	0.191698501	FALSE
GB11566	57	15	1.925999419	1.06148191	TRUE
GB11567	356	193	0.883276394	0.018758885	FALSE
GB11568	342	275	0.314564706	-0.549952802	TRUE
GB11569	179	125	0.518031493	-0.346486016	FALSE
GB11570	6	4	0.584962501	-0.279555008	FALSE
GB11571	7	1	2.807354922	1.942837414	TRUE
GB11572	5980	2637	1.181247913	0.316730405	FALSE
GB11573	3613	1673	1.110759809	0.246242301	FALSE
GB11574	3414	2303	0.567948647	-0.296568861	FALSE
GB11575	NA	NA	NA	NA	FALSE
GB11576	366	210	0.801454321	-0.063063188	FALSE
GB11577	21	6	1.807354922	0.942837414	TRUE

GB11578	1092	498	1.132755209	0.268237701	FALSE
GB11579	12	7	0.777607579	-0.08690993	FALSE
GB11580	320	132	1.277533976	0.413016467	FALSE
GB11581	702	342	1.037474705	0.172957197	FALSE
GB11582	680	354	0.941785386	0.077267878	FALSE
GB11583	280	200	0.485426827	-0.379090681	FALSE
GB11584	800	391	1.032831392	0.168313884	FALSE
GB11585	838	492	0.768291928	-0.09622558	FALSE
GB11586	415	202	1.038756043	0.174238535	FALSE
GB11587	7812	4475	0.803804267	-0.060713241	FALSE
GB11588	146867	85334	0.783317688	-0.08119982	FALSE
GB11589	1	2	-1	-1.864517508	TRUE
GB11590	838	584	0.520981875	-0.343535634	FALSE
GB11591	1218	708	0.782692868	-0.081824641	FALSE
GB11592	4	2	1	0.135482492	FALSE
GB11593	982	561	0.807722254	-0.056795255	FALSE
GB11594	731	357	1.033947332	0.169429824	FALSE
GB11595	3346	1590	1.07341068	0.208893172	FALSE
GB11596	481	382	0.332464256	-0.532053253	TRUE
GB11597	1152	824	0.483424474	-0.381093034	FALSE
GB11598	2541	1554	0.709409872	-0.155107637	FALSE
GB11599	NA	NA	NA	NA	FALSE
GB11600	481	298	0.690724563	-0.173792945	FALSE
GB11601	174	102	0.770518154	-0.093999354	FALSE
GB11602	2	0	NA	NA	FALSE
GB11603	809	276	1.551471436	0.686953927	TRUE
GB11604	165	105	0.652076697	-0.212440812	FALSE
GB11605	190	92	1.046293652	0.181776144	FALSE
GB11606	740	417	0.827477887	-0.037039621	FALSE
GB11607	15	9	0.736965594	-0.127551914	FALSE
GB11608	179	123	0.541301272	-0.323216236	FALSE
GB11609	523	243	1.105854633	0.241337124	FALSE
GB11610	1324	553	1.259551737	0.395034228	FALSE
GB11611	337	155	1.120480376	0.255962868	FALSE
GB11612	163	100	0.704871964	-0.159645544	FALSE
GB11613	595	351	0.761418638	-0.10309887	FALSE
GB11614	2784	1850	0.58963394	-0.274883568	FALSE
GB11615	1909	1225	0.640035354	-0.224482155	FALSE
GB11616	623	333	0.903709986	0.039192478	FALSE
GB11617	263	134	0.972829799	0.108312291	FALSE
GB11618	321	147	1.126757142	0.262239634	FALSE
GB11619	NA	NA	NA	NA	FALSE
GB11620	2	1	1	0.135482492	FALSE
GB11621	1504	848	0.826668397	-0.037849111	FALSE

GB11622	1285	655	0.972201548	0.107684039	FALSE
GB11623	1225	673	0.864103339	-0.000414169	FALSE
GB11624	1538	871	0.820310879	-0.044206629	FALSE
GB11625	1230	649	0.922367932	0.057850424	FALSE
GB11626	265	143	0.889977213	0.025459704	FALSE
GB11627	973	567	0.77909107	-0.085426438	FALSE
GB11628	1274	691	0.882607662	0.018090154	FALSE
GB11629	821	462	0.82948937	-0.035028138	FALSE
GB11630	75	41	0.871266686	0.006749178	FALSE
GB11631	509	302	0.753117107	-0.111400402	FALSE
GB11632	0	1	NA	NA	FALSE
GB11633	462	217	1.090197809	0.225680301	FALSE
GB11634	1213	617	0.975237156	0.110719648	FALSE
GB11635	192	97	0.985049659	0.12053215	FALSE
GB11636	82	40	1.03562391	0.171106401	FALSE
GB11637	262	120	1.126532406	0.262014898	FALSE
GB11638	1010	564	0.840588225	-0.023929283	FALSE
GB11639	6	1	2.584962501	1.720444992	TRUE
GB11640	281	177	0.66682077	-0.197696738	FALSE
GB11641	295	182	0.696776504	-0.167741004	FALSE
GB11642	9025	8030	0.168526944	-0.695990564	TRUE
GB11643	26093	18519	0.494656628	-0.36986088	FALSE
GB11644	604	341	0.82477681	-0.039740698	FALSE
GB11645	303	170	0.833783047	-0.030734461	FALSE
GB11646	481	302	0.671488344	-0.193029164	FALSE
GB11647	3546	1640	1.112496721	0.247979213	FALSE
GB11648	NA	NA	NA	NA	FALSE
GB11649	6257	2935	1.092110601	0.227593092	FALSE
GB11650	0	8	NA	NA	FALSE
GB11651	670	439	0.609940156	-0.254577353	FALSE
GB11652	2384	1725	0.466787874	-0.397729634	FALSE
GB11653	3	1	1.584962501	0.720444992	TRUE
GB11654	70	38	0.881355504	0.016837995	FALSE
GB11655	8903	5551	0.681543863	-0.182973646	FALSE
GB11656	0	1	NA	NA	FALSE
GB11657	2	3	-0.584962501	-1.449480009	TRUE
GB11658	NA	NA	NA	NA	FALSE
GB11659	2463	1652	0.576202941	-0.288314567	FALSE
GB11660	260	126	1.04508789	0.180570381	FALSE
GB11661	2558	1460	0.809047895	-0.055469613	FALSE
GB11662	1400	940	0.574694165	-0.289823343	FALSE
GB11663	2303	1316	0.807354922	-0.057162586	FALSE
GB11664	858	407	1.075948853	0.211431345	FALSE
GB11665	142033	75798	0.905994478	0.041476969	FALSE

GB11666	611	344	0.828763815	-0.035753693	FALSE
GB11667	2042	1307	0.643723725	-0.220793783	FALSE
GB11668	6	1	2.584962501	1.720444992	TRUE
GB11669	301	186	0.694460866	-0.170056643	FALSE
GB11670	790	428	0.884241857	0.019724348	FALSE
GB11671	1225	665	0.881355504	0.016837995	FALSE
GB11672	657	355	0.888074346	0.023556838	FALSE
GB11673	184	98	0.908852112	0.044334604	FALSE
GB11674	1822	1041	0.807552891	-0.056964618	FALSE
GB11675	1457	608	1.260857649	0.39634014	FALSE
GB11676	NA	NA	NA	NA	FALSE
GB11677	387	221	0.808287197	-0.056230312	FALSE
GB11678	NA	NA	NA	NA	FALSE
GB11679	10580	4172	1.342528564	0.478011056	FALSE
GB11680	355	255	0.477321778	-0.387195731	FALSE
GB11681	17	45	-1.404390255	-2.268907763	TRUE
GB11682	61	36	0.760812336	-0.103705172	FALSE
GB11683	1783	1186	0.588202693	-0.276314815	FALSE
GB11684	228	167	0.449185722	-0.415331787	FALSE
GB11685	1350	860	0.650550842	-0.213966666	FALSE
GB11686	159	113	0.492703993	-0.371813515	FALSE
GB11687	1261	721	0.806497111	-0.058020397	FALSE
GB11688	88	60	0.552541023	-0.311976485	FALSE
GB11689	3125	1913	0.708019316	-0.156498192	FALSE
GB11690	562	311	0.85365555	-0.010861958	FALSE
GB11691	252	105	1.263034406	0.398516898	FALSE
GB11692	2431	1152	1.077409177	0.212891668	FALSE
GB11693	544	313	0.797443994	-0.067073514	FALSE
GB11694	159	57	1.479992941	0.615475433	TRUE
GB11695	79	34	1.216317907	0.351800399	FALSE
GB11696	201	148	0.441598326	-0.422919183	FALSE
GB11697	336	185	0.860935962	-0.003581546	FALSE
GB11698	2687	1079	1.316301457	0.451783948	FALSE
GB11699	211	122	0.790361851	-0.074155657	FALSE
GB11700	570	450	0.341036918	-0.52348059	TRUE
GB11701	4156	2334	0.832391093	-0.032126415	FALSE
GB11702	909	530	0.778287935	-0.086229574	FALSE
GB11703	303	131	1.209750982	0.345233474	FALSE
GB11704	4523	2579	0.810468223	-0.054049285	FALSE
GB11705	2456	1021	1.266327694	0.401810186	FALSE
GB11706	7559	4238	0.834811801	-0.029705707	FALSE
GB11707	6833	4070	0.747490332	-0.117027176	FALSE
GB11708	2073	1047	0.985458674	0.120941166	FALSE
GB11709	NA	NA	NA	NA	FALSE

GB11710	1804	1019	0.824045287	-0.040472221	FALSE
GB11711	1080	516	1.065588342	0.201070833	FALSE
GB11712	4404	2661	0.726845959	-0.13767155	FALSE
GB11713	9	14	-0.637429921	-1.501947429	TRUE
GB11714	7	4	0.807354922	-0.057162586	FALSE
GB11715	723	315	1.198643819	0.33412631	FALSE
GB11716	8	2	2	1.135482492	TRUE
GB11717	1677	705	1.250187526	0.385670018	FALSE
GB11718	1556	945	0.719455826	-0.145061682	FALSE
GB11719	814	488	0.738147647	-0.126369862	FALSE
GB11720	11	2	2.459431619	1.59491411	TRUE
GB11721	187	123	0.604379955	-0.260137554	FALSE
GB11722	NA	NA	NA	NA	FALSE
GB11723	30	20	0.584962501	-0.279555008	FALSE
GB11724	45	32	0.491853096	-0.372664412	FALSE
GB11725	548	220	1.316672369	0.452154861	FALSE
GB11726	12	8	0.584962501	-0.279555008	FALSE
GB11727	10158	5016	1.018007126	0.153489617	FALSE
GB11728	1318	852	0.629425035	-0.235092474	FALSE
GB11729	47	24	0.969626351	0.105108843	FALSE
GB11730	6	0	NA	NA	FALSE
GB11731	53754	28524	0.914195805	0.049678297	FALSE
GB11732	312	153	1.028014376	0.163496868	FALSE
GB11733	726	517	0.489805268	-0.374712241	FALSE
GB11734	860	511	0.751013369	-0.11350414	FALSE
GB11735	443	151	1.552758149	0.688240641	TRUE
GB11736	NA	NA	NA	NA	FALSE
GB11737	252	109	1.209095599	0.34457809	FALSE
GB11738	346	167	1.050923935	0.186406427	FALSE
GB11739	113	68	0.732716121	-0.131801387	FALSE
GB11740	218	115	0.922694274	0.058176766	FALSE
GB11741	685	514	0.414335629	-0.45018188	FALSE
GB11742	NA	NA	NA	NA	FALSE
GB11743	781	440	0.827819025	-0.036698484	FALSE
GB11744	7032	3573	0.976799157	0.112281648	FALSE
GB11745	434	240	0.854660637	-0.009856871	FALSE
GB11746	38	11	1.788495895	0.923978386	TRUE
GB11747	208	129	0.689212463	-0.175305046	FALSE
GB11748	334	149	1.164535772	0.300018264	FALSE
GB11749	8618	5043	0.773070873	-0.091446635	FALSE
GB11750	8	4	1	0.135482492	FALSE
GB11751	249	137	0.861969849	-0.002547659	FALSE
GB11752	114	44	1.373458396	0.508940887	TRUE
GB11753	1157	557	1.054639632	0.190122123	FALSE

GB11754	3	0	NA	NA	FALSE
GB11755	254	105	1.274439169	0.409921661	FALSE
GB11756	201	129	0.639824436	-0.224693073	FALSE
GB11757	611	231	1.403279528	0.53876202	TRUE
GB11758	925	560	0.724026538	-0.14049097	FALSE
GB11759	1459	1000	0.544979883	-0.319537625	FALSE
GB11760	971	569	0.771042643	-0.093474865	FALSE
GB11761	1072	540	0.989273593	0.124756085	FALSE
GB11762	173	115	0.589138177	-0.275379332	FALSE
GB11763	225	110	1.032421478	0.167903969	FALSE
GB11764	67	22	1.606657572	0.742140063	TRUE
GB11765	277	260	0.091374353	-0.773143155	TRUE
GB11766	1322	745	0.827409846	-0.037107662	FALSE
GB11767	317	136	1.220876189	0.356358681	FALSE
GB11768	15	4	1.906890596	1.042373087	TRUE
GB11769	107	61	0.810729649	-0.053787859	FALSE
GB11770	520	317	0.714028783	-0.150488725	FALSE
GB11771	8338	4810	0.793664478	-0.07085303	FALSE
GB11772	3	8	-1.415037499	-2.279555008	TRUE
GB11773	2268	1368	0.72935241	-0.135165098	FALSE
GB11774	322	218	0.562732553	-0.301784955	FALSE
GB11775	2331	1151	1.018061171	0.153543663	FALSE
GB11776	5512	2821	0.966369222	0.101851714	FALSE
GB11777	371	146	1.345450818	0.480933309	FALSE
GB11778	1833	1051	0.802444117	-0.062073392	FALSE
GB11779	635	183	1.794912943	0.930395435	TRUE
GB11780	NA	NA	NA	NA	FALSE
GB11781	133	57	1.222392421	0.357874913	FALSE
GB11782	616	373	0.72375472	-0.140762788	FALSE
GB11783	6180	3330	0.892084661	0.027567153	FALSE
GB11784	2384	1359	0.81083878	-0.053678729	FALSE
GB11785	NA	NA	NA	NA	FALSE
GB11786	8752	4689	0.900332459	0.035814951	FALSE
GB11787	24	13	0.884522783	0.020005274	FALSE
GB11788	119	41	1.537265759	0.67274825	TRUE
GB11789	463	292	0.665043824	-0.199473684	FALSE
GB11790	586	441	0.410122009	-0.454395499	FALSE
GB11791	725	597	0.280250064	-0.584267445	TRUE
GB11792	565	250	1.176322773	0.311805264	FALSE
GB11793	96	40	1.263034406	0.398516898	FALSE
GB11794	1	0	NA	NA	FALSE
GB11795	523	229	1.191463348	0.32694584	FALSE
GB11796	1827	982	0.895681704	0.031164196	FALSE
GB11797	23	12	0.938599455	0.074081947	FALSE

GB11798	3	0	NA	NA	FALSE
GB11799	83	37	1.165586066	0.301068557	FALSE
GB11800	1	0	NA	NA	FALSE
GB11801	333	184	0.855816411	-0.008701097	FALSE
GB11802	194	150	0.371094152	-0.493423357	FALSE
GB11803	295	204	0.532145802	-0.332371706	FALSE
GB11804	918	527	0.800691192	-0.063826317	FALSE
GB11805	1540	916	0.749510847	-0.115006661	FALSE
GB11806	588	353	0.736147972	-0.128369537	FALSE
GB11807	1136	507	1.163905183	0.299387674	FALSE
GB11808	18909	8081	1.22646733	0.361949822	FALSE
GB11809	72	37	0.960471636	0.095954127	FALSE
GB11810	1158	566	1.032761295	0.168243787	FALSE
GB11811	202	80	1.336283388	0.47176588	FALSE
GB11812	791	412	0.941033357	0.076515849	FALSE
GB11813	78	52	0.584962501	-0.279555008	FALSE
GB11814	3099	2066	0.584962501	-0.279555008	FALSE
GB11815	501	317	0.660327763	-0.204189745	FALSE
GB11816	34	9	1.91753784	1.053020331	TRUE
GB11817	306	158	0.953607095	0.089089586	FALSE
GB11818	963	609	0.66109357	-0.203423938	FALSE
GB11819	501	241	1.055777457	0.191259949	FALSE
GB11820	353	169	1.062644937	0.198127429	FALSE
GB11821	451	242	0.898120386	0.033602878	FALSE
GB11822	713	269	1.406295904	0.541778396	TRUE
GB11823	648	438	0.565062943	-0.299454565	FALSE
GB11824	NA	NA	NA	NA	FALSE
GB11825	827	550	0.588455711	-0.276061798	FALSE
GB11826	1253	708	0.823565149	-0.040952359	FALSE
GB11827	532	247	1.106915204	0.242397696	FALSE
GB11828	582	261	1.156969346	0.292451838	FALSE
GB11829	2545	1381	0.881952337	0.017434828	FALSE
GB11830	2608	1665	0.647421692	-0.217095816	FALSE
GB11831	8952	5171	0.791766731	-0.072750777	FALSE
GB11832	1275	637	1.001131969	0.136614461	FALSE
GB11833	NA	NA	NA	NA	FALSE
GB11834	590	199	1.567946524	0.703429015	TRUE
GB11835	95	40	1.247927513	0.383410005	FALSE
GB11836	23	11	1.064130337	0.199612829	FALSE
GB11837	48	20	1.263034406	0.398516898	FALSE
GB11838	479	281	0.769455526	-0.095061983	FALSE
GB11839	1910	869	1.136144556	0.271627048	FALSE
GB11840	5407	2408	1.166992964	0.302475456	FALSE
GB11841	NA	NA	NA	NA	FALSE

GB11842	52	28	0.893084796	0.028567288	FALSE
GB11843	1751	950	0.882179665	0.017662157	FALSE
GB11844	69	21	1.716207034	0.851689526	TRUE
GB11845	165	83	0.991282783	0.126765275	FALSE
GB11846	2515	1207	1.059132724	0.194615216	FALSE
GB11847	177	138	0.359081093	-0.505436415	TRUE
GB11848	372	167	1.155454519	0.29093701	FALSE
GB11849	4	1	2	1.135482492	TRUE
GB11850	543	431	0.333264329	-0.53125318	TRUE
GB11851	533	245	1.121353784	0.256836275	FALSE
GB11852	649	447	0.537943647	-0.326573862	FALSE
GB11853	4534	2629	0.786270249	-0.078247259	FALSE
GB11854	2070	1133	0.869482907	0.004965398	FALSE
GB11855	421	166	1.342636992	0.478119483	FALSE
GB11856	2497	1427	0.807210486	-0.057307022	FALSE
GB11857	18	7	1.362570079	0.498052571	FALSE
GB11858	699	422	0.728049457	-0.136468052	FALSE
GB11859	10	4	1.321928095	0.457410587	FALSE
GB11860	1315	658	0.99890331	0.134385802	FALSE
GB11861	381	188	1.019058336	0.154540827	FALSE
GB11862	17220	8587	1.003859046	0.139341538	FALSE
GB11863	408	213	0.937715722	0.073198213	FALSE
GB11864	930	623	0.577998553	-0.286518955	FALSE
GB11865	3244	1691	0.93989716	0.075379651	FALSE
GB11866	14	7	1	0.135482492	FALSE
GB11867	14	7	1	0.135482492	FALSE
GB11868	36	23	0.646363045	-0.218154463	FALSE
GB11869	6	1	2.584962501	1.720444992	TRUE
GB11870	2127	826	1.364606347	0.500088838	TRUE
GB11871	143	57	1.326981323	0.462463814	FALSE
GB11872	66	47	0.489805268	-0.374712241	FALSE
GB11873	1003	531	0.91753784	0.053020331	FALSE
GB11874	813	377	1.108690829	0.24417332	FALSE
GB11875	751	413	0.862671126	-0.001846382	FALSE
GB11876	16119	7016	1.200041591	0.335524083	FALSE
GB11877	388	296	0.390459477	-0.474058032	FALSE
GB11878	NA	NA	NA	NA	FALSE
GB11879	161	90	0.839063782	-0.025453727	FALSE
GB11880	26	13	1	0.135482492	FALSE
GB11881	126	77	0.710493383	-0.154024126	FALSE
GB11882	414	206	1.00698643	0.142468922	FALSE
GB11883	NA	NA	NA	NA	FALSE
GB11884	541	243	1.15467228	0.290154772	FALSE
GB11885	2977	1722	0.789774079	-0.074743429	FALSE

GB11886	1	2	-1	-1.864517508	TRUE
GB11887	310	192	0.691161905	-0.173355604	FALSE
GB11888	NA	NA	NA	NA	FALSE
GB11889	154	98	0.652076697	-0.212440812	FALSE
GB11890	NA	NA	NA	NA	FALSE
GB11891	406	233	0.801149773	-0.063367736	FALSE
GB11892	9379	6904	0.442001645	-0.422515864	FALSE
GB11893	285	173	0.720189881	-0.144327627	FALSE
GB11894	2	0	NA	NA	FALSE
GB11895	361	233	0.631668882	-0.232848626	FALSE
GB11896	1720	829	1.052964558	0.18844705	FALSE
GB11897	1349	643	1.068999706	0.204482197	FALSE
GB11898	1596	1060	0.590396387	-0.274121122	FALSE
GB11899	2124	1027	1.048347584	0.183830076	FALSE
GB11900	908	345	1.396095936	0.531578427	TRUE
GB11901	43	15	1.519374159	0.654856651	TRUE
GB11902	364	164	1.150242636	0.285725127	FALSE
GB11903	NA	NA	NA	NA	FALSE
GB11904	0	1	NA	NA	FALSE
GB11905	337	192	0.81164228	-0.052875228	FALSE
GB11906	1215	730	0.734987945	-0.129529564	FALSE
GB11907	1334	475	1.489759248	0.62524174	TRUE
GB11908	10	2	2.321928095	1.457410587	TRUE
GB11909	84	51	0.719892081	-0.144625428	FALSE
GB11910	218	130	0.745816512	-0.118700997	FALSE
GB11911	1727	859	1.007538046	0.143020538	FALSE
GB11912	30	18	0.736965594	-0.127551914	FALSE
GB11913	1280	666	0.942549728	0.078032219	FALSE
GB11914	2442	1565	0.641900543	-0.222616965	FALSE
GB11915	3850	2031	0.922668206	0.058150698	FALSE
GB11916	50	36	0.473931188	-0.39058632	FALSE
GB11917	NA	NA	NA	NA	FALSE
GB11918	0	1	NA	NA	FALSE
GB11919	709	346	1.03501359	0.170496081	FALSE
GB11920	5986	2843	1.07417818	0.209660671	FALSE
GB11921	466	295	0.659615	-0.204902508	FALSE
GB11922	NA	NA	NA	NA	FALSE
GB11923	5802	4127	0.491456857	-0.373060651	FALSE
GB11924	1583	932	0.764259395	-0.100258113	FALSE
GB11925	532	326	0.706554281	-0.157963227	FALSE
GB11926	2	1	1	0.135482492	FALSE
GB11927	1894	1054	0.845561464	-0.018956044	FALSE
GB11928	13074	7473	0.806941175	-0.057576333	FALSE
GB11929	893	580	0.622607275	-0.241910233	FALSE

GB11930	11	3	1.874469118	1.00995161	TRUE
GB11931	1	0	NA	NA	FALSE
GB11932	555	350	0.665132849	-0.199384659	FALSE
GB11933	3635	1969	0.884492253	0.019974745	FALSE
GB11934	1368	739	0.888421961	0.023904452	FALSE
GB11935	2078	1024	1.020979939	0.156462431	FALSE
GB11936	581	355	0.710719139	-0.153798369	FALSE
GB11937	NA	NA	NA	NA	FALSE
GB11938	152	82	0.890375509	0.025858	FALSE
GB11939	1	1	0	-0.864517508	TRUE
GB11940	NA	NA	NA	NA	FALSE
GB11941	897	473	0.923267802	0.058750293	FALSE
GB11942	252	96	1.392317423	0.527799914	TRUE
GB11943	170	77	1.142604395	0.278086887	FALSE
GB11944	1114	571	0.964186582	0.099669074	FALSE
GB11945	1071	569	0.912457922	0.047940414	FALSE
GB11946	5654	2965	0.931239778	0.06672227	FALSE
GB11947	5043	2733	0.883796765	0.019279257	FALSE
GB11948	463	200	1.211012193	0.346494685	FALSE
GB11949	79	43	0.877515993	0.012998485	FALSE
GB11950	4035	2439	0.726278915	-0.138238593	FALSE
GB11951	7	2	1.807354922	0.942837414	TRUE
GB11952	31	12	1.36923381	0.504716301	TRUE
GB11953	598	292	1.034177115	0.169659607	FALSE
GB11954	273	176	0.633325522	-0.231191986	FALSE
GB11955	1	1	0	-0.864517508	TRUE
GB11956	1106	605	0.870344338	0.00582683	FALSE
GB11957	38	23	0.724365557	-0.140151951	FALSE
GB11958	1007	540	0.899032371	0.034514863	FALSE
GB11959	7	4	0.807354922	-0.057162586	FALSE
GB11960	452	193	1.227721925	0.363204417	FALSE
GB11961	NA	NA	NA	NA	FALSE
GB11962	1155	471	1.294093887	0.429576378	FALSE
GB11963	397	292	0.443170638	-0.42134687	FALSE
GB11964	129	55	1.229867542	0.365350034	FALSE
GB11965	6	4	0.584962501	-0.279555008	FALSE
GB11966	633	380	0.736206081	-0.128311427	FALSE
GB11967	582	341	0.771247414	-0.093270094	FALSE
GB11968	321	167	0.942725195	0.078207686	FALSE
GB11969	20540	13092	0.649750674	-0.214766834	FALSE
GB11970	335	186	0.848858474	-0.015659034	FALSE
GB11971	289	169	0.774046246	-0.090471262	FALSE
GB11972	237	142	0.738996129	-0.125521379	FALSE
GB11973	89906	46029	0.965874299	0.101356791	FALSE

GB11974	114	82	0.47533801	-0.389179499	FALSE
GB11975	546	354	0.625151591	-0.239365917	FALSE
GB11976	725	393	0.883451683	0.018934174	FALSE
GB11977	271	115	1.23665899	0.372141482	FALSE
GB11978	5339	2682	0.993260312	0.128742803	FALSE
GB11979	1180	682	0.790943215	-0.073574293	FALSE
GB11980	516	350	0.560016144	-0.304501365	FALSE
GB11981	5831	3359	0.795711527	-0.068805981	FALSE
GB11982	220	149	0.562191193	-0.302326315	FALSE
GB11983	27499	18576	0.565939279	-0.29857823	FALSE
GB11984	507	368	0.462279981	-0.402237527	FALSE
GB11986	NA	NA	NA	NA	FALSE
GB11987	18	7	1.362570079	0.498052571	FALSE
GB11988	18	23	-0.353636955	-1.218154463	TRUE
GB11989	993	574	0.790742981	-0.073774527	FALSE
GB11990	1049	627	0.74247733	-0.122040179	FALSE
GB11991	154	41	1.909234536	1.044717028	TRUE
GB11992	885	507	0.803691708	-0.0608258	FALSE
GB11993	238	134	0.828728573	-0.035788935	FALSE
GB11994	460	232	0.987509056	0.122991547	FALSE
GB11995	446	310	0.524775495	-0.339742014	FALSE
GB11996	84	52	0.691877705	-0.172639804	FALSE
GB11997	NA	NA	NA	NA	FALSE
GB11998	388	218	0.831728517	-0.032788991	FALSE
GB11999	458	197	1.217151969	0.35263446	FALSE
GB12000	86	21	2.033947332	1.169429824	TRUE
GB12001	328	191	0.780123177	-0.084394332	FALSE
GB12002	NA	NA	NA	NA	FALSE
GB12003	80	30	1.415037499	0.550519991	TRUE
GB12004	2552	1182	1.110398294	0.245880785	FALSE
GB12005	95	73	0.380031049	-0.484486459	FALSE
GB12006	238	87	1.451874267	0.587356759	TRUE
GB12007	144	69	1.061400545	0.196883036	FALSE
GB12008	295	118	1.321928095	0.457410587	FALSE
GB12009	661	425	0.63718743	-0.227330078	FALSE
GB12010	330	188	0.811733363	-0.052784146	FALSE
GB12011	292	255	0.195471122	-0.669046386	TRUE
GB12012	539	316	0.770360715	-0.094156794	FALSE
GB12013	NA	NA	NA	NA	FALSE
GB12014	0	3	NA	NA	FALSE
GB12015	900	455	0.984058456	0.119540948	FALSE
GB12016	2336	1189	0.974291559	0.109774051	FALSE
GB12017	436	264	0.723790205	-0.140727303	FALSE
GB12018	1	0	NA	NA	FALSE

GB12019	725	477	0.603991729	-0.260525779	FALSE
GB12020	181	135	0.42303029	-0.441487218	FALSE
GB12021	377	198	0.929064093	0.064546585	FALSE
GB12022	208	77	1.433653177	0.569135669	TRUE
GB12023	1303	573	1.18523004	0.320712531	FALSE
GB12024	251	183	0.455843716	-0.408673793	FALSE
GB12025	676	318	1.087996481	0.223478973	FALSE
GB12026	468	249	0.910362788	0.045845279	FALSE
GB12027	171	98	0.803142671	-0.061374838	FALSE
GB12028	3	4	-0.415037499	-1.279555008	TRUE
GB12029	2179	1250	0.801738102	-0.062779407	FALSE
GB12030	1894	962	0.977327532	0.112810023	FALSE
GB12031	520	208	1.321928095	0.457410587	FALSE
GB12032	304	182	0.740132873	-0.124384635	FALSE
GB12033	1627	975	0.738740127	-0.125777381	FALSE
GB12034	546	269	1.021294778	0.15677727	FALSE
GB12035	1675	1113	0.589707503	-0.274810005	FALSE
GB12036	2681	1430	0.906756072	0.042238564	FALSE
GB12037	NA	NA	NA	NA	FALSE
GB12038	217	126	0.784271309	-0.080246199	FALSE
GB12039	821	458	0.842034624	-0.022482885	FALSE
GB12040	8	3	1.415037499	0.550519991	TRUE
GB12041	5	5	0	-0.864517508	TRUE
GB12042	1219	849	0.521861667	-0.342655841	FALSE
GB12043	682	343	0.991563163	0.127045655	FALSE
GB12044	1099	727	0.596164117	-0.268353391	FALSE
GB12045	16	13	0.299560282	-0.564957226	TRUE
GB12046	1122	634	0.82351793	-0.040999578	FALSE
GB12047	1201	601	0.998799255	0.134281747	FALSE
GB12048	705	411	0.778484864	-0.086032645	FALSE
GB12049	2828	1421	0.992875566	0.128358057	FALSE
GB12050	1146	663	0.789526269	-0.07499124	FALSE
GB12051	3424	1899	0.850442796	-0.014074712	FALSE
GB12052	970	394	1.299789118	0.435271609	FALSE
GB12053	82	67	0.291462814	-0.573054694	TRUE
GB12054	488	341	0.517109409	-0.3474081	FALSE
GB12055	523	308	0.763880596	-0.100636913	FALSE
GB12056	NA	NA	NA	NA	FALSE
GB12057	358	226	0.663636815	-0.200880693	FALSE
GB12058	25	10	1.321928095	0.457410587	FALSE
GB12059	693	443	0.645548654	-0.218968855	FALSE
GB12060	1458	839	0.797248004	-0.067269504	FALSE
GB12061	2188	1342	0.705228067	-0.159289442	FALSE
GB12062	35	31	0.175086707	-0.689430802	TRUE

GB12063	239	130	0.878498995	0.013981487	FALSE
GB12064	11	7	0.652076697	-0.212440812	FALSE
GB12065	6	0	NA	NA	FALSE
GB12066	110	66	0.736965594	-0.127551914	FALSE
GB12067	1284	807	0.670004624	-0.194512884	FALSE
GB12068	90	51	0.819427754	-0.045089754	FALSE
GB12069	857	490	0.806513455	-0.058004053	FALSE
GB12070	1	0	NA	NA	FALSE
GB12071	211	135	0.644283592	-0.220233917	FALSE
GB12072	18	7	1.362570079	0.498052571	FALSE
GB12073	351	156	1.169925001	0.305407493	FALSE
GB12074	532	369	0.527805429	-0.336712079	FALSE
GB12075	62	27	1.199308808	0.3347913	FALSE
GB12076	53	17	1.640457613	0.775940105	TRUE
GB12077	326	219	0.573941095	-0.290576414	FALSE
GB12078	12	2	2.584962501	1.720444992	TRUE
GB12079	1353	670	1.013928839	0.14941133	FALSE
GB12080	112	63	0.830074999	-0.03444251	FALSE
GB12081	1647	903	0.867042662	0.002525154	FALSE
GB12082	393	202	0.96017402	0.095656511	FALSE
GB12083	1561	829	0.91302653	0.048509022	FALSE
GB12084	82	69	0.249027548	-0.61548996	TRUE
GB12085	6666	3284	1.02136719	0.156849682	FALSE
GB12086	564	284	0.989804233	0.125286725	FALSE
GB12087	617	370	0.737745219	-0.12677229	FALSE
GB12088	NA	NA	NA	NA	FALSE
GB12089	8617	6091	0.500506579	-0.364010929	FALSE
GB12090	3049	2013	0.598988978	-0.26552853	FALSE
GB12091	55	30	0.874469118	0.00995161	FALSE
GB12092	630	326	0.950479864	0.085962356	FALSE
GB12093	314	136	1.207157908	0.342640399	FALSE
GB12094	6222	3474	0.840780641	-0.023736868	FALSE
GB12095	52	17	1.612976877	0.748459369	TRUE
GB12096	1	0	NA	NA	FALSE
GB12097	294	141	1.060120992	0.195603484	FALSE
GB12098	3524	2157	0.708187748	-0.156329761	FALSE
GB12099	4129	2158	0.936097554	0.071580045	FALSE
GB12100	826	534	0.62930204	-0.235215469	FALSE
GB12101	454	356	0.350815056	-0.513702452	TRUE
GB12102	18	15	0.263034406	-0.601483102	TRUE
GB12104	357	170	1.070389328	0.20587182	FALSE
GB12105	816	427	0.934333082	0.069815574	FALSE
GB12106	NA	NA	NA	NA	FALSE
GB12107	3066	1628	0.913256997	0.048739489	FALSE

GB12108	17	9	0.91753784	0.053020331	FALSE
GB12109	969	523	0.889685719	0.025168211	FALSE
GB12110	74	38	0.961525852	0.097008344	FALSE
GB12111	846	481	0.814620769	-0.049896739	FALSE
GB12112	947	513	0.8844056	0.019888092	FALSE
GB12113	10519	6964	0.595009452	-0.269508057	FALSE
GB12114	332	140	1.245756414	0.381238906	FALSE
GB12115	4	0	NA	NA	FALSE
GB12116	1913	989	0.951794448	0.087276939	FALSE
GB12117	179	120	0.576925182	-0.287592327	FALSE
GB12118	834	546	0.611146433	-0.253371076	FALSE
GB12119	254	155	0.712560281	-0.151957227	FALSE
GB12120	366	228	0.682809824	-0.181707684	FALSE
GB12121	14420	6170	1.22472877	0.360211262	FALSE
GB12122	1848	1238	0.577953442	-0.286564066	FALSE
GB12123	4322	3169	0.447671412	-0.416846096	FALSE
GB12124	699	397	0.816153448	-0.04836406	FALSE
GB12125	8297	4422	0.907890761	0.043373253	FALSE
GB12126	625	358	0.803896602	-0.060620906	FALSE
GB12127	1220	652	0.903937278	0.03941977	FALSE
GB12128	119	59	1.012174714	0.147657206	FALSE
GB12129	1114	549	1.020871178	0.15635367	FALSE
GB12130	3	2	0.584962501	-0.279555008	FALSE
GB12131	5	0	NA	NA	FALSE
GB12132	3633	1978	0.87711894	0.012601431	FALSE
GB12133	589	381	0.628476636	-0.236040872	FALSE
GB12134	400	207	0.950369232	0.085851724	FALSE
GB12135	8971	5011	0.840170274	-0.024347234	FALSE
GB12136	1	1	0	-0.864517508	TRUE
GB12137	696	483	0.527064117	-0.337453391	FALSE
GB12138	223	111	1.006484034	0.141966525	FALSE
GB12139	2697	1491	0.855075264	-0.009442244	FALSE
GB12140	24203	15713	0.62322723	-0.241290278	FALSE
GB12141	NA	NA	NA	NA	FALSE
GB12142	1614	810	0.994646766	0.130129257	FALSE
GB12143	676	468	0.530514717	-0.334002792	FALSE
GB12144	1079	527	1.033819998	0.16930249	FALSE
GB12145	940	467	1.009238207	0.144720699	FALSE
GB12146	1129	651	0.794316038	-0.070201471	FALSE
GB12147	617	339	0.863985216	-0.000532292	FALSE
GB12148	2133	1068	0.997972319	0.13345481	FALSE
GB12149	210	106	0.986325063	0.121807555	FALSE
GB12150	578	307	0.912830837	0.048313329	FALSE
GB12151	17434	9768	0.835768509	-0.028748999	FALSE

GB12152	1	0	NA	NA	FALSE
GB12153	29	5	2.5360529	1.671535392	TRUE
GB12154	NA	NA	NA	NA	FALSE
GB12155	585	338	0.791413378	-0.07310413	FALSE
GB12156	2304	1050	1.133751389	0.269233881	FALSE
GB12157	5	0	NA	NA	FALSE
GB12158	20164	9154	1.139307662	0.274790154	FALSE
GB12159	1913	984	0.959106653	0.094589145	FALSE
GB12160	1142	886	0.366184047	-0.498333462	FALSE
GB12161	94	53	0.826668397	-0.037849111	FALSE
GB12162	238	148	0.685364398	-0.179153111	FALSE
GB12163	19	10	0.925999419	0.06148191	FALSE
GB12164	972	800	0.280956314	-0.583561194	TRUE
GB12165	650	365	0.832543254	-0.031974254	FALSE
GB12166	425	178	1.2555856	0.391068092	FALSE
GB12167	102	55	0.891065628	0.02654812	FALSE
GB12168	256	133	0.944717564	0.080200056	FALSE
GB12169	40	26	0.621488377	-0.243029132	FALSE
GB12170	623	381	0.709441166	-0.155076343	FALSE
GB12171	1767	901	0.971703029	0.10718552	FALSE
GB12172	263	165	0.672596775	-0.191920733	FALSE
GB12173	1394	860	0.696821996	-0.167695512	FALSE
GB12174	262	171	0.615570487	-0.248947022	FALSE
GB12175	1173	711	0.722281548	-0.14223596	FALSE
GB12176	298	154	0.95238198	0.087864471	FALSE
GB12177	42	19	1.144389909	0.279872401	FALSE
GB12178	292	240	0.282933963	-0.581583545	TRUE
GB12179	4233	1896	1.158721524	0.294204016	FALSE
GB12180	2256	1462	0.625823756	-0.238693752	FALSE
GB12181	2111	1206	0.807696671	-0.056820837	FALSE
GB12182	7505	4235	0.825490102	-0.039027406	FALSE
GB12183	142	78	0.864344901	-0.000172608	FALSE
GB12184	32324	15705	1.041381795	0.176864287	FALSE
GB12185	6060	2613	1.213610669	0.349093161	FALSE
GB12186	1115	685	0.702867817	-0.161649691	FALSE
GB12187	192	61	1.654225163	0.789707655	TRUE
GB12188	101	54	0.903323981	0.038806472	FALSE
GB12189	1128	698	0.692468126	-0.172049382	FALSE
GB12190	6041	1853	1.704924505	0.840406997	TRUE
GB12191	12	6	1	0.135482492	FALSE
GB12192	2	5	-1.321928095	-2.186445603	TRUE
GB12193	910	487	0.901944773	0.037427265	FALSE
GB12194	11	25	-1.184424571	-2.048942079	TRUE
GB12195	2	1	1	0.135482492	FALSE

GB12196	381	220	0.792287474	-0.072230034	FALSE
GB12197	1	0	NA	NA	FALSE
GB12198	50963	47179	0.111305366	-0.753212142	TRUE
GB12199	124	45	1.462343214	0.597825706	TRUE
GB12200	4	4	0	-0.864517508	TRUE
GB12201	2052	1273	0.688798312	-0.175719197	FALSE
GB12202	650	306	1.086908065	0.222390557	FALSE
GB12203	145	80	0.857980995	-0.006536513	FALSE
GB12204	NA	NA	NA	NA	FALSE
GB12205	5001	3604	0.472617594	-0.391899914	FALSE
GB12206	15	10	0.584962501	-0.279555008	FALSE
GB12207	2041	746	1.452028647	0.587511138	TRUE
GB12208	0	1	NA	NA	FALSE
GB12209	1350	796	0.762119071	-0.102398437	FALSE
GB12210	146	106	0.461904104	-0.402613404	FALSE
GB12211	19	9	1.078002512	0.213485004	FALSE
GB12212	NA	NA	NA	NA	FALSE
GB12213	1656	747	1.148522525	0.284005016	FALSE
GB12214	7901	3606	1.131635865	0.267118356	FALSE
GB12215	5927	3783	0.647771281	-0.216746227	FALSE
GB12216	NA	NA	NA	NA	FALSE
GB12217	1398	817	0.774956377	-0.089561131	FALSE
GB12218	670	405	0.726239188	-0.138278321	FALSE
GB12219	1267	677	0.904188786	0.039671277	FALSE
GB12220	812	474	0.776592668	-0.08792484	FALSE
GB12221	261	146	0.838081438	-0.026436071	FALSE
GB12222	407	238	0.774067221	-0.090450287	FALSE
GB12223	358	189	0.921573353	0.057055845	FALSE
GB12224	1267	747	0.762236376	-0.102281132	FALSE
GB12225	94	56	0.74723393	-0.117283579	FALSE
GB12226	3433	1732	0.987030927	0.122513419	FALSE
GB12227	452	215	1.071986113	0.207468604	FALSE
GB12228	9	2	2.169925001	1.305407493	TRUE
GB12229	196	113	0.794530882	-0.069986627	FALSE
GB12230	22369	14617	0.61385352	-0.250663988	FALSE
GB12231	12648	7687	0.718416699	-0.146100809	FALSE
GB12232	10	2	2.321928095	1.457410587	TRUE
GB12233	1	0	NA	NA	FALSE
GB12234	2	1	1	0.135482492	FALSE
GB12235	260	179	0.538552036	-0.325965473	FALSE
GB12236	98	41	1.257157839	0.392640331	FALSE
GB12237	1015	676	0.586384576	-0.278132933	FALSE
GB12238	983	593	0.729159312	-0.135358197	FALSE
GB12239	12013	4977	1.271248187	0.406730679	FALSE

GB12240	6	3	1	0.135482492	FALSE
GB12241	450	233	0.949595047	0.085077538	FALSE
GB12242	835	498	0.745630455	-0.118887053	FALSE
GB12243	1175	639	0.878772921	0.014255412	FALSE
GB12244	1431	705	1.021328509	0.156811001	FALSE
GB12245	43	13	1.725825037	0.861307528	TRUE
GB12246	1625	831	0.967519336	0.103001828	FALSE
GB12247	1253	517	1.277150229	0.412632721	FALSE
GB12248	1343	653	1.040304408	0.1757869	FALSE
GB12249	615	309	0.992979572	0.128462064	FALSE
GB12250	134	86	0.639824436	-0.224693073	FALSE
GB12251	472	269	0.811180687	-0.053336822	FALSE
GB12252	491	218	1.17139489	0.306877381	FALSE
GB12253	45	14	1.684498174	0.819980666	TRUE
GB12254	2	9	-2.169925001	-3.03444251	TRUE
GB12255	2	0	NA	NA	FALSE
GB12256	55	18	1.611434712	0.746917204	TRUE
GB12257	68	35	0.958179824	0.093662316	FALSE
GB12258	0	1	NA	NA	FALSE
GB12259	10324	5013	1.042255907	0.177738399	FALSE
GB12260	NA	NA	NA	NA	FALSE
GB12261	493	331	0.57475643	-0.289761079	FALSE
GB12262	1033	498	1.052622607	0.188105098	FALSE
GB12263	260	142	0.872620694	0.008103185	FALSE
GB12264	613	330	0.893421049	0.028903541	FALSE
GB12265	283	155	0.868533838	0.004016329	FALSE
GB12266	85	44	0.949959318	0.085441809	FALSE
GB12267	442	324	0.448052557	-0.416464952	FALSE
GB12268	14	9	0.637429921	-0.227087588	FALSE
GB12269	415	220	0.915607813	0.051090304	FALSE
GB12270	1099	726	0.598149933	-0.266367575	FALSE
GB12271	170	97	0.809478094	-0.055039414	FALSE
GB12272	990	655	0.595933619	-0.26858389	FALSE
GB12273	189	84	1.169925001	0.305407493	FALSE
GB12274	304	250	0.282143229	-0.58237428	TRUE
GB12275	NA	NA	NA	NA	FALSE
GB12276	267	167	0.676991639	-0.187525869	FALSE
GB12277	708	405	0.805827452	-0.058690056	FALSE
GB12278	1	1	0	-0.864517508	TRUE
GB12279	18	14	0.362570079	-0.501947429	TRUE
GB12280	310	134	1.210035215	0.345517706	FALSE
GB12281	716	407	0.814930793	-0.049586715	FALSE
GB12282	NA	NA	NA	NA	FALSE
GB12283	14774	9096	0.699756323	-0.164761185	FALSE

GB12284	12142	8264	0.555093919	-0.309423589	FALSE
GB12285	46	22	1.064130337	0.199612829	FALSE
GB12286	457	176	1.376618736	0.512101228	TRUE
GB12287	409	198	1.046600413	0.182082905	FALSE
GB12288	258	161	0.680310377	-0.184207131	FALSE
GB12289	3659	1952	0.906496363	0.041978854	FALSE
GB12290	20	14	0.514573173	-0.349944335	FALSE
GB12291	1528	731	1.063701232	0.199183724	FALSE
GB12292	3	4	-0.415037499	-1.279555008	TRUE
GB12293	39188	15154	1.370713292	0.506195784	TRUE
GB12294	NA	NA	NA	NA	FALSE
GB12295	703	481	0.547487795	-0.317029713	FALSE
GB12296	366	259	0.498891551	-0.365625958	FALSE
GB12297	482	272	0.825426495	-0.039091013	FALSE
GB12298	701	347	1.014478781	0.149961273	FALSE
GB12299	919	355	1.372245837	0.507728329	TRUE
GB12300	5	3	0.736965594	-0.127551914	FALSE
GB12301	46	21	1.131244533	0.266727025	FALSE
GB12302	445	146	1.607836967	0.743319459	TRUE
GB12303	1474	768	0.940558308	0.0760408	FALSE
GB12304	NA	NA	NA	NA	FALSE
GB12305	462	256	0.851749041	-0.012768467	FALSE
GB12306	NA	NA	NA	NA	FALSE
GB12307	1310	676	0.95447166	0.089954152	FALSE
GB12308	4272	2306	0.889519134	0.025001626	FALSE
GB12309	NA	NA	NA	NA	FALSE
GB12310	NA	NA	NA	NA	FALSE
GB12311	682	348	0.970684433	0.106166925	FALSE
GB12312	7466	4485	0.735227523	-0.129289985	FALSE
GB12313	72	33	1.125530882	0.261013374	FALSE
GB12314	5110	2662	0.94081272	0.076295212	FALSE
GB12315	51	32	0.672425342	-0.192092166	FALSE
GB12316	321	187	0.779535027	-0.084982481	FALSE
GB12317	1393	807	0.787554679	-0.076962829	FALSE
GB12318	3334	1438	1.213190429	0.34867292	FALSE
GB12319	83	10	3.053111336	2.188593828	TRUE
GB12320	46	24	0.938599455	0.074081947	FALSE
GB12321	691	366	0.916842062	0.052324554	FALSE
GB12322	480	248	0.952694285	0.088176777	FALSE
GB12323	407	295	0.46431384	-0.400203668	FALSE
GB12324	934	536	0.801189549	-0.063327959	FALSE
GB12325	357	238	0.584962501	-0.279555008	FALSE
GB12326	10848	5357	1.017931882	0.153414374	FALSE
GB12327	105069	63136	0.734802307	-0.129715201	FALSE

GB12328	545	300	0.861293729	-0.003223779	FALSE
GB12329	7	1	2.807354922	1.942837414	TRUE
GB12330	2591	1165	1.153179061	0.288661552	FALSE
GB12331	207	80	1.371558863	0.507041354	TRUE
GB12332	1	0	NA	NA	FALSE
GB12333	NA	NA	NA	NA	FALSE
GB12334	563	302	0.898586373	0.034068864	FALSE
GB12335	809	476	0.765178129	-0.099339379	FALSE
GB12336	1	0	NA	NA	FALSE
GB12337	2	1	1	0.135482492	FALSE
GB12338	36029	23104	0.641015962	-0.223501546	FALSE
GB12339	867	383	1.178687601	0.314170093	FALSE
GB12340	NA	NA	NA	NA	FALSE
GB12341	1690	1010	0.742667954	-0.121849555	FALSE
GB12342	157	72	1.124695747	0.260178239	FALSE
GB12343	758	536	0.499964848	-0.364552661	FALSE
GB12344	372	239	0.638292003	-0.226225505	FALSE
GB12345	808	402	1.007159792	0.142642283	FALSE
GB12346	528	240	1.137503524	0.272986015	FALSE
GB12347	1233	737	0.742436275	-0.122081233	FALSE
GB12348	19508	10219	0.932811852	0.068294344	FALSE
GB12349	729	337	1.113170223	0.248652715	FALSE
GB12350	394	236	0.73940877	-0.125108738	FALSE
GB12351	488	318	0.617854382	-0.246663126	FALSE
GB12352	1	2	-1	-1.864517508	TRUE
GB12353	388	144	1.429987841	0.565470332	TRUE
GB12354	1370	709	0.950318361	0.085800852	FALSE
GB12355	142	64	1.14974712	0.285229611	FALSE
GB12356	946	598	0.661694699	-0.202822809	FALSE
GB12357	373	271	0.460882779	-0.403634729	FALSE
GB12358	489	358	0.449874878	-0.414642631	FALSE
GB12359	2530	1262	1.003425475	0.138907966	FALSE
GB12360	2046	935	1.129767875	0.265250367	FALSE
GB12361	12988	9393	0.467521375	-0.396996133	FALSE
GB12362	1	2	-1	-1.864517508	TRUE
GB12363	288	128	1.169925001	0.305407493	FALSE
GB12364	12876	7248	0.829029621	-0.035487887	FALSE
GB12365	101	47	1.103622631	0.239105123	FALSE
GB12366	807	437	0.884935394	0.020417885	FALSE
GB12367	429	252	0.767553914	-0.096963594	FALSE
GB12368	31	9	1.784271309	0.919753801	TRUE
GB12369	13	2	2.700439718	1.83592221	TRUE
GB12370	145	84	0.787591667	-0.076925841	FALSE
GB12371	17256	9081	0.926175001	0.061657492	FALSE

GB12372	3234	1967	0.717322721	-0.147194787	FALSE
GB12373	447	204	1.131705679	0.267188171	FALSE
GB12374	829	377	1.136807578	0.27229007	FALSE
GB12375	3252	1562	1.057932804	0.193415296	FALSE
GB12376	NA	NA	NA	NA	FALSE
GB12377	201	145	0.471142601	-0.393374907	FALSE
GB12378	1411	613	1.202759009	0.338241501	FALSE
GB12379	24	16	0.584962501	-0.279555008	FALSE
GB12380	4	6	-0.584962501	-1.449480009	TRUE
GB12381	3580	2464	0.538957331	-0.325560177	FALSE
GB12382	2039	1171	0.8001207	-0.064396809	FALSE
GB12383	10299	6864	0.585382805	-0.279134703	FALSE
GB12384	1795	1075	0.739647184	-0.124870324	FALSE
GB12385	862	563	0.614552947	-0.249964561	FALSE
GB12386	178	144	0.30580843	-0.558709079	TRUE
GB12387	350	160	1.129283017	0.264765509	FALSE
GB12388	392	248	0.660513534	-0.204003975	FALSE
GB12389	706	508	0.474839686	-0.389677822	FALSE
GB12390	2291	1399	0.711581496	-0.152936012	FALSE
GB12391	389	211	0.882527156	0.018009648	FALSE
GB12392	553	266	1.055853235	0.191335726	FALSE
GB12393	NA	NA	NA	NA	FALSE
GB12394	469	210	1.159198595	0.294681087	FALSE
GB12395	1928	999	0.948548468	0.08403096	FALSE
GB12396	736	362	1.023716069	0.159198561	FALSE
GB12397	401	276	0.53893397	-0.325583539	FALSE
GB12398	299	168	0.831684251	-0.032833257	FALSE
GB12399	2048	1446	0.502148163	-0.362369345	FALSE
GB12400	2701	1520	0.829422316	-0.035095192	FALSE
GB12401	1	7	-2.807354922	-3.67187243	TRUE
GB12402	1670	1060	0.655783838	-0.20873367	FALSE
GB12403	NA	NA	NA	NA	FALSE
GB12404	527	305	0.788993719	-0.075523789	FALSE
GB12405	11	7	0.652076697	-0.212440812	FALSE
GB12406	274	133	1.042749647	0.178232139	FALSE
GB12407	400	259	0.627047902	-0.237469606	FALSE
GB12408	225	102	1.141355849	0.276838341	FALSE
GB12409	598	325	0.879705766	0.015188258	FALSE
GB12410	NA	NA	NA	NA	FALSE
GB12411	42	11	1.932885804	1.068368296	TRUE
GB12412	1896	1083	0.807925721	-0.056591787	FALSE
GB12413	NA	NA	NA	NA	FALSE
GB12414	847	544	0.638755318	-0.22576219	FALSE
GB12415	1	2	-1	-1.864517508	TRUE

GB12416	152	65	1.2255597	0.361042192	FALSE
GB12417	2781	1588	0.808392832	-0.056124676	FALSE
GB12418	2411	1502	0.682746838	-0.18177067	FALSE
GB12419	844	396	1.091742569	0.22722506	FALSE
GB12420	12514	8470	0.563109134	-0.301408374	FALSE
GB12421	408	177	1.204819792	0.340302284	FALSE
GB12422	613	296	1.050289898	0.18577239	FALSE
GB12423	1954	678	1.527073289	0.662555781	TRUE
GB12424	14	16	-0.192645078	-1.057162586	TRUE
GB12425	3000	1795	0.740978657	-0.123538852	FALSE
GB12426	191	120	0.670538232	-0.193979276	FALSE
GB12427	161	62	1.376720568	0.512203059	TRUE
GB12428	82	39	1.072149786	0.207632277	FALSE
GB12429	1	1	0	-0.864517508	TRUE
GB12430	503	312	0.689012371	-0.175505137	FALSE
GB12431	1260	455	1.469485283	0.604967775	TRUE
GB12432	1067	490	1.122706522	0.258189013	FALSE
GB12433	1076	573	0.909071034	0.044553525	FALSE
GB12434	55	24	1.196397213	0.331879704	FALSE
GB12435	294	220	0.418312631	-0.446204877	FALSE
GB12436	5440	2717	1.001592086	0.137074578	FALSE
GB12437	5	5	0	-0.864517508	TRUE
GB12438	100	57	0.810966176	-0.053551333	FALSE
GB12439	1927	1391	0.470234152	-0.394283357	FALSE
GB12440	207	112	0.886132035	0.021614527	FALSE
GB12441	19959	13423	0.572332294	-0.292185214	FALSE
GB12442	168	79	1.088536675	0.224019166	FALSE
GB12443	39	26	0.584962501	-0.279555008	FALSE
GB12444	107	67	0.675377796	-0.189139712	FALSE
GB12445	9	4	1.169925001	0.305407493	FALSE
GB12446	521	298	0.805971042	-0.058546467	FALSE
GB12447	147	65	1.177304532	0.312787023	FALSE
GB12448	4	3	0.415037499	-0.449480009	FALSE
GB12449	1	2	-1	-1.864517508	TRUE
GB12450	276	140	0.97924144	0.114723932	FALSE
GB12451	180	123	0.549338591	-0.315178917	FALSE
GB12452	8	3	1.415037499	0.550519991	TRUE
GB12453	12	6	1	0.135482492	FALSE
GB12454	507	215	1.237649087	0.373131579	FALSE
GB12455	894	525	0.767957409	-0.0965601	FALSE
GB12456	8	3	1.415037499	0.550519991	TRUE
GB12457	134	57	1.233199176	0.368681668	FALSE
GB12458	2	0	NA	NA	FALSE
GB12459	888	697	0.34940102	-0.515116488	TRUE

GB12460	12	12	0	-0.864517508	TRUE
GB12461	57	52	0.132450296	-0.732067212	TRUE
GB12462	495	220	1.169925001	0.305407493	FALSE
GB12463	957	458	1.063171326	0.198653818	FALSE
GB12464	243	85	1.515421567	0.650904059	TRUE
GB12465	87	40	1.121015401	0.256497893	FALSE
GB12466	770	505	0.608575058	-0.25594245	FALSE
GB12467	9	7	0.362570079	-0.501947429	TRUE
GB12468	2094	1036	1.015237439	0.150719931	FALSE
GB12469	634	312	1.022936811	0.158419303	FALSE
GB12470	4177	2186	0.934173742	0.069656234	FALSE
GB12471	609	410	0.570818318	-0.29369919	FALSE
GB12472	553	337	0.714530889	-0.149986619	FALSE
GB12473	0	1	NA	NA	FALSE
GB12474	409	252	0.698677109	-0.165840399	FALSE
GB12475	4716	3155	0.579923713	-0.284593795	FALSE
GB12476	102	71	0.522678222	-0.341839286	FALSE
GB12477	NA	NA	NA	NA	FALSE
GB12478	247	132	0.903973112	0.039455604	FALSE
GB12479	256	149	0.78083148	-0.083686029	FALSE
GB12480	85	27	1.654503434	0.789985926	TRUE
GB12481	625	333	0.908334012	0.043816504	FALSE
GB12482	209	113	0.88718017	0.022662661	FALSE
GB12483	413	205	1.010517872	0.146000364	FALSE
GB12484	65	40	0.700439718	-0.16407779	FALSE
GB12485	186	129	0.527931556	-0.336585953	FALSE
GB12486	1038	512	1.019590728	0.15507322	FALSE
GB12487	163	132	0.304334035	-0.560183473	TRUE
GB12488	33998	14940	1.186269731	0.321752223	FALSE
GB12489	401	235	0.77094148	-0.093576028	FALSE
GB12490	228	100	1.189033824	0.324516316	FALSE
GB12491	361	289	0.320929344	-0.543588164	TRUE
GB12492	278	161	0.788024195	-0.076493314	FALSE
GB12493	1099	659	0.737841016	-0.126676492	FALSE
GB12494	368	226	0.703382994	-0.161134515	FALSE
GB12495	4451	2138	1.057867648	0.193350139	FALSE
GB12496	984	537	0.873736227	0.009218719	FALSE
GB12497	949	520	0.867896464	0.003378956	FALSE
GB12498	646	335	0.947373069	0.082855561	FALSE
GB12499	997	520	0.939081881	0.074564373	FALSE
GB12500	32	10	1.678071905	0.813554397	TRUE
GB12501	1120	648	0.789433014	-0.075084494	FALSE
GB12502	58	27	1.103093493	0.238575985	FALSE
GB12503	73	48	0.604862058	-0.25965545	FALSE

GB12504	1647	906	0.8622576	-0.002259909	FALSE
GB12505	8	1	3	2.135482492	TRUE
GB12506	11998	6990	0.779429576	-0.085087932	FALSE
GB12507	0	4	NA	NA	FALSE
GB12508	34492	22397	0.622956284	-0.241561224	FALSE
GB12509	421	202	1.05946494	0.194947432	FALSE
GB12510	2826	1817	0.637203046	-0.227314462	FALSE
GB12511	638	318	1.004529658	0.14001215	FALSE
GB12512	72	50	0.526068812	-0.338448697	FALSE
GB12513	2847	1632	0.802801436	-0.061716073	FALSE
GB12514	121	36	1.748938236	0.884420728	TRUE
GB12515	511	342	0.579326966	-0.285190542	FALSE
GB12516	75	63	0.251538767	-0.612978741	TRUE
GB12517	583	414	0.493865116	-0.370652393	FALSE
GB12518	128	40	1.678071905	0.813554397	TRUE
GB12519	5	13	-1.378511623	-2.243029132	TRUE
GB12520	738	430	0.779284156	-0.085233352	FALSE
GB12521	267	150	0.831877241	-0.032640267	FALSE
GB12522	8	4	1	0.135482492	FALSE
GB12523	280	117	1.258918297	0.394400789	FALSE
GB12524	0	1	NA	NA	FALSE
GB12525	4650	2475	0.909802191	0.045284683	FALSE
GB12526	5383	3079	0.805948558	-0.05856895	FALSE
GB12527	NA	NA	NA	NA	FALSE
GB12528	2680	2068	0.373996815	-0.490520693	FALSE
GB12529	832	465	0.839352812	-0.025164696	FALSE
GB12530	NA	NA	NA	NA	FALSE
GB12531	289	166	0.799886251	-0.064631257	FALSE
GB12532	2565	1286	0.996068183	0.131550675	FALSE
GB12533	629	352	0.837484588	-0.02703292	FALSE
GB12534	707	374	0.918671945	0.054154437	FALSE
GB12535	12	4	1.584962501	0.720444992	TRUE
GB12536	50	22	1.184424571	0.319907063	FALSE
GB12537	13	7	0.893084796	0.028567288	FALSE
GB12538	8124	4034	1.009979156	0.145461647	FALSE
GB12539	362	236	0.617202838	-0.247314671	FALSE
GB12540	1019	433	1.234715121	0.370197613	FALSE
GB12541	2015	937	1.104658886	0.240141377	FALSE
GB12542	1985	1077	0.882120757	0.017603249	FALSE
GB12543	3	3	0	-0.864517508	TRUE
GB12544	104	59	0.817796669	-0.04672084	FALSE
GB12545	1668	889	0.907863965	0.043346456	FALSE
GB12546	574	253	1.181913352	0.317395844	FALSE
GB12547	920	511	0.84831057	-0.016206938	FALSE

GB12548	298	205	0.539688421	-0.324829087	FALSE
GB12549	1173	432	1.441099796	0.576582288	TRUE
GB12550	0	1	NA	NA	FALSE
GB12551	1	0	NA	NA	FALSE
GB12552	440	299	0.557358039	-0.307159469	FALSE
GB12553	556	276	1.010416616	0.145899108	FALSE
GB12554	3269	1711	0.934009617	0.069492109	FALSE
GB12555	74	53	0.481532911	-0.382984597	FALSE
GB12556	439	251	0.806533576	-0.057983933	FALSE
GB12557	3335	2052	0.70065603	-0.163861478	FALSE
GB12558	163	68	1.261265313	0.396747805	FALSE
GB12559	179	119	0.588998014	-0.275519494	FALSE
GB12560	16	16	0	-0.864517508	TRUE
GB12561	4782	2514	0.92762948	0.063111972	FALSE
GB12562	12869	7583	0.763059324	-0.101458185	FALSE
GB12563	20	28	-0.485426827	-1.349944335	TRUE
GB12564	223	98	1.186190056	0.321672547	FALSE
GB12565	1240	717	0.790295097	-0.074222412	FALSE
GB12566	3136	1661	0.916873486	0.052355978	FALSE
GB12567	15157	12792	0.244742388	-0.619775121	TRUE
GB12568	772	484	0.6735938	-0.190923708	FALSE
GB12569	1025	506	1.01841462	0.153897111	FALSE
GB12570	1672	730	1.195606478	0.33108897	FALSE
GB12571	54	13	2.054447784	1.189930276	TRUE
GB12572	NA	NA	NA	NA	FALSE
GB12573	9612	6394	0.588117904	-0.276399604	FALSE
GB12574	269	181	0.571616475	-0.292901033	FALSE
GB12575	10809	4809	1.168424226	0.303906718	FALSE
GB12576	1469	792	0.89126206	0.026744552	FALSE
GB12577	237	133	0.833460813	-0.031056695	FALSE
GB12578	971	570	0.768509376	-0.096008132	FALSE
GB12579	331	138	1.26216295	0.397645442	FALSE
GB12580	54	36	0.584962501	-0.279555008	FALSE
GB12581	11	8	0.459431619	-0.40508589	FALSE
GB12582	449	220	1.029211921	0.164694413	FALSE
GB12583	7156	3613	0.985956133	0.121438624	FALSE
GB12584	1	1	0	-0.864517508	TRUE
GB12585	1899	1035	0.875609138	0.011091629	FALSE
GB12586	11754	9605	0.291294284	-0.573223224	TRUE
GB12587	273	136	1.0052943	0.140776791	FALSE
GB12588	19	11	0.788495895	-0.076021614	FALSE
GB12589	337	183	0.880904943	0.016387435	FALSE
GB12590	566	375	0.593911457	-0.270606051	FALSE
GB12591	797	437	0.866946444	0.002428936	FALSE

GB12592	931	587	0.665420664	-0.199096844	FALSE
GB12593	555	271	1.03419492	0.169677412	FALSE
GB12594	443	309	0.519699861	-0.344817648	FALSE
GB12595	195	119	0.71251255	-0.152004958	FALSE
GB12596	172	124	0.472068444	-0.392449064	FALSE
GB12597	28	22	0.347923303	-0.516594205	TRUE
GB12598	19	8	1.247927513	0.383410005	FALSE
GB12599	481	286	0.750021747	-0.114495761	FALSE
GB12600	8026	3947	1.023924637	0.159407129	FALSE
GB12601	468	279	0.746243408	-0.118274101	FALSE
GB12602	568	252	1.172467196	0.307949688	FALSE
GB12603	295	227	0.378022657	-0.486494851	FALSE
GB12604	234	125	0.904580435	0.040062927	FALSE
GB12605	20163	9894	1.027084505	0.162566997	FALSE
GB12606	NA	NA	NA	NA	FALSE
GB12607	1452	452	1.683646776	0.819129267	TRUE
GB12608	20	10	1	0.135482492	FALSE
GB12609	51	22	1.212993723	0.348476215	FALSE
GB12610	NA	NA	NA	NA	FALSE
GB12611	879	536	0.713630165	-0.150887344	FALSE
GB12612	5	1	2.321928095	1.457410587	TRUE
GB12613	1590	726	1.130985312	0.266467804	FALSE
GB12614	6167	2579	1.257757073	0.393239565	FALSE
GB12615	NA	NA	NA	NA	FALSE
GB12616	11	5	1.137503524	0.272986015	FALSE
GB12617	535	295	0.858823937	-0.005693571	FALSE
GB12618	105	88	0.254813899	-0.609703609	TRUE
GB12619	758	378	1.003811614	0.139294106	FALSE
GB12620	2329	1205	0.950677493	0.086159985	FALSE
GB12621	396	243	0.704544116	-0.159973392	FALSE
GB12622	124	102	0.281770968	-0.58274654	TRUE
GB12623	732	308	1.248913298	0.384395789	FALSE
GB12624	2301	1440	0.676192172	-0.188325336	FALSE
GB12625	1	0	NA	NA	FALSE
GB12626	707	517	0.451545934	-0.412971574	FALSE
GB12627	710	405	0.809897117	-0.054620392	FALSE
GB12628	4785	2872	0.736463176	-0.128054333	FALSE
GB12629	216	166	0.379848071	-0.484669438	FALSE
GB12630	2066	1439	0.521773662	-0.342743846	FALSE
GB12631	1165	588	0.986441895	0.121924386	FALSE
GB12632	1	0	NA	NA	FALSE
GB12633	21367	14472	0.56212005	-0.302397459	FALSE
GB12634	340	251	0.437847382	-0.426670126	FALSE
GB12635	357	220	0.698420551	-0.166096958	FALSE

GB12636	8	2	2	1.135482492	TRUE
GB12637	17384	11750	0.565099323	-0.299418185	FALSE
GB12638	8	12	-0.584962501	-1.449480009	TRUE
GB12639	72	31	1.215728691	0.351211183	FALSE
GB12640	1575	1101	0.51653736	-0.347980149	FALSE
GB12641	58	33	0.813586876	-0.050930633	FALSE
GB12642	NA	NA	NA	NA	FALSE
GB12643	663	356	0.897131629	0.032614121	FALSE
GB12644	1484	932	0.671089232	-0.193428276	FALSE
GB12645	11	22	-1	-1.864517508	TRUE
GB12646	707	525	0.429392792	-0.435124716	FALSE
GB12647	2660	1821	0.546695323	-0.317822185	FALSE
GB12648	99	60	0.722466024	-0.142051484	FALSE
GB12649	5368	2346	1.194181658	0.32966415	FALSE
GB12650	1203	439	1.454343798	0.589826289	TRUE
GB12651	2	2	0	-0.864517508	TRUE
GB12652	995	623	0.675464362	-0.189053146	FALSE
GB12653	1462	919	0.669806545	-0.194710964	FALSE
GB12654	1171	603	0.957511169	0.09299366	FALSE
GB12655	66	35	0.915111102	0.050593594	FALSE
GB12656	740	424	0.803461006	-0.061056502	FALSE
GB12657	24	10	1.263034406	0.398516898	FALSE
GB12658	4354	2382	0.870167994	0.005650486	FALSE
GB12659	NA	NA	NA	NA	FALSE
GB12660	332	164	1.017487427	0.152969918	FALSE
GB12661	4	3	0.415037499	-0.449480009	FALSE
GB12662	5	0	NA	NA	FALSE
GB12663	190	113	0.749676646	-0.114840862	FALSE
GB12664	280	142	0.979535897	0.115018389	FALSE
GB12665	439	248	0.823880819	-0.040636689	FALSE
GB12666	2433	1159	1.069855754	0.205338246	FALSE
GB12667	106	73	0.538095896	-0.326421613	FALSE
GB12668	135	122	0.146078259	-0.718439249	TRUE
GB12669	3	0	NA	NA	FALSE
GB12670	24	5	2.263034406	1.398516898	TRUE
GB12671	13	11	0.2410081	-0.623509409	TRUE
GB12672	8	0	NA	NA	FALSE
GB12673	12	13	-0.115477217	-0.979994726	TRUE
GB12674	814	624	0.383482765	-0.481034743	FALSE
GB12675	238	116	1.036836768	0.17231926	FALSE
GB12676	1360	729	0.899615932	0.035098423	FALSE
GB12677	956	628	0.606246059	-0.258271449	FALSE
GB12678	NA	NA	NA	NA	FALSE
GB12679	1639	915	0.840972206	-0.023545302	FALSE

GB12680	371	161	1.204358499	0.33984099	FALSE
GB12681	11665	6110	0.932942022	0.068424514	FALSE
GB12682	35	25	0.485426827	-0.379090681	FALSE
GB12683	546	311	0.811986371	-0.052531138	FALSE
GB12684	527	373	0.498627331	-0.365890177	FALSE
GB12685	713	413	0.787760295	-0.076757213	FALSE
GB12686	8	5	0.678071905	-0.186445603	FALSE
GB12687	174	56	1.635588574	0.771071065	TRUE
GB12688	7971	3967	1.006712331	0.142194823	FALSE
GB12689	0	3	NA	NA	FALSE
GB12690	457	75	2.607231665	1.742714156	TRUE
GB12691	16438	9982	0.719633969	-0.144883539	FALSE
GB12692	48	26	0.884522783	0.020005274	FALSE
GB12693	6727	3868	0.798375463	-0.066142045	FALSE
GB12694	2104	1236	0.767455961	-0.097061547	FALSE
GB12695	366	192	0.930737338	0.066219829	FALSE
GB12696	101	84	0.26589406	-0.598623448	TRUE
GB12697	309	166	0.896423597	0.031906088	FALSE
GB12698	3461	1773	0.964996405	0.100478897	FALSE
GB12699	41	13	1.657112286	0.792594778	TRUE
GB12700	2029	960	1.079662554	0.215145046	FALSE
GB12701	236	145	0.702733959	-0.161783549	FALSE
GB12702	1114	612	0.864145675	-0.000371834	FALSE
GB12703	6	9	-0.584962501	-1.449480009	TRUE
GB12704	2016	1122	0.845422963	-0.019094545	FALSE
GB12705	862	248	1.797347749	0.93283024	TRUE
GB12706	1479	839	0.817879337	-0.046638172	FALSE
GB12707	6111	3816	0.67934731	-0.185170199	FALSE
GB12708	375	184	1.027184829	0.162667321	FALSE
GB12709	19	4	2.247927513	1.383410005	TRUE
GB12710	1383	798	0.793340505	-0.071177003	FALSE
GB12711	903	608	0.570654664	-0.293862844	FALSE
GB12712	1680	1300	0.36994961	-0.494567899	FALSE
GB12713	196	85	1.205318908	0.3408014	FALSE
GB12714	701	395	0.827561791	-0.036955717	FALSE
GB12715	527	334	0.657954859	-0.206562649	FALSE
GB12716	129970	70771	0.876948445	0.012430937	FALSE
GB12717	581	407	0.513509369	-0.351008139	FALSE
GB12718	440	202	1.123148231	0.258630722	FALSE
GB12719	304	179	0.764111736	-0.100405772	FALSE
GB12720	781	384	1.024216237	0.159698729	FALSE
GB12721	898	411	1.127577051	0.263059543	FALSE
GB12722	579	303	0.934245555	0.069728046	FALSE
GB12723	330	191	0.788893386	-0.075624122	FALSE

GB12724	59	27	1.127755547	0.263238039	FALSE
GB12725	28	14	1	0.135482492	FALSE
GB12726	14	5	1.485426827	0.620909319	TRUE
GB12727	1384	776	0.834715385	-0.029802123	FALSE
GB12728	NA	NA	NA	NA	FALSE
GB12729	NA	NA	NA	NA	FALSE
GB12730	2175	1228	0.82470484	-0.039812668	FALSE
GB12731	1077	776	0.472889692	-0.391627816	FALSE
GB12732	5176	3213	0.687916637	-0.176600872	FALSE
GB12733	6103	3777	0.692277807	-0.172239702	FALSE
GB12734	500	288	0.795859283	-0.068658225	FALSE
GB12735	79	28	1.496425826	0.631908318	TRUE
GB12736	693	425	0.705392511	-0.159124997	FALSE
GB12737	631	328	0.94394419	0.079426682	FALSE
GB12738	1153	824	0.48467627	-0.379841238	FALSE
GB12739	2209	1440	0.617324607	-0.247192901	FALSE
GB12740	505	289	0.805213895	-0.059303613	FALSE
GB12741	61975	40989	0.596449573	-0.268067935	FALSE
GB12742	979	491	0.995585835	0.131068327	FALSE
GB12743	1552	820	0.920432743	0.055915234	FALSE
GB12744	587	363	0.693390955	-0.171126553	FALSE
GB12745	129	38	1.763299742	0.898782234	TRUE
GB12746	738	396	0.898120386	0.033602878	FALSE
GB12747	6399	4397	0.541326934	-0.323190574	FALSE
GB12748	35	16	1.129283017	0.264765509	FALSE
GB12749	2844	1566	0.860837252	-0.003680256	FALSE
GB12750	1176	739	0.670241791	-0.194275718	FALSE
GB12751	460	248	0.891293741	0.026776232	FALSE
GB12752	390	193	1.014873276	0.150355768	FALSE
GB12753	103	103	0	-0.864517508	TRUE
GB12754	230	90	1.353636955	0.489119446	FALSE
GB12755	NA	NA	NA	NA	FALSE
GB12756	954	549	0.797183117	-0.067334391	FALSE
GB12757	NA	NA	NA	NA	FALSE
GB12758	1912	915	1.063238875	0.198721366	FALSE
GB12759	714	375	0.929033479	0.06451597	FALSE
GB12760	1099	596	0.88280715	0.018289642	FALSE
GB12761	2492	1661	0.585251995	-0.279265513	FALSE
GB12762	742	186	1.996116566	1.131599057	TRUE
GB12763	126	55	1.19592021	0.331402702	FALSE
GB12764	819	432	0.922832139	0.058314631	FALSE
GB12765	1190	774	0.620556102	-0.243961406	FALSE
GB12766	108	38	1.506959989	0.64244248	TRUE
GB12767	415	241	0.78407819	-0.080439318	FALSE

GB12768	1490	646	1.205706261	0.341188752	FALSE
GB12769	38	33	0.203533394	-0.660984114	TRUE
GB12770	4	1	2	1.135482492	TRUE
GB12771	5467	3327	0.716527509	-0.147989999	FALSE
GB12772	1	2	-1	-1.864517508	TRUE
GB12773	NA	NA	NA	NA	FALSE
GB12774	346	189	0.872385803	0.007868295	FALSE
GB12775	9077	4340	1.064520515	0.200003006	FALSE
GB12776	194	102	0.9274875	0.062969992	FALSE
GB12777	694	429	0.693958015	-0.170559493	FALSE
GB12778	5281	3361	0.651920599	-0.21259691	FALSE
GB12779	744	437	0.767669342	-0.096848167	FALSE
GB12780	284	153	0.892359277	0.027841768	FALSE
GB12781	2437	2175	0.16409085	-0.700426658	TRUE
GB12782	1652	969	0.769645116	-0.094872392	FALSE
GB12783	43	11	1.966833136	1.102315628	TRUE
GB12784	1143	610	0.905944256	0.041426747	FALSE
GB12785	122	58	1.072756342	0.208238834	FALSE
GB12786	1246	511	1.285908872	0.421391364	FALSE
GB12787	3	0	NA	NA	FALSE
GB12788	4966	2364	1.070854226	0.206336718	FALSE
GB12789	140	120	0.222392421	-0.642125087	TRUE
GB12790	76	36	1.078002512	0.213485004	FALSE
GB12791	33	22	0.584962501	-0.279555008	FALSE
GB12792	637	297	1.100830441	0.236312933	FALSE
GB12793	959	572	0.745515668	-0.11900184	FALSE
GB12794	25	14	0.836501268	-0.028016241	FALSE
GB12795	16812	11228	0.582390393	-0.282127116	FALSE
GB12796	20	13	0.621488377	-0.243029132	FALSE
GB12797	58017	28950	1.002912349	0.138394841	FALSE
GB12798	1655	689	1.264255329	0.399737821	FALSE
GB12799	475	288	0.721858702	-0.142658807	FALSE
GB12800	3565	1467	1.281033206	0.416515697	FALSE
GB12801	2876	1463	0.975133906	0.110616398	FALSE
GB12802	2906	1598	0.862767295	-0.001750214	FALSE
GB12803	427	178	1.262358829	0.39784132	FALSE
GB12804	358	225	0.670034586	-0.194482922	FALSE
GB12805	3	0	NA	NA	FALSE
GB12806	231	69	1.743224585	0.878707076	TRUE
GB12807	1645	995	0.725319153	-0.139198355	FALSE
GB12808	5483	3602	0.606167292	-0.258350216	FALSE
GB12809	NA	NA	NA	NA	FALSE
GB12810	401	214	0.90599144	0.041473932	FALSE
GB12811	91	50	0.86393845	-0.000579058	FALSE

GB12812	4	2	1	0.135482492	FALSE
GB12813	847	492	0.783703654	-0.080813854	FALSE
GB12814	4085	2164	0.916635579	0.052118071	FALSE
GB12815	1	0	NA	NA	FALSE
GB12816	760	337	1.173250827	0.308733319	FALSE
GB12817	139	94	0.564352221	-0.300165287	FALSE
GB12818	21	19	0.144389909	-0.720127599	TRUE
GB12819	84	22	1.932885804	1.068368296	TRUE
GB12820	6	0	NA	NA	FALSE
GB12821	11	6	0.874469118	0.00995161	FALSE
GB12822	67	32	1.06608919	0.201571682	FALSE
GB12823	545	353	0.626588046	-0.237929462	FALSE
GB12824	820	489	0.745789445	-0.118728064	FALSE
GB12825	2214	1267	0.805238698	-0.059278811	FALSE
GB12826	2	0	NA	NA	FALSE
GB12827	3934	2396	0.715369049	-0.149148459	FALSE
GB12828	37	8	2.209453366	1.344935857	TRUE
GB12829	730	557	0.390219136	-0.474298372	FALSE
GB12830	12	4	1.584962501	0.720444992	TRUE
GB12831	166	88	0.915607813	0.051090304	FALSE
GB12832	213	109	0.966525295	0.102007787	FALSE
GB12833	35	9	1.959358016	1.094840507	TRUE
GB12834	3	2	0.584962501	-0.279555008	FALSE
GB12835	547	274	0.99736494	0.132847432	FALSE
GB12836	NA	NA	NA	NA	FALSE
GB12837	98	58	0.756728849	-0.107788659	FALSE
GB12838	492	251	0.970970951	0.106453443	FALSE
GB12839	540	310	0.800691192	-0.063826317	FALSE
GB12840	2352	1234	0.930545666	0.066028157	FALSE
GB12841	1420	1140	0.316857105	-0.547660403	TRUE
GB12842	3690	1612	1.194769072	0.330251564	FALSE
GB12843	1147	661	0.795143215	-0.069374294	FALSE
GB12844	2060	1107	0.895989115	0.031471607	FALSE
GB12845	996	571	0.802654997	-0.061862512	FALSE
GB12846	88	37	1.249978253	0.385460745	FALSE
GB12847	559	314	0.832083724	-0.032433784	FALSE
GB12848	369	106	1.799556551	0.935039043	TRUE
GB12849	252	109	1.209095599	0.34457809	FALSE
GB12850	609	384	0.665335917	-0.199181591	FALSE
GB12851	169	67	1.334790246	0.470272737	FALSE
GB12852	9	11	-0.289506617	-1.154024126	TRUE
GB12853	1216	557	1.126393996	0.261876488	FALSE
GB12854	8897	5668	0.650479194	-0.214038314	FALSE
GB12855	3995	1979	1.013423891	0.148906382	FALSE

GB12856	33	19	0.796466606	-0.068050902	FALSE
GB12857	73	46	0.666262603	-0.198254906	FALSE
GB12858	402	263	0.612132702	-0.252384806	FALSE
GB12859	141	72	0.969626351	0.105108843	FALSE
GB12860	14162	11546	0.294631891	-0.569885617	TRUE
GB12861	374	201	0.895842769	0.03132526	FALSE
GB12862	8	6	0.415037499	-0.449480009	FALSE
GB12863	464	298	0.638812475	-0.225705034	FALSE
GB12864	69	32	1.108524457	0.244006948	FALSE
GB12865	477	319	0.580432842	-0.284084666	FALSE
GB12866	403	199	1.018011408	0.1534939	FALSE
GB12867	1339	811	0.723382141	-0.141135367	FALSE
GB12868	NA	NA	NA	NA	FALSE
GB12869	7	9	-0.362570079	-1.227087588	TRUE
GB12870	1618	964	0.747106556	-0.117410952	FALSE
GB12871	107	44	1.282035368	0.417517859	FALSE
GB12872	1890	1038	0.864579791	824232199282e-05	FALSE
GB12873	109	64	0.768184325	-0.096333184	FALSE
GB12874	188	162	0.214738849	-0.64977866	TRUE
GB12875	2696	1561	0.788349959	-0.076167549	FALSE
GB12876	1051	691	0.605005054	-0.259512455	FALSE
GB12877	NA	NA	NA	NA	FALSE
GB12878	582	288	1.014950341	0.150432833	FALSE
GB12879	2149	1307	0.717406342	-0.147111167	FALSE
GB12880	45	35	0.362570079	-0.501947429	TRUE
GB12881	141	60	1.232660757	0.368143248	FALSE
GB12882	204	72	1.502500341	0.637982832	TRUE
GB12883	34	18	0.91753784	0.053020331	FALSE
GB12884	3935	2089	0.913551143	0.049033635	FALSE
GB12885	1	1	0	-0.864517508	TRUE
GB12886	180	114	0.658963082	-0.205554426	FALSE
GB12887	3845	2472	0.637304855	-0.227212653	FALSE
GB12888	621	356	0.802716027	-0.061801481	FALSE
GB12889	289	137	1.0768936	0.212376091	FALSE
GB12890	87	28	1.635588574	0.771071065	TRUE
GB12891	827	513	0.688928504	-0.175589005	FALSE
GB12892	238	177	0.427212213	-0.437305295	FALSE
GB12893	102	37	1.462971976	0.598454468	TRUE
GB12894	1029	766	0.425826685	-0.438690823	FALSE
GB12895	33428	17909	0.900372258	0.03585475	FALSE
GB12896	343	101	1.763853283	0.899335775	TRUE
GB12897	505	236	1.097496528	0.23297902	FALSE
GB12898	136	103	0.400962314	-0.463555194	FALSE
GB12899	684	318	1.10496956	0.240452051	FALSE

GB12900	260	172	0.596103058	-0.26841445	FALSE
GB12901	1272	1036	0.296074668	-0.568442841	TRUE
GB12902	1138	666	0.772906475	-0.091611033	FALSE
GB12903	511	290	0.817270391	-0.047247117	FALSE
GB12904	1771	916	0.951144709	0.0866272	FALSE
GB12905	504	262	0.943856922	0.079339414	FALSE
GB12906	1323	672	0.977279923	0.112762415	FALSE
GB12907	463	249	0.894866451	0.030348943	FALSE
GB12908	830	372	1.157808715	0.293291207	FALSE
GB12909	273	164	0.735205136	-0.129312372	FALSE
GB12910	921	451	1.030073723	0.165556215	FALSE
GB12911	171	92	0.894290559	0.029773051	FALSE
GB12912	365	239	0.610885846	-0.253631663	FALSE
GB12913	4191	2685	0.642372433	-0.222145075	FALSE
GB12914	444	206	1.107915339	0.243397831	FALSE
GB12915	285	131	1.121395108	0.256877599	FALSE
GB12916	2941	1662	0.823386402	-0.041131106	FALSE
GB12917	3483	2113	0.721037706	-0.143479803	FALSE
GB12918	NA	NA	NA	NA	FALSE
GB12919	368	230	0.678071905	-0.186445603	FALSE
GB12920	976	461	1.082114397	0.217596889	FALSE
GB12921	411	250	0.717210299	-0.147307209	FALSE
GB12922	181	107	0.758378901	-0.106138608	FALSE
GB12923	123	75	0.713695815	-0.150821693	FALSE
GB12924	1327	592	1.16449929	0.299981781	FALSE
GB12925	1574	767	1.037137058	0.17261955	FALSE
GB12926	500	428	0.224317298	-0.64020021	TRUE
GB12927	161	71	1.181169759	0.31665225	FALSE
GB12928	7	8	-0.192645078	-1.057162586	TRUE
GB12929	696	288	1.273018494	0.408500986	FALSE
GB12930	422	246	0.778584683	-0.085932825	FALSE
GB12931	1440	799	0.849801403	-0.014716105	FALSE
GB12932	41	20	1.03562391	0.171106401	FALSE
GB12933	248	118	1.071553261	0.207035753	FALSE
GB12934	428	254	0.7527823	-0.111735209	FALSE
GB12935	115	44	1.386058432	0.521540924	TRUE
GB12936	197	80	1.300123725	0.435606216	FALSE
GB12937	7	3	1.222392421	0.357874913	FALSE
GB12938	3	0	NA	NA	FALSE
GB12939	228	140	0.703606997	-0.160910511	FALSE
GB12940	1761	1058	0.735055282	-0.129462227	FALSE
GB12941	36	24	0.584962501	-0.279555008	FALSE
GB12942	2559	1372	0.899299666	0.034782158	FALSE
GB12943	1	1	0	-0.864517508	TRUE

GB12944	187	86	1.120629705	0.256112197	FALSE
GB12945	621	329	0.916505684	0.051988176	FALSE
GB12946	2973	2035	0.546890669	-0.31762684	FALSE
GB12947	572	228	1.326981323	0.462463814	FALSE
GB12948	2640	1350	0.967578522	0.103061014	FALSE
GB12949	13069	12069	0.114842611	-0.749674897	TRUE
GB12950	5	2	1.321928095	0.457410587	FALSE
GB12951	27270	14724	0.889145045	0.024627537	FALSE
GB12952	580	302	0.941504351	0.076986842	FALSE
GB12953	3803	2530	0.588000554	-0.276516954	FALSE
GB12954	6	3	1	0.135482492	FALSE
GB12955	3064	1548	0.985010826	0.120493317	FALSE
GB12956	127033	81995	0.631595479	-0.232922029	FALSE
GB12957	670	347	0.949225433	0.084707924	FALSE
GB12958	51	16	1.672425342	0.807907834	TRUE
GB12959	NA	NA	NA	NA	FALSE
GB12960	906	511	0.826187759	-0.038329749	FALSE
GB12961	6409	2895	1.146535922	0.282018413	FALSE
GB12962	74	43	0.783188611	-0.081328897	FALSE
GB12963	412	253	0.703506952	-0.161010556	FALSE
GB12964	465	286	0.701215569	-0.163301939	FALSE
GB12965	114	97	0.232977172	-0.631540336	TRUE
GB12966	2274	1208	0.9126118	0.048094291	FALSE
GB12967	2734	1169	1.225738313	0.361220805	FALSE
GB12968	114	47	1.278301162	0.413783654	FALSE
GB12969	101	52	0.957771765	0.093254256	FALSE
GB12970	82	41	1	0.135482492	FALSE
GB12971	503	239	1.073547782	0.209030273	FALSE
GB12972	31	31	0	-0.864517508	TRUE
GB12973	110	68	0.693896872	-0.170620636	FALSE
GB12974	124	70	0.824913293	-0.039604215	FALSE
GB12975	494	177	1.480761682	0.616244173	TRUE
GB12976	861	680	0.340478491	-0.524039017	TRUE
GB12977	1367	822	0.733802944	-0.130714564	FALSE
GB12978	705	439	0.683402318	-0.181115191	FALSE
GB12979	NA	NA	NA	NA	FALSE
GB12980	1122	779	0.526377443	-0.338140066	FALSE
GB12981	1282	741	0.790850814	-0.073666694	FALSE
GB12982	2275	1402	0.698380196	-0.166137312	FALSE
GB12983	1223	706	0.792684315	-0.071833193	FALSE
GB12984	367	211	0.798537064	-0.065980444	FALSE
GB12985	114	70	0.703606997	-0.160910511	FALSE
GB12986	802	478	0.746591618	-0.11792589	FALSE
GB12987	6321	3267	0.95218636	0.087668852	FALSE

GB12988	1169	681	0.779548227	-0.084969282	FALSE
GB12989	640	440	0.540568381	-0.323949127	FALSE
GB12990	66	27	1.289506617	0.424989109	FALSE
GB12991	506	292	0.793169016	-0.071348493	FALSE
GB12992	1	0	NA	NA	FALSE
GB12993	494	292	0.758542673	-0.105974836	FALSE
GB12994	499	327	0.60974918	-0.254768328	FALSE
GB12995	1288	621	1.05246742	0.187949912	FALSE
GB12996	114	56	1.025535092	0.161017584	FALSE
GB12997	677	324	1.063162021	0.198644512	FALSE
GB12998	138	77	0.841737916	-0.022779592	FALSE
GB12999	240	168	0.514573173	-0.349944335	FALSE
GB13000	899	553	0.701041635	-0.163475873	FALSE
GB13001	183	87	1.072756342	0.208238834	FALSE
GB13002	127	59	1.106041637	0.241524129	FALSE
GB13003	808	528	0.613817363	-0.250700145	FALSE
GB13004	13525	6473	1.063122185	0.198604677	FALSE
GB13005	2099	1265	0.730564782	-0.133952727	FALSE
GB13006	1	0	NA	NA	FALSE
GB13007	132	50	1.40053793	0.536020421	TRUE
GB13008	2	0	NA	NA	FALSE
GB13009	267	209	0.3533368	-0.511180709	TRUE
GB13010	767	581	0.400688414	-0.463829094	FALSE
GB13011	NA	NA	NA	NA	FALSE
GB13012	192	136	0.497499659	-0.367017849	FALSE
GB13013	257	151	0.76721981	-0.097297698	FALSE
GB13014	409	207	0.982470075	0.117952567	FALSE
GB13015	887	545	0.702677875	-0.161839634	FALSE
GB13016	7	3	1.222392421	0.357874913	FALSE
GB13017	916	638	0.521791174	-0.342726334	FALSE
GB13018	438	228	0.941897045	0.077379537	FALSE
GB13019	1287	702	0.874469118	0.00995161	FALSE
GB13020	NA	NA	NA	NA	FALSE
GB13021	1048	541	0.953938218	0.089420709	FALSE
GB13022	2	1	1	0.135482492	FALSE
GB13023	1943	1064	0.86878775	0.004270242	FALSE
GB13024	510	338	0.593474001	-0.271043508	FALSE
GB13025	NA	NA	NA	NA	FALSE
GB13026	NA	NA	NA	NA	FALSE
GB13027	1398	857	0.705997251	-0.158520257	FALSE
GB13028	374	246	0.604379955	-0.260137554	FALSE
GB13029	206	78	1.401098308	0.5365808	TRUE
GB13030	309	128	1.271463028	0.40694552	FALSE
GB13031	709	359	0.981801783	0.117284275	FALSE

GB13032	274	159	0.785149128	-0.079368381	FALSE
GB13033	6274	4280	0.551774733	-0.312742776	FALSE
GB13034	533	251	1.086448169	0.22193066	FALSE
GB13035	988	450	1.13458604	0.270068532	FALSE
GB13036	18	11	0.710493383	-0.154024126	FALSE
GB13037	14448	5452	1.406012331	0.541494822	TRUE
GB13038	312	166	0.910362788	0.045845279	FALSE
GB13039	NA	NA	NA	NA	FALSE
GB13040	561	252	1.154577037	0.290059529	FALSE
GB13041	504	256	0.977279923	0.112762415	FALSE
GB13042	8664	4425	0.969355788	0.104838279	FALSE
GB13043	1054	629	0.744742945	-0.119774564	FALSE
GB13044	316	165	0.937458534	0.072941026	FALSE
GB13045	3770	1560	1.273018494	0.408500986	FALSE
GB13046	376	182	1.046794211	0.182276703	FALSE
GB13047	2816	1705	0.723875595	-0.140641914	FALSE
GB13048	810	541	0.582293314	-0.282224194	FALSE
GB13049	7380	2685	1.458698728	0.59418122	TRUE
GB13050	107	38	1.493539473	0.629021965	TRUE
GB13051	1377	722	0.931457817	0.066940309	FALSE
GB13052	224	92	1.283792966	0.419275458	FALSE
GB13053	54	37	0.545434137	-0.319083372	FALSE
GB13054	3	1	1.584962501	0.720444992	TRUE
GB13055	4347	3094	0.490546899	-0.373970609	FALSE
GB13056	905	408	1.14934864	0.284831132	FALSE
GB13057	487	267	0.86708203	0.002564522	FALSE
GB13058	103079	52199	0.981656373	0.117138864	FALSE
GB13059	1901	688	1.466278062	0.601760554	TRUE
GB13060	8260	4000	1.046141782	0.181624273	FALSE
GB13061	206	135	0.60968493	-0.254832578	FALSE
GB13062	941	578	0.70312523	-0.161392278	FALSE
GB13063	1727	1124	0.619626047	-0.244891461	FALSE
GB13064	26	7	1.893084796	1.028567288	TRUE
GB13065	49	24	1.029747343	0.165229835	FALSE
GB13066	330	190	0.796466606	-0.068050902	FALSE
GB13067	301	190	0.663764068	-0.20075344	FALSE
GB13068	402	279	0.526930379	-0.337587129	FALSE
GB13069	147	111	0.405256478	-0.45926103	FALSE
GB13070	265	113	1.229669587	0.365152079	FALSE
GB13071	2574	1440	0.837943242	-0.026574266	FALSE
GB13072	11687	7715	0.599166582	-0.265350926	FALSE
GB13073	4357	2929	0.57292692	-0.291590588	FALSE
GB13074	3134	1843	0.765949109	-0.098568399	FALSE
GB13075	462	222	1.057333175	0.192815667	FALSE

GB13076	144	126	0.192645078	-0.67187243	TRUE
GB13077	242	124	0.964666927	0.100149419	FALSE
GB13078	1958	1275	0.618883518	-0.24563399	FALSE
GB13079	61	28	1.123382416	0.258864907	FALSE
GB13080	640	335	0.93391081	0.069393301	FALSE
GB13081	45	56	-0.315501826	-1.180019334	TRUE
GB13082	5111	3686	0.47154952	-0.392967988	FALSE
GB13083	1	1	0	-0.864517508	TRUE
GB13084	28	47	-0.74723393	-1.611751438	TRUE
GB13085	677	384	0.818049523	-0.046467985	FALSE
GB13086	52	13	2	1.135482492	TRUE
GB13087	883	438	1.011482568	0.14696506	FALSE
GB13088	5	2	1.321928095	0.457410587	FALSE
GB13089	5	5	0	-0.864517508	TRUE
GB13090	37	16	1.209453366	0.344935857	FALSE
GB13091	553	223	1.31023577	0.445718262	FALSE
GB13092	156	73	1.09557766	0.231060152	FALSE
GB13093	146	65	1.167456746	0.302939238	FALSE
GB13094	1197	678	0.820065974	-0.044451535	FALSE
GB13095	34	18	0.91753784	0.053020331	FALSE
GB13096	0	1	NA	NA	FALSE
GB13097	160	64	1.321928095	0.457410587	FALSE
GB13098	1750	1076	0.701676844	-0.162840664	FALSE
GB13099	1760	1118	0.654655241	-0.209862268	FALSE
GB13100	428	225	0.927685795	0.063168287	FALSE
GB13101	2935	1866	0.653411517	-0.211105991	FALSE
GB13102	265	134	0.983759359	0.119241851	FALSE
GB13103	58	34	0.770518154	-0.093999354	FALSE
GB13104	4677	2571	0.863253819	-0.00126369	FALSE
GB13105	17	21	-0.304854582	-1.16937209	TRUE
GB13106	72	26	1.469485283	0.604967775	TRUE
GB13107	3504	1940	0.852946123	-0.011571386	FALSE
GB13108	172	44	1.966833136	1.102315628	TRUE
GB13109	11805	5237	1.172585532	0.308068023	FALSE
GB13110	2123	1318	0.687754001	-0.176763507	FALSE
GB13111	600	274	1.130786608	0.266269099	FALSE
GB13112	896	570	0.652536813	-0.211980695	FALSE
GB13113	213	82	1.377157616	0.512640107	TRUE
GB13114	626	384	0.705056346	-0.159461162	FALSE
GB13115	11971	5960	1.006159437	0.141641929	FALSE
GB13116	7	7	0	-0.864517508	TRUE
GB13117	1397	688	1.021851551	0.157334042	FALSE
GB13118	2751	1773	0.633763603	-0.230753905	FALSE
GB13119	5162	3243	0.670601118	-0.193916391	FALSE

GB13120	109	46	1.244622369	0.38010486	FALSE
GB13121	1	0	NA	NA	FALSE
GB13122	NA	NA	NA	NA	FALSE
GB13123	1	5	-2.321928095	-3.186445603	TRUE
GB13124	293	135	1.117941257	0.253423749	FALSE
GB13125	751	300	1.323850407	0.459332899	FALSE
GB13126	967	591	0.710357759	-0.154159749	FALSE
GB13127	1234	742	0.733851303	-0.130666206	FALSE
GB13128	425	196	1.116609187	0.252091679	FALSE
GB13129	655	234	1.484986377	0.620468869	TRUE
GB13130	1440	832	0.791413378	-0.07310413	FALSE
GB13131	NA	NA	NA	NA	FALSE
GB13132	752	403	0.899952823	0.035435315	FALSE
GB13133	3	0	NA	NA	FALSE
GB13134	224	126	0.830074999	-0.03444251	FALSE
GB13135	5052	2639	0.936863288	0.07234578	FALSE
GB13136	257	125	1.039840265	0.175322756	FALSE
GB13137	1004	610	0.718878122	-0.145639387	FALSE
GB13138	144	93	0.63076619	-0.233751318	FALSE
GB13139	682	442	0.62572537	-0.238792139	FALSE
GB13140	1122	707	0.666290556	-0.198226953	FALSE
GB13141	4	0	NA	NA	FALSE
GB13142	339	211	0.684042274	-0.180475234	FALSE
GB13143	336	186	0.853158612	-0.011358897	FALSE
GB13144	424	259	0.711112167	-0.153405341	FALSE
GB13145	994	395	1.331393198	0.46687569	FALSE
GB13146	7	4	0.807354922	-0.057162586	FALSE
GB13147	47	17	1.46712601	0.602608502	TRUE
GB13148	50	26	0.943416472	0.078898963	FALSE
GB13149	NA	NA	NA	NA	FALSE
GB13150	44	30	0.552541023	-0.311976485	FALSE
GB13151	NA	NA	NA	NA	FALSE
GB13152	5436	2347	1.211727614	0.347210106	FALSE
GB13153	767	419	0.872276334	0.007758825	FALSE
GB13154	249	182	0.452207292	-0.412310216	FALSE
GB13155	180	60	1.584962501	0.720444992	TRUE
GB13156	1050	636	0.723290657	-0.141226851	FALSE
GB13157	53	39	0.442518236	-0.421999273	FALSE
GB13158	8601	5295	0.699873721	-0.164643787	FALSE
GB13159	1504	658	1.192645078	0.32812757	FALSE
GB13160	19342	11905	0.700169359	-0.164348149	FALSE
GB13161	2466	1596	0.627712148	-0.23680536	FALSE
GB13162	276	141	0.968973104	0.104455596	FALSE
GB13163	22	6	1.874469118	1.00995161	TRUE

GB13164	1295	748	0.791841923	-0.072675586	FALSE
GB13165	219	150	0.545968369	-0.318549139	FALSE
GB13166	83	56	0.567684509	-0.296832999	FALSE
GB13167	766	425	0.849881551	-0.014635957	FALSE
GB13168	1270	828	0.617125824	-0.247391684	FALSE
GB13169	4	0	NA	NA	FALSE
GB13170	1583	751	1.075776443	0.211258934	FALSE
GB13171	99	61	0.698619283	-0.165898226	FALSE
GB13172	46	12	1.938599455	1.074081947	TRUE
GB13173	758	408	0.893628696	0.029111188	FALSE
GB13174	90	42	1.099535674	0.235018165	FALSE
GB13175	4022	2201	0.869753936	0.005236428	FALSE
GB13176	547	339	0.69025556	-0.174261949	FALSE
GB13177	NA	NA	NA	NA	FALSE
GB13178	81	43	0.913585248	0.04906774	FALSE
GB13179	7	2	1.807354922	0.942837414	TRUE
GB13180	910	385	1.2410081	0.376490591	FALSE
GB13181	102	49	1.057715498	0.19319799	FALSE
GB13182	1766	1141	0.630186551	-0.234330957	FALSE
GB13183	138	93	0.569365646	-0.295151863	FALSE
GB13184	1879	1188	0.661430231	-0.203087278	FALSE
GB13185	31	17	0.866733469	0.002215961	FALSE
GB13186	491	221	1.151676655	0.287159147	FALSE
GB13187	409	307	0.413862188	-0.450655321	FALSE
GB13188	1334	744	0.84238414	-0.022133368	FALSE
GB13189	NA	NA	NA	NA	FALSE
GB13190	1855	971	0.933875986	0.069358478	FALSE
GB13191	918	641	0.518169797	-0.346347712	FALSE
GB13192	1073	550	0.964146552	0.099629044	FALSE
GB13193	30	13	1.206450877	0.341933369	FALSE
GB13194	12	1	3.584962501	2.720444992	TRUE
GB13195	116	51	1.185555653	0.321038145	FALSE
GB13196	205	123	0.736965594	-0.127551914	FALSE
GB13197	345	189	0.868210127	0.003692619	FALSE
GB13198	2685	1685	0.672173497	-0.192344012	FALSE
GB13199	833	439	0.924095556	0.059578048	FALSE
GB13200	NA	NA	NA	NA	FALSE
GB13201	NA	NA	NA	NA	FALSE
GB13202	NA	NA	NA	NA	FALSE
GB13203	309	114	1.438573014	0.574055505	TRUE
GB13204	845	375	1.172060746	0.307543237	FALSE
GB13205	291	172	0.758610588	-0.10590692	FALSE
GB13206	645	427	0.595063091	-0.269454418	FALSE
GB13207	NA	NA	NA	NA	FALSE

GB13208	127	51	1.316259345	0.451741836	FALSE
GB13209	233	87	1.421242649	0.55672514	TRUE
GB13210	7782	6671	0.222237939	-0.642279569	TRUE
GB13211	324	196	0.725140159	-0.13937735	FALSE
GB13212	80	48	0.736965594	-0.127551914	FALSE
GB13213	35320	20756	0.766956902	-0.097560606	FALSE
GB13214	2956	1800	0.715649363	-0.148868145	FALSE
GB13215	4004	1956	1.033535604	0.169018096	FALSE
GB13216	308	236	0.384143491	-0.480374017	FALSE
GB13217	467	255	0.872925303	0.008407795	FALSE
GB13218	106	45	1.236067358	0.37154985	FALSE
GB13219	563	261	1.109085116	0.244567607	FALSE
GB13220	5059	3248	0.639300607	-0.225216902	FALSE
GB13221	29	11	1.398549376	0.534031868	TRUE
GB13222	1454	977	0.573596802	-0.290920706	FALSE
GB13223	30144	17778	0.761777838	-0.10273967	FALSE
GB13224	4324	2129	1.022190573	0.157673065	FALSE
GB13225	1988	1012	0.974108467	0.109590959	FALSE
GB13226	414	255	0.699133521	-0.165383988	FALSE
GB13227	869	452	0.943033404	0.078515896	FALSE
GB13228	10149	4354	1.22092427	0.356406762	FALSE
GB13229	8453	3713	1.186878135	0.322360626	FALSE
GB13230	365	130	1.489384841	0.624867332	TRUE
GB13231	2621	1671	0.64940562	-0.215111888	FALSE
GB13232	5	2	1.321928095	0.457410587	FALSE
GB13233	NA	NA	NA	NA	FALSE
GB13234	1	2	-1	-1.864517508	TRUE
GB13235	46	15	1.61667136	0.752153852	TRUE
GB13236	9146	3896	1.231147242	0.366629733	FALSE
GB13237	3400	2705	0.329906152	-0.534611356	TRUE
GB13238	2582	1027	1.330052819	0.465535311	FALSE
GB13239	2753	1656	0.733301937	-0.131215571	FALSE
GB13240	2022	1225	0.723001248	-0.14151626	FALSE
GB13241	35736	20213	0.822094698	-0.04242281	FALSE
GB13242	406	236	0.782692868	-0.081824641	FALSE
GB13243	50	21	1.251538767	0.387021259	FALSE
GB13244	43	28	0.618909833	-0.245607676	FALSE
GB13245	3062	1503	1.026629274	0.162111765	FALSE
GB13246	186	73	1.349334252	0.484816744	FALSE
GB13247	1	0	NA	NA	FALSE
GB13248	1	1	0	-0.864517508	TRUE
GB13249	1441	765	0.913538683	0.049021174	FALSE
GB13250	NA	NA	NA	NA	FALSE
GB13251	7493	4466	0.7465602	-0.117957308	FALSE

GB13252	4	1	2	1.135482492	TRUE
GB13253	364	177	1.04018909	0.175671582	FALSE
GB13254	727	410	0.826331454	-0.038186054	FALSE
GB13255	238	115	1.049327712	0.184810204	FALSE
GB13256	2013	1128	0.835580105	-0.028937404	FALSE
GB13257	180	70	1.362570079	0.498052571	FALSE
GB13258	179	115	0.638325726	-0.226191782	FALSE
GB13259	288	177	0.702319451	-0.162198057	FALSE
GB13260	23	8	1.523561956	0.659044448	TRUE
GB13261	625	302	1.04930764	0.184790132	FALSE
GB13262	407	241	0.755995648	-0.10852186	FALSE
GB13263	5285	2073	1.350183355	0.485665847	FALSE
GB13264	69	44	0.649092838	-0.21542467	FALSE
GB13265	180	99	0.862496476	-0.002021032	FALSE
GB13266	173	75	1.205809537	0.341292029	FALSE
GB13267	2	0	NA	NA	FALSE
GB13268	64	26	1.299560282	0.435042774	FALSE
GB13269	246	172	0.516249751	-0.348267758	FALSE
GB13270	1	1	0	-0.864517508	TRUE
GB13271	870	574	0.599964664	-0.264552844	FALSE
GB13272	2143	1044	1.037510138	0.17299263	FALSE
GB13273	NA	NA	NA	NA	FALSE
GB13274	3	4	-0.415037499	-1.279555008	TRUE
GB13275	2997	1666	0.847130683	-0.017386825	FALSE
GB13276	66	39	0.7589919	-0.105525608	FALSE
GB13277	324	218	0.571665678	-0.29285183	FALSE
GB13278	NA	NA	NA	NA	FALSE
GB13279	21	13	0.691877705	-0.172639804	FALSE
GB13280	358	204	0.811390435	-0.053127073	FALSE
GB13281	10	3	1.736965594	0.872448086	TRUE
GB13282	1947	1231	0.661422122	-0.203095386	FALSE
GB13283	396	267	0.568660688	-0.29585682	FALSE
GB13284	1475	1073	0.459064878	-0.40545263	FALSE
GB13285	NA	NA	NA	NA	FALSE
GB13286	578	379	0.608871644	-0.255645864	FALSE
GB13287	110	75	0.552541023	-0.311976485	FALSE
GB13288	NA	NA	NA	NA	FALSE
GB13289	15983	6063	1.398434499	0.533916991	TRUE
GB13290	1720	864	0.993305347	0.128787839	FALSE
GB13291	706	418	0.756165241	-0.108352267	FALSE
GB13292	3708	1684	1.138749106	0.274231597	FALSE
GB13293	1111	473	1.231946728	0.36742922	FALSE
GB13294	NA	NA	NA	NA	FALSE
GB13295	1342	999	0.425828088	-0.43868942	FALSE

GB13296	31	21	0.561878888	-0.302638621	FALSE
GB13297	1760	959	0.875972709	0.0114552	FALSE
GB13298	3163	1323	1.257480494	0.392962986	FALSE
GB13299	6	5	0.263034406	-0.601483102	TRUE
GB13300	294	151	0.961267606	0.096750097	FALSE
GB13301	NA	NA	NA	NA	FALSE
GB13302	256	135	0.923184403	0.058666895	FALSE
GB13303	440	204	1.108934372	0.244416863	FALSE
GB13304	595	297	1.002426737	0.137909229	FALSE
GB13305	NA	NA	NA	NA	FALSE
GB13306	6	3	1	0.135482492	FALSE
GB13307	7	4	0.807354922	-0.057162586	FALSE
GB13308	5838	2897	1.010914526	0.146397018	FALSE
GB13309	447	282	0.664579669	-0.19993784	FALSE
GB13310	642	319	1.009016873	0.144499365	FALSE
GB13311	667	378	0.819300527	-0.045216981	FALSE
GB13312	682	369	0.886150923	0.021633415	FALSE
GB13313	178	96	0.89077093	0.026253422	FALSE
GB13314	304	150	1.019108823	0.154591315	FALSE
GB13315	444	219	1.019628807	0.155111298	FALSE
GB13316	14	9	0.637429921	-0.227087588	FALSE
GB13317	591	303	0.963840337	0.099322828	FALSE
GB13318	NA	NA	NA	NA	FALSE
GB13319	512	297	0.785680879	-0.078836629	FALSE
GB13320	2735	1803	0.601141436	-0.263376072	FALSE
GB13321	1489	910	0.710405303	-0.154112205	FALSE
GB13322	374	257	0.541269911	-0.323247598	FALSE
GB13323	1072	612	0.808701348	-0.055816161	FALSE
GB13324	5669	3706	0.613231388	-0.251286121	FALSE
GB13325	54	53	0.026967048	-0.837550461	TRUE
GB13326	49	23	1.091147888	0.22663038	FALSE
GB13327	1347	669	1.009671735	0.145154226	FALSE
GB13328	643	349	0.881591701	0.017074193	FALSE
GB13329	337	145	1.216695691	0.352178183	FALSE
GB13330	72	46	0.646363045	-0.218154463	FALSE
GB13331	180	93	0.952694285	0.088176777	FALSE
GB13332	17305	12424	0.478059207	-0.386458302	FALSE
GB13333	190	95	1	0.135482492	FALSE
GB13334	535	315	0.764187063	-0.100330445	FALSE
GB13335	5107	2828	0.852693939	-0.011823569	FALSE
GB13336	253	157	0.688372826	-0.176144683	FALSE
GB13337	2654	1365	0.95926742	0.094749911	FALSE
GB13338	658	375	0.811196988	-0.05332052	FALSE
GB13339	64	58	0.142019005	-0.722498503	TRUE

GB13340	434	279	0.637429921	-0.227087588	FALSE
GB13341	417	282	0.564352221	-0.300165287	FALSE
GB13342	254	163	0.639956533	-0.224560976	FALSE
GB13343	1036	529	0.969684376	0.105166867	FALSE
GB13344	2581	1429	0.852924225	-0.011593283	FALSE
GB13345	128	77	0.733213459	-0.131304049	FALSE
GB13346	1844	915	1.010995007	0.146477499	FALSE
GB13347	952	623	0.61172941	-0.252788098	FALSE
GB13348	245	127	0.947953252	0.083435744	FALSE
GB13349	35	19	0.881355504	0.016837995	FALSE
GB13350	1288	702	0.875589658	0.011072149	FALSE
GB13351	4	4	0	-0.864517508	TRUE
GB13352	5	4	0.321928095	-0.542589413	TRUE
GB13353	7	2	1.807354922	0.942837414	TRUE
GB13354	717	461	0.637206368	-0.22731114	FALSE
GB13355	77	43	0.840521786	-0.023995722	FALSE
GB13356	59	32	0.882643049	0.018125541	FALSE
GB13357	4	4	0	-0.864517508	TRUE
GB13358	159	73	1.123058396	0.258540888	FALSE
GB13359	591	323	0.871623965	0.007106457	FALSE
GB13360	10	9	0.152003093	-0.712514415	TRUE
GB13361	45	18	1.321928095	0.457410587	FALSE
GB13362	338	222	0.60646357	-0.258053938	FALSE
GB13363	461	267	0.787927009	-0.0765905	FALSE
GB13364	NA	NA	NA	NA	FALSE
GB13365	2	0	NA	NA	FALSE
GB13366	1284	698	0.879346261	0.014828753	FALSE
GB13367	163	106	0.6208077	-0.243709809	FALSE
GB13368	24818	18266	0.44222612	-0.422291389	FALSE
GB13369	63	32	0.977279923	0.112762415	FALSE
GB13370	947	633	0.581158926	-0.283358582	FALSE
GB13371	211	140	0.591816172	-0.272701337	FALSE
GB13372	578	388	0.57501284	-0.289504668	FALSE
GB13373	1193	568	1.070631208	0.2061137	FALSE
GB13374	238	122	0.964080426	0.099562917	FALSE
GB13375	2365	1265	0.902702799	0.03818529	FALSE
GB13376	185	156	0.245979242	-0.618538267	TRUE
GB13377	1	0	NA	NA	FALSE
GB13378	1882	1208	0.639646173	-0.224871335	FALSE
GB13379	431	226	0.931365097	0.066847588	FALSE
GB13380	33635	18153	0.889755268	0.025237759	FALSE
GB13381	NA	NA	NA	NA	FALSE
GB13382	359	220	0.70648032	-0.158037188	FALSE
GB13383	618	359	0.783622994	-0.080894514	FALSE

GB13384	2258	1350	0.742086079	-0.122431429	FALSE
GB13385	1	0	NA	NA	FALSE
GB13386	498	241	1.047112596	0.182595088	FALSE
GB13387	392	206	0.928209317	0.063691809	FALSE
GB13388	1088	664	0.71242341	-0.152094098	FALSE
GB13389	199	112	0.829269698	-0.03524781	FALSE
GB13390	1	1	0	-0.864517508	TRUE
GB13391	56	35	0.678071905	-0.186445603	FALSE
GB13392	288	149	0.950756481	0.086238973	FALSE
GB13393	1096	581	0.91563773	0.051120221	FALSE
GB13394	201	115	0.80556164	-0.058955868	FALSE
GB13395	4620	2358	0.970329133	0.105811625	FALSE
GB13396	40	32	0.321928095	-0.542589413	TRUE
GB13397	913	310	1.558346645	0.693829136	TRUE
GB13398	72	37	0.960471636	0.095954127	FALSE
GB13399	44685	21572	1.050630685	0.186113177	FALSE
GB13400	2030	1071	0.922521247	0.058003739	FALSE
GB13401	128033	56819	1.172070361	0.307552853	FALSE
GB13402	1248	677	0.882390195	0.017872687	FALSE
GB13403	140	94	0.574694165	-0.289823343	FALSE
GB13404	1050	607	0.790620906	-0.073896602	FALSE
GB13405	847	409	1.050261126	0.185743618	FALSE
GB13406	1058	651	0.700610179	-0.163907329	FALSE
GB13407	1161	650	0.836856349	-0.027661159	FALSE
GB13408	1	0	NA	NA	FALSE
GB13409	22	5	2.137503524	1.272986015	TRUE
GB13410	477	293	0.703088602	-0.161428907	FALSE
GB13411	1267	782	0.696176012	-0.168341496	FALSE
GB13412	2	0	NA	NA	FALSE
GB13413	291	191	0.607446515	-0.257070993	FALSE
GB13414	42	23	0.868755467	0.004237958	FALSE
GB13415	470	293	0.681760092	-0.182757416	FALSE
GB13416	1736	1292	0.426160878	-0.438356631	FALSE
GB13417	77	42	0.874469118	0.00995161	FALSE
GB13418	155	94	0.721535554	-0.142981955	FALSE
GB13419	11	2	2.459431619	1.59491411	TRUE
GB13420	245	204	0.264212597	-0.600304911	TRUE
GB13421	1460	693	1.075041112	0.210523603	FALSE
GB13422	1431	695	1.041938789	0.177421281	FALSE
GB13423	66	54	0.289506617	-0.575010891	TRUE
GB13424	465	241	0.94819757	0.083680061	FALSE
GB13425	209	111	0.912943266	0.048425757	FALSE
GB13426	424	188	1.173331603	0.308814095	FALSE
GB13427	4928	2925	0.752565631	-0.111951877	FALSE

GB13428	669	351	0.93053518	0.066017672	FALSE
GB13429	3226	1611	1.001789944	0.137272436	FALSE
GB13430	100	43	1.217591435	0.353073927	FALSE
GB13431	362	168	1.107528464	0.243010956	FALSE
GB13432	1276	731	0.803685018	-0.060832491	FALSE
GB13433	717	496	0.531632998	-0.33288451	FALSE
GB13434	302	204	0.565979397	-0.298538111	FALSE
GB13435	286	201	0.508819646	-0.355697863	FALSE
GB13436	1142	765	0.578030998	-0.286486511	FALSE
GB13437	869	431	1.011668308	0.147150799	FALSE
GB13438	43	14	1.618909833	0.754392324	TRUE
GB13439	NA	NA	NA	NA	FALSE
GB13440	257	164	0.648072545	-0.216444964	FALSE
GB13441	5234	2717	0.945899359	0.08138185	FALSE
GB13442	104	56	0.893084796	0.028567288	FALSE
GB13443	160	79	1.018147347	0.153629838	FALSE
GB13444	177	118	0.584962501	-0.279555008	FALSE
GB13445	787	379	1.054165787	0.189648279	FALSE
GB13446	50	18	1.473931188	0.60941368	TRUE
GB13447	489	282	0.794139303	-0.070378206	FALSE
GB13448	12	10	0.263034406	-0.601483102	TRUE
GB13449	2833	1478	0.938684332	0.074166824	FALSE
GB13450	8	0	NA	NA	FALSE
GB13451	333	143	1.21950703	0.354989522	FALSE
GB13452	NA	NA	NA	NA	FALSE
GB13453	27	14	0.94753258	0.083015072	FALSE
GB13454	676	412	0.714378909	-0.150138599	FALSE
GB13455	28	11	1.347923303	0.483405795	FALSE
GB13456	1247	772	0.691788713	-0.172728796	FALSE
GB13457	57	90	-0.658963082	-1.52348059	TRUE
GB13458	7297	4031	0.856165671	-0.008351838	FALSE
GB13459	499	194	1.362983163	0.498465655	FALSE
GB13460	5559	3139	0.824520353	-0.039997155	FALSE
GB13461	381	242	0.65478395	-0.209733558	FALSE
GB13462	353	126	1.48624445	0.621726941	TRUE
GB13463	92	63	0.546282033	-0.318235476	FALSE
GB13464	1695	930	0.865982652	0.001465144	FALSE
GB13465	242	180	0.427010141	-0.437507367	FALSE
GB13466	6	1	2.584962501	1.720444992	TRUE
GB13467	2953	1844	0.679342701	-0.185174808	FALSE
GB13468	NA	NA	NA	NA	FALSE
GB13469	2948	1710	0.785740199	-0.078777309	FALSE
GB13470	1406	758	0.891326841	0.026809333	FALSE
GB13471	113	64	0.820178962	-0.044338546	FALSE

GB13472	3073	2110	0.542404768	-0.32211274	FALSE
GB13473	224	138	0.698830465	-0.165687043	FALSE
GB13474	45	45	0	-0.864517508	TRUE
GB13475	257	122	1.074887212	0.210369703	FALSE
GB13476	NA	NA	NA	NA	FALSE
GB13477	129	51	1.338801913	0.474284405	FALSE
GB13478	638	362	0.817566727	-0.046950782	FALSE
GB13479	2036	1100	0.888234038	0.023716529	FALSE
GB13480	505	275	0.876851769	0.012334261	FALSE
GB13481	4	1	2	1.135482492	TRUE
GB13482	1126	724	0.637145225	-0.227372283	FALSE
GB13483	6135	3105	0.982470075	0.117952567	FALSE
GB13484	9982	4201	1.248596118	0.38407861	FALSE
GB13485	3	2	0.584962501	-0.279555008	FALSE
GB13486	2	0	NA	NA	FALSE
GB13487	58	25	1.214124805	0.349607297	FALSE
GB13488	720	455	0.662130361	-0.202387147	FALSE
GB13489	653	362	0.851093294	-0.013424214	FALSE
GB13490	NA	NA	NA	NA	FALSE
GB13491	736	391	0.912537159	0.04801965	FALSE
GB13492	7665	3329	1.203196921	0.338679413	FALSE
GB13493	8	3	1.415037499	0.550519991	TRUE
GB13494	75	54	0.473931188	-0.39058632	FALSE
GB13495	1475	883	0.740229611	-0.124287897	FALSE
GB13496	45	9	2.321928095	1.457410587	TRUE
GB13497	2785	1525	0.868868085	0.004350577	FALSE
GB13498	40	17	1.234465254	0.369947745	FALSE
GB13499	2075	1168	0.829071062	-0.035446446	FALSE
GB13500	33	10	1.722466024	0.857948516	TRUE
GB13501	446	207	1.107412942	0.242895434	FALSE
GB13502	2	2	0	-0.864517508	TRUE
GB13503	655	287	1.19044417	0.325926661	FALSE
GB13504	NA	NA	NA	NA	FALSE
GB13505	3250	1404	1.210896782	0.346379274	FALSE
GB13506	182	106	0.779874186	-0.084643323	FALSE
GB13507	1731	1342	0.367221053	-0.497296455	FALSE
GB13508	5336	3275	0.70426376	-0.160253748	FALSE
GB13509	19	7	1.440572591	0.576055083	TRUE
GB13510	1280	828	0.628441137	-0.236076371	FALSE
GB13511	226	134	0.754089772	-0.110427736	FALSE
GB13512	294	223	0.398772445	-0.465745063	FALSE
GB13513	1	3	-1.584962501	-2.449480009	TRUE
GB13514	1928	1163	0.729253955	-0.135263554	FALSE
GB13515	1894	1088	0.799757774	-0.064759734	FALSE

GB13516	730	345	1.081300102	0.216782594	FALSE
GB13517	143	58	1.301890342	0.437372833	FALSE
GB13518	3185	1524	1.06343047	0.198912961	FALSE
GB13519	5632	3203	0.814223536	-0.050293972	FALSE
GB13520	1036	497	1.059706246	0.195188738	FALSE
GB13521	1	1	0	-0.864517508	TRUE
GB13522	1923	1057	0.863383386	-0.001134122	FALSE
GB13523	1557	949	0.714288952	-0.150228556	FALSE
GB13524	577	323	0.837037154	-0.027480354	FALSE
GB13525	216	133	0.699605067	-0.164912442	FALSE
GB13526	1245	777	0.680159239	-0.18435827	FALSE
GB13527	36	52	-0.530514717	-1.395032225	TRUE
GB13528	695	407	0.771984183	-0.092533325	FALSE
GB13529	2935	1786	0.716628423	-0.147889085	FALSE
GB13530	331	220	0.589327693	-0.275189815	FALSE
GB13531	1579	911	0.793488212	-0.071029296	FALSE
GB13532	648	403	0.685213974	-0.179303534	FALSE
GB13533	1492	757	0.97888233	0.114364822	FALSE
GB13534	102	78	0.387023123	-0.477494385	FALSE
GB13535	1217	767	0.666030685	-0.198486823	FALSE
GB13536	1811	953	0.926238427	0.061720919	FALSE
GB13537	175	105	0.736965594	-0.127551914	FALSE
GB13538	103	54	0.931613025	0.067095517	FALSE
GB13539	5312	2850	0.898293227	0.033775719	FALSE
GB13540	1334	1042	0.356403389	-0.508114119	TRUE
GB13541	14	20	-0.514573173	-1.379090681	TRUE
GB13542	24	7	1.777607579	0.91309007	TRUE
GB13543	3	0	NA	NA	FALSE
GB13544	758	343	1.143989272	0.279471764	FALSE
GB13545	11	6	0.874469118	0.00995161	FALSE
GB13546	1645	835	0.978239481	0.113721973	FALSE
GB13547	66	23	1.520832163	0.656314655	TRUE
GB13548	1268	706	0.844814657	-0.019702851	FALSE
GB13549	NA	NA	NA	NA	FALSE
GB13550	1263	771	0.712051874	-0.152465634	FALSE
GB13551	12989	6096	1.091355557	0.226838048	FALSE
GB13552	1137	622	0.870245769	0.00572826	FALSE
GB13553	44	29	0.601450624	-0.263066885	FALSE
GB13554	190	99	0.940498988	0.07598148	FALSE
GB13555	462	252	0.874469118	0.00995161	FALSE
GB13556	1149	686	0.744098316	-0.120419192	FALSE
GB13557	768	423	0.860448648	-0.004068861	FALSE
GB13558	2	1	1	0.135482492	FALSE
GB13559	108	68	0.667424661	-0.197092847	FALSE

GB13560	2	0	NA	NA	FALSE
GB13561	313	175	0.838807735	-0.025709773	FALSE
GB13562	363	223	0.702925838	-0.16159167	FALSE
GB13563	846	455	0.894791118	0.03027361	FALSE
GB13564	76	44	0.788495895	-0.076021614	FALSE
GB13565	20	11	0.862496476	-0.002021032	FALSE
GB13566	1	1	0	-0.864517508	TRUE
GB13567	1217	716	0.765297675	-0.099219833	FALSE
GB13568	60	32	0.906890596	0.042373087	FALSE
GB13569	473	279	0.761575062	-0.102942447	FALSE
GB13570	800	479	0.739974344	-0.124543164	FALSE
GB13571	1	0	NA	NA	FALSE
GB13572	445	207	1.104174568	0.23965706	FALSE
GB13573	1994	981	1.023340368	0.15882286	FALSE
GB13574	115	75	0.61667136	-0.247846148	FALSE
GB13575	386	207	0.89897008	0.034452571	FALSE
GB13576	867	360	1.268035087	0.403517579	FALSE
GB13577	1707	800	1.093391153	0.228873645	FALSE
GB13578	344	208	0.725825037	-0.138692472	FALSE
GB13579	76	30	1.341036918	0.47651941	FALSE
GB13580	243	104	1.224372785	0.359855277	FALSE
GB13581	18	5	1.847996907	0.983479398	TRUE
GB13582	NA	NA	NA	NA	FALSE
GB13583	1830	1179	0.63427993	-0.230237578	FALSE
GB13584	NA	NA	NA	NA	FALSE
GB13585	0	1	NA	NA	FALSE
GB13586	187	72	1.376969458	0.51245195	TRUE
GB13587	NA	NA	NA	NA	FALSE
GB13588	216	130	0.732519689	-0.131997819	FALSE
GB13589	335	254	0.399332599	-0.46518491	FALSE
GB13590	56	28	1	0.135482492	FALSE
GB13591	375	140	1.421463768	0.55694626	TRUE
GB13592	1776	823	1.109667246	0.245149738	FALSE
GB13593	NA	NA	NA	NA	FALSE
GB13594	11	6	0.874469118	0.00995161	FALSE
GB13595	2238	1393	0.684014778	-0.18050273	FALSE
GB13596	34392	23523	0.54800095	-0.316516559	FALSE
GB13597	413	197	1.067946152	0.203428644	FALSE
GB13598	2698	1142	1.240327698	0.375810189	FALSE
GB13599	1291	883	0.548003658	-0.316513851	FALSE
GB13600	828	466	0.829300813	-0.035216695	FALSE
GB13601	3302	1178	1.487000581	0.622483073	TRUE
GB13602	6802	3682	0.885469379	0.020951871	FALSE
GB13603	93	43	1.112894056	0.248376548	FALSE

GB13604	727	455	0.676088819	-0.188428689	FALSE
GB13605	2718	1244	1.127558971	0.263041462	FALSE
GB13606	1584	591	1.4223423	0.557824792	TRUE
GB13607	217	132	0.717157113	-0.147360395	FALSE
GB13608	243	150	0.695993813	-0.168523695	FALSE
GB13609	11	9	0.289506617	-0.575010891	TRUE
GB13610	11423	6219	0.87718707	0.012669562	FALSE
GB13611	4	1	2	1.135482492	TRUE
GB13612	708	401	0.820147124	-0.044370385	FALSE
GB13613	3440594	1571359	1.130644837	0.266127329	FALSE
GB13614	62	34	0.866733469	0.002215961	FALSE
GB13615	3990	1301	1.616767784	0.752250276	TRUE
GB13616	1028	639	0.685952428	-0.17856508	FALSE
GB13617	3393	1576	1.106293895	0.241776387	FALSE
GB13618	294	141	1.060120992	0.195603484	FALSE
GB13619	1943	849	1.194449442	0.329931934	FALSE
GB13620	472	195	1.275312736	0.410795227	FALSE
GB13621	5781	2728	1.083475428	0.21895792	FALSE
GB13622	539	423	0.34962761	-0.514889899	TRUE
GB13623	7	3	1.222392421	0.357874913	FALSE
GB13624	75	16	2.22881869	1.364301182	TRUE
GB13625	59	28	1.075288127	0.210770619	FALSE
GB13626	26354	16049	0.715538543	-0.148978965	FALSE
GB13627	610	362	0.752819545	-0.111697963	FALSE
GB13628	NA	NA	NA	NA	FALSE
GB13629	382	233	0.713242683	-0.151274825	FALSE
GB13630	162	100	0.695993813	-0.168523695	FALSE
GB13631	206	172	0.260235772	-0.604281736	TRUE
GB13632	NA	NA	NA	NA	FALSE
GB13633	NA	NA	NA	NA	FALSE
GB13634	6	6	0	-0.864517508	TRUE
GB13635	147	74	0.990218979	0.125701471	FALSE
GB13636	35	24	0.544320516	-0.320196992	FALSE
GB13637	1497	753	0.991352451	0.126834943	FALSE
GB13638	105	72	0.544320516	-0.320196992	FALSE
GB13639	592	332	0.834413934	-0.030103574	FALSE
GB13640	34	7	2.280107919	1.415590411	TRUE
GB13641	909	656	0.47058448	-0.393933029	FALSE
GB13642	NA	NA	NA	NA	FALSE
GB13643	208	142	0.550692599	-0.31382491	FALSE
GB13644	79	42	0.911463325	0.046945817	FALSE
GB13645	23	9	1.353636955	0.489119446	FALSE
GB13646	2243	1217	0.882100452	0.017582944	FALSE
GB13647	114	68	0.745427173	-0.119090335	FALSE

GB13648	459	381	0.268703156	-0.595814352	TRUE
GB13649	10	1	3.321928095	2.457410587	TRUE
GB13650	NA	NA	NA	NA	FALSE
GB13651	555	353	0.652819588	-0.21169792	FALSE
GB13652	NA	NA	NA	NA	FALSE
GB13653	1	1	0	-0.864517508	TRUE
GB13654	2129	1052	1.017041245	0.152523737	FALSE
GB13655	172	104	0.725825037	-0.138692472	FALSE
GB13656	1935	979	0.982952801	0.118435293	FALSE
GB13657	1983	1026	0.950653947	0.086136438	FALSE
GB13658	37889	17293	1.131590893	0.267073385	FALSE
GB13659	836	584	0.517534573	-0.346982935	FALSE
GB13660	110	73	0.591535155	-0.272982354	FALSE
GB13661	289	193	0.582468645	-0.282048863	FALSE
GB13662	925	596	0.634141035	-0.230376473	FALSE
GB13663	15	7	1.099535674	0.235018165	FALSE
GB13664	NA	NA	NA	NA	FALSE
GB13665	2322	1378	0.752792084	-0.111725424	FALSE
GB13666	502	267	0.910847622	0.046330114	FALSE
GB13667	388	218	0.831728517	-0.032788991	FALSE
GB13668	1520	846	0.845341755	-0.019175753	FALSE
GB13669	685	424	0.692039723	-0.172477785	FALSE
GB13670	55	18	1.611434712	0.746917204	TRUE
GB13671	299	125	1.25821739	0.393699881	FALSE
GB13672	2	1	1	0.135482492	FALSE
GB13673	162	96	0.754887502	-0.109630006	FALSE
GB13674	853	598	0.512400257	-0.352117251	FALSE
GB13675	1036	502	1.045264734	0.180747225	FALSE
GB13676	73	66	0.14543044	-0.719087069	TRUE
GB13677	1099	641	0.777795124	-0.086722384	FALSE
GB13678	913	409	1.158514017	0.293996509	FALSE
GB13679	725	411	0.818842601	-0.045674907	FALSE
GB13680	24786	16293	0.605273197	-0.259244311	FALSE
GB13681	1103	405	1.445438978	0.580921469	TRUE
GB13682	5	4	0.321928095	-0.542589413	TRUE
GB13683	510	281	0.859927117	-0.004590392	FALSE
GB13684	424	240	0.821029859	-0.043487649	FALSE
GB13685	913	516	0.823243795	-0.041273714	FALSE
GB13686	2305	1280	0.84862294	-0.015894568	FALSE
GB13687	12	4	1.584962501	0.720444992	TRUE
GB13688	38547	19180	1.007015863	0.142498355	FALSE
GB13689	6434	5466	0.235230476	-0.629287032	TRUE
GB13690	109	86	0.34191957	-0.522597938	TRUE
GB13691	NA	NA	NA	NA	FALSE

GB13692	7550	5051	0.579907603	-0.284609906	FALSE
GB13693	551	286	0.946037172	0.081519663	FALSE
GB13694	0	1	NA	NA	FALSE
GB13695	2276	1400	0.70107373	-0.163443778	FALSE
GB13696	565	328	0.784555053	-0.079962456	FALSE
GB13697	190	122	0.639118271	-0.225399238	FALSE
GB13698	206	86	1.260235772	0.395718264	FALSE
GB13699	2373	1471	0.689914854	-0.174602654	FALSE
GB13700	253	123	1.040479069	0.175961561	FALSE
GB13701	313	179	0.80620307	-0.058314439	FALSE
GB13702	1267	792	0.677844189	-0.186673319	FALSE
GB13703	129	81	0.671377253	-0.193140256	FALSE
GB13704	1181	704	0.746361631	-0.118155878	FALSE
GB13705	265	160	0.727920455	-0.136597054	FALSE
GB13706	381	215	0.825454338	-0.03906317	FALSE
GB13707	5	4	0.321928095	-0.542589413	TRUE
GB13708	1129	671	0.750660815	-0.113856694	FALSE
GB13709	1430	743	0.944581031	0.080063523	FALSE
GB13710	79	44	0.84434913	-0.020168379	FALSE
GB13711	407	210	0.954639467	0.090121958	FALSE
GB13712	98	46	1.091147888	0.22663038	FALSE
GB13713	5	0	NA	NA	FALSE
GB13714	362	189	0.937603463	0.073085955	FALSE
GB13715	814	541	0.5894002	-0.275117308	FALSE
GB13716	1012	607	0.737440868	-0.12707664	FALSE
GB13717	1009	516	0.967483204	0.102965695	FALSE
GB13718	510	292	0.804528878	-0.05998863	FALSE
GB13719	200	156	0.358453971	-0.506063537	TRUE
GB13720	18	3	2.584962501	1.720444992	TRUE
GB13721	1786	1169	0.611457151	-0.253060358	FALSE
GB13722	2927	902	1.698223407	0.833705899	TRUE
GB13723	206	54	1.931613025	1.067095517	TRUE
GB13724	3827	2057	0.895672107	0.031154599	FALSE
GB13725	852	471	0.855126371	-0.009391138	FALSE
GB13726	122	68	0.843274496	-0.021243012	FALSE
GB13727	1210	624	0.955389113	0.090871605	FALSE
GB13728	1505	903	0.736965594	-0.127551914	FALSE
GB13729	704	402	0.808379927	-0.056137581	FALSE
GB13730	6915	3793	0.86638988	0.001872371	FALSE
GB13731	16187	10805	0.583136558	-0.28138095	FALSE
GB13732	658	314	1.067323025	0.202805517	FALSE
GB13733	630	356	0.823474587	-0.041042921	FALSE
GB13734	170	87	0.96644744	0.101929932	FALSE
GB13735	1163	562	1.049209061	0.184691553	FALSE

GB13736	345	171	1.012600037	0.148082528	FALSE
GB13737	35	16	1.129283017	0.264765509	FALSE
GB13738	91	48	0.922832139	0.058314631	FALSE
GB13739	623	377	0.72466764	-0.139849869	FALSE
GB13740	30	14	1.099535674	0.235018165	FALSE
GB13741	36501	24159	0.59537525	-0.269142258	FALSE
GB13742	NA	NA	NA	NA	FALSE
GB13743	1288	777	0.72914609	-0.135371419	FALSE
GB13744	627	375	0.741574847	-0.122942661	FALSE
GB13745	12	8	0.584962501	-0.279555008	FALSE
GB13746	566	320	0.822730148	-0.04178736	FALSE
GB13747	2081	1108	0.909319084	0.044801575	FALSE
GB13748	1394	531	1.392446795	0.527929287	TRUE
GB13749	711	344	1.047440995	0.182923487	FALSE
GB13750	421	185	1.186294963	0.321777454	FALSE
GB13751	531	288	0.882643049	0.018125541	FALSE
GB13752	43	40	0.10433666	-0.760180849	TRUE
GB13753	103	59	0.803857478	-0.060660031	FALSE
GB13754	2941	1166	1.334738996	0.470221487	FALSE
GB13755	0	2	NA	NA	FALSE
GB13756	2	0	NA	NA	FALSE
GB13757	959	592	0.695933639	-0.168583869	FALSE
GB13758	411	219	0.908207524	0.043690016	FALSE
GB13759	249	132	0.915607813	0.051090304	FALSE
GB13760	1230	838	0.553636167	-0.310881342	FALSE
GB13761	75	33	1.184424571	0.319907063	FALSE
GB13762	742	341	1.121647448	0.257129939	FALSE
GB13763	361	296	0.286401661	-0.578115847	TRUE
GB13764	520	405	0.360589715	-0.503927793	TRUE
GB13765	333	249	0.419376435	-0.445141073	FALSE
GB13766	1277	796	0.681918189	-0.182599319	FALSE
GB13767	705	372	0.922320636	0.057803128	FALSE
GB13768	485	256	0.921840937	0.057323429	FALSE
GB13769	1413	957	0.562170636	-0.302346872	FALSE
GB13770	2740	2065	0.408034112	-0.456483397	FALSE
GB13771	674	351	0.941277561	0.076760053	FALSE
GB13772	12982	7567	0.778719311	-0.085798197	FALSE
GB13773	257	160	0.683696454	-0.180821054	FALSE
GB13774	346	177	0.967022678	0.102505169	FALSE
GB13775	312	143	1.125530882	0.261013374	FALSE
GB13776	1634	1070	0.610797187	-0.253720321	FALSE
GB13777	2810	1649	0.768978732	-0.095538777	FALSE
GB13778	7287	2881	1.33875533	0.474237822	FALSE
GB13779	2308	1151	1.003755391	0.139237882	FALSE

GB13780	2351	1301	0.853653577	-0.010863931	FALSE
GB13781	NA	NA	NA	NA	FALSE
GB13782	425	241	0.818429695	-0.046087814	FALSE
GB13783	437	245	0.83485153	-0.029665978	FALSE
GB13784	5	5	0	-0.864517508	TRUE
GB13785	NA	NA	NA	NA	FALSE
GB13786	626	426	0.555309227	-0.309208282	FALSE
GB13787	15	14	0.099535674	-0.764981835	TRUE
GB13788	79	26	1.60334103	0.738823522	TRUE
GB13789	NA	NA	NA	NA	FALSE
GB13790	340	254	0.420706249	-0.443811259	FALSE
GB13791	20	11	0.862496476	-0.002021032	FALSE
GB13792	1781	935	0.929649246	0.065131738	FALSE
GB13793	108	42	1.362570079	0.498052571	FALSE
GB13794	1	2	-1	-1.864517508	TRUE
GB13795	223	109	1.032715575	0.168198067	FALSE
GB13796	434	213	1.026841612	0.162324104	FALSE
GB13797	63	40	0.655351829	-0.20916568	FALSE
GB13798	2095	1155	0.859057392	-0.005460116	FALSE
GB13799	68	30	1.180572246	0.316054737	FALSE
GB13800	137	66	1.053637964	0.189120455	FALSE
GB13801	108	64	0.754887502	-0.109630006	FALSE
GB13802	790	366	1.110009005	0.245491496	FALSE
GB13803	1	0	NA	NA	FALSE
GB13804	126	75	0.748461233	-0.116056275	FALSE
GB13805	2263	1392	0.701077373	-0.163440135	FALSE
GB13806	242	142	0.769116118	-0.095401391	FALSE
GB13807	118	40	1.560714954	0.696197446	TRUE
GB13808	152	107	0.506460527	-0.358056981	FALSE
GB13809	535	318	0.750512126	-0.114005382	FALSE
GB13810	486	229	1.085608716	0.221091207	FALSE
GB13811	2	1	1	0.135482492	FALSE
GB13812	137	73	0.908207524	0.043690016	FALSE
GB13813	118	79	0.578862301	-0.285655207	FALSE
GB13814	2	0	NA	NA	FALSE
GB13815	3897	2454	0.667228683	-0.197288826	FALSE
GB13816	1322	677	0.965494438	0.10097693	FALSE
GB13817	2926	1499	0.964929386	0.100411878	FALSE
GB13818	355	200	0.827819025	-0.036698484	FALSE
GB13819	1477	733	1.010784723	0.146267214	FALSE
GB13820	565	298	0.922938537	0.058421029	FALSE
GB13821	29545	17631	0.744799695	-0.119717814	FALSE
GB13822	251	111	1.177127688	0.312610179	FALSE
GB13823	404	207	0.964724525	0.100207017	FALSE

GB13824	890	446	0.996761626	0.132244118	FALSE
GB13825	521	297	0.810820441	-0.053697067	FALSE
GB13826	2182	1452	0.587609648	-0.27690786	FALSE
GB13827	2239	1237	0.856009028	-0.00850848	FALSE
GB13828	0	1	NA	NA	FALSE
GB13829	11	5	1.137503524	0.272986015	FALSE
GB13830	894	443	1.012968133	0.148450624	FALSE
GB13831	8	0	NA	NA	FALSE
GB13832	1366	857	0.672590374	-0.191927134	FALSE
GB13833	233	129	0.852958889	-0.011558619	FALSE
GB13834	78	12	2.700439718	1.83592221	TRUE
GB13835	5	3	0.736965594	-0.127551914	FALSE
GB13836	99	73	0.439532061	-0.424985447	FALSE
GB13837	860	502	0.776649296	-0.087868213	FALSE
GB13838	103	39	1.401098308	0.5365808	TRUE
GB13839	459	240	0.935459748	0.070942239	FALSE
GB13840	7221	3408	1.083273307	0.218755799	FALSE
GB13841	1753	988	0.827243049	-0.037274459	FALSE
GB13842	2215	1327	0.739138328	-0.12537918	FALSE
GB13843	389	225	0.789845154	-0.074672355	FALSE
GB13844	48	22	1.125530882	0.261013374	FALSE
GB13845	135	62	1.122619287	0.258101778	FALSE
GB13846	556	336	0.72662365	-0.137893858	FALSE
GB13847	7	2	1.807354922	0.942837414	TRUE
GB13848	21	15	0.485426827	-0.379090681	FALSE
GB13849	473	252	0.90841645	0.043898942	FALSE
GB13850	981	482	1.02521999	0.160702482	FALSE
GB13851	NA	NA	NA	NA	FALSE
GB13852	4899	2720	0.84888064	-0.015636868	FALSE
GB13853	3553	1753	1.019211693	0.154694184	FALSE
GB13854	NA	NA	NA	NA	FALSE
GB13855	946	556	0.766755301	-0.097762208	FALSE
GB13856	NA	NA	NA	NA	FALSE
GB13857	470	225	1.062735755	0.198218247	FALSE
GB13858	1573	688	1.193038201	0.328520692	FALSE
GB13859	1502	920	0.707179047	-0.157338462	FALSE
GB13860	2	4	-1	-1.864517508	TRUE
GB13861	2353	1111	1.082642504	0.218124996	FALSE
GB13862	1073	570	0.912616252	0.048098743	FALSE
GB13863	13	6	1.115477217	0.250959709	FALSE
GB13864	1215	707	0.781174194	-0.083343315	FALSE
GB13865	3302	1604	1.041665978	0.17714847	FALSE
GB13866	33	31	0.090197809	-0.774319699	TRUE
GB13867	484	290	0.738954147	-0.125563361	FALSE

GB13868	768	512	0.584962501	-0.279555008	FALSE
GB13869	5908	3320	0.831486585	-0.033030924	FALSE
GB13870	198	175	0.178145508	-0.686372	TRUE
GB13871	335	187	0.841122825	-0.023394683	FALSE
GB13872	116	52	1.157541277	0.293023769	FALSE
GB13873	798	391	1.029220139	0.164702631	FALSE
GB13874	280	95	1.559427409	0.6949099	TRUE
GB13875	560	352	0.669851398	-0.19466611	FALSE
GB13876	1	1	0	-0.864517508	TRUE
GB13877	38	24	0.662965013	-0.201552496	FALSE
GB13878	6	1	2.584962501	1.720444992	TRUE
GB13879	925	445	1.05564803	0.191130521	FALSE
GB13880	6847	3268	1.067064029	0.202546521	FALSE
GB13881	356	211	0.754634242	-0.109883266	FALSE
GB13882	3684	1616	1.188845863	0.324328355	FALSE
GB13883	138	57	1.275634443	0.411116934	FALSE
GB13884	9	3	1.584962501	0.720444992	TRUE
GB13885	43	36	0.256339753	-0.608177755	TRUE
GB13886	795	450	0.821029859	-0.043487649	FALSE
GB13887	231	120	0.944858446	0.080340937	FALSE
GB13888	5215	3461	0.591478311	-0.273039197	FALSE
GB13889	36	13	1.469485283	0.604967775	TRUE
GB13890	1457	837	0.79970135	-0.064816159	FALSE
GB13891	2143	1006	1.091001545	0.226484037	FALSE
GB13892	1316	581	1.17954942	0.315031912	FALSE
GB13893	4867	3115	0.643800611	-0.220716897	FALSE
GB13894	66	39	0.7589919	-0.105525608	FALSE
GB13895	456	271	0.750740973	-0.113776536	FALSE
GB13896	298	212	0.491248066	-0.373269442	FALSE
GB13897	25	13	0.943416472	0.078898963	FALSE
GB13898	900	537	0.745002913	-0.119514595	FALSE
GB13899	1342	706	0.926644583	0.062127075	FALSE
GB13900	2514	1285	0.96821629	0.103698782	FALSE
GB13901	444	203	1.129079949	0.264562441	FALSE
GB13902	329	180	0.870090677	0.005573169	FALSE
GB13903	69	25	1.464668267	0.600150759	TRUE
GB13904	122	76	0.682809824	-0.181707684	FALSE
GB13905	NA	NA	NA	NA	FALSE
GB13906	3894	2397	0.700022975	-0.164494533	FALSE
GB13907	463	379	0.288814345	-0.575703163	TRUE
GB13908	51	32	0.672425342	-0.192092166	FALSE
GB13909	22	6	1.874469118	1.00995161	TRUE
GB13910	2513	1476	0.76771795	-0.096799559	FALSE
GB13911	1	1	0	-0.864517508	TRUE

GB13912	775	424	0.870132046	0.005614537	FALSE
GB13913	957	556	0.783434042	-0.081083467	FALSE
GB13914	3	1	1.584962501	0.720444992	TRUE
GB13915	NA	NA	NA	NA	FALSE
GB13916	193	76	1.344529524	0.480012015	FALSE
GB13917	7729	5076	0.606589697	-0.257927811	FALSE
GB13918	114	54	1.078002512	0.213485004	FALSE
GB13919	1105	538	1.038368292	0.173850783	FALSE
GB13920	985	628	0.649359165	-0.215158343	FALSE
GB13921	755	306	1.302944992	0.438427483	FALSE
GB13922	173	88	0.975196609	0.110679101	FALSE
GB13923	224	100	1.163498732	0.298981224	FALSE
GB13924	4777	3671	0.379931762	-0.484585746	FALSE
GB13925	NA	NA	NA	NA	FALSE
GB13926	93	40	1.217230716	0.352713208	FALSE
GB13927	831	443	0.907541778	0.04302427	FALSE
GB13928	2009	1205	0.737444418	-0.127073091	FALSE
GB13929	269	151	0.833057623	-0.031459885	FALSE
GB13930	2293	1033	1.150396101	0.285878592	FALSE
GB13931	991	378	1.390498823	0.525981315	TRUE
GB13932	945	562	0.749744199	-0.114773309	FALSE
GB13933	607	311	0.964781936	0.100264428	FALSE
GB13934	548	370	0.566650622	-0.297866886	FALSE
GB13935	110	59	0.898716664	0.034199156	FALSE
GB13936	349	263	0.408164237	-0.456353271	FALSE
GB13937	89	31	1.521537121	0.657019612	TRUE
GB13938	2	0	NA	NA	FALSE
GB13939	19	9	1.078002512	0.213485004	FALSE
GB13940	305	154	0.985878892	0.121361383	FALSE
GB13941	112	62	0.853158612	-0.011358897	FALSE
GB13942	923	590	0.645615693	-0.218901815	FALSE
GB13943	NA	NA	NA	NA	FALSE
GB13944	1008	717	0.491450615	-0.373066894	FALSE
GB13945	465	332	0.486047475	-0.378470034	FALSE
GB13946	169	81	1.061029433	0.196511925	FALSE
GB13947	1175	668	0.814740749	-0.049776759	FALSE
GB13948	25	10	1.321928095	0.457410587	FALSE
GB13949	34	13	1.387023123	0.522505615	TRUE
GB13950	3	5	-0.736965594	-1.601483102	TRUE
GB13951	437	211	1.050390281	0.185872772	FALSE
GB13952	41	50	-0.286304185	-1.150821693	TRUE
GB13953	469	305	0.62077868	-0.243738828	FALSE
GB13954	2699	1299	1.055023546	0.190506038	FALSE
GB13955	783	481	0.702975414	-0.161542095	FALSE

GB13956	3519	1986	0.825299891	-0.039217617	FALSE
GB13957	154	78	0.981384322	0.116866814	FALSE
GB13958	137	67	1.031942893	0.167425384	FALSE
GB13959	160	81	0.982078092	0.117560584	FALSE
GB13960	472	309	0.611180021	-0.253337487	FALSE
GB13961	80	20	2	1.135482492	TRUE
GB13962	106	44	1.268488836	0.403971328	FALSE
GB13963	NA	NA	NA	NA	FALSE
GB13964	NA	NA	NA	NA	FALSE
GB13965	2287	1333	0.778779585	-0.085737923	FALSE
GB13966	195334	101135	0.949660728	0.085143219	FALSE
GB13967	19843	10500	0.918240831	0.053723322	FALSE
GB13968	624	301	1.051782542	0.187265034	FALSE
GB13969	2953	1565	0.916018699	0.051501191	FALSE
GB13970	1161	742	0.64587688	-0.218640628	FALSE
GB13971	451	262	0.783560622	-0.080956887	FALSE
GB13972	50	17	1.556393349	0.69187584	TRUE
GB13973	456	163	1.48416186	0.619644352	TRUE
GB13974	720	342	1.074000581	0.209483073	FALSE
GB13975	12825	9264	0.469251667	-0.395265841	FALSE
GB13976	151	74	1.028951374	0.164433865	FALSE
GB13977	406	246	0.722821412	-0.141696096	FALSE
GB13978	NA	NA	NA	NA	FALSE
GB13979	11	2	2.459431619	1.59491411	TRUE
GB13980	660	388	0.766409372	-0.098108136	FALSE
GB13981	125	61	1.035046947	0.170529439	FALSE
GB13982	17336	9318	0.895678823	0.031161315	FALSE
GB13983	3	7	-1.222392421	-2.08690993	TRUE
GB13984	1	0	NA	NA	FALSE
GB13985	794	476	0.738177434	-0.126340074	FALSE
GB13986	370	219	0.756594401	-0.107923107	FALSE
GB13987	900	553	0.702645521	-0.161871987	FALSE
GB13988	347	202	0.78058037	-0.083937139	FALSE
GB13989	1588	1064	0.577712762	-0.286804747	FALSE
GB13990	1717	964	0.832784988	-0.031732521	FALSE
GB13991	8	6	0.415037499	-0.449480009	FALSE
GB13992	558	401	0.476662885	-0.387854623	FALSE
GB13993	631	443	0.510333306	-0.354184202	FALSE
GB13994	327	255	0.358793389	-0.50572412	TRUE
GB13995	2004	1110	0.852322832	-0.012194676	FALSE
GB13996	672	390	0.784987109	-0.079530399	FALSE
GB13997	2211	1230	0.84604071	-0.018476799	FALSE
GB13998	9	1	3.169925001	2.305407493	TRUE
GB13999	1544	1544	0	-0.864517508	TRUE

GB14000	1562	1202	0.377957557	-0.486559951	FALSE
GB14001	337	135	1.319789184	0.455271676	FALSE
GB14002	424	231	0.876171413	0.011653905	FALSE
GB14003	886	460	0.945672838	0.081155329	FALSE
GB14004	31	13	1.253756592	0.389239084	FALSE
GB14005	765	337	1.182711156	0.318193648	FALSE
GB14006	143	70	1.03058832	0.166070812	FALSE
GB14007	262	122	1.102685664	0.238168156	FALSE
GB14008	4	0	NA	NA	FALSE
GB14009	212	98	1.11321061	0.248693102	FALSE
GB14010	41	24	0.772589504	-0.091928004	FALSE
GB14011	3	1	1.584962501	0.720444992	TRUE
GB14012	5849	3732	0.648241003	-0.216276505	FALSE
GB14013	323	142	1.185643235	0.321125727	FALSE
GB14014	172	57	1.593374741	0.728857232	TRUE
GB14015	0	1	NA	NA	FALSE
GB14016	1032	564	0.871675903	0.007158395	FALSE
GB14017	832	523	0.669772582	-0.194744926	FALSE
GB14018	0	1	NA	NA	FALSE
GB14019	NA	NA	NA	NA	FALSE
GB14020	NA	NA	NA	NA	FALSE
GB14021	NA	NA	NA	NA	FALSE
GB14022	42	30	0.485426827	-0.379090681	FALSE
GB14023	894	493	0.858687185	-0.005830324	FALSE
GB14024	1065	454	1.230089228	0.365571719	FALSE
GB14025	356	229	0.636529643	-0.227987865	FALSE
GB14026	2723	1448	0.91113538	0.046617872	FALSE
GB14027	1066	480	1.151101127	0.286583619	FALSE
GB14028	993	686	0.533585141	-0.330932367	FALSE
GB14029	157	82	0.937068744	0.072551236	FALSE
GB14030	540	241	1.163926261	0.299408752	FALSE
GB14031	772	513	0.589642022	-0.274875487	FALSE
GB14032	2994	1268	1.239519476	0.375001968	FALSE
GB14033	83	42	0.982722009	0.1182045	FALSE
GB14034	3940	2790	0.497930508	-0.366587001	FALSE
GB14035	1	1	0	-0.864517508	TRUE
GB14036	NA	NA	NA	NA	FALSE
GB14037	9754	4410	1.145215317	0.280697808	FALSE
GB14038	6	8	-0.415037499	-1.279555008	TRUE
GB14039	225	125	0.847996907	-0.016520602	FALSE
GB14040	1164	658	0.822931569	-0.041585939	FALSE
GB14041	NA	NA	NA	NA	FALSE
GB14042	598	392	0.60929183	-0.255225678	FALSE
GB14043	418	227	0.880810645	0.016293136	FALSE

GB14044	1216	743	0.710709113	-0.153808395	FALSE
GB14045	1	0	NA	NA	FALSE
GB14046	NA	NA	NA	NA	FALSE
GB14047	1123	381	1.559495025	0.694977517	TRUE
GB14048	25	8	1.64385619	0.779338681	TRUE
GB14049	7	2	1.807354922	0.942837414	TRUE
GB14050	606	338	0.842294547	-0.022222961	FALSE
GB14051	6371	5012	0.346133423	-0.518384086	TRUE
GB14052	151	64	1.238404739	0.373887231	FALSE
GB14053	259	133	0.961525852	0.097008344	FALSE
GB14054	28	14	1	0.135482492	FALSE
GB14055	420	296	0.504792152	-0.359725356	FALSE
GB14056	3699	2104	0.814000596	-0.050516912	FALSE
GB14057	321	212	0.598509033	-0.266008476	FALSE
GB14058	2	1	1	0.135482492	FALSE
GB14059	7551	4896	0.625064159	-0.239453349	FALSE
GB14060	2099	1117	0.910072981	0.045555472	FALSE
GB14061	791	415	0.930566358	0.06604885	FALSE
GB14062	1182	524	1.173591319	0.30907381	FALSE
GB14063	444	202	1.136204384	0.271686875	FALSE
GB14064	994	565	0.814994984	-0.049522524	FALSE
GB14065	353	173	1.028896146	0.164378637	FALSE
GB14066	9	3	1.584962501	0.720444992	TRUE
GB14067	590	328	0.84701914	-0.017498369	FALSE
GB14068	5026	2613	0.943703547	0.079186038	FALSE
GB14069	3206	2071	0.630446872	-0.234070636	FALSE
GB14070	NA	NA	NA	NA	FALSE
GB14071	27	17	0.667424661	-0.197092847	FALSE
GB14072	533	323	0.722601368	-0.14191614	FALSE
GB14073	181	87	1.056902391	0.192384883	FALSE
GB14074	126	52	1.276840205	0.412322697	FALSE
GB14075	NA	NA	NA	NA	FALSE
GB14076	1146	659	0.798256674	-0.066260835	FALSE
GB14077	23	27	-0.231325546	-1.095843054	TRUE
GB14078	136	65	1.065095028	0.20057752	FALSE
GB14079	NA	NA	NA	NA	FALSE
GB14080	4	0	NA	NA	FALSE
GB14081	158	57	1.470890734	0.606373226	TRUE
GB14082	1519	877	0.792473122	-0.072044386	FALSE
GB14083	49	24	1.029747343	0.165229835	FALSE
GB14084	1655	800	1.048759312	0.184241804	FALSE
GB14085	2	6	-1.584962501	-2.449480009	TRUE
GB14086	NA	NA	NA	NA	FALSE
GB14087	1	1	0	-0.864517508	TRUE

GB14088	291	187	0.637980883	-0.226536625	FALSE
GB14089	64	76	-0.247927513	-1.112445022	TRUE
GB14090	1	1	0	-0.864517508	TRUE
GB14091	1959	1370	0.515941504	-0.348576004	FALSE
GB14092	47	17	1.46712601	0.602608502	TRUE
GB14093	864	471	0.875304253	0.010786744	FALSE
GB14094	12	7	0.777607579	-0.08690993	FALSE
GB14095	4899	2751	0.832531152	-0.031986356	FALSE
GB14096	125	34	1.878321443	1.013803935	TRUE
GB14097	615	428	0.522975614	-0.341541895	FALSE
GB14098	529	429	0.302290075	-0.562227434	TRUE
GB14099	2	5	-1.321928095	-2.186445603	TRUE
GB14100	824	569	0.534215685	-0.330301823	FALSE
GB14101	1071	638	0.747330151	-0.117187357	FALSE
GB14102	86	33	1.381870635	0.517353127	TRUE
GB14103	259	147	0.817135943	-0.047381565	FALSE
GB14104	11723	5669	1.048175639	0.18365813	FALSE
GB14105	9610	3510	1.4530654	0.588547892	TRUE
GB14106	528	244	1.113656782	0.249139273	FALSE
GB14107	33	23	0.520832163	-0.343685345	FALSE
GB14108	3969	2386	0.734181519	-0.130335989	FALSE
GB14109	3	0	NA	NA	FALSE
GB14110	1186	585	1.01959548	0.155077972	FALSE
GB14111	138	97	0.508611615	-0.355905894	FALSE
GB14112	1234	623	0.986038326	0.121520818	FALSE
GB14113	14227	8136	0.806239893	-0.058277615	FALSE
GB14114	12532	8813	0.507911565	-0.356605944	FALSE
GB14115	2471	1327	0.89692664	0.032409132	FALSE
GB14116	518	313	0.726789441	-0.137728068	FALSE
GB14117	631	368	0.777934239	-0.086583269	FALSE
GB14118	1022	522	0.969273484	0.104755976	FALSE
GB14119	1	0	NA	NA	FALSE
GB14120	276	186	0.569365646	-0.295151863	FALSE
GB14121	559	437	0.355215003	-0.509302505	TRUE
GB14122	311	192	0.695808269	-0.168709239	FALSE
GB14123	0	2	NA	NA	FALSE
GB14124	649	406	0.676738751	-0.187778758	FALSE
GB14125	110	45	1.289506617	0.424989109	FALSE
GB14126	704	203	1.794095701	0.929578193	TRUE
GB14127	9924	6615	0.585180579	-0.27933693	FALSE
GB14128	3954	1954	1.016882404	0.152364895	FALSE
GB14129	38	18	1.078002512	0.213485004	FALSE
GB14130	688	353	0.962740381	0.098222873	FALSE
GB14131	NA	NA	NA	NA	FALSE

GB14132	1766	858	1.04143579	0.176918282	FALSE
GB14133	NA	NA	NA	NA	FALSE
GB14134	1	3	-1.584962501	-2.449480009	TRUE
GB14135	1845	1045	0.820117874	-0.044399634	FALSE
GB14136	3	3	0	-0.864517508	TRUE
GB14137	1820	974	0.901944773	0.037427265	FALSE
GB14138	4424	2672	0.727431378	-0.137086131	FALSE
GB14139	1015	633	0.681202323	-0.183315186	FALSE
GB14140	636	608	0.064955442	-0.799562066	TRUE
GB14141	431	187	1.204649599	0.340132091	FALSE
GB14142	828	486	0.768674454	-0.095843054	FALSE
GB14143	103	59	0.803857478	-0.060660031	FALSE
GB14144	2137	1206	0.825357001	-0.039160508	FALSE
GB14145	1125	512	1.135709286	0.271191778	FALSE
GB14146	970	555	0.805496976	-0.059020532	FALSE
GB14147	31	8	1.95419631	1.089678802	TRUE
GB14148	715	325	1.137503524	0.272986015	FALSE
GB14149	434	173	1.326923005	0.462405496	FALSE
GB14150	1771	1113	0.670110619	-0.194406889	FALSE
GB14151	24	14	0.777607579	-0.08690993	FALSE
GB14152	8151	4332	0.911943823	0.047426315	FALSE
GB14153	485	306	0.664453094	-0.200064414	FALSE
GB14154	5737	2963	0.953237892	0.088720383	FALSE
GB14155	NA	NA	NA	NA	FALSE
GB14156	207	121	0.77462372	-0.089893788	FALSE
GB14157	335	175	0.936806174	0.072288665	FALSE
GB14158	596	449	0.408596886	-0.455920623	FALSE
GB14159	80	69	0.213403638	-0.65111387	TRUE
GB14160	132	72	0.874469118	0.00995161	FALSE
GB14161	1152	460	1.32443495	0.459917442	FALSE
GB14162	422	211	1	0.135482492	FALSE
GB14163	1430	703	1.024418553	0.159901044	FALSE
GB14164	94	54	0.79970135	-0.064816159	FALSE
GB14165	439	210	1.063831612	0.199314104	FALSE
GB14166	309	169	0.870583592	0.006066083	FALSE
GB14167	395	230	0.780218792	-0.084298716	FALSE
GB14168	219	147	0.575114715	-0.289402794	FALSE
GB14169	1504	605	1.31379752	0.449280011	FALSE
GB14170	1852	990	0.903583668	0.03906616	FALSE
GB14171	4096	2969	0.464238622	-0.400278886	FALSE
GB14172	2947	1628	0.856146361	-0.008371147	FALSE
GB14173	NA	NA	NA	NA	FALSE
GB14174	1427	738	0.951292613	0.086775105	FALSE
GB14175	274	140	0.968749066	0.104231558	FALSE

GB14176	244	178	0.455003907	-0.409513602	FALSE
GB14177	0	3	NA	NA	FALSE
GB14178	1234	653	0.918187498	0.053669989	FALSE
GB14179	84	31	1.438121112	0.573603604	TRUE
GB14180	9	4	1.169925001	0.305407493	FALSE
GB14181	186	56	1.731803889	0.867286381	TRUE
GB14182	1848	973	0.925453047	0.060935538	FALSE
GB14183	967	681	0.505861091	-0.358656417	FALSE
GB14184	376	246	0.612074346	-0.252443162	FALSE
GB14185	2720	1846	0.559204098	-0.30531341	FALSE
GB14186	101	54	0.903323981	0.038806472	FALSE
GB14187	NA	NA	NA	NA	FALSE
GB14188	1050	581	0.853779259	-0.010738249	FALSE
GB14189	2502	1301	0.943460827	0.078943319	FALSE
GB14190	142	83	0.774707688	-0.08980982	FALSE
GB14191	4668	2978	0.648460807	-0.216056701	FALSE
GB14192	833	533	0.644180963	-0.220336546	FALSE
GB14193	110	42	1.389042291	0.524524782	TRUE
GB14194	250	130	0.943416472	0.078898963	FALSE
GB14195	2223	1188	0.903973112	0.039455604	FALSE
GB14196	1	0	NA	NA	FALSE
GB14197	203	138	0.55681146	-0.307706048	FALSE
GB14198	500	352	0.506352666	-0.358164842	FALSE
GB14199	1687	966	0.804364879	-0.060152629	FALSE
GB14200	8	8	0	-0.864517508	TRUE
GB14201	353	240	0.556633778	-0.307883731	FALSE
GB14202	563	259	1.120182824	0.255665316	FALSE
GB14203	921	563	0.710066234	-0.154451274	FALSE
GB14204	20	14	0.514573173	-0.349944335	FALSE
GB14205	10824	10273	0.075376194	-0.789141314	TRUE
GB14206	NA	NA	NA	NA	FALSE
GB14207	369	239	0.626610198	-0.23790731	FALSE
GB14208	370	213	0.79667184	-0.067845668	FALSE
GB14209	1631	896	0.864186145	-0.000331364	FALSE
GB14210	1152	658	0.807981228	-0.056536281	FALSE
GB14211	380	221	0.781953049	-0.082564459	FALSE
GB14212	431	200	1.107687869	0.243170361	FALSE
GB14213	NA	NA	NA	NA	FALSE
GB14214	981	585	0.745816512	-0.118700997	FALSE
GB14215	12	7	0.777607579	-0.08690993	FALSE
GB14216	475	170	1.482392767	0.617875259	TRUE
GB14217	2	1	1	0.135482492	FALSE
GB14218	334	193	0.791247255	-0.073270253	FALSE
GB14219	1522	687	1.147586355	0.283068846	FALSE

GB14220	1212	746	0.700142163	-0.164375345	FALSE
GB14221	NA	NA	NA	NA	FALSE
GB14222	529	173	1.612495684	0.747978176	TRUE
GB14223	768	694	0.146170648	-0.71834686	TRUE
GB14224	5412	2212	1.290810454	0.426292945	FALSE
GB14225	61	43	0.504472583	-0.360044925	FALSE
GB14226	593	458	0.372684506	-0.491833002	FALSE
GB14227	448	257	0.801730373	-0.062787135	FALSE
GB14228	910	447	1.025591714	0.161074206	FALSE
GB14229	438	207	1.081300102	0.216782594	FALSE
GB14230	543	52	3.38436867	2.519851161	TRUE
GB14231	754	408	0.885995371	0.021477863	FALSE
GB14232	752	466	0.690402707	-0.174114801	FALSE
GB14233	7	5	0.485426827	-0.379090681	FALSE
GB14234	491	265	0.889730665	0.025213157	FALSE
GB14235	41	38	0.109624491	-0.754893017	TRUE
GB14236	182	77	1.2410081	0.376490591	FALSE
GB14237	7	4	0.807354922	-0.057162586	FALSE
GB14238	981	498	0.978107394	0.113589886	FALSE
GB14239	185	136	0.443918619	-0.420598889	FALSE
GB14240	1825	940	0.957163802	0.092646294	FALSE
GB14241	2424	1563	0.63307192	-0.231445588	FALSE
GB14242	355	199	0.835050594	-0.029466914	FALSE
GB14243	109	55	0.986824611	0.122307103	FALSE
GB14244	1185	701	0.75740071	-0.107116799	FALSE
GB14245	0	1	NA	NA	FALSE
GB14246	14	9	0.637429921	-0.227087588	FALSE
GB14247	985	646	0.60858956	-0.255927949	FALSE
GB14248	3	0	NA	NA	FALSE
GB14249	306	125	1.291603558	0.42708605	FALSE
GB14250	1793	846	1.08364592	0.219128411	FALSE
GB14251	33	6	2.459431619	1.59491411	TRUE
GB14252	28	24	0.222392421	-0.642125087	TRUE
GB14253	862	462	0.899795018	0.035277509	FALSE
GB14254	1272	902	0.495899332	-0.368618176	FALSE
GB14255	1	3	-1.584962501	-2.449480009	TRUE
GB14256	547	290	0.915487933	0.050970424	FALSE
GB14257	0	1	NA	NA	FALSE
GB14258	1	3	-1.584962501	-2.449480009	TRUE
GB14259	37	13	1.509013647	0.644496139	TRUE
GB14260	202	113	0.83803252	-0.026484988	FALSE
GB14261	501723	322372	0.638164633	-0.226352875	FALSE
GB14262	1792	1499	0.257570254	-0.606947254	TRUE
GB14263	222	74	1.584962501	0.720444992	TRUE

GB14264	452	251	0.848635408	-0.0158821	FALSE
GB14265	2176	1198	0.861050648	-0.00346686	FALSE
GB14266	268	159	0.753206235	-0.111311273	FALSE
GB14267	24	15	0.678071905	-0.186445603	FALSE
GB14268	1	0	NA	NA	FALSE
GB14269	1763	1303	0.436195391	-0.428322118	FALSE
GB14270	5017	2590	0.95387284	0.089355332	FALSE
GB14271	NA	NA	NA	NA	FALSE
GB14272	1133	658	0.783988372	-0.080529136	FALSE
GB14273	1300	623	1.061207555	0.196690047	FALSE
GB14274	18	1	4.169925001	3.305407493	TRUE
GB14275	18	10	0.847996907	-0.016520602	FALSE
GB14276	435	311	0.484100821	-0.380416688	FALSE
GB14277	1539	1104	0.47925306	-0.385264449	FALSE
GB14278	1	0	NA	NA	FALSE
GB14279	845	509	0.731285685	-0.133231823	FALSE
GB14280	1222	620	0.978904165	0.114386656	FALSE
GB14281	719	454	0.663299473	-0.201218035	FALSE
GB14282	162	68	1.252387162	0.387869653	FALSE
GB14283	NA	NA	NA	NA	FALSE
GB14284	66388	43631	0.60556895	-0.258948558	FALSE
GB14285	3	1	1.584962501	0.720444992	TRUE
GB14286	205	155	0.403355694	-0.461161814	FALSE
GB14287	1524	1026	0.570832172	-0.293685336	FALSE
GB14288	369	268	0.461387816	-0.403129693	FALSE
GB14289	2722	1516	0.844397313	-0.020120195	FALSE
GB14290	5815	3320	0.80859595	-0.055921558	FALSE
GB14291	2607	1177	1.147276262	0.282758754	FALSE
GB14292	47	34	0.46712601	-0.397391498	FALSE
GB14293	1296	909	0.511713519	-0.35280399	FALSE
GB14294	742	388	0.935362534	0.070845026	FALSE
GB14295	965	531	0.861817081	-0.002700427	FALSE
GB14296	0	1	NA	NA	FALSE
GB14297	18	8	1.169925001	0.305407493	FALSE
GB14298	7069	3842	0.879648623	0.015131115	FALSE
GB14299	735	470	0.645083493	-0.219434015	FALSE
GB14300	203	121	0.74647268	-0.118044828	FALSE
GB14301	89	67	0.409644241	-0.454873268	FALSE
GB14302	1236	647	0.933841126	0.069323618	FALSE
GB14303	922	589	0.646499117	-0.218018392	FALSE
GB14304	407	227	0.842336497	-0.022181011	FALSE
GB14305	NA	NA	NA	NA	FALSE
GB14306	3	9	-1.584962501	-2.449480009	TRUE
GB14307	3191	1654	0.948049374	0.083531866	FALSE

GB14308	18	8	1.169925001	0.305407493	FALSE
GB14309	53	36	0.557995453	-0.306522055	FALSE
GB14310	598	301	0.990381997	0.125864489	FALSE
GB14311	130	74	0.812914447	-0.051603061	FALSE
GB14312	296	145	1.029544276	0.165026767	FALSE
GB14313	3766	2122	0.827608344	-0.036909165	FALSE
GB14314	1963	1141	0.782761381	-0.081756127	FALSE
GB14315	1472	821	0.842323544	-0.022193964	FALSE
GB14316	138	61	1.177787119	0.313269611	FALSE
GB14317	610	220	1.471305719	0.606788211	TRUE
GB14318	39	41	-0.072149786	-0.936667294	TRUE
GB14319	203	165	0.299013703	-0.565503805	TRUE
GB14320	40	28	0.514573173	-0.349944335	FALSE
GB14321	2517	1672	0.590130369	-0.274387139	FALSE
GB14322	NA	NA	NA	NA	FALSE
GB14323	813	292	1.477286983	0.612769475	TRUE
GB14324	3437	2312	0.572008454	-0.292509054	FALSE
GB14325	5	3	0.736965594	-0.127551914	FALSE
GB14326	535	363	0.559569343	-0.304948165	FALSE
GB14327	36	22	0.710493383	-0.154024126	FALSE
GB14328	56234	35029	0.682892868	-0.18162464	FALSE
GB14329	246	98	1.327804661	0.463287153	FALSE
GB14330	348	124	1.488747185	0.624229677	TRUE
GB14331	580	337	0.783304309	-0.081213199	FALSE
GB14332	281	96	1.549463819	0.684946311	TRUE
GB14333	NA	NA	NA	NA	FALSE
GB14334	475	260	0.86941589	0.004898382	FALSE
GB14335	639	393	0.701286619	-0.16323089	FALSE
GB14336	3	1	1.584962501	0.720444992	TRUE
GB14337	587	359	0.709376659	-0.155140849	FALSE
GB14338	58	26	1.157541277	0.293023769	FALSE
GB14339	1473	875	0.751402508	-0.113115	FALSE
GB14340	637	336	0.922832139	0.058314631	FALSE
GB14341	735	554	0.407858274	-0.456659235	FALSE
GB14342	693	561	0.304854582	-0.559662927	TRUE
GB14343	927	461	1.007802588	0.14328508	FALSE
GB14344	262	153	0.776035159	-0.088482349	FALSE
GB14345	1272	592	1.10342959	0.238912081	FALSE
GB14346	6577	3309	0.991034379	0.12651687	FALSE
GB14347	2	3	-0.584962501	-1.449480009	TRUE
GB14348	1257	785	0.679220091	-0.185297418	FALSE
GB14349	5328	2992	0.832483907	-0.032033601	FALSE
GB14350	1090	557	0.968578902	0.104061394	FALSE
GB14351	99	44	1.169925001	0.305407493	FALSE

GB14352	77	31	1.31259023	0.448072722	FALSE
GB14353	1044	440	1.246546283	0.382028775	FALSE
GB14354	343	208	0.721625048	-0.14289246	FALSE
GB14355	272	105	1.373217324	0.508699815	TRUE
GB14356	1027	746	0.461188646	-0.403328862	FALSE
GB14357	1038	478	1.11872392	0.254206412	FALSE
GB14358	2606	1279	1.02682082	0.162303311	FALSE
GB14359	48	26	0.884522783	0.020005274	FALSE
GB14360	889	552	0.687515152	-0.177002356	FALSE
GB14361	11530970	5524047	1.06171638	0.197198872	FALSE
GB14362	39	5	2.963474124	2.098956616	TRUE
GB14363	123	92	0.418952549	-0.445564959	FALSE
GB14364	NA	NA	NA	NA	FALSE
GB14365	907	559	0.698254268	-0.166263241	FALSE
GB14366	129	52	1.310787537	0.446270029	FALSE
GB14367	NA	NA	NA	NA	FALSE
GB14368	440	269	0.709897351	-0.154620157	FALSE
GB14369	203	141	0.525784565	-0.338732944	FALSE
GB14370	254	139	0.869743614	0.005226106	FALSE
GB14371	311	174	0.837827274	-0.026690234	FALSE
GB14372	1547	967	0.677885402	-0.186632106	FALSE
GB14373	1023	580	0.81868134	-0.045836169	FALSE
GB14374	2584	1188	1.121071234	0.256553726	FALSE
GB14375	3	6	-1	-1.864517508	TRUE
GB14376	2	1	1	0.135482492	FALSE
GB14377	18	7	1.362570079	0.498052571	FALSE
GB14378	789	424	0.895961035	0.031443527	FALSE
GB14379	192	147	0.385290156	-0.479227352	FALSE
GB14380	1964	1131	0.796196	-0.068321508	FALSE
GB14381	NA	NA	NA	NA	FALSE
GB14382	1674	832	1.008644094	0.144126586	FALSE
GB14383	NA	NA	NA	NA	FALSE
GB14384	102	35	1.543142325	0.678624817	TRUE
GB14385	120	50	1.263034406	0.398516898	FALSE
GB14386	432	263	0.715968513	-0.148548995	FALSE
GB14387	NA	NA	NA	NA	FALSE
GB14388	3732	2202	0.761134517	-0.103382991	FALSE
GB14389	367	181	1.019790366	0.155272857	FALSE
GB14390	928	507	0.872139058	0.00762155	FALSE
GB14391	NA	NA	NA	NA	FALSE
GB14392	NA	NA	NA	NA	FALSE
GB14393	NA	NA	NA	NA	FALSE
GB14394	835	514	0.700007838	-0.16450967	FALSE
GB14395	1100	567	0.956082883	0.091565375	FALSE

GB14396	33472	13927	1.265070235	0.400552726	FALSE
GB14397	13422	7888	0.766868205	-0.097649303	FALSE
GB14398	225	134	0.747692001	-0.116825508	FALSE
GB14399	381	186	1.034488376	0.169970868	FALSE
GB14400	1038	598	0.795589054	-0.068928454	FALSE
GB14401	345	212	0.702532097	-0.161985411	FALSE
GB14402	4241	1798	1.238011462	0.373493954	FALSE
GB14403	NA	NA	NA	NA	FALSE
GB14404	1	1	0	-0.864517508	TRUE
GB14405	591	389	0.603387975	-0.261129533	FALSE
GB14406	39	26	0.584962501	-0.279555008	FALSE
GB14407	112	67	0.741265732	-0.123251777	FALSE
GB14408	251	152	0.723616041	-0.140901468	FALSE
GB14409	23250	15589	0.576702331	-0.287815177	FALSE
GB14410	115	68	0.75802721	-0.106490299	FALSE
GB14411	266	133	1	0.135482492	FALSE
GB14412	533	229	1.218787935	0.354270426	FALSE
GB14413	1000	543	0.880975897	0.016458389	FALSE
GB14414	24	9	1.415037499	0.550519991	TRUE
GB14415	115	45	1.353636955	0.489119446	FALSE
GB14416	308	118	1.384143491	0.519625983	TRUE
GB14417	896	656	0.449802917	-0.414714591	FALSE
GB14418	229	137	0.741171705	-0.123345803	FALSE
GB14419	97	45	1.108059746	0.243542238	FALSE
GB14420	1316	779	0.756464256	-0.108053253	FALSE
GB14421	247	102	1.27594189	0.411424381	FALSE
GB14422	1473	745	0.9834451	0.118927591	FALSE
GB14423	777	454	0.775222301	-0.089295207	FALSE
GB14424	12283	5380	1.19098489	0.326467381	FALSE
GB14425	60	48	0.321928095	-0.542589413	TRUE
GB14426	141	61	1.208814015	0.344296507	FALSE
GB14427	585	370	0.660911354	-0.203606154	FALSE
GB14428	30	26	0.206450877	-0.658066631	TRUE
GB14429	843	456	0.886498807	0.021981298	FALSE
GB14430	NA	NA	NA	NA	FALSE
GB14431	557	424	0.393613063	-0.470904446	FALSE
GB14432	804	552	0.542527234	-0.321990274	FALSE
GB14433	676	304	1.152951923	0.288434415	FALSE
GB14434	2903	1535	0.919305916	0.054788408	FALSE
GB14435	709	308	1.202855277	0.338337768	FALSE
GB14436	3503	2070	0.75896022	-0.105557288	FALSE
GB14437	1012	510	0.988640138	0.12412263	FALSE
GB14438	873	499	0.806941838	-0.05757567	FALSE
GB14439	1678	1046	0.681859864	-0.182657644	FALSE

GB14440	198	109	0.861172295	-0.003345213	FALSE
GB14441	610	315	0.953457414	0.088939906	FALSE
GB14442	252	131	0.943856922	0.079339414	FALSE
GB14443	1620	973	0.735482103	-0.129035405	FALSE
GB14444	1228	764	0.684666017	-0.179851491	FALSE
GB14445	1	3	-1.584962501	-2.449480009	TRUE
GB14446	29	24	0.273018494	-0.591499014	TRUE
GB14447	11	4	1.459431619	0.59491411	TRUE
GB14448	707	416	0.765126687	-0.099390822	FALSE
GB14449	NA	NA	NA	NA	FALSE
GB14450	924	532	0.796466606	-0.068050902	FALSE
GB14451	1066	687	0.633825434	-0.230692074	FALSE
GB14452	40	17	1.234465254	0.369947745	FALSE
GB14453	2	2	0	-0.864517508	TRUE
GB14454	2448	1120	1.128104826	0.263587317	FALSE
GB14455	369	270	0.450661409	-0.413856099	FALSE
GB14456	365	112	1.704397732	0.839880223	TRUE
GB14457	595	223	1.415845958	0.55132845	TRUE
GB14458	810	551	0.555869589	-0.308647919	FALSE
GB14459	120	75	0.678071905	-0.186445603	FALSE
GB14460	12	4	1.584962501	0.720444992	TRUE
GB14461	971	544	0.835864644	-0.028652864	FALSE
GB14462	1382	685	1.012581723	0.148064214	FALSE
GB14463	160	95	0.752072487	-0.112445022	FALSE
GB14464	NA	NA	NA	NA	FALSE
GB14465	3	1	1.584962501	0.720444992	TRUE
GB14466	37	22	0.750021747	-0.114495761	FALSE
GB14467	3973	2152	0.884550716	0.020033207	FALSE
GB14468	112	69	0.698830465	-0.165687043	FALSE
GB14469	4533	2517	0.848760945	-0.015756564	FALSE
GB14470	1	1	0	-0.864517508	TRUE
GB14471	NA	NA	NA	NA	FALSE
GB14472	1160	604	0.941504351	0.076986842	FALSE
GB14473	1980	1002	0.982617922	0.118100413	FALSE
GB14474	304	187	0.701033054	-0.163484455	FALSE
GB14475	704	348	1.016488123	0.151970614	FALSE
GB14476	565	376	0.587518206	-0.276999303	FALSE
GB14477	143	73	0.970046778	0.10552927	FALSE
GB14478	NA	NA	NA	NA	FALSE
GB14479	324	157	1.045229254	0.180711746	FALSE
GB14480	0	1	NA	NA	FALSE
GB14481	3111	1207	1.365952719	0.50143521	TRUE
GB14482	256	127	1.011315313	0.146797805	FALSE
GB14483	9	3	1.584962501	0.720444992	TRUE

GB14484	1492	984	0.600517315	-0.264000193	FALSE
GB14485	3332	2020	0.722033108	-0.142484401	FALSE
GB14486	876	464	0.916806064	0.052288556	FALSE
GB14487	379	245	0.629416099	-0.235101409	FALSE
GB14488	1339	687	0.962773957	0.098256448	FALSE
GB14489	862	559	0.624839586	-0.239677922	FALSE
GB14490	4986	2161	1.206183811	0.341666302	FALSE
GB14491	2042	1145	0.834635268	-0.02988224	FALSE
GB14492	17	6	1.502500341	0.637982832	TRUE
GB14493	173	44	1.975196609	1.110679101	TRUE
GB14494	8257	9278	-0.168196141	-1.032713649	TRUE
GB14495	1757	974	0.851120514	-0.013396994	FALSE
GB14496	3588	1953	0.877487941	0.012970433	FALSE
GB14497	139	48	1.533978572	0.669461064	TRUE
GB14498	56	18	1.637429921	0.772912412	TRUE
GB14499	2500	1655	0.595096878	-0.26942063	FALSE
GB14500	247	120	1.041476636	0.176959128	FALSE
GB14501	11	0	NA	NA	FALSE
GB14502	136	91	0.579668201	-0.284849307	FALSE
GB14503	279	130	1.101753499	0.23723599	FALSE
GB14504	119	57	1.061927749	0.197410241	FALSE
GB14505	308	125	1.301002256	0.436484748	FALSE
GB14506	768	309	1.313499473	0.448981964	FALSE
GB14507	731	511	0.516548115	-0.347969393	FALSE
GB14508	1763	914	0.947766404	0.083248896	FALSE
GB14509	6	2	1.584962501	0.720444992	TRUE
GB14510	399	254	0.651560249	-0.212957259	FALSE
GB14511	0	1	NA	NA	FALSE
GB14512	6	8	-0.415037499	-1.279555008	TRUE
GB14513	659	383	0.782934073	-0.081583435	FALSE
GB14514	664	371	0.839764055	-0.024753454	FALSE
GB14515	0	2	NA	NA	FALSE
GB14516	96	51	0.912537159	0.04801965	FALSE
GB14517	11545	7489	0.624423178	-0.24009433	FALSE
GB14518	500	310	0.689659879	-0.174857629	FALSE
GB14519	1	0	NA	NA	FALSE
GB14520	2266	1010	1.165792568	0.30127506	FALSE
GB14521	195	130	0.584962501	-0.279555008	FALSE
GB14522	1763	1028	0.77819221	-0.086325298	FALSE
GB14523	397	207	0.93950824	0.074990731	FALSE
GB14524	386	211	0.871357849	0.00684034	FALSE
GB14525	1033	481	1.102731455	0.238213947	FALSE
GB14526	2	3	-0.584962501	-1.449480009	TRUE
GB14527	6	9	-0.584962501	-1.449480009	TRUE

GB14528	15	13	0.206450877	-0.658066631	TRUE
GB14529	3364	1745	0.946950669	0.082433161	FALSE
GB14530	28	23	0.283792966	-0.580724542	TRUE
GB14531	468	264	0.8259706	-0.038546908	FALSE
GB14532	6	4	0.584962501	-0.279555008	FALSE
GB14533	481	207	1.216406126	0.351888618	FALSE
GB14534	81	33	1.295455884	0.430938375	FALSE
GB14535	472	222	1.088227183	0.223709675	FALSE
GB14536	NA	NA	NA	NA	FALSE
GB14537	684	305	1.165187082	0.300669574	FALSE
GB14538	450	241	0.900891855	0.036374347	FALSE
GB14539	1499	994	0.592682626	-0.271834882	FALSE
GB14540	1166	519	1.167761345	0.303243837	FALSE
GB14541	682	422	0.69252874	-0.171988768	FALSE
GB14542	584	262	1.156401557	0.291884049	FALSE
GB14543	271	192	0.497186541	-0.367330968	FALSE
GB14544	111	76	0.546488353	-0.318029155	FALSE
GB14545	246	172	0.516249751	-0.348267758	FALSE
GB14546	671	367	0.870532703	0.006015195	FALSE
GB14547	23	7	1.716207034	0.851689526	TRUE
GB14548	143	56	1.352516415	0.487998906	FALSE
GB14549	18	27	-0.584962501	-1.449480009	TRUE
GB14550	97	69	0.491388385	-0.373129123	FALSE
GB14551	548	399	0.457787147	-0.406730362	FALSE
GB14552	540	271	0.994666556	0.130149047	FALSE
GB14553	1069	679	0.654778373	-0.209739135	FALSE
GB14554	772	498	0.632455105	-0.232062403	FALSE
GB14555	1298	495	1.390789953	0.526272445	TRUE
GB14556	930	364	1.353292266	0.488774757	FALSE
GB14557	764	462	0.725679787	-0.138837722	FALSE
GB14558	114	86	0.406625259	-0.457892249	FALSE
GB14559	131	65	1.011055189	0.14653768	FALSE
GB14560	137	93	0.558873272	-0.305644236	FALSE
GB14561	8	7	0.192645078	-0.67187243	TRUE
GB14562	18	10	0.847996907	-0.016520602	FALSE
GB14563	2011	1224	0.716309524	-0.148207985	FALSE
GB14564	20775	17912	0.213922044	-0.650595465	TRUE
GB14565	946	606	0.64252239	-0.221995118	FALSE
GB14566	1381	926	0.576629221	-0.287888287	FALSE
GB14567	323	169	0.934510918	0.06999341	FALSE
GB14568	1	0	NA	NA	FALSE
GB14569	9820	5955	0.721621516	-0.142895992	FALSE
GB14570	1633	866	0.915085861	0.050568353	FALSE
GB14571	111	68	0.706953025	-0.157564483	FALSE

GB14572	499	217	1.201344773	0.336827265	FALSE
GB14573	19	7	1.440572591	0.576055083	TRUE
GB14574	NA	NA	NA	NA	FALSE
GB14575	884	457	0.951852204	0.087334696	FALSE
GB14576	NA	NA	NA	NA	FALSE
GB14577	1036	554	0.903066122	0.038548613	FALSE
GB14578	870	422	1.043772402	0.179254894	FALSE
GB14579	0	1	NA	NA	FALSE
GB14580	11	1	3.459431619	2.59491411	TRUE
GB14581	95	55	0.788495895	-0.076021614	FALSE
GB14582	244	155	0.654612932	-0.209904576	FALSE
GB14583	1064	709	0.585640618	-0.27887689	FALSE
GB14584	512	318	0.687117045	-0.177400464	FALSE
GB14585	28	14	1	0.135482492	FALSE
GB14586	665	272	1.289747689	0.425230181	FALSE
GB14587	47	29	0.696607857	-0.167909652	FALSE
GB14588	1163	559	1.056930909	0.1924134	FALSE
GB14589	647	256	1.337621902	0.473104394	FALSE
GB14590	NA	NA	NA	NA	FALSE
GB14591	1	0	NA	NA	FALSE
GB14592	381	211	0.852547999	-0.01196951	FALSE
GB14593	69	24	1.523561956	0.659044448	TRUE
GB14594	11680	11019	0.084046972	-0.780470536	TRUE
GB14595	19850	13424	0.564324386	-0.300193122	FALSE
GB14596	810	508	0.673093411	-0.191424097	FALSE
GB14597	1773	887	0.999186527	0.134669018	FALSE
GB14598	104	85	0.291048782	-0.573468726	TRUE
GB14599	493	257	0.939819287	0.075301779	FALSE
GB14600	652	288	1.178803153	0.314285644	FALSE
GB14601	625	372	0.748553568	-0.11596394	FALSE
GB14602	850	287	1.566412104	0.701894596	TRUE
GB14603	287774	173967	0.726122587	-0.138394921	FALSE
GB14604	NA	NA	NA	NA	FALSE
GB14605	1	0	NA	NA	FALSE
GB14606	NA	NA	NA	NA	FALSE
GB14607	1516599	787027	0.946354642	0.081837133	FALSE
GB14608	704	340	1.050040682	0.185523174	FALSE
GB14609	4	1	2	1.135482492	TRUE
GB14610	222	162	0.454565863	-0.409951645	FALSE
GB14611	229	177	0.371598238	-0.49291927	FALSE
GB14612	1612	989	0.704809318	-0.159708191	FALSE
GB14613	66	41	0.686842115	-0.177675394	FALSE
GB14614	46	32	0.523561956	-0.340955552	FALSE
GB14615	17	15	0.180572246	-0.683945263	TRUE

GB14616	1222	682	0.841400641	-0.023116868	FALSE
GB14617	18	7	1.362570079	0.498052571	FALSE
GB14618	447	265	0.754282472	-0.110235037	FALSE
GB14619	3096	1562	0.987011018	0.12249351	FALSE
GB14620	229	90	1.347350692	0.482833183	FALSE
GB14621	19184	10371	0.887348554	0.022831046	FALSE
GB14622	1801	892	1.013682566	0.149165058	FALSE
GB14623	3888	2180	0.834700084	-0.029817424	FALSE
GB14624	1931	1141	0.759049373	-0.105468136	FALSE
GB14625	95	51	0.897430266	0.032912758	FALSE
GB14626	35	21	0.736965594	-0.127551914	FALSE
GB14627	601	504	0.253941257	-0.610576251	TRUE
GB14628	677	340	0.993621087	0.129103579	FALSE
GB14629	1489	731	1.026400443	0.161882934	FALSE
GB14630	531	357	0.572787787	-0.291729722	FALSE
GB14631	0	1	NA	NA	FALSE
GB14632	179	122	0.55307844	-0.311439069	FALSE
GB14633	90	74	0.282399731	-0.582117778	TRUE
GB14634	23929	14097	0.763371934	-0.101145574	FALSE
GB14635	1711	1010	0.760484467	-0.104033041	FALSE
GB14636	381	170	1.164256251	0.299738743	FALSE
GB14637	2260	1253	0.850936358	-0.01358115	FALSE
GB14638	190	83	1.194816177	0.330298669	FALSE
GB14639	374	142	1.39714734	0.532629832	TRUE
GB14640	609	447	0.446167397	-0.418350112	FALSE
GB14641	355	168	1.079357792	0.214840283	FALSE
GB14642	172644	76410	1.175966831	0.311449322	FALSE
GB14643	796	342	1.218772106	0.354254597	FALSE
GB14644	2	1	1	0.135482492	FALSE
GB14645	923	461	1.001563897	0.137046389	FALSE
GB14646	16	3	2.415037499	1.550519991	TRUE
GB14647	12	13	-0.115477217	-0.979994726	TRUE
GB14648	7	7	0	-0.864517508	TRUE
GB14649	808	372	1.119052672	0.254535163	FALSE
GB14650	351	170	1.045936284	0.181418776	FALSE
GB14651	390	163	1.25860216	0.394084651	FALSE
GB14652	270	124	1.122619287	0.258101778	FALSE
GB14653	226	165	0.453856748	-0.41066076	FALSE
GB14654	12	6	1	0.135482492	FALSE
GB14655	78	51	0.612976877	-0.251540631	FALSE
GB14656	3829	2827	0.437695777	-0.426821731	FALSE
GB14657	11725	5840	1.005547649	0.14103014	FALSE
GB14658	755	448	0.752977912	-0.111539596	FALSE
GB14659	392	207	0.921222887	0.056705378	FALSE

GB14660	106	75	0.499101764	-0.365415744	FALSE
GB14661	NA	NA	NA	NA	FALSE
GB14662	NA	NA	NA	NA	FALSE
GB14663	84	43	0.966052668	0.10153516	FALSE
GB14664	591	335	0.818997035	-0.045520473	FALSE
GB14665	305	144	1.082740431	0.218222923	FALSE
GB14666	3424	2060	0.733038364	-0.131479144	FALSE
GB14667	772	414	0.89897008	0.034452571	FALSE
GB14668	352	191	0.882002791	0.017485282	FALSE
GB14669	302	139	1.119463667	0.254946158	FALSE
GB14670	30	24	0.321928095	-0.542589413	TRUE
GB14671	677	322	1.072095145	0.207577637	FALSE
GB14672	505	241	1.067250241	0.202732733	FALSE
GB14673	3736	2278	0.713726708	-0.1507908	FALSE
GB14674	4033	2350	0.779192649	-0.085324859	FALSE
GB14675	157	89	0.818887318	-0.04563019	FALSE
GB14676	194	161	0.268995964	-0.595521544	TRUE
GB14677	5493	3692	0.573191739	-0.291325769	FALSE
GB14678	3844	2461	0.643363678	-0.22115383	FALSE
GB14679	1104	709	0.63888264	-0.225634869	FALSE
GB14680	27	14	0.94753258	0.083015072	FALSE
GB14681	177	134	0.40151636	-0.463001149	FALSE
GB14682	5	4	0.321928095	-0.542589413	TRUE
GB14683	389	222	0.809210479	-0.05530703	FALSE
GB14684	1074	834	0.364874705	-0.499642804	FALSE
GB14685	316	189	0.741538324	-0.122979184	FALSE
GB14686	1360	828	0.715903979	-0.14861353	FALSE
GB14687	369	191	0.950048178	0.08553067	FALSE
GB14688	474	256	0.888743249	0.024225741	FALSE
GB14689	930	349	1.41400368	0.549486171	TRUE
GB14690	949	631	0.588768082	-0.275749426	FALSE
GB14691	525	310	0.760049207	-0.104468301	FALSE
GB14692	206	119	0.791682764	-0.072834744	FALSE
GB14693	1134	609	0.896906507	0.032388999	FALSE
GB14694	158	68	1.216317907	0.351800399	FALSE
GB14695	87	47	0.888354644	0.023837136	FALSE
GB14696	655	353	0.891826723	0.027309215	FALSE
GB14697	1203	603	0.996406735	0.131889227	FALSE
GB14698	1087	625	0.798423845	-0.066093663	FALSE
GB14699	NA	NA	NA	NA	FALSE
GB14700	145	92	0.656347134	-0.208170374	FALSE
GB14701	890	597	0.576074405	-0.288443104	FALSE
GB14702	328	184	0.833990049	-0.03052746	FALSE
GB14703	5635	2289	1.299698148	0.435180639	FALSE

GB14704	NA	NA	NA	NA	FALSE
GB14705	1392	739	0.913512942	0.048995433	FALSE
GB14706	1009	515	0.970281837	0.105764329	FALSE
GB14707	418	229	0.868155344	0.003637836	FALSE
GB14708	332	184	0.851477475	-0.013040033	FALSE
GB14709	8851	6882	0.36301257	-0.501504938	TRUE
GB14710	3879	1795	1.111700932	0.247183423	FALSE
GB14711	NA	NA	NA	NA	FALSE
GB14712	417	355	0.232228359	-0.632289149	TRUE
GB14713	NA	NA	NA	NA	FALSE
GB14714	35	14	1.321928095	0.457410587	FALSE
GB14715	2529	1424	0.828617891	-0.035899618	FALSE
GB14716	1	0	NA	NA	FALSE
GB14717	265	157	0.755227801	-0.109289708	FALSE
GB14718	663	443	0.581702172	-0.282815337	FALSE
GB14719	752	289	1.379663169	0.515145661	TRUE
GB14720	371	169	1.13439594	0.269878432	FALSE
GB14721	1083	473	1.195121154	0.330603646	FALSE
GB14722	1905	1022	0.898395801	0.033878293	FALSE
GB14723	1231	960	0.358724451	-0.505793057	TRUE
GB14724	346	249	0.474626296	-0.389891213	FALSE
GB14725	1	0	NA	NA	FALSE
GB14726	209	86	1.281094377	0.416576869	FALSE
GB14727	1217	688	0.822848698	-0.04166881	FALSE
GB14728	NA	NA	NA	NA	FALSE
GB14729	80	36	1.152003093	0.287485585	FALSE
GB14730	848	469	0.854476342	-0.010041166	FALSE
GB14731	2597	1412	0.879105925	0.014588417	FALSE
GB14732	10368	5766	0.846494881	-0.018022627	FALSE
GB14733	697	412	0.758514319	-0.10600319	FALSE
GB14734	4609	2608	0.821509898	-0.04300761	FALSE
GB14735	713	471	0.598175017	-0.266342491	FALSE
GB14736	240	158	0.603109847	-0.261407661	FALSE
GB14737	561	338	0.730977524	-0.133539984	FALSE
GB14738	493	297	0.731124716	-0.133392793	FALSE
GB14739	6	5	0.263034406	-0.601483102	TRUE
GB14740	805	482	0.739955637	-0.124561872	FALSE
GB14741	1536	679	1.177694736	0.313177228	FALSE
GB14742	164	106	0.62963155	-0.234885958	FALSE
GB14743	478	322	0.56994993	-0.294567578	FALSE
GB14744	751	415	0.855701571	-0.008815937	FALSE
GB14745	1169	648	0.851209212	-0.013308297	FALSE
GB14746	30	22	0.447458977	-0.417058531	FALSE
GB14747	754	442	0.770518154	-0.093999354	FALSE

GB14748	5	3	0.736965594	-0.127551914	FALSE
GB14749	4938	2651	0.897390166	0.032872658	FALSE
GB14750	6548	3688	0.828215666	-0.036301842	FALSE
GB14751	431	186	1.212385248	0.34786774	FALSE
GB14752	139	59	1.236298023	0.371780515	FALSE
GB14753	2307	1156	0.996876606	0.132359098	FALSE
GB14754	376	261	0.526682855	-0.337834653	FALSE
GB14755	636	284	1.163135836	0.298618327	FALSE
GB14756	NA	NA	NA	NA	FALSE
GB14757	31	19	0.706268797	-0.158248711	FALSE
GB14758	30532	21275	0.521162965	-0.343354544	FALSE
GB14759	259	138	0.908283831	0.043766323	FALSE
GB14760	276	136	1.021061616	0.156544107	FALSE
GB14761	3418	2038	0.745998346	-0.118519163	FALSE
GB14762	403	229	0.81543224	-0.049085268	FALSE
GB14763	651	363	0.842687995	-0.021829513	FALSE
GB14764	71	28	1.342392197	0.477874689	FALSE
GB14765	1058	639	0.727451791	-0.137065717	FALSE
GB14766	235	149	0.657348426	-0.207169082	FALSE
GB14767	36	7	2.362570079	1.498052571	TRUE
GB14768	1	0	NA	NA	FALSE
GB14769	444	244	0.863678529	-0.00083898	FALSE
GB14770	1368	595	1.201106657	0.336589148	FALSE
GB14771	828	547	0.598089935	-0.266427574	FALSE
GB14772	674	364	0.888810141	0.024292633	FALSE
GB14773	2753	1460	0.915036241	0.050518733	FALSE
GB14774	6	1	2.584962501	1.720444992	TRUE
GB14775	251	139	0.852602481	-0.011915027	FALSE
GB14776	81	50	0.695993813	-0.168523695	FALSE
GB14777	4	5	-0.321928095	-1.186445603	TRUE
GB14778	787	518	0.603411538	-0.261105971	FALSE
GB14779	10	11	-0.137503524	-1.002021032	TRUE
GB14780	344	176	0.966833136	0.102315628	FALSE
GB14781	106	55	0.946560741	0.082043233	FALSE
GB14782	508	337	0.592079906	-0.272437603	FALSE
GB14783	1472	903	0.704979779	-0.15953773	FALSE
GB14784	58248	35713	0.705759185	-0.158758324	FALSE
GB14785	1081	722	0.582295781	-0.282221727	FALSE
GB14786	2175	1111	0.969156584	0.104639076	FALSE
GB14787	13	1	3.700439718	2.83592221	TRUE
GB14788	2037	1243	0.712619684	-0.151897824	FALSE
GB14789	7420	4401	0.753587815	-0.110929693	FALSE
GB14790	93	46	1.015596855	0.151079347	FALSE
GB14791	39008	24719	0.65814965	-0.206367858	FALSE

GB14792	474	292	0.69891869	-0.165598818	FALSE
GB14793	81	52	0.639410285	-0.225107224	FALSE
GB14795	1083	545	0.990705108	0.1261876	FALSE
GB14796	557	369	0.594056511	-0.270460997	FALSE
GB14797	370	182	1.02358682	0.159069312	FALSE
GB14798	33021	23760	0.474848975	-0.389668533	FALSE
GB14799	511	368	0.473617525	-0.390899983	FALSE
GB14800	1131	698	0.696299988	-0.168217521	FALSE
GB14801	61	21	1.538419915	0.673902406	TRUE
GB14802	7	1	2.807354922	1.942837414	TRUE
GB14803	3214	1435	1.163319192	0.298801684	FALSE
GB14804	288	197	0.547873182	-0.316644326	FALSE
GB14805	207	116	0.835505962	-0.029011546	FALSE
GB14806	533	291	0.87311638	0.008598872	FALSE
GB14807	420	311	0.433474748	-0.431042761	FALSE
GB14808	13	4	1.700439718	0.83592221	TRUE
GB14809	726	340	1.094434802	0.229917294	FALSE
GB14810	697	379	0.878960808	0.014443299	FALSE
GB14811	17	6	1.502500341	0.637982832	TRUE
GB14812	906	537	0.754588962	-0.109928546	FALSE
GB14813	19379	13382	0.534200377	-0.330317131	FALSE
GB14814	672	437	0.620827953	-0.243689555	FALSE
GB14815	302	154	0.971618199	0.10710069	FALSE
GB14816	33	29	0.186413124	-0.678104384	TRUE
GB14817	2051	923	1.151924939	0.287407431	FALSE
GB14818	4	0	NA	NA	FALSE
GB14819	17	10	0.765534746	-0.098982762	FALSE
GB14820	20	9	1.152003093	0.287485585	FALSE
GB14821	432	296	0.545434137	-0.319083372	FALSE
GB14822	241	106	1.184968882	0.320451373	FALSE
GB14823	276	129	1.097297201	0.232779693	FALSE
GB14824	3174	1059	1.583599539	0.719082031	TRUE
GB14825	976	513	0.927922322	0.063404814	FALSE
GB14826	1854	818	1.180468496	0.315950987	FALSE
GB14827	215	130	0.725825037	-0.138692472	FALSE
GB14828	NA	NA	NA	NA	FALSE
GB14829	776	407	0.931027858	0.06651035	FALSE
GB14830	612	245	1.320749904	0.456232395	FALSE
GB14831	467	282	0.727727387	-0.136790121	FALSE
GB14832	1959	1125	0.800192396	-0.064325112	FALSE
GB14833	341	132	1.36923381	0.504716301	TRUE
GB14834	717	395	0.860120466	-0.004397043	FALSE
GB14835	436	229	0.928980537	0.064463028	FALSE
GB14836	4996	270	4.209742165	3.345224656	TRUE

GB14837	1151	692	0.73404389	-0.130473618	FALSE
GB14838	821	503	0.706823822	-0.157693686	FALSE
GB14839	100	57	0.810966176	-0.053551333	FALSE
GB14840	0	2	NA	NA	FALSE
GB14841	167	75	1.154885602	0.290368094	FALSE
GB14842	470	270	0.79970135	-0.064816159	FALSE
GB14843	190	79	1.26607486	0.401557352	FALSE
GB14844	82	53	0.62963155	-0.234885958	FALSE
GB14845	207	101	1.035275475	0.170757966	FALSE
GB14846	1936	964	1.005973901	0.141456393	FALSE
GB14847	332	169	0.974159995	0.109642487	FALSE
GB14848	7509	3332	1.172232391	0.307714883	FALSE
GB14849	28060	16450	0.770427425	-0.094090083	FALSE
GB14850	19	16	0.247927513	-0.616589995	TRUE
GB14851	2423	1016	1.253894003	0.389376494	FALSE
GB14852	136995	85978	0.672083783	-0.192433725	FALSE
GB14853	3942	2373	0.732215676	-0.132301832	FALSE
GB14854	86	68	0.338801913	-0.525715595	TRUE
GB14855	11	14	-0.347923303	-1.212440812	TRUE
GB14856	122	75	0.701918647	-0.162598861	FALSE
GB14857	1375	641	1.101035357	0.236517848	FALSE
GB14858	519	271	0.937441687	0.072924179	FALSE
GB14859	93	49	0.924448967	0.059931459	FALSE
GB14860	375	223	0.749846885	-0.114670623	FALSE
GB14861	18	3	2.584962501	1.720444992	TRUE
GB14862	1060	518	1.033040262	0.168522753	FALSE
GB14863	1995	1034	0.948152561	0.083635052	FALSE
GB14864	477	252	0.920565533	0.056048024	FALSE
GB14865	6166	3711	0.732526888	-0.13199062	FALSE
GB14866	5327	4688	0.184350711	-0.680166797	TRUE
GB14867	51	38	0.424497829	-0.44001968	FALSE
GB14868	675	319	1.081331078	0.21681357	FALSE
GB14869	362	178	1.024112456	0.159594948	FALSE
GB14870	12802	5606	1.191325564	0.326808056	FALSE
GB14871	109	131	-0.265238677	-1.129756185	TRUE
GB14872	826	463	0.835129588	-0.02938792	FALSE
GB14873	217	56	1.95419631	1.089678802	TRUE
GB14874	5163	2963	0.801150972	-0.063366537	FALSE
GB14875	255	163	0.645625283	-0.218892226	FALSE
GB14876	175	76	1.203283598	0.33876609	FALSE
GB14877	44	38	0.211504105	-0.653013403	TRUE
GB14878	35	20	0.807354922	-0.057162586	FALSE
GB14879	177	102	0.795180208	-0.0693373	FALSE
GB14880	571	371	0.622071559	-0.24244595	FALSE

GB14881	803	376	1.094667326	0.230149818	FALSE
GB14882	172	127	0.437580068	-0.42693744	FALSE
GB14883	NA	NA	NA	NA	FALSE
GB14884	160	120	0.415037499	-0.449480009	FALSE
GB14885	379	200	0.922197848	0.05768034	FALSE
GB14886	3509	2047	0.777548845	-0.086968663	FALSE
GB14887	280	150	0.900464326	0.035946818	FALSE
GB14888	9	5	0.847996907	-0.016520602	FALSE
GB14889	5637	3121	0.85291921	-0.011598298	FALSE
GB14890	6	1	2.584962501	1.720444992	TRUE
GB14891	349	230	0.601593175	-0.262924333	FALSE
GB14892	374	295	0.342323316	-0.522194193	TRUE
GB14893	NA	NA	NA	NA	FALSE
GB14894	1273	792	0.684660084	-0.179857425	FALSE
GB14895	1325	949	0.481512367	-0.383005141	FALSE
GB14896	1	0	NA	NA	FALSE
GB14897	657	400	0.715893371	-0.148624138	FALSE
GB14898	299	285	0.069183565	-0.795333943	TRUE
GB14899	481	312	0.624490865	-0.240026643	FALSE
GB14900	114	82	0.47533801	-0.389179499	FALSE
GB14901	366	226	0.695520876	-0.168996632	FALSE
GB14902	30	11	1.447458977	0.582941469	TRUE
GB14903	500	299	0.74178261	-0.122734898	FALSE
GB14904	159	89	0.837149524	-0.027367984	FALSE
GB14905	59	34	0.795180208	-0.0693373	FALSE
GB14906	263	172	0.612654235	-0.251863274	FALSE
GB14907	5952	4030	0.562594688	-0.301922821	FALSE
GB14908	1475	788	0.90444742	0.039929911	FALSE
GB14909	1777	840	1.080982448	0.21646494	FALSE
GB14910	674	373	0.853572961	-0.010944547	FALSE
GB14911	347	184	0.915229897	0.050712388	FALSE
GB14912	NA	NA	NA	NA	FALSE
GB14913	17820	7058	1.336166002	0.471648493	FALSE
GB14914	653	316	1.047158433	0.182640925	FALSE
GB14915	130	67	0.956278623	0.091761114	FALSE
GB14916	2849	1178	1.274116082	0.409598574	FALSE
GB14917	NA	NA	NA	NA	FALSE
GB14918	1452	831	0.805121071	-0.059396437	FALSE
GB14919	89	52	0.775293713	-0.089223796	FALSE
GB14920	13	6	1.115477217	0.250959709	FALSE
GB14921	265	85	1.640457613	0.775940105	TRUE
GB14922	267	112	1.25334101	0.388823501	FALSE
GB14923	743	422	0.816119212	-0.048398296	FALSE
GB14924	190	132	0.525461489	-0.339056019	FALSE

GB14925	407	175	1.217673872	0.353156364	FALSE
GB14926	1635	623	1.391986567	0.527469059	TRUE
GB14927	38	16	1.247927513	0.383410005	FALSE
GB14928	2	0	NA	NA	FALSE
GB14929	1055	593	0.831138989	-0.033378519	FALSE
GB14930	NA	NA	NA	NA	FALSE
GB14931	457	236	0.953407306	0.088889797	FALSE
GB14932	4	1	2	1.135482492	TRUE
GB14933	131	60	1.126532406	0.262014898	FALSE
GB14934	816	539	0.598283879	-0.266233629	FALSE
GB14935	0	1	NA	NA	FALSE
GB14936	252	148	0.767826558	-0.09669095	FALSE
GB14937	462	306	0.594361199	-0.27015631	FALSE
GB14938	10	11	-0.137503524	-1.002021032	TRUE
GB14939	1020	609	0.744055019	-0.120462489	FALSE
GB14940	1	0	NA	NA	FALSE
GB14941	4696	3022	0.635928748	-0.22858876	FALSE
GB14942	NA	NA	NA	NA	FALSE
GB14943	905	539	0.747632519	-0.116884989	FALSE
GB14944	423	271	0.642364812	-0.222152697	FALSE
GB14945	396	196	1.014646776	0.150129268	FALSE
GB14946	216	144	0.584962501	-0.279555008	FALSE
GB14947	8104	5822	0.477119335	-0.387398174	FALSE
GB14948	177	106	0.739685096	-0.124832413	FALSE
GB14949	NA	NA	NA	NA	FALSE
GB14950	450	232	0.955800196	0.091282688	FALSE
GB14951	190	93	1.030696797	0.166179289	FALSE
GB14952	91	48	0.922832139	0.058314631	FALSE
GB14953	2147	1130	0.925999419	0.06148191	FALSE
GB14954	20	11	0.862496476	-0.002021032	FALSE
GB14955	722	441	0.711220181	-0.153297327	FALSE
GB14956	29095	14867	0.96865769	0.104140181	FALSE
GB14957	272	150	0.858644151	-0.005873358	FALSE
GB14958	5095	3122	0.706611609	-0.157905899	FALSE
GB14959	3	1	1.584962501	0.720444992	TRUE
GB14960	1818	1005	0.855156698	-0.00936081	FALSE
GB14961	2008	1393	0.527564011	-0.336953497	FALSE
GB14962	1137	476	1.256198776	0.391681267	FALSE
GB14963	96	62	0.63076619	-0.233751318	FALSE
GB14964	34	17	1	0.135482492	FALSE
GB14965	0	2	NA	NA	FALSE
GB14966	221	118	0.90525951	0.040742002	FALSE
GB14967	12033	5516	1.12530201	0.260784502	FALSE
GB14968	21	13	0.691877705	-0.172639804	FALSE

GB14969	365	219	0.736965594	-0.127551914	FALSE
GB14970	19401	8948	1.116493855	0.251976346	FALSE
GB14971	433	198	1.128866595	0.264349086	FALSE
GB14972	6570	3966	0.728208693	-0.136308815	FALSE
GB14973	909	485	0.906295547	0.041778039	FALSE
GB14974	836	527	0.66569998	-0.198817528	FALSE
GB14975	7323	4068	0.848115117	-0.016402392	FALSE
GB14976	451	238	0.92216586	0.057648352	FALSE
GB14977	561	365	0.620104307	-0.244413201	FALSE
GB14978	NA	NA	NA	NA	FALSE
GB14979	1	0	NA	NA	FALSE
GB14980	4114	2967	0.471536867	-0.392980641	FALSE
GB14981	3752	2191	0.776070344	-0.088447165	FALSE
GB14982	578	301	0.941306006	0.076788497	FALSE
GB14983	172	103	0.739764228	-0.124753281	FALSE
GB14984	309	182	0.763668388	-0.100849121	FALSE
GB14985	1661	970	0.775995421	-0.088522087	FALSE
GB14986	3733	1809	1.045143101	0.180625592	FALSE
GB14987	294	134	1.133583154	0.269065646	FALSE
GB14988	1408	776	0.859518776	-0.004998732	FALSE
GB14989	261	138	0.91938154	0.054864031	FALSE
GB14990	468	251	0.898821166	0.034303657	FALSE
GB14991	620	307	1.01402956	0.149512052	FALSE
GB14992	80	34	1.234465254	0.369947745	FALSE
GB14993	201	135	0.574236094	-0.290281414	FALSE
GB14994	2	0	NA	NA	FALSE
GB14995	232	145	0.678071905	-0.186445603	FALSE
GB14996	NA	NA	NA	NA	FALSE
GB14997	557	259	1.10472523	0.240207721	FALSE
GB14998	542	317	0.773810011	-0.090707497	FALSE
GB14999	299	151	0.985596935	0.121079427	FALSE
GB15000	2373	1032	1.20126913	0.336751621	FALSE
GB15001	140	85	0.719892081	-0.144625428	FALSE
GB15002	14515	8685	0.740946818	-0.12357069	FALSE
GB15003	118	91	0.374848409	-0.489669099	FALSE
GB15004	30	16	0.906890596	0.042373087	FALSE
GB15005	1128	657	0.779801792	-0.084715716	FALSE
GB15006	829	602	0.461608615	-0.402908894	FALSE
GB15007	130	66	0.977973694	0.113456185	FALSE
GB15008	17732	10423	0.766584687	-0.097932822	FALSE
GB15009	534	385	0.471981296	-0.392536212	FALSE
GB15010	229	139	0.720262715	-0.144254793	FALSE
GB15011	1	1	0	-0.864517508	TRUE
GB15012	1610	975	0.723586564	-0.140930944	FALSE

GB15013	509	339	0.586380383	-0.278137125	FALSE
GB15014	1434	805	0.832984336	-0.031533173	FALSE
GB15015	877	576	0.606508031	-0.258009477	FALSE
GB15016	41168	36263	0.18302508	-0.681492429	TRUE
GB15017	829	419	0.984421858	0.119904349	FALSE
GB15018	85420	37906	1.172147673	0.307630164	FALSE
GB15019	160	108	0.567040593	-0.297476916	FALSE
GB15020	423	318	0.411630898	-0.45288661	FALSE
GB15021	3033	1649	0.879154099	0.014636591	FALSE
GB15022	NA	NA	NA	NA	FALSE
GB15023	994	677	0.554090018	-0.31042749	FALSE
GB15024	4551	2632	0.790024097	-0.074493411	FALSE
GB15025	1481	863	0.779139176	-0.085378332	FALSE
GB15026	820	475	0.787696396	-0.076821112	FALSE
GB15027	300	174	0.785875195	-0.078642314	FALSE
GB15028	566	350	0.693447131	-0.171070377	FALSE
GB15029	607	412	0.559052179	-0.305465329	FALSE
GB15030	3525	1640	1.103927443	0.239409934	FALSE
GB15031	1046	683	0.614925368	-0.24959214	FALSE
GB15032	NA	NA	NA	NA	FALSE
GB15033	838	384	1.125843933	0.261326425	FALSE
GB15034	1683	881	0.933821252	0.069303744	FALSE
GB15035	1262	578	1.126570512	0.262053004	FALSE
GB15036	1277	925	0.465233254	-0.399284254	FALSE
GB15037	9	4	1.169925001	0.305407493	FALSE
GB15038	0	4	NA	NA	FALSE
GB15039	16118	12962	0.314384399	-0.550133109	TRUE
GB15040	16	18	-0.169925001	-1.03444251	TRUE
GB15041	28	10	1.485426827	0.620909319	TRUE
GB15042	315	189	0.736965594	-0.127551914	FALSE
GB15043	447	311	0.523360251	-0.341157257	FALSE
GB15044	47024	25033	0.909566068	0.045048559	FALSE
GB15045	711	312	1.188303531	0.323786022	FALSE
GB15046	4961	1727	1.522362874	0.657845366	TRUE
GB15047	519	308	0.752804188	-0.111713321	FALSE
GB15048	306	192	0.672425342	-0.192092166	FALSE
GB15049	29052	13886	1.065006409	0.2004889	FALSE
GB15050	81	28	1.532495081	0.667977572	TRUE
GB15051	1498	766	0.967621327	0.103103818	FALSE
GB15052	3797	3539	0.101518237	-0.762999271	TRUE
GB15053	5051	2791	0.855786917	-0.008730591	FALSE
GB15054	1	2	-1	-1.864517508	TRUE
GB15055	1639040	696867	1.23389582	0.369378312	FALSE
GB15056	47	30	0.647698256	-0.216819252	FALSE

GB15057	19	17	0.160464672	-0.704052836	TRUE
GB15058	2966	1607	0.884148669	0.01963116	FALSE
GB15059	284	147	0.950074775	0.085557266	FALSE
GB15060	60	20	1.584962501	0.720444992	TRUE
GB15061	643	276	1.220150471	0.355632962	FALSE
GB15062	3480	1795	0.955103462	0.090585954	FALSE
GB15063	781	399	0.968933802	0.104416294	FALSE
GB15064	633	292	1.116237131	0.251719622	FALSE
GB15065	47	30	0.647698256	-0.216819252	FALSE
GB15066	812	481	0.755442833	-0.109074675	FALSE
GB15067	2656	1884	0.495456182	-0.369061327	FALSE
GB15068	300	203	0.563482773	-0.301034735	FALSE
GB15069	352	138	1.350907162	0.486389654	FALSE
GB15070	NA	NA	NA	NA	FALSE
GB15071	1768	1062	0.735334509	-0.129183	FALSE
GB15072	1251	872	0.520681749	-0.343835759	FALSE
GB15073	12	4	1.584962501	0.720444992	TRUE
GB15074	1	0	NA	NA	FALSE
GB15075	316	205	0.624300649	-0.24021686	FALSE
GB15076	756	549	0.461580085	-0.402937423	FALSE
GB15077	1451	693	1.066120262	0.201602754	FALSE
GB15078	24	12	1	0.135482492	FALSE
GB15079	6690	3324	1.009085829	0.14456832	FALSE
GB15080	3	3	0	-0.864517508	TRUE
GB15081	264	134	0.978304929	0.113787421	FALSE
GB15082	158	89	0.828047317	-0.036470191	FALSE
GB15083	1053	566	0.895631478	0.03111397	FALSE
GB15084	353	176	1.004092755	0.139575246	FALSE
GB15085	273	116	1.234776146	0.370258637	FALSE
GB15086	1217	763	0.673574206	-0.190943302	FALSE
GB15087	420	298	0.495076997	-0.369440511	FALSE
GB15088	1267	811	0.643642705	-0.220874803	FALSE
GB15089	101	52	0.957771765	0.093254256	FALSE
GB15090	1132	613	0.884914979	0.020397471	FALSE
GB15091	235	185	0.345135486	-0.519382022	TRUE
GB15092	3167	1860	0.767814246	-0.096703262	FALSE
GB15093	1570	775	1.018496344	0.153978835	FALSE
GB15094	146	84	0.797507136	-0.067010372	FALSE
GB15095	412	308	0.419713986	-0.444803522	FALSE
GB15096	1065	697	0.611622869	-0.252894639	FALSE
GB15097	1216	748	0.701033054	-0.163484455	FALSE
GB15098	1579	799	0.982743763	0.118226255	FALSE
GB15099	793	494	0.682809824	-0.181707684	FALSE
GB15100	356	148	1.266280065	0.401762557	FALSE

GB15101	603	321	0.909584705	0.045067196	FALSE
GB15102	3016	1972	0.612976877	-0.251540631	FALSE
GB15103	288	161	0.839008123	-0.025509385	FALSE
GB15104	NA	NA	NA	NA	FALSE
GB15105	197	130	0.599684006	-0.264833502	FALSE
GB15106	97	56	0.79255792	-0.071959588	FALSE
GB15107	408	241	0.759536006	-0.104981503	FALSE
GB15108	1781	969	0.878118946	0.013601437	FALSE
GB15109	514	268	0.939535359	0.07501785	FALSE
GB15110	882	454	0.958086358	0.09356885	FALSE
GB15111	1194	556	1.102646049	0.23812854	FALSE
GB15112	0	1	NA	NA	FALSE
GB15113	16675	8864	0.911656975	0.047139467	FALSE
GB15114	130	69	0.913843356	0.049325848	FALSE
GB15115	661	321	1.042076974	0.177559466	FALSE
GB15116	884	445	0.990241034	0.125723525	FALSE
GB15117	15	1	3.906890596	3.042373087	TRUE
GB15118	11	4	1.459431619	0.59491411	TRUE
GB15119	978	637	0.618541093	-0.245976416	FALSE
GB15120	1428	969	0.559427409	-0.3050901	FALSE
GB15121	2995	1705	0.812784264	-0.051733245	FALSE
GB15122	139	77	0.852154532	-0.012362976	FALSE
GB15123	379	203	0.900718121	0.036200613	FALSE
GB15124	646	419	0.624583921	-0.239933587	FALSE
GB15125	12	3	2	1.135482492	TRUE
GB15126	7	4	0.807354922	-0.057162586	FALSE
GB15127	1	3	-1.584962501	-2.449480009	TRUE
GB15128	277	133	1.058459731	0.193942222	FALSE
GB15129	3	5	-0.736965594	-1.601483102	TRUE
GB15130	660	403	0.711686186	-0.152831323	FALSE
GB15131	706	320	1.141596278	0.27707877	FALSE
GB15132	1193	709	0.75073651	-0.113780998	FALSE
GB15133	2345	1261	0.895019647	0.030502139	FALSE
GB15134	1271	655	0.956397219	0.09187971	FALSE
GB15135	12	19	-0.662965013	-1.527482521	TRUE
GB15136	236	143	0.722771713	-0.141745796	FALSE
GB15137	5658	3364	0.750114471	-0.114403037	FALSE
GB15138	43	40	0.10433666	-0.760180849	TRUE
GB15139	595	382	0.63931703	-0.225200478	FALSE
GB15140	784	499	0.651813839	-0.21270367	FALSE
GB15141	1590	891	0.835529429	-0.02898808	FALSE
GB15142	4302	2793	0.623191951	-0.241325557	FALSE
GB15143	9839	7666	0.360037695	-0.504479814	TRUE
GB15144	5	5	0	-0.864517508	TRUE

GB15145	102	76	0.424497829	-0.44001968	FALSE
GB15146	4	2	1	0.135482492	FALSE
GB15147	1062	625	0.764855671	-0.099661837	FALSE
GB15148	1518	943	0.686842115	-0.177675394	FALSE
GB15149	174	105	0.728697978	-0.13581953	FALSE
GB15150	1050	494	1.087806381	0.223288873	FALSE
GB15151	118	87	0.439699554	-0.424817955	FALSE
GB15152	579	311	0.896648768	0.03213126	FALSE
GB15153	351	224	0.647972298	-0.21654521	FALSE
GB15154	330	215	0.618129365	-0.246388144	FALSE
GB15155	265	96	1.464886049	0.60036854	TRUE
GB15156	237	150	0.659924558	-0.20459295	FALSE
GB15157	4194	2404	0.802889965	-0.061627543	FALSE
GB15158	108	70	0.625604485	-0.238913023	FALSE
GB15159	136	97	0.487549999	-0.376967509	FALSE
GB15160	310	176	0.816692787	-0.047824722	FALSE
GB15161	662	387	0.774497651	-0.090019858	FALSE
GB15162	54	48	0.169925001	-0.694592507	TRUE
GB15163	9366	4185	1.162205415	0.297687907	FALSE
GB15164	270	121	1.15795236	0.293434851	FALSE
GB15165	449	298	0.591403114	-0.273114394	FALSE
GB15166	518	206	1.330307761	0.465790252	FALSE
GB15167	2350	1356	0.793303578	-0.07121393	FALSE
GB15168	3229	1695	0.929802168	0.065284659	FALSE
GB15169	2245	926	1.277631346	0.413113838	FALSE
GB15170	1	0	NA	NA	FALSE
GB15171	25	27	-0.111031312	-0.975548821	TRUE
GB15172	4661	2975	0.647749844	-0.216767664	FALSE
GB15173	655	469	0.481906984	-0.382610524	FALSE
GB15174	17	6	1.502500341	0.637982832	TRUE
GB15175	88	42	1.067114196	0.202596688	FALSE
GB15176	2622	1262	1.054955775	0.190438267	FALSE
GB15177	198	90	1.137503524	0.272986015	FALSE
GB15178	1309	770	0.765534746	-0.098982762	FALSE
GB15179	3	0	NA	NA	FALSE
GB15180	167	100	0.739848103	-0.124669406	FALSE
GB15181	10	6	0.736965594	-0.127551914	FALSE
GB15182	193	118	0.709813988	-0.15470352	FALSE
GB15183	265	143	0.889977213	0.025459704	FALSE
GB15184	1584	865	0.872800298	0.008282789	FALSE
GB15185	637	331	0.944462155	0.079944647	FALSE
GB15186	2736	1539	0.830074999	-0.03444251	FALSE
GB15187	492	243	1.017702002	0.153184493	FALSE
GB15188	185	195	-0.075948853	-0.940466362	TRUE

GB15189	1697	1164	0.543895507	-0.320622002	FALSE
GB15190	534	361	0.564840905	-0.299676604	FALSE
GB15191	1139	685	0.733591854	-0.130925654	FALSE
GB15192	837	415	1.012116286	0.147598778	FALSE
GB15193	637	443	0.523986674	-0.340530835	FALSE
GB15194	452	287	0.655272036	-0.209245473	FALSE
GB15195	277	81	1.773892163	0.909374655	TRUE
GB15196	51	23	1.148863386	0.284345878	FALSE
GB15197	78	53	0.557481764	-0.307035744	FALSE
GB15198	932	410	1.184706045	0.320188537	FALSE
GB15199	35	23	0.605721061	-0.258796447	FALSE
GB15200	329	131	1.328520772	0.464003264	FALSE
GB15201	136	88	0.628031223	-0.236486286	FALSE
GB15202	2250	1506	0.579203231	-0.285314277	FALSE
GB15203	401	184	1.12389647	0.259378962	FALSE
GB15204	1328	732	0.859339593	-0.005177915	FALSE
GB15205	439	250	0.812292845	-0.052224663	FALSE
GB15206	NA	NA	NA	NA	FALSE
GB15207	1454	896	0.698456632	-0.166060876	FALSE
GB15208	158	84	0.911463325	0.046945817	FALSE
GB15209	683	358	0.931925991	0.067408483	FALSE
GB15210	200	139	0.524915117	-0.339602391	FALSE
GB15211	NA	NA	NA	NA	FALSE
GB15212	700	447	0.647080091	-0.217437418	FALSE
GB15213	81	29	1.481869008	0.617351499	TRUE
GB15214	315	192	0.714245518	-0.150271991	FALSE
GB15215	260	121	1.103504576	0.238987067	FALSE
GB15216	27	13	1.054447784	0.189930276	FALSE
GB15217	4124	2561	0.68733708	-0.177180429	FALSE
GB15218	5331	3363	0.664657403	-0.199860105	FALSE
GB15219	327	203	0.687810908	-0.1767066	FALSE
GB15220	7434	3675	1.016394438	0.15187693	FALSE
GB15221	1070	513	1.060580066	0.196062557	FALSE
GB15222	NA	NA	NA	NA	FALSE
GB15223	89	64	0.475733431	-0.388784077	FALSE
GB15224	1249	593	1.074669467	0.210151959	FALSE
GB15225	15748	10256	0.618700451	-0.245817058	FALSE
GB15226	2131	1179	0.853966875	-0.010550634	FALSE
GB15227	1077	606	0.829628551	-0.034888957	FALSE
GB15228	NA	NA	NA	NA	FALSE
GB15229	1488	812	0.873822894	0.009305386	FALSE
GB15230	20459	8618	1.247310627	0.382793119	FALSE
GB15231	1050	603	0.800159421	-0.064358088	FALSE
GB15232	558	267	1.06342538	0.198907872	FALSE

GB15233	100837	45246	1.156162942	0.291645433	FALSE
GB15234	337	200	0.752748591	-0.111768917	FALSE
GB15235	8	2	2	1.135482492	TRUE
GB15236	169	131	0.367456435	-0.497061074	FALSE
GB15237	11	7	0.652076697	-0.212440812	FALSE
GB15238	345	200	0.786596362	-0.077921146	FALSE
GB15239	3580	1997	0.842125255	-0.022392254	FALSE
GB15240	111	63	0.817135943	-0.047381565	FALSE
GB15241	618	359	0.783622994	-0.080894514	FALSE
GB15242	1235	615	1.005852726	0.141335218	FALSE
GB15243	701	463	0.598402251	-0.266115258	FALSE
GB15244	535	306	0.806007239	-0.05851027	FALSE
GB15245	1937	1258	0.622692032	-0.241825477	FALSE
GB15246	44	20	1.137503524	0.272986015	FALSE
GB15247	0	1	NA	NA	FALSE
GB15248	170	85	1	0.135482492	FALSE
GB15249	482	239	1.012022528	0.14750502	FALSE
GB15250	1	1	0	-0.864517508	TRUE
GB15251	NA	NA	NA	NA	FALSE
GB15252	1366	931	0.553104411	-0.311413098	FALSE
GB15253	115	68	0.75802721	-0.106490299	FALSE
GB15254	9839	4519	1.122508136	0.257990627	FALSE
GB15255	2426	1313	0.885712634	0.021195126	FALSE
GB15256	0	1	NA	NA	FALSE
GB15257	914	438	1.061263295	0.196745787	FALSE
GB15258	292	141	1.050273206	0.185755698	FALSE
GB15259	4	5	-0.321928095	-1.186445603	TRUE
GB15260	144	68	1.08246216	0.217944652	FALSE
GB15261	35092	23651	0.569240989	-0.295276519	FALSE
GB15262	92	52	0.823122238	-0.04139527	FALSE
GB15263	566	270	1.067842646	0.203325137	FALSE
GB15264	0	1	NA	NA	FALSE
GB15265	3810	2223	0.777283049	-0.087234459	FALSE
GB15266	679	348	0.964324268	0.09980676	FALSE
GB15267	328	193	0.765094967	-0.099422541	FALSE
GB15268	36	5	2.847996907	1.983479398	TRUE
GB15269	1601	908	0.818209105	-0.046308403	FALSE
GB15270	471	261	0.851677253	-0.012840255	FALSE
GB15271	1063	614	0.791831036	-0.072686472	FALSE
GB15272	6217	3157	0.977664318	0.11314681	FALSE
GB15273	187	90	1.055041364	0.190523855	FALSE
GB15274	1125	640	0.813781191	-0.050736317	FALSE
GB15275	397	330	0.266672983	-0.597844525	TRUE
GB15276	110	80	0.459431619	-0.40508589	FALSE

GB15277	2663	1505	0.823288942	-0.041228567	FALSE
GB15278	261	137	0.929873914	0.065356405	FALSE
GB15279	249	164	0.602449927	-0.262067581	FALSE
GB15280	147	85	0.790281409	-0.0742361	FALSE
GB15281	6579	3205	1.037543956	0.173026448	FALSE
GB15282	82	38	1.109624491	0.245106983	FALSE
GB15283	1991	1285	0.631724862	-0.232792647	FALSE
GB15284	1	5	-2.321928095	-3.186445603	TRUE
GB15285	4778	2783	0.779765945	-0.084751563	FALSE
GB15286	458	205	1.159723689	0.29520618	FALSE
GB15287	608	339	0.84278605	-0.021731458	FALSE
GB15288	353	172	1.037259619	0.17274211	FALSE
GB15289	1593	961	0.729137931	-0.135379578	FALSE
GB15290	205	136	0.592017258	-0.27250025	FALSE
GB15291	5805	3579	0.697739523	-0.166777985	FALSE
GB15292	2	0	NA	NA	FALSE
GB15293	96	42	1.192645078	0.32812757	FALSE
GB15294	28	9	1.637429921	0.772912412	TRUE
GB15295	248	90	1.462343214	0.597825706	TRUE
GB15296	367	242	0.600773016	-0.263744493	FALSE
GB15297	93	56	0.731803889	-0.132713619	FALSE
GB15298	282	174	0.696607857	-0.167909652	FALSE
GB15299	6359	2933	1.116422838	0.251905329	FALSE
GB15300	956	465	1.039779902	0.175262394	FALSE
GB15301	3550	1787	0.99027939	0.125761882	FALSE
GB15302	NA	NA	NA	NA	FALSE
GB15303	14	6	1.222392421	0.357874913	FALSE
GB15304	5231	2948	0.827350246	-0.037167263	FALSE
GB15305	7	5	0.485426827	-0.379090681	FALSE
GB15306	1	0	NA	NA	FALSE
GB15307	4253	2156	0.980123677	0.115606169	FALSE
GB15308	394	217	0.860500587	-0.004016921	FALSE
GB15309	141	102	0.46712601	-0.397391498	FALSE
GB15310	1765	1088	0.697989627	-0.166527881	FALSE
GB15311	2317	1140	1.02322422	0.158706711	FALSE
GB15312	699	519	0.429557917	-0.434959591	FALSE
GB15313	74	56	0.402098444	-0.462419065	FALSE
GB15314	1380	711	0.956746802	0.092229294	FALSE
GB15315	667	326	1.032814797	0.168297289	FALSE
GB15316	844	389	1.117472844	0.252955335	FALSE
GB15317	1316	738	0.834466768	-0.030050741	FALSE
GB15318	711	477	0.575860294	-0.288657215	FALSE
GB15319	176	106	0.731511164	-0.133006344	FALSE
GB15320	2103	1415	0.571646797	-0.292870711	FALSE

GB15321	244	152	0.682809824	-0.181707684	FALSE
GB15322	1	1	0	-0.864517508	TRUE
GB15323	559	338	0.725825037	-0.138692472	FALSE
GB15324	180	146	0.302028537	-0.562488971	TRUE
GB15325	826	393	1.071612469	0.207094961	FALSE
GB15326	1006	626	0.684395743	-0.180121765	FALSE
GB15327	181043	125363	0.530220787	-0.334296722	FALSE
GB15328	137	56	1.290677161	0.426159653	FALSE
GB15329	368	231	0.671812915	-0.192704594	FALSE
GB15330	1152	483	1.254045623	0.389528114	FALSE
GB15331	4852	2896	0.744517948	-0.11999956	FALSE
GB15332	341	227	0.587079442	-0.277438067	FALSE
GB15333	4165	2587	0.687036442	-0.177481067	FALSE
GB15334	562	340	0.725035384	-0.139482124	FALSE
GB15335	1849	1131	0.709146295	-0.155371213	FALSE
GB15336	1401	793	0.821064185	-0.043453324	FALSE
GB15337	89	43	1.049468676	0.184951168	FALSE
GB15338	508	279	0.864563375	[illegible]666156444298e-05	FALSE
GB15339	1553	822	0.917847531	0.053330022	FALSE
GB15340	683	396	0.786385148	-0.07813236	FALSE
GB15341	625	380	0.717856771	-0.146660737	FALSE
GB15342	16	15	0.093109404	-0.771408104	TRUE
GB15343	516	338	0.610347819	-0.254169689	FALSE
GB15344	7069	3480	1.022418836	0.157901327	FALSE
GB15345	5676	2436	1.220360456	0.355842948	FALSE
GB15346	692	375	0.883881442	0.019363934	FALSE
GB15347	1048	585	0.841130187	-0.023387321	FALSE
GB15348	367	226	0.69945729	-0.165060218	FALSE
GB15349	648	447	0.535718982	-0.328798527	FALSE
GB15350	450	185	1.282399731	0.417882222	FALSE
GB15351	1445	820	0.817373678	-0.04714383	FALSE
GB15352	211	141	0.581547836	-0.282969672	FALSE
GB15353	1688	972	0.796286685	-0.068230823	FALSE
GB15354	14	11	0.347923303	-0.516594205	TRUE
GB15355	1711	1051	0.703077091	-0.161440418	FALSE
GB15356	1285	705	0.866073197	0.001555688	FALSE
GB15357	477	235	1.021328509	0.156811001	FALSE
GB15358	97	60	0.693022247	-0.171495262	FALSE
GB15359	4879	2613	0.900878359	0.03636085	FALSE
GB15360	689	420	0.714114655	-0.150402853	FALSE
GB15361	14946	9097	0.716296669	-0.148220839	FALSE
GB15362	1212	794	0.610178786	-0.254338722	FALSE
GB15363	1449	754	0.942421166	0.077903658	FALSE
GB15364	47	29	0.696607857	-0.167909652	FALSE

GB15365	3	1	1.584962501	0.720444992	TRUE
GB15366	NA	NA	NA	NA	FALSE
GB15367	122	66	0.886343218	0.02182571	FALSE
GB15368	159	116	0.45490196	-0.409615548	FALSE
GB15369	2	0	NA	NA	FALSE
GB15370	333	223	0.578478467	-0.286039041	FALSE
GB15371	480	237	1.018147347	0.153629838	FALSE
GB15372	1090	693	0.653400878	-0.211116631	FALSE
GB15373	165	114	0.5334322	-0.331085308	FALSE
GB15374	703	380	0.887525271	0.023007762	FALSE
GB15375	12457	8072	0.625958589	-0.23855892	FALSE
GB15376	447	191	1.226702193	0.362184685	FALSE
GB15377	344	233	0.56207861	-0.302438898	FALSE
GB15378	1256	572	1.134749412	0.270231904	FALSE
GB15379	833	390	1.094842372	0.230324863	FALSE
GB15380	112	40	1.485426827	0.620909319	TRUE
GB15381	25	8	1.64385619	0.779338681	TRUE
GB15382	994	487	1.029324079	0.164806571	FALSE
GB15383	175	136	0.363748271	-0.500769238	TRUE
GB15384	88	51	0.787006277	-0.077511232	FALSE
GB15385	701	453	0.629903394	-0.234614114	FALSE
GB15386	2849	1515	0.911137828	0.04662032	FALSE
GB15387	1699	1069	0.668423999	-0.196093509	FALSE
GB15388	201	110	0.869691978	0.005174469	FALSE
GB15389	213	124	0.78051331	-0.084004198	FALSE
GB15390	0	3	NA	NA	FALSE
GB15391	512	306	0.742612157	-0.121905351	FALSE
GB15392	705	492	0.518964942	-0.345552566	FALSE
GB15393	311	149	1.06160225	0.197084741	FALSE
GB15394	173	124	0.480431917	-0.384085591	FALSE
GB15395	199	128	0.636624621	-0.227892888	FALSE
GB15396	508	307	0.726589841	-0.137927667	FALSE
GB15397	406	254	0.67665123	-0.187866278	FALSE
GB15398	1382	621	1.154092442	0.289574934	FALSE
GB15399	987	697	0.501891429	-0.36262608	FALSE
GB15400	381	293	0.378890333	-0.485627175	FALSE
GB15401	423	241	0.811624517	-0.052892991	FALSE
GB15402	243	124	0.970616193	0.106098685	FALSE
GB15403	1	2	-1	-1.864517508	TRUE
GB15404	677	436	0.634827699	-0.22968981	FALSE
GB15405	421	256	0.717676423	-0.146841085	FALSE
GB15406	1886	1279	0.560313412	-0.304204097	FALSE
GB15407	527	270	0.964843555	0.100326046	FALSE
GB15408	7998	4794	0.738409372	-0.126108136	FALSE

GB15409	1952	2004	-0.037929456	-0.902446964	TRUE
GB15410	938	980	-0.063193826	-0.927711335	TRUE
GB15411	8	8	0	-0.864517508	TRUE
GB15412	1084	504	1.104869118	0.24035161	FALSE
GB15413	16	6	1.415037499	0.550519991	TRUE
GB15414	3484	1941	0.843944506	-0.020573002	FALSE
GB15415	2	0	NA	NA	FALSE
GB15416	1194	714	0.741806857	-0.122710651	FALSE
GB15417	69	25	1.464668267	0.600150759	TRUE
GB15418	336	284	0.242570303	-0.621947205	TRUE
GB15419	843	458	0.880185033	0.015667525	FALSE
GB15420	829	431	0.943684232	0.079166724	FALSE
GB15421	448	234	0.936990202	0.072472694	FALSE
GB15422	202	154	0.391424942	-0.473092566	FALSE
GB15423	1623	869	0.901234918	0.036717409	FALSE
GB15424	36	22	0.710493383	-0.154024126	FALSE
GB15425	NA	NA	NA	NA	FALSE
GB15426	6	1	2.584962501	1.720444992	TRUE
GB15427	1	2	-1	-1.864517508	TRUE
GB15428	27356	12717	1.105098922	0.240581414	FALSE
GB15429	96	59	0.702319451	-0.162198057	FALSE
GB15430	16	13	0.299560282	-0.564957226	TRUE
GB15431	2268	1366	0.731463157	-0.133054352	FALSE
GB15432	339	176	0.945709844	0.081192336	FALSE
GB15433	92	47	0.968973104	0.104455596	FALSE
GB15434	10	3	1.736965594	0.872448086	TRUE
GB15435	373	191	0.965602992	0.101085484	FALSE
GB15436	1148	739	0.635476373	-0.229041136	FALSE
GB15437	2858	1537	0.894888751	0.030371243	FALSE
GB15438	335	189	0.825774861	-0.038742647	FALSE
GB15439	650	379	0.77824187	-0.086275639	FALSE
GB15440	747	536	0.478875242	-0.385642266	FALSE
GB15441	733	354	1.050063838	0.18554633	FALSE
GB15442	123	82	0.584962501	-0.279555008	FALSE
GB15443	21670	13686	0.662998301	-0.201519207	FALSE
GB15444	12	4	1.584962501	0.720444992	TRUE
GB15445	40	26	0.621488377	-0.243029132	FALSE
GB15446	6132	2860	1.10034255	0.235825042	FALSE
GB15447	663	325	1.028569152	0.164051644	FALSE
GB15448	899	578	0.637251623	-0.227265885	FALSE
GB15449	493	204	1.273018494	0.408500986	FALSE
GB15450	1262	688	0.87523144	0.010713932	FALSE
GB15451	1829	902	1.019855736	0.155338228	FALSE
GB15452	1540	994	0.631612594	-0.232904914	FALSE

GB15453	2932	1453	1.012850401	0.148332892	FALSE
GB15454	568	285	0.99492901	0.130411502	FALSE
GB15455	273	171	0.674904626	-0.189612882	FALSE
GB15456	17	8	1.087462841	0.222945333	FALSE
GB15457	3519	2117	0.733144245	-0.131373264	FALSE
GB15458	148	93	0.670294555	-0.194222954	FALSE
GB15459	10	5	1	0.135482492	FALSE
GB15460	6	3	1	0.135482492	FALSE
GB15461	787	496	0.666023515	-0.198493993	FALSE
GB15462	4	4	0	-0.864517508	TRUE
GB15463	178	122	0.544996093	-0.319521415	FALSE
GB15464	2668	1449	0.880701072	0.016183563	FALSE
GB15465	593	418	0.504529162	-0.359988346	FALSE
GB15466	281	129	1.123199065	0.258681556	FALSE
GB15467	94	34	1.46712601	0.602608502	TRUE
GB15468	5397	3154	0.774975027	-0.089542481	FALSE
GB15469	14	4	1.807354922	0.942837414	TRUE
GB15470	736	436	0.755377631	-0.109139877	FALSE
GB15471	439	253	0.795083555	-0.069433953	FALSE
GB15472	1170	715	0.710493383	-0.154024126	FALSE
GB15473	5	2	1.321928095	0.457410587	FALSE
GB15474	166	104	0.674599713	-0.189917795	FALSE
GB15475	826	578	0.515072289	-0.349445219	FALSE
GB15476	660	313	1.076303367	0.211785859	FALSE
GB15477	122	82	0.573185333	-0.291332175	FALSE
GB15478	1947	946	1.041340795	0.176823287	FALSE
GB15479	96	36	1.415037499	0.550519991	TRUE
GB15480	451	252	0.8397037	-0.024813809	FALSE
GB15481	739	396	0.900073934	0.035556426	FALSE
GB15482	2	2	0	-0.864517508	TRUE
GB15483	325	135	1.267480311	0.402962803	FALSE
GB15484	267	176	0.601264313	-0.263253195	FALSE
GB15485	2004	968	1.049803556	0.185286048	FALSE
GB15486	2630	1980	0.409562369	-0.454955139	FALSE
GB15487	3189	1887	0.757009675	-0.107507834	FALSE
GB15488	222	146	0.604591307	-0.259926201	FALSE
GB15489	1	0	NA	NA	FALSE
GB15490	5	0	NA	NA	FALSE
GB15491	178	99	0.846376811	-0.018140697	FALSE
GB15492	233	131	0.830763143	-0.033754365	FALSE
GB15493	183	96	0.930737338	0.066219829	FALSE
GB15494	26533	11988	1.146196817	0.281679308	FALSE
GB15495	336	184	0.868755467	0.004237958	FALSE
GB15496	937	512	0.871905238	0.007387729	FALSE

GB15497	0	1	NA	NA	FALSE
GB15498	18128	9299	0.963072282	0.098554773	FALSE
GB15499	1220	613	0.992922169	0.12840466	FALSE
GB15500	2602	1681	0.630301237	-0.234216271	FALSE
GB15501	44	41	0.101879614	-0.762637894	TRUE
GB15502	33	13	1.343954401	0.479436893	FALSE
GB15503	55195	37010	0.576622447	-0.287895062	FALSE
GB15504	5	6	-0.263034406	-1.127551914	TRUE
GB15505	NA	NA	NA	NA	FALSE
GB15506	11709	5816	1.009518694	0.145001186	FALSE
GB15507	70	47	0.574694165	-0.289823343	FALSE
GB15508	841	528	0.671567871	-0.192949637	FALSE
GB15509	944	382	1.305214221	0.440696713	FALSE
GB15510	1	0	NA	NA	FALSE
GB15511	994	601	0.725880861	-0.138636647	FALSE
GB15512	1512	911	0.73093518	-0.133582328	FALSE
GB15513	177	79	1.163824802	0.299307294	FALSE
GB15514	94	59	0.671945802	-0.192571706	FALSE
GB15515	166	91	0.867244791	0.002727283	FALSE
GB15516	183	106	0.787779384	-0.076738125	FALSE
GB15517	412	288	0.516575526	-0.347941983	FALSE
GB15518	4820	2022	1.253250149	0.388732641	FALSE
GB15519	77	29	1.408805546	0.544288037	TRUE
GB15520	3489	1838	0.924676831	0.060159323	FALSE
GB15521	367	227	0.693087766	-0.171429743	FALSE
GB15522	647	358	0.853806125	-0.010711384	FALSE
GB15523	32	37	-0.209453366	-1.073970874	TRUE
GB15524	1463	1122	0.382857094	-0.481660415	FALSE
GB15525	2360	1936	0.285707907	-0.578809601	TRUE
GB15526	1357	698	0.959121779	0.094604271	FALSE
GB15527	410	204	1.007054758	0.142537249	FALSE
GB15528	463	161	1.523951505	0.659433997	TRUE
GB15529	99	35	1.500073603	0.635556095	TRUE
GB15530	5	7	-0.485426827	-1.349944335	TRUE
GB15531	16	16	0	-0.864517508	TRUE
GB15532	1169	565	1.048952157	0.184434649	FALSE
GB15533	214	114	0.908576972	0.044059464	FALSE
GB15534	NA	NA	NA	NA	FALSE
GB15535	518	281	0.882381967	0.017864459	FALSE
GB15536	171	63	1.440572591	0.576055083	TRUE
GB15537	2647	1404	0.914815259	0.050297751	FALSE
GB15538	411	201	1.031942893	0.167425384	FALSE
GB15539	674	389	0.792978436	-0.071539072	FALSE
GB15540	46	32	0.523561956	-0.340955552	FALSE

GB15541	2214	1363	0.69986966	-0.164647848	FALSE
GB15542	569	307	0.890189997	0.025672489	FALSE
GB15543	199	107	0.895157634	0.030640126	FALSE
GB15544	91	67	0.44170545	-0.422812059	FALSE
GB15545	395	233	0.761522698	-0.10299481	FALSE
GB15546	880	499	0.818463708	-0.0460538	FALSE
GB15547	488	345	0.500284786	-0.364232722	FALSE
GB15548	101	48	1.073248982	0.208731474	FALSE
GB15549	102	39	1.387023123	0.522505615	TRUE
GB15550	21	8	1.392317423	0.527799914	TRUE
GB15551	80	53	0.59400764	-0.270509868	FALSE
GB15552	0	1	NA	NA	FALSE
GB15553	1477	876	0.753667051	-0.110850457	FALSE
GB15554	2	3	-0.584962501	-1.449480009	TRUE
GB15555	79	35	1.174497731	0.309980223	FALSE
GB15556	1	1	0	-0.864517508	TRUE
GB15557	0	1	NA	NA	FALSE
GB15558	1653	996	0.730869077	-0.133648431	FALSE
GB15559	60	29	1.0489096	0.184392092	FALSE
GB15560	1374	803	0.774910111	-0.089607397	FALSE
GB15561	56	25	1.163498732	0.298981224	FALSE
GB15562	2	2	0	-0.864517508	TRUE
GB15563	53	25	1.084064265	0.219546756	FALSE
GB15564	13	10	0.378511623	-0.486005885	FALSE
GB15565	2168	1641	0.401789518	-0.46272799	FALSE
GB15566	48	10	2.263034406	1.398516898	TRUE
GB15567	182	150	0.27897595	-0.585541559	TRUE
GB15568	1161	597	0.959565136	0.095047627	FALSE
GB15569	16307	6487	1.32986805	0.465350541	FALSE
GB15570	96	46	1.061400545	0.196883036	FALSE
GB15571	2	2	0	-0.864517508	TRUE
GB15572	32	17	0.912537159	0.04801965	FALSE
GB15573	78	23	1.761840263	0.897322754	TRUE
GB15574	364	205	0.828314541	-0.036202968	FALSE
GB15575	2287	1385	0.723570389	-0.140947119	FALSE
GB15576	42	23	0.868755467	0.004237958	FALSE
GB15577	240	107	1.165423609	0.300906101	FALSE
GB15578	2582	1378	0.905913113	0.041395604	FALSE
GB15579	810	374	1.114883638	0.25036613	FALSE
GB15580	34	13	1.387023123	0.522505615	TRUE
GB15581	10	3	1.736965594	0.872448086	TRUE
GB15582	26709	14694	0.862098782	-0.002418726	FALSE
GB15583	83	53	0.647118977	-0.217398532	FALSE
GB15584	221	110	1.006542846	0.142025338	FALSE

GB15585	255	177	0.526747887	-0.337769622	FALSE
GB15586	16	7	1.192645078	0.32812757	FALSE
GB15587	120	53	1.178970141	0.314452633	FALSE
GB15588	24	16	0.584962501	-0.279555008	FALSE
GB15589	12	5	1.263034406	0.398516898	FALSE
GB15590	337	172	0.970340026	0.105822518	FALSE
GB15591	53	31	0.773724144	-0.090793364	FALSE
GB15592	313	228	0.457128833	-0.407388676	FALSE
GB15593	1162	609	0.932095935	0.067578427	FALSE
GB15594	20	13	0.621488377	-0.243029132	FALSE
GB15595	43	34	0.338801913	-0.525715595	TRUE
GB15596	3449	1637	1.075123807	0.210606299	FALSE
GB15597	631	295	1.096925051	0.232407542	FALSE
GB15598	976	484	1.0118741	0.147356592	FALSE
GB15599	34	19	0.839535328	-0.024982181	FALSE
GB15600	155	103	0.589623878	-0.27489363	FALSE
GB15601	1217	893	0.446597088	-0.417920421	FALSE
GB15602	631	394	0.679444376	-0.185073133	FALSE
GB15603	526	346	0.604290762	-0.260226747	FALSE
GB15604	1387	652	1.089023918	0.22450641	FALSE
GB15605	16181	10771	0.587148572	-0.277368937	FALSE
GB15606	1130	563	1.005115945	0.140598437	FALSE
GB15607	18	3	2.584962501	1.720444992	TRUE
GB15608	159	72	1.142957954	0.278440446	FALSE
GB15609	881	618	0.511535181	-0.352982327	FALSE
GB15610	52	29	0.842458723	-0.022058785	FALSE
GB15611	24543	11679	1.071394856	0.206877348	FALSE
GB15612	2	0	NA	NA	FALSE
GB15613	4833	3604	0.423319984	-0.441197525	FALSE
GB15614	388	158	1.296132094	0.431614586	FALSE
GB15615	NA	NA	NA	NA	FALSE
GB15616	38	46	-0.275634443	-1.140151951	TRUE
GB15617	177	59	1.584962501	0.720444992	TRUE
GB15618	259	158	0.71302754	-0.151489969	FALSE
GB15619	32764	29854	0.134187255	-0.730330253	TRUE
GB15620	1475	781	0.917320501	0.052802993	FALSE
GB15621	1332	666	1	0.135482492	FALSE
GB15622	274	197	0.475980264	-0.388537245	FALSE
GB15623	280	182	0.621488377	-0.243029132	FALSE
GB15624	581	331	0.811706947	-0.052810562	FALSE
GB15625	340	237	0.520647687	-0.343869821	FALSE
GB15626	387	229	0.756985968	-0.10753154	FALSE
GB15627	302	89	1.762671308	0.8981538	TRUE
GB15628	1029	569	0.854742425	-0.009775084	FALSE

GB15629	3477	2261	0.620882075	-0.243635433	FALSE
GB15630	461	231	0.996873899	0.132356391	FALSE
GB15631	245	134	0.870548749	0.00603124	FALSE
GB15632	22	9	1.289506617	0.424989109	FALSE
GB15633	4936	2646	0.899529333	0.035011825	FALSE
GB15634	5360	2654	1.01406463	0.149547122	FALSE
GB15635	1114	467	1.254254778	0.389737269	FALSE
GB15636	63	51	0.304854582	-0.559662927	TRUE
GB15637	35363	23964	0.561371932	-0.303145577	FALSE
GB15638	1000	604	0.727379545	-0.137137963	FALSE
GB15639	375	230	0.705256734	-0.159260774	FALSE
GB15640	1714	757	1.179001904	0.314484396	FALSE
GB15641	380	210	0.855610091	-0.008907418	FALSE
GB15642	2	3	-0.584962501	-1.449480009	TRUE
GB15643	393	221	0.830482943	-0.034034565	FALSE
GB15644	1067	516	1.048117205	0.183599697	FALSE
GB15645	4613	1924	1.261596493	0.397078985	FALSE
GB15646	68	34	1	0.135482492	FALSE
GB15647	1393	743	0.906761142	0.042243634	FALSE
GB15648	18565	12857	0.530031266	-0.334486242	FALSE
GB15649	56	35	0.678071905	-0.186445603	FALSE
GB15650	17	23	-0.436099115	-1.300616623	TRUE
GB15651	75	38	0.980891177	0.116373669	FALSE
GB15652	7236	3749	0.948686582	0.084169074	FALSE
GB15653	351	120	1.548436625	0.683919116	TRUE
GB15654	15	8	0.906890596	0.042373087	FALSE
GB15655	903	531	0.766014127	-0.098503382	FALSE
GB15656	3840	1999	0.941827839	0.077310331	FALSE
GB15657	18	7	1.362570079	0.498052571	FALSE
GB15658	921	533	0.789065623	-0.075451885	FALSE
GB15659	1138	645	0.819129492	-0.045388016	FALSE
GB15660	17619	11291	0.641958778	-0.22255873	FALSE
GB15661	369	199	0.890852386	0.026334877	FALSE
GB15662	1372	774	0.82587501	-0.038642498	FALSE
GB15663	16	12	0.415037499	-0.449480009	FALSE
GB15664	20685	10463	0.98328839	0.118770882	FALSE
GB15665	203	67	1.599246727	0.734729218	TRUE
GB15666	209	139	0.588418059	-0.276099449	FALSE
GB15667	110	61	0.850622376	-0.013895132	FALSE
GB15668	266	158	0.751501687	-0.113015821	FALSE
GB15669	4187	2275	0.880050373	0.015532864	FALSE
GB15670	5	1	2.321928095	1.457410587	TRUE
GB15671	10550	5540	0.929285118	0.064767609	FALSE
GB15672	345	121	1.511589314	0.647071806	TRUE

GB15673	1689	695	1.281084445	0.416566937	FALSE
GB15674	1834	1232	0.573991383	-0.290526125	FALSE
GB15675	1625	918	0.823873659	-0.040643849	FALSE
GB15676	994	374	1.410207582	0.545690073	TRUE
GB15677	877	551	0.670524524	-0.193992984	FALSE
GB15678	75	47	0.674229839	-0.19028767	FALSE
GB15679	245	117	1.066273219	0.201755711	FALSE
GB15680	728	480	0.600904045	-0.263613464	FALSE
GB15681	4030	1295	1.637827741	0.773310233	TRUE
GB15682	1291	676	0.933393849	0.068876341	FALSE
GB15683	1	1	0	-0.864517508	TRUE
GB15684	400	287	0.478949263	-0.385568245	FALSE
GB15685	750	391	0.939721988	0.07520448	FALSE
GB15686	211	101	1.062887706	0.198370198	FALSE
GB15687	292	148	0.980371193	0.115853685	FALSE
GB15688	374	227	0.720345973	-0.144171536	FALSE
GB15689	1013	556	0.865477386	0.000959878	FALSE
GB15690	449	295	0.60600049	-0.258517018	FALSE
GB15691	1812	1299	0.480181525	-0.384335984	FALSE
GB15692	14415	10055	0.519657755	-0.344859753	FALSE
GB15693	618	356	0.795729597	-0.068787911	FALSE
GB15694	909	511	0.830957003	-0.033560505	FALSE
GB15695	532	361	0.559427409	-0.3050901	FALSE
GB15696	11699	4089	1.516565249	0.652047741	TRUE
GB15697	345	141	1.290901199	0.426383691	FALSE
GB15698	122	62	0.976541027	0.112023519	FALSE
GB15699	668	397	0.750709095	-0.113808413	FALSE
GB15700	5026	4413	0.187650924	-0.676866584	TRUE
GB15701	NA	NA	NA	NA	FALSE
GB15702	58	70	-0.271302022	-1.13581953	TRUE
GB15703	381	172	1.147382433	0.282864924	FALSE
GB15704	408	273	0.579668201	-0.284849307	FALSE
GB15705	239	83	1.525827377	0.661309868	TRUE
GB15706	1155	638	0.856264523	-0.008252986	FALSE
GB15707	1	1	0	-0.864517508	TRUE
GB15708	1597	959	0.735761592	-0.128755916	FALSE
GB15709	1073	568	0.917687241	0.053169733	FALSE
GB15710	530	253	1.066854975	0.202337466	FALSE
GB15711	2519	1451	0.795803603	-0.068713906	FALSE
GB15712	55	31	0.827163403	-0.037354105	FALSE
GB15713	189	63	1.584962501	0.720444992	TRUE
GB15714	1429	780	0.873459887	0.008942379	FALSE
GB15715	477	245	0.961207517	0.096690009	FALSE
GB15716	1043	689	0.59816327	-0.266354239	FALSE

GB15717	461	277	0.734880774	-0.129636734	FALSE
GB15718	36814	18985	0.955394514	0.090877006	FALSE
GB15719	1491	826	0.852066571	-0.012450937	FALSE
GB15720	485	257	0.916216388	0.05169888	FALSE
GB15721	937	374	1.325010778	0.460493269	FALSE
GB15722	NA	NA	NA	NA	FALSE
GB15723	678	395	0.77943262	-0.085084888	FALSE
GB15724	441	239	0.883768038	0.019250529	FALSE
GB15725	8	0	NA	NA	FALSE
GB15726	1550	1228	0.335957655	-0.528559854	TRUE
GB15727	2164	1131	0.93610157	0.071584062	FALSE
GB15728	1486	695	1.096349233	0.231831725	FALSE
GB15729	559	344	0.700439718	-0.16407779	FALSE
GB15730	349	172	1.020818472	0.156300963	FALSE
GB15731	451	259	0.800175336	-0.064342173	FALSE
GB15732	893	482	0.889627029	0.025109521	FALSE
GB15733	1	2	-1	-1.864517508	TRUE
GB15734	521	222	1.230723696	0.366206188	FALSE
GB15735	1634	876	0.899405209	0.0348877	FALSE
GB15736	304	135	1.171111916	0.306594408	FALSE
GB15737	NA	NA	NA	NA	FALSE
GB15738	2148	1328	0.693738847	-0.170778662	FALSE
GB15739	1359	819	0.730610099	-0.133907409	FALSE
GB15740	32	3	3.415037499	2.550519991	TRUE
GB15741	239	101	1.242655325	0.378137817	FALSE
GB15742	37	58	-0.648527629	-1.513045138	TRUE
GB15743	777	471	0.722187539	-0.14232997	FALSE
GB15744	1767	938	0.913642212	0.049124704	FALSE
GB15745	13556	8454	0.681225524	-0.183291984	FALSE
GB15746	1857	1199	0.631142157	-0.233375352	FALSE
GB15747	622	382	0.703341942	-0.161175566	FALSE
GB15748	1237	679	0.865362021	0.000844512	FALSE
GB15749	469	255	0.879090676	0.014573167	FALSE
GB15750	2576	1088	1.243454037	0.378936529	FALSE
GB15751	76	40	0.925999419	0.06148191	FALSE
GB15752	220	148	0.571906348	-0.29261116	FALSE
GB15753	NA	NA	NA	NA	FALSE
GB15754	14	6	1.222392421	0.357874913	FALSE
GB15755	14231	9832	0.533480222	-0.331037287	FALSE
GB15756	NA	NA	NA	NA	FALSE
GB15757	1155	617	0.904550457	0.040032949	FALSE
GB15758	114	93	0.293731203	-0.570786305	TRUE
GB15759	4987	1927	1.371815631	0.507298123	TRUE
GB15760	111	57	0.961525852	0.097008344	FALSE

GB15761	NA	NA	NA	NA	FALSE
GB15762	2344	1823	0.362658008	-0.5018595	TRUE
GB15763	162	88	0.880418384	0.015900876	FALSE
GB15764	1380	585	1.238159737	0.373642229	FALSE
GB15765	15	0	NA	NA	FALSE
GB15766	NA	NA	NA	NA	FALSE
GB15767	1094	534	1.034701091	0.170183583	FALSE
GB15768	4129	1941	1.0889923	0.224474792	FALSE
GB15769	1028	522	0.977718553	0.113201044	FALSE
GB15770	525	350	0.584962501	-0.279555008	FALSE
GB15771	1101	625	0.816886374	-0.047631134	FALSE
GB15772	1397	730	0.936363652	0.071846143	FALSE
GB15773	525	262	1.002750611	0.138233103	FALSE
GB15774	737	401	0.878062383	0.013544874	FALSE
GB15775	1934	1107	0.804932573	-0.059584936	FALSE
GB15776	238	179	0.411001986	-0.453515522	FALSE
GB15777	192	83	1.209923069	0.345405561	FALSE
GB15778	707	341	1.051938476	0.187420967	FALSE
GB15779	6278	5439	0.206963603	-0.657553905	TRUE
GB15780	2999	1348	1.153661026	0.289143517	FALSE
GB15781	2247	1489	0.593656371	-0.270861138	FALSE
GB15782	992	475	1.062412607	0.197895099	FALSE
GB15783	NA	NA	NA	NA	FALSE
GB15784	862	341	1.33791613	0.473398622	FALSE
GB15785	1640	1103	0.572263024	-0.292254484	FALSE
GB15786	60	18	1.736965594	0.872448086	TRUE
GB15787	17488	8818	0.987841924	0.123324416	FALSE
GB15788	0	1	NA	NA	FALSE
GB15789	100	77	0.377069649	-0.487447859	FALSE
GB15790	167	155	0.107579887	-0.756937621	TRUE
GB15791	29	22	0.398549376	-0.465968132	FALSE
GB15792	16976	9208	0.882536823	0.018019314	FALSE
GB15793	9422	2701	1.802539692	0.938022184	TRUE
GB15794	417	191	1.126474745	0.261957237	FALSE
GB15795	330	205	0.686842115	-0.177675394	FALSE
GB15796	3730	2476	0.591164316	-0.273353192	FALSE
GB15797	2	0	NA	NA	FALSE
GB15798	126	128	-0.022720077	-0.887237585	TRUE
GB15799	531	369	0.525091045	-0.339426464	FALSE
GB15800	NA	NA	NA	NA	FALSE
GB15801	24	19	0.337034987	-0.527482521	TRUE
GB15802	293	134	1.128667664	0.264150156	FALSE
GB15803	1401	947	0.565020625	-0.299496883	FALSE
GB15804	472	219	1.10785599	0.243338481	FALSE

GB15805	1935	1321	0.5507031	-0.313814409	FALSE
GB15806	1509	957	0.657001976	-0.207515532	FALSE
GB15807	8449	4926	0.77836397	-0.086153538	FALSE
GB15808	NA	NA	NA	NA	FALSE
GB15809	1140	627	0.862496476	-0.002021032	FALSE
GB15810	NA	NA	NA	NA	FALSE
GB15811	2	1	1	0.135482492	FALSE
GB15812	6	2	1.584962501	0.720444992	TRUE
GB15813	1	0	NA	NA	FALSE
GB15814	269	170	0.662071426	-0.202446082	FALSE
GB15815	700	406	0.785875195	-0.078642314	FALSE
GB15816	2651	1682	0.656358965	-0.208158544	FALSE
GB15817	2	1	1	0.135482492	FALSE
GB15818	5647	3765	0.584834766	-0.279682742	FALSE
GB15819	781	491	0.669599524	-0.194917985	FALSE
GB15820	50	19	1.395928676	0.531411168	TRUE
GB15821	NA	NA	NA	NA	FALSE
GB15822	1853	882	1.07101232	0.206494812	FALSE
GB15823	NA	NA	NA	NA	FALSE
GB15824	310	216	0.521236903	-0.343280605	FALSE
GB15825	150	65	1.206450877	0.341933369	FALSE
GB15826	976	648	0.590887335	-0.273630174	FALSE
GB15827	11563	6150	0.910857436	0.046339927	FALSE
GB15828	616	379	0.700732503	-0.163785006	FALSE
GB15829	72	35	1.040641984	0.176124476	FALSE
GB15830	1	0	NA	NA	FALSE
GB15831	13690	7750	0.820854231	-0.043663277	FALSE
GB15832	1101	511	1.107419273	0.242901764	FALSE
GB15833	2144	1183	0.857854832	-0.006662676	FALSE
GB15834	3290	2065	0.671945802	-0.192571706	FALSE
GB15835	833	459	0.859822342	-0.004695166	FALSE
GB15836	18	15	0.263034406	-0.601483102	TRUE
GB15837	1624	833	0.963163232	0.098645723	FALSE
GB15838	821	429	0.936404574	0.071887066	FALSE
GB15839	159	89	0.837149524	-0.027367984	FALSE
GB15840	NA	NA	NA	NA	FALSE
GB15841	314	172	0.868355994	0.003838486	FALSE
GB15842	NA	NA	NA	NA	FALSE
GB15843	143	92	0.636309381	-0.228208128	FALSE
GB15844	NA	NA	NA	NA	FALSE
GB15845	NA	NA	NA	NA	FALSE
GB15846	656	341	0.943924076	0.079406567	FALSE
GB15847	463	195	1.24753807	0.383020561	FALSE
GB15848	61	17	1.843274496	0.978756988	TRUE

GB15849	3703	2444	0.599450264	-0.265067244	FALSE
GB15850	1	0	NA	NA	FALSE
GB15851	783	382	1.035439669	0.170922161	FALSE
GB15852	NA	NA	NA	NA	FALSE
GB15853	2505	1341	0.901501366	0.036983858	FALSE
GB15854	9933	5651	0.813723322	-0.050794186	FALSE
GB15855	5566	3546	0.650448372	-0.214069136	FALSE
GB15856	710	423	0.747161361	-0.117356147	FALSE
GB15857	886	420	1.076917371	0.212399863	FALSE
GB15858	2357	1149	1.036572961	0.172055452	FALSE
GB15859	711	484	0.554842512	-0.309674996	FALSE
GB15860	2376	1021	1.21855197	0.354034462	FALSE
GB15861	91	67	0.44170545	-0.422812059	FALSE
GB15862	267	131	1.02727293	0.162755422	FALSE
GB15863	949	542	0.808115236	-0.056402273	FALSE
GB15864	516	294	0.811554911	-0.052962598	FALSE
GB15865	NA	NA	NA	NA	FALSE
GB15866	6	3	1	0.135482492	FALSE
GB15867	557	244	1.19079618	0.326278671	FALSE
GB15868	NA	NA	NA	NA	FALSE
GB15869	357	140	1.350497247	0.485979739	FALSE
GB15870	112	86	0.381090167	-0.483427341	FALSE
GB15871	721	390	0.886525135	0.022007627	FALSE
GB15872	602	294	1.033947332	0.169429824	FALSE
GB15873	NA	NA	NA	NA	FALSE
GB15874	2275	1603	0.50509212	-0.359425389	FALSE
GB15875	362	201	0.848794196	-0.015723312	FALSE
GB15876	47	20	1.232660757	0.368143248	FALSE
GB15877	769	583	0.399487715	-0.465029794	FALSE
GB15878	4	4	0	-0.864517508	TRUE
GB15879	104	64	0.700439718	-0.16407779	FALSE
GB15880	1225	674	0.861961253	-0.002556256	FALSE
GB15881	1044	779	0.422426479	-0.44209103	FALSE
GB15882	195	134	0.541241123	-0.323276385	FALSE
GB15883	353	200	0.819668183	-0.044849325	FALSE
GB15884	159	70	1.183599938	0.31908243	FALSE
GB15885	679	529	0.360143852	-0.504373656	TRUE
GB15886	4453	2174	1.034425671	0.169908163	FALSE
GB15887	334	162	1.04385429	0.179336781	FALSE
GB15888	1351	670	1.011794674	0.147277166	FALSE
GB15889	838	451	0.89382281	0.029305302	FALSE
GB15890	209	149	0.488190612	-0.376326897	FALSE
GB15891	NA	NA	NA	NA	FALSE
GB15892	135	61	1.146078259	0.281560751	FALSE

GB15893	464	238	0.963163232	0.098645723	FALSE
GB15894	0	2	NA	NA	FALSE
GB15895	908	465	0.965461581	0.100944073	FALSE
GB15896	3333	1710	0.962824992	0.098307484	FALSE
GB15897	7	2	1.807354922	0.942837414	TRUE
GB15898	6	2	1.584962501	0.720444992	TRUE
GB15899	2	4	-1	-1.864517508	TRUE
GB15900	240	121	0.988027358	0.12350985	FALSE
GB15901	331	202	0.712475924	-0.152041584	FALSE
GB15902	663	368	0.849303104	-0.015214404	FALSE
GB15903	370	215	0.783188611	-0.081328897	FALSE
GB15904	53	15	1.821029859	0.956512351	TRUE
GB15905	1957	818	1.258471008	0.393953499	FALSE
GB15906	35	14	1.321928095	0.457410587	FALSE
GB15907	436	220	0.986824611	0.122307103	FALSE
GB15908	3270	1589	1.041171511	0.176654003	FALSE
GB15909	846	382	1.147085025	0.282567517	FALSE
GB15910	20	25	-0.321928095	-1.186445603	TRUE
GB15911	19	9	1.078002512	0.213485004	FALSE
GB15912	9	12	-0.415037499	-1.279555008	TRUE
GB15913	311	179	0.796954993	-0.067562515	FALSE
GB15914	810	487	0.734000136	-0.130517373	FALSE
GB15915	329	184	0.838381818	-0.026135691	FALSE
GB15916	1119	676	0.727114885	-0.137402624	FALSE
GB15917	208	62	1.746243408	0.881725899	TRUE
GB15918	170	102	0.736965594	-0.127551914	FALSE
GB15919	1118	558	1.002583161	0.138065653	FALSE
GB15920	106	49	1.11321061	0.248693102	FALSE
GB15921	6452	3204	1.009872291	0.145354782	FALSE
GB15922	711	407	0.804820765	-0.059696743	FALSE
GB15923	NA	NA	NA	NA	FALSE
GB15924	317	155	1.032214625	0.167697117	FALSE
GB15925	1758	964	0.866830019	0.002312511	FALSE
GB15926	1	0	NA	NA	FALSE
GB15927	35	21	0.736965594	-0.127551914	FALSE
GB15928	62	25	1.310340121	0.445822612	FALSE
GB15929	292	210	0.475579041	-0.388938467	FALSE
GB15930	215	127	0.759508163	-0.105009346	FALSE
GB15931	239	131	0.867443806	0.002926298	FALSE
GB15932	726	419	0.793019304	-0.071498204	FALSE
GB15933	9387	5710	0.717173414	-0.147344095	FALSE
GB15934	152	78	0.962525295	0.098007786	FALSE
GB15935	1	0	NA	NA	FALSE
GB15936	284	172	0.723482365	-0.141035144	FALSE

GB15937	259	288	-0.153116714	-1.017634222	TRUE
GB15938	NA	NA	NA	NA	FALSE
GB15939	1	5	-2.321928095	-3.186445603	TRUE
GB15940	855	573	0.577389281	-0.287128227	FALSE
GB15941	327	179	0.869331048	0.00481354	FALSE
GB15942	406	260	0.642968104	-0.221549404	FALSE
GB15943	118	41	1.525091045	0.660573536	TRUE
GB15944	NA	NA	NA	NA	FALSE
GB15945	47	23	1.031026896	0.166509387	FALSE
GB15946	2592	1456	0.832055363	-0.032462146	FALSE
GB15947	1243	545	1.189498161	0.324980653	FALSE
GB15948	1099	659	0.737841016	-0.126676492	FALSE
GB15949	383	240	0.674309986	-0.190207522	FALSE
GB15950	198	120	0.722466024	-0.142051484	FALSE
GB15951	NA	NA	NA	NA	FALSE
GB15952	725	494	0.553469953	-0.311047555	FALSE
GB15953	445	217	1.036110293	0.171592785	FALSE
GB15954	521	284	0.875392443	0.010874934	FALSE
GB15955	116	64	0.857980995	-0.006536513	FALSE
GB15956	19	12	0.662965013	-0.201552496	FALSE
GB15957	594	223	1.413419221	0.548901713	TRUE
GB15958	420	244	0.78350818	-0.081009328	FALSE
GB15959	1547	1170	0.402964667	-0.461552841	FALSE
GB15960	385	312	0.303312417	-0.561205092	TRUE
GB15961	217	135	0.684735635	-0.179781873	FALSE
GB15962	1434	875	0.712690102	-0.151827406	FALSE
GB15963	2834	1333	1.088162978	0.22364547	FALSE
GB15964	2	3	-0.584962501	-1.449480009	TRUE
GB15965	317	136	1.220876189	0.356358681	FALSE
GB15966	143	132	0.115477217	-0.749040291	TRUE
GB15967	4	4	0	-0.864517508	TRUE
GB15968	5	10	-1	-1.864517508	TRUE
GB15969	303	236	0.360530934	-0.503986574	TRUE
GB15970	410	111	1.885064233	1.020546725	TRUE
GB15971	39	40	-0.036525876	-0.901043384	TRUE
GB15972	108	80	0.432959407	-0.431558101	FALSE
GB15973	494	425	0.217048201	-0.647469308	TRUE
GB15974	21	16	0.392317423	-0.472200086	FALSE
GB15975	377	232	0.700439718	-0.16407779	FALSE
GB15976	NA	NA	NA	NA	FALSE
GB15977	589	287	1.037216897	0.172699389	FALSE
GB15978	972	462	1.073063462	0.208545954	FALSE
GB15979	5497	3147	0.804667301	-0.059850208	FALSE
GB15980	1174	512	1.197216693	0.332699185	FALSE

GB15981	271	140	0.952866024	0.088348516	FALSE
GB15982	276	200	0.464668267	-0.399849241	FALSE
GB15983	571	305	0.904681503	0.040163995	FALSE
GB15984	571	267	1.096651004	0.232133495	FALSE
GB15985	3	0	NA	NA	FALSE
GB15986	2725	1575	0.790904401	-0.073613107	FALSE
GB15987	70	28	1.321928095	0.457410587	FALSE
GB15988	49	28	0.807354922	-0.057162586	FALSE
GB15989	1362	640	1.089582893	0.225065385	FALSE
GB15990	244691	108802	1.169255962	0.304738454	FALSE
GB15991	741	469	0.65988562	-0.204631889	FALSE
GB15992	2978	1656	0.846641081	-0.017876427	FALSE
GB15993	3270	2032	0.686390234	-0.178127275	FALSE
GB15994	4294	1967	1.126325234	0.261807725	FALSE
GB15995	110	81	0.441509711	-0.423007798	FALSE
GB15996	8	8	0	-0.864517508	TRUE
GB15997	179	95	0.913960169	0.049442661	FALSE
GB15998	739	487	0.601652592	-0.262864916	FALSE
GB15999	224	122	0.876617584	0.012100076	FALSE
GB16000	9	1	3.169925001	2.305407493	TRUE
GB16001	1080	672	0.684498174	-0.180019334	FALSE
GB16002	14	6	1.222392421	0.357874913	FALSE
GB16003	1009	562	0.844284139	-0.020233369	FALSE
GB16004	1	0	NA	NA	FALSE
GB16005	40	19	1.074000581	0.209483073	FALSE
GB16006	46	23	1	0.135482492	FALSE
GB16007	50	45	0.152003093	-0.712514415	TRUE
GB16008	1524	981	0.635537861	-0.228979647	FALSE
GB16009	8	3	1.415037499	0.550519991	TRUE
GB16010	3832	2170	0.820402518	-0.04411499	FALSE
GB16011	390	210	0.893084796	0.028567288	FALSE
GB16012	1887	775	1.283826207	0.419308699	FALSE
GB16013	0	2	NA	NA	FALSE
GB16014	260	119	1.12755005	0.263032541	FALSE
GB16015	97	2	5.599912842	4.735395334	TRUE
GB16016	15420	8339	0.886856472	0.022338964	FALSE
GB16017	285	188	0.600229257	-0.264288251	FALSE
GB16018	2873	1718	0.741827956	-0.122689552	FALSE
GB16019	980	525	0.900464326	0.035946818	FALSE
GB16020	NA	NA	NA	NA	FALSE
GB16021	27054	13666	0.985250884	0.120733376	FALSE
GB16022	49	17	1.527247003	0.662729495	TRUE
GB16023	28	24	0.222392421	-0.642125087	TRUE
GB16024	1641	964	0.767470187	-0.097047321	FALSE

GB16025	903	387	1.222392421	0.357874913	FALSE
GB16026	78	33	1.2410081	0.376490591	FALSE
GB16027	457	352	0.376618736	-0.487898772	FALSE
GB16028	5702	3459	0.721113026	-0.143404483	FALSE
GB16029	432	224	0.94753258	0.083015072	FALSE
GB16030	69	50	0.464668267	-0.399849241	FALSE
GB16031	52	21	1.308122295	0.443604787	FALSE
GB16032	123	49	1.327804661	0.463287153	FALSE
GB16033	422	195	1.113768875	0.249251367	FALSE
GB16034	551	300	0.877089818	0.01257231	FALSE
GB16035	1	0	NA	NA	FALSE
GB16036	178	72	1.30580843	0.441290921	FALSE
GB16037	13	4	1.700439718	0.83592221	TRUE
GB16038	331	140	1.24140439	0.376886882	FALSE
GB16039	502	274	0.873511471	0.008993963	FALSE
GB16040	0	1	NA	NA	FALSE
GB16041	13	10	0.378511623	-0.486005885	FALSE
GB16042	2062	1166	0.822476544	-0.042040964	FALSE
GB16043	266	140	0.925999419	0.06148191	FALSE
GB16044	408	208	0.971985624	0.107468116	FALSE
GB16045	89	25	1.831877241	0.967359733	TRUE
GB16046	605	301	1.007171655	0.142654147	FALSE
GB16047	3873	2344	0.724478932	-0.140038577	FALSE
GB16048	614	387	0.665905089	-0.198612419	FALSE
GB16049	5887	4916	0.260047712	-0.604469796	TRUE
GB16050	1395	926	0.591181023	-0.273336485	FALSE
GB16051	240	242	-0.011972642	-0.87649015	TRUE
GB16052	65	80	-0.299560282	-1.16407779	TRUE
GB16053	6	2	1.584962501	0.720444992	TRUE
GB16054	1057	554	0.932017495	0.067499987	FALSE
GB16055	148	69	1.100928909	0.236411401	FALSE
GB16056	177	82	1.110053545	0.245536037	FALSE
GB16057	1802	702	1.360056076	0.495538567	FALSE
GB16058	55	25	1.137503524	0.272986015	FALSE
GB16059	3608	1889	0.933576637	0.069059129	FALSE
GB16060	41	25	0.713695815	-0.150821693	FALSE
GB16061	NA	NA	NA	NA	FALSE
GB16062	NA	NA	NA	NA	FALSE
GB16063	782	594	0.396705677	-0.467811832	FALSE
GB16064	3744	1888	0.98772167	0.123204162	FALSE
GB16065	9	1	3.169925001	2.305407493	TRUE
GB16066	3952	2188	0.852970209	-0.0115473	FALSE
GB16067	757	422	0.843050301	-0.021467207	FALSE
GB16068	681	430	0.663318138	-0.20119937	FALSE

GB16069	712	484	0.556870194	-0.307647315	FALSE
GB16070	100	65	0.621488377	-0.243029132	FALSE
GB16071	1001	456	1.134336245	0.269818736	FALSE
GB16072	9366	5539	0.7578075	-0.106710009	FALSE
GB16073	3158	1773	0.832818635	-0.031698873	FALSE
GB16074	351	146	1.265502661	0.400985153	FALSE
GB16075	2903	2863	0.020016902	-0.844500607	TRUE
GB16076	15	3	2.321928095	1.457410587	TRUE
GB16077	664	385	0.786324796	-0.078192713	FALSE
GB16078	336	213	0.657607803	-0.206909706	FALSE
GB16079	72	28	1.362570079	0.498052571	FALSE
GB16080	6697	2834	1.24067521	0.376157701	FALSE
GB16081	572	241	1.246982001	0.382464492	FALSE
GB16082	3441	1848	0.896863135	0.032345627	FALSE
GB16083	77	33	1.222392421	0.357874913	FALSE
GB16084	225	153	0.556393349	-0.30812416	FALSE
GB16085	828	485	0.77164602	-0.092871488	FALSE
GB16086	758	551	0.46014553	-0.404371979	FALSE
GB16087	0	3	NA	NA	FALSE
GB16088	206	94	1.131911676	0.267394167	FALSE
GB16089	60	48	0.321928095	-0.542589413	TRUE
GB16090	403	210	0.940390511	0.075873003	FALSE
GB16091	259	161	0.68589141	-0.178626099	FALSE
GB16092	45	15	1.584962501	0.720444992	TRUE
GB16093	522	303	0.784732013	-0.079785495	FALSE
GB16094	1837	1115	0.720307916	-0.144209592	FALSE
GB16095	156	111	0.490986353	-0.373531156	FALSE
GB16096	315	199	0.662583398	-0.20193411	FALSE
GB16097	473	294	0.686024029	-0.17849348	FALSE
GB16098	NA	NA	NA	NA	FALSE
GB16099	354	209	0.760246418	-0.10427109	FALSE
GB16100	164	100	0.713695815	-0.150821693	FALSE
GB16101	1002	494	1.020299562	0.155782053	FALSE
GB16102	53	22	1.268488836	0.403971328	FALSE
GB16103	445	261	0.769755529	-0.094761979	FALSE
GB16104	1354	829	0.707783732	-0.156733776	FALSE
GB16105	536	271	0.983940149	0.119422641	FALSE
GB16106	3	3	0	-0.864517508	TRUE
GB16107	69	31	1.154328146	0.289810638	FALSE
GB16108	231	131	0.81832604	-0.046191468	FALSE
GB16109	1005	583	0.785627713	-0.078889795	FALSE
GB16110	2421	1692	0.516873511	-0.347643997	FALSE
GB16111	22190	14166	0.64747722	-0.217040288	FALSE
GB16112	1806	857	1.075430783	0.210913275	FALSE

GB16113	205	146	0.489655541	-0.374861968	FALSE
GB16114	282	240	0.232660757	-0.631856752	TRUE
GB16115	56	28	1	0.135482492	FALSE
GB16116	703	331	1.086693472	0.222175964	FALSE
GB16117	1364	709	0.943986112	0.079468603	FALSE
GB16118	366	246	0.573185333	-0.291332175	FALSE
GB16119	390	278	0.488389241	-0.376128267	FALSE
GB16120	1115	945	0.238657476	-0.625860033	TRUE
GB16121	1	0	NA	NA	FALSE
GB16122	3	0	NA	NA	FALSE
GB16123	1095	409	1.420758122	0.556240613	TRUE
GB16124	325	246	0.401781403	-0.462736106	FALSE
GB16125	4801	2542	0.917370906	0.052853397	FALSE
GB16126	NA	NA	NA	NA	FALSE
GB16127	NA	NA	NA	NA	FALSE
GB16128	13	4	1.700439718	0.83592221	TRUE
GB16129	3990	2481	0.685467011	-0.179050497	FALSE
GB16130	180	119	0.597035333	-0.267482175	FALSE
GB16131	17	3	2.502500341	1.637982832	TRUE
GB16132	6363	4533	0.48924096	-0.375276548	FALSE
GB16133	908	516	0.815321232	-0.049196276	FALSE
GB16134	2315	1250	0.889084099	0.02456659	FALSE
GB16135	4880	2230	1.129837438	0.265319929	FALSE
GB16136	19	4	2.247927513	1.383410005	TRUE
GB16137	1225	858	0.513732196	-0.350785312	FALSE
GB16138	1329	872	0.607941064	-0.256576444	FALSE
GB16139	395	211	0.904609654	0.040092146	FALSE
GB16140	1877	997	0.91276324	0.048245732	FALSE
GB16141	3977	2040	0.96311141	0.098593902	FALSE
GB16142	1365	721	0.920829787	0.056312278	FALSE
GB16143	1075	712	0.594387514	-0.270129995	FALSE
GB16144	1394	762	0.871367658	0.00685015	FALSE
GB16145	93	53	0.811238357	-0.053279152	FALSE
GB16146	1146	632	0.858610581	-0.005906928	FALSE
GB16147	1951	1512	0.367755638	-0.49676187	FALSE
GB16148	1094	590	0.890825879	0.02630837	FALSE
GB16149	7951	5776	0.461065578	-0.40345193	FALSE
GB16150	208	124	0.746243408	-0.118274101	FALSE
GB16151	19	3	2.662965013	1.798447504	TRUE
GB16152	4306	2201	0.968189174	0.103671666	FALSE
GB16153	3353	1798	0.899059462	0.034541954	FALSE
GB16154	479	296	0.69442848	-0.170089028	FALSE
GB16155	289	166	0.799886251	-0.064631257	FALSE
GB16156	328	154	1.090765464	0.226247956	FALSE

GB16157	826	452	0.869819009	0.005301501	FALSE
GB16158	978	476	1.038872892	0.174355383	FALSE
GB16159	2174	1030	1.077707603	0.213190095	FALSE
GB16160	767	481	0.673189684	-0.191327825	FALSE
GB16161	1	0	NA	NA	FALSE
GB16162	5286	2882	0.875106089	0.010588581	FALSE
GB16163	1044	569	0.875621154	0.011103646	FALSE
GB16164	5	1	2.321928095	1.457410587	TRUE
GB16165	380	224	0.762500686	-0.102016822	FALSE
GB16166	22	12	0.874469118	0.00995161	FALSE
GB16167	827	531	0.639175468	-0.22534204	FALSE
GB16168	6265	3833	0.708840511	-0.155676997	FALSE
GB16169	8879	5206	0.77022189	-0.094295618	FALSE
GB16170	75	20	1.906890596	1.042373087	TRUE
GB16171	283	128	1.144658243	0.280140735	FALSE
GB16172	435	205	1.085391491	0.220873983	FALSE
GB16173	2	0	NA	NA	FALSE
GB16174	1315	707	0.895280679	0.030763171	FALSE
GB16175	2942	2447	0.265783145	-0.598734363	TRUE
GB16176	2681	1424	0.912822073	0.048304565	FALSE
GB16177	NA	NA	NA	NA	FALSE
GB16178	393	109	1.850201177	0.985683669	TRUE
GB16179	971	514	0.917702936	0.053185428	FALSE
GB16180	422	252	0.743819265	-0.120698243	FALSE
GB16181	1722	744	1.210710616	0.346193108	FALSE
GB16182	17849	11560	0.626701851	-0.237815657	FALSE
GB16183	1597	834	0.937245024	0.072727516	FALSE
GB16184	13136	9279	0.501484793	-0.363032715	FALSE
GB16185	307	132	1.217700726	0.353183218	FALSE
GB16186	4702	2965	0.665242434	-0.199275074	FALSE
GB16187	210	110	0.932885804	0.068368296	FALSE
GB16188	175	57	1.618321098	0.753803589	TRUE
GB16189	8534	4659	0.87320178	0.008684272	FALSE
GB16190	110	81	0.441509711	-0.423007798	FALSE
GB16191	14	3	2.222392421	1.357874913	TRUE
GB16192	134	80	0.744161096	-0.120356413	FALSE
GB16193	214	156	0.456064768	-0.408452741	FALSE
GB16194	1553	746	1.057810294	0.193292786	FALSE
GB16195	33	17	0.956931278	0.09241377	FALSE
GB16196	4332	1399	1.63063728	0.766119772	TRUE
GB16197	64	41	0.642447995	-0.222069513	FALSE
GB16198	485	244	0.9911036	0.126586091	FALSE
GB16199	99	34	1.541893779	0.677376271	TRUE
GB16200	1088	838	0.376656408	-0.487861101	FALSE

GB16201	12292	6341	0.95493739	0.090419882	FALSE
GB16202	615	864	-0.490444902	-1.35496241	TRUE
GB16203	207	96	1.108524457	0.244006948	FALSE
GB16204	32	11	1.540568381	0.676050873	TRUE
GB16205	1469	904	0.700439718	-0.16407779	FALSE
GB16206	1	0	NA	NA	FALSE
GB16207	458	282	0.699652436	-0.164865073	FALSE
GB16208	38	10	1.925999419	1.06148191	TRUE
GB16209	450	298	0.594612671	-0.269904838	FALSE
GB16210	1286	762	0.75502774	-0.109489768	FALSE
GB16211	NA	NA	NA	NA	FALSE
GB16212	344	179	0.942448977	0.077931469	FALSE
GB16213	211	152	0.473171675	-0.391345833	FALSE
GB16214	449	193	1.218114597	0.353597089	FALSE
GB16215	3030	1250	1.277389699	0.41287219	FALSE
GB16216	1663	835	0.993940066	0.129422558	FALSE
GB16217	402	183	1.135351853	0.270834345	FALSE
GB16218	632	300	1.074962058	0.210444549	FALSE
GB16219	1090	864	0.335224918	-0.529292591	TRUE
GB16220	151	98	0.623694895	-0.240822613	FALSE
GB16221	3	1	1.584962501	0.720444992	TRUE
GB16222	208	126	0.723159795	-0.141357714	FALSE
GB16223	1703	778	1.130236375	0.265718866	FALSE
GB16224	357	193	0.887323227	0.022805718	FALSE
GB16225	2937	1839	0.675421786	-0.189095722	FALSE
GB16226	257	145	0.825715459	-0.038802049	FALSE
GB16227	6	3	1	0.135482492	FALSE
GB16228	1	0	NA	NA	FALSE
GB16229	1063	594	0.839606761	-0.024910748	FALSE
GB16230	1320	740	0.834940754	-0.029576755	FALSE
GB16231	851	365	1.221262668	0.35674516	FALSE
GB16232	NA	NA	NA	NA	FALSE
GB16233	886	623	0.508074536	-0.356442973	FALSE
GB16234	1283	626	1.035286608	0.1707691	FALSE
GB16235	2076	1025	1.018182534	0.153665026	FALSE
GB16236	14310	5538	1.369586713	0.505069205	TRUE
GB16237	882	480	0.87774425	0.013226742	FALSE
GB16238	1543	925	0.738212791	-0.126304717	FALSE
GB16239	186	74	1.329705445	0.465187937	FALSE
GB16240	20	13	0.621488377	-0.243029132	FALSE
GB16241	2423	1179	1.039230686	0.174713178	FALSE
GB16242	203	171	0.247483402	-0.617034106	TRUE
GB16243	69	25	1.464668267	0.600150759	TRUE
GB16244	8	7	0.192645078	-0.67187243	TRUE

GB16245	24852	12576	0.982688837	0.118171328	FALSE
GB16246	0	1	NA	NA	FALSE
GB16247	69	27	1.353636955	0.489119446	FALSE
GB16248	251	150	0.742724863	-0.121792645	FALSE
GB16249	8	1	3	2.135482492	TRUE
GB16250	889	480	0.889149013	0.024631505	FALSE
GB16251	398	210	0.922379103	0.057861595	FALSE
GB16252	1533	812	0.916806064	0.052288556	FALSE
GB16253	2698	1600	0.753818443	-0.110699065	FALSE
GB16254	135	66	1.032421478	0.167903969	FALSE
GB16255	495	258	0.94005746	0.075539951	FALSE
GB16256	4113	2096	0.972552355	0.108034847	FALSE
GB16257	2977	1612	0.885007478	0.020489969	FALSE
GB16258	9	3	1.584962501	0.720444992	TRUE
GB16259	3	0	NA	NA	FALSE
GB16260	604	393	0.620019237	-0.244498271	FALSE
GB16261	4528	2896	0.644812356	-0.219705153	FALSE
GB16262	64	32	1	0.135482492	FALSE
GB16263	669	265	1.336013851	0.471496343	FALSE
GB16264	70	8	3.129283017	2.264765509	TRUE
GB16265	1740	1001	0.797645332	-0.066872176	FALSE
GB16266	4	4	0	-0.864517508	TRUE
GB16267	789	457	0.787831135	-0.076686373	FALSE
GB16268	611	409	0.579071537	-0.285445971	FALSE
GB16269	50	35	0.514573173	-0.349944335	FALSE
GB16270	281	178	0.658692889	-0.205824619	FALSE
GB16271	160	97	0.722015253	-0.142502256	FALSE
GB16272	1147	539	1.089508213	0.224990705	FALSE
GB16273	97	68	0.512450001	-0.352067507	FALSE
GB16274	426	219	0.959922561	0.095405052	FALSE
GB16275	1225	579	1.081146496	0.216628988	FALSE
GB16276	2118	1363	0.635917027	-0.228600481	FALSE
GB16277	340	228	0.576500922	-0.288016586	FALSE
GB16278	NA	NA	NA	NA	FALSE
GB16279	25	18	0.473931188	-0.39058632	FALSE
GB16280	455	277	0.715980569	-0.148536939	FALSE
GB16281	2422	1471	0.719401618	-0.14511589	FALSE
GB16282	1749	1274	0.457165012	-0.407352497	FALSE
GB16283	30	15	1	0.135482492	FALSE
GB16284	1299	813	0.676074173	-0.188443335	FALSE
GB16285	267	183	0.544996093	-0.319521415	FALSE
GB16286	655	474	0.466607848	-0.397909661	FALSE
GB16287	1461	499	1.549844457	0.685326949	TRUE
GB16288	411	210	0.968749066	0.104231558	FALSE

GB16289	653	320	1.029011087	0.164493578	FALSE
GB16290	209	105	0.993113614	0.128596106	FALSE
GB16291	2178	1069	1.026742101	0.162224593	FALSE
GB16292	1062	434	1.291016818	0.42649931	FALSE
GB16293	2	2	0	-0.864517508	TRUE
GB16294	2024	1023	0.984403145	0.119885637	FALSE
GB16295	327	200	0.709290636	-0.155226873	FALSE
GB16296	709	427	0.731549558	-0.132967951	FALSE
GB16297	47	37	0.345135486	-0.519382022	TRUE
GB16298	700	219	1.676424052	0.811906544	TRUE
GB16299	153	75	1.028569152	0.164051644	FALSE
GB16300	63	29	1.119298928	0.25478142	FALSE
GB16301	1	0	NA	NA	FALSE
GB16302	1603	926	0.791690327	-0.072827181	FALSE
GB16303	571	421	0.439670512	-0.424846996	FALSE
GB16304	845	385	1.134092896	0.269575387	FALSE
GB16305	1375	881	0.642217694	-0.222299814	FALSE
GB16306	323	241	0.422501018	-0.44201649	FALSE
GB16307	323	189	0.77314793	-0.091369578	FALSE
GB16308	222	97	1.194503024	0.329985516	FALSE
GB16309	463	222	1.060452517	0.195935009	FALSE
GB16310	2943	1532	0.941871245	0.077353737	FALSE
GB16311	35	20	0.807354922	-0.057162586	FALSE
GB16312	1862	1134	0.715432433	-0.149085076	FALSE
GB16313	3007	1462	1.040381557	0.175864048	FALSE
GB16314	58	25	1.214124805	0.349607297	FALSE
GB16315	640	340	0.912537159	0.04801965	FALSE
GB16316	2054	1314	0.644470906	-0.220046602	FALSE
GB16317	NA	NA	NA	NA	FALSE
GB16318	295	188	0.649982293	-0.214535216	FALSE
GB16319	1576	706	1.158527446	0.294009938	FALSE
GB16320	3387	2022	0.74422499	-0.120292519	FALSE
GB16321	3	3	0	-0.864517508	TRUE
GB16322	133	77	0.788495895	-0.076021614	FALSE
GB16323	28	8	1.807354922	0.942837414	TRUE
GB16324	1	1	0	-0.864517508	TRUE
GB16325	2	0	NA	NA	FALSE
GB16326	232	90	1.366127899	0.50161039	TRUE
GB16327	1587	1010	0.651946835	-0.212570673	FALSE
GB16328	94	84	0.162271429	-0.702246079	TRUE
GB16329	248	186	0.415037499	-0.449480009	FALSE
GB16330	3680	1842	0.998432705	0.133915197	FALSE
GB16331	89	50	0.831877241	-0.032640267	FALSE
GB16332	464	271	0.775831954	-0.088685555	FALSE

GB16333	191	95	1.00757322	0.143055711	FALSE
GB16334	178	122	0.544996093	-0.319521415	FALSE
GB16335	8884	6084	0.54618924	-0.318328269	FALSE
GB16336	5070	2734	0.890972504	0.026454996	FALSE
GB16337	20	9	1.152003093	0.287485585	FALSE
GB16338	120	52	1.206450877	0.341933369	FALSE
GB16339	0	3	NA	NA	FALSE
GB16340	112	67	0.741265732	-0.123251777	FALSE
GB16341	606	306	0.985786141	0.121268632	FALSE
GB16342	1	0	NA	NA	FALSE
GB16343	746	259	1.526223533	0.661706024	TRUE
GB16344	33	10	1.722466024	0.857948516	TRUE
GB16345	253	144	0.813068573	-0.051448935	FALSE
GB16346	1189	825	0.527282691	-0.337234818	FALSE
GB16347	0	1	NA	NA	FALSE
GB16348	1837	968	0.924272674	0.059755166	FALSE
GB16349	265	119	1.155030786	0.290513278	FALSE
GB16350	240	179	0.423074818	-0.44144269	FALSE
GB16351	15	7	1.099535674	0.235018165	FALSE
GB16352	2	1	1	0.135482492	FALSE
GB16353	4320	2907	0.571500241	-0.293017267	FALSE
GB16354	6236	3293	0.921218416	0.056700908	FALSE
GB16355	41	27	0.602664502	-0.261853006	FALSE
GB16356	5219	3513	0.571069825	-0.293447683	FALSE
GB16357	NA	NA	NA	NA	FALSE
GB16358	2258	1230	0.876387171	0.011869662	FALSE
GB16359	NA	NA	NA	NA	FALSE
GB16360	1021	558	0.871645839	0.007128331	FALSE
GB16361	163	81	1.008878151	0.144360643	FALSE
GB16362	4051	2730	0.569377134	-0.295140374	FALSE
GB16363	934	386	1.274821702	0.410304194	FALSE
GB16364	611	364	0.74723393	-0.117283579	FALSE
GB16365	31	25	0.310340121	-0.554177388	TRUE
GB16366	653	307	1.088844336	0.224326828	FALSE
GB16367	1	1	0	-0.864517508	TRUE
GB16368	203	111	0.870920051	0.006402543	FALSE
GB16369	51	24	1.087462841	0.222945333	FALSE
GB16370	731	399	0.87348266	0.008965151	FALSE
GB16371	1199	696	0.784672448	-0.079845061	FALSE
GB16372	118	58	1.024662054	0.160144546	FALSE
GB16373	NA	NA	NA	NA	FALSE
GB16374	NA	NA	NA	NA	FALSE
GB16375	2011	1034	0.959676896	0.095159388	FALSE
GB16376	NA	NA	NA	NA	FALSE

GB16377	107	45	1.24961389	0.385096382	FALSE
GB16378	1	0	NA	NA	FALSE
GB16379	94	38	1.306661338	0.44214383	FALSE
GB16380	142	73	0.959922561	0.095405052	FALSE
GB16381	44	9	2.289506617	1.424989109	TRUE
GB16382	1	0	NA	NA	FALSE
GB16383	0	1	NA	NA	FALSE
GB16384	38	15	1.341036918	0.47651941	FALSE
GB16385	952	524	0.861394762	-0.003122747	FALSE
GB16386	56	21	1.415037499	0.550519991	TRUE
GB16387	629	219	1.522129147	0.657611639	TRUE
GB16388	712	445	0.678071905	-0.186445603	FALSE
GB16389	282	192	0.554588852	-0.309928657	FALSE
GB16390	2272	964	1.236857783	0.372340275	FALSE
GB16391	863	385	1.164502114	0.299984605	FALSE
GB16392	3576	2049	0.803426752	-0.061090756	FALSE
GB16393	570	402	0.503766418	-0.36075109	FALSE
GB16394	1786	1062	0.749948314	-0.114569194	FALSE
GB16395	318	180	0.821029859	-0.043487649	FALSE
GB16396	5121	2447	1.065411458	0.200893949	FALSE
GB16397	627	426	0.557612013	-0.306905496	FALSE
GB16398	65	15	2.115477217	1.250959709	TRUE
GB16399	11735	5162	1.184815795	0.320298287	FALSE
GB16400	469	244	0.942706775	0.078189267	FALSE
GB16401	1169	575	1.023641069	0.15912356	FALSE
GB16402	341	171	0.995775414	0.131257906	FALSE
GB16403	967	455	1.087649344	0.223131836	FALSE
GB16404	4	3	0.415037499	-0.449480009	FALSE
GB16405	1444	677	1.092843003	0.228325495	FALSE
GB16406	912	663	0.460024954	-0.404492554	FALSE
GB16407	71	21	1.757429697	0.892912188	TRUE
GB16408	274	144	0.928107082	0.063589573	FALSE
GB16409	935	592	0.659369189	-0.205148319	FALSE
GB16410	738	390	0.920146692	0.055629184	FALSE
GB16411	NA	NA	NA	NA	FALSE
GB16412	24234	14328	0.758195309	-0.106322199	FALSE
GB16413	518	347	0.578016435	-0.286501073	FALSE
GB16414	381	161	1.242730309	0.378212801	FALSE
GB16415	653	313	1.060920335	0.196402826	FALSE
GB16416	10483	4457	1.233906777	0.369389269	FALSE
GB16417	738	335	1.139459721	0.274942212	FALSE
GB16418	839	466	0.848340856	-0.016176653	FALSE
GB16419	NA	NA	NA	NA	FALSE
GB16420	778	458	0.764422557	-0.100094951	FALSE

GB16421	118	54	1.127755547	0.263238039	FALSE
GB16422	880	458	0.942155925	0.077638417	FALSE
GB16423	8211	4614	0.831539931	-0.032977577	FALSE
GB16424	6315	2078	1.60358708	0.739069571	TRUE
GB16425	NA	NA	NA	NA	FALSE
GB16426	NA	NA	NA	NA	FALSE
GB16427	446	205	1.1214198	0.256902292	FALSE
GB16428	11	7	0.652076697	-0.212440812	FALSE
GB16429	12942	9880	0.389477635	-0.475039873	FALSE
GB16430	NA	NA	NA	NA	FALSE
GB16431	46	26	0.823122238	-0.04139527	FALSE
GB16432	737	456	0.692630795	-0.171886713	FALSE
GB16433	316	224	0.496425826	-0.368091682	FALSE
GB16434	4	1	2	1.135482492	TRUE
GB16435	1189	745	0.674436384	-0.190081124	FALSE
GB16436	346	211	0.713529039	-0.150988469	FALSE
GB16437	6185	3149	0.973879838	0.109362329	FALSE
GB16438	481	313	0.619874237	-0.244643271	FALSE
GB16439	1065	534	0.995941783	0.131424275	FALSE
GB16440	964	653	0.561950155	-0.302567354	FALSE
GB16441	470	297	0.662197826	-0.202319683	FALSE
GB16442	462	237	0.963005793	0.098488284	FALSE
GB16443	6562	5123	0.357146702	-0.507370806	TRUE
GB16444	4561	1733	1.396078516	0.531561007	TRUE
GB16445	980	768	0.351675438	-0.51284207	TRUE
GB16446	2032	1180	0.784113543	-0.080403966	FALSE
GB16447	3092	1919	0.688185608	-0.176331901	FALSE
GB16448	8393	3522	1.252791672	0.388274163	FALSE
GB16449	1808	870	1.055307372	0.190789863	FALSE
GB16450	1054	415	1.344691625	0.480174117	FALSE
GB16451	1395	693	1.009337865	0.144820356	FALSE
GB16452	5574	3532	0.658227659	-0.206289849	FALSE
GB16453	355	190	0.901819606	0.037302098	FALSE
GB16454	886	440	1.009803175	0.145285667	FALSE
GB16455	579	272	1.089956697	0.225439188	FALSE
GB16456	182	93	0.968635829	0.104118321	FALSE
GB16457	1193	505	1.24023875	0.375721242	FALSE
GB16458	310	187	0.729229945	-0.135287563	FALSE
GB16459	26	51	-0.971985624	-1.836503132	TRUE
GB16460	1106	534	1.050439739	0.18592223	FALSE
GB16461	393	201	0.967333811	0.102816303	FALSE
GB16462	33	23	0.520832163	-0.343685345	FALSE
GB16463	128	58	1.142019005	0.277501497	FALSE
GB16464	12851	10364	0.310299706	-0.554217802	TRUE

GB16465	214	160	0.419538892	-0.444978617	FALSE
GB16466	645	412	0.646654823	-0.217862685	FALSE
GB16467	1	0	NA	NA	FALSE
GB16468	8631	5330	0.695392189	-0.16912532	FALSE
GB16469	23393	12205	0.938604595	0.074087086	FALSE
GB16470	570	356	0.679084678	-0.18543283	FALSE
GB16471	917	619	0.566982324	-0.297535184	FALSE
GB16472	1895	956	0.987115325	0.122597817	FALSE
GB16473	11	8	0.459431619	-0.40508589	FALSE
GB16474	702	492	0.512812715	-0.351704793	FALSE
GB16475	65	23	1.498805857	0.634288349	TRUE
GB16476	1283	756	0.763063031	-0.101454477	FALSE
GB16477	207	79	1.389706209	0.525188701	TRUE
GB16478	343	139	1.303123693	0.438606185	FALSE
GB16479	746	487	0.615253858	-0.24926365	FALSE
GB16480	9435	4449	1.084541419	0.220023911	FALSE
GB16481	NA	NA	NA	NA	FALSE
GB16482	527	404	0.383447669	-0.481069839	FALSE
GB16483	77	29	1.408805546	0.544288037	TRUE
GB16484	NA	NA	NA	NA	FALSE
GB16485	2728	1835	0.572063581	-0.292453927	FALSE
GB16486	783	422	0.891769309	0.0272518	FALSE
GB16487	7690	3848	0.998874799	0.134357291	FALSE
GB16488	663	319	1.055452446	0.190934938	FALSE
GB16489	NA	NA	NA	NA	FALSE
GB16490	84	33	1.347923303	0.483405795	FALSE
GB16491	NA	NA	NA	NA	FALSE
GB16492	11	6	0.874469118	0.00995161	FALSE
GB16493	525	450	0.222392421	-0.642125087	TRUE
GB16494	1977	1425	0.472350952	-0.392166557	FALSE
GB16495	366	202	0.857488356	-0.007029153	FALSE
GB16496	565	291	0.957231714	0.092714206	FALSE
GB16497	2473	1399	0.821866277	-0.042651231	FALSE
GB16498	1000	478	1.064917477	0.200399968	FALSE
GB16499	252	156	0.691877705	-0.172639804	FALSE
GB16500	35720	23666	0.593916196	-0.270601312	FALSE
GB16501	NA	NA	NA	NA	FALSE
GB16502	290	197	0.557857271	-0.306660238	FALSE
GB16503	116	39	1.572578776	0.708061268	TRUE
GB16504	268	165	0.699766976	-0.164750532	FALSE
GB16505	71	53	0.421826665	-0.442690843	FALSE
GB16506	923	419	1.139380404	0.274862896	FALSE
GB16507	8812	4597	0.938776829	0.074259321	FALSE
GB16508	352	161	1.128514741	0.263997232	FALSE

GB16509	87	37	1.23349013	0.368972622	FALSE
GB16510	NA	NA	NA	NA	FALSE
GB16511	6	10	-0.736965594	-1.601483102	TRUE
GB16512	408	190	1.102569734	0.238052225	FALSE
GB16513	61	33	0.886343218	0.02182571	FALSE
GB16514	1	1	0	-0.864517508	TRUE
GB16515	540	273	0.984058456	0.119540948	FALSE
GB16516	1914	1049	0.867576152	0.003058644	FALSE
GB16517	5	3	0.736965594	-0.127551914	FALSE
GB16518	40	41	-0.03562391	-0.900141418	TRUE
GB16519	1096	641	0.773851536	-0.090665972	FALSE
GB16520	1688	1367	0.304301661	-0.560215847	TRUE
GB16521	NA	NA	NA	NA	FALSE
GB16522	184	140	0.394278939	-0.470238569	FALSE
GB16523	283	115	1.299168192	0.434650684	FALSE
GB16524	2	0	NA	NA	FALSE
GB16525	391	263	0.572105808	-0.2924117	FALSE
GB16526	3355	1954	0.779882299	-0.084635209	FALSE
GB16527	258	147	0.811554911	-0.052962598	FALSE
GB16528	512	308	0.733213459	-0.131304049	FALSE
GB16529	2199	1096	1.004599806	0.140082298	FALSE
GB16530	1624	1005	0.692356131	-0.172161377	FALSE
GB16531	120	93	0.367731785	-0.496785724	FALSE
GB16532	353	187	0.916629913	0.052112405	FALSE
GB16533	349	263	0.408164237	-0.456353271	FALSE
GB16534	1074	581	0.886383925	0.021866416	FALSE
GB16535	2	2	0	-0.864517508	TRUE
GB16536	86	40	1.10433666	0.239819151	FALSE
GB16537	9121	4885	0.900833444	0.036315935	FALSE
GB16538	223	111	1.006484034	0.141966525	FALSE
GB16539	795	388	1.034898208	0.1703807	FALSE
GB16540	1189	617	0.946406321	0.081888812	FALSE
GB16541	1683	959	0.811432456	-0.053085052	FALSE
GB16542	232	117	0.987616276	0.123098767	FALSE
GB16543	442	247	0.839535328	-0.024982181	FALSE
GB16544	14	8	0.807354922	-0.057162586	FALSE
GB16545	44	17	1.371968777	0.507451269	TRUE
GB16546	5	9	-0.847996907	-1.712514415	TRUE
GB16547	14014	9707	0.529771404	-0.334746104	FALSE
GB16548	14	16	-0.192645078	-1.057162586	TRUE
GB16549	514	243	1.080812046	0.216294537	FALSE
GB16550	1341	872	0.620909197	-0.243608311	FALSE
GB16551	NA	NA	NA	NA	FALSE
GB16552	10623	4902	1.11574886	0.251231352	FALSE

GB16553	0	1	NA	NA	FALSE
GB16554	3515	1970	0.83532906	-0.029188449	FALSE
GB16555	681	328	1.053958983	0.189441475	FALSE
GB16556	13449	5762	1.22285734	0.358339831	FALSE
GB16557	326	181	0.848882267	-0.015635241	FALSE
GB16558	1	5	-2.321928095	-3.186445603	TRUE
GB16559	1493	782	0.932973653	0.068456144	FALSE
GB16560	140	54	1.374395515	0.509878006	TRUE
GB16561	21750	11826	0.879053219	0.01453571	FALSE
GB16562	689	496	0.474163862	-0.390353646	FALSE
GB16563	2304	1530	0.590609064	-0.273908444	FALSE
GB16564	997	532	0.906167259	0.041649751	FALSE
GB16565	37	15	1.30256277	0.438045262	FALSE
GB16566	1989	1146	0.795436232	-0.069081276	FALSE
GB16567	174	103	0.756442969	-0.10807454	FALSE
GB16568	1883	1375	0.453601381	-0.410916127	FALSE
GB16569	528	311	0.763623349	-0.100894159	FALSE
GB16570	77	34	1.179323699	0.314806191	FALSE
GB16571	116	54	1.103093493	0.238575985	FALSE
GB16572	NA	NA	NA	NA	FALSE
GB16573	NA	NA	NA	NA	FALSE
GB16574	1	1	0	-0.864517508	TRUE
GB16575	709	465	0.608554911	-0.255962597	FALSE
GB16576	639	366	0.803972283	-0.060545226	FALSE
GB16577	2808	1844	0.60670428	-0.257813228	FALSE
GB16578	4	4	0	-0.864517508	TRUE
GB16579	36012	22681	0.666993475	-0.197524033	FALSE
GB16580	396	261	0.601450624	-0.263066885	FALSE
GB16581	0	1	NA	NA	FALSE
GB16582	51	30	0.765534746	-0.098982762	FALSE
GB16583	1188	636	0.901436166	0.036918657	FALSE
GB16584	625	386	0.695255342	-0.169262166	FALSE
GB16585	577	356	0.696694078	-0.167823431	FALSE
GB16586	700	361	0.955356085	0.090838577	FALSE
GB16587	3399	1431	1.24808669	0.383569181	FALSE
GB16588	47	30	0.647698256	-0.216819252	FALSE
GB16589	1210	607	0.995238626	0.130721118	FALSE
GB16590	NA	NA	NA	NA	FALSE
GB16591	851	397	1.100020125	0.235502616	FALSE
GB16592	495	342	0.5334322	-0.331085308	FALSE
GB16593	481	293	0.715136229	-0.149381279	FALSE
GB16594	327	211	0.632047637	-0.232469872	FALSE
GB16595	1080	706	0.613291224	-0.251226285	FALSE
GB16596	19	5	1.925999419	1.06148191	TRUE

GB16597	2296	1339	0.777966681	-0.086550827	FALSE
GB16598	1	3	-1.584962501	-2.449480009	TRUE
GB16599	11925	5350	1.15637847	0.291860961	FALSE
GB16600	2922	1222	1.257711893	0.393194385	FALSE
GB16601	2093	1019	1.03841826	0.173900752	FALSE
GB16602	10905	6373	0.774945204	-0.089572304	FALSE
GB16603	4003	2840	0.495190686	-0.369326822	FALSE
GB16604	7204	4547	0.663883177	-0.200634331	FALSE
GB16605	556	280	0.989658056	0.125140547	FALSE
GB16606	129	104	0.310787537	-0.553729971	TRUE
GB16607	205	128	0.6794801	-0.185037409	FALSE
GB16608	129	52	1.310787537	0.446270029	FALSE
GB16609	483	250	0.950095094	0.085577586	FALSE
GB16610	NA	NA	NA	NA	FALSE
GB16611	47	24	0.969626351	0.105108843	FALSE
GB16612	318	179	0.829067178	-0.03545033	FALSE
GB16613	933	498	0.905731339	0.04121383	FALSE
GB16614	3636	2092	0.797469348	-0.06704816	FALSE
GB16615	595	339	0.811604395	-0.052913113	FALSE
GB16616	767	329	1.221138994	0.356621485	FALSE
GB16617	369	188	0.972888154	0.108370646	FALSE
GB16618	1820	1122	0.697865774	-0.166651734	FALSE
GB16619	10087	4956	1.02524907	0.160731562	FALSE
GB16620	959	445	1.107725479	0.243207971	FALSE
GB16621	550	293	0.908530954	0.044013446	FALSE
GB16622	NA	NA	NA	NA	FALSE
GB16623	248	145	0.77428722	-0.090230288	FALSE
GB16624	194	138	0.491388385	-0.373129123	FALSE
GB16625	216	108	1	0.135482492	FALSE
GB16626	240	165	0.540568381	-0.323949127	FALSE
GB16627	1247	571	1.126898814	0.262381306	FALSE
GB16628	27826	18412	0.595787188	-0.26873032	FALSE
GB16629	250	157	0.671163536	-0.193353973	FALSE
GB16630	1	0	NA	NA	FALSE
GB16631	4	3	0.415037499	-0.449480009	FALSE
GB16632	1215	656	0.889188594	0.024671086	FALSE
GB16633	1033	568	0.862877419	-0.001640089	FALSE
GB16634	NA	NA	NA	NA	FALSE
GB16635	562	381	0.560779133	-0.303738376	FALSE
GB16636	329	269	0.290481411	-0.574036097	TRUE
GB16637	2789	1306	1.094593037	0.230075529	FALSE
GB16638	143	71	1.010124217	0.145606709	FALSE
GB16639	1	1	0	-0.864517508	TRUE
GB16640	0	1	NA	NA	FALSE

GB16641	609	371	0.715023041	-0.149494467	FALSE
GB16642	145	107	0.438442104	-0.426075405	FALSE
GB16643	314	205	0.615140649	-0.249376859	FALSE
GB16644	2359	1363	0.791389856	-0.073127652	FALSE
GB16645	415	208	0.996527808	0.1320103	FALSE
GB16646	173	112	0.627273306	-0.237244203	FALSE
GB16647	593	402	0.560836603	-0.303680905	FALSE
GB16648	2247	1042	1.108644847	0.244127339	FALSE
GB16649	1970	1048	0.910556913	0.046039404	FALSE
GB16650	1914	1056	0.857980995	-0.006536513	FALSE
GB16651	553	413	0.421137699	-0.44337981	FALSE
GB16652	570	309	0.883355081	0.018837573	FALSE
GB16653	4	1	2	1.135482492	TRUE
GB16654	51	28	0.86507042	0.000552912	FALSE
GB16655	187	121	0.628031223	-0.236486286	FALSE
GB16656	33	21	0.652076697	-0.212440812	FALSE
GB16657	742	411	0.852280793	-0.012236715	FALSE
GB16658	4	2	1	0.135482492	FALSE
GB16659	1369	792	0.789550111	-0.074967397	FALSE
GB16660	39	37	0.075948853	-0.788568655	TRUE
GB16661	5	1	2.321928095	1.457410587	TRUE
GB16662	17	10	0.765534746	-0.098982762	FALSE
GB16663	1813	896	1.016808288	0.152290779	FALSE
GB16664	11550	7211	0.679621605	-0.184895904	FALSE
GB16665	357	213	0.745070644	-0.119446865	FALSE
GB16666	1361	911	0.579144108	-0.285373401	FALSE
GB16667	NA	NA	NA	NA	FALSE
GB16668	80	38	1.074000581	0.209483073	FALSE
GB16669	126	100	0.333423734	-0.531093775	TRUE
GB16670	0	1	NA	NA	FALSE
GB16671	1058	648	0.707273909	-0.157243599	FALSE
GB16672	626	334	0.906314554	0.041797046	FALSE
GB16673	457	236	0.953407306	0.088889797	FALSE
GB16674	125	83	0.590744853	-0.273772655	FALSE
GB16675	44	16	1.459431619	0.59491411	TRUE
GB16676	350	155	1.175086707	0.310569198	FALSE
GB16677	665	356	0.901477099	0.036959591	FALSE
GB16678	2357	1380	0.772283492	-0.092234017	FALSE
GB16679	437	268	0.705400279	-0.159117229	FALSE
GB16680	816	543	0.587616954	-0.276900554	FALSE
GB16681	134	61	1.135351853	0.270834345	FALSE
GB16682	13	5	1.378511623	0.513994115	TRUE
GB16683	3933	1779	1.144563676	0.280046167	FALSE
GB16684	2901	2498	0.215776819	-0.64874069	TRUE

GB16685	447	226	0.983952059	0.11943455	FALSE
GB16686	7056	2388	1.563047724	0.698530216	TRUE
GB16687	798	402	0.989193245	0.124675737	FALSE
GB16688	111	67	0.728326676	-0.136190832	FALSE
GB16689	361	190	0.925999419	0.06148191	FALSE
GB16690	326	197	0.726676335	-0.137841174	FALSE
GB16691	375	271	0.468597744	-0.395919764	FALSE
GB16692	32	11	1.540568381	0.676050873	TRUE
GB16693	NA	NA	NA	NA	FALSE
GB16694	180	109	0.723668772	-0.140848737	FALSE
GB16695	1	0	NA	NA	FALSE
GB16696	1	1	0	-0.864517508	TRUE
GB16697	667	209	1.674183819	0.809666311	TRUE
GB16698	1	0	NA	NA	FALSE
GB16699	1970	1213	0.699616079	-0.164901429	FALSE
GB16700	188	74	1.345135486	0.480617978	FALSE
GB16701	858	456	0.911943823	0.047426315	FALSE
GB16702	1087	747	0.541171792	-0.323345716	FALSE
GB16703	6	3	1	0.135482492	FALSE
GB16704	1088	628	0.792842092	-0.071675416	FALSE
GB16705	385	232	0.73073364	-0.133783868	FALSE
GB16706	1714	703	1.285770515	0.421253007	FALSE
GB16707	3	3	0	-0.864517508	TRUE
GB16708	86	55	0.644905041	-0.219612467	FALSE
GB16709	2071	1147	0.852462162	-0.012055346	FALSE
GB16710	63	55	0.19592021	-0.668597298	TRUE
GB16711	883	527	0.744610476	-0.119907032	FALSE
GB16712	65	44	0.562936194	-0.301581314	FALSE
GB16713	527	239	1.140792344	0.276274835	FALSE
GB16714	1257	817	0.621576666	-0.242940842	FALSE
GB16715	813	441	0.882476697	0.017959188	FALSE
GB16716	275	123	1.160773303	0.296255795	FALSE
GB16717	1100	543	1.018479421	0.153961912	FALSE
GB16718	52	25	1.056583528	0.19206602	FALSE
GB16719	140	70	1	0.135482492	FALSE
GB16720	1645	923	0.833685031	-0.030832477	FALSE
GB16721	526	290	0.859009899	-0.005507609	FALSE
GB16722	81	62	0.385653692	-0.478863816	FALSE
GB16723	1175	645	0.865289691	0.000772183	FALSE
GB16724	769	329	1.224896014	0.360378506	FALSE
GB16725	1174	961	0.288824072	-0.575693436	TRUE
GB16726	1524	803	0.92439101	0.059873502	FALSE
GB16727	158	90	0.811927652	-0.052589856	FALSE
GB16728	119	59	1.012174714	0.147657206	FALSE

GB16729	765	502	0.607772384	-0.256745125	FALSE
GB16730	25478	14506	0.812602278	-0.051915231	FALSE
GB16731	224	86	1.381090167	0.516572659	TRUE
GB16732	13804	8497	0.700060909	-0.164456599	FALSE
GB16733	546	221	1.304854582	0.440337073	FALSE
GB16734	21	14	0.584962501	-0.279555008	FALSE
GB16735	68	44	0.628031223	-0.236486286	FALSE
GB16736	519	265	0.969742179	0.105224671	FALSE
GB16737	1058	526	1.008204923	0.143687414	FALSE
GB16738	NA	NA	NA	NA	FALSE
GB16739	663	335	0.984847775	0.120330266	FALSE
GB16740	131	109	0.265238677	-0.599278832	TRUE
GB16741	130	60	1.115477217	0.250959709	FALSE
GB16742	959	507	0.919545068	0.05502756	FALSE
GB16743	652	463	0.493859771	-0.370657737	FALSE
GB16744	547	331	0.724709616	-0.139807892	FALSE
GB16745	213	164	0.377157616	-0.487359893	FALSE
GB16746	366	183	1	0.135482492	FALSE
GB16747	860	434	0.986641617	0.122124109	FALSE
GB16748	1214	640	0.923624611	0.059107103	FALSE
GB16749	NA	NA	NA	NA	FALSE
GB16750	35	23	0.605721061	-0.258796447	FALSE
GB16751	3568	2526	0.498260976	-0.366256532	FALSE
GB16752	69	34	1.021061616	0.156544107	FALSE
GB16753	26	7	1.893084796	1.028567288	TRUE
GB16754	585	303	0.949118831	0.084601323	FALSE
GB16755	1	0	NA	NA	FALSE
GB16756	59	11	2.423211431	1.558693922	TRUE
GB16757	2283	1860	0.295628238	-0.56888927	TRUE
GB16758	968	517	0.904842767	0.040325259	FALSE
GB16759	141	68	1.052088511	0.187571003	FALSE
GB16760	1024	746	0.45696818	-0.407549329	FALSE
GB16761	33	15	1.137503524	0.272986015	FALSE
GB16762	2	1	1	0.135482492	FALSE
GB16763	453	318	0.510484285	-0.354033224	FALSE
GB16764	1290	521	1.308015788	0.44349828	FALSE
GB16765	376	212	0.826668397	-0.037849111	FALSE
GB16766	62	53	0.226275856	-0.638241653	TRUE
GB16767	1390	669	1.055006767	0.190489259	FALSE
GB16768	569	306	0.894897	0.030379491	FALSE
GB16769	222	118	0.911772817	0.047255309	FALSE
GB16770	489	323	0.5983003	-0.266217208	FALSE
GB16771	NA	NA	NA	NA	FALSE
GB16772	140	70	1	0.135482492	FALSE

GB16773	1655	553	1.581479831	0.716962323	TRUE
GB16774	26	4	2.700439718	1.83592221	TRUE
GB16775	47	18	1.38466385	0.520146342	TRUE
GB16776	156	40	1.963474124	1.098956616	TRUE
GB16777	3851	2092	0.880350272	0.015832763	FALSE
GB16778	11	5	1.137503524	0.272986015	FALSE
GB16779	1943	1475	0.397570946	-0.466946562	FALSE
GB16780	348	170	1.03355256	0.169035051	FALSE
GB16781	353	222	0.669108507	-0.195409001	FALSE
GB16782	192	85	1.175571565	0.311054056	FALSE
GB16783	465	256	0.861086906	-0.003430602	FALSE
GB16784	52	38	0.452512205	-0.412005304	FALSE
GB16785	37	17	1.121990524	0.257473016	FALSE
GB16786	5953	3665	0.699803696	-0.164713812	FALSE
GB16787	653	394	0.728887362	-0.135630146	FALSE
GB16788	1018	633	0.685460157	-0.179057352	FALSE
GB16789	3758	1990	0.917196636	0.052679128	FALSE
GB16790	1937	916	1.080404451	0.215886942	FALSE
GB16791	174	97	0.843030654	-0.021486855	FALSE
GB16792	2345	1286	0.86669728	0.002179772	FALSE
GB16793	526	263	1	0.135482492	FALSE
GB16794	NA	NA	NA	NA	FALSE
GB16795	18	5	1.847996907	0.983479398	TRUE
GB16796	2145	1236	0.795298904	-0.069218604	FALSE
GB16797	NA	NA	NA	NA	FALSE
GB16798	NA	NA	NA	NA	FALSE
GB16799	580	263	1.140990101	0.276472592	FALSE
GB16800	229	106	1.111283334	0.246765825	FALSE
GB16801	2153	1379	0.642725863	-0.221791646	FALSE
GB16802	1196	811	0.56044357	-0.304073938	FALSE
GB16803	16992	5605	1.600069393	0.735551885	TRUE
GB16804	3	2	0.584962501	-0.279555008	FALSE
GB16805	4237	2079	1.027153371	0.162635862	FALSE
GB16806	101287	60712	0.738395414	-0.126122095	FALSE
GB16807	13193	4549	1.536151321	0.671633813	TRUE
GB16808	1119	618	0.856531293	-0.007986215	FALSE
GB16809	366	117	1.645335119	0.78081761	TRUE
GB16810	691	443	0.641379012	-0.223138496	FALSE
GB16811	746	304	1.295104307	0.430586798	FALSE
GB16812	672	391	0.781292625	-0.083224883	FALSE
GB16813	1511	837	0.852204133	-0.012313376	FALSE
GB16814	14	4	1.807354922	0.942837414	TRUE
GB16815	406	261	0.637429921	-0.227087588	FALSE
GB16816	972	519	0.905221775	0.040704267	FALSE

GB16817	7	1	2.807354922	1.942837414	TRUE
GB16818	479	298	0.684713325	-0.179804183	FALSE
GB16819	NA	NA	NA	NA	FALSE
GB16820	13	10	0.378511623	-0.486005885	FALSE
GB16821	50	26	0.943416472	0.078898963	FALSE
GB16822	2664	1380	0.948925815	0.084408307	FALSE
GB16823	1019	403	1.338302308	0.473784799	FALSE
GB16824	870	450	0.9510904	0.086572891	FALSE
GB16825	335	218	0.619832961	-0.244684548	FALSE
GB16826	112	79	0.503574174	-0.360943334	FALSE
GB16827	178	100	0.831877241	-0.032640267	FALSE
GB16828	628	337	0.898015968	0.033498459	FALSE
GB16829	3061	1737	0.817405291	-0.047112218	FALSE
GB16830	1103	642	0.780787588	-0.08372992	FALSE
GB16831	1282	706	0.860656173	-0.003861335	FALSE
GB16832	1707	836	1.029888211	0.165370703	FALSE
GB16833	437	281	0.637063149	-0.227454359	FALSE
GB16834	1527	873	0.806646503	-0.057871005	FALSE
GB16835	69	35	0.97924144	0.114723932	FALSE
GB16836	198	110	0.847996907	-0.016520602	FALSE
GB16837	NA	NA	NA	NA	FALSE
GB16838	753	419	0.845699621	-0.018817887	FALSE
GB16839	1652	982	0.750418757	-0.114098751	FALSE
GB16840	455	253	0.84672916	-0.017788348	FALSE
GB16841	2	0	NA	NA	FALSE
GB16842	23479	15244	0.62312945	-0.241388058	FALSE
GB16843	519	340	0.610199792	-0.254317716	FALSE
GB16844	155135	98686	0.652606872	-0.211910637	FALSE
GB16845	964	462	1.061140295	0.196622786	FALSE
GB16846	11	4	1.459431619	0.59491411	TRUE
GB16847	2207	998	1.144974909	0.280457401	FALSE
GB16848	718	375	0.937093248	0.07257574	FALSE
GB16849	156	89	0.809668788	-0.05484872	FALSE
GB16850	2983	1688	0.821449074	-0.043068435	FALSE
GB16851	NA	NA	NA	NA	FALSE
GB16852	1036	482	1.103918951	0.239401443	FALSE
GB16853	1985	1163	0.771287911	-0.093229598	FALSE
GB16854	676	313	1.110860589	0.246343081	FALSE
GB16855	649	380	0.77221906	-0.092298449	FALSE
GB16856	343	184	0.89850281	0.033985302	FALSE
GB16857	919	457	1.007870696	0.143353188	FALSE
GB16858	1760	1002	0.81269292	-0.051824588	FALSE
GB16859	46	23	1	0.135482492	FALSE
GB16860	209	125	0.741574847	-0.122942661	FALSE

GB16861	250	117	1.095419565	0.230902057	FALSE
GB16862	476	313	0.604798916	-0.259718592	FALSE
GB16863	173	111	0.640212361	-0.224305147	FALSE
GB16864	5981	3933	0.604756531	-0.259760977	FALSE
GB16865	247	116	1.090386236	0.225868728	FALSE
GB16866	21	7	1.584962501	0.720444992	TRUE
GB16867	53	21	1.335603032	0.471085523	FALSE
GB16868	676	337	1.004274655	0.139757147	FALSE
GB16869	155	75	1.047305715	0.182788206	FALSE
GB16870	47	35	0.425305835	-0.439211674	FALSE
GB16871	601	341	0.817593252	-0.046924257	FALSE
GB16872	181	132	0.455451768	-0.409065741	FALSE
GB16873	918	466	0.978164199	0.11364669	FALSE
GB16874	1	0	NA	NA	FALSE
GB16875	345	210	0.716207034	-0.148310474	FALSE
GB16876	4171	2649	0.654945469	-0.20957204	FALSE
GB16877	643	309	1.057211899	0.192694391	FALSE
GB16878	NA	NA	NA	NA	FALSE
GB16879	803	529	0.602132265	-0.262385243	FALSE
GB16880	353	247	0.515157142	-0.349360367	FALSE
GB16881	35429	17312	1.033158341	0.168640833	FALSE
GB16882	10797	6418	0.750434813	-0.114082695	FALSE
GB16883	103896	55897	0.894297351	0.029779843	FALSE
GB16884	1	2	-1	-1.864517508	TRUE
GB16885	57	35	0.703606997	-0.160910511	FALSE
GB16886	831	486	0.773892163	-0.090625345	FALSE
GB16887	998	487	1.035118043	0.170600535	FALSE
GB16888	NA	NA	NA	NA	FALSE
GB16889	17	14	0.280107919	-0.584409589	TRUE
GB16890	323	157	1.040769606	0.176252097	FALSE
GB16891	460	262	0.812067049	-0.052450459	FALSE
GB16892	544	273	0.9947057	0.130188192	FALSE
GB16893	775	522	0.570146504	-0.294371005	FALSE
GB16894	257	152	0.757697036	-0.106820473	FALSE
GB16895	819	476	0.782901878	-0.08161563	FALSE
GB16896	2160	1286	0.74814067	-0.116376839	FALSE
GB16897	563	280	1.007708095	0.143190587	FALSE
GB16898	1736	1020	0.767197796	-0.097319713	FALSE
GB16899	456	287	0.667983087	-0.196534421	FALSE
GB16900	1	0	NA	NA	FALSE
GB16901	1206	599	1.009601999	0.145084491	FALSE
GB16902	19	11	0.788495895	-0.076021614	FALSE
GB16903	120327	79195	0.60347915	-0.261038359	FALSE
GB16904	860	454	0.921644362	0.057126854	FALSE

GB16905	888	519	0.774825138	-0.08969237	FALSE
GB16906	15	16	-0.093109404	-0.957626913	TRUE
GB16907	999	509	0.972819022	0.108301513	FALSE
GB16908	515	293	0.813671768	-0.050845741	FALSE
GB16909	3892	1796	1.11572436	0.251206852	FALSE
GB16910	5405	2656	1.025039471	0.160521963	FALSE
GB16911	2322	1245	0.89922223	0.034704722	FALSE
GB16912	120	101	0.248679113	-0.615838395	TRUE
GB16913	25	9	1.473931188	0.60941368	TRUE
GB16914	19304	12074	0.676996114	-0.187521394	FALSE
GB16915	NA	NA	NA	NA	FALSE
GB16916	2627	1310	1.003849389	0.13933188	FALSE
GB16917	1066	638	0.740579109	-0.123938399	FALSE
GB16918	1838	1124	0.709494731	-0.155022777	FALSE
GB16919	732	433	0.757476624	-0.107040885	FALSE
GB16920	743	329	1.175274627	0.310757118	FALSE
GB16921	1043	515	1.01809482	0.153577312	FALSE
GB16922	1	1	0	-0.864517508	TRUE
GB16923	4130	2147	0.94381959	0.079302082	FALSE
GB16924	340	200	0.765534746	-0.098982762	FALSE
GB16925	91	44	1.048363022	0.183845513	FALSE
GB16926	376	207	0.861101894	-0.003415614	FALSE
GB16927	5992	2394	1.323614472	0.459096963	FALSE
GB16928	676	375	0.850132651	-0.014384857	FALSE
GB16929	52	22	1.2410081	0.376490591	FALSE
GB16930	361	178	1.020121596	0.155604088	FALSE
GB16931	384	284	0.435215381	-0.429302127	FALSE
GB16932	356	231	0.62398439	-0.240533119	FALSE
GB16933	704	491	0.519852404	-0.344665104	FALSE
GB16934	2462	1365	0.850929811	-0.013587698	FALSE
GB16935	414	311	0.412716187	-0.451801321	FALSE
GB16936	10036	4264	1.234905033	0.370387524	FALSE
GB16937	682	290	1.233718839	0.369201331	FALSE
GB16938	2	0	NA	NA	FALSE
GB16939	208	96	1.115477217	0.250959709	FALSE
GB16940	1380	748	0.883558092	0.019040583	FALSE
GB16941	0	1	NA	NA	FALSE
GB16942	276	149	0.889355936	0.024838428	FALSE
GB16943	443	243	0.866350385	0.001832877	FALSE
GB16944	1306	687	0.926772893	0.062255384	FALSE
GB16945	1009	574	0.813803532	-0.050713976	FALSE
GB16946	1205	592	1.025364065	0.160846557	FALSE
GB16947	40	19	1.074000581	0.209483073	FALSE
GB16948	10	5	1	0.135482492	FALSE

GB16949	866	379	1.192169177	0.327651668	FALSE
GB16950	804	508	0.662367004	-0.202150504	FALSE
GB16951	12221	7549	0.695004889	-0.169512619	FALSE
GB16952	16375	10660	0.619287469	-0.24523004	FALSE
GB16953	170	112	0.602036014	-0.262481494	FALSE
GB16954	267	151	0.822291192	-0.042226316	FALSE
GB16955	1616	925	0.804901927	-0.059615581	FALSE
GB16956	968	581	0.736468884	-0.128048624	FALSE
GB16957	NA	NA	NA	NA	FALSE
GB16958	3180	1627	0.966812514	0.102295006	FALSE
GB16959	37120	24925	0.574603206	-0.289914302	FALSE
GB16960	64	30	1.093109404	0.228591896	FALSE
GB16961	741	488	0.602592395	-0.261925114	FALSE
GB16962	3	1	1.584962501	0.720444992	TRUE
GB16963	15	13	0.206450877	-0.658066631	TRUE
GB16964	611	398	0.618403949	-0.246113559	FALSE
GB16965	1252	878	0.511941717	-0.352575791	FALSE
GB16966	4558	2214	1.041745702	0.177228194	FALSE
GB16967	203	159	0.352452962	-0.512064546	TRUE
GB16968	2719	1552	0.808947594	-0.055569914	FALSE
GB16969	972	589	0.72268868	-0.141828829	FALSE
GB16970	1529	742	1.043097315	0.178579806	FALSE
GB16971	149	111	0.424752654	-0.439764854	FALSE
GB16972	4075	2533	0.685952982	-0.178564526	FALSE
GB16973	14621	6565	1.155175071	0.290657563	FALSE
GB16974	1	1	0	-0.864517508	TRUE
GB16975	235	141	0.736965594	-0.127551914	FALSE
GB16976	475	225	1.078002512	0.213485004	FALSE
GB16977	330	235	0.489805268	-0.374712241	FALSE
GB16978	2087	2088	-0.000691111	-0.86520862	TRUE
GB16979	472	317	0.574304019	-0.290213489	FALSE
GB16980	1703	855	0.99408211	0.129564602	FALSE
GB16981	543	317	0.776469358	-0.088048151	FALSE
GB16982	710	380	0.901819606	0.037302098	FALSE
GB16983	353	245	0.526886434	-0.337631074	FALSE
GB16984	2230	917	1.282050071	0.417532563	FALSE
GB16985	641	352	0.864748928	0.00023142	FALSE
GB16986	1325	1199	0.144160701	-0.720356807	TRUE
GB16987	315	190	0.72935241	-0.135165098	FALSE
GB16988	1450	742	0.966561808	0.1020443	FALSE
GB16989	66	40	0.722466024	-0.142051484	FALSE
GB16990	1056	470	1.167877173	0.303359664	FALSE
GB16991	95	56	0.762500686	-0.102016822	FALSE
GB16992	1062	697	0.607553205	-0.256964303	FALSE

GB16993	1	0	NA	NA	FALSE
GB16994	5626	2668	1.076350886	0.211833378	FALSE
GB16995	755	516	0.549105579	-0.31541193	FALSE
GB16996	350	196	0.836501268	-0.028016241	FALSE
GB16997	1284	746	0.783397667	-0.081119841	FALSE
GB16998	3525	2012	0.808992952	-0.055524556	FALSE
GB16999	5	4	0.321928095	-0.542589413	TRUE
GB17000	8	10	-0.321928095	-1.186445603	TRUE
GB17001	47	16	1.554588852	0.690071343	TRUE
GB17002	505	356	0.504406147	-0.360111362	FALSE
GB17003	403	195	1.047305715	0.182788206	FALSE
GB17004	459	274	0.74431826	-0.120199248	FALSE
GB17005	264	167	0.660689827	-0.203827681	FALSE
GB17006	134	98	0.451379346	-0.413138162	FALSE
GB17007	703	354	0.989775329	0.125257821	FALSE
GB17008	19	9	1.078002512	0.213485004	FALSE
GB17009	NA	NA	NA	NA	FALSE
GB17010	175	68	1.363748271	0.499230762	FALSE
GB17011	687	267	1.363470357	0.498952849	FALSE
GB17012	247	130	0.925999419	0.06148191	FALSE
GB17013	590	273	1.111814003	0.247296495	FALSE
GB17014	421	275	0.614388615	-0.250128894	FALSE
GB17015	58	77	-0.408805546	-1.273323054	TRUE
GB17016	998	599	0.736483813	-0.128033696	FALSE
GB17017	1089	503	1.114373649	0.249856141	FALSE
GB17018	46	38	0.275634443	-0.588883066	TRUE
GB17019	90	43	1.065588342	0.201070833	FALSE
GB17020	79	39	1.018378529	0.153861021	FALSE
GB17021	150	88	0.769387072	-0.095130436	FALSE
GB17022	557	328	0.763981513	-0.100535996	FALSE
GB17023	427	217	0.976541027	0.112023519	FALSE
GB17024	11	5	1.137503524	0.272986015	FALSE
GB17025	5	0	NA	NA	FALSE
GB17026	1508	843	0.839031892	-0.025485616	FALSE
GB17027	661	367	0.848870209	-0.0156473	FALSE
GB17028	94	47	1	0.135482492	FALSE
GB17029	90	58	0.633872101	-0.230645407	FALSE
GB17030	9378	4967	0.916905532	0.052388024	FALSE
GB17031	1110	593	0.904455667	0.039938158	FALSE
GB17032	8	6	0.415037499	-0.449480009	FALSE
GB17033	NA	NA	NA	NA	FALSE
GB17034	999	304	1.716413354	0.851895846	TRUE
GB17035	18251	10924	0.740474294	-0.124043214	FALSE
GB17036	94	73	0.364764293	-0.499753216	FALSE

GB17037	1141	575	0.98866493	0.124147422	FALSE
GB17038	1330	802	0.729752104	-0.134765404	FALSE
GB17039	2602	1491	0.803340704	-0.061176804	FALSE
GB17040	578	335	0.786908397	-0.077609111	FALSE
GB17041	479	225	1.090100655	0.225583146	FALSE
GB17042	2489	1368	0.863498	-0.001019508	FALSE
GB17043	75	30	1.321928095	0.457410587	FALSE
GB17044	1272	974	0.385104993	-0.479412515	FALSE
GB17045	4620	2216	1.05993497	0.195417462	FALSE
GB17046	111	47	1.239827015	0.375309506	FALSE
GB17047	308	160	0.944858446	0.080340937	FALSE
GB17048	383	171	1.163348067	0.298830559	FALSE
GB17049	1	3	-1.584962501	-2.449480009	TRUE
GB17050	968	620	0.642738832	-0.221778676	FALSE
GB17051	394	217	0.860500587	-0.004016921	FALSE
GB17052	328	178	0.881818574	0.017301065	FALSE
GB17053	1	0	NA	NA	FALSE
GB17054	2628	1505	0.804201789	-0.06031572	FALSE
GB17055	6547	3898	0.748099888	-0.11641762	FALSE
GB17056	25953	17288	0.586130337	-0.278387171	FALSE
GB17057	1143	716	0.674793911	-0.189723597	FALSE
GB17058	715	307	1.219704586	0.355187078	FALSE
GB17059	778	491	0.664047131	-0.200470378	FALSE
GB17060	868	415	1.064583706	0.200066198	FALSE
GB17061	2221	1078	1.042852215	0.178334706	FALSE
GB17062	4	0	NA	NA	FALSE
GB17063	306	123	1.314873337	0.450355829	FALSE
GB17064	7	10	-0.514573173	-1.379090681	TRUE
GB17065	311	214	0.539303784	-0.325213725	FALSE
GB17066	608	398	0.611302893	-0.253214615	FALSE
GB17067	5298	3139	0.755142815	-0.109374694	FALSE
GB17068	88	28	1.652076697	0.787559188	TRUE
GB17069	1366	735	0.894141329	0.02962382	FALSE
GB17070	231	161	0.520832163	-0.343685345	FALSE
GB17071	175	113	0.631032149	-0.233485359	FALSE
GB17072	NA	NA	NA	NA	FALSE
GB17073	338	241	0.4879901	-0.376527408	FALSE
GB17074	480	282	0.767339243	-0.097178265	FALSE
GB17075	14649	7238	1.017139171	0.152621663	FALSE
GB17076	605	332	0.865751901	0.001234392	FALSE
GB17077	NA	NA	NA	NA	FALSE
GB17078	0	1	NA	NA	FALSE
GB17079	3036	2260	0.425849018	-0.43866849	FALSE
GB17080	1140	541	1.075333325	0.210815817	FALSE

GB17081	2569	1279	1.006190626	0.141673118	FALSE
GB17082	152	123	0.305413008	-0.5591045	TRUE
GB17083	479	252	0.926601922	0.062084414	FALSE
GB17084	430	230	0.902702799	0.03818529	FALSE
GB17085	1381	678	1.026356141	0.161838633	FALSE
GB17086	82	31	1.403355694	0.538838186	TRUE
GB17087	5359	3175	0.755207224	-0.109310284	FALSE
GB17088	1491	1069	0.480018405	-0.384499104	FALSE
GB17089	992	528	0.909802191	0.045284683	FALSE
GB17090	5	1	2.321928095	1.457410587	TRUE
GB17091	2	2	0	-0.864517508	TRUE
GB17092	1125	444	1.34129342	0.476775911	FALSE
GB17093	836	557	0.585825615	-0.278691894	FALSE
GB17094	205	101	1.021268617	0.156751108	FALSE
GB17095	5718	3660	0.643666971	-0.220850537	FALSE
GB17096	1140	883	0.368548481	-0.495969027	FALSE
GB17097	100	80	0.321928095	-0.542589413	TRUE
GB17098	331	149	1.151518886	0.287001378	FALSE
GB17099	2091	1342	0.63980839	-0.224709118	FALSE
GB17100	4035	2404	0.747131777	-0.117385731	FALSE
GB17101	393	227	0.791837015	-0.072680493	FALSE
GB17102	2364	1137	1.055997781	0.191480273	FALSE
GB17103	1105	526	1.070911665	0.206394157	FALSE
GB17104	17	8	1.087462841	0.222945333	FALSE
GB17105	187	106	0.818974005	-0.045543503	FALSE
GB17106	1118	622	0.845933703	-0.018583806	FALSE
GB17107	38	23	0.724365557	-0.140151951	FALSE
GB17108	17151	12282	0.481747188	-0.38277032	FALSE
GB17109	239	106	1.172946353	0.308428845	FALSE
GB17110	912	554	0.719147848	-0.14536966	FALSE
GB17111	170	77	1.142604395	0.278086887	FALSE
GB17112	499	267	0.902200074	0.037682565	FALSE
GB17113	2966	1891	0.649369235	-0.215148274	FALSE
GB17114	65	23	1.498805857	0.634288349	TRUE
GB17115	1	0	NA	NA	FALSE
GB17116	NA	NA	NA	NA	FALSE
GB17117	208	125	0.734655433	-0.129862075	FALSE
GB17118	834	406	1.038567656	0.174050148	FALSE
GB17119	1328	709	0.905397614	0.040880106	FALSE
GB17120	476	274	0.79678568	-0.067731828	FALSE
GB17121	62	28	1.146841388	0.28232388	FALSE
GB17122	834	600	0.475084883	-0.389432625	FALSE
GB17123	382	174	1.134485332	0.269967824	FALSE
GB17124	1	0	NA	NA	FALSE

GB17125	25347	16684	0.603349787	-0.261167722	FALSE
GB17126	592	353	0.745928992	-0.118588516	FALSE
GB17127	3	7	-1.222392421	-2.08690993	TRUE
GB17128	1389	754	0.881410171	0.016892662	FALSE
GB17129	1644	952	0.78817682	-0.076340688	FALSE
GB17130	131	95	0.463567393	-0.400950115	FALSE
GB17131	1	0	NA	NA	FALSE
GB17132	225	138	0.705256734	-0.159260774	FALSE
GB17133	109	49	1.153474481	0.288956972	FALSE
GB17134	1443	607	1.249302878	0.38478537	FALSE
GB17135	3	1	1.584962501	0.720444992	TRUE
GB17136	4	2	1	0.135482492	FALSE
GB17137	881	442	0.99509565	0.130578141	FALSE
GB17138	25698	14363	0.839298967	-0.025218542	FALSE
GB17139	174	122	0.512206158	-0.35231135	FALSE
GB17140	160	66	1.277533976	0.413016467	FALSE
GB17141	1047	611	0.777017157	-0.087500351	FALSE
GB17142	713	339	1.072616803	0.208099295	FALSE
GB17143	198	80	1.307428525	0.442911017	FALSE
GB17144	1376	744	0.887105944	0.022588435	FALSE
GB17145	357	142	1.330033145	0.465515636	FALSE
GB17146	520	285	0.867549704	0.003032196	FALSE
GB17147	861	608	0.501941914	-0.362575594	FALSE
GB17148	6	7	-0.222392421	-1.08690993	TRUE
GB17149	700	336	1.058893689	0.194376181	FALSE
GB17150	1177	741	0.667568873	-0.196948636	FALSE
GB17151	498	244	1.029264595	0.164747086	FALSE
GB17152	NA	NA	NA	NA	FALSE
GB17153	171	93	0.878693704	0.014176195	FALSE
GB17154	71	27	1.394859617	0.530342109	TRUE
GB17155	1428	596	1.260611744	0.396094235	FALSE
GB17156	280	171	0.711430502	-0.153087006	FALSE
GB17157	1266	529	1.258937777	0.394420269	FALSE
GB17158	8498	4667	0.864627876	0.000110368	FALSE
GB17159	819	501	0.709052848	-0.15546466	FALSE
GB17160	444	293	0.599659012	-0.264858496	FALSE
GB17161	541	216	1.324597282	0.460079773	FALSE
GB17162	1804	1191	0.599025925	-0.265491583	FALSE
GB17163	496	381	0.380549123	-0.483968385	FALSE
GB17164	375	306	0.293358943	-0.571158566	TRUE
GB17165	247	138	0.839842775	-0.024674734	FALSE
GB17166	7383	4792	0.62357925	-0.240938258	FALSE
GB17167	880	384	1.196397213	0.331879704	FALSE
GB17168	657	347	0.920957708	0.056440199	FALSE

GB17169	1638	890	0.880058116	0.015540607	FALSE
GB17170	55	26	1.080919995	0.216402487	FALSE
GB17171	127	53	1.260764232	0.396246724	FALSE
GB17172	1039	544	0.933517098	0.068999589	FALSE
GB17173	49	37	0.405256478	-0.45926103	FALSE
GB17174	359	212	0.759919579	-0.104597929	FALSE
GB17175	2603	1423	0.87123965	0.006722142	FALSE
GB17176	5570	2517	1.145972111	0.281454603	FALSE
GB17177	680	331	1.038703529	0.174186021	FALSE
GB17178	2	2	0	-0.864517508	TRUE
GB17179	50	36	0.473931188	-0.39058632	FALSE
GB17180	0	1	NA	NA	FALSE
GB17181	1116	569	0.97183647	0.107318961	FALSE
GB17182	955	520	0.87698911	0.012471602	FALSE
GB17183	668	389	0.780077947	-0.084439561	FALSE
GB17184	457	260	0.813682542	-0.050834966	FALSE
GB17185	196	70	1.485426827	0.620909319	TRUE
GB17186	141	112	0.33219643	-0.532321078	TRUE
GB17187	606	394	0.621122164	-0.243395344	FALSE
GB17188	2185	871	1.326888656	0.462371147	FALSE
GB17189	NA	NA	NA	NA	FALSE
GB17190	3055	1489	1.036828626	0.172311118	FALSE
GB17191	481	279	0.785771772	-0.078745736	FALSE
GB17192	1256	597	1.073033628	0.208516119	FALSE
GB17193	20	4	2.321928095	1.457410587	TRUE
GB17194	5811	3306	0.81369973	-0.050817778	FALSE
GB17195	1543	906	0.768155107	-0.096362402	FALSE
GB17196	147	71	1.049925225	0.185407717	FALSE
GB17197	NA	NA	NA	NA	FALSE
GB17198	229	163	0.490475634	-0.374041874	FALSE
GB17199	291	157	0.890254594	0.025737086	FALSE
GB17200	843	557	0.597855304	-0.266662205	FALSE
GB17201	116	67	0.791891805	-0.072625704	FALSE
GB17202	NA	NA	NA	NA	FALSE
GB17203	133	128	0.055282436	-0.809235073	TRUE
GB17204	300	105	1.514573173	0.650055665	TRUE
GB17205	1002	490	1.032028854	0.167511346	FALSE
GB17206	1269	888	0.515060487	-0.349457021	FALSE
GB17207	749	327	1.195675083	0.331157575	FALSE
GB17208	753	506	0.57351248	-0.291005028	FALSE
GB17209	637	371	0.779874186	-0.084643323	FALSE
GB17210	988	521	0.923227669	0.058710161	FALSE
GB17211	915	565	0.695520876	-0.168996632	FALSE
GB17212	85	41	1.051838932	0.187321423	FALSE

GB17213	109	37	1.558730959	0.694213451	TRUE
GB17214	29250	19371	0.594538192	-0.269979317	FALSE
GB17215	5	3	0.736965594	-0.127551914	FALSE
GB17216	NA	NA	NA	NA	FALSE
GB17217	3	5	-0.736965594	-1.601483102	TRUE
GB17218	4264	2576	0.727074845	-0.137442664	FALSE
GB17219	NA	NA	NA	NA	FALSE
GB17220	3193	1036	1.62388855	0.759371042	TRUE
GB17221	1229	561	1.13141224	0.266894731	FALSE
GB17222	1342	690	0.959716405	0.095198896	FALSE
GB17223	4080	2373	0.781857052	-0.082660457	FALSE
GB17224	1130	454	1.31555857	0.451041062	FALSE
GB17225	24	22	0.125530882	-0.738986626	TRUE
GB17226	281	140	1.005143303	0.140625795	FALSE
GB17227	457	235	0.959533408	0.0950159	FALSE
GB17228	193	86	1.166192283	0.301674774	FALSE
GB17229	696	304	1.195015982	0.330498474	FALSE
GB17230	461	278	0.729681868	-0.134835641	FALSE
GB17231	15	5	1.584962501	0.720444992	TRUE
GB17232	1482	845	0.810522201	-0.053995307	FALSE
GB17233	468	240	0.963474124	0.098956616	FALSE
GB17234	NA	NA	NA	NA	FALSE
GB17235	985	520	0.921612101	0.057094593	FALSE
GB17236	319	202	0.659201131	-0.205316377	FALSE
GB17237	17	6	1.502500341	0.637982832	TRUE
GB17238	2433	2197	0.147201451	-0.717316058	TRUE
GB17239	4	0	NA	NA	FALSE
GB17240	1098	585	0.908369525	0.043852016	FALSE
GB17241	1112	593	0.907052778	0.04253527	FALSE
GB17242	754	478	0.657553905	-0.206963603	FALSE
GB17243	390	238	0.71251255	-0.152004958	FALSE
GB17244	614	338	0.861215409	-0.003302099	FALSE
GB17245	2	0	NA	NA	FALSE
GB17246	523	333	0.651288769	-0.213228739	FALSE
GB17247	11301	5087	1.15156344	0.287045931	FALSE
GB17248	932	602	0.630566468	-0.23395104	FALSE
GB17249	610	305	1	0.135482492	FALSE
GB17250	266	172	0.629017681	-0.235499828	FALSE
GB17251	18670	10116	0.884082985	0.019565477	FALSE
GB17252	NA	NA	NA	NA	FALSE
GB17253	500	198	1.336427665	0.471910156	FALSE
GB17254	2	2	0	-0.864517508	TRUE
GB17255	2987	1934	0.627109443	-0.237408065	FALSE
GB17256	1301	611	1.090376677	0.225859169	FALSE

GB17257	1969	971	1.01991991	0.155402402	FALSE
GB17258	1247	503	1.30983116	0.445313652	FALSE
GB17259	16	7	1.192645078	0.32812757	FALSE
GB17260	120	80	0.584962501	-0.279555008	FALSE
GB17261	604	412	0.551904212	-0.312613296	FALSE
GB17262	308	244	0.336049203	-0.528468305	TRUE
GB17263	453	224	1.016012318	0.15149481	FALSE
GB17264	81	50	0.695993813	-0.168523695	FALSE
GB17265	NA	NA	NA	NA	FALSE
GB17266	3	1	1.584962501	0.720444992	TRUE
GB17267	1802	1111	0.697740194	-0.166777314	FALSE
GB17268	37	4	3.209453366	2.344935857	TRUE
GB17269	1309	708	0.886643832	0.022126324	FALSE
GB17270	3129	1442	1.117630494	0.253112986	FALSE
GB17271	644	349	0.883833652	0.019316144	FALSE
GB17272	41	39	0.072149786	-0.792367723	TRUE
GB17273	573	444	0.367975462	-0.496542046	FALSE
GB17274	45	25	0.847996907	-0.016520602	FALSE
GB17275	53	24	1.142957954	0.278440446	FALSE
GB17276	336	236	0.509674373	-0.354843135	FALSE
GB17277	562	292	0.944601761	0.080084253	FALSE
GB17278	7	6	0.222392421	-0.642125087	TRUE
GB17279	695	401	0.793410741	-0.071106767	FALSE
GB17280	450	293	0.619024337	-0.245493172	FALSE
GB17281	1431	906	0.659440717	-0.205076792	FALSE
GB17282	68393	53712	0.348604231	-0.515913277	TRUE
GB17283	3349	1956	0.775824006	-0.088693503	FALSE
GB17284	331	183	0.854987569	-0.00952994	FALSE
GB17285	227	145	0.646639397	-0.217878111	FALSE
GB17286	23741	16236	0.548184459	-0.316333049	FALSE
GB17287	92	27	1.768674454	0.904156946	TRUE
GB17288	1266	626	1.016042842	0.151525334	FALSE
GB17289	40758	21652	0.912582967	0.048065459	FALSE
GB17290	1211	782	0.630958352	-0.233559156	FALSE
GB17291	1894	979	0.952055566	0.087538058	FALSE
GB17292	1	0	NA	NA	FALSE
GB17293	7	3	1.222392421	0.357874913	FALSE
GB17294	940	439	1.098439817	0.233922309	FALSE
GB17295	307	176	0.802663227	-0.061854282	FALSE
GB17296	992	374	1.40730185	0.542784342	TRUE
GB17297	NA	NA	NA	NA	FALSE
GB17298	1	1	0	-0.864517508	TRUE
GB17299	60	45	0.415037499	-0.449480009	FALSE
GB17300	305	137	1.154633349	0.290115841	FALSE

GB17301	104	41	1.342887714	0.478370205	FALSE
GB17302	1001	533	0.909234536	0.044717028	FALSE
GB17303	1432	687	1.059649488	0.19513198	FALSE
GB17304	1734	1072	0.693798993	-0.170718516	FALSE
GB17305	3649	2374	0.620181216	-0.244336292	FALSE
GB17306	447	302	0.565726282	-0.298791226	FALSE
GB17307	1	0	NA	NA	FALSE
GB17308	1307	738	0.82456642	-0.039951089	FALSE
GB17309	352	224	0.652076697	-0.212440812	FALSE
GB17310	134	49	1.451379346	0.586861838	TRUE
GB17311	2558	1405	0.864446134	.374553985093e-05	FALSE
GB17312	8	4	1	0.135482492	FALSE
GB17313	1457	876	0.733998102	-0.130519406	FALSE
GB17314	NA	NA	NA	NA	FALSE
GB17315	438	256	0.77478706	-0.089730449	FALSE
GB17316	NA	NA	NA	NA	FALSE
GB17317	2150	1242	0.791671486	-0.072846022	FALSE
GB17318	NA	NA	NA	NA	FALSE
GB17319	1327	803	0.724696478	-0.13982103	FALSE
GB17320	1706	1066	0.678410209	-0.1861073	FALSE
GB17321	1722	1109	0.634825777	-0.229691731	FALSE
GB17322	79	43	0.877515993	0.012998485	FALSE
GB17323	3065	1487	1.043482427	0.178964918	FALSE
GB17324	69	5	3.786596362	2.922078854	TRUE
GB17325	9858	7395	0.414744834	-0.449772675	FALSE
GB17326	6	0	NA	NA	FALSE
GB17327	2630	1466	0.843177696	-0.021339812	FALSE
GB17328	464	193	1.265523958	0.40100645	FALSE
GB17329	80	39	1.036525876	0.172008368	FALSE
GB17330	55	25	1.137503524	0.272986015	FALSE
GB17331	2877	1351	1.090537546	0.226020038	FALSE
GB17332	477	274	0.799813373	-0.064704135	FALSE
GB17333	1053	553	0.929154051	0.064636542	FALSE
GB17334	NA	NA	NA	NA	FALSE
GB17335	3124	1368	1.191326223	0.326808715	FALSE
GB17336	8	1	3	2.135482492	TRUE
GB17337	342	252	0.440572591	-0.423944917	FALSE
GB17338	734	428	0.778169266	-0.086348242	FALSE
GB17339	8	3	1.415037499	0.550519991	TRUE
GB17340	NA	NA	NA	NA	FALSE
GB17341	136	86	0.661198087	-0.203319422	FALSE
GB17342	424	251	0.756376901	-0.108140608	FALSE
GB17343	951	556	0.774360458	-0.09015705	FALSE
GB17344	90	69	0.38332864	-0.481188869	FALSE

GB17345	181	87	1.056902391	0.192384883	FALSE
GB17346	164	136	0.270089163	-0.594428345	TRUE
GB17347	1799	893	1.010463106	0.145945598	FALSE
GB17348	2110	1439	0.552176407	-0.312341101	FALSE
GB17349	6833	3703	0.883824577	0.019307069	FALSE
GB17350	NA	NA	NA	NA	FALSE
GB17351	4148	2092	0.987533043	0.123015534	FALSE
GB17352	121	69	0.81033878	-0.054178728	FALSE
GB17353	8300	4339	0.93574875	0.071231242	FALSE
GB17354	NA	NA	NA	NA	FALSE
GB17355	118	63	0.905363126	0.040845618	FALSE
GB17356	584	213	1.455114939	0.59059743	TRUE
GB17357	17	12	0.502500341	-0.362017168	FALSE
GB17358	NA	NA	NA	NA	FALSE
GB17359	170	113	0.589211974	-0.275305535	FALSE
GB17360	548	261	1.070126086	0.205608578	FALSE
GB17361	3157	1778	0.828298936	-0.036218572	FALSE
GB17362	1	0	NA	NA	FALSE
GB17363	699	491	0.509569431	-0.354948077	FALSE
GB17364	1143	637	0.843460126	-0.021057382	FALSE
GB17365	1395	574	1.28114248	0.416624972	FALSE
GB17366	602	322	0.902702799	0.03818529	FALSE
GB17367	176	86	1.033166864	0.168649356	FALSE
GB17368	1010	476	1.085321814	0.220804306	FALSE
GB17369	273	110	1.311397427	0.446879919	FALSE
GB17370	10	4	1.321928095	0.457410587	FALSE
GB17371	703	415	0.760413353	-0.104104155	FALSE
GB17372	19	8	1.247927513	0.383410005	FALSE
GB17373	405	273	0.569020957	-0.295496551	FALSE
GB17374	420	195	1.106915204	0.242397696	FALSE
GB17375	191	96	0.992466327	0.127948819	FALSE
GB17376	1725	1149	0.586217564	-0.278299944	FALSE
GB17377	6457	3623	0.833679221	-0.030838287	FALSE
GB17378	618	389	0.667836683	-0.196680825	FALSE
GB17379	1915	1360	0.493737741	-0.370779768	FALSE
GB17380	15910	9073	0.810282272	-0.054235237	FALSE
GB17381	443	193	1.198705851	0.334188343	FALSE
GB17382	845	398	1.086182911	0.221665402	FALSE
GB17383	945	507	0.898328582	0.033811074	FALSE
GB17384	300	110	1.447458977	0.582941469	TRUE
GB17385	568	305	0.897081687	0.032564179	FALSE
GB17386	47	21	1.162271429	0.297753921	FALSE
GB17387	157	64	1.294620749	0.430103241	FALSE
GB17388	499	257	0.957271456	0.092753948	FALSE

GB17389	359	236	0.605196984	-0.259320524	FALSE
GB17390	238	155	0.618693358	-0.24582415	FALSE
GB17391	1	2	-1	-1.864517508	TRUE
GB17392	1	1	0	-0.864517508	TRUE
GB17393	1129	551	1.034921262	0.170403754	FALSE
GB17394	1870	952	0.974004791	0.109487283	FALSE
GB17395	1484	633	1.229213687	0.364696179	FALSE
GB17396	22	14	0.652076697	-0.212440812	FALSE
GB17397	62	36	0.784271309	-0.080246199	FALSE
GB17398	96	61	0.654225163	-0.210292345	FALSE
GB17399	1096	752	0.543443231	-0.321074277	FALSE
GB17400	2203	1074	1.036475502	0.171957993	FALSE
GB17401	263	213	0.304209369	-0.560308139	TRUE
GB17402	237	132	0.84434913	-0.020168379	FALSE
GB17403	199	94	1.082035769	0.217518261	FALSE
GB17404	1704	983	0.793662014	-0.070855494	FALSE
GB17405	113	59	0.937535913	0.073018405	FALSE
GB17406	156	72	1.115477217	0.250959709	FALSE
GB17407	3	3	0	-0.864517508	TRUE
GB17408	173	92	0.911066272	0.046548763	FALSE
GB17409	684	352	0.958420896	0.093903388	FALSE
GB17410	339	207	0.711654506	-0.152863003	FALSE
GB17411	1146	629	0.865475122	0.000957614	FALSE
GB17412	1461	644	1.181823585	0.317306076	FALSE
GB17413	717	472	0.603186259	-0.261331249	FALSE
GB17414	1092	730	0.581004487	-0.283513021	FALSE
GB17415	1514	851	0.831134168	-0.03338334	FALSE
GB17416	1	1	0	-0.864517508	TRUE
GB17417	1	0	NA	NA	FALSE
GB17418	194	104	0.899473124	0.034955616	FALSE
GB17419	267	161	0.729779054	-0.134738455	FALSE
GB17420	NA	NA	NA	NA	FALSE
GB17421	4332	2539	0.770772848	-0.09374466	FALSE
GB17422	150	76	0.980891177	0.116373669	FALSE
GB17423	1239	820	0.595480373	-0.269037136	FALSE
GB17424	1899	1007	0.915176222	0.050658714	FALSE
GB17425	316	208	0.60334103	-0.261176478	FALSE
GB17426	645	424	0.605234896	-0.259282613	FALSE
GB17427	2006	1073	0.90267153	0.038154022	FALSE
GB17428	1874	977	0.939690486	0.075172977	FALSE
GB17429	608	329	0.88598374	0.021466231	FALSE
GB17430	655	302	1.116946357	0.252428849	FALSE
GB17431	215	131	0.714769848	-0.14974766	FALSE
GB17432	8484	3905	1.119422073	0.254904564	FALSE

GB17433	7	1	2.807354922	1.942837414	TRUE
GB17434	81	37	1.130396637	0.265879129	FALSE
GB17435	886	505	0.811023311	-0.053494197	FALSE
GB17436	55	23	1.257797757	0.393280249	FALSE
GB17437	97	60	0.693022247	-0.171495262	FALSE
GB17438	102	71	0.522678222	-0.341839286	FALSE
GB17439	1916	1156	0.728956163	-0.135561345	FALSE
GB17440	4827	2976	0.6977523	-0.166765208	FALSE
GB17441	3	3	0	-0.864517508	TRUE
GB17442	463	282	0.715317031	-0.149200477	FALSE
GB17443	2046	1427	0.51982081	-0.344696698	FALSE
GB17444	8258	4131	0.999301358	0.13478385	FALSE
GB17445	56	25	1.163498732	0.298981224	FALSE
GB17446	30	9	1.736965594	0.872448086	TRUE
GB17447	91	46	0.984232684	0.119715176	FALSE
GB17448	1965	1111	0.822670496	-0.041847013	FALSE
GB17449	457	236	0.953407306	0.088889797	FALSE
GB17450	166	91	0.867244791	0.002727283	FALSE
GB17451	886	653	0.440223707	-0.424293801	FALSE
GB17452	295	163	0.85584299	-0.008674518	FALSE
GB17453	1971	1098	0.844049722	-0.020467786	FALSE
GB17454	316	139	1.184839675	0.320322167	FALSE
GB17455	12	2	2.584962501	1.720444992	TRUE
GB17456	2	2	0	-0.864517508	TRUE
GB17457	7	2	1.807354922	0.942837414	TRUE
GB17458	723	512	0.497851837	-0.366665671	FALSE
GB17459	362	245	0.563207948	-0.30130956	FALSE
GB17460	NA	NA	NA	NA	FALSE
GB17461	4457	1950	1.192598837	0.328081329	FALSE
GB17462	587	447	0.393085672	-0.471431836	FALSE
GB17463	1	0	NA	NA	FALSE
GB17464	2164	1630	0.408828535	-0.455688974	FALSE
GB17465	536	372	0.526930379	-0.337587129	FALSE
GB17466	4516	2258	1	0.135482492	FALSE
GB17467	745	432	0.786209113	-0.078308395	FALSE
GB17468	146	84	0.797507136	-0.067010372	FALSE
GB17469	12429	6495	0.936308795	0.071791287	FALSE
GB17470	190	110	0.788495895	-0.076021614	FALSE
GB17471	893	491	0.862937151	-0.001580358	FALSE
GB17472	248	115	1.108706259	0.244188751	FALSE
GB17473	10245	7382	0.472836342	-0.391681167	FALSE
GB17474	604	282	1.098853387	0.234335879	FALSE
GB17475	0	2	NA	NA	FALSE
GB17476	1198	819	0.548692551	-0.315824957	FALSE

GB17477	748	368	1.023332504	0.158814996	FALSE
GB17478	321	156	1.041027268	0.17650976	FALSE
GB17479	NA	NA	NA	NA	FALSE
GB17480	3376	1793	0.912939416	0.048421908	FALSE
GB17481	4805	2742	0.80930786	-0.055209648	FALSE
GB17482	38	18	1.078002512	0.213485004	FALSE
GB17483	331	173	0.936059179	0.071541671	FALSE
GB17484	2345	1420	0.723696993	-0.140820515	FALSE
GB17485	34	27	0.332575339	-0.531942169	TRUE
GB17486	261	116	1.169925001	0.305407493	FALSE
GB17487	1286	709	0.85903311	-0.005484398	FALSE
GB17488	12821	8032	0.674677618	-0.18983989	FALSE
GB17489	799	336	1.24973427	0.385216762	FALSE
GB17490	87	46	0.91938154	0.054864031	FALSE
GB17491	1778	1314	0.436290049	-0.42822746	FALSE
GB17492	589	310	0.925999419	0.06148191	FALSE
GB17493	173	65	1.412260415	0.547742906	TRUE
GB17494	969	550	0.817065047	-0.047452461	FALSE
GB17495	70	36	0.959358016	0.094840507	FALSE
GB17496	1030	462	1.156679581	0.292162072	FALSE
GB17497	74	42	0.817135943	-0.047381565	FALSE
GB17498	2230	1428	0.643047731	-0.221469778	FALSE
GB17499	29714	18419	0.689948092	-0.174569416	FALSE
GB17500	14718	7523	0.968201644	0.103684136	FALSE
GB17501	90	40	1.169925001	0.305407493	FALSE
GB17502	1234	756	0.706884255	-0.157633253	FALSE
GB17503	1615	1066	0.599326727	-0.265190782	FALSE
GB17504	0	1	NA	NA	FALSE
GB17505	4	2	1	0.135482492	FALSE
GB17506	27	26	0.054447784	-0.810069724	TRUE
GB17507	NA	NA	NA	NA	FALSE
GB17508	3176	2075	0.614099576	-0.250417932	FALSE
GB17509	545	279	0.965991108	0.1014736	FALSE
GB17510	531	291	0.867692708	0.0031752	FALSE
GB17511	3174	2284	0.474739477	-0.389778031	FALSE
GB17512	2063	1256	0.715907357	-0.148610151	FALSE
GB17513	34	19	0.839535328	-0.024982181	FALSE
GB17514	911	502	0.85976369	-0.004753818	FALSE
GB17515	1660	910	0.867244791	0.002727283	FALSE
GB17516	345	195	0.823122238	-0.04139527	FALSE
GB17517	1475	508	1.537814552	0.673297044	TRUE
GB17518	28	26	0.106915204	-0.757602304	TRUE
GB17519	3	3	0	-0.864517508	TRUE
GB17520	589	315	0.902915805	0.038398297	FALSE

GB17521	NA	NA	NA	NA	FALSE
GB17522	1456	804	0.856742949	-0.007774559	FALSE
GB17523	888	509	0.80289402	-0.061623488	FALSE
GB17524	971	688	0.497062731	-0.367454778	FALSE
GB17525	1400	864	0.69632361	-0.168193899	FALSE
GB17526	497	350	0.50589093	-0.358626579	FALSE
GB17527	426	234	0.864344901	-0.000172608	FALSE
GB17528	699	426	0.714439025	-0.150078483	FALSE
GB17529	1966	1004	0.969504052	0.104986544	FALSE
GB17530	8864	5878	0.592632522	-0.271884986	FALSE
GB17531	3749	2126	0.818364229	-0.04615328	FALSE
GB17532	182	62	1.55359833	0.689080821	TRUE
GB17533	558	306	0.866733469	0.002215961	FALSE
GB17534	2551	1536	0.731884683	-0.132632825	FALSE
GB17535	10	7	0.514573173	-0.349944335	FALSE
GB17536	1150	623	0.884329793	0.019812284	FALSE
GB17537	983	668	0.557343314	-0.307174194	FALSE
GB17538	280	138	1.02075856	0.156241052	FALSE
GB17539	677	353	0.93948765	0.074970142	FALSE
GB17540	384	241	0.672073164	-0.192444344	FALSE
GB17541	6590	3225	1.030979305	0.166461796	FALSE
GB17542	510	307	0.732258591	-0.132258917	FALSE
GB17543	189	130	0.539874611	-0.324642897	FALSE
GB17544	2010	1159	0.794314935	-0.070202573	FALSE
GB17545	1	0	NA	NA	FALSE
GB17546	1	0	NA	NA	FALSE
GB17547	37	23	0.68589141	-0.178626099	FALSE
GB17548	2316	2087	0.150204653	-0.714312855	TRUE
GB17549	3972	2743	0.534111001	-0.330406508	FALSE
GB17550	2715	1750	0.633597276	-0.230920232	FALSE
GB17551	15	3	2.321928095	1.457410587	TRUE
GB17552	830	479	0.79308568	-0.071431828	FALSE
GB17553	1181	653	0.854854068	-0.00966344	FALSE
GB17554	1	0	NA	NA	FALSE
GB17555	18143	8095	1.164310044	0.299792536	FALSE
GB17556	5	10	-1	-1.864517508	TRUE
GB17557	2630	1355	0.956769948	0.09225244	FALSE
GB17558	8379	4823	0.796847264	-0.067670244	FALSE
GB17559	1510	744	1.021174023	0.156656515	FALSE
GB17560	7	2	1.807354922	0.942837414	TRUE
GB17561	272	229	0.248259053	-0.616258455	TRUE
GB17562	1066	723	0.560139886	-0.304377623	FALSE
GB17563	2428	1211	1.003569557	0.139052048	FALSE
GB17564	16484	10969	0.587634362	-0.276883147	FALSE

GB17565	488	233	1.066551193	0.202033685	FALSE
GB17566	1070	661	0.69488862	-0.169628889	FALSE
GB17567	6	7	-0.222392421	-1.08690993	TRUE
GB17568	6	5	0.263034406	-0.601483102	TRUE
GB17569	143	85	0.750480401	-0.114037108	FALSE
GB17570	NA	NA	NA	NA	FALSE
GB17571	431	295	0.546972915	-0.317544593	FALSE
GB17572	983	623	0.657959253	-0.206558255	FALSE
GB17573	403	276	0.546111572	-0.318405937	FALSE
GB17574	60	43	0.480625841	-0.383891667	FALSE
GB17575	40	21	0.929610672	0.065093164	FALSE
GB17576	21	24	-0.192645078	-1.057162586	TRUE
GB17577	643	289	1.153749245	0.289231736	FALSE
GB17578	NA	NA	NA	NA	FALSE
GB17579	9575	3345	1.517266276	0.652748768	TRUE
GB17580	4	2	1	0.135482492	FALSE
GB17581	273	150	0.86393845	-0.000579058	FALSE
GB17582	1245	501	1.313263234	0.448745725	FALSE
GB17583	771	382	1.013158222	0.148640714	FALSE
GB17584	768	463	0.730094117	-0.134423391	FALSE
GB17585	512	288	0.830074999	-0.03444251	FALSE
GB17586	132	65	1.022026306	0.157508798	FALSE
GB17587	132	75	0.815575429	-0.048942079	FALSE
GB17588	38540	13859	1.475533408	0.6110159	TRUE
GB17589	13	9	0.530514717	-0.334002792	FALSE
GB17590	77	22	1.807354922	0.942837414	TRUE
GB17591	650	372	0.805137097	-0.059380412	FALSE
GB17592	5892	4766	0.305978479	-0.558539029	TRUE
GB17593	1	1	0	-0.864517508	TRUE
GB17594	379	259	0.54924575	-0.315271758	FALSE
GB17595	1648	1114	0.56496701	-0.299550498	FALSE
GB17596	454	327	0.473401662	-0.391115847	FALSE
GB17597	387	156	1.310787537	0.446270029	FALSE
GB17598	66	76	-0.203533394	-1.068050902	TRUE
GB17599	63	50	0.333423734	-0.531093775	TRUE
GB17600	179	97	0.883902935	0.019385427	FALSE
GB17601	895	558	0.68162256	-0.182894948	FALSE
GB17602	1078	839	0.361614462	-0.502903046	TRUE
GB17603	365	233	0.647566509	-0.216950999	FALSE
GB17604	217	93	1.222392421	0.357874913	FALSE
GB17605	221	103	1.101402032	0.236884524	FALSE
GB17606	339	201	0.754089772	-0.110427736	FALSE
GB17607	70	24	1.544320516	0.679803008	TRUE
GB17608	1305	807	0.693409228	-0.17110828	FALSE

GB17609	1256	832	0.594181031	-0.270336478	FALSE
GB17610	3979	2270	0.809713602	-0.054803907	FALSE
GB17611	266	194	0.455369593	-0.409147915	FALSE
GB17612	1868	978	0.933588085	0.069070576	FALSE
GB17613	32	22	0.540568381	-0.323949127	FALSE
GB17614	3396	2080	0.707252931	-0.157264578	FALSE
GB17615	1	3	-1.584962501	-2.449480009	TRUE
GB17616	258	144	0.841302254	-0.023215254	FALSE
GB17617	4252	2204	0.948017373	0.083499865	FALSE
GB17618	54	18	1.584962501	0.720444992	TRUE
GB17619	2768	1637	0.757789621	-0.106727887	FALSE
GB17620	144	65	1.147557188	0.28303968	FALSE
GB17621	718	301	1.254220357	0.389702849	FALSE
GB17622	104	52	1	0.135482492	FALSE
GB17623	306	191	0.679959015	-0.184558494	FALSE
GB17624	793	550	0.527889247	-0.336628261	FALSE
GB17625	494	225	1.13458604	0.270068532	FALSE
GB17626	3609	2814	0.358976815	-0.505540694	TRUE
GB17627	3916	2130	0.878527334	0.014009826	FALSE
GB17628	NA	NA	NA	NA	FALSE
GB17629	38482	25177	0.612077394	-0.252440114	FALSE
GB17630	525	429	0.291339775	-0.573177733	TRUE
GB17631	8	6	0.415037499	-0.449480009	FALSE
GB17632	1198	569	1.07412735	0.209609842	FALSE
GB17633	142	115	0.304257069	-0.56026044	TRUE
GB17634	494	291	0.763491889	-0.10102562	FALSE
GB17635	3020	1491	1.018268292	0.153750784	FALSE
GB17636	201	148	0.441598326	-0.422919183	FALSE
GB17637	689	581	0.245965819	-0.618551689	TRUE
GB17638	113	43	1.393914208	0.529396699	TRUE
GB17639	325	224	0.536940986	-0.327576522	FALSE
GB17640	942	394	1.25753143	0.393013922	FALSE
GB17641	5396	3142	0.780207168	-0.084310341	FALSE
GB17642	1	1	0	-0.864517508	TRUE
GB17643	604	355	0.766729525	-0.097787983	FALSE
GB17644	266	153	0.797894593	-0.066622916	FALSE
GB17645	860	449	0.937621215	0.073103707	FALSE
GB17646	120	81	0.567040593	-0.297476916	FALSE
GB17647	423	259	0.707705565	-0.156811943	FALSE
GB17648	8433	4267	0.982823844	0.118306336	FALSE
GB17649	5	4	0.321928095	-0.542589413	TRUE
GB17650	2043	1156	0.821547806	-0.042969702	FALSE
GB17651	5	2	1.321928095	0.457410587	FALSE
GB17652	252	139	0.858338851	-0.006178658	FALSE

GB17653	5170	3710	0.478745094	-0.385772415	FALSE
GB17654	177	128	0.46760555	-0.396911958	FALSE
GB17655	29	12	1.273018494	0.408500986	FALSE
GB17656	NA	NA	NA	NA	FALSE
GB17657	88	42	1.067114196	0.202596688	FALSE
GB17658	652	354	0.881122604	0.016605096	FALSE
GB17659	413	247	0.74163074	-0.122886768	FALSE
GB17660	3431	1904	0.849595647	-0.014921861	FALSE
GB17661	1192	665	0.84195799	-0.022559518	FALSE
GB17662	13	9	0.530514717	-0.334002792	FALSE
GB17663	737	319	1.208108195	0.343590687	FALSE
GB17664	3691	1619	1.188908752	0.324391244	FALSE
GB17665	1002	547	0.87326977	0.008752262	FALSE
GB17666	4	2	1	0.135482492	FALSE
GB17667	54	19	1.506959989	0.64244248	TRUE
GB17668	25485	16388	0.637008555	-0.227508953	FALSE
GB17669	355	205	0.792195115	-0.072322393	FALSE
GB17670	970	703	0.464460058	-0.40005745	FALSE
GB17671	4	1	2	1.135482492	TRUE
GB17672	1455	876	0.732016378	-0.13250113	FALSE
GB17673	5457	2186	1.319814643	0.455297134	FALSE
GB17674	160	85	0.912537159	0.04801965	FALSE
GB17675	220	112	0.974004791	0.109487283	FALSE
GB17676	1703	1044	0.705956723	-0.158560785	FALSE
GB17677	388	175	1.14870173	0.284184222	FALSE
GB17678	229	113	1.019024826	0.154507317	FALSE
GB17679	31	16	0.95419631	0.089678802	FALSE
GB17680	3001	1466	1.033558215	0.169040707	FALSE
GB17681	95796	49759	0.945007925	0.080490416	FALSE
GB17682	63	31	1.023083613	0.158566105	FALSE
GB17683	NA	NA	NA	NA	FALSE
GB17684	8319	6330	0.394204617	-0.470312891	FALSE
GB17685	201	149	0.431883171	-0.432634338	FALSE
GB17686	593	368	0.688326338	-0.17619117	FALSE
GB17687	722	316	1.192074279	0.32755677	FALSE
GB17688	418	297	0.493040011	-0.371477497	FALSE
GB17689	8	6	0.415037499	-0.449480009	FALSE
GB17690	580	326	0.831180936	-0.033336573	FALSE
GB17691	1123	566	0.98848397	0.123966461	FALSE
GB17692	130	71	0.872620694	0.008103185	FALSE
GB17693	560	326	0.780554863	-0.083962646	FALSE
GB17694	8384	5192	0.691348334	-0.173169175	FALSE
GB17695	837	486	0.784271309	-0.080246199	FALSE
GB17696	770	403	0.934078607	0.069561099	FALSE

GB17697	NA	NA	NA	NA	FALSE
GB17698	2141	986	1.118625244	0.254107736	FALSE
GB17699	717	493	0.540385472	-0.324132036	FALSE
GB17700	1174	716	0.713400916	-0.151116592	FALSE
GB17701	1332	731	0.865650771	0.001133263	FALSE
GB17702	564	401	0.492092926	-0.372424582	FALSE
GB17703	622	300	1.05195208	0.187434571	FALSE
GB17704	26	14	0.893084796	0.028567288	FALSE
GB17705	915	623	0.55453958	-0.309977928	FALSE
GB17706	301	178	0.757886246	-0.106631263	FALSE
GB17707	395	221	0.837806284	-0.026711225	FALSE
GB17708	275	145	0.923378718	0.05886121	FALSE
GB17709	2062	1195	0.787033714	-0.077483794	FALSE
GB17710	1029	532	0.951744831	0.087227323	FALSE
GB17711	2	0	NA	NA	FALSE
GB17712	485	258	0.910613682	0.046096173	FALSE
GB17713	2167	1084	0.999334397	0.134816888	FALSE
GB17714	641	296	1.114727181	0.250209673	FALSE
GB17715	1336	889	0.587664684	-0.276852825	FALSE
GB17716	202	170	0.248820547	-0.615696962	TRUE
GB17717	311	172	0.854506015	-0.010011493	FALSE
GB17718	102	39	1.387023123	0.522505615	TRUE
GB17719	77	43	0.840521786	-0.023995722	FALSE
GB17720	152	112	0.440572591	-0.423944917	FALSE
GB17721	467	296	0.657825374	-0.206692134	FALSE
GB17722	1	4	-2	-2.864517508	TRUE
GB17723	NA	NA	NA	NA	FALSE
GB17724	42	8	2.392317423	1.527799914	TRUE
GB17725	678	353	0.94161709	0.077099582	FALSE
GB17726	705	419	0.750673014	-0.113844495	FALSE
GB17727	315	186	0.760049207	-0.104468301	FALSE
GB17728	882	605	0.543843513	-0.320673995	FALSE
GB17729	3	1	1.584962501	0.720444992	TRUE
GB17730	183	104	0.81526012	-0.049257388	FALSE
GB17731	1643	874	0.910627295	0.046109787	FALSE
GB17732	1	0	NA	NA	FALSE
GB17733	2693	1134	1.24779359	0.383276081	FALSE
GB17734	2195	1011	1.118437943	0.253920434	FALSE
GB17735	420	298	0.495076997	-0.369440511	FALSE
GB17736	11372	9432	0.269850381	-0.594667128	TRUE
GB17737	1106	581	0.928741317	0.064223809	FALSE
GB17738	819	451	0.860736018	-0.00378149	FALSE
GB17739	45385	22068	1.040259672	0.175742163	FALSE
GB17740	839	413	1.022529029	0.158011521	FALSE

GB17741	348	174	1	0.135482492	FALSE
GB17742	376	200	0.910732662	0.046215154	FALSE
GB17743	1647	913	0.85115379	-0.013363719	FALSE
GB17744	237	104	1.188303531	0.323786022	FALSE
GB17745	967	340	1.507981143	0.643463635	TRUE
GB17746	202	166	0.283172051	-0.581345457	TRUE
GB17747	NA	NA	NA	NA	FALSE
GB17748	NA	NA	NA	NA	FALSE
GB17749	603	417	0.532110618	-0.33240689	FALSE
GB17750	231	158	0.547968293	-0.316549215	FALSE
GB17751	119	100	0.250961574	-0.613555935	TRUE
GB17752	1280	426	1.587218475	0.722700966	TRUE
GB17753	27763	13864	1.001819911	0.137302402	FALSE
GB17754	244	151	0.692332598	-0.17218491	FALSE
GB17755	NA	NA	NA	NA	FALSE
GB17756	755	414	0.866845877	0.002328368	FALSE
GB17757	641	421	0.606504124	-0.258013385	FALSE
GB17758	68	61	0.156725504	-0.707792005	TRUE
GB17759	1675	831	1.011240713	0.146723205	FALSE
GB17760	NA	NA	NA	NA	FALSE
GB17761	3354	1432	1.227851196	0.363333688	FALSE
GB17762	1402	769	0.866430846	0.001913338	FALSE
GB17763	1020	439	1.216276307	0.351758799	FALSE
GB17764	3903	2083	0.905920623	0.041403115	FALSE
GB17765	1	0	NA	NA	FALSE
GB17766	8	7	0.192645078	-0.67187243	TRUE
GB17767	505	356	0.504406147	-0.360111362	FALSE
GB17768	842	480	0.810785827	-0.053731681	FALSE
GB17769	909	495	0.876851769	0.012334261	FALSE
GB17770	449	292	0.620747076	-0.243770432	FALSE
GB17771	811	416	0.963118386	0.098600878	FALSE
GB17772	51	28	0.86507042	0.000552912	FALSE
GB17773	1557	898	0.793981594	-0.070535914	FALSE
GB17774	10	5	1	0.135482492	FALSE
GB17775	3	0	NA	NA	FALSE
GB17776	1289	700	0.880825437	0.016307928	FALSE
GB17777	227	116	0.968567492	0.104049984	FALSE
GB17778	1275	671	0.926112576	0.061595067	FALSE
GB17779	159	101	0.654671473	-0.209846036	FALSE
GB17780	15	3	2.321928095	1.457410587	TRUE
GB17781	345	162	1.090602549	0.22608504	FALSE
GB17782	206	136	0.599037686	-0.265479822	FALSE
GB17783	937	519	0.852314509	-0.012202999	FALSE
GB17784	225	119	0.918963428	0.05444592	FALSE

GB17785	844	517	0.707078718	-0.15743879	FALSE
GB17786	959	630	0.606178987	-0.258338522	FALSE
GB17787	3002	1959	0.615806579	-0.248710929	FALSE
GB17788	581	303	0.93922037	0.074702862	FALSE
GB17789	448	198	1.177998302	0.313480794	FALSE
GB17790	437	280	0.642206453	-0.222311056	FALSE
GB17791	772	481	0.682563953	-0.181953555	FALSE
GB17792	3503	1660	1.077407747	0.212890238	FALSE
GB17793	16268	7597	1.09853517	0.234017661	FALSE
GB17794	87	30	1.5360529	0.671535392	TRUE
GB17795	405	177	1.194172548	0.329655039	FALSE
GB17796	2	0	NA	NA	FALSE
GB17797	564	286	0.979680016	0.115162507	FALSE
GB17798	1675	944	0.827302331	-0.037215177	FALSE
GB17799	300	155	0.952694285	0.088176777	FALSE
GB17800	239	140	0.771583791	-0.092933717	FALSE
GB17801	1326	656	1.015313055	0.150795547	FALSE
GB17802	7894	4104	0.943725788	0.07920828	FALSE
GB17803	672	432	0.637429921	-0.227087588	FALSE
GB17804	513	304	0.754887502	-0.109630006	FALSE
GB17805	483	291	0.731004036	-0.133513472	FALSE
GB17806	80	63	0.344648171	-0.519869337	TRUE
GB17807	12	7	0.777607579	-0.08690993	FALSE
GB17808	743	488	0.606481063	-0.258036445	FALSE
GB17809	142	101	0.491535637	-0.372981872	FALSE
GB17810	796	485	0.714783683	-0.149733825	FALSE
GB17811	189	70	1.432959407	0.568441899	TRUE
GB17812	328	142	1.207804885	0.343287377	FALSE
GB17813	NA	NA	NA	NA	FALSE
GB17814	44	29	0.601450624	-0.263066885	FALSE
GB17815	96	56	0.777607579	-0.08690993	FALSE
GB17816	826	496	0.735801661	-0.128715847	FALSE
GB17817	875	479	0.869257361	0.004739853	FALSE
GB17818	29916	15116	0.984840865	0.120323357	FALSE
GB17819	12	11	0.125530882	-0.738986626	TRUE
GB17820	224	138	0.698830465	-0.165687043	FALSE
GB17821	3	4	-0.415037499	-1.279555008	TRUE
GB17822	742	419	0.824468943	-0.040048565	FALSE
GB17823	25	14	0.836501268	-0.028016241	FALSE
GB17824	93	90	0.047305715	-0.817211794	TRUE
GB17825	1453	887	0.712028693	-0.152488815	FALSE
GB17826	1153	643	0.84250187	-0.022015638	FALSE
GB17827	170	72	1.239465935	0.374948426	FALSE
GB17828	476	253	0.911824189	0.04730668	FALSE

GB17829	1094	542	1.013247981	0.148730473	FALSE
GB17830	14	7	1	0.135482492	FALSE
GB17831	1678	1069	0.650480863	-0.214036646	FALSE
GB17832	91	49	0.893084796	0.028567288	FALSE
GB17833	419	256	0.710806434	-0.153711075	FALSE
GB17834	120	58	1.0489096	0.184392092	FALSE
GB17835	195	96	1.022367813	0.157850305	FALSE
GB17836	2048	1282	0.675819453	-0.188698055	FALSE
GB17837	1644	935	0.814172029	-0.050345479	FALSE
GB17838	111	62	0.840219556	-0.024297952	FALSE
GB17839	114	67	0.766800824	-0.097716685	FALSE
GB17840	999	616	0.697554327	-0.166963181	FALSE
GB17841	2	0	NA	NA	FALSE
GB17842	217	159	0.448668277	-0.415849231	FALSE
GB17843	2063	1132	0.865869863	0.001352355	FALSE
GB17844	91	61	0.577057303	-0.287460206	FALSE
GB17845	1028	421	1.287948126	0.423430618	FALSE
GB17846	166	50	1.731183242	0.866665733	TRUE
GB17847	6	4	0.584962501	-0.279555008	FALSE
GB17848	731	418	0.806368464	-0.058149044	FALSE
GB17849	691	245	1.495903961	0.631386453	TRUE
GB17850	746	421	0.825355397	-0.039162111	FALSE
GB17851	256	116	1.142019005	0.277501497	FALSE
GB17852	12329	7888	0.644324331	-0.220193177	FALSE
GB17853	405	222	0.867362231	0.002844723	FALSE
GB17854	160	91	0.814133455	-0.050384054	FALSE
GB17855	6	2	1.584962501	0.720444992	TRUE
GB17856	14	5	1.485426827	0.620909319	TRUE
GB17857	749	452	0.728642946	-0.135874562	FALSE
GB17858	228	124	0.878693704	0.014176195	FALSE
GB17859	93	69	0.430634354	-0.433883154	FALSE
GB17860	20	12	0.736965594	-0.127551914	FALSE
GB17861	155	87	0.833180909	-0.031336599	FALSE
GB17862	600	307	0.966723845	0.102206337	FALSE
GB17863	460	267	0.784794119	-0.079723389	FALSE
GB17864	37057	19986	0.890756331	0.026238823	FALSE
GB17865	556	293	0.924184218	0.05966671	FALSE
GB17866	736	405	0.861783858	-0.00273365	FALSE
GB17867	119	46	1.371255807	0.506738299	TRUE
GB17868	4232	2827	0.582067744	-0.282449764	FALSE
GB17869	53	45	0.236067358	-0.62845015	TRUE
GB17870	539	338	0.673262026	-0.191255482	FALSE
GB17871	NA	NA	NA	NA	FALSE
GB17872	264	178	0.568660688	-0.29585682	FALSE

GB17873	46	23	1	0.135482492	FALSE
GB17874	347	198	0.809435232	-0.055082276	FALSE
GB17875	13	8	0.700439718	-0.16407779	FALSE
GB17876	5359	3046	0.815047874	-0.049469634	FALSE
GB17877	1788	1131	0.660747807	-0.203769701	FALSE
GB17878	531	301	0.818948374	-0.045569134	FALSE
GB17879	5976	2590	1.20622805	0.341710542	FALSE
GB17880	NA	NA	NA	NA	FALSE
GB17881	183	135	0.438884241	-0.425633267	FALSE
GB17882	1193	660	0.854056113	-0.010461395	FALSE
GB17883	557	348	0.678590021	-0.185927487	FALSE
GB17884	552	285	0.953706348	0.089188839	FALSE
GB17885	50	26	0.943416472	0.078898963	FALSE
GB17886	203	152	0.417408404	-0.447109105	FALSE
GB17887	2202	1366	0.688856985	-0.175660523	FALSE
GB17888	12	15	-0.321928095	-1.186445603	TRUE
GB17889	1960	1014	0.950796002	0.086278494	FALSE
GB17890	397	255	0.63864176	-0.225875748	FALSE
GB17891	NA	NA	NA	NA	FALSE
GB17892	1913	981	0.963511832	0.098994324	FALSE
GB17893	1301	754	0.786984533	-0.077532975	FALSE
GB17894	422	206	1.034598662	0.170081153	FALSE
GB17895	1045	692	0.594658999	-0.269858509	FALSE
GB17896	145	90	0.688055994	-0.176461515	FALSE
GB17897	348	152	1.195015982	0.330498474	FALSE
GB17898	72	25	1.526068812	0.661551303	TRUE
GB17899	1052	651	0.692405256	-0.172112252	FALSE
GB17900	2650	1235	1.101481318	0.23696381	FALSE
GB17901	1159	567	1.031459926	0.166942418	FALSE
GB17902	2867	1641	0.804966666	-0.059550843	FALSE
GB17903	102	48	1.087462841	0.222945333	FALSE
GB17904	21	13	0.691877705	-0.172639804	FALSE
GB17905	3466	1641	1.078696416	0.214178907	FALSE
GB17906	4029	1907	1.079116962	0.214599454	FALSE
GB17907	599	318	0.913529238	0.049011729	FALSE
GB17908	577	359	0.684587475	-0.179930034	FALSE
GB17909	460	300	0.61667136	-0.247846148	FALSE
GB17910	298	160	0.897240426	0.032722917	FALSE
GB17911	68	73	-0.102361718	-0.966879226	TRUE
GB17912	50	31	0.689659879	-0.174857629	FALSE
GB17913	510	220	1.212993723	0.348476215	FALSE
GB17914	NA	NA	NA	NA	FALSE
GB17915	NA	NA	NA	NA	FALSE
GB17916	NA	NA	NA	NA	FALSE

GB17917	7434	3943	0.914844978	0.050327469	FALSE
GB17918	NA	NA	NA	NA	FALSE
GB17919	3332	1802	0.88678939	0.022271881	FALSE
GB17920	2	0	NA	NA	FALSE
GB17921	14	5	1.485426827	0.620909319	TRUE
GB17922	0	1	NA	NA	FALSE
GB17923	15	10	0.584962501	-0.279555008	FALSE
GB17924	1396	893	0.644566861	-0.219950647	FALSE
GB17925	2	0	NA	NA	FALSE
GB17926	149	71	1.069421401	0.204903893	FALSE
GB17927	NA	NA	NA	NA	FALSE
GB17928	326	147	1.149055809	0.284538301	FALSE
GB17929	1614	1037	0.638224684	-0.226292824	FALSE
GB17930	906	466	0.959181095	0.094663587	FALSE
GB17931	3033	1510	1.006196948	0.14167944	FALSE
GB17932	414	217	0.931935725	0.067418217	FALSE
GB17933	44	20	1.137503524	0.272986015	FALSE
GB17934	517	249	1.054018538	0.18950103	FALSE
GB17935	520	292	0.832543254	-0.031974254	FALSE
GB17936	335	154	1.121230745	0.256713236	FALSE
GB17937	1068	524	1.02727293	0.162755422	FALSE
GB17938	238	57	2.061927749	1.197410241	TRUE
GB17939	1183	612	0.950846516	0.086329007	FALSE
GB17940	563	293	0.942234258	0.077716749	FALSE
GB17941	27	13	1.054447784	0.189930276	FALSE
GB17942	533	307	0.795896877	-0.068620631	FALSE
GB17943	664	509	0.383517585	-0.480999923	FALSE
GB17944	168	63	1.415037499	0.550519991	TRUE
GB17945	1478	849	0.799809811	-0.064707698	FALSE
GB17946	260	144	0.852442812	-0.012074697	FALSE
GB17947	1556	724	1.103780458	0.23926295	FALSE
GB17948	1487	765	0.958872994	0.094355486	FALSE
GB17949	125	75	0.736965594	-0.127551914	FALSE
GB17950	8877	5965	0.573550059	-0.290967449	FALSE
GB17951	900	630	0.514573173	-0.349944335	FALSE
GB17952	141	92	0.615989396	-0.248528112	FALSE
GB17953	6	13	-1.115477217	-1.979994726	TRUE
GB17954	1893	1087	0.800322471	-0.064195038	FALSE
GB17955	4308	2530	0.767880865	-0.096636643	FALSE
GB17956	502	259	0.954735266	0.090217758	FALSE
GB17957	453	261	0.795461243	-0.069056265	FALSE
GB17958	128	71	0.85025288	-0.014264628	FALSE
GB17959	6783	4141	0.71194429	-0.152573218	FALSE
GB17960	1067	620	0.783220056	-0.081297453	FALSE

GB17961	1493	735	1.02239801	0.157880502	FALSE
GB17962	488	279	0.806616026	-0.057901483	FALSE
GB17963	481	250	0.944108799	0.079591291	FALSE
GB17964	2	0	NA	NA	FALSE
GB17965	744	443	0.747995923	-0.116521586	FALSE
GB17966	104	67	0.634350528	-0.230166981	FALSE
GB17967	587	387	0.601026937	-0.263490571	FALSE
GB17968	247	136	0.86090439	-0.003613118	FALSE
GB17969	100	31	1.689659879	0.825142371	TRUE
GB17970	NA	NA	NA	NA	FALSE
GB17971	805	402	1.001793282	0.137275773	FALSE
GB17972	944	478	0.981776241	0.117258733	FALSE
GB17973	160	99	0.692571475	-0.171946034	FALSE
GB17974	301	142	1.083872557	0.219355049	FALSE
GB17975	170	38	2.161463423	1.296945914	TRUE
GB17976	134	83	0.691049759	-0.173467749	FALSE
GB17977	485	298	0.702672417	-0.161845092	FALSE
GB17978	48	24	1	0.135482492	FALSE
GB17979	112	69	0.698830465	-0.165687043	FALSE
GB17980	88	44	1	0.135482492	FALSE
GB17981	NA	NA	NA	NA	FALSE
GB17982	593	291	1.027012952	0.162495443	FALSE
GB17983	97	76	0.351985329	-0.51253218	TRUE
GB17984	1	1	0	-0.864517508	TRUE
GB17985	56	13	2.106915204	1.242397696	TRUE
GB17986	931	555	0.746293396	-0.118224112	FALSE
GB17987	7820	3467	1.173480771	0.308963263	FALSE
GB17988	26	11	1.2410081	0.376490591	FALSE
GB17989	1471	594	1.30826241	0.443744902	FALSE
GB17990	8	2	2	1.135482492	TRUE
GB17991	56	29	0.949373927	0.084856419	FALSE
GB17992	23	8	1.523561956	0.659044448	TRUE
GB17993	1	0	NA	NA	FALSE
GB17994	480	280	0.777607579	-0.08690993	FALSE
GB17995	901	508	0.826698609	-0.037818899	FALSE
GB17996	759	471	0.688372826	-0.176144683	FALSE
GB17997	19639	12624	0.63755236	-0.226965148	FALSE
GB17998	321	203	0.66109357	-0.203423938	FALSE
GB17999	287	159	0.852023971	-0.012493537	FALSE
GB18000	2506	1637	0.614332093	-0.250185415	FALSE
GB18001	3928	2018	0.960868755	0.096351247	FALSE
GB18002	NA	NA	NA	NA	FALSE
GB18003	1556	1170	0.411333531	-0.453183978	FALSE
GB18004	126830	57660	1.137253295	0.272735786	FALSE

GB18005	348	180	0.9510904	0.086572891	FALSE
GB18006	424	251	0.756376901	-0.108140608	FALSE
GB18007	100	42	1.251538767	0.387021259	FALSE
GB18008	439	243	0.853264626	-0.011252882	FALSE
GB18009	153	104	0.556948125	-0.307569384	FALSE
GB18010	48	14	1.777607579	0.91309007	TRUE
GB18011	1276	682	0.903784685	0.039267176	FALSE
GB18012	642	324	0.986579484	0.122061976	FALSE
GB18013	127369	43010	1.56627015	0.701752642	TRUE
GB18014	283	132	1.100264123	0.235746615	FALSE
GB18015	212	117	0.857555735	-0.006961773	FALSE
GB18016	349	203	0.781747309	-0.082770199	FALSE
GB18017	937	508	0.883220551	0.018703043	FALSE
GB18018	226	182	0.312384322	-0.552133186	TRUE
GB18019	160	66	1.277533976	0.413016467	FALSE
GB18020	1616	964	0.745322147	-0.119195362	FALSE
GB18021	NA	NA	NA	NA	FALSE
GB18022	31	16	0.95419631	0.089678802	FALSE
GB18023	304	204	0.575502171	-0.289015337	FALSE
GB18024	50	44	0.184424571	-0.680092937	TRUE
GB18025	1047	613	0.772302463	-0.092215045	FALSE
GB18026	2616	1304	1.004418671	0.139901163	FALSE
GB18027	380	194	0.969942766	0.105425258	FALSE
GB18028	3604	2130	0.758745581	-0.105771928	FALSE
GB18029	111	79	0.490635118	-0.37388239	FALSE
GB18030	24503	15146	0.694021561	-0.170495947	FALSE
GB18031	2511	1309	0.939796931	0.075279423	FALSE
GB18032	1	6	-2.584962501	-3.449480009	TRUE
GB18033	1	0	NA	NA	FALSE
GB18034	NA	NA	NA	NA	FALSE
GB18035	406	185	1.133954457	0.269436948	FALSE
GB18036	5325	2913	0.870275824	0.005758316	FALSE
GB18037	267	142	0.910948812	0.046431304	FALSE
GB18038	NA	NA	NA	NA	FALSE
GB18039	703	410	0.77790078	-0.086616729	FALSE
GB18040	696	332	1.067904065	0.203386556	FALSE
GB18041	340	178	0.933657505	0.069139997	FALSE
GB18042	7426	4893	0.601866032	-0.262651476	FALSE
GB18043	760	430	0.821662759	-0.04285475	FALSE
GB18044	1105	776	0.509917812	-0.354599696	FALSE
GB18045	17697	7616	1.216399431	0.351881923	FALSE
GB18046	1061	588	0.851536596	-0.012980912	FALSE
GB18047	7	5	0.485426827	-0.379090681	FALSE
GB18048	NA	NA	NA	NA	FALSE

GB18049	285	114	1.321928095	0.457410587	FALSE
GB18050	145	100	0.5360529	-0.328464608	FALSE
GB18051	146	92	0.666262603	-0.198254906	FALSE
GB18052	2100	1112	0.91723254	0.052715032	FALSE
GB18053	2491	1245	1.000579279	0.136061771	FALSE
GB18054	432	210	1.040641984	0.176124476	FALSE
GB18055	7	3	1.222392421	0.357874913	FALSE
GB18056	3233	2090	0.629370565	-0.235146943	FALSE
GB18057	356	193	0.883276394	0.018758885	FALSE
GB18058	7	1	2.807354922	1.942837414	TRUE
GB18059	2220	1239	0.841383489	-0.023134019	FALSE
GB18060	32	32	0	-0.864517508	TRUE
GB18061	NA	NA	NA	NA	FALSE
GB18062	1096	697	0.653017237	-0.211500271	FALSE
GB18063	NA	NA	NA	NA	FALSE
GB18064	36	17	1.08246216	0.217944652	FALSE
GB18065	309	149	1.052294507	0.187776999	FALSE
GB18066	NA	NA	NA	NA	FALSE
GB18067	6	11	-0.874469118	-1.738986626	TRUE
GB18068	1261	712	0.824619129	-0.039898379	FALSE
GB18069	278	116	1.260960078	0.396442569	FALSE
GB18070	5	6	-0.263034406	-1.127551914	TRUE
GB18071	1347	985	0.451554221	-0.412963287	FALSE
GB18072	20	18	0.152003093	-0.712514415	TRUE
GB18073	584	304	0.941897045	0.077379537	FALSE
GB18074	0	1	NA	NA	FALSE
GB18075	42	23	0.868755467	0.004237958	FALSE
GB18076	12030	6367	0.917950973	0.053433465	FALSE
GB18077	2142	786	1.446357262	0.581839754	TRUE
GB18078	2151	1289	0.738755261	-0.125762247	FALSE
GB18079	2878	1265	1.185929207	0.321411699	FALSE
GB18080	724	344	1.073581132	0.209063624	FALSE
GB18081	430	227	0.921644362	0.057126854	FALSE
GB18082	1598	598	1.418050019	0.55353251	TRUE
GB18083	52	32	0.700439718	-0.16407779	FALSE
GB18084	NA	NA	NA	NA	FALSE
GB18085	5	2	1.321928095	0.457410587	FALSE
GB18086	6898	4028	0.776114445	-0.088403063	FALSE
GB18087	3458	2436	0.505423736	-0.359093773	FALSE
GB18088	878	426	1.043367509	0.178850001	FALSE
GB18089	115	56	1.038135129	0.173617621	FALSE
GB18090	207	119	0.798669194	-0.065848314	FALSE
GB18091	249	155	0.683877527	-0.180639982	FALSE
GB18092	NA	NA	NA	NA	FALSE

GB18093	874	455	0.941766734	0.077249226	FALSE
GB18094	4	1	2	1.135482492	TRUE
GB18095	7	4	0.807354922	-0.057162586	FALSE
GB18096	399	109	1.872060611	1.007543103	TRUE
GB18097	575	321	0.840988659	-0.02352885	FALSE
GB18098	841	470	0.839445044	-0.025072465	FALSE
GB18099	1384	709	0.96498641	0.100468902	FALSE
GB18100	99	64	0.62935662	-0.235160888	FALSE
GB18101	449	317	0.502232605	-0.362284904	FALSE
GB18102	133	88	0.595850817	-0.268666691	FALSE
GB18103	0	1	NA	NA	FALSE
GB18104	232	144	0.688055994	-0.176461515	FALSE
GB18105	1899	792	1.26166757	0.397150062	FALSE
GB18106	8678	4088	1.08596739	0.221449882	FALSE
GB18107	1501	816	0.87928292	0.014765411	FALSE
GB18108	14467	10255	0.496438292	-0.368079217	FALSE
GB18109	26696	18245	0.549122441	-0.315395068	FALSE
GB18110	204	98	1.057715498	0.19319799	FALSE
GB18111	261	109	1.259721672	0.395204163	FALSE
GB18112	5322	2780	0.936883627	0.072366119	FALSE
GB18113	632	477	0.405935292	-0.458582216	FALSE
GB18114	620	323	0.940734051	0.076216542	FALSE
GB18115	118	60	0.975752454	0.111234945	FALSE
GB18116	1501	863	0.798491512	-0.066025996	FALSE
GB18117	512	311	0.71922923	-0.145288278	FALSE
GB18118	535	260	1.041027268	0.17650976	FALSE
GB18119	30	11	1.447458977	0.582941469	TRUE
GB18120	129	62	1.057030945	0.192513437	FALSE
GB18121	3823	2393	0.675884803	-0.188632705	FALSE
GB18122	1263	596	1.083470403	0.218952895	FALSE
GB18123	1316	561	1.230086813	0.365569305	FALSE
GB18124	3243	2476	0.389317709	-0.475199799	FALSE
GB18125	303	157	0.948553235	0.084035726	FALSE
GB18126	406	291	0.480460574	-0.384056934	FALSE
GB18127	422	186	1.181940378	0.317422869	FALSE
GB18128	8	5	0.678071905	-0.186445603	FALSE
GB18129	376	192	0.969626351	0.105108843	FALSE
GB18130	761	348	1.128809148	0.264291639	FALSE
GB18131	4	7	-0.807354922	-1.67187243	TRUE
GB18132	1249	677	0.883545738	0.01902823	FALSE
GB18133	202	140	0.528928466	-0.335589043	FALSE
GB18134	NA	NA	NA	NA	FALSE
GB18135	474	229	1.049539461	0.185021952	FALSE
GB18136	279	150	0.895302621	0.030785113	FALSE

GB18137	2536	1254	1.016017397	0.151499889	FALSE
GB18138	NA	NA	NA	NA	FALSE
GB18139	1056	706	0.580869746	-0.283647762	FALSE
GB18140	1407	765	0.879090676	0.014573167	FALSE
GB18141	347	199	0.802167232	-0.062350276	FALSE
GB18142	484	260	0.896495424	0.031977916	FALSE
GB18143	404	274	0.5601794	-0.304338109	FALSE
GB18144	1924	1074	0.841114806	-0.023402703	FALSE
GB18145	2567	1409	0.865411684	0.000894176	FALSE
GB18146	216	130	0.732519689	-0.131997819	FALSE
GB18147	339	168	1.01282404	0.148306532	FALSE
GB18148	160	247	-0.626439137	-1.490956645	TRUE
GB18149	311	193	0.688313733	-0.176203775	FALSE
GB18150	42751	17521	1.286873049	0.42235554	FALSE
GB18151	19	5	1.925999419	1.06148191	TRUE
GB18152	2604	1825	0.512832985	-0.351684524	FALSE
GB18153	1322	675	0.96976277	0.105245261	FALSE
GB18154	4	4	0	-0.864517508	TRUE
GB18155	11	4	1.459431619	0.59491411	TRUE
GB18156	659	502	0.392591101	-0.471926407	FALSE
GB18157	875	399	1.13289427	0.268376762	FALSE
GB18158	58	40	0.5360529	-0.328464608	FALSE
GB18159	322	137	1.232884795	0.368367287	FALSE
GB18160	2	5	-1.321928095	-2.186445603	TRUE
GB18161	2	0	NA	NA	FALSE
GB18162	11900	5411	1.136994427	0.272476919	FALSE
GB18163	91	72	0.337869639	-0.52664787	TRUE
GB18164	1	0	NA	NA	FALSE
GB18165	263	109	1.270734665	0.406217156	FALSE
GB18166	NA	NA	NA	NA	FALSE
GB18167	306	226	0.43720888	-0.427308628	FALSE
GB18168	457	250	0.87026607	0.005748562	FALSE
GB18169	287	167	0.781202634	-0.083314874	FALSE
GB18170	4255	2360	0.850372272	-0.014145236	FALSE
GB18171	750	419	0.839940352	-0.024577157	FALSE
GB18172	683	562	0.281315448	-0.58320206	TRUE
GB18173	3005	1923	0.644006228	-0.22051128	FALSE
GB18174	82	29	1.499571009	0.635053501	TRUE
GB18175	784	386	1.022252807	0.157735299	FALSE
GB18176	1261	672	0.908035138	0.043517629	FALSE
GB18177	92	46	1	0.135482492	FALSE
GB18178	54	17	1.667424661	0.802907153	TRUE
GB18179	NA	NA	NA	NA	FALSE
GB18180	711	434	0.712154517	-0.152362991	FALSE

GB18181	16	6	1.415037499	0.550519991	TRUE
GB18182	515	355	0.536753408	-0.327764101	FALSE
GB18183	1899	1328	0.515984759	-0.34853275	FALSE
GB18184	969	486	0.995540352	0.131022843	FALSE
GB18185	700	283	1.306552869	0.442035361	FALSE
GB18186	473	227	1.059147886	0.194630378	FALSE
GB18187	2257	1254	0.84786907	-0.016648438	FALSE
GB18188	204	139	0.553484269	-0.311033239	FALSE
GB18189	1278	587	1.122455428	0.25793792	FALSE
GB18190	NA	NA	NA	NA	FALSE
GB18191	34152	21719	0.653012389	-0.21150512	FALSE
GB18192	5813	3919	0.568797336	-0.295720173	FALSE
GB18193	53	20	1.40599236	0.541474851	TRUE
GB18194	942	585	0.687290435	-0.177227073	FALSE
GB18195	437	262	0.738066468	-0.12645104	FALSE
GB18196	957	514	0.896750565	0.032233057	FALSE
GB18197	97	37	1.390459477	0.525941968	TRUE
GB18198	4216	2539	0.731614472	-0.132903036	FALSE
GB18199	257	190	0.435768941	-0.428748567	FALSE
GB18200	84	32	1.392317423	0.527799914	TRUE
GB18201	1128	338	1.738671916	0.874154408	TRUE
GB18202	1560	976	0.676592976	-0.187924532	FALSE
GB18203	331	235	0.49417046	-0.370347048	FALSE
GB18204	1079	544	0.988016308	0.1234988	FALSE
GB18205	3157	1062	1.571770495	0.707252986	TRUE
GB18206	198	94	1.074767768	0.21025026	FALSE
GB18207	941	395	1.25234207	0.387824561	FALSE
GB18208	259	166	0.641768856	-0.222748652	FALSE
GB18209	407	313	0.378866137	-0.485651371	FALSE
GB18210	171	42	2.025535092	1.161017584	TRUE
GB18211	1031	801	0.364170185	-0.500347323	TRUE
GB18212	77	47	0.712197689	-0.152319819	FALSE
GB18213	689	409	0.75240314	-0.112114369	FALSE
GB18214	2149	1303	0.721828399	-0.142689109	FALSE
GB18215	460	227	1.018941564	0.154424055	FALSE
GB18216	387	222	0.80177389	-0.062743619	FALSE
GB18217	1635	1089	0.586286682	-0.278230827	FALSE
GB18218	17	11	0.628031223	-0.236486286	FALSE
GB18219	2711	1247	1.120363648	0.25584614	FALSE
GB18220	193	85	1.183066101	0.318548593	FALSE
GB18221	914	550	0.732762547	-0.131754962	FALSE
GB18222	2021	1325	0.609076962	-0.255440546	FALSE
GB18223	170	78	1.123988717	0.259471209	FALSE
GB18224	50	10	2.321928095	1.457410587	TRUE

GB18225	243	116	1.066831508	0.202314	FALSE
GB18226	2297	1465	0.648850192	-0.215667316	FALSE
GB18227	203	106	0.937415463	0.072897954	FALSE
GB18228	27	13	1.054447784	0.189930276	FALSE
GB18229	197	83	1.247012388	0.38249488	FALSE
GB18230	235	98	1.261807102	0.397289594	FALSE
GB18231	2	0	NA	NA	FALSE
GB18232	1533	867	0.822253798	-0.04226371	FALSE
GB18233	6737	4756	0.502357584	-0.362159924	FALSE
GB18234	566	323	0.809267888	-0.05524962	FALSE
GB18235	416	212	0.972519264	0.108001755	FALSE
GB18236	706	410	0.784044274	-0.080473235	FALSE
GB18237	193	112	0.785102115	-0.079415393	FALSE
GB18238	193	145	0.412547947	-0.451969561	FALSE
GB18239	674	426	0.661895161	-0.202622347	FALSE
GB18240	78	14	2.478047297	1.613529788	TRUE
GB18241	32	28	0.192645078	-0.67187243	TRUE
GB18242	1554	795	0.966959738	0.10244223	FALSE
GB18243	169	88	0.941447818	0.076930309	FALSE
GB18244	87	59	0.560300446	-0.304217062	FALSE
GB18245	2653	1470	0.851808521	-0.012708988	FALSE
GB18246	707	469	0.592122292	-0.272395216	FALSE
GB18247	171	124	0.463656204	-0.400861304	FALSE
GB18248	342	155	1.14172811	0.277210601	FALSE
GB18249	5105	2890	0.820841468	-0.04367604	FALSE
GB18250	672	455	0.562594688	-0.301922821	FALSE
GB18251	4338	2732	0.667072569	-0.197444939	FALSE
GB18252	403	139	1.535694956	0.671177447	TRUE
GB18253	NA	NA	NA	NA	FALSE
GB18254	658	203	1.696607857	0.832090348	TRUE
GB18255	1382	683	1.016800132	0.152282624	FALSE
GB18256	1619	832	0.960447552	0.095930044	FALSE
GB18257	0	1	NA	NA	FALSE
GB18258	1	1	0	-0.864517508	TRUE
GB18259	772	446	0.791557137	-0.072960371	FALSE
GB18260	23	18	0.353636955	-0.510880554	TRUE
GB18261	1586	1390	0.190307888	-0.67420962	TRUE
GB18262	492	239	1.041647697	0.177130189	FALSE
GB18263	4166	2087	0.997232239	0.132714731	FALSE
GB18264	423	338	0.323634417	-0.540883091	TRUE
GB18265	620	314	0.981503656	0.116986148	FALSE
GB18266	6	1	2.584962501	1.720444992	TRUE
GB18267	961	484	0.989529383	0.125011875	FALSE
GB18268	2457	998	1.299786137	0.435268629	FALSE

GB18269	296	232	0.351472371	-0.513045138	TRUE
GB18270	252	129	0.966052668	0.10153516	FALSE
GB18271	5	1	2.321928095	1.457410587	TRUE
GB18272	2708	1396	0.955928797	0.091411289	FALSE
GB18273	901	517	0.801362825	-0.063154683	FALSE
GB18274	NA	NA	NA	NA	FALSE
GB18275	341	227	0.587079442	-0.277438067	FALSE
GB18276	809	485	0.738154955	-0.126362553	FALSE
GB18277	528	364	0.536599479	-0.327918029	FALSE
GB18278	76	38	1	0.135482492	FALSE
GB18279	2217	976	1.183655717	0.319138209	FALSE
GB18280	17	3	2.502500341	1.637982832	TRUE
GB18281	1621	968	0.743805138	-0.12071237	FALSE
GB18282	3166	1498	1.079623632	0.215106123	FALSE
GB18283	548	322	0.767115205	-0.097402303	FALSE
GB18284	2397	1455	0.720210756	-0.144306752	FALSE
GB18285	408	268	0.606336152	-0.258181357	FALSE
GB18286	65	36	0.852442812	-0.012074697	FALSE
GB18287	3098	1914	0.694746314	-0.169771194	FALSE
GB18288	466	214	1.122719158	0.25820165	FALSE
GB18289	236	137	0.784610966	-0.079906542	FALSE
GB18290	41	40	0.03562391	-0.828893599	TRUE
GB18291	108	60	0.847996907	-0.016520602	FALSE
GB18292	3500	2253	0.635507608	-0.2290099	FALSE
GB18293	430	218	0.980008525	0.115491016	FALSE
GB18294	1050	489	1.102482958	0.237965449	FALSE
GB18295	1866	1161	0.684581014	-0.179936494	FALSE
GB18296	85	51	0.736965594	-0.127551914	FALSE
GB18297	21	4	2.392317423	1.527799914	TRUE
GB18298	NA	NA	NA	NA	FALSE
GB18299	415	173	1.262339299	0.39782179	FALSE
GB18300	392	106	1.88678939	1.022271881	TRUE
GB18301	578	191	1.597496854	0.732979346	TRUE
GB18302	448	222	1.012939056	0.148421547	FALSE
GB18303	7	5	0.485426827	-0.379090681	FALSE
GB18304	30	20	0.584962501	-0.279555008	FALSE
GB18305	184	113	0.703382994	-0.161134515	FALSE
GB18306	262	144	0.863498	-0.001019508	FALSE
GB18307	842	431	0.966132364	0.101614856	FALSE
GB18308	631	273	1.208739054	0.344221546	FALSE
GB18309	653	334	0.967234889	0.102717381	FALSE
GB18310	226	148	0.610725597	-0.253791912	FALSE
GB18311	78	30	1.378511623	0.513994115	TRUE
GB18312	300	62	2.27462238	1.410104872	TRUE

GB18313	2061	1024	1.00912879	0.144611281	FALSE
GB18314	1754	935	0.907610478	0.043092969	FALSE
GB18315	807	479	0.752543018	-0.111974491	FALSE
GB18316	NA	NA	NA	NA	FALSE
GB18317	401	239	0.746591618	-0.11792589	FALSE
GB18318	227	93	1.287389676	0.422872168	FALSE
GB18319	211	78	1.43569697	0.571179462	TRUE
GB18320	937	443	1.080742349	0.216224841	FALSE
GB18321	688	460	0.580774704	-0.283742805	FALSE
GB18322	245	94	1.382049087	0.517531579	TRUE
GB18323	643	116	2.470693932	1.606176424	TRUE
GB18324	1019	492	1.050423831	0.185906322	FALSE
GB18325	382	182	1.069634188	0.20511668	FALSE
GB18326	685	439	0.641883048	-0.22263446	FALSE
GB18327	553	251	1.139592116	0.275074608	FALSE
GB18328	4	5	-0.321928095	-1.186445603	TRUE
GB18329	1	0	NA	NA	FALSE
GB18330	190	94	1.015266757	0.150749248	FALSE
GB18331	306	140	1.128104826	0.263587317	FALSE
GB18332	17496	10900	0.68269699	-0.181820518	FALSE
GB18333	1106	717	0.625306362	-0.239211147	FALSE
GB18334	30	173	-2.527737632	-3.39225514	TRUE
GB18335	5468	2924	0.903069932	0.038552423	FALSE
GB18336	251	179	0.487727777	-0.376789732	FALSE
GB18337	2627	1400	0.907989373	0.043471865	FALSE
GB18338	2650	1370	0.951816466	0.087298958	FALSE
GB18339	57	46	0.309328058	-0.55518945	TRUE
GB18340	102	104	-0.028014376	-0.892531884	TRUE
GB18341	961	515	0.899963999	0.03544649	FALSE
GB18342	361	159	1.182972072	0.318454563	FALSE
GB18343	NA	NA	NA	NA	FALSE
GB18344	1147	496	1.209453366	0.344935857	FALSE
GB18345	NA	NA	NA	NA	FALSE
GB18346	809	355	1.188320678	0.32380317	FALSE
GB18347	1317	627	1.070717997	0.206200489	FALSE
GB18348	717	391	0.874804511	0.010287003	FALSE
GB18349	78	60	0.378511623	-0.486005885	FALSE
GB18350	318	150	1.084064265	0.219546756	FALSE
GB18351	1910	862	1.147812864	0.283295356	FALSE
GB18352	437	257	0.76586492	-0.098652588	FALSE
GB18353	494	255	0.954013795	0.089496286	FALSE
GB18354	4032	2263	0.833259054	-0.031258454	FALSE
GB18355	483	239	1.015012571	0.150495063	FALSE
GB18356	728	374	0.96090018	0.096382672	FALSE

GB18357	4948	3149	0.651951743	-0.212565765	FALSE
GB18358	547	292	0.905572464	0.041054956	FALSE
GB18359	0	1	NA	NA	FALSE
GB18360	1075	382	1.492692116	0.628174608	TRUE
GB18361	10	2	2.321928095	1.457410587	TRUE
GB18362	432	251	0.783343948	-0.08117356	FALSE
GB18363	10534	4530	1.217470409	0.352952901	FALSE
GB18364	86	36	1.256339753	0.391822245	FALSE
GB18365	2693	1320	1.0286763	0.164158792	FALSE
GB18366	898	514	0.804947086	-0.059570423	FALSE
GB18367	1	4	-2	-2.864517508	TRUE
GB18368	195	129	0.596103058	-0.26841445	FALSE
GB18369	3	5	-0.736965594	-1.601483102	TRUE
GB18370	141	78	0.854149134	-0.010368375	FALSE
GB18371	817	501	0.705525475	-0.158992033	FALSE
GB18372	1054	821	0.36042074	-0.504096768	TRUE
GB18373	464	353	0.394456622	-0.470060886	FALSE
GB18374	151	110	0.457045026	-0.407472483	FALSE
GB18375	127	88	0.529253068	-0.33526444	FALSE
GB18376	25	11	1.184424571	0.319907063	FALSE
GB18377	NA	NA	NA	NA	FALSE
GB18378	1284	699	0.877280842	0.012763333	FALSE
GB18379	339	166	1.030102032	0.165584523	FALSE
GB18380	700	328	1.093659107	0.229141599	FALSE
GB18381	1935	1200	0.689299161	-0.175218348	FALSE
GB18382	253	133	0.927711139	0.063193631	FALSE
GB18383	392	192	1.029747343	0.165229835	FALSE
GB18384	311	121	1.361907533	0.497390025	FALSE
GB18385	710	343	1.049610448	0.18509294	FALSE
GB18386	1264	673	0.909318054	0.044800545	FALSE
GB18387	900	586	0.619024337	-0.245493172	FALSE
GB18388	1102	667	0.724365557	-0.140151951	FALSE
GB18389	164	88	0.898120386	0.033602878	FALSE
GB18390	1362	1046	0.380843852	-0.483673657	FALSE
GB18391	1	0	NA	NA	FALSE
GB18392	493	273	0.852686695	-0.011830813	FALSE
GB18393	55	31	0.827163403	-0.037354105	FALSE
GB18394	2432	1185	1.03725617	0.172738661	FALSE
GB18395	1409	920	0.614965845	-0.249551663	FALSE
GB18396	16	5	1.678071905	0.813554397	TRUE
GB18397	NA	NA	NA	NA	FALSE
GB18398	283	132	1.100264123	0.235746615	FALSE
GB18399	14	7	1	0.135482492	FALSE
GB18400	2	0	NA	NA	FALSE

GB18401	59	20	1.560714954	0.696197446	TRUE
GB18402	2100	1268	0.727834582	-0.136682926	FALSE
GB18403	12	4	1.584962501	0.720444992	TRUE
GB18404	4484	2857	0.650285242	-0.214232266	FALSE
GB18405	402	231	0.79930265	-0.065214859	FALSE
GB18406	476	294	0.695145418	-0.16937209	FALSE
GB18407	1866	1085	0.782253944	-0.082263565	FALSE
GB18408	787	394	0.998168006	0.133650498	FALSE
GB18409	18	13	0.469485283	-0.395032225	FALSE
GB18410	616	327	0.913639715	0.049122207	FALSE
GB18411	3	2	0.584962501	-0.279555008	FALSE
GB18412	411	227	0.856446096	-0.008071412	FALSE
GB18413	179	79	1.180035029	0.315517521	FALSE
GB18414	372	135	1.462343214	0.597825706	TRUE
GB18415	6145	4061	0.597577983	-0.266939525	FALSE
GB18416	640	469	0.448483982	-0.416033526	FALSE
GB18417	6375	4092	0.639619197	-0.224898311	FALSE
GB18418	205	137	0.581448017	-0.283069492	FALSE
GB18419	1071	521	1.039603202	0.175085694	FALSE
GB18420	374	202	0.888682977	0.024165469	FALSE
GB18421	NA	NA	NA	NA	FALSE
GB18422	30	8	1.906890596	1.042373087	TRUE
GB18423	152	83	0.872888082	0.008370574	FALSE
GB18424	262	152	0.785495488	-0.07902202	FALSE
GB18425	242	179	0.43504746	-0.429470048	FALSE
GB18426	68	62	0.133266531	-0.731250977	TRUE
GB18427	158	79	1	0.135482492	FALSE
GB18428	87	57	0.610053482	-0.254464027	FALSE
GB18429	809	462	0.808246851	-0.056270657	FALSE
GB18430	688	392	0.811554911	-0.052962598	FALSE
GB18431	485	276	0.81331648	-0.051201028	FALSE
GB18432	39	22	0.8259706	-0.038546908	FALSE
GB18433	461	247	0.900255709	0.035738201	FALSE
GB18434	941	546	0.785293772	-0.079223737	FALSE
GB18435	453	180	1.331514144	0.466996635	FALSE
GB18436	3	4	-0.415037499	-1.279555008	TRUE
GB18437	117	75	0.641546029	-0.222971479	FALSE
GB18438	NA	NA	NA	NA	FALSE
GB18439	8167	4220	0.952563229	0.08804572	FALSE
GB18440	4450	2531	0.814097829	-0.05041968	FALSE
GB18441	149	106	0.491248066	-0.373269442	FALSE
GB18442	292	188	0.635235707	-0.229281801	FALSE
GB18443	585	274	1.094260732	0.229743223	FALSE
GB18444	342	289	0.242926832	-0.621590676	TRUE

GB18445	1	0	NA	NA	FALSE
GB18446	34408	21852	0.654978708	-0.2095388	FALSE
GB18447	539	268	1.008052272	0.143534764	FALSE
GB18448	1583	907	0.8034868	-0.061030709	FALSE
GB18449	1	1	0	-0.864517508	TRUE
GB18450	39	18	1.115477217	0.250959709	FALSE
GB18451	74	21	1.817135943	0.952618435	TRUE
GB18452	1023	695	0.557721262	-0.306796246	FALSE
GB18453	3	1	1.584962501	0.720444992	TRUE
GB18454	16518	7373	1.163715354	0.299197845	FALSE
GB18455	494	260	0.925999419	0.06148191	FALSE
GB18456	91	71	0.358047521	-0.506469988	TRUE
GB18457	4	3	0.415037499	-0.449480009	FALSE
GB18458	801	504	0.668378509	-0.196138999	FALSE
GB18459	11363	5891	0.94775932	0.083241812	FALSE
GB18460	479	300	0.675063155	-0.189454353	FALSE
GB18461	438	246	0.832272554	-0.032244954	FALSE
GB18462	12	12	0	-0.864517508	TRUE
GB18463	8280	4904	0.755671789	-0.10884572	FALSE
GB18464	9	2	2.169925001	1.305407493	TRUE
GB18465	721	374	0.946960989	0.082443481	FALSE
GB18466	NA	NA	NA	NA	FALSE
GB18467	640	378	0.759685671	-0.104831838	FALSE
GB18468	157	98	0.679910905	-0.184606604	FALSE
GB18469	NA	NA	NA	NA	FALSE
GB18470	3131	1511	1.051119848	0.18660234	FALSE
GB18471	749	306	1.291434066	0.426916557	FALSE
GB18472	3	4	-0.415037499	-1.279555008	TRUE
GB18473	15	12	0.321928095	-0.542589413	TRUE
GB18474	128	114	0.167109986	-0.697407522	TRUE
GB18475	213	86	1.308444866	0.443927357	FALSE
GB18476	1909	1260	0.599393369	-0.265124139	FALSE
GB18477	1137	697	0.706001693	-0.158515815	FALSE
GB18478	79	35	1.174497731	0.309980223	FALSE
GB18479	1800	725	1.311944006	0.447426498	FALSE
GB18480	170	82	1.051838932	0.187321423	FALSE
GB18481	460	266	0.790207615	-0.074309893	FALSE
GB18482	571	358	0.673531158	-0.19098635	FALSE
GB18483	454	222	1.032132621	0.167615113	FALSE
GB18484	230	56	2.038135129	1.173617621	TRUE
GB18485	1528	563	1.440437716	0.575920208	TRUE
GB18486	784	429	0.869876007	0.005358498	FALSE
GB18487	278	124	1.164744762	0.300227254	FALSE
GB18488	449	186	1.271412824	0.406895315	FALSE

GB18489	96	54	0.830074999	-0.03444251	FALSE
GB18490	1122	862	0.380312902	-0.484204607	FALSE
GB18491	812	522	0.637429921	-0.227087588	FALSE
GB18492	269	160	0.749534268	-0.114983241	FALSE
GB18493	941	470	1.001533966	0.137016458	FALSE
GB18494	3585	1519	1.238851249	0.374333741	FALSE
GB18495	344	236	0.543621705	-0.320895803	FALSE
GB18496	1450	897	0.69287301	-0.171644498	FALSE
GB18497	10465	6931	0.594436888	-0.27008062	FALSE
GB18498	22956	14993	0.614582194	-0.249935315	FALSE
GB18499	436	228	0.935294311	0.070776802	FALSE
GB18500	5	5	0	-0.864517508	TRUE
GB18501	5688	4177	0.445454321	-0.419063187	FALSE
GB18502	312	164	0.927850214	0.063332706	FALSE
GB18503	890	425	1.066342495	0.201824987	FALSE
GB18504	764	443	0.786265939	-0.078251569	FALSE
GB18505	327	153	1.095758983	0.231241474	FALSE
GB18506	677	391	0.791987226	-0.072530282	FALSE
GB18507	949	455	1.060541542	0.196024034	FALSE
GB18508	1342	810	0.728390858	-0.13612665	FALSE
GB18509	62	44	0.494764692	-0.369752817	FALSE
GB18510	348	201	0.791891805	-0.072625704	FALSE
GB18511	294	156	0.914270126	0.049752618	FALSE
GB18512	19562	8370	1.22475435	0.360236841	FALSE
GB18513	356	161	1.144816553	0.280299045	FALSE
GB18514	179	128	0.483815777	-0.380701731	FALSE
GB18515	395	254	0.637024156	-0.227493352	FALSE
GB18516	656	436	0.58936768	-0.275149828	FALSE
GB18517	1296	789	0.715968513	-0.148548995	FALSE
GB18518	53	27	0.973032952	0.108515444	FALSE
GB18519	80	37	1.112474729	0.247957221	FALSE
GB18520	1109	868	0.353492418	-0.511025091	TRUE
GB18521	97	21	2.207595419	1.343077911	TRUE
GB18522	560	279	1.005161705	0.140644197	FALSE
GB18523	171	103	0.731351988	-0.133165521	FALSE
GB18524	51	9	2.502500341	1.637982832	TRUE
GB18525	673	362	0.894616808	0.030099299	FALSE
GB18526	645	321	1.006725863	0.142208355	FALSE
GB18527	3975	1644	1.273744561	0.409227053	FALSE
GB18528	7	7	0	-0.864517508	TRUE
GB18529	3183	1695	0.909101884	0.044584375	FALSE
GB18530	729	154	2.242988464	1.378470955	TRUE
GB18531	142	87	0.706803624	-0.157713885	FALSE
GB18532	NA	NA	NA	NA	FALSE

GB18533	140	82	0.771731012	-0.092786496	FALSE
GB18534	2704	1760	0.619519723	-0.244997786	FALSE
GB18535	554	313	0.823723319	-0.040794189	FALSE
GB18536	2047	1039	0.978315448	0.11379794	FALSE
GB18537	248	150	0.72537762	-0.139139888	FALSE
GB18538	10	10	0	-0.864517508	TRUE
GB18539	211	147	0.521426844	-0.343090664	FALSE
GB18540	312	144	1.115477217	0.250959709	FALSE
GB18541	60	24	1.321928095	0.457410587	FALSE
GB18542	NA	NA	NA	NA	FALSE
GB18543	13	5	1.378511623	0.513994115	TRUE
GB18544	2501	1691	0.564628398	-0.299889111	FALSE
GB18545	2661	1755	0.60049748	-0.264020028	FALSE
GB18546	0	3	NA	NA	FALSE
GB18547	0	2	NA	NA	FALSE
GB18548	38	11	1.788495895	0.923978386	TRUE
GB18549	4057	2163	0.907379637	0.042862129	FALSE
GB18550	206	121	0.76763729	-0.096880218	FALSE
GB18551	NA	NA	NA	NA	FALSE
GB18552	234	144	0.700439718	-0.16407779	FALSE
GB18553	1438	860	0.741655111	-0.122862397	FALSE
GB18554	2125	1075	0.983126181	0.118608673	FALSE
GB18555	1212	679	0.835906219	-0.028611289	FALSE
GB18556	278	192	0.533978572	-0.330538936	FALSE
GB18557	14981	7296	1.037956294	0.173438786	FALSE
GB18558	2636	1934	0.446762576	-0.417754933	FALSE
GB18559	2536	1327	0.934386375	0.069868866	FALSE
GB18560	252	94	1.422691072	0.558173563	TRUE
GB18561	205	120	0.772589504	-0.091928004	FALSE
GB18562	53	12	2.142957954	1.278440446	TRUE
GB18563	823	481	0.774855537	-0.089661972	FALSE
GB18564	21656	13247	0.709101118	-0.15541639	FALSE
GB18565	10	2	2.321928095	1.457410587	TRUE
GB18566	23	13	0.823122238	-0.04139527	FALSE
GB18567	620	391	0.665099608	-0.1994179	FALSE
GB18568	9	1	3.169925001	2.305407493	TRUE
GB18569	1813	1020	0.829809773	-0.034707735	FALSE
GB18570	900	549	0.713118852	-0.151398656	FALSE
GB18571	171	89	0.942119084	0.077601576	FALSE
GB18572	1020	790	0.368644594	-0.495872915	FALSE
GB18573	424	221	0.940017895	0.075500387	FALSE
GB18574	281	199	0.4978017	-0.366715809	FALSE
GB18575	116	49	1.243271151	0.378753643	FALSE
GB18576	993	486	1.030837404	0.166319896	FALSE

GB18577	539	410	0.394661363	-0.469856145	FALSE
GB18578	7645	4354	0.812175094	-0.052342414	FALSE
GB18579	832	523	0.669772582	-0.194744926	FALSE
GB18580	6	4	0.584962501	-0.279555008	FALSE
GB18581	577	257	1.166802959	0.302285451	FALSE
GB18582	907	496	0.87076243	0.006244922	FALSE
GB18583	1054	613	0.781915888	-0.08260162	FALSE
GB18584	3	1	1.584962501	0.720444992	TRUE
GB18585	857	344	1.316886639	0.452369131	FALSE
GB18586	778	350	1.152415233	0.287897725	FALSE
GB18587	934	578	0.692353057	-0.172164451	FALSE
GB18588	159	94	0.758294104	-0.106223405	FALSE
GB18589	1617	837	0.950020151	0.085502643	FALSE
GB18590	292	153	0.932436716	0.067919208	FALSE
GB18591	4	1	2	1.135482492	TRUE
GB18592	9121	4208	1.116057301	0.251539793	FALSE
GB18593	NA	NA	NA	NA	FALSE
GB18594	252	141	0.837728571	-0.026788937	FALSE
GB18595	92	50	0.879705766	0.015188258	FALSE
GB18596	440	314	0.486738965	-0.377778544	FALSE
GB18597	8	4	1	0.135482492	FALSE
GB18598	165	91	0.858527574	-0.005989934	FALSE
GB18599	640	263	1.283009106	0.418491597	FALSE
GB18600	1319	852	0.630519229	-0.233998279	FALSE
GB18601	1	1	0	-0.864517508	TRUE
GB18602	238	85	1.485426827	0.620909319	TRUE
GB18603	562	277	1.020684154	0.156166646	FALSE
GB18604	NA	NA	NA	NA	FALSE
GB18605	1227	876	0.486132474	-0.378385034	FALSE
GB18606	598	243	1.299189171	0.434671662	FALSE
GB18607	213	144	0.564784619	-0.29973289	FALSE
GB18608	40	16	1.321928095	0.457410587	FALSE
GB18609	83	37	1.165586066	0.301068557	FALSE
GB18610	2913	1689	0.786336373	-0.078181135	FALSE
GB18611	86	43	1	0.135482492	FALSE
GB18612	296	175	0.758242254	-0.106275255	FALSE
GB18613	2166	1041	1.057063174	0.192545666	FALSE
GB18614	107	53	1.013546532	0.149029024	FALSE
GB18615	629	464	0.438935212	-0.425582297	FALSE
GB18616	369	189	0.965234582	0.100717074	FALSE
GB18617	489	318	0.6208077	-0.243709809	FALSE
GB18618	2999	1355	1.146188671	0.281671162	FALSE
GB18619	49608	30391	0.706928648	-0.15758886	FALSE
GB18620	701	291	1.268395291	0.403877783	FALSE

GB18621	1327	539	1.299811193	0.435293684	FALSE
GB18622	1813	1089	0.735374971	-0.129142537	FALSE
GB18623	8	8	0	-0.864517508	TRUE
GB18624	10	2	2.321928095	1.457410587	TRUE
GB18625	1205	615	0.970374831	0.105857323	FALSE
GB18626	13068	6451	1.018443638	0.15392613	FALSE
GB18627	68	44	0.628031223	-0.236486286	FALSE
GB18628	7	1	2.807354922	1.942837414	TRUE
GB18629	247	166	0.5733278	-0.291189708	FALSE
GB18630	1278	729	0.809897117	-0.054620392	FALSE
GB18631	NA	NA	NA	NA	FALSE
GB18632	1351	363	1.895986221	1.031468713	TRUE
GB18633	10463	8971	0.22195585	-0.642561658	TRUE
GB18634	1012	484	1.064130337	0.199612829	FALSE
GB18635	245	184	0.413075983	-0.451441525	FALSE
GB18636	2083	1318	0.660312469	-0.204205039	FALSE
GB18637	2734	2031	0.428823003	-0.435694505	FALSE
GB18638	404	183	1.142511644	0.277994136	FALSE
GB18639	601	334	0.847516888	-0.01700062	FALSE
GB18640	449	297	0.596252514	-0.268264994	FALSE
GB18641	NA	NA	NA	NA	FALSE
GB18642	3126	1437	1.121257717	0.256740208	FALSE
GB18643	4387	2473	0.826972467	-0.037545042	FALSE
GB18644	231	109	1.083564717	0.219047208	FALSE
GB18645	2614	1485	0.81579621	-0.048721298	FALSE
GB18646	11686	6145	0.927296279	0.06277877	FALSE
GB18647	7153	5163	0.470338843	-0.394178665	FALSE
GB18648	514	247	1.057257318	0.192739809	FALSE
GB18649	4312	4543	-0.075288127	-0.939805636	TRUE
GB18650	2187	1051	1.057190551	0.192673043	FALSE
GB18651	1896	975	0.95948484	0.094967332	FALSE
GB18652	163	92	0.825166198	-0.03935131	FALSE
GB18653	1847	1017	0.860864187	-0.003653321	FALSE
GB18654	43	26	0.725825037	-0.138692472	FALSE
GB18655	260	147	0.822695468	-0.04182204	FALSE
GB18656	61	42	0.538419915	-0.326097594	FALSE
GB18657	1	1	0	-0.864517508	TRUE
GB18658	1	0	NA	NA	FALSE
GB18659	329	208	0.661504056	-0.203013453	FALSE
GB18660	8	3	1.415037499	0.550519991	TRUE
GB18661	3387	1812	0.902425031	0.037907523	FALSE
GB18662	730	379	0.945698616	0.081181107	FALSE
GB18663	739	332	1.154391123	0.289873614	FALSE
GB18664	2715	1454	0.900924929	0.03640742	FALSE

GB18665	1	0	NA	NA	FALSE
GB18666	48	26	0.884522783	0.020005274	FALSE
GB18667	3311	1989	0.735223735	-0.129293774	FALSE
GB18668	20	5	2	1.135482492	TRUE
GB18669	5454	2444	1.158070415	0.293552907	FALSE
GB18670	9	7	0.362570079	-0.501947429	TRUE
GB18671	144	88	0.710493383	-0.154024126	FALSE
GB18672	1	0	NA	NA	FALSE
GB18673	1300	779	0.73881639	-0.125701118	FALSE
GB18674	431	233	0.887357914	0.022840406	FALSE
GB18675	210	171	0.296393003	-0.568124506	TRUE
GB18676	5759	2603	1.14564301	0.281125502	FALSE
GB18677	257	160	0.683696454	-0.180821054	FALSE
GB18678	10	2	2.321928095	1.457410587	TRUE
GB18679	69	20	1.786596362	0.922078854	TRUE
GB18680	202	129	0.646984227	-0.217533281	FALSE
GB18681	136	86	0.661198087	-0.203319422	FALSE
GB18682	314	213	0.559911129	-0.30460638	FALSE
GB18683	1585	884	0.842364566	-0.022152943	FALSE
GB18684	15565	5426	1.520344624	0.655827116	TRUE
GB18685	3971	1945	1.029732206	0.165214697	FALSE
GB18686	237	154	0.621956708	-0.2425608	FALSE
GB18687	308	209	0.559427409	-0.3050901	FALSE
GB18688	463	278	0.735927311	-0.128590198	FALSE
GB18689	2372	1224	0.954500452	0.089982944	FALSE
GB18690	242	132	0.874469118	0.00995161	FALSE
GB18691	3966	2710	0.549391826	-0.315125682	FALSE
GB18692	1336	712	0.907970862	0.043453353	FALSE
GB18693	670	436	0.619832961	-0.244684548	FALSE
GB18694	374	258	0.535667204	-0.328850304	FALSE
GB18695	1417	747	0.92365961	0.059142102	FALSE
GB18696	441	178	1.308901415	0.444383906	FALSE
GB18697	3	0	NA	NA	FALSE
GB18698	90	42	1.099535674	0.235018165	FALSE
GB18699	107	41	1.383914982	0.519397473	TRUE
GB18700	625	559	0.161007907	-0.703509602	TRUE
GB18701	2	0	NA	NA	FALSE
GB18702	13051	5765	1.178767841	0.314250333	FALSE
GB18703	595	487	0.288967896	-0.575549612	TRUE
GB18704	691	391	0.821517103	-0.043000405	FALSE
GB18705	18	13	0.469485283	-0.395032225	FALSE
GB18706	59	42	0.490325627	-0.374191882	FALSE
GB18707	36	7	2.362570079	1.498052571	TRUE
GB18708	NA	NA	NA	NA	FALSE

GB18709	168	88	0.932885804	0.068368296	FALSE
GB18710	982	455	1.109856479	0.245338971	FALSE
GB18711	NA	NA	NA	NA	FALSE
GB18712	2427	1475	0.718459154	-0.146058354	FALSE
GB18713	837	543	0.624275425	-0.240242084	FALSE
GB18714	97	65	0.577545029	-0.286972479	FALSE
GB18715	1089	670	0.700770953	-0.163746555	FALSE
GB18716	NA	NA	NA	NA	FALSE
GB18717	391	153	1.353636955	0.489119446	FALSE
GB18718	6	1	2.584962501	1.720444992	TRUE
GB18719	31431	16310	0.946431391	0.081913883	FALSE
GB18720	66	23	1.520832163	0.656314655	TRUE
GB18721	13	11	0.2410081	-0.623509409	TRUE
GB18722	131	68	0.94596016	0.081442652	FALSE
GB18723	329	122	1.431206436	0.566688928	TRUE
GB18724	711	485	0.551864813	-0.312652696	FALSE
GB18725	NA	NA	NA	NA	FALSE
GB18726	92	32	1.523561956	0.659044448	TRUE
GB18727	12270	7536	0.703264379	-0.161253129	FALSE
GB18728	363	170	1.094434802	0.229917294	FALSE
GB18729	475	244	0.961046366	0.096528857	FALSE
GB18730	192	94	1.030373649	0.165856141	FALSE
GB18731	49	37	0.405256478	-0.45926103	FALSE
GB18732	NA	NA	NA	NA	FALSE
GB18733	113	51	1.14775362	0.283236112	FALSE
GB18734	791	499	0.664637879	-0.199879629	FALSE
GB18735	81	30	1.432959407	0.568441899	TRUE
GB18736	679	236	1.524624715	0.660107207	TRUE
GB18737	41971	47606	-0.181750577	-1.046268085	TRUE
GB18738	1982	1262	0.651245052	-0.213272456	FALSE
GB18739	545	388	0.490199577	-0.374317931	FALSE
GB18740	0	3	NA	NA	FALSE
GB18741	470	240	0.969626351	0.105108843	FALSE
GB18742	771	398	0.953962429	0.089444921	FALSE
GB18743	NA	NA	NA	NA	FALSE
GB18744	199	129	0.625397365	-0.239120143	FALSE
GB18745	252	104	1.276840205	0.412322697	FALSE
GB18746	1179	590	0.998776859	0.13425935	FALSE
GB18747	NA	NA	NA	NA	FALSE
GB18748	1	0	NA	NA	FALSE
GB18749	97	62	0.645716532	-0.218800977	FALSE
GB18750	3632	1942	0.903221002	0.038703494	FALSE
GB18751	1331	619	1.104499257	0.239981748	FALSE
GB18752	1	0	NA	NA	FALSE

GB18753	653	340	0.941548245	0.077030737	FALSE
GB18754	NA	NA	NA	NA	FALSE
GB18755	476	252	0.91753784	0.053020331	FALSE
GB18756	1980	1015	0.964020703	0.099503195	FALSE
GB18757	1565	970	0.690106005	-0.174411504	FALSE
GB18758	838	513	0.707991418	-0.15652609	FALSE
GB18759	2	0	NA	NA	FALSE
GB18760	9889	6521	0.60073142	-0.263786089	FALSE
GB18761	394	246	0.679537314	-0.184980194	FALSE
GB18762	103	37	1.477047162	0.612529653	TRUE
GB18763	NA	NA	NA	NA	FALSE
GB18764	25937	17384	0.57725154	-0.287265968	FALSE
GB18765	2222	1333	0.737182036	-0.127335472	FALSE
GB18766	667	461	0.532920011	-0.331597498	FALSE
GB18767	49	29	0.756728849	-0.107788659	FALSE
GB18768	380	249	0.609853676	-0.254663832	FALSE
GB18769	7128	4876	0.547799211	-0.316718297	FALSE
GB18770	10753	6866	0.647197454	-0.217320055	FALSE
GB18771	7726	5225	0.56429064	-0.300226868	FALSE
GB18772	783	482	0.699979161	-0.164538347	FALSE
GB18773	193	106	0.864536583	[illegible]743775478897e-05	FALSE
GB18774	69	51	0.436099115	-0.428418394	FALSE
GB18775	556	256	1.118941073	0.254423564	FALSE
GB18776	487	264	0.883383843	0.018866334	FALSE
GB18777	0	1	NA	NA	FALSE
GB18778	15	8	0.906890596	0.042373087	FALSE
GB18779	NA	NA	NA	NA	FALSE
GB18780	41	23	0.833990049	-0.03052746	FALSE
GB18781	724	505	0.519706309	-0.344811199	FALSE
GB18782	NA	NA	NA	NA	FALSE
GB18783	2391	1211	0.981415265	0.116897757	FALSE
GB18784	27	10	1.432959407	0.568441899	TRUE
GB18785	621	251	1.306905904	0.442388396	FALSE
GB18786	34	10	1.765534746	0.901017238	TRUE
GB18787	NA	NA	NA	NA	FALSE
GB18788	416	150	1.471621028	0.607103519	TRUE
GB18789	762	326	1.224919033	0.360401525	FALSE
GB18790	549	381	0.527015152	-0.337502357	FALSE
GB18791	157	124	0.340424439	-0.52409307	TRUE
GB18792	1	1	0	-0.864517508	TRUE
GB18793	NA	NA	NA	NA	FALSE
GB18794	201	88	1.191620073	0.327102564	FALSE
GB18795	90	50	0.847996907	-0.016520602	FALSE
GB18796	130	69	0.913843356	0.049325848	FALSE

GB18797	819	522	0.649813645	-0.214703863	FALSE
GB18798	2280	1234	0.88569143	0.021173922	FALSE
GB18799	55	17	1.693896872	0.829379364	TRUE
GB18800	768	348	1.142019005	0.277501497	FALSE
GB18801	1088	645	0.754307491	-0.110210017	FALSE
GB18802	3469	1765	0.974851657	0.110334149	FALSE
GB18803	339	151	1.166736724	0.302219215	FALSE
GB18804	447	222	1.009715155	0.145197647	FALSE
GB18805	64	31	1.04580369	0.181286181	FALSE
GB18806	205	108	0.924592597	0.060075089	FALSE
GB18807	83	54	0.620151929	-0.244365579	FALSE
GB18808	310	280	0.146841388	-0.71767612	TRUE
GB18809	530	377	0.491427836	-0.373089672	FALSE
GB18810	513	250	1.037030731	0.172513223	FALSE
GB18811	19	8	1.247927513	0.383410005	FALSE
GB18812	272	162	0.747612838	-0.11690467	FALSE
GB18813	379	183	1.0503542	0.185836692	FALSE
GB18814	780	371	1.072054937	0.207537429	FALSE
GB18815	6112	3447	0.826303245	-0.038214264	FALSE
GB18816	380	123	1.627341103	0.762823595	TRUE
GB18817	58	16	1.857980995	0.993463487	TRUE
GB18818	517	339	0.608879007	-0.255638501	FALSE
GB18819	53220	22272	1.256737394	0.392219886	FALSE
GB18820	5475	2733	1.002373505	0.137855997	FALSE
GB18821	408	237	0.783682093	-0.080835415	FALSE
GB18822	425	276	0.622794574	-0.241722934	FALSE
GB18823	1	1	0	-0.864517508	TRUE
GB18824	713	351	1.022431046	0.157913538	FALSE
GB18825	255	132	0.949959318	0.085441809	FALSE
GB18826	1451	900	0.689050613	-0.175466895	FALSE
GB18827	NA	NA	NA	NA	FALSE
GB18828	820	488	0.748742762	-0.115774746	FALSE
GB18829	NA	NA	NA	NA	FALSE
GB18830	412	223	0.885600627	0.021083119	FALSE
GB18831	6	2	1.584962501	0.720444992	TRUE
GB18832	114	78	0.547487795	-0.317029713	FALSE
GB18833	3	0	NA	NA	FALSE
GB18834	226	189	0.257936538	-0.60658097	TRUE
GB18835	17	9	0.91753784	0.053020331	FALSE
GB18836	25	12	1.058893689	0.194376181	FALSE
GB18837	323	161	1.004473477	0.139955968	FALSE
GB18838	9	18	-1	-1.864517508	TRUE
GB18839	408	224	0.86507042	0.000552912	FALSE
GB18840	509	369	0.46404484	-0.400472668	FALSE

GB18841	355	182	0.963880574	0.099363066	FALSE
GB18842	868	484	0.842687995	-0.021829513	FALSE
GB18843	1733	1223	0.502847251	-0.361670258	FALSE
GB18844	9407	5461	0.784569548	-0.079947961	FALSE
GB18845	3	0	NA	NA	FALSE
GB18846	546	292	0.902932582	0.038415074	FALSE
GB18847	511	334	0.613475188	-0.25104232	FALSE
GB18848	2	0	NA	NA	FALSE
GB18849	163	70	1.219445137	0.354927629	FALSE
GB18850	4928	2449	1.008809482	0.144291974	FALSE
GB18851	619	330	0.907473385	0.042955877	FALSE
GB18852	698	469	0.573639114	-0.290878395	FALSE
GB18853	108	70	0.625604485	-0.238913023	FALSE
GB18854	971	558	0.799206174	-0.065311335	FALSE
GB18855	NA	NA	NA	NA	FALSE
GB18856	49	29	0.756728849	-0.107788659	FALSE
GB18857	817	474	0.785449019	-0.079068489	FALSE
GB18858	479	337	0.507277065	-0.357240444	FALSE
GB18859	886	392	1.176453044	0.311935536	FALSE
GB18860	201	103	0.964551164	0.100033656	FALSE
GB18861	2123	1132	0.907230413	0.042712905	FALSE
GB18862	51	28	0.86507042	0.000552912	FALSE
GB18863	78	46	0.761840263	-0.102677246	FALSE
GB18864	2389	1306	0.871251957	0.006734449	FALSE
GB18865	1	0	NA	NA	FALSE
GB18866	1357	579	1.228785467	0.364267959	FALSE
GB18867	3599	1943	0.889310201	0.024792693	FALSE
GB18868	2	3	-0.584962501	-1.449480009	TRUE
GB18869	284	95	1.579891511	0.715374003	TRUE
GB18870	265	160	0.727920455	-0.136597054	FALSE
GB18871	0	1	NA	NA	FALSE
GB18872	64	43	0.573735245	-0.290782263	FALSE
GB18873	4	0	NA	NA	FALSE
GB18874	1543	1027	0.58730188	-0.277215628	FALSE
GB18875	12	6	1	0.135482492	FALSE
GB18876	98	42	1.222392421	0.357874913	FALSE
GB18877	1134	604	0.908800186	0.044282677	FALSE
GB18878	194	111	0.805496976	-0.059020532	FALSE
GB18879	65	53	0.294447358	-0.57007015	TRUE
GB18880	957	396	1.273018494	0.408500986	FALSE
GB18881	311	127	1.292086083	0.427568575	FALSE
GB18882	540	280	0.94753258	0.083015072	FALSE
GB18883	254	109	1.220500362	0.355982854	FALSE
GB18884	34	15	1.180572246	0.316054737	FALSE

GB18885	958	471	1.024298596	0.159781088	FALSE
GB18886	85	42	1.017073513	0.152556005	FALSE
GB18887	1285	653	0.976613463	0.112095954	FALSE
GB18888	108	77	0.488100961	-0.376416547	FALSE
GB18889	16	7	1.192645078	0.32812757	FALSE
GB18890	221	164	0.430350555	-0.434166954	FALSE
GB18891	5617	3394	0.726813238	-0.13770427	FALSE
GB18892	1119	705	0.666514874	-0.198002635	FALSE
GB18893	193	93	1.053298226	0.188780718	FALSE
GB18894	269	208	0.371022644	-0.493494864	FALSE
GB18895	348	173	1.008315268	0.14379776	FALSE
GB18896	19591	7706	1.346136949	0.481619441	FALSE
GB18897	1980	1055	0.908257431	0.043739923	FALSE
GB18898	9440	7104	0.410155278	-0.45436223	FALSE
GB18899	10938	8584	0.349626986	-0.514890522	TRUE
GB18900	11	1	3.459431619	2.59491411	TRUE
GB18901	12	7	0.777607579	-0.08690993	FALSE
GB18902	303	189	0.680931559	-0.183585949	FALSE
GB18903	NA	NA	NA	NA	FALSE
GB18904	8160	6192	0.398163681	-0.466353828	FALSE
GB18905	4140	2875	0.526068812	-0.338448697	FALSE
GB18906	919	457	1.007870696	0.143353188	FALSE
GB18907	3460	2418	0.516957793	-0.347559715	FALSE
GB18908	12	3	2	1.135482492	TRUE
GB18909	181	62	1.545649577	0.681132068	TRUE
GB18910	739	457	0.693380199	-0.171137309	FALSE
GB18911	243	129	0.913585248	0.04906774	FALSE
GB18912	22281	13396	0.734011703	-0.130505805	FALSE
GB18913	3665	2181	0.748823428	-0.11569408	FALSE
GB18914	430	211	1.027093661	0.162576153	FALSE
GB18915	417	249	0.743901641	-0.120615867	FALSE
GB18916	339	163	1.056413309	0.191895801	FALSE
GB18917	9876	5392	0.87310634	0.008588832	FALSE
GB18918	3898	2968	0.393242999	-0.47127451	FALSE
GB18919	4	0	NA	NA	FALSE
GB18920	1588	983	0.691947591	-0.172569918	FALSE
GB18921	3225	1527	1.078599098	0.21408159	FALSE
GB18922	610	324	0.91281543	0.048297921	FALSE
GB18923	2615	1368	0.934742716	0.070225208	FALSE
GB18924	48	16	1.584962501	0.720444992	TRUE
GB18925	5	2	1.321928095	0.457410587	FALSE
GB18926	1	0	NA	NA	FALSE
GB18927	1562	685	1.18921856	0.324701052	FALSE
GB18928	737	309	1.254057781	0.389540273	FALSE

GB18929	11	3	1.874469118	1.00995161	TRUE
GB18930	5226	2660	0.974280879	0.109763371	FALSE
GB18931	493	212	1.217523382	0.353005873	FALSE
GB18932	607	353	0.782028333	-0.082489175	FALSE
GB18933	61	17	1.843274496	0.978756988	TRUE
GB18934	949	672	0.497946854	-0.366570654	FALSE
GB18935	1357	643	1.077530078	0.21301257	FALSE
GB18936	930	434	1.099535674	0.235018165	FALSE
GB18937	1329	679	0.968857625	0.104340117	FALSE
GB18938	1417	904	0.648445081	-0.216072428	FALSE
GB18939	256	139	0.881058927	0.016541419	FALSE
GB18940	21	10	1.070389328	0.20587182	FALSE
GB18941	1501	684	1.133855747	0.269338238	FALSE
GB18942	2	0	NA	NA	FALSE
GB18943	154	81	0.926936538	0.062419029	FALSE
GB18944	23	4	2.523561956	1.659044448	TRUE
GB18945	2137	1442	0.567515743	-0.297001765	FALSE
GB18946	404	244	0.727474145	-0.137043363	FALSE
GB18947	352	146	1.26960706	0.405089551	FALSE
GB18948	1974	1278	0.627234154	-0.237283355	FALSE
GB18949	1036	588	0.817135943	-0.047381565	FALSE
GB18950	0	2	NA	NA	FALSE
GB18951	0	1	NA	NA	FALSE
GB18952	2708	1594	0.76457611	-0.099941399	FALSE
GB18953	1054	884	0.253756592	-0.610760916	TRUE
GB18954	5909	4226	0.483621232	-0.380896277	FALSE
GB18955	2640	1500	0.815575429	-0.048942079	FALSE
GB18956	1756	1201	0.548056694	-0.316460814	FALSE
GB18957	28	13	1.106915204	0.242397696	FALSE
GB18958	41	25	0.713695815	-0.150821693	FALSE
GB18959	725	389	0.89821084	0.033693332	FALSE
GB18960	1488	867	0.779270628	-0.08524688	FALSE
GB18961	4	0	NA	NA	FALSE
GB18962	55	28	0.974004791	0.109487283	FALSE
GB18963	69	48	0.523561956	-0.340955552	FALSE
GB18964	87	34	1.355480655	0.490963146	FALSE
GB18965	1446	897	0.688887662	-0.175629846	FALSE
GB18966	93	72	0.36923381	-0.495283699	FALSE
GB18967	3509	1952	0.846106895	-0.018410613	FALSE
GB18968	NA	NA	NA	NA	FALSE
GB18969	42451	36307	0.225550812	-0.638966696	TRUE
GB18970	0	1	NA	NA	FALSE
GB18971	892	332	1.425860469	0.56134296	TRUE
GB18972	88	28	1.652076697	0.787559188	TRUE

GB18973	571	294	0.957674591	0.093157082	FALSE
GB18974	1129	703	0.683448892	-0.181068617	FALSE
GB18975	NA	NA	NA	NA	FALSE
GB18976	771	384	1.005624549	0.141107041	FALSE
GB18977	262	162	0.693572999	-0.17094451	FALSE
GB18978	1173	674	0.799382517	-0.065134991	FALSE
GB18979	477	302	0.659440717	-0.205076792	FALSE
GB18980	NA	NA	NA	NA	FALSE
GB18981	912	432	1.078002512	0.213485004	FALSE
GB18982	317	177	0.84073348	-0.023784028	FALSE
GB18983	1577	942	0.743383695	-0.121133813	FALSE
GB18984	2106	1433	0.555466827	-0.309050682	FALSE
GB18985	541	251	1.10794123	0.243423722	FALSE
GB18986	1574	1065	0.56358211	-0.300935398	FALSE
GB18987	4041	2496	0.695094417	-0.169423091	FALSE
GB18988	3293	1734	0.925298613	0.060781105	FALSE
GB18989	681	379	0.84545695	-0.019060558	FALSE
GB18990	3	2	0.584962501	-0.279555008	FALSE
GB18991	416	216	0.945552216	0.081034708	FALSE
GB18992	19	6	1.662965013	0.798447504	TRUE
GB18993	3333	1762	0.919607393	0.055089885	FALSE
GB18994	1199	739	0.698185389	-0.166332119	FALSE
GB18995	182	90	1.015941544	0.151424036	FALSE
GB18996	270	189	0.514573173	-0.349944335	FALSE
GB18997	716	368	0.960253821	0.095736313	FALSE
GB18998	1738	1001	0.795986108	-0.0685314	FALSE
GB18999	828	413	1.003488986	0.138971478	FALSE
GB19000	109	100	0.124328135	-0.740189373	TRUE
GB19001	2440	1180	1.048094288	0.18357678	FALSE
GB19002	376	182	1.046794211	0.182276703	FALSE
GB19003	2755	1819	0.598906776	-0.265610733	FALSE
GB19004	6295	3811	0.72403677	-0.140480739	FALSE
GB19005	644	424	0.602996424	-0.261521085	FALSE
GB19006	12124	5995	1.016034098	0.15151659	FALSE
GB19007	408	183	1.156725504	0.292207995	FALSE
GB19008	1140	597	0.933230988	0.068713479	FALSE
GB19009	381	193	0.98119015	0.116672642	FALSE
GB19010	65	39	0.736965594	-0.127551914	FALSE
GB19011	19	9	1.078002512	0.213485004	FALSE
GB19012	3269	1779	0.877782867	0.013265358	FALSE
GB19013	25	15	0.736965594	-0.127551914	FALSE
GB19014	752	602	0.320969175	-0.543548333	TRUE
GB19015	932	410	1.184706045	0.320188537	FALSE
GB19016	11571	6520	0.827569682	-0.036947826	FALSE

GB19017	1765	1340	0.397435183	-0.467082326	FALSE
GB19018	332	226	0.554860469	-0.309657039	FALSE
GB19019	1096	561	0.966175122	0.101657614	FALSE
GB19020	1542	553	1.47945138	0.614933871	TRUE
GB19021	53	22	1.268488836	0.403971328	FALSE
GB19022	34517	19940	0.79164167	-0.072875838	FALSE
GB19023	3777	2034	0.892921105	0.028403596	FALSE
GB19024	94	45	1.062735755	0.198218247	FALSE
GB19025	4	3	0.415037499	-0.449480009	FALSE
GB19026	583	346	0.752723846	-0.111793663	FALSE
GB19027	368	228	0.690671942	-0.173845566	FALSE
GB19028	964	504	0.935609413	0.071091904	FALSE
GB19029	16	2	3	2.135482492	TRUE
GB19030	12420	9537	0.381057751	-0.483459757	FALSE
GB19031	62	26	1.253756592	0.389239084	FALSE
GB19032	143	87	0.716927841	-0.147589667	FALSE
GB19033	54	27	1	0.135482492	FALSE
GB19034	638	343	0.895347848	0.030830339	FALSE
GB19035	610	220	1.471305719	0.606788211	TRUE
GB19036	80	51	0.649502753	-0.215014755	FALSE
GB19037	174	72	1.273018494	0.408500986	FALSE
GB19038	57	36	0.662965013	-0.201552496	FALSE
GB19039	514	295	0.801053405	-0.063464103	FALSE
GB19040	66	17	1.956931278	1.09241377	TRUE
GB19041	1257	616	1.028982394	0.164464885	FALSE
GB19042	850	490	0.794681092	-0.069836416	FALSE
GB19043	18	15	0.263034406	-0.601483102	TRUE
GB19044	NA	NA	NA	NA	FALSE
GB19045	NA	NA	NA	NA	FALSE
GB19046	548	366	0.582332245	-0.282185264	FALSE
GB19047	487	248	0.973581652	0.109064143	FALSE
GB19048	3879	2708	0.518457037	-0.346060471	FALSE
GB19049	154	94	0.712197689	-0.152319819	FALSE
GB19050	695	415	0.743901641	-0.120615867	FALSE
GB19051	51	42	0.280107919	-0.584409589	TRUE
GB19052	12712	7260	0.808149576	-0.056367932	FALSE
GB19053	1310	850	0.624032065	-0.240485443	FALSE
GB19054	332	197	0.752987612	-0.111529896	FALSE
GB19055	99	40	1.307428525	0.442911017	FALSE
GB19056	197	99	0.992695199	0.128177691	FALSE
GB19057	1542	944	0.707944001	-0.156573508	FALSE
GB19058	1458	741	0.976445272	0.111927764	FALSE
GB19059	53	43	0.3016557	-0.562861808	TRUE
GB19060	2783	1608	0.791373502	-0.073144006	FALSE

GB19061	1210	925	0.387481777	-0.477035732	FALSE
GB19062	417	222	0.909487707	0.044970199	FALSE
GB19063	157	89	0.818887318	-0.04563019	FALSE
GB19064	769	495	0.635555073	-0.228962435	FALSE
GB19065	900	452	0.993602229	0.12908472	FALSE
GB19066	9	12	-0.415037499	-1.279555008	TRUE
GB19067	3	1	1.584962501	0.720444992	TRUE
GB19068	1689	983	0.780906006	-0.083611502	FALSE
GB19069	1602	962	0.735765349	-0.12875216	FALSE
GB19070	3608	3362	0.101879614	-0.762637894	TRUE
GB19071	1472	700	1.072350844	0.207833336	FALSE
GB19072	296	174	0.76650987	-0.098007639	FALSE
GB19073	NA	NA	NA	NA	FALSE
GB19074	242	102	1.246437895	0.381920387	FALSE
GB19075	11448	6402	0.838500995	-0.026016513	FALSE
GB19076	2365	1317	0.844584838	-0.01993267	FALSE
GB19077	1593	1164	0.452655209	-0.4118623	FALSE
GB19078	7125	4833	0.559971019	-0.304546489	FALSE
GB19079	840	416	1.0138058	0.149288291	FALSE
GB19080	597	444	0.427171255	-0.437346253	FALSE
GB19081	6	1	2.584962501	1.720444992	TRUE
GB19082	28180	17485	0.688553816	-0.175963693	FALSE
GB19083	4	20	-2.321928095	-3.186445603	TRUE
GB19084	1445	721	1.002998328	0.13848082	FALSE
GB19085	12231	6634	0.882591445	0.018073937	FALSE
GB19086	5	0	NA	NA	FALSE
GB19087	431	231	0.899795018	0.035277509	FALSE
GB19088	NA	NA	NA	NA	FALSE
GB19089	167	161	0.052787414	-0.811730094	TRUE
GB19090	168	79	1.088536675	0.224019166	FALSE
GB19091	21	6	1.807354922	0.942837414	TRUE
GB19092	903	513	0.815767162	-0.048750346	FALSE
GB19093	160	76	1.074000581	0.209483073	FALSE
GB19094	NA	NA	NA	NA	FALSE
GB19095	473	248	0.931500063	0.066982555	FALSE
GB19096	745	506	0.558103041	-0.306414468	FALSE
GB19097	859	491	0.806935107	-0.057582402	FALSE
GB19098	21	16	0.392317423	-0.472200086	FALSE
GB19099	575	306	0.910030303	0.045512795	FALSE
GB19100	574	238	1.270089163	0.405571655	FALSE
GB19101	2406	1024	1.232420927	0.367903419	FALSE
GB19102	1194	603	0.985572929	0.121055421	FALSE
GB19103	20405	11684	0.804388447	-0.060129061	FALSE
GB19104	2	0	NA	NA	FALSE

GB19105	0	2	NA	NA	FALSE
GB19106	833	526	0.663253696	-0.201263812	FALSE
GB19107	1	0	NA	NA	FALSE
GB19108	1081	569	0.925865965	0.061348457	FALSE
GB19109	123	79	0.638733757	-0.225783751	FALSE
GB19110	288	144	1	0.135482492	FALSE
GB19111	2531	1344	0.913174369	0.048656861	FALSE
GB19112	NA	NA	NA	NA	FALSE
GB19113	1015	276	1.878739555	1.014222047	TRUE
GB19114	1129	768	0.55586727	-0.308650238	FALSE
GB19115	1073	621	0.788984903	-0.075532606	FALSE
GB19116	8	5	0.678071905	-0.186445603	FALSE
GB19117	8	3	1.415037499	0.550519991	TRUE
GB19118	4	5	-0.321928095	-1.186445603	TRUE
GB19119	591	361	0.711159293	-0.153358215	FALSE
GB19120	45	28	0.684498174	-0.180019334	FALSE
GB19121	1340	765	0.808701348	-0.055816161	FALSE
GB19122	553	248	1.15693936	0.292421852	FALSE
GB19123	1550	912	0.765162486	-0.099355022	FALSE
GB19124	928	774	0.261791239	-0.602726269	TRUE
GB19125	NA	NA	NA	NA	FALSE
GB19126	1027	625	0.716508087	-0.148009422	FALSE
GB19127	140	72	0.959358016	0.094840507	FALSE
GB19128	300	105	1.514573173	0.650055665	TRUE
GB19129	732	414	0.822212881	-0.042304628	FALSE
GB19130	888	638	0.477003253	-0.387514256	FALSE
GB19131	438	241	0.861897723	-0.002619785	FALSE
GB19132	4162	2899	0.521721632	-0.342795877	FALSE
GB19133	548	252	1.120752159	0.256234651	FALSE
GB19134	NA	NA	NA	NA	FALSE
GB19135	3	2	0.584962501	-0.279555008	FALSE
GB19136	0	1	NA	NA	FALSE
GB19137	135	93	0.537656786	-0.326860722	FALSE
GB19138	405	224	0.854423176	-0.010094333	FALSE
GB19139	19	19	0	-0.864517508	TRUE
GB19140	658	307	1.099848928	0.23533142	FALSE
GB19141	1	0	NA	NA	FALSE
GB19142	581	277	1.068652187	0.204134679	FALSE
GB19143	304	99	1.618570893	0.754053385	TRUE
GB19144	5	1	2.321928095	1.457410587	TRUE
GB19145	1231	723	0.76776321	-0.096754299	FALSE
GB19146	143	86	0.733606582	-0.130910926	FALSE
GB19147	263	131	1.005495988	0.140978479	FALSE
GB19148	9	7	0.362570079	-0.501947429	TRUE

GB19149	61	35	0.801454321	-0.063063188	FALSE
GB19150	480	324	0.567040593	-0.297476916	FALSE
GB19151	246	87	1.499571009	0.635053501	TRUE
GB19152	26	13	1	0.135482492	FALSE
GB19153	NA	NA	NA	NA	FALSE
GB19154	1547	892	0.794357582	-0.070159927	FALSE
GB19155	8070	4378	0.882296719	0.017779211	FALSE
GB19156	470	269	0.805054584	-0.059462924	FALSE
GB19157	132	84	0.652076697	-0.212440812	FALSE
GB19158	282	154	0.872764812	0.008247303	FALSE
GB19159	170	68	1.321928095	0.457410587	FALSE
GB19160	1337	656	1.027231745	0.162714237	FALSE
GB19161	2142	1231	0.799127718	-0.06538979	FALSE
GB19162	768	463	0.730094117	-0.134423391	FALSE
GB19163	65	30	1.115477217	0.250959709	FALSE
GB19164	NA	NA	NA	NA	FALSE
GB19165	454	277	0.712806321	-0.151711187	FALSE
GB19166	7329	3889	0.91421713	0.049699621	FALSE
GB19167	132	66	1	0.135482492	FALSE
GB19168	2655	1501	0.822787884	-0.041729624	FALSE
GB19169	5355	2474	1.114041075	0.249523566	FALSE
GB19170	475	280	0.762500686	-0.102016822	FALSE
GB19171	13783	6476	1.089715048	0.225197539	FALSE
GB19172	441	264	0.740240726	-0.124276782	FALSE
GB19173	1049	719	0.544951002	-0.319566506	FALSE
GB19174	414	267	0.632791026	-0.231726483	FALSE
GB19175	16	13	0.299560282	-0.564957226	TRUE
GB19176	298	187	0.672274061	-0.192243448	FALSE
GB19177	1	1	0	-0.864517508	TRUE
GB19178	690	369	0.902975546	0.038458037	FALSE
GB19179	27	14	0.94753258	0.083015072	FALSE
GB19180	611	417	0.551124996	-0.313392512	FALSE
GB19181	179	133	0.428533342	-0.435984167	FALSE
GB19182	73	63	0.212544635	-0.651972873	TRUE
GB19183	24	11	1.125530882	0.261013374	FALSE
GB19184	169	85	0.9914885	0.126970992	FALSE
GB19185	340	171	0.991538421	0.127020913	FALSE
GB19186	3582	2090	0.777262395	-0.087255113	FALSE
GB19187	257	116	1.147643554	0.283126046	FALSE
GB19188	1230	822	0.581448017	-0.283069492	FALSE
GB19189	3067	1992	0.622610519	-0.241906989	FALSE
GB19190	1013	550	0.88113065	0.016613142	FALSE
GB19191	8	6	0.415037499	-0.449480009	FALSE
GB19192	29	17	0.770518154	-0.093999354	FALSE

GB19193	5719	3189	0.842658808	-0.0218587	FALSE
GB19194	664	313	1.085020584	0.220503076	FALSE
GB19195	293	138	1.086232398	0.221714889	FALSE
GB19196	669	369	0.858385395	-0.006132114	FALSE
GB19197	1073	604	0.829029621	-0.035487887	FALSE
GB19198	726	318	1.190942783	0.326425274	FALSE
GB19199	532	277	0.941540269	0.077022761	FALSE
GB19200	7095	4697	0.595063091	-0.269454418	FALSE
GB19201	167	134	0.317615102	-0.546902406	TRUE
GB19202	1	1	0	-0.864517508	TRUE
GB19203	46	32	0.523561956	-0.340955552	FALSE
GB19204	502	205	1.292063454	0.427545946	FALSE
GB19205	2531	1541	0.715840646	-0.148676863	FALSE
GB19206	449	254	0.821886948	-0.04263056	FALSE
GB19207	261	118	1.145262947	0.280745439	FALSE
GB19208	4116	2598	0.663841551	-0.200675957	FALSE
GB19209	NA	NA	NA	NA	FALSE
GB19210	798	498	0.680243004	-0.184274504	FALSE
GB19211	1430	747	0.936834999	0.072317491	FALSE
GB19212	206	142	0.536753408	-0.327764101	FALSE
GB19213	NA	NA	NA	NA	FALSE
GB19214	415	291	0.512092183	-0.352425325	FALSE
GB19215	1025	608	0.753480681	-0.111036827	FALSE
GB19216	1	1	0	-0.864517508	TRUE
GB19217	8	3	1.415037499	0.550519991	TRUE
GB19218	2704	1440	0.90902634	0.044508832	FALSE
GB19219	80	48	0.736965594	-0.127551914	FALSE
GB19220	221	99	1.158545939	0.294028431	FALSE
GB19221	198	89	1.153623189	0.289105681	FALSE
GB19222	1172	714	0.71497659	-0.149540918	FALSE
GB19223	5	3	0.736965594	-0.127551914	FALSE
GB19224	7508	4903	0.614763889	-0.24975362	FALSE
GB19225	316	187	0.756886288	-0.10763122	FALSE
GB19226	2270	1556	0.544850237	-0.319667271	FALSE
GB19227	454	268	0.760459297	-0.104058211	FALSE
GB19228	605	228	1.407901318	0.54338381	TRUE
GB19230	469	241	0.960554776	0.096037268	FALSE
GB19231	4	2	1	0.135482492	FALSE
GB19232	406	264	0.620941798	-0.243575711	FALSE
GB19233	1768	1011	0.806335277	-0.058182231	FALSE
GB19234	387	212	0.868269302	0.003751793	FALSE
GB19235	444	223	0.993515966	0.128998458	FALSE
GB19236	718	381	0.914192846	0.049675338	FALSE
GB19237	1372	654	1.068917941	0.204400432	FALSE

GB19238	1335	751	0.829954929	-0.034562579	FALSE
GB19239	22	9	1.289506617	0.424989109	FALSE
GB19240	839	513	0.709711985	-0.154805523	FALSE
GB19241	869	442	0.975309807	0.110792299	FALSE
GB19242	18	7	1.362570079	0.498052571	FALSE
GB19243	649	393	0.723689166	-0.140828343	FALSE
GB19244	190	88	1.11042399	0.245906481	FALSE
GB19245	1681	825	1.0268537	0.162336192	FALSE
GB19246	449	268	0.744482444	-0.120035064	FALSE
GB19247	91311	55548	0.7170537	-0.147463808	FALSE
GB19248	238	170	0.485426827	-0.379090681	FALSE
GB19249	9	1	3.169925001	2.305407493	TRUE
GB19250	25	12	1.058893689	0.194376181	FALSE
GB19251	698	377	0.888662513	0.024145005	FALSE
GB19252	944	588	0.682970705	-0.181546804	FALSE
GB19253	134	66	1.021695071	0.157177563	FALSE
GB19254	7	1	2.807354922	1.942837414	TRUE
GB19255	1	1	0	-0.864517508	TRUE
GB19256	684	463	0.562984132	-0.301533377	FALSE
GB19257	18	6	1.584962501	0.720444992	TRUE
GB19258	2770	1429	0.95488006	0.090362552	FALSE
GB19259	4	8	-1	-1.864517508	TRUE
GB19260	794	398	0.996370577	0.131853068	FALSE
GB19261	203	128	0.665335917	-0.199181591	FALSE
GB19262	629	464	0.438935212	-0.425582297	FALSE
GB19263	37	13	1.509013647	0.644496139	TRUE
GB19264	160	65	1.299560282	0.435042774	FALSE
GB19265	6375	4327	0.559058221	-0.305459287	FALSE
GB19266	3798	2374	0.677919971	-0.186597538	FALSE
GB19267	119	46	1.371255807	0.506738299	TRUE
GB19268	324	183	0.824150165	-0.040367344	FALSE
GB19269	239	96	1.315904307	0.451386799	FALSE
GB19270	217	57	1.928661218	1.06414371	TRUE
GB19271	2646	1446	0.871745509	0.007228001	FALSE
GB19272	2517	1404	0.842162281	-0.022355227	FALSE
GB19273	7118	4843	0.55557093	-0.308946578	FALSE
GB19274	219	201	0.123735368	-0.74078214	TRUE
GB19275	2011	1209	0.734098837	-0.130418671	FALSE
GB19276	1865	972	0.940147412	0.075629903	FALSE
GB19277	11	11	0	-0.864517508	TRUE
GB19278	1490	653	1.190157434	0.325639925	FALSE
GB19279	403	256	0.654636029	-0.20988148	FALSE
GB19280	491	271	0.857430173	-0.007087335	FALSE
GB19281	94	55	0.773229138	-0.09128837	FALSE

GB19282	403	220	0.873276315	0.008758807	FALSE
GB19283	543	358	0.600992611	-0.263524898	FALSE
GB19284	338	202	0.742667954	-0.121849555	FALSE
GB19285	868	471	0.881967983	0.017450475	FALSE
GB19286	619	363	0.769969861	-0.094547647	FALSE
GB19287	472	254	0.893958363	0.029440854	FALSE
GB19288	NA	NA	NA	NA	FALSE
GB19289	32	17	0.912537159	0.04801965	FALSE
GB19290	849	331	1.358933337	0.494415828	FALSE
GB19291	414	245	0.756849018	-0.10766849	FALSE
GB19292	6	5	0.263034406	-0.601483102	TRUE
GB19293	756	549	0.461580085	-0.402937423	FALSE
GB19294	135	70	0.94753258	0.083015072	FALSE
GB19295	2830	1341	1.077492816	0.212975307	FALSE
GB19296	1501	726	1.047882524	0.183365015	FALSE
GB19297	14705	7676	0.937880168	0.073362659	FALSE
GB19298	0	1	NA	NA	FALSE
GB19299	5	3	0.736965594	-0.127551914	FALSE
GB19300	1809	953	0.924644289	0.06012678	FALSE
GB19301	2270	1393	0.70449704	-0.160020469	FALSE
GB19302	5151	2385	1.110863274	0.246345766	FALSE
GB19303	259	141	0.877256935	0.012739427	FALSE
GB19304	178	78	1.190331212	0.325813704	FALSE
GB19305	30106	20034	0.587600539	-0.276916969	FALSE
GB19306	2232	1634	0.449929044	-0.414588465	FALSE
GB19307	21	26	-0.308122295	-1.172639804	TRUE
GB19308	429	235	0.868316891	0.003799383	FALSE
GB19309	1226	548	1.161711181	0.297193672	FALSE
GB19310	139	71	0.969193953	0.104676445	FALSE
GB19311	2376	1455	0.707515683	-0.157001825	FALSE
GB19312	618	339	0.866321565	0.001804056	FALSE
GB19313	406	245	0.728697978	-0.13581953	FALSE
GB19314	4000	2271	0.816672294	-0.047845214	FALSE
GB19315	1	1	0	-0.864517508	TRUE
GB19316	866	396	1.128866595	0.264349086	FALSE
GB19317	428	159	1.428584031	0.564066523	TRUE
GB19318	4	1	2	1.135482492	TRUE
GB19319	153	103	0.570887316	-0.293630193	FALSE
GB19320	85	42	1.017073513	0.152556005	FALSE
GB19321	1277	740	0.787161349	-0.077356159	FALSE
GB19322	472	160	1.560714954	0.696197446	TRUE
GB19323	NA	NA	NA	NA	FALSE
GB19324	389	168	1.211308922	0.346791414	FALSE
GB19325	3	4	-0.415037499	-1.279555008	TRUE

GB19326	533	339	0.65285026	-0.211667249	FALSE
GB19327	1	0	NA	NA	FALSE
GB19328	4453	1760	1.339202183	0.474684675	FALSE
GB19329	NA	NA	NA	NA	FALSE
GB19330	169	107	0.65941245	-0.205105058	FALSE
GB19331	135	92	0.553253641	-0.311263867	FALSE
GB19332	624	341	0.87177429	0.007256782	FALSE
GB19333	10	16	-0.678071905	-1.542589413	TRUE
GB19334	73	37	0.980371193	0.115853685	FALSE
GB19335	88	45	0.967578522	0.103061014	FALSE
GB19336	0	1	NA	NA	FALSE
GB19337	8	4	1	0.135482492	FALSE
GB19338	48240	27912	0.789344405	-0.075173104	FALSE
GB19339	1	0	NA	NA	FALSE
GB19340	NA	NA	NA	NA	FALSE
GB19341	608	433	0.489704299	-0.37481321	FALSE
GB19342	539	326	0.725413309	-0.1391042	FALSE
GB19343	528	269	0.972931757	0.108414248	FALSE
GB19344	467	182	1.3594841	0.494966591	FALSE
GB19345	2245	971	1.209172244	0.344654736	FALSE
GB19346	1311	715	0.874652539	0.01013503	FALSE
GB19347	1448	1521	-0.070958551	-0.935476059	TRUE
GB19348	373	216	0.788144318	-0.07637319	FALSE
GB19349	73	42	0.797507136	-0.067010372	FALSE
GB19350	1277	668	0.934838517	0.070321009	FALSE
GB19351	4254	2460	0.790161718	-0.074355791	FALSE
GB19352	41	24	0.772589504	-0.091928004	FALSE
GB19353	1	0	NA	NA	FALSE
GB19354	876	516	0.763559804	-0.100957704	FALSE
GB19355	2005	1090	0.879274102	0.014756593	FALSE
GB19356	6	2	1.584962501	0.720444992	TRUE
GB19357	858	523	0.714166701	-0.150350807	FALSE
GB19358	274	177	0.630426533	-0.234090975	FALSE
GB19359	7618	4003	0.928330672	0.063813164	FALSE
GB19360	204	81	1.332575339	0.468057831	FALSE
GB19361	1	4	-2	-2.864517508	TRUE
GB19362	63	16	1.977279923	1.112762415	TRUE
GB19363	82	52	0.657112286	-0.207405222	FALSE
GB19364	318	169	0.912003519	0.047486011	FALSE
GB19365	395	200	0.981852653	0.117335145	FALSE
GB19366	NA	NA	NA	NA	FALSE
GB19367	33	7	2.237039197	1.372521689	TRUE
GB19368	837	477	0.811238357	-0.053279152	FALSE
GB19369	742	452	0.715096414	-0.149421094	FALSE

GB19370	2584	1364	0.921762426	0.057244917	FALSE
GB19371	3304	1582	1.062464087	0.197946579	FALSE
GB19372	24	29	-0.273018494	-1.137536003	TRUE
GB19373	5718	4006	0.513348199	-0.35116931	FALSE
GB19374	4	2	1	0.135482492	FALSE
GB19375	34	15	1.180572246	0.316054737	FALSE
GB19376	167	82	1.026152288	0.16163478	FALSE
GB19377	466	206	1.177685617	0.313168109	FALSE
GB19378	4099	1904	1.106238511	0.241721003	FALSE
GB19379	1151	733	0.65100273	-0.213514778	FALSE
GB19380	37911	24271	0.643382961	-0.221134547	FALSE
GB19381	398	221	0.848722061	-0.015795447	FALSE
GB19382	3	0	NA	NA	FALSE
GB19383	859	430	0.998321472	0.133803963	FALSE
GB19384	870	455	0.935148856	0.070631347	FALSE
GB19385	468	294	0.670692375	-0.193825134	FALSE
GB19386	1307	603	1.116029234	0.251511726	FALSE
GB19387	6219	3159	0.97721468	0.112697172	FALSE
GB19388	1955	1634	0.258760624	-0.605756884	TRUE
GB19389	140	69	1.02075856	0.156241052	FALSE
GB19390	1481	826	0.842357954	-0.022159554	FALSE
GB19391	18	3	2.584962501	1.720444992	TRUE
GB19392	1	3	-1.584962501	-2.449480009	TRUE
GB19393	184	85	1.11417102	0.249653512	FALSE
GB19394	2139	1152	0.892795766	0.028278257	FALSE
GB19395	640	449	0.51135646	-0.353161048	FALSE
GB19396	955	492	0.956842418	0.092324909	FALSE
GB19397	2270	1261	0.848124022	-0.016393486	FALSE
GB19398	299	179	0.740185897	-0.124331611	FALSE
GB19399	3565	2885	0.305330758	-0.559186751	TRUE
GB19400	3	1	1.584962501	0.720444992	TRUE
GB19401	360	148	1.282399731	0.417882222	FALSE
GB19402	389	246	0.66111184	-0.203405669	FALSE
GB19403	1861	948	0.973119091	0.108601583	FALSE
GB19404	785	425	0.885229813	0.020712304	FALSE
GB19405	14549	7027	1.049939192	0.185421683	FALSE
GB19406	2342	1420	0.721850146	-0.142667362	FALSE
GB19407	1495	1037	0.52772959	-0.336787918	FALSE
GB19408	324	187	0.792955543	-0.071561965	FALSE
GB19409	480	223	1.105990696	0.241473187	FALSE
GB19410	554	418	0.406383034	-0.458134474	FALSE
GB19411	1421	736	0.949128883	0.084611375	FALSE
GB19412	972	467	1.057533764	0.193016256	FALSE
GB19413	11	3	1.874469118	1.00995161	TRUE

GB19414	40	42	-0.070389328	-0.934906836	TRUE
GB19415	NA	NA	NA	NA	FALSE
GB19416	1279	662	0.950113142	0.085595634	FALSE
GB19417	1	1	0	-0.864517508	TRUE
GB19418	67	41	0.708537186	-0.155980322	FALSE
GB19419	5	3	0.736965594	-0.127551914	FALSE
GB19420	302	189	0.676162315	-0.188355193	FALSE
GB19421	39	24	0.700439718	-0.16407779	FALSE
GB19422	1386	779	0.831232024	-0.033285484	FALSE
GB19423	2353	1212	0.957111622	0.092594113	FALSE
GB19424	601	504	0.253941257	-0.610576251	TRUE
GB19425	4571	2991	0.611881908	-0.2526356	FALSE
GB19426	48	15	1.678071905	0.813554397	TRUE
GB19427	797	413	0.948437943	0.083920434	FALSE
GB19428	2293	1195	0.940225737	0.075708228	FALSE
GB19429	9786	5873	0.736621645	-0.127895863	FALSE
GB19430	892	371	1.265624523	0.401107015	FALSE
GB19431	796	564	0.497073268	-0.36744424	FALSE
GB19432	93	54	0.784271309	-0.080246199	FALSE
GB19433	608	380	0.678071905	-0.186445603	FALSE
GB19434	422	184	1.197537233	0.333019724	FALSE
GB19435	18688	11970	0.642689027	-0.221828481	FALSE
GB19436	1	0	NA	NA	FALSE
GB19437	1122	592	0.922403595	0.057886087	FALSE
GB19438	29	17	0.770518154	-0.093999354	FALSE
GB19439	149	73	1.029343962	0.164826453	FALSE
GB19440	238	143	0.734946427	-0.129571082	FALSE
GB19441	14	10	0.485426827	-0.379090681	FALSE
GB19442	12780	9400	0.443155174	-0.421362334	FALSE
GB19443	2525	1482	0.76873794	-0.095779568	FALSE
GB19444	11785	5896	0.999143329	0.134625821	FALSE
GB19445	33	18	0.874469118	0.00995161	FALSE
GB19446	1084	703	0.624768162	-0.239749346	FALSE
GB19447	1	0	NA	NA	FALSE
GB19448	162	67	1.273760812	0.409243304	FALSE
GB19449	3	11	-1.874469118	-2.738986626	TRUE
GB19450	619	334	0.890091307	0.025573798	FALSE
GB19451	4	1	2	1.135482492	TRUE
GB19452	210	109	0.946061193	0.081543685	FALSE
GB19453	994	597	0.73551492	-0.129002588	FALSE
GB19454	NA	NA	NA	NA	FALSE
GB19455	36	25	0.526068812	-0.338448697	FALSE
GB19456	4575	2877	0.669206522	-0.195310986	FALSE
GB19457	1624	810	1.003557819	0.139040311	FALSE

GB19458	0	5	NA	NA	FALSE
GB19459	64	36	0.830074999	-0.03444251	FALSE
GB19460	126801	85088	0.575538536	-0.288978973	FALSE
GB19461	751	420	0.83842358	-0.026093928	FALSE
GB19462	875	454	0.946590719	0.082073211	FALSE
GB19463	2094	1156	0.857120044	-0.007397464	FALSE
GB19464	35	19	0.881355504	0.016837995	FALSE
GB19465	17393	12445	0.482940564	-0.381576944	FALSE
GB19466	6	1	2.584962501	1.720444992	TRUE
GB19467	1031	442	1.221926058	0.35740855	FALSE
GB19468	148	40	1.887525271	1.023007762	TRUE
GB19469	2	4	-1	-1.864517508	TRUE
GB19470	83	57	0.542149417	-0.322368091	FALSE
GB19471	1513	686	1.141131506	0.276613998	FALSE
GB19472	376	217	0.793037619	-0.071479889	FALSE
GB19473	1223	717	0.77037938	-0.094138129	FALSE
GB19474	43	24	0.841302254	-0.023215254	FALSE
GB19475	402	183	1.135351853	0.270834345	FALSE
GB19476	1102	710	0.634233294	-0.230284214	FALSE
GB19477	307	179	0.778279068	-0.08623844	FALSE
GB19478	1201	463	1.375152052	0.510634544	TRUE
GB19479	1	1	0	-0.864517508	TRUE
GB19480	1675	772	1.117488343	0.252970835	FALSE
GB19481	2842	1435	0.985855818	0.121338309	FALSE
GB19482	8	6	0.415037499	-0.449480009	FALSE
GB19483	373	206	0.856531293	-0.007986215	FALSE
GB19484	2401	1500	0.678672903	-0.185844605	FALSE
GB19485	1329	640	1.054197294	0.189679786	FALSE
GB19486	272	158	0.783682093	-0.080835415	FALSE
GB19487	15426	10071	0.615157073	-0.249360435	FALSE
GB19488	9	4	1.169925001	0.305407493	FALSE
GB19489	188	114	0.721698838	-0.142818671	FALSE
GB19490	544	336	0.695145418	-0.16937209	FALSE
GB19491	2355	1118	1.074806872	0.210289363	FALSE
GB19492	486	270	0.847996907	-0.016520602	FALSE
GB19493	8	5	0.678071905	-0.186445603	FALSE
GB19494	20283	14010	0.533814097	-0.330703411	FALSE
GB19495	1619	910	0.831164535	-0.033352973	FALSE
GB19496	NA	NA	NA	NA	FALSE
GB19497	229	84	1.446886365	0.582368857	TRUE
GB19498	2242	1215	0.883829964	0.019312456	FALSE
GB19499	626	271	1.207869806	0.343352297	FALSE
GB19500	1706	963	0.825009943	-0.039507565	FALSE
GB19501	11036	4461	1.306778309	0.4422608	FALSE

GB19502	1096	575	0.930613937	0.066096429	FALSE
GB19503	4914	2122	1.211473201	0.346955693	FALSE
GB19504	327	179	0.869331048	0.00481354	FALSE
GB19505	1	1	0	-0.864517508	TRUE
GB19506	403	277	0.540893862	-0.323623646	FALSE
GB19507	98	83	0.239670413	-0.624847096	TRUE
GB19508	352	181	0.959585732	0.095068223	FALSE
GB19509	3000	1421	1.078055946	0.213538438	FALSE
GB19510	1177	622	0.920127835	0.055610327	FALSE
GB19511	1	0	NA	NA	FALSE
GB19512	709	425	0.738322786	-0.126194722	FALSE
GB19513	1659	903	0.877515993	0.012998485	FALSE
GB19514	766	504	0.603920658	-0.26059685	FALSE
GB19515	370	193	0.938924423	0.074406915	FALSE
GB19516	NA	NA	NA	NA	FALSE
GB19517	549	344	0.674397584	-0.190119924	FALSE
GB19518	326	185	0.817346694	-0.047170815	FALSE
GB19519	10	7	0.514573173	-0.349944335	FALSE
GB19520	163	83	0.973688723	0.109171215	FALSE
GB19521	402	236	0.768408642	-0.096108867	FALSE
GB19522	3490	2432	0.521083808	-0.343433701	FALSE
GB19523	689	346	0.993731945	0.129214437	FALSE
GB19524	2127	764	1.47717549	0.612657982	TRUE
GB19525	1437	734	0.969208094	0.104690585	FALSE
GB19526	145	71	1.030161971	0.165644462	FALSE
GB19527	704	282	1.319880266	0.455362758	FALSE
GB19528	709	374	0.922747357	0.058229849	FALSE
GB19529	3470	1697	1.031949098	0.16743159	FALSE
GB19530	85	42	1.017073513	0.152556005	FALSE
GB19531	5902	3974	0.570612048	-0.29390546	FALSE
GB19532	4	0	NA	NA	FALSE
GB19533	137	75	0.869213392	0.004695884	FALSE
GB19534	284	142	1	0.135482492	FALSE
GB19535	12	5	1.263034406	0.398516898	FALSE
GB19536	380	228	0.736965594	-0.127551914	FALSE
GB19537	NA	NA	NA	NA	FALSE
GB19538	659	397	0.731139458	-0.13337805	FALSE
GB19539	452	227	0.993630475	0.129112967	FALSE
GB19540	NA	NA	NA	NA	FALSE
GB19541	2132	1176	0.858319378	-0.00619813	FALSE
GB19542	385	195	0.981384322	0.116866814	FALSE
GB19543	881	481	0.873105125	0.008587617	FALSE
GB19544	246	188	0.387925654	-0.476591855	FALSE
GB19545	0	1	NA	NA	FALSE

GB19546	238	125	0.929033479	0.06451597	FALSE
GB19547	358	188	0.929226926	0.064709417	FALSE
GB19548	949	501	0.921597484	0.057079975	FALSE
GB19549	129	98	0.396517411	-0.468000097	FALSE
GB19550	20	20	0	-0.864517508	TRUE
GB19551	843	466	0.855202676	-0.009314832	FALSE
GB19552	288	111	1.375509135	0.510991627	TRUE
GB19553	6550	4235	0.629132937	-0.235384571	FALSE
GB19554	2	6	-1.584962501	-2.449480009	TRUE
GB19555	2	2	0	-0.864517508	TRUE
GB19556	555	342	0.698491446	-0.166026062	FALSE
GB19557	1135	551	1.042568074	0.178050565	FALSE
GB19558	16948	11675	0.537692484	-0.326825024	FALSE
GB19559	334	173	0.949076065	0.084558557	FALSE
GB19560	1054	631	0.740162957	-0.124354552	FALSE
GB19561	91	35	1.378511623	0.513994115	TRUE
GB19562	381	155	1.297522782	0.433005274	FALSE
GB19563	288	148	0.960471636	0.095954127	FALSE
GB19564	623	230	1.437598302	0.573080794	TRUE
GB19565	1959	1228	0.673806837	-0.190710671	FALSE
GB19566	404	225	0.844430292	-0.020087217	FALSE
GB19567	424	268	0.661831264	-0.202686244	FALSE
GB19568	545	270	1.013296823	0.148779314	FALSE
GB19569	1875	1070	0.809279799	-0.055237709	FALSE
GB19570	2388	1591	0.585869001	-0.278648507	FALSE
GB19571	163	115	0.503238103	-0.361279405	FALSE
GB19572	1172	632	0.890976106	0.026458598	FALSE
GB19573	1802	857	1.072231902	0.207714393	FALSE
GB19574	2651	1397	0.924204649	0.059687141	FALSE
GB19575	197	86	1.195787065	0.331269556	FALSE
GB19576	6	5	0.263034406	-0.601483102	TRUE
GB19577	8	1	3	2.135482492	TRUE
GB19578	10588	6121	0.790590827	-0.073926682	FALSE
GB19579	534	256	1.060695932	0.196178423	FALSE
GB19580	1151	658	0.806728344	-0.057789164	FALSE
GB19581	490	278	0.817696866	-0.046820642	FALSE
GB19582	14648	7682	0.931149827	0.066632319	FALSE
GB19583	376	245	0.617950913	-0.246566596	FALSE
GB19584	1789	1098	0.704275333	-0.160242175	FALSE
GB19585	33673	16771	1.005623545	0.141106037	FALSE
GB19586	3	7	-1.222392421	-2.08690993	TRUE
GB19587	3785	1382	1.453535684	0.589018176	TRUE
GB19588	724	363	0.996020149	0.131502641	FALSE
GB19589	372	234	0.668794092	-0.195723417	FALSE

GB19590	376	217	0.793037619	-0.071479889	FALSE
GB19591	725	359	1.013997151	0.149479643	FALSE
GB19592	1776	765	1.215099929	0.35058242	FALSE
GB19593	1712	734	1.221830734	0.357313225	FALSE
GB19594	88	54	0.704544116	-0.159973392	FALSE
GB19595	4	1	2	1.135482492	TRUE
GB19596	240	193	0.314433558	-0.55008395	TRUE
GB19597	29	16	0.857980995	-0.006536513	FALSE
GB19598	188	131	0.52116585	-0.343351658	FALSE
GB19599	379	253	0.583060463	-0.281457045	FALSE
GB19600	200	97	1.043943348	0.179425839	FALSE
GB19601	584	322	0.858907681	-0.005609828	FALSE
GB19602	1382	752	0.877953049	0.01343554	FALSE
GB19603	2846	1785	0.673011588	-0.191505921	FALSE
GB19604	3968	2064	0.942969055	0.078451547	FALSE
GB19605	215	83	1.373153418	0.50863591	TRUE
GB19606	14282	7516	0.926161051	0.061643543	FALSE
GB19607	1021	482	1.082877815	0.218360306	FALSE
GB19608	NA	NA	NA	NA	FALSE
GB19609	324	145	1.159940913	0.295423405	FALSE
GB19610	537	340	0.659387342	-0.205130166	FALSE
GB19611	437	292	0.581664911	-0.282852598	FALSE
GB19612	10010	4332	1.208336826	0.343819318	FALSE
GB19613	50	26	0.943416472	0.078898963	FALSE
GB19614	1401	723	0.954389403	0.089871895	FALSE
GB19615	44	24	0.874469118	0.00995161	FALSE
GB19616	351	245	0.518689281	-0.345828227	FALSE
GB19617	1121	719	0.640722602	-0.223794906	FALSE
GB19618	506	384	0.398031074	-0.466486434	FALSE
GB19619	366	224	0.708344916	-0.156172592	FALSE
GB19620	190	135	0.493040011	-0.371477497	FALSE
GB19621	NA	NA	NA	NA	FALSE
GB19622	NA	NA	NA	NA	FALSE
GB19623	3371	1577	1.095993967	0.231476459	FALSE
GB19624	130	74	0.812914447	-0.051603061	FALSE
GB19625	339	207	0.711654506	-0.152863003	FALSE
GB19626	11	3	1.874469118	1.00995161	TRUE
GB19627	485	313	0.63182209	-0.232695418	FALSE
GB19628	13	7	0.893084796	0.028567288	FALSE
GB19629	24	11	1.125530882	0.261013374	FALSE
GB19630	490	313	0.646619092	-0.217898416	FALSE
GB19631	NA	NA	NA	NA	FALSE
GB19632	670	336	0.995699863	0.131182354	FALSE
GB19633	6272	3332	0.912537159	0.04801965	FALSE

GB19634	8855	4293	1.044506134	0.179988626	FALSE
GB19635	1386	694	0.99791969	0.133402181	FALSE
GB19636	914	484	0.917187118	0.052669609	FALSE
GB19637	2379	1202	0.984918376	0.120400867	FALSE
GB19638	358	141	1.344264425	0.479746917	FALSE
GB19639	1640	1238	0.4056845	-0.458833008	FALSE
GB19640	85	51	0.736965594	-0.127551914	FALSE
GB19641	NA	NA	NA	NA	FALSE
GB19642	584	313	0.899805712	0.035288204	FALSE
GB19643	5862	2090	1.487890026	0.623372517	TRUE
GB19644	2559	1478	0.791933878	-0.07258363	FALSE
GB19645	149	82	0.861616516	-0.002900992	FALSE
GB19646	419	304	0.46287892	-0.401638588	FALSE
GB19647	1163	556	1.064694309	0.2001768	FALSE
GB19648	NA	NA	NA	NA	FALSE
GB19649	508	275	0.885396878	0.02087937	FALSE
GB19650	638	334	0.933708321	0.069190813	FALSE
GB19651	1119	549	1.027331982	0.162814474	FALSE
GB19652	449	283	0.665913392	-0.198604116	FALSE
GB19653	128	72	0.830074999	-0.03444251	FALSE
GB19654	433	247	0.809855983	-0.054661525	FALSE
GB19655	202	77	1.391424942	0.526907434	TRUE
GB19656	1458	652	1.16104685	0.296529342	FALSE
GB19657	8	3	1.415037499	0.550519991	TRUE
GB19658	898	511	0.813392154	-0.051125355	FALSE
GB19659	1	3	-1.584962501	-2.449480009	TRUE
GB19660	535	264	1.019000962	0.154483454	FALSE
GB19661	280	164	0.771731012	-0.092786496	FALSE
GB19662	1268	637	0.993189468	0.12867196	FALSE
GB19663	36	21	0.777607579	-0.08690993	FALSE
GB19664	223	93	1.261741089	0.39722358	FALSE
GB19665	241	142	0.763142217	-0.101375292	FALSE
GB19666	68	33	1.043068722	0.178551214	FALSE
GB19667	44	17	1.371968777	0.507451269	TRUE
GB19668	7	7	0	-0.864517508	TRUE
GB19669	1344	627	1.09999579	0.235478282	FALSE
GB19670	1071	479	1.160860919	0.296343411	FALSE
GB19671	520	318	0.709484858	-0.155032651	FALSE
GB19672	68	38	0.839535328	-0.024982181	FALSE
GB19673	94	46	1.031026896	0.166509387	FALSE
GB19674	1153	580	0.991267708	0.126750199	FALSE
GB19675	140	66	1.084888898	0.220371389	FALSE
GB19676	10936	5966	0.874249265	0.009731757	FALSE
GB19677	1468	721	1.025780804	0.161263295	FALSE

GB19678	385	201	0.937662944	0.073145436	FALSE
GB19679	44	19	1.211504105	0.346986597	FALSE
GB19680	1090	682	0.676484491	-0.188033018	FALSE
GB19681	648	341	0.926222074	0.061704566	FALSE
GB19682	550	310	0.827163403	-0.037354105	FALSE
GB19683	2235	1147	0.96240944	0.097891932	FALSE
GB19684	16	2	3	2.135482492	TRUE
GB19685	5	8	-0.678071905	-1.542589413	TRUE
GB19686	1375	729	0.915440899	0.050923391	FALSE
GB19687	1054	514	1.036034602	0.171517094	FALSE
GB19688	144	48	1.584962501	0.720444992	TRUE
GB19689	621	356	0.802716027	-0.061801481	FALSE
GB19690	410	201	1.028428408	0.1639109	FALSE
GB19691	0	1	NA	NA	FALSE
GB19692	542	273	0.9893919	0.124874392	FALSE
GB19693	1351	625	1.11209958	0.247582071	FALSE
GB19694	539	345	0.643688911	-0.220828597	FALSE
GB19695	NA	NA	NA	NA	FALSE
GB19696	14	4	1.807354922	0.942837414	TRUE
GB19697	3	0	NA	NA	FALSE
GB19698	414	221	0.905584398	0.04106689	FALSE
GB19699	8	6	0.415037499	-0.449480009	FALSE
GB19700	1	1	0	-0.864517508	TRUE
GB19701	3736	3070	0.283255799	-0.581261709	TRUE
GB19702	10	2	2.321928095	1.457410587	TRUE
GB19703	11	3	1.874469118	1.00995161	TRUE
GB19704	43	8	2.426264755	1.561747246	TRUE
GB19705	1529	897	0.769408516	-0.095108992	FALSE
GB19706	135	62	1.122619287	0.258101778	FALSE
GB19707	NA	NA	NA	NA	FALSE
GB19708	823	434	0.923197388	0.05867988	FALSE
GB19709	199	68	1.549161779	0.684644271	TRUE
GB19710	280	160	0.807354922	-0.057162586	FALSE
GB19711	136	69	0.978938384	0.114420876	FALSE
GB19712	817	518	0.65738398	-0.207133528	FALSE
GB19713	307	111	1.467678979	0.603161471	TRUE
GB19714	1651	982	0.749545191	-0.114972318	FALSE
GB19715	NA	NA	NA	NA	FALSE
GB19716	36	22	0.710493383	-0.154024126	FALSE
GB19717	4	1	2	1.135482492	TRUE
GB19718	79	43	0.877515993	0.012998485	FALSE
GB19719	168	77	1.125530882	0.261013374	FALSE
GB19720	434	303	0.518377249	-0.346140259	FALSE
GB19721	316	185	0.772399288	-0.092118221	FALSE

GB19722	842	480	0.810785827	-0.053731681	FALSE
GB19723	170	71	1.259643817	0.395126308	FALSE
GB19724	27113	15278	0.827529057	-0.036988452	FALSE
GB19725	909	622	0.547365714	-0.317151794	FALSE
GB19726	7	12	-0.777607579	-1.642125087	TRUE
GB19727	472	304	0.634715536	-0.229801972	FALSE
GB19728	124	48	1.36923381	0.504716301	TRUE
GB19729	16	7	1.192645078	0.32812757	FALSE
GB19730	433	336	0.365905792	-0.498611716	FALSE
GB19731	1952	1005	0.957757551	0.093240043	FALSE
GB19732	0	1	NA	NA	FALSE
GB19733	276	197	0.486472637	-0.378044871	FALSE
GB19734	534	314	0.766075183	-0.098442326	FALSE
GB19735	2066	1014	1.026782602	0.162265094	FALSE
GB19736	700	313	1.161192265	0.296674757	FALSE
GB19737	4367	2303	0.92312812	0.058610612	FALSE
GB19738	465	295	0.656515762	-0.208001747	FALSE
GB19739	1858	922	1.010911846	0.146394338	FALSE
GB19740	8569	4202	1.02805069	0.163533182	FALSE
GB19741	1486	858	0.792384563	-0.072132945	FALSE
GB19742	28092	11067	1.343895145	0.479377637	FALSE
GB19743	177	82	1.110053545	0.245536037	FALSE
GB19744	NA	NA	NA	NA	FALSE
GB19745	171244	75395	1.183512684	0.318995176	FALSE
GB19746	1197	575	1.057789291	0.193271783	FALSE
GB19747	223	120	0.894009304	0.029491796	FALSE
GB19748	10	6	0.736965594	-0.127551914	FALSE
GB19749	492	256	0.942514505	0.077996997	FALSE
GB19750	2143	1191	0.847458437	-0.017059072	FALSE
GB19751	810	424	0.933857643	0.069340135	FALSE
GB19752	1582	809	0.967537992	0.103020484	FALSE
GB19753	3571	2360	0.597541274	-0.266976234	FALSE
GB19754	1878	646	1.539590993	0.675073485	TRUE
GB19755	5007	3569	0.488426554	-0.376090954	FALSE
GB19756	584	404	0.531613076	-0.332904432	FALSE
GB19757	21	9	1.222392421	0.357874913	FALSE
GB19758	554	343	0.6916774	-0.172840108	FALSE
GB19759	662	380	0.800831798	-0.06368571	FALSE
GB19760	NA	NA	NA	NA	FALSE
GB19761	10	0	NA	NA	FALSE
GB19762	620	349	0.829041179	-0.035476329	FALSE
GB19763	0	2	NA	NA	FALSE
GB19764	373	262	0.509608819	-0.35490869	FALSE
GB19765	259	114	1.183918274	0.319400765	FALSE

GB19766	1538	573	1.424448459	0.559930951	TRUE
GB19767	2176	1197	0.862255404	-0.002262104	FALSE
GB19768	122	65	0.908369525	0.043852016	FALSE
GB19769	715	432	0.72691193	-0.137605579	FALSE
GB19770	1206	630	0.936806174	0.072288665	FALSE
GB19771	306	160	0.935459748	0.070942239	FALSE
GB19772	1	0	NA	NA	FALSE
GB19773	945	559	0.757466046	-0.107051462	FALSE
GB19774	37	12	1.624490865	0.759973357	TRUE
GB19775	26	17	0.612976877	-0.251540631	FALSE
GB19776	0	1	NA	NA	FALSE
GB19777	5316	3273	0.699727502	-0.164790006	FALSE
GB19778	787	487	0.692441863	-0.172075645	FALSE
GB19779	1801	905	0.992808484	0.128290976	FALSE
GB19780	NA	NA	NA	NA	FALSE
GB19781	733	401	0.870210962	0.005693453	FALSE
GB19782	853	559	0.609697458	-0.25482005	FALSE
GB19783	NA	NA	NA	NA	FALSE
GB19784	237	156	0.60334103	-0.261176478	FALSE
GB19785	55	30	0.874469118	0.00995161	FALSE
GB19786	353	179	0.979708596	0.115191088	FALSE
GB19787	495	271	0.869135674	0.004618165	FALSE
GB19788	1944	919	1.080891452	0.216373944	FALSE
GB19789	1669	1037	0.686568061	-0.177949448	FALSE
GB19790	230	134	0.77940086	-0.085116648	FALSE
GB19791	1539	890	0.79011599	-0.074401518	FALSE
GB19792	584	223	1.388924659	0.524407151	TRUE
GB19793	14113	6741	1.065990164	0.201472655	FALSE
GB19794	1725	1044	0.72447465	-0.140042858	FALSE
GB19795	476	364	0.387023123	-0.477494385	FALSE
GB19796	496	353	0.490671937	-0.373845571	FALSE
GB19797	15682	8169	0.940878177	0.076360668	FALSE
GB19798	1937	1034	0.905587768	0.04107026	FALSE
GB19799	8962	4806	0.898984078	0.03446657	FALSE
GB19800	324	150	1.111031312	0.246513804	FALSE
GB19801	929	516	0.848307531	-0.016209977	FALSE
GB19802	0	1	NA	NA	FALSE
GB19803	NA	NA	NA	NA	FALSE
GB19804	9	7	0.362570079	-0.501947429	TRUE
GB19805	1725	893	0.949864281	0.085346773	FALSE
GB19806	975	395	1.303549566	0.439032057	FALSE
GB19807	456	322	0.501973136	-0.362544372	FALSE
GB19808	1158	682	0.763791609	-0.100725899	FALSE
GB19809	1	0	NA	NA	FALSE

GB19810	0	1	NA	NA	FALSE
GB19811	4466	1934	1.207395456	0.342877948	FALSE
GB19812	489	303	0.690516671	-0.174000837	FALSE
GB19813	NA	NA	NA	NA	FALSE
GB19814	220	117	0.910994994	0.046477486	FALSE
GB19815	1238	706	0.810271226	-0.054246282	FALSE
GB19816	626	319	0.972606233	0.108088725	FALSE
GB19817	103	66	0.642106408	-0.222411101	FALSE
GB19818	834	392	1.089193729	0.224676221	FALSE
GB19819	54600	21043	1.375560554	0.511043045	TRUE
GB19820	31907	16545	0.947477676	0.082960168	FALSE
GB19821	608	362	0.748081626	-0.116435882	FALSE
GB19822	1251	777	0.687095286	-0.177422223	FALSE
GB19823	783	375	1.062121712	0.197604204	FALSE
GB19824	1189	656	0.857980995	-0.006536513	FALSE
GB19825	10	8	0.321928095	-0.542589413	TRUE
GB19826	615	258	1.253215345	0.388697836	FALSE
GB19827	167	74	1.174250927	0.309733419	FALSE
GB19828	5281	2600	1.022299518	0.15778201	FALSE
GB19829	276	163	0.759796303	-0.104721206	FALSE
GB19830	364	164	1.150242636	0.285725127	FALSE
GB19831	2900	1353	1.099891061	0.235373553	FALSE
GB19832	72057	35684	1.013861245	0.149343737	FALSE
GB19833	1	1	0	-0.864517508	TRUE
GB19834	120	56	1.099535674	0.235018165	FALSE
GB19835	1048	489	1.099732347	0.235214838	FALSE
GB19836	10	7	0.514573173	-0.349944335	FALSE
GB19837	3578	1810	0.98316369	0.118646182	FALSE
GB19838	814	444	0.874469118	0.00995161	FALSE
GB19839	1	2	-1	-1.864517508	TRUE
GB19840	1037	628	0.72357943	-0.140938078	FALSE
GB19841	279	154	0.857334771	-0.007182737	FALSE
GB19842	594	438	0.439532061	-0.424985447	FALSE
GB19843	208	104	1	0.135482492	FALSE
GB19844	215	87	1.305249354	0.440731845	FALSE
GB19845	1417	913	0.634152993	-0.230364515	FALSE
GB19846	51043	32613	0.646265967	-0.218251541	FALSE
GB19847	706	343	1.041459607	0.176942099	FALSE
GB19848	0	1	NA	NA	FALSE
GB19849	83	53	0.647118977	-0.217398532	FALSE
GB19850	0	1	NA	NA	FALSE
GB19851	2	2	0	-0.864517508	TRUE
GB19852	80	51	0.649502753	-0.215014755	FALSE
GB19853	243	114	1.091922489	0.227404981	FALSE

GB19854	629	419	0.586109773	-0.278407735	FALSE
GB19855	128	99	0.37064338	-0.493874128	FALSE
GB19856	101	58	0.800230488	-0.064287021	FALSE
GB19857	62109	37356	0.733462357	-0.131055151	FALSE
GB19858	17	7	1.280107919	0.415590411	FALSE
GB19859	1	1	0	-0.864517508	TRUE
GB19860	12151	9407	0.36926844	-0.495249068	FALSE
GB19861	425	257	0.725694482	-0.138823026	FALSE
GB19862	251	107	1.230076568	0.365559059	FALSE
GB19863	1480	925	0.678071905	-0.186445603	FALSE
GB19864	520	287	0.857460886	-0.007056622	FALSE
GB19865	230	104	1.145050333	0.280532824	FALSE
GB19866	1	1	0	-0.864517508	TRUE
GB19867	27	24	0.169925001	-0.694592507	TRUE
GB19868	897	503	0.834549585	-0.029967923	FALSE
GB19869	57	33	0.788495895	-0.076021614	FALSE
GB19870	1626	721	1.173256093	0.308738585	FALSE
GB19871	240	143	0.747019259	-0.117498249	FALSE
GB19872	17	13	0.387023123	-0.477494385	FALSE
GB19873	5670	3164	0.841599135	-0.022918373	FALSE
GB19874	NA	NA	NA	NA	FALSE
GB19875	1631	929	0.81200628	-0.052511228	FALSE
GB19876	NA	NA	NA	NA	FALSE
GB19877	9	4	1.169925001	0.305407493	FALSE
GB19878	3760	2109	0.834173567	-0.030343942	FALSE
GB19879	2250	1161	0.954557029	0.090039521	FALSE
GB19880	6215	3847	0.692020562	-0.172496947	FALSE
GB19881	4674	2362	0.984648769	0.120131261	FALSE
GB19882	17	4	2.087462841	1.222945333	TRUE
GB19883	22	21	0.067114196	-0.797403312	TRUE
GB19884	966	418	1.208520247	0.344002738	FALSE
GB19885	28763	21816	0.398827552	-0.465689956	FALSE
GB19886	232	112	1.050626073	0.186108565	FALSE
GB19887	184	96	0.938599455	0.074081947	FALSE
GB19888	4772	3628	0.395419587	-0.469097921	FALSE
GB19889	2827	1272	1.152173213	0.287655704	FALSE
GB19890	NA	NA	NA	NA	FALSE
GB19891	2800	1335	1.068587085	0.204069577	FALSE
GB19892	122	80	0.608809243	-0.255708266	FALSE
GB19893	2	0	NA	NA	FALSE
GB19894	NA	NA	NA	NA	FALSE
GB19895	75	31	1.27462238	0.410104872	FALSE
GB19896	70	19	1.881355504	1.016837995	TRUE
GB19897	2969	1583	0.907315838	0.04279833	FALSE

GB19898	1041	612	0.766366511	-0.098150998	FALSE
GB19899	330	215	0.618129365	-0.246388144	FALSE
GB19900	64	43	0.573735245	-0.290782263	FALSE
GB19901	838	362	1.210960547	0.346443038	FALSE
GB19902	252	158	0.673499175	-0.191018333	FALSE
GB19903	588	384	0.614709844	-0.249807664	FALSE
GB19904	277	132	1.069348047	0.204830538	FALSE
GB19905	146	61	1.259087221	0.394569713	FALSE
GB19906	25	18	0.473931188	-0.39058632	FALSE
GB19907	266	165	0.688960221	-0.175557287	FALSE
GB19908	346	231	0.582879186	-0.281638322	FALSE
GB19909	654	352	0.893715207	0.029197699	FALSE
GB19910	711	245	1.537067811	0.672550302	TRUE
GB19911	0	1	NA	NA	FALSE
GB19912	831	435	0.933833076	0.069315568	FALSE
GB19913	183	72	1.345774837	0.481257329	FALSE
GB19914	364	207	0.814307683	-0.050209826	FALSE
GB19915	657	386	0.767292523	-0.097224985	FALSE
GB19916	5158	2517	1.035106557	0.170589048	FALSE
GB19917	311	175	0.829559658	-0.03495785	FALSE
GB19918	253	169	0.582114138	-0.28240337	FALSE
GB19919	542	176	1.622717423	0.758199914	TRUE
GB19920	1192	716	0.735352743	-0.129164765	FALSE
GB19921	1014	652	0.637113783	-0.227403726	FALSE
GB19922	1045	659	0.665152572	-0.199364936	FALSE
GB19923	9216	4982	0.887415695	0.022898187	FALSE
GB19924	46	23	1	0.135482492	FALSE
GB19925	561	260	1.109489148	0.244971639	FALSE
GB19926	NA	NA	NA	NA	FALSE
GB19927	699	448	0.641793723	-0.222723785	FALSE
GB19928	1709	764	1.161507854	0.296990345	FALSE
GB19929	411	176	1.223562965	0.359045457	FALSE
GB19930	949	430	1.142071427	0.277553919	FALSE
GB19931	660	340	0.956931278	0.09241377	FALSE
GB19932	25	17	0.556393349	-0.30812416	FALSE
GB19933	317	145	1.12842994	0.263912432	FALSE
GB19934	45	28	0.684498174	-0.180019334	FALSE
GB19935	108642	67590	0.684700224	-0.179817284	FALSE
GB19936	385	302	0.350309896	-0.514207612	TRUE
GB19937	587	287	1.032309766	0.167792258	FALSE
GB19938	1688	993	0.765449281	-0.099068227	FALSE
GB19939	275	140	0.974004791	0.109487283	FALSE
GB19940	76	38	1	0.135482492	FALSE
GB19941	793	287	1.466270129	0.601752621	TRUE

GB19942	568	325	0.805451212	-0.059066297	FALSE
GB19943	356	116	1.617752436	0.753234928	TRUE
GB19944	1	1	0	-0.864517508	TRUE
GB19945	224	54	2.05246742	1.187949912	TRUE
GB19946	238	124	0.940621453	0.076103945	FALSE
GB19947	1	0	NA	NA	FALSE
GB19948	576	285	1.015106892	0.150589384	FALSE
GB19949	363	214	0.762358752	-0.102158757	FALSE
GB19950	262	140	0.904139985	0.039622476	FALSE
GB19951	15	10	0.584962501	-0.279555008	FALSE
GB19952	1899	1045	0.861736963	-0.002780545	FALSE
GB19953	69	42	0.716207034	-0.148310474	FALSE
GB19954	291	159	0.871992388	0.007474879	FALSE
GB19955	247	112	1.14101231	0.276494801	FALSE
GB19956	465	272	0.773624065	-0.090893444	FALSE
GB19957	1062	383	1.471367469	0.606849961	TRUE
GB19958	148	79	0.905672617	0.041155109	FALSE
GB19959	201	70	1.521768674	0.657251166	TRUE
GB19960	837	535	0.645688731	-0.218828777	FALSE
GB19961	4784	1937	1.304393436	0.439875927	FALSE
GB19962	329	154	1.095157233	0.230639725	FALSE
GB19963	15	6	1.321928095	0.457410587	FALSE
GB19964	388	239	0.699046034	-0.165471474	FALSE
GB19965	171	117	0.547487795	-0.317029713	FALSE
GB19966	278	134	1.052851882	0.188334374	FALSE
GB19967	19717	8154	1.273860196	0.409342688	FALSE
GB19968	129	67	0.945138065	0.080620557	FALSE
GB19969	422	248	0.766902878	-0.09761463	FALSE
GB19970	962	607	0.664340378	-0.200177131	FALSE
GB19971	57	34	0.745427173	-0.119090335	FALSE
GB19972	274	149	0.878863562	0.014346054	FALSE
GB19973	705	375	0.910732662	0.046215154	FALSE
GB19974	20	8	1.321928095	0.457410587	FALSE
GB19975	2075	1422	0.545189872	-0.319327637	FALSE
GB19976	177	94	0.913016698	0.04849919	FALSE
GB19977	5	0	NA	NA	FALSE
GB19978	NA	NA	NA	NA	FALSE
GB19979	14	17	-0.280107919	-1.144625428	TRUE
GB19980	NA	NA	NA	NA	FALSE
GB19981	2583	1734	0.574943745	-0.289573763	FALSE
GB19982	30	15	1	0.135482492	FALSE
GB19983	343	269	0.350602404	-0.513915105	TRUE
GB19984	75	39	0.943416472	0.078898963	FALSE
GB19985	1	1	0	-0.864517508	TRUE

GB19986	620	330	0.909802191	0.045284683	FALSE
GB19987	18	5	1.847996907	0.983479398	TRUE
GB19988	15658	13567	0.206798208	-0.6577193	TRUE
GB19989	138	94	0.553935605	-0.310581903	FALSE
GB19990	1	1	0	-0.864517508	TRUE
GB19991	NA	NA	NA	NA	FALSE
GB19992	1233	694	0.829165232	-0.035352277	FALSE
GB19993	887	461	0.944167354	0.079649846	FALSE
GB19994	5328	2510	1.085906718	0.22138921	FALSE
GB19995	189	88	1.102810806	0.238293297	FALSE
GB19996	671	402	0.739117265	-0.125400243	FALSE
GB19997	876	472	0.89214401	0.027626502	FALSE
GB19998	610	268	1.186576242	0.322058734	FALSE
GB19999	1622	728	1.155763464	0.291245956	FALSE
GB20000	10003	5103	0.971015197	0.106497688	FALSE
GB20001	275	154	0.836501268	-0.028016241	FALSE
GB20002	12500	6818	0.87450759	0.009990082	FALSE
GB20003	1976	1324	0.577679825	-0.286837684	FALSE
GB20004	367	216	0.764748751	-0.099768758	FALSE
GB20005	36	16	1.169925001	0.305407493	FALSE
GB20006	466	232	1.00620515	0.141687641	FALSE
GB20007	363	264	0.459431619	-0.40508589	FALSE
GB20008	453	238	0.928549477	0.064031968	FALSE
GB20009	1	1	0	-0.864517508	TRUE
GB20010	0	2	NA	NA	FALSE
GB20011	810	445	0.864116572	-0.000400936	FALSE
GB20012	6085	3761	0.694140957	-0.170376552	FALSE
GB20013	3039	1904	0.674563196	-0.189954312	FALSE
GB20014	219	100	1.13093087	0.266413361	FALSE
GB20015	23	7	1.716207034	0.851689526	TRUE
GB20016	8	4	1	0.135482492	FALSE
GB20017	13012	7973	0.706648153	-0.157869355	FALSE
GB20018	503	292	0.784590031	-0.079927477	FALSE
GB20019	575	351	0.712090926	-0.152426583	FALSE
GB20020	196	300	-0.614108846	-1.478626355	TRUE
GB20021	34	14	1.280107919	0.415590411	FALSE
GB20022	633	263	1.2671427	0.402625192	FALSE
GB20023	508	296	0.779231321	-0.085286187	FALSE
GB20024	1457	838	0.797978728	-0.06653878	FALSE
GB20025	912	507	0.847048077	-0.017469431	FALSE
GB20026	NA	NA	NA	NA	FALSE
GB20027	4469	2394	0.900528892	0.036011384	FALSE
GB20028	140	82	0.771731012	-0.092786496	FALSE
GB20029	881	527	0.741339057	-0.123178451	FALSE

GB20030	347	224	0.631436931	-0.233080578	FALSE
GB20031	240	165	0.540568381	-0.323949127	FALSE
GB20032	13	3	2.115477217	1.250959709	TRUE
GB20033	617	363	0.765300941	-0.099216567	FALSE
GB20034	7210	4066	0.826389044	-0.038128464	FALSE
GB20035	283	158	0.840877495	-0.023640014	FALSE
GB20036	396	168	1.237039197	0.372521689	FALSE
GB20037	2112	1116	0.920272808	0.055755299	FALSE
GB20038	6625	3420	0.953924129	0.089406621	FALSE
GB20039	629	420	0.582670689	-0.281846819	FALSE
GB20040	520	324	0.68251781	-0.181999698	FALSE
GB20041	2655	1393	0.930516603	0.065999095	FALSE
GB20042	148	36	2.039528364	1.175010856	TRUE
GB20043	179	86	1.057551023	0.193033514	FALSE
GB20044	70	48	0.544320516	-0.320196992	FALSE
GB20045	330	202	0.708110731	-0.156406777	FALSE
GB20046	39	21	0.893084796	0.028567288	FALSE
GB20047	430	209	1.040833718	0.176316209	FALSE
GB20048	14	2	2.807354922	1.942837414	TRUE
GB20049	8	5	0.678071905	-0.186445603	FALSE
GB20050	883	571	0.628922692	-0.235594816	FALSE
GB20051	25	7	1.836501268	0.971983759	TRUE
GB20052	1	0	NA	NA	FALSE
GB20053	9	4	1.169925001	0.305407493	FALSE
GB20054	291	162	0.84502534	-0.019492168	FALSE
GB20055	10971	5855	0.905953957	0.041436448	FALSE
GB20056	121	86	0.492598483	-0.371919026	FALSE
GB20057	8	0	NA	NA	FALSE
GB20058	4242	2553	0.732551083	-0.131966425	FALSE
GB20059	314	193	0.702163712	-0.162353797	FALSE
GB20060	44	15	1.552541023	0.688023515	TRUE
GB20061	197	81	1.282201817	0.417684308	FALSE
GB20062	720	326	1.143124942	0.278607434	FALSE
GB20063	2432	1377	0.820614669	-0.043902839	FALSE
GB20064	NA	NA	NA	NA	FALSE
GB20065	308	196	0.652076697	-0.212440812	FALSE
GB20066	NA	NA	NA	NA	FALSE
GB20067	1683	954	0.818974005	-0.045543503	FALSE
GB20068	907	616	0.5581722	-0.306345308	FALSE
GB20069	1249	779	0.681078244	-0.183439265	FALSE
GB20070	78089	50607	0.625782384	-0.238735124	FALSE
GB20071	NA	NA	NA	NA	FALSE
GB20072	2036	938	1.118077734	0.253560225	FALSE
GB20073	NA	NA	NA	NA	FALSE

GB20074	117	52	1.169925001	0.305407493	FALSE
GB20075	18	4	2.169925001	1.305407493	TRUE
GB20076	411	200	1.039138394	0.174620886	FALSE
GB20077	1262	631	1	0.135482492	FALSE
GB20078	248	174	0.511252815	-0.353264694	FALSE
GB20079	5534	2624	1.076554924	0.212037416	FALSE
GB20080	6349	3883	0.709357671	-0.155159838	FALSE
GB20081	277	151	0.875337427	0.010819918	FALSE
GB20082	2505	1186	1.078706594	0.214189085	FALSE
GB20083	107	66	0.697072867	-0.167444641	FALSE
GB20084	645	323	0.997764996	0.133247487	FALSE
GB20085	593	329	0.849944521	-0.014572988	FALSE
GB20086	382	148	1.367975462	0.503457954	TRUE
GB20087	139	45	1.627087976	0.762570468	TRUE
GB20088	482	252	0.935609413	0.071091904	FALSE
GB20089	3690	2053	0.845887189	-0.018630319	FALSE
GB20090	1517	782	0.955980573	0.091463065	FALSE
GB20091	419	223	0.909906534	0.045389025	FALSE
GB20092	412	310	0.410376122	-0.454141386	FALSE
GB20093	1048	545	0.943310582	0.078793074	FALSE
GB20094	3139	1761	0.83391012	-0.030607389	FALSE
GB20095	24	12	1	0.135482492	FALSE
GB20096	306	181	0.757541956	-0.106975553	FALSE
GB20097	472	333	0.503264682	-0.361252826	FALSE
GB20098	11502	5928	0.95626739	0.091749882	FALSE
GB20099	NA	NA	NA	NA	FALSE
GB20100	5	4	0.321928095	-0.542589413	TRUE
GB20101	342	120	1.510961919	0.646444411	TRUE
GB20102	311	110	1.499411057	0.634893548	TRUE
GB20103	2712	1378	0.97678129	0.112263782	FALSE
GB20104	1969	708	1.475641846	0.611124337	TRUE
GB20105	118	32	1.882643049	1.018125541	TRUE
GB20106	21	0	NA	NA	FALSE
GB20107	725	375	0.9510904	0.086572891	FALSE
GB20108	801	379	1.079604394	0.215086886	FALSE
GB20109	798	405	0.978466838	0.11394933	FALSE
GB20110	825	398	1.051625689	0.18710818	FALSE
GB20111	2906	1876	0.631374875	-0.233142633	FALSE
GB20112	628	357	0.814840485	-0.049677023	FALSE
GB20113	6	3	1	0.135482492	FALSE
GB20114	12709	8705	0.545934314	-0.318583194	FALSE
GB20115	2673	1617	0.725140159	-0.13937735	FALSE
GB20116	368	201	0.872510265	0.007992757	FALSE
GB20117	104195	56696	0.877967189	0.013449681	FALSE

GB20118	850	363	1.227493293	0.362975785	FALSE
GB20119	393	261	0.590479506	-0.274038003	FALSE
GB20120	1247	604	1.045841011	0.181323502	FALSE
GB20121	2428	1282	0.92137216	0.056854651	FALSE
GB20122	1712	766	1.160266404	0.295748896	FALSE
GB20123	24	7	1.777607579	0.91309007	TRUE
GB20124	1	0	NA	NA	FALSE
GB20125	754	474	0.669677464	-0.194840044	FALSE
GB20126	389	231	0.751877304	-0.112640205	FALSE
GB20127	675	467	0.531464952	-0.333052556	FALSE
GB20128	1633	1078	0.599167613	-0.265349896	FALSE
GB20129	3641	2073	0.812614624	-0.051902884	FALSE
GB20130	246	103	1.256013978	0.39149647	FALSE
GB20131	897	655	0.453613078	-0.41090443	FALSE
GB20132	26	4	2.700439718	1.83592221	TRUE
GB20133	638	316	1.013631866	0.149114357	FALSE
GB20134	2	0	NA	NA	FALSE
GB20135	NA	NA	NA	NA	FALSE
GB20136	1481	801	0.886697493	0.022179985	FALSE
GB20137	833	518	0.685364398	-0.179153111	FALSE
GB20138	240	134	0.840801405	-0.023716103	FALSE
GB20139	17421	10658	0.708890702	-0.155626807	FALSE
GB20140	690	404	0.772241069	-0.092276439	FALSE
GB20141	144	67	1.103835811	0.239318303	FALSE
GB20142	3085	1576	0.969002955	0.104485446	FALSE
GB20143	1	0	NA	NA	FALSE
GB20144	3718	2061	0.851182265	-0.013335243	FALSE
GB20145	72	62	0.215728691	-0.648788817	TRUE
GB20146	22	9	1.289506617	0.424989109	FALSE
GB20147	NA	NA	NA	NA	FALSE
GB20148	760623	299029	1.34689616	0.482378651	FALSE
GB20149	1913	1078	0.827479696	-0.037037813	FALSE
GB20150	30	19	0.658963082	-0.205554426	FALSE
GB20151	658	339	0.956802311	0.092284802	FALSE
GB20152	17160	11198	0.615808468	-0.248709041	FALSE
GB20153	NA	NA	NA	NA	FALSE
GB20154	344	208	0.725825037	-0.138692472	FALSE
GB20155	18	17	0.08246216	-0.782055348	TRUE
GB20156	838	475	0.81902273	-0.045494778	FALSE
GB20157	1177	572	1.041027268	0.17650976	FALSE
GB30000	NA	NA	NA	NA	FALSE
GB30001	4086	1262	1.694977294	0.830459785	TRUE
GB30002	1	0	NA	NA	FALSE
GB30003	NA	NA	NA	NA	FALSE

GB30004	13	6	1.115477217	0.250959709	FALSE
GB30005	3	0	NA	NA	FALSE
GB30006	1	3	-1.584962501	-2.449480009	TRUE
GB30007	17	6	1.502500341	0.637982832	TRUE
GB30008	0	1	NA	NA	FALSE
GB30010	NA	NA	NA	NA	FALSE
GB30011	NA	NA	NA	NA	FALSE
GB30012	51	22	1.212993723	0.348476215	FALSE
GB30013	81	42	0.94753258	0.083015072	FALSE
GB30013_2	NA	NA	NA	NA	FALSE
GB30014	NA	NA	NA	NA	FALSE
GB30014_2	NA	NA	NA	NA	FALSE
GB30015	71	38	0.901819606	0.037302098	FALSE
GB30016	1	4	-2	-2.864517508	TRUE
GB30017	4	2	1	0.135482492	FALSE
GB30018	0	1	NA	NA	FALSE
GB30019	28	14	1	0.135482492	FALSE
GB30021	2	0	NA	NA	FALSE
GB30022	10	6	0.736965594	-0.127551914	FALSE
GB30023	18	3	2.584962501	1.720444992	TRUE
GB30024	89	35	1.346450414	0.481932906	FALSE
GB30025	3	4	-0.415037499	-1.279555008	TRUE
GB30026	2	0	NA	NA	FALSE
GB30027	10	4	1.321928095	0.457410587	FALSE
GB30028	33	13	1.343954401	0.479436893	FALSE
GB30029	1236	702	0.816135808	-0.048381701	FALSE
GB30031	0	1	NA	NA	FALSE
GB30031_2	26	12	1.115477217	0.250959709	FALSE
GB30031_3	6	5	0.263034406	-0.601483102	TRUE
GB30032	18	11	0.710493383	-0.154024126	FALSE
GB30033	1539	1118	0.461073043	-0.403444465	FALSE
GB30034	354	196	0.852895706	-0.011621802	FALSE
GB30035	10	1	3.321928095	2.457410587	TRUE
GB30036	NA	NA	NA	NA	FALSE
GB30037	NA	NA	NA	NA	FALSE
GB30038	NA	NA	NA	NA	FALSE
GB30039	1	0	NA	NA	FALSE
GB30040	1006	573	0.812023261	-0.052494247	FALSE
GB30041	NA	NA	NA	NA	FALSE
GB30042	NA	NA	NA	NA	FALSE
GB30043	127	88	0.529253068	-0.33526444	FALSE
GB30044	8	7	0.192645078	-0.67187243	TRUE
GB30045	NA	NA	NA	NA	FALSE
GB30046	NA	NA	NA	NA	FALSE

GB30047	NA	NA	NA	NA	FALSE
GB30049	360	279	0.367731785	-0.496785724	FALSE
GB30050	17	23	-0.436099115	-1.300616623	TRUE
GB30051	2	0	NA	NA	FALSE
GB30052	111	50	1.150559677	0.286042168	FALSE
GB30053	3	1	1.584962501	0.720444992	TRUE
GB30054	0	1	NA	NA	FALSE
GB30055	41	20	1.03562391	0.171106401	FALSE
GB30056	NA	NA	NA	NA	FALSE
GB30057	1978	1009	0.971116252	0.106598743	FALSE
GB30058	1	0	NA	NA	FALSE
GB30059	1	1	0	-0.864517508	TRUE
GB30060	0	2	NA	NA	FALSE
GB30062	45	28	0.684498174	-0.180019334	FALSE
GB30063	43	22	0.966833136	0.102315628	FALSE
GB30064	3	2	0.584962501	-0.279555008	FALSE
GB30065	NA	NA	NA	NA	FALSE
GB30066	173	49	1.819918384	0.955400875	TRUE
GB30067	354	142	1.317858431	0.453340922	FALSE
GB30068	NA	NA	NA	NA	FALSE
GB30069	NA	NA	NA	NA	FALSE
GB30070	NA	NA	NA	NA	FALSE
GB30070_2	NA	NA	NA	NA	FALSE
GB30071	1110	547	1.020946938	0.15642943	FALSE
GB30072	9	6	0.584962501	-0.279555008	FALSE
GB30073	149	96	0.63420602	-0.230311489	FALSE
GB30074	14	4	1.807354922	0.942837414	TRUE
GB30075	31	19	0.706268797	-0.158248711	FALSE
GB30076	6	6	0	-0.864517508	TRUE
GB30077	NA	NA	NA	NA	FALSE
GB30078	9	3	1.584962501	0.720444992	TRUE
GB30079	179	96	0.898853277	0.034335768	FALSE
GB30080	NA	NA	NA	NA	FALSE
GB30081	3	4	-0.415037499	-1.279555008	TRUE
GB30082	39	15	1.378511623	0.513994115	TRUE
GB30083	10	4	1.321928095	0.457410587	FALSE
GB30084	9	2	2.169925001	1.305407493	TRUE
GB30086	5	4	0.321928095	-0.542589413	TRUE
GB30087	376	184	1.031026896	0.166509387	FALSE
GB30088	83	66	0.330645312	-0.533872196	TRUE
GB30089	309	115	1.425972977	0.561455469	TRUE
GB30090	120	51	1.234465254	0.369947745	FALSE
GB30092	3	1	1.584962501	0.720444992	TRUE
GB30093	69	44	0.649092838	-0.21542467	FALSE

GB30094	2	0	NA	NA	FALSE
GB30095	NA	NA	NA	NA	FALSE
GB30096	NA	NA	NA	NA	FALSE
GB30097	471	300	0.650764559	-0.213752949	FALSE
GB30098	8	1	3	2.135482492	TRUE
GB30099	NA	NA	NA	NA	FALSE
GB30100	NA	NA	NA	NA	FALSE
GB30101	NA	NA	NA	NA	FALSE
GB30102	63	27	1.222392421	0.357874913	FALSE
GB30103	97	38	1.351985329	0.48746782	FALSE
GB30104	453	270	0.746551643	-0.117965865	FALSE
GB30105	NA	NA	NA	NA	FALSE
GB30106	NA	NA	NA	NA	FALSE
GB30107	37	22	0.750021747	-0.114495761	FALSE
GB30108	71	45	0.657894023	-0.206623485	FALSE
GB30109	78	36	1.115477217	0.250959709	FALSE
GB30110	39	15	1.378511623	0.513994115	TRUE
GB30111	2	1	1	0.135482492	FALSE
GB30112	NA	NA	NA	NA	FALSE
GB30113	NA	NA	NA	NA	FALSE
GB30114	NA	NA	NA	NA	FALSE
GB30115	0	1	NA	NA	FALSE
GB30116	NA	NA	NA	NA	FALSE
GB30117	NA	NA	NA	NA	FALSE
GB30118	NA	NA	NA	NA	FALSE
GB30119	NA	NA	NA	NA	FALSE
GB30120	NA	NA	NA	NA	FALSE
GB30121	NA	NA	NA	NA	FALSE
GB30122	51	21	1.280107919	0.415590411	FALSE
GB30123	12	28	-1.222392421	-2.08690993	TRUE
GB30124	97	65	0.577545029	-0.286972479	FALSE
GB30125	17	13	0.387023123	-0.477494385	FALSE
GB30126	158	61	1.373043411	0.508525902	TRUE
GB30127	NA	NA	NA	NA	FALSE
GB30128	18	15	0.263034406	-0.601483102	TRUE
GB30129	NA	NA	NA	NA	FALSE
GB30131	NA	NA	NA	NA	FALSE
GB30132	NA	NA	NA	NA	FALSE
GB30133	NA	NA	NA	NA	FALSE
GB30134	NA	NA	NA	NA	FALSE
GB30135	NA	NA	NA	NA	FALSE
GB30136	1	1	0	-0.864517508	TRUE
GB30137	NA	NA	NA	NA	FALSE
GB30138	136	65	1.065095028	0.20057752	FALSE

GB30139	6	2	1.584962501	0.720444992	TRUE
GB30140	240	113	1.086711633	0.222194125	FALSE
GB30141	NA	NA	NA	NA	FALSE
GB30142	4	1	2	1.135482492	TRUE
GB30143	NA	NA	NA	NA	FALSE
GB30144	1	0	NA	NA	FALSE
GB30145	NA	NA	NA	NA	FALSE
GB30146	1	0	NA	NA	FALSE
GB30148	1	0	NA	NA	FALSE
GB30149	6	0	NA	NA	FALSE
GB30150	3745	1923	0.961606956	0.097089448	FALSE
GB30151	0	1	NA	NA	FALSE
GB30152	1	0	NA	NA	FALSE
GB30153	0	1	NA	NA	FALSE
GB30154	NA	NA	NA	NA	FALSE
GB30155	NA	NA	NA	NA	FALSE
GB30156	NA	NA	NA	NA	FALSE
GB30158	1	0	NA	NA	FALSE
GB30159	NA	NA	NA	NA	FALSE
GB30160	4	0	NA	NA	FALSE
GB30161	NA	NA	NA	NA	FALSE
GB30162	3071	1732	0.826269582	-0.038247926	FALSE
GB30163	73	39	0.90442234	0.039904832	FALSE
GB30164	3	0	NA	NA	FALSE
GB30165	13	10	0.378511623	-0.486005885	FALSE
GB30166	93	27	1.784271309	0.919753801	TRUE
GB30167	NA	NA	NA	NA	FALSE
GB30168	NA	NA	NA	NA	FALSE
GB30169	1	2	-1	-1.864517508	TRUE
GB30170	0	1	NA	NA	FALSE
GB30171	NA	NA	NA	NA	FALSE
GB30172	3	2	0.584962501	-0.279555008	FALSE
GB30173	NA	NA	NA	NA	FALSE
GB30174	NA	NA	NA	NA	FALSE
GB30175	NA	NA	NA	NA	FALSE
GB30176	2	0	NA	NA	FALSE
GB30177	0	2	NA	NA	FALSE
GB30178	1	0	NA	NA	FALSE
GB30179	1	1	0	-0.864517508	TRUE
GB30180	NA	NA	NA	NA	FALSE
GB30181	NA	NA	NA	NA	FALSE
GB30182	NA	NA	NA	NA	FALSE
GB30183	NA	NA	NA	NA	FALSE
GB30184	NA	NA	NA	NA	FALSE

GB30185	3	0	NA	NA	FALSE
GB30186	NA	NA	NA	NA	FALSE
GB30186_2	NA	NA	NA	NA	FALSE
GB30187	NA	NA	NA	NA	FALSE
GB30188	1	0	NA	NA	FALSE
GB30189	28	27	0.05246742	-0.812050088	TRUE
GB30190	NA	NA	NA	NA	FALSE
GB30191	1	1	0	-0.864517508	TRUE
GB30192	NA	NA	NA	NA	FALSE
GB30193	NA	NA	NA	NA	FALSE
GB30194	NA	NA	NA	NA	FALSE
GB30195	77	34	1.179323699	0.314806191	FALSE
GB30196	NA	NA	NA	NA	FALSE
GB30197	1056	570	0.88957601	0.025058502	FALSE
GB30198	0	1	NA	NA	FALSE
GB30199	NA	NA	NA	NA	FALSE
GB30200	45	14	1.684498174	0.819980666	TRUE
GB30201	1	0	NA	NA	FALSE
GB30202	NA	NA	NA	NA	FALSE
GB30203	NA	NA	NA	NA	FALSE
GB30204	NA	NA	NA	NA	FALSE
GB30205	37	20	0.887525271	0.023007762	FALSE
GB30206	177	86	1.041340795	0.176823287	FALSE
GB30207	5	0	NA	NA	FALSE
GB30208	1	1	0	-0.864517508	TRUE
GB30209	52	31	0.746243408	-0.118274101	FALSE
GB30210	NA	NA	NA	NA	FALSE
GB30211	26	20	0.378511623	-0.486005885	FALSE
GB30212	NA	NA	NA	NA	FALSE
GB30213	17	5	1.765534746	0.901017238	TRUE
GB30214	79	37	1.094327383	0.229809874	FALSE
GB30215	NA	NA	NA	NA	FALSE
GB30216	240	129	0.89566334	0.031145832	FALSE
GB30217	1	1	0	-0.864517508	TRUE
GB30218	NA	NA	NA	NA	FALSE
GB30219	NA	NA	NA	NA	FALSE
GB30222	NA	NA	NA	NA	FALSE
GB30223	NA	NA	NA	NA	FALSE
GB30224	14	3	2.222392421	1.357874913	TRUE
GB30225	76	22	1.788495895	0.923978386	TRUE
GB30226	NA	NA	NA	NA	FALSE
GB30227	NA	NA	NA	NA	FALSE
GB30227_2	NA	NA	NA	NA	FALSE
GB30228	NA	NA	NA	NA	FALSE

GB30229	1	1	0	-0.864517508	TRUE
GB30230	220	133	0.726077278	-0.13844023	FALSE
GB30231	13	6	1.115477217	0.250959709	FALSE
GB30232	115	77	0.57870351	-0.285813998	FALSE
GB30233	0	1	NA	NA	FALSE
GB30234	NA	NA	NA	NA	FALSE
GB30235	31	14	1.146841388	0.28232388	FALSE
GB30236	47	39	0.269186633	-0.595330876	TRUE
GB30237	NA	NA	NA	NA	FALSE
GB30238	636	296	1.10342959	0.238912081	FALSE
GB30239	339	151	1.166736724	0.302219215	FALSE
GB30240	0	1	NA	NA	FALSE
GB30241	NA	NA	NA	NA	FALSE
GB30242	NA	NA	NA	NA	FALSE
GB30243	NA	NA	NA	NA	FALSE
GB30244	408	254	0.683740655	-0.180776853	FALSE
GB30245	NA	NA	NA	NA	FALSE
GB30246	0	1	NA	NA	FALSE
GB30247	NA	NA	NA	NA	FALSE
GB30248	8	7	0.192645078	-0.67187243	TRUE
GB30249	NA	NA	NA	NA	FALSE
GB30250	7	7	0	-0.864517508	TRUE
GB30251	3	1	1.584962501	0.720444992	TRUE
GB30252	NA	NA	NA	NA	FALSE
GB30253	NA	NA	NA	NA	FALSE
GB30255	2	2	0	-0.864517508	TRUE
GB30256	NA	NA	NA	NA	FALSE
GB30257	NA	NA	NA	NA	FALSE
GB30258	3	1	1.584962501	0.720444992	TRUE
GB30259	NA	NA	NA	NA	FALSE
GB30260	5	2	1.321928095	0.457410587	FALSE
GB30261	1	4	-2	-2.864517508	TRUE
GB30262	2	0	NA	NA	FALSE
GB30263	3	0	NA	NA	FALSE
GB30264	5	2	1.321928095	0.457410587	FALSE
GB30265	NA	NA	NA	NA	FALSE
GB30266	83	55	0.593679718	-0.270837791	FALSE
GB30267	NA	NA	NA	NA	FALSE
GB30268	106367	65244	0.70513349	-0.159384018	FALSE
GB30269	681	329	1.049567214	0.185049706	FALSE
GB30270	0	2	NA	NA	FALSE
GB30271	5	2	1.321928095	0.457410587	FALSE
GB30272	3	3	0	-0.864517508	TRUE
GB30274	2	0	NA	NA	FALSE

GB30275	NA	NA	NA	NA	FALSE
GB30276	1	1	0	-0.864517508	TRUE
GB30277	1	0	NA	NA	FALSE
GB30278	NA	NA	NA	NA	FALSE
GB30279	1	0	NA	NA	FALSE
GB30280	7	0	NA	NA	FALSE
GB30281	NA	NA	NA	NA	FALSE
GB30282	NA	NA	NA	NA	FALSE
GB30283	2	0	NA	NA	FALSE
GB30284	NA	NA	NA	NA	FALSE
GB30285	NA	NA	NA	NA	FALSE
GB30286	NA	NA	NA	NA	FALSE
GB30287	1	0	NA	NA	FALSE
GB30288	38	24	0.662965013	-0.201552496	FALSE
GB30289	916	468	0.968839069	0.10432156	FALSE
GB30289_2	NA	NA	NA	NA	FALSE
GB30290	NA	NA	NA	NA	FALSE
GB30291	NA	NA	NA	NA	FALSE
GB30292	NA	NA	NA	NA	FALSE
GB30293	24	5	2.263034406	1.398516898	TRUE
GB30294	NA	NA	NA	NA	FALSE
GB30295	NA	NA	NA	NA	FALSE
GB30296	1	0	NA	NA	FALSE
GB30297	NA	NA	NA	NA	FALSE
GB30297_2	NA	NA	NA	NA	FALSE
GB30298	54	36	0.584962501	-0.279555008	FALSE
GB30299	NA	NA	NA	NA	FALSE
GB30300	NA	NA	NA	NA	FALSE
GB30301	NA	NA	NA	NA	FALSE
GB30302	NA	NA	NA	NA	FALSE
GB30303	NA	NA	NA	NA	FALSE
GB30304	22	16	0.459431619	-0.40508589	FALSE
GB30305	6	5	0.263034406	-0.601483102	TRUE
GB30306	1	0	NA	NA	FALSE
GB30307	6	2	1.584962501	0.720444992	TRUE
GB30308	1	5	-2.321928095	-3.186445603	TRUE
GB30309	30	15	1	0.135482492	FALSE
GB30310	466	291	0.679310802	-0.185206707	FALSE
GB30312	49	6	3.029747343	2.165229835	TRUE
GB30313	374	272	0.459431619	-0.40508589	FALSE
GB30314	199	91	1.12882998	0.264312472	FALSE
GB30315	NA	NA	NA	NA	FALSE
GB30316	NA	NA	NA	NA	FALSE
GB30317	NA	NA	NA	NA	FALSE

GB30318	2	0	NA	NA	FALSE
GB30319	NA	NA	NA	NA	FALSE
GB30320	5	3	0.736965594	-0.127551914	FALSE
GB30321	6	2	1.584962501	0.720444992	TRUE
GB30322	NA	NA	NA	NA	FALSE
GB30323	2	1	1	0.135482492	FALSE
GB30324	3	1	1.584962501	0.720444992	TRUE
GB30325	161	103	0.644416351	-0.220101157	FALSE
GB30326	NA	NA	NA	NA	FALSE
GB30327	39	23	0.761840263	-0.102677246	FALSE
GB30328	293	181	0.694910967	-0.169606541	FALSE
GB30329	4352	2064	1.076235586	0.211718077	FALSE
GB30330	NA	NA	NA	NA	FALSE
GB30331	64	53	0.272079545	-0.592437963	TRUE
GB30332	351	169	1.054447784	0.189930276	FALSE
GB30333	2	0	NA	NA	FALSE
GB30334	462	206	1.165248514	0.300731006	FALSE
GB30335	1	4	-2	-2.864517508	TRUE
GB30336	NA	NA	NA	NA	FALSE
GB30337	NA	NA	NA	NA	FALSE
GB30338	0	1	NA	NA	FALSE
GB30338_2	NA	NA	NA	NA	FALSE
GB30339	921	640	0.525129251	-0.339388257	FALSE
GB30340	159	73	1.123058396	0.258540888	FALSE
GB30341	282	111	1.345135486	0.480617978	FALSE
GB30342	NA	NA	NA	NA	FALSE
GB30342_2	1	0	NA	NA	FALSE
GB30343	327	139	1.234205753	0.369688244	FALSE
GB30343_2	3	3	0	-0.864517508	TRUE
GB30344	0	2	NA	NA	FALSE
GB30345	1	1	0	-0.864517508	TRUE
GB30346	0	1	NA	NA	FALSE
GB30347	1	2	-1	-1.864517508	TRUE
GB30348	2	2	0	-0.864517508	TRUE
GB30349	14	13	0.106915204	-0.757602304	TRUE
GB30350	53	25	1.084064265	0.219546756	FALSE
GB30351	166	109	0.606855107	-0.257662402	FALSE
GB30352	118	50	1.23878686	0.374269351	FALSE
GB30353	6	4	0.584962501	-0.279555008	FALSE
GB30354	NA	NA	NA	NA	FALSE
GB30356	NA	NA	NA	NA	FALSE
GB30357	6	2	1.584962501	0.720444992	TRUE
GB30358	269	185	0.540080902	-0.324436606	FALSE
GB30359	1367	861	0.6669281	-0.197589408	FALSE

GB30360	NA	NA	NA	NA	FALSE
GB30361	NA	NA	NA	NA	FALSE
GB30362	151889	79650	0.931271125	0.066753617	FALSE
GB30363	NA	NA	NA	NA	FALSE
GB30364	NA	NA	NA	NA	FALSE
GB30365	2134	1868	0.192065721	-0.672451787	TRUE
GB30366	817	488	0.743454931	-0.121062578	FALSE
GB30367	810	430	0.913585248	0.04906774	FALSE
GB30368	80	43	0.89566334	0.031145832	FALSE
GB30369	1	0	NA	NA	FALSE
GB30370	NA	NA	NA	NA	FALSE
GB30371	11	13	-0.2410081	-1.105525608	TRUE
GB30372	31	5	2.632268215	1.767750707	TRUE
GB30373	1	1	0	-0.864517508	TRUE
GB30374	2	2	0	-0.864517508	TRUE
GB30375	212	105	1.013674937	0.149157429	FALSE
GB30376	28	14	1	0.135482492	FALSE
GB30377	24	15	0.678071905	-0.186445603	FALSE
GB30378	5	0	NA	NA	FALSE
GB30379	NA	NA	NA	NA	FALSE
GB30380	594	471	0.334735871	-0.529781637	TRUE
GB30381	NA	NA	NA	NA	FALSE
GB30382	218	168	0.375866902	-0.488650606	FALSE
GB30383	539	337	0.677536682	-0.186980827	FALSE
GB30384	2	3	-0.584962501	-1.449480009	TRUE
GB30385	2	1	1	0.135482492	FALSE
GB30386	8	2	2	1.135482492	TRUE
GB30387	1	2	-1	-1.864517508	TRUE
GB30388	4306	2911	0.56483348	-0.299684028	FALSE
GB30389	NA	NA	NA	NA	FALSE
GB30390	NA	NA	NA	NA	FALSE
GB30391	340	270	0.332575339	-0.531942169	TRUE
GB30392	1	0	NA	NA	FALSE
GB30393	NA	NA	NA	NA	FALSE
GB30395	174	91	0.935148856	0.070631347	FALSE
GB30397	3	0	NA	NA	FALSE
GB30399	148	104	0.509013647	-0.355503861	FALSE
GB30400	1787	1166	0.615971846	-0.248545662	FALSE
GB30401	4	0	NA	NA	FALSE
GB30402	NA	NA	NA	NA	FALSE
GB30403	0	1	NA	NA	FALSE
GB30404	492	194	1.342601663	0.478084155	FALSE
GB30405	972	626	0.634793657	-0.229723852	FALSE
GB30406	13	9	0.530514717	-0.334002792	FALSE

GB30407	6099	2729	1.160200321	0.295682813	FALSE
GB30408	1	0	NA	NA	FALSE
GB30409	56	35	0.678071905	-0.186445603	FALSE
GB30410	40	9	2.152003093	1.287485585	TRUE
GB30412	372	225	0.72537762	-0.139139888	FALSE
GB30413	274	154	0.831245542	-0.033271966	FALSE
GB30414	381	218	0.805462863	-0.059054646	FALSE
GB30416	15722	9253	0.764791659	-0.099725849	FALSE
GB30417	1147	485	1.241808739	0.377291231	FALSE
GB30418	2	6	-1.584962501	-2.449480009	TRUE
GB30419	3	0	NA	NA	FALSE
GB30420	5	2	1.321928095	0.457410587	FALSE
GB30421	2	0	NA	NA	FALSE
GB30423	NA	NA	NA	NA	FALSE
GB30426	NA	NA	NA	NA	FALSE
GB30427	NA	NA	NA	NA	FALSE
GB30428	NA	NA	NA	NA	FALSE
GB30429	1	0	NA	NA	FALSE
GB30431	0	1	NA	NA	FALSE
GB30432	1	4	-2	-2.864517508	TRUE
GB30434	1	0	NA	NA	FALSE
GB30435	0	1	NA	NA	FALSE
GB30436	NA	NA	NA	NA	FALSE
GB30437	NA	NA	NA	NA	FALSE
GB30438	NA	NA	NA	NA	FALSE
GB30439	NA	NA	NA	NA	FALSE
GB30440	NA	NA	NA	NA	FALSE
GB30441	NA	NA	NA	NA	FALSE
GB30442	NA	NA	NA	NA	FALSE
GB30443	NA	NA	NA	NA	FALSE
GB30444	NA	NA	NA	NA	FALSE
GB30445	NA	NA	NA	NA	FALSE
GB30446	NA	NA	NA	NA	FALSE
GB30447	NA	NA	NA	NA	FALSE
GB30448	NA	NA	NA	NA	FALSE
GB30449	0	1	NA	NA	FALSE
GB30450	0	2	NA	NA	FALSE
GB30451	NA	NA	NA	NA	FALSE
GB30452	NA	NA	NA	NA	FALSE
GB30453	NA	NA	NA	NA	FALSE
GB30454	NA	NA	NA	NA	FALSE
GB30455	NA	NA	NA	NA	FALSE
GB30456	NA	NA	NA	NA	FALSE
GB30457	NA	NA	NA	NA	FALSE

GB30458	NA	NA	NA	NA	FALSE
GB30458_2	1	1	0	-0.864517508	TRUE
GB30459	6	3	1	0.135482492	FALSE
GB30460	NA	NA	NA	NA	FALSE
GB30462	20	10	1	0.135482492	FALSE
GB30463	0	1	NA	NA	FALSE
GB30463_2	30	6	2.321928095	1.457410587	TRUE
GB30465	NA	NA	NA	NA	FALSE
GB30467	93	74	0.329705445	-0.534812063	TRUE
GB30468	218	64	1.768184325	0.903666816	TRUE
GB30470	1	0	NA	NA	FALSE
GB30471	8	4	1	0.135482492	FALSE
GB30472	7	2	1.807354922	0.942837414	TRUE
GB30473	NA	NA	NA	NA	FALSE
GB30474	9	2	2.169925001	1.305407493	TRUE
GB30476	NA	NA	NA	NA	FALSE
GB30477	3	1	1.584962501	0.720444992	TRUE
GB30478	14	3	2.222392421	1.357874913	TRUE
GB30480	10	3	1.736965594	0.872448086	TRUE
GB30482	NA	NA	NA	NA	FALSE
GB30483	NA	NA	NA	NA	FALSE
GB30484	0	2	NA	NA	FALSE
GB30485	NA	NA	NA	NA	FALSE
GB30486	NA	NA	NA	NA	FALSE
GB30487	NA	NA	NA	NA	FALSE
GB30489	0	3	NA	NA	FALSE
GB30490	0	1	NA	NA	FALSE
GB30491	411	187	1.136100124	0.271582615	FALSE
GB30491_2	522	249	1.067904065	0.203386556	FALSE
GB30491_3	25	13	0.943416472	0.078898963	FALSE
GB30491_4	327	175	0.901935714	0.037418205	FALSE
GB30492	8749	5099	0.77890379	-0.085613718	FALSE
GB30492_2	676	395	0.775170593	-0.089346915	FALSE
GB30493	4	4	0	-0.864517508	TRUE
GB30493_2	8	8	0	-0.864517508	TRUE
GB30494	3	0	NA	NA	FALSE
GB30504	NA	NA	NA	NA	FALSE
GB30505	219	144	0.604862058	-0.25965545	FALSE
GB30506	NA	NA	NA	NA	FALSE
GB30507	NA	NA	NA	NA	FALSE
GB30508	NA	NA	NA	NA	FALSE
GB30509	NA	NA	NA	NA	FALSE
GB30509_2	1	0	NA	NA	FALSE
GB30510	2	1	1	0.135482492	FALSE

GB30510_2	374	237	0.658151211	-0.206366297	FALSE
GB30511	1012	761	0.411240931	-0.453276577	FALSE
GB30511_2	722	515	0.487426405	-0.377091104	FALSE
GB30511_3	NA	NA	NA	NA	FALSE
GB30512	1012	576	0.813068573	-0.051448935	FALSE
GB30513	NA	NA	NA	NA	FALSE
GB30513_2	NA	NA	NA	NA	FALSE
GB30514	NA	NA	NA	NA	FALSE
GB30515	NA	NA	NA	NA	FALSE
GB30517	NA	NA	NA	NA	FALSE
GB30518	1444	736	0.972293071	0.107775563	FALSE
GB30519	131	99	0.404066381	-0.460451127	FALSE
GB30520	NA	NA	NA	NA	FALSE
GB30520_2	36	24	0.584962501	-0.279555008	FALSE
GB30521	282	136	1.052088511	0.187571003	FALSE
GB30522	NA	NA	NA	NA	FALSE
GB30523	NA	NA	NA	NA	FALSE
GB30525	6242	5174	0.270728303	-0.593789205	TRUE
GB30526	1	0	NA	NA	FALSE
GB30527	34	32	0.087462841	-0.777054667	TRUE
GB30527_2	NA	NA	NA	NA	FALSE
GB30528	NA	NA	NA	NA	FALSE
GB30528_2	NA	NA	NA	NA	FALSE
GB30529	NA	NA	NA	NA	FALSE
GB30529_2	25	20	0.321928095	-0.542589413	TRUE
GB30530	349	230	0.601593175	-0.262924333	FALSE
GB30530_2	NA	NA	NA	NA	FALSE
GB30531	NA	NA	NA	NA	FALSE
GB30532	3	0	NA	NA	FALSE
GB30533	260	185	0.490986353	-0.373531156	FALSE
GB30534	1	3	-1.584962501	-2.449480009	TRUE
GB30535	NA	NA	NA	NA	FALSE
GB30540	NA	NA	NA	NA	FALSE
GB30540_2	348	164	1.085391491	0.220873983	FALSE
GB30541	NA	NA	NA	NA	FALSE
GB30541_2	NA	NA	NA	NA	FALSE
GB30542	NA	NA	NA	NA	FALSE
GB30542_2	NA	NA	NA	NA	FALSE
GB30543	16	6	1.415037499	0.550519991	TRUE
GB30544	42	28	0.584962501	-0.279555008	FALSE
GB30545	846	390	1.117183539	0.252666031	FALSE
GB30547	NA	NA	NA	NA	FALSE
GB30548	4	1	2	1.135482492	TRUE
GB30549	NA	NA	NA	NA	FALSE

GB30550	NA	NA	NA	NA	FALSE
GB30551	3	1	1.584962501	0.720444992	TRUE
GB30551_2	196	161	0.283792966	-0.580724542	TRUE
GB30552	NA	NA	NA	NA	FALSE
GB30553	66	29	1.186413124	0.321895616	FALSE
GB30553_2	NA	NA	NA	NA	FALSE
GB30554	5	2	1.321928095	0.457410587	FALSE
GB30555	3	1	1.584962501	0.720444992	TRUE
GB30555_2	108	39	1.469485283	0.604967775	TRUE
GB30556	6	2	1.584962501	0.720444992	TRUE
GB30557	172	115	0.580774704	-0.283742805	FALSE
GB30558	NA	NA	NA	NA	FALSE
GB30559	1953	897	1.122512059	0.257994551	FALSE
GB30560	228	110	1.051530301	0.187012792	FALSE
GB30561	193	126	0.615177114	-0.249340395	FALSE
GB30561_2	NA	NA	NA	NA	FALSE
GB30562	30	22	0.447458977	-0.417058531	FALSE
GB30563	0	1	NA	NA	FALSE
GB30564	132	98	0.429684275	-0.434833233	FALSE
GB30565	86	54	0.671377253	-0.193140256	FALSE
GB30566	3	7	-1.222392421	-2.08690993	TRUE
GB30567	NA	NA	NA	NA	FALSE
GB30568	34	22	0.628031223	-0.236486286	FALSE
GB30569	3	1	1.584962501	0.720444992	TRUE
GB30570	165	81	1.026472211	0.161954703	FALSE
GB30571	1012	598	0.7589919	-0.105525608	FALSE
GB30572	NA	NA	NA	NA	FALSE
GB30573	NA	NA	NA	NA	FALSE
GB30574	NA	NA	NA	NA	FALSE
GB30575	8	5	0.678071905	-0.186445603	FALSE
GB30576	15	13	0.206450877	-0.658066631	TRUE
GB30577	NA	NA	NA	NA	FALSE
GB30578	1606	965	0.734871045	-0.129646463	FALSE
GB30579	59	19	1.634715536	0.770198028	TRUE
GB30579_2	47	25	0.910732662	0.046215154	FALSE
GB30580	NA	NA	NA	NA	FALSE
GB30580_2	NA	NA	NA	NA	FALSE
GB30581	1	3	-1.584962501	-2.449480009	TRUE
GB30582	194	102	0.9274875	0.062969992	FALSE
GB30582_2	387	214	0.85472277	-0.009794739	FALSE
GB30583	26	18	0.530514717	-0.334002792	FALSE
GB30584	19	14	0.440572591	-0.423944917	FALSE
GB30585	14	11	0.347923303	-0.516594205	TRUE
GB30585_2	NA	NA	NA	NA	FALSE

GB30586	21	16	0.392317423	-0.472200086	FALSE
GB30594	146	88	0.73039294	-0.134124568	FALSE
GB30595	325	181	0.844450021	-0.020067487	FALSE
GB30597	136	80	0.765534746	-0.098982762	FALSE
GB30598	NA	NA	NA	NA	FALSE
GB30599	7	5	0.485426827	-0.379090681	FALSE
GB31001	NA	NA	NA	NA	FALSE
GB31002	2	0	NA	NA	FALSE
