# Supplementary material for: Transient Exposure to Low Levels of Insecticide Affects Metabolic Networks of Honeybee Larvae
Source: PLoS One. 2013 Jul 2;8(7):e68191. doi: 10.1371/journal.pone.0068191 (PMC3699529; doi:10.1371/journal.pone.0068191)
Supplement: Table S2 — List of 300 differentially expressed genes. (PDF) [file pone.0068191.s004.pdf]

Table S2 **List of 300 genes with significantly altered RNA levels: DEGseq data comparing 'C' and 'IE' samples**

Transient exposure to low levels of insecticide affects metabolic network of honeybee larvae. Derecka et al. (2013)

A list of 300 differentially expressed genes was collated: Up-regulated in IE-samples = (red, down-regulated in IE-samples = blue. The seleceted genes had to pass the four statistical tests used by DEGseq analysis (Fisher's Exact Test, FET; Likelihood Ratio Test, LRT, and Map-Plot with Random Sampling MARS (p-value ≤0.001) and Fold Changes FC ≥0.5 (log2 normalised fold change). In addition, only genes with ≥ 10 reads per kilobase of exon model per million mapped reads (RPKM) in either 'C', 'IE', or both data sets were selected. Some of the *Apis mellifera* genes have multiple orthologs in *Drosophila;* only one Drosophila orthologue is listed. were up-regulated (red) in IE-samples, while seven miRNAs were down-regulated (blue).

Gene ID	Name or Function (partially based on *Drosophila* orthologs)	log2 (Fold_change) IE/C	log2 (Fold_change) normalised IE/C	IE RPKM	C RPKM	Sample IE Read Count	Sample C Read Count	*Drosophila* orthologs
GB14836	Cyp9-clade	4.2	3.3	41	4	4996	270	CG4485
GB18323	abaecin	2.5	1.6	51	17	643	116	abaecin
GB18530	Trypsin-like	2.2	1.4	12	4	729	154	CG10405
GB12762		2.0	1.1	46	20	742	186	
GB13108		2.0	1.1	10	5	172	44	
GB10134	chymotrypsin inhibitor-like	1.9	1.1	28	13	486	128	CG33259
GB18632	transmembrane transport	1.9	1.0	13	6	1351	363	CG1358
GB19113	Cyp6-clade	1.9	1.0	10	5	1015	276	CG8453
GB15793	Cyp6-clade	1.8	0.9	80	41	9422	2701	CG1644
GB12705	CG30101-like	1.8	0.9	10	5	862	248	CG30101
GB10483		1.8	0.9	12	6	1050	305	CG9449
GB18201		1.7	0.9	10	5	1128	338	CG14762
GB12190		1.7	0.8	37	20	6041	1853	CG9317
GB13722	Glucosylceramidase	1.7	0.8	24	13	2927	902	CG31148
GB30001		1.7	0.8	48	27	4086	1262	CG16885
GB12607	Maltase	1.7	0.8	10	6	1452	452	CG8694
GB10387	Pxd	1.7	0.8	32	18	5222	1634	CG4009
GB16697		1.7	0.8	11	6	667	209	CG42575
GB10421	transmembrane transport	1.6	0.8	18	10	2262	724	CG14691
GB15681	Cyp6-clade	1.6	0.8	34	20	4030	1295	CG1644
GB16196	Phosphoenolpyruvate carboxykinase, PEPCK	1.6	0.8	28	16	4332	1399	CG10924
GB17220	Lip3-like	1.6	0.8	10	6	3193	1036	CG17292
GB13615	Kaz1-ORFB	1.6	0.8	129	75	3990	1301	CG1220
GB16424	transmembrane transport	1.6	0.7	49	29	6315	2078	CG3168
GB16803	Cyp9-clade	1.6	0.7	136	80	16992	5605	CG4486
GB18301	jdp	1.6	0.7	14	8	578	191	CG2239
GB17752	monosaccharide transmembrane transporter	1.6	0.7	11	6	1280	426	CG31100
GB14824	oxidoreductase activity	1.6	0.7	47	28	3174	1059	CG7675
GB16773	Tre1	1.6	0.7	21	12	1655	553	CG3171
GB18205	CG15444-like	1.6	0.7	15	9	3157	1062	CG15444
GB18013		1.6	0.7	1453	877	127369	43010	
GB10584	sphingolipid metabolic process	1.6	0.7	56	34	6946	2349	CG31148
GB16686		1.6	0.7	508	307	7056	2388	
GB14047	transmembrane transport	1.6	0.7	10	6	1123	381	CG1358
GB13397	Serine protease-like protein	1.6	0.7	13	8	913	310	
GB10070		1.5	0.7	11	7	804	276	
GB19754	Jhl-26 / Juvenile hormone-inducible protein 26	1.5	0.7	21	13	1878	646	CG3767
GB16807	GMC oxidoreductase, Glucose dehydrogenase	1.5	0.7	117	72	13193	4549	CG9512
GB16343	67 aa peptide	1.5	0.7	49	30	746	259	
GB15528	pirk	1.5	0.7	10	6	463	161	CG15678
GB10560	Ef1α48D	1.5	0.7	15	9	1604	558	CG8280
GB15046		1.5	0.7	35	22	4961	1727	
GB19959		1.5	0.7	15	9	201	70	CG30108
GB18684		1.5	0.7	204	127	15565	5426	
GB17579	Trypsin Inhibitor-like	1.5	0.7	452	282	9575	3345	CG34189
GB15696	Futsch	1.5	0.7	33	21	11699	4089	CG34387
GB10515		1.5	0.7	14	9	575	201	
GB17745	Lip3-like	1.5	0.6	10	6	967	340	CG8093
GB18360	4-hydroxyphenylpyruvate dioxygenase	1.5	0.6	12	8	1075	382	CG11796
GB11907		1.5	0.6	13	8	1334	475	
GB13230		1.5	0.6	29	18	365	130	
GB19643		1.5	0.6	20	13	5862	2090	
GB13601		1.5	0.6	105	67	3302	1178	CG8541
GB13129		1.5	0.6	11	7	655	234	
GB19020	Nervana 2	1.5	0.6	21	13	1542	553	CG9261
GB20104	glycerate kinase activity	1.5	0.6	80	52	1969	708	CG9886
GB17588	Cyp6-clade	1.5	0.6	255	164	38540	13859	CG10240
GB10325	Chloride channel-a	1.5	0.6	18	12	3883	1402	CG31116
GB13059	Phytanoyl-CoA dioxygenase	1.5	0.6	17	11	1901	688	CG14688
GB13049	β-Tubulin at 85D	1.5	0.6	70	46	7380	2685	CG9359
GB10905	Cyp4-clade	1.5	0.6	59	39	7148	2608	CG11715
GB19587	Skel2	1.5	0.6	12	8	3785	1382	CG43161
GB14105	Organic anion transporting polypeptide 58Dc	1.5	0.6	57	37	9610	3510	CG3380

GB12549	GlcAT-P	1.4	0.6	13	9	1173	432	CG6207
GB10018	Scp2	1.4	0.6	257	169	11121	4098	CG14904
GB11412	oxidation-reduction process	1.4	0.6	10	6	1225	453	CG1443
GB19766	Sugar transporter	1.4	0.6	14	9	1538	573	
GB13606		1.4	0.6	38	25	1584	591	
GB18082	CHK kinase-like	1.4	0.6	17	11	1598	598	CG13360
GB13037	Msr-110	1.4	0.5	84	57	14448	5452	CG10596
GB13289	Fatty Acyl-CoA Synthetase	1.4	0.5	100	68	15983	6063	CG6178
GB16444		1.4	0.5	22	15	4561	1733	CG10737
GB13748	Cyp9-clade	1.4	0.5	12	8	1394	531	CG4486
GB14926	Uracil-DNA degrading factor	1.4	0.5	15	11	1635	623	CG18410
GB10428		1.4	0.5	1939	1327	57109	21858	CG33998
GB19819	Immune-regulated catalase	1.4	0.5	288	198	54600	21043	CG8913
GB11402		1.4	0.5	20	14	306	118	
GB15759	Sodium/solute symporter	1.4	0.5	36	25	4987	1927	CG42235
GB12293	Pyridoxal phosphate-dependent enzyme	1.4	0.5	358	247	39188	15154	CG8129
GB16236	sarcomere organization	1.4	0.5	56	39	14310	5538	CG32019
GB14481	cation transport	1.4	0.5	11	7	3111	1207	CG32000
GB17011		1.4	0.5	14	9	687	267	CG14968
GB16057		1.4	0.5	16	11	1802	702	CG42323
GB14556		1.4	0.5	23	16	930	364	CG14950
GB13263	Peptidase S1/S6	1.4	0.5	31	22	5285	2073	CG12951
GB20148	de novo' pyrimidine base biosynthetic process	1.3	0.5	8496	5970	760623	299029	CG3027
GB18896	Glycoside hydrolase	1.3	0.5	179	126	19591	7706	CG9701
GB16450		1.3	0.5	16	11	1054	415	
GB19742		1.3	0.5	1002	706	28092	11067	CG8369
GB11679		1.3	0.5	115	81	10580	4172	CG4818
GB19328	Jupiter	1.3	0.5	89	63	4453	1760	CG31363
GB15784		1.3	0.5	22	15	862	341	CG10311
GB10339	HSP20-like chaperone	1.3	0.5	226	160	10188	4031	CG4533
GB14589		1.3	0.5	19	13	647	256	CG14515
GB14913	Cyp6-clade	1.3	0.5	151	107	17820	7058	CG1644
GB13754	Hydr2	1.3	0.5	31	22	2941	1166	CG3488
GB15569		1.3	0.5	975	693	16307	6487	CG13315
GB17188	PGRP-LC	1.3	0.5	21	15	2185	871	CG4432
GB15721		1.3	0.5	10	7	937	374	
GB17161		1.3	0.5	14	10	541	216	CG31038
GB14161		1.3	0.5	13	9	1152	460	CG10933
GB16927		1.3	0.5	44	32	5992	2394	
GB11298		1.3	0.5	125	89	12262	4903	CG5390
GB10114	Cabut	1.3	0.5	41	29	3587	1440	CG4427
GB11698	Peptidase S1/S6	1.3	0.5	32	23	2687	1079	CG32260
GB11741	Bet3	0.4	-0.5	16	22	685	514	CG3911
GB13187		0.4	-0.5	9	12	409	307	CG12106
GB11001		0.4	-0.5	19	26	1006	756	CG9849
GB18003	eIF4E-4-like	0.4	-0.5	28	37	1556	1170	CG4035
GB30511	Glycoside hydrolase	0.4	-0.5	17	23	1012	761	CG33138
GB18898	TRAM	0.4	-0.5	106	143	9440	7104	CG11642
GB11790		0.4	-0.5	21	28	586	441	
GB15486	SelR	0.4	-0.5	74	100	2630	1980	CG6584
GB17464		0.4	-0.5	13	17	2164	1630	CG4840
GB14158		0.4	-0.5	14	19	596	449	CG9099
GB13770	FKBP59	0.4	-0.5	25	34	2740	2065	CG4535
GB19410		0.4	-0.5	10	13	554	418	CG1381
GB18113	Mcc	0.4	-0.5	16	21	632	477	CG2118
GB11205	blos2	0.4	-0.5	8	11	310	234	CG14145
GB19639	ttm3	0.4	-0.5	18	24	1640	1238	CG6691
GB15959	Gprk1	0.4	-0.5	10	13	1547	1170	CG40129
GB15565		0.4	-0.5	21	28	2168	1641	CG5044
GB15877	SmF	0.4	-0.5	37	50	769	583	CG16792
GB11144	mRpL51	0.4	-0.5	24	33	860	652	CG13098
GB13589		0.4	-0.5	11	15	335	254	CG12975
GB19885	pyruvate carboxylase	0.4	-0.5	112	152	28763	21816	CG1516
GB13512		0.4	-0.5	9	12	294	223	CG7506
GB11274		0.4	-0.5	7	10	804	610	CG11710
GB18904		0.4	-0.5	60	82	8160	6192	CG32751
GB19618	resilin	0.4	-0.5	10	13	506	384	CG15920
GB16779		0.4	-0.5	18	24	1943	1475	CG32112
GB19017	Mal-B1	0.4	-0.5	13	18	1765	1340	CG14934
GB19888		0.4	-0.5	33	45	4772	3628	CG12140
GB18577		0.4	-0.5	8	11	539	410	CG6984
GB18373		0.4	-0.5	20	27	464	353	CG14817
GB17684	ACC	0.4	-0.5	15	20	8319	6330	CG11198
GB18918	zfh1	0.4	-0.5	25	34	3898	2968	CG1322
GB17462		0.4	-0.5	10	14	587	447	
GB18156	Probable splicing factor 3B	0.4	-0.5	33	44	659	502	CG11985
GB16429	Pgi	0.4	-0.5	99	135	12942	9880	CG8251
GB18124	CHOp24	0.4	-0.5	67	91	3243	2476	CG3564
GB19061	Ssb-c31a	0.4	-0.5	43	59	1210	925	CG8396
GB11017		0.4	-0.5	14	20	3551	2716	CG5660
GB10695	PyK	0.4	-0.5	115	157	14658	11217	CG7070
GB17044	nop5	0.4	-0.5	10	14	1272	974	CG10206
GB17943	eIF-2α	0.4	-0.5	28	39	664	509	CG9946
GB12674		0.4	-0.5	11	15	814	624	

GB10353	RpS15	0.4	-0.5	530	727	18482	14185	CG8332
GB19030		0.4	-0.5	166	228	12420	9537	CG6084
GB11162	mRpS35	0.4	-0.5	8	12	642	493	CG2101
GB18390	Mys45A	0.4	-0.5	7	10	1362	1046	CG8070
GB17163		0.4	-0.5	7	10	496	381	CG10476
GB18490		0.4	-0.5	12	17	1122	862	CG2970
GB13924	Sec61β	0.4	-0.5	213	293	4777	3671	CG10130
GB12603		0.4	-0.5	17	23	295	227	CG30373
GB14000	P58IPK	0.4	-0.5	13	17	1562	1202	CG8286
GB16200		0.4	-0.5	48	66	1088	838	CG40002
GB11240	phospholipid biosynthetic process	0.4	-0.5	53	73	10555	8132	CG5508
GB11516		0.4	-0.5	8	11	567	437	CG10336
GB12528		0.4	-0.5	51	71	2680	2068	CG11999
GB10242		0.4	-0.5	8	12	330	255	CG1307
GB12712	rRNA methyltransferase activity	0.4	-0.5	8	12	1680	1300	CG8939
GB19860	Hsc70-5	0.4	-0.5	75	104	12151	9407	CG8542
GB18572	Glycerol kinase	0.4	-0.5	7	10	1020	790	CG18374
GB17096		0.4	-0.5	60	83	1140	883	CG9669
GB17273	tsunagi	0.4	-0.5	15	20	573	444	CG8781
GB16147		0.4	-0.5	22	31	1951	1512	
GB13507		0.4	-0.5	23	32	1731	1342	CG10621
GB14684		0.4	-0.5	17	24	1074	834	CG30008
GB14709	AQuaPorin?	0.4	-0.5	122	169	8851	6882	CG4019
GB15762	Gale	0.4	-0.5	28	39	2344	1823	CG12030
GB15885		0.4	-0.5	59	82	679	529	CG17776
GB15143	RpL36A	0.4	-0.5	402	559	9839	7666	CG7424
GB17626	Pyruvate dehydrogenase E3 subunit / DHLDH	0.4	-0.5	30	42	3609	2814	CG7430
GB14723	mRpL12	0.4	-0.5	29	40	1231	960	CG5012
GB16443	lethal (1) G0334	0.4	-0.5	70	98	6562	5123	CG7010
GB13540	eIF-1A	0.4	-0.5	38	53	1334	1042	CG8053
GB14121		0.4	-0.5	15	21	559	437	CG7048
GB10130	Cct1	0.4	-0.5	13	18	1238	969	CG1049
GB13009		0.4	-0.5	8	11	267	209	CG3224
GB10109		0.4	-0.5	15	21	1504	1178	CG6404
GB11230		0.4	-0.5	8	11	374	293	CG32147
GB16445		0.4	-0.5	35	49	980	768	CG12859
GB19983		0.4	-0.5	14	20	343	269	CG31950
GB19936		0.4	-0.5	7	10	385	302	CG2046
GB10176		0.3	-0.5	61	85	4480	3515	
GB13622		0.3	-0.5	13	18	539	423	CG11329
GB18899	Adh	0.3	-0.5	174	244	10938	8584	CG3481
GB17282	Crc	0.3	-0.5	719	1009	68393	53712	CG9429
GB14051		0.3	-0.5	58	82	6371	5012	CG9547
GB11322	Sec22	0.3	-0.5	19	26	943	742	CG7359
GB14892		0.3	-0.5	12	17	374	295	CG9865
GB12976		0.3	-0.5	7	10	861	680	CG8791
GB15726		0.3	-0.5	24	34	1550	1228	CG32857
GB10219		0.3	-0.5	16	23	1970	1561	CG9706
GB16219		0.3	-0.5	29	42	1090	864	
GB10714		0.3	-0.5	16	23	594	471	CG7950
GB11850	LP04985p	0.3	-0.5	15	22	543	431	CG11258
GB30391		0.3	-0.5	8	12	340	270	
GB13237	Pgm	0.3	-0.5	26	36	3400	2705	CG5165
GB11348	Nop60B	0.3	-0.5	11	16	1333	1062	CG3333
GB19014	mRpL9	0.3	-0.5	25	35	752	602	CG31478
GB12841		0.3	-0.5	74	105	1420	1140	CG7580
GB11568		0.3	-0.5	7	10	342	275	CG7168
GB15039	Eno	0.3	-0.6	207	298	16118	12962	CG17654
GB16464	Mdh2	0.3	-0.6	161	232	12851	10364	CG7998
GB10107	P32	0.3	-0.6	75	108	4726	3821	CG6459
GB17592	SsRβ	0.3	-0.6	130	188	5892	4766	CG5474
GB19399		0.3	-0.6	8	11	3565	2885	
GB10732		0.3	-0.6	29	43	6500	5261	CG2918
GB14342		0.3	-0.6	7	11	693	561	CG3021
GB16520	FK506-bp1	0.3	-0.6	20	29	1688	1367	CG6226
GB15960		0.3	-0.6	10	15	385	312	CG31957
GB14098		0.3	-0.6	9	14	529	429	CG6937
GB10926		0.3	-0.6	17	25	415	337	CG14290
GB12901	T3dh	0.3	-0.6	13	19	1272	1036	CG3425
GB16757		0.3	-0.6	21	30	2283	1860	CG12288
GB12860	Tudor-SN	0.3	-0.6	67	98	14162	11546	CG7008
GB17630	Tim13	0.3	-0.6	24	36	525	429	CG11611
GB12586	Pdi	0.3	-0.6	184	269	11754	9605	CG6988
GB16636		0.3	-0.6	14	21	329	269	
GB18703		0.3	-0.6	11	16	595	487	CG13890
GB16725		0.3	-0.6	35	51	1174	961	CG15881
GB13763		0.3	-0.6	24	35	361	296	CG7637
GB15525		0.3	-0.6	28	41	2360	1936	CG4164
GB19701	Hexokinase putative	0.3	-0.6	34	50	3736	3070	CG3001
GB12274	Dhfr	0.3	-0.6	7	11	304	250	CG14887
GB18172	DhpD	0.3	-0.6	7	11	683	562	CG18143
GB12164	Ucrh	0.3	-0.6	47	69	972	800	CG41623
GB11791		0.3	-0.6	9	14	725	597	CG4882
GB30525		0.3	-0.6	828	1227	6242	5174	

GB17736	Pdi	0.3	-0.6	225	333	11372	9432	CG6988
GB14676	oxen	0.3	-0.6	9	14	194	161	CG8764
GB13648		0.3	-0.6	14	20	459	381	CG15019
GB15275		0.3	-0.6	14	21	397	330	
GB16175	Eap	0.3	-0.6	26	39	2942	2447	CG3594
GB19124		0.3	-0.6	19	29	928	774	CG4038
GB16049		0.3	-0.6	137	204	5887	4916	CG5885
GB19388		0.3	-0.6	128	191	1955	1634	CG32276
GB14262	ade5	0.3	-0.6	18	27	1792	1499	CG3989
GB18953		0.3	-0.6	33	49	1054	884	
GB17561	mRpL36	0.2	-0.6	14	21	272	229	CG18767
GB17637	mRpS31	0.2	-0.6	12	19	689	581	CG5904
GB12567	bgm	0.2	-0.6	94	141	15157	12792	CG4501
GB15418		0.2	-0.6	9	13	336	284	CG18643
GB16120	bor / dATAD3A	0.2	-0.6	8	12	1115	945	CG6815
GB13689	Derlin-1	0.2	-0.6	51	77	6434	5466	CG10908
GB10947		0.2	-0.6	7	10	452	386	CG13850
GB18969	Hsp60C	0.2	-0.6	316	483	42451	36307	CG7235
GB16493	mRpS33	0.2	-0.6	20	31	525	450	CG10406
GB13210	Ef1β	0.2	-0.6	152	232	7782	6671	CG6341
GB18633	regucalcin	0.2	-0.6	141	217	10463	8971	CG1803
GB16684		0.2	-0.6	40	61	2901	2498	CG6084
GB10060	Manf	0.2	-0.7	27	42	1130	974	CG7013
GB14564	viking	0.2	-0.7	48	75	20775	17912	CG16858
GB15779	G6PD / Zwischenferment	0.2	-0.7	51	80	6278	5439	CG12529
GB19988		0.2	-0.7	434	673	15658	13567	CG6426
GB12011		0.2	-0.7	10	16	292	255	CG12848
GB30365	Obp56i	0.2	-0.7	77	120	2134	1868	CG30448
GB18261	Nurf-38	0.2	-0.7	20	32	1586	1390	CG4634
GB15700		0.2	-0.7	32	50	5026	4413	CG33129
GB11056	Pgk	0.2	-0.7	55	86	6296	5538	CG3127
GB14866		0.2	-0.7	57	90	5327	4688	
GB15016	Hsc70-3	0.2	-0.7	265	417	41168	36263	CG4147
GB11642	Sec61α	0.2	-0.7	80	128	9025	8030	CG9539
GB12781	LKR / lysine ketoglutarate reductase	0.2	-0.7	11	18	2437	2175	CG7144
GB18700		0.2	-0.7	20	32	625	559	CG14407
GB10507		0.2	-0.7	7	12	157	141	CG12935
GB17548	Short-chain dehydrogenase	0.2	-0.7	40	64	2316	2087	CG3603
GB17238	Pyruvate dehydrogenase E1β subunit / PDH	0.1	-0.7	31	50	2433	2197	CG11876
GB14223	NHP2	0.1	-0.7	22	35	768	694	CG5258
GB16986	Glycoside hydrolase	0.1	-0.7	16	25	1325	1199	CG9307
GB15619	Transketolase	0.1	-0.7	223	364	32764	29854	CG5103
GB19000	Tim9a	0.1	-0.7	12	19	109	100	CG1660
GB19274	Surf6, heme transporter activity	0.1	-0.7	7	11	219	201	CG4510
GB15966	α4GT1	0.1	-0.7	8	14	143	132	CG17223
GB12949	phosphogluconate dehydrogenase activity.	0.1	-0.7	113	186	13069	12069	CG3724
GB12198	FAS, Fatty acid synthase	0.1	-0.8	92	151	50963	47179	CG3523
GB15790		0.1	-0.8	8	13	167	155	CG13606
GB19070	Baldspot / Fatty acyl-CoA elongase	0.1	-0.8	53	88	3608	3362	CG3971
GB15052	Pglym78	0.1	-0.8	63	106	3797	3539	CG1721
GB11765		0.1	-0.8	13	21	277	260	CG14818
GB14594	Cyp6-clade	0.1	-0.8	99	168	11680	11019	CG10240
GB14205	Chaperonin Cpn10; GroES-like	0.1	-0.8	442	749	10824	10273	CG9920
GB14898		0.1	-0.8	159	270	299	285	
GB14140	Tim8	0.1	-0.8	31	53	636	608	CG1728
GB16075		0.0	-0.8	140	247	2903	2863	
GB16978	FAD linked oxidase	0.0	-0.9	19	34	2087	2088	CG3835
GB15409	Cyp6-clade	0.0	-0.9	16	30	1952	2004	CG8453
GB15410	nucleotide binding; oxidoreductase activity	-0.1	-0.9	15	28	938	980	CG18814
GB18649	enoyl-CoA hydratase activity	-0.1	-0.9	67	126	4312	4543	CG8778
GB10992	ATPCL	-0.1	-1.0	61	116	15579	16633	CG33138
GB10047		-0.1	-1.0	14	27	511	548	
GB15937	sel	-0.2	-1.0	5	11	259	288	CG12918
GB14494	Hsp90	-0.2	-1.0	49	98	8257	9278	CG1242
GB18737	Alcohol dehydrogenase	-0.2	-1.0	675	1368	41971	47606	CG3481
GB10851	AcCoAS	-0.2	-1.1	14	30	1966	2279	CG9390
GB16202	Glycine N-methyltransferase	-0.5	-1.4	9	22	615	864	CG6188
GB15742		-0.6	-1.5	10	28	37	58	
GB18334	carbohydrate metabolic process	-2.5	-3.4	3	32	30	173	
