# Supplementary material for: Transient Exposure to Low Levels of Insecticide Affects Metabolic Networks of Honeybee Larvae
Source: PLoS One. 2013 Jul 2;8(7):e68191. doi: 10.1371/journal.pone.0068191 (PMC3699529; doi:10.1371/journal.pone.0068191)
Supplement: Table S3 — Lipid profiles: LC/MS data. (PDF) [file pone.0068191.s005.pdf]

Table S3 **Lipid profile: Ions found in the lipid fraction of honeybee larvae using LC/MS**

Transient exposure to low levels of insecticide affects metabolic network of honeybee larvae. Derecka et al. (2013)

m/z	Elution time(Min)	ES mode	ID (where available)	P value (T-Test)	Ratio of IMIDACLOPRID-EXPOSED/ CONTROL (IE/E)
850.788	18.083	+	TG + NH4?	8.83E-05	3.50E-03
896.719	10.783	+	PC	6.20E-05	8.30E-03
871.721	6.388	+	Glycerophosphocholines Diacylglycerophosphocholines	3.97E-05	1.90E-02
850.787	7.248	+		6.48E-05	1.90E-02
897.742	7.641	+	PC(22:1(13E)/22:1 Diacylglycerophosphocholines	3.54E-05	2.20E-02
929.815	14.98	+	PC(23:0/23:0), or PC(20:0/26:0)	8.26E-06	2.40E-02
923.755	8.603	+	Diacylglycerophosphocholines	4.38E-04	2.60E-02
860.783	12.213	+		5.70E-05	4.30E-02
924.757	13.765	+		3.33E-04	0.1
872.728	9.736	+		2.74E-04	0.1
892.808	10.251	+		1.47E-04	0.1
929.811	15.758	+		2.97E-04	0.1
899.768	6.408	+		3.19E-05	0.1
814.637	3.898	+		1.46E-05	0.1
824.778	16.956	+	PC	4.27E-04	0.1
789.548	8.558	-		0.000722	0.1
591.514	9.563	+		8.51E-06	0.1
551.514	12.032	+		4.91E-05	0.1

871.725	7.006	+		7.13E-05	0.1
897.753	7.987	+		2.53E-04	0.1
802.7	13.704	+		9.00E-05	0.1
898.744	13.551	+	TG/SM	4.29E-04	0.1
924.759	15.083	+		3.31E-04	0.1
801.698	13.89	+		1.38E-05	0.1
812.627	18.907	+	TG	2.61E-04	0.1
895.737	8.44	+		6.17E-04	0.1
591.513	18.254	+		8.31E-06	0.1
593.515	11.409	+		5.54E-06	0.2
866.788	11.941	+	DG	3.39E-04	0.2
802.697	14.331	+		3.73E-05	0.2
552.519	12.264	+		5.29E-08	0.2
873.742	10.723	+		8.02E-05	0.2
621.541	11.409	+		1.55E-06	0.2
788.544	8.192	-	DG	0.000952	0.2
603.536	11.631	+	PS	2.70E-04	0.2
361.194	0.543	-		0.000166	0.2
811.595	3.072	+		4.76E-07	0.2
812.543	6.843	-		5.96E-05	0.3
551.504	13.702	+		4.59E-05	0.3
604.54	10.188	+	DG	4.29E-04	0.3
604.53	9.333	+		1.54E-04	0.3
866.786	11.353	+		2.08E-04	0.3
859.786	14.318	+		1.89E-04	0.3
772.584	3.268	-		1.77E-07	0.3
604.541	13.609	+	PE/PC	1.40E-05	0.3
878.512	18.628	-		6.36E-07	0.3
621.544	12.031	+		9.48E-06	0.3
603.535	13.657	+	DG	3.12E-05	0.3
577.52	10.144	+	DG	1.13E-07	0.3
551.505	15.478	+		9.72E-04	0.3
793.572	2.903	+	DG(P-14:/18:1(9Z))	2.02E-05	0.3
865.575	7.25	-		0.000927	0.3

471.321	6.201	+	PI	5.02E-05	0.3
577.521	14.528	+		7.15E-06	0.3
793.573	2.232	+		8.39E-05	0.3
888.584	18.745	+		1.64E-04	0.3
876.573	3.67	+		7.17E-04	0.3
470.318	5.959	+		2.52E-05	0.4
824.568	5.186	+		2.32E-04	0.4
772.587	4.634	-		0.000136	0.4
746.561	13.295	+	PE/PC	2.53E-05	0.4
579.531	16.975	+		8.75E-05	0.4
464.359	6.145	+		1.21E-05	0.4
469.315	5.638	+		5.28E-05	0.4
793.068	0.766	+	PC	9.48E-04	0.4
798.543	1.121	+		2.00E-04	0.4
321.211	2.674	+		2.27E-07	0.4
339.221	2.558	+		5.57E-06	0.5
399.77	2.481	+		3.15E-04	0.5
331.187	0.794	-		0.000973	0.5
321.212	0.617	+		2.14E-04	0.5
171.138	5.449	+		8.69E-04	0.6
321.21	1.22	+	DIOL	2.84E-04	0.6
504.346	2.423	+		2.40E-04	0.6
478.331	2.996	+		3.26E-04	0.6
149.024	2.293	+	PE(P-19:1(12Z)/:)	1.42E-04	0.6
112.985	2.689	-		0.000171	0.7
478.33	2.461	+		6.18E-04	0.7
149.024	1.388	+	PE(P-19:1(12Z)/:)	2.68E-04	1.4
265.253	0.712	+		1.80E-04	1.6
233.154	1.937	-		0.000868	1.7
255.233	4.537	-	LARGE ESTER/FFA C15H22O2	0.000596	1.8
177.104	0.748	+	FFA C16H32O2	9.72E-04	1.8
107.05	0.732	+	C10H12N2O 3 candidates including serotonin, HT, cotinine	3.12E-05	1.9
123.082	1.008	+	BENZALEHYDE	5.82E-05	1.9

152.995	1.389	-		7.35E-05	2.2
240.269	0.713	+	Propanoyl phosphate	4.95E-04	2.2
109.102	3.052	+		6.76E-04	2.2
121.03	1.045	+		9.21E-06	2.3
173.097	0.752	+		1.53E-05	2.3
273.168	0.731	+	Glycylproline	3.30E-04	2.3
466.33	2.285	-		0.000163	2.4
149.06	1.028	+	PE	1.40E-05	2.4
195.102	1.045	+	C9H8O2 7 CANDIDATES	1.37E-05	2.4
167.071	0.97	+		1.32E-05	2.4
742.541	8.231	-	C9H10O3 25 CANDIDATES	0.000425	2.4
123.118	3.052	+	PE/PC	2.76E-04	2.4
313.274	15.299	+		3.61E-04	2.4
168.074	1.047	+	C19H36O3 FFA ESTERS	1.13E-05	2.5
133.087	0.578	+		6.89E-04	2.5
552.5	9.517	-		0.00057	2.5
904.635	10.143	-		0.000641	2.5
336.327	12.768	-		9.55E-05	2.5
226.253	0.664	+	Acyl amide	8.81E-05	2.5
245.137	0.577	+		9.37E-05	2.5
522.283	2.233	-		0.000787	2.6
146.118	0.575	+	PS	4.34E-04	2.6
231.121	0.578	+	Acetylcholine/ 8 CANDIDATES of C7H15NO2	7.76E-05	2.6
265.218	0.76	-		0.00017	2.6
906.648	10.099	-	FFA C17H30O2	0.000188	2.7
299.223	0.81	-		6.36E-05	2.7
435.252	1.602	-	FFA/MG C17H32O4	6.11E-05	2.8
750.588	9.857	+	glycerophosphate	9.43E-04	2.8
930.652	10.709	-		0.000915	2.8
566.516	10.422	-		0.000202	2.8
685.437	10.914	+		6.67E-04	2.9
567.519	10.422	-		0.000195	2.9
274.275	0.694	+	12:0 Cholesteryl ester	3.71E-05	2.9

352.322	9.504	-	C16 Sphinganine	0.000841	2.9
111.118	3.034	+	Anandamide (20:l, n-9)	8.68E-04	2.9
228.233	0.731	+		1.16E-04	2.9
571.289	1.388	-		5.01E-05	2.9
580.339	1.465	-	PI	3.83E-06	2.9
597.305	0.926	-		0.000468	2.9
506.495	10.428	-	PI	9.15E-05	3
555.519	10.432	-		0.000117	3.1
592.53	11.576	-		0.000114	3.1
247.243	0.73	+		3.41E-08	3.1
602.493	10.426	-		0.000159	3.1
580.531	11.486	-		4.30E-06	3.1
246.244	0.675	+		7.64E-06	3.1
593.534	11.558	-		0.000223	3.2
221.154	0.816	-		2.44E-07	3.2
256.264	0.649	+	Large ESTER C14H22O2	3.69E-05	3.2
536.505	10.428	-	FATTY AMIDE	2.40E-05	3.2
583.54	11.484	-	N-Palmitoylsphingosine/ Ceramide (d18:1/16:0)	3.25E-06	3.2
537.508	10.445	-		2.34E-05	3.2
539.512	10.428	-		3.20E-05	3.2
762.566	10.089	-		0.000112	3.2
582.51	10.445	-		2.14E-05	3.2
550.522	11.448	-		2.48E-05	3.3
662.244	2.253	-		0.000171	3.3
764.566	10.089	-		0.000103	3.3
617.511	11.33	-	PE/PC	3.08E-05	3.3
598.542	10.347	-		8.80E-05	3.4
322.73	1.951	+		1.26E-04	3.4
544.339	1.166	+		8.30E-04	3.4
511.492	9.428	-	PC/Lyso PC	7.95E-05	3.4
545.342	1.166	+		8.35E-04	3.4
904.639	9.139	-		0.000129	3.4
473.283	1.837	-		9.14E-05	3.5

618.573	16.382	+		9.25E-04	3.5
726.59	10.089	-		0.000848	3.5
598.308	1.39	-	Glucosylceramide	2.16E-05	3.5
599.314	1.541	-		0.000129	3.5
564.537	10.089	-		0.000291	3.5
285.243	2.021	+	CERAMIDE	3.13E-04	3.5
294.03	0.68	-	C17H32O3	2.83E-06	3.6
260.046	0.68	-		4.62E-06	3.6
510.489	9.428	-		8.06E-05	3.6
616.508	11.446	-	Cer(d18:0/14:0)	1.08E-06	3.6
599.546	10.327	-		7.28E-05	3.7
701.577	9.382	-		0.000932	3.7
656.575	9.309	-		0.000357	3.7
611.399	9.309	-		2.50E-06	3.7
569.272	0.779	-		0.00026	3.8
619.509	11.446	-		7.91E-07	3.8
616.566	16.382	+	PA (GLYCEROPHOSPATE)	7.66E-04	3.8
578.515	10.64	-		0.000153	3.8
572.481	10.445	-		2.82E-06	3.8
574.48	10.423	-		3.26E-06	3.8
1175.777	9.324	-		0.000868	3.9
554.515	10.64	-		0.000243	3.9
1177.782	9.336	-	CERAMIDE	0.000843	4
583.551	11.27	-		3.43E-06	4
614.492	10.514	-		0.000255	4
538.521	11.44	-	DG	7.54E-08	4.1
232.929	0.578	+		5.47E-04	4.2
584.526	11.44	-		1.49E-08	4.2
701.41	10.918	+		6.49E-04	4.2
540.528	11.44	-		9.62E-08	4.3
652.397	9.428	-		0.000294	4.5
1344.945	11.205	+		5.72E-04	4.5
560.503	10.819	+		5.21E-04	4.5
561.506	10.786	+	N-Palmitoylsphingosine	5.20E-04	4.5

381.353	9.076	+		1.34E-04	4.6
1329.926	11.107	+		9.65E-04	4.6
395.278	1.339	+		1.54E-04	4.8
112.113	0.637	+	C23H38O5 ESTERS	8.53E-04	4.9
562.508	10.716	+		9.85E-05	5
128.021	0.617	+		2.46E-04	5
131.003	2.135	+		2.83E-06	5.2
639.43	10.107	-		5.50E-05	5.2
590.55	14.493	+		2.68E-04	5.3
562.518	10.943	+		2.16E-04	5.4
117.092	0.676	+		9.51E-05	5.5
574.497	11.398	-		3.46E-08	5.5
619.427	10.633	-		8.58E-05	5.5
439.416	10.608	-		1.02E-06	5.7
120.082	1.083	+	28-hydroxy-octacosanoic acid	6.23E-05	5.8
620.599	9.309	-		0.000897	5.9
816.679	15.458	+	CERAMIDE	8.91E-04	5.9
149.013	2.166	+	PE	9.94E-06	6
590.541	13.203	+		4.81E-04	6
576.496	11.398	-		8.40E-06	6
324.12	8.958	-		0.000131	6.1
633.508	10.066	+		1.32E-05	6.2
655.571	17.153	+		3.91E-04	6.3
1329.915	11.068	+		5.75E-04	6.3
581.368	1.132	-		0.000271	6.3
661.541	11.168	+		1.67E-05	6.6
815.675	16.146	+	TG Na+	4.51E-04	6.6
110.01	0.617	+		1.46E-04	6.7
693.564	15.018	+		5.25E-07	7.3
663.466	15.418	+	PC	6.31E-06	7.4
381.301	17.312	+		1.93E-04	7.5
625.394	1.132	-	MG	0.000277	7.5
157.994	0.626	-		2.40E-06	7.9
667.461	10.106	-		4.76E-05	8.1

654.568	17.312	+		1.03E-04	8.2
661.539	11.805	+		2.54E-05	8.3
1154.079	13.184	+		8.71E-04	8.3
693.565	16.914	+		1.02E-04	8.6
1155.08	13.186	+	PC	8.18E-04	8.9
234.963	0.617	+		2.06E-04	9.4
843.707	11.181	+		1.95E-05	9.5
844.709	13.013	+		8.93E-04	9.9
675.525	9.15	-		8.94E-05	10.4
617.513	15.359	+		2.27E-04	10.6
681.421	14.4	+	DG	1.38E-04	10.8
216.952	0.617	+		1.91E-04	11.1
278.053	0.626	-		6.78E-06	11.1
602.551	14.474	+		4.19E-04	13.9
841.682	11.132	+		4.74E-04	14
662.552	10.884	+		1.15E-04	14.6
867.697	14.506	+		8.87E-04	15.5
663.456	15.577	+		5.44E-07	15.9
666.536	12.649	+		1.90E-04	20.2
648.563	16.086	+		1.54E-04	22.6
843.709	11.701	+		2.08E-05	48.5
602.55	15.008	+		8.98E-04	57.8
813.659	16.842	+		7.05E-05	62.6
859.622	16.771	+		9.08E-05	69
843.706	12.913	+		8.39E-05	169.9
644.597	9.293	+		4.44E-04	195.9
860.624	16.781	+		3.42E-04	327.4
843.708	12.264	+	PC	7.19E-05	347.1
