# Supplementary material for: Transient Exposure to Low Levels of Insecticide Affects Metabolic Networks of Honeybee Larvae
Source: PLoS One. 2013 Jul 2;8(7):e68191. doi: 10.1371/journal.pone.0068191 (PMC3699529; doi:10.1371/journal.pone.0068191)
Supplement: Table S4 — List of agrochemicals applied to fields. (PDF) [file pone.0068191.s006.pdf]

TABLE S4
Field data

Information of Agrochemicals applied to the fields adjacent of the hives

Transient exposure to low levels of insecticide affects metabolic network of honeybee larvae. Derecka et al. (2013)

FIELD S31

YEAR	CROP	TREATMENT	APPLICATION DATE
2009/2010	Winter Wheat	Slurry	17/03/2010
		Cyproconazole	10/04/2010
		Chlorothalonil	10/04/2010
		Propiconazole	10/04/2010
		Chlormequat	10/04/2010
		Fluroxypyr	03/05/2010
		Chlorothalonil	03/05/2010
		Epoxiconazole	03/05/2010
		Chlormequat	03/05/2010
		Choline chloride	03/05/2010
		Chlorothalonil	28/05/2010
		Epoxiconazole	28/05/2010
		Metsulfuron-methyl	28/05/2010
		Pyraclostrobin	17/06/2010
		Tebuconazole	17/06/2010
2010/2011	Winter Wheat	Glyphosate480	07/10/2010
		Flufenacet	24/11/2010
		Diflufenican	24/11/2010
		Cypermethrin	24/11/2010
		Iodosulfuron-methyl-sodium	18/03/2011
		Mesosulfuron-methyl	18/03/2011
		Chlormequat	17/04/2011
		Choline chloride	17/04/2011
		Cyproconazole	17/04/2011
		Chlorothalonil	17/04/2011
		Propiconazole	17/04/2011
		Epoxiconazole	03/05/2011
		Azoxystrobin	03/05/2011
		Tebuconazole	03/05/2011
2011/2012	Oilseed Rape	Propyzamide	17/11/2011
		Flusilazole	17/11/2011
		Cypermethrin	17/11/2011
		Carbendazim	05/04/2012
		Tau-fluvalinate	05/04/2012
		Tebuconazole	05/04/2012
		Prothioconazole	05/04/2012

FIELD S24

YEAR	CROP	TREATMENT	APPLICATION DATE
2009/2010	Winter Wheat	Iodosulfuron-methyl-sodium	09/04/2010
		Cyproconazole	21/04/2010
		Chlorothalonil	21/04/2010
		Propiconazole	21/04/2010
		Chlormequat	21/04/2010
		Nuram + S	27/04/2010
		Nuram + S	28/04/2010
		Azoxystrobin	09/05/2010
		Chlorothalonil	09/05/2010
		Epoxiconazole	09/05/2010
		Metsulfuron-methyl	09/05/2010
		Tebuconazole	21/06/2010
2010/2011	Winter Oats	Slurry	Feb-Apr
		Glyphosate480	17/09/2010
		Diflufenican	03/11/2010
		Nitram	17/03/2011
		Mn Jett	23/03/2011
		Chlormequat	23/03/2011
		Fluroxypyr	23/03/2011
		Metsulfuron-methyl	23/03/2011
		Nuram + S	04/05/2011
2011/2012	Winter Wheat	Flufenacet	31/10/2011
		Diflufenican	31/10/2011
		Lambda-cyhalothrin	31/10/2011
		N35S	13/03/2012
		Boscalid	12/04/2012
		Epoxiconazole	12/04/2012
		Chlorothalonil	12/04/2012
		Trinexapac-ethyl	12/04/2012
		Epoxiconazole	14/05/2012
		Fluxapyroxad	14/05/2012
		Metsulfuron-methyl	14/05/2012
		Tribenuron-methyl	14/05/2012
		Fluroxypyr	14/05/2012
