# Supplementary material for: Transient Exposure to Low Levels of Insecticide Affects Metabolic Networks of Honeybee Larvae
Source: PLoS One. 2013 Jul 2;8(7):e68191. doi: 10.1371/journal.pone.0068191 (PMC3699529; doi:10.1371/journal.pone.0068191)
Supplement: Table S5 — Larva. (PDF) [file pone.0068191.s007.pdf]

TABLE S5 **Larval weights**

Transient exposure to low levels of insecticide affects metabolic network of honeybee larvae. Derecka et al. (2013)

Individual worker-bee larvae from each hive were weighed and numbered. The IDs of larvae used for RNA-Seq (micro RNA and whole transcriptome) are indicated (red numbers). Larvae had been collected in the field and stored in ice-cold RNAlater solution and were subsequently stored at -80oC. Weighing of larvae took place after thawing the RNAlater solution, while larvae were still in a semi-frozen state. Thus, the recorded weights do not capture with 100% accuracy the actual weights of larvae. Small droplets or crystals of RNAlater solution are likely to have been contributing factors to the measurements of some larvae.

larval weight (mg)	hives			hives		
	IE1	IE2	IE3	C1	C2	C3
34						77
50	18,19,20				78,79,80,81,82	
60	1		38, 39, 40	51, 52, 53, 54, 55		
65						83
70	2,3,4,	21	41,42,43	56	63,64	84
75	5,6				65,66	
80	7,8,9,10,	22, 23, 24, 25, 26,			67,68	85
90	11	27,28,29,30		57,58	69,70,71	
100	12	31,32	44,45		72,73,74,75	86,87
120	13,14,	33, 34,35, 36, 37	46	61,62	76	
140	15,26			59,60		
150						
190	47					
