# Supplementary material for: Transient Exposure to Low Levels of Insecticide Affects Metabolic Networks of Honeybee Larvae
Source: PLoS One. 2013 Jul 2;8(7):e68191. doi: 10.1371/journal.pone.0068191 (PMC3699529; doi:10.1371/journal.pone.0068191)
Supplement: Data S3 — Box plots of 17 genes/GeXP-based multiplex RT-PCR data. (PDF) [file pone.0068191.s010.pdf]

Data S3 Validation of RNA-Seq data by GeXP-based multiplex RT-PCR

(Transient exposure to low levels of insecticide affects metabolic networks of honeybee larvae. Derecka et al. 2013)

Plots reflect the variance in RNA expression levels as assessed between treatment groups (‘treatment effect’plots on the left), among hives (‘hive effects’ plots; middle column = variation across individuals, within a hive), and for individual larvae (‘larval effects’ plots on the right). Box and whisker plots show the full range of data: 25th percentile (bottom of box), 50th percentile (middle of box), 75th percentile (top of box), median (line in middle of box). Relative mRNA levels were measured twice (two technical replicates) for 17 genes from individual, whole larval samples using the GenomeLab Gene Expression Profiler genetic analysis system (GeXP / Beckman Coulter, USA) multiplex RT-PCR detection platform. Relative expression levels are compared to a standardisation factor: the geometric mean of three genes (Actin, Ubiquitin and GB19767), whose expression levels were relatively consistent and Kan, a synthetic reference control transcript provided in the GeXP assay kit (Beckman Coulter). Note, that y-axes here are scaled consistently within a gene, but not between genes; the values on the y-axis can change for each different gene. ‘Larva effect’ plots: samples 1 – 34 came from larvae of control hives ‘C1, C2, C3’; samples 35 - 74 came from imidacloprid-exposed hives ‘IE1, IE2, IE3’. As two measurements were made for each larva, the height of each column in the ‘larval effect’ plots (right) can be interpreted as the confidence interval on estimates for that larva: a small point for an individual larva indicates consistent measurements for the two technical replicates, while two divergent measurements are indicated by large bars. For some genes not all 74 data points are provided. That is, information was excluded from our analysis when a given larval RNA sample did not yield valid data for a particular gene.

Cyp6 clade (c) / GB15793

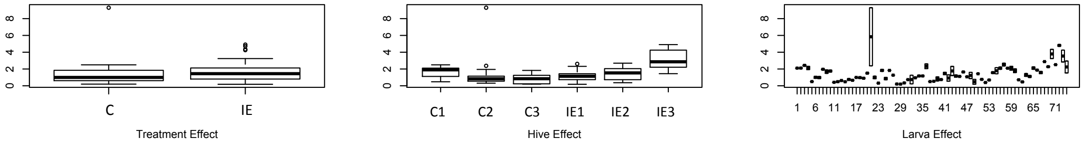

Cyp9 clade (b) / GB14836

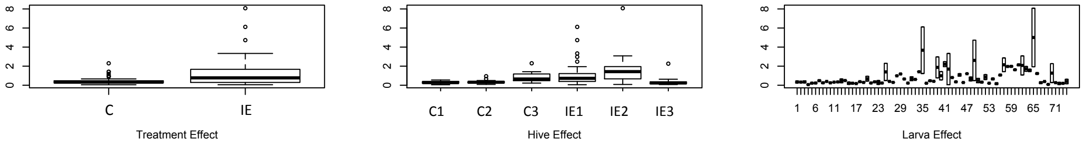

Cyp9 clade (a) / GB16803

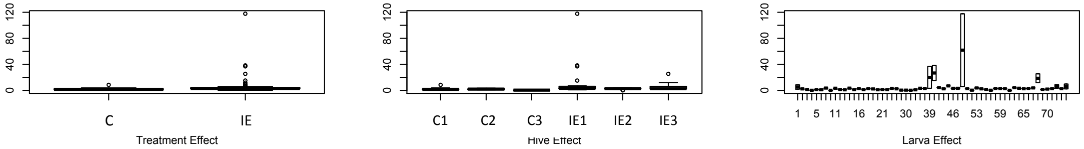

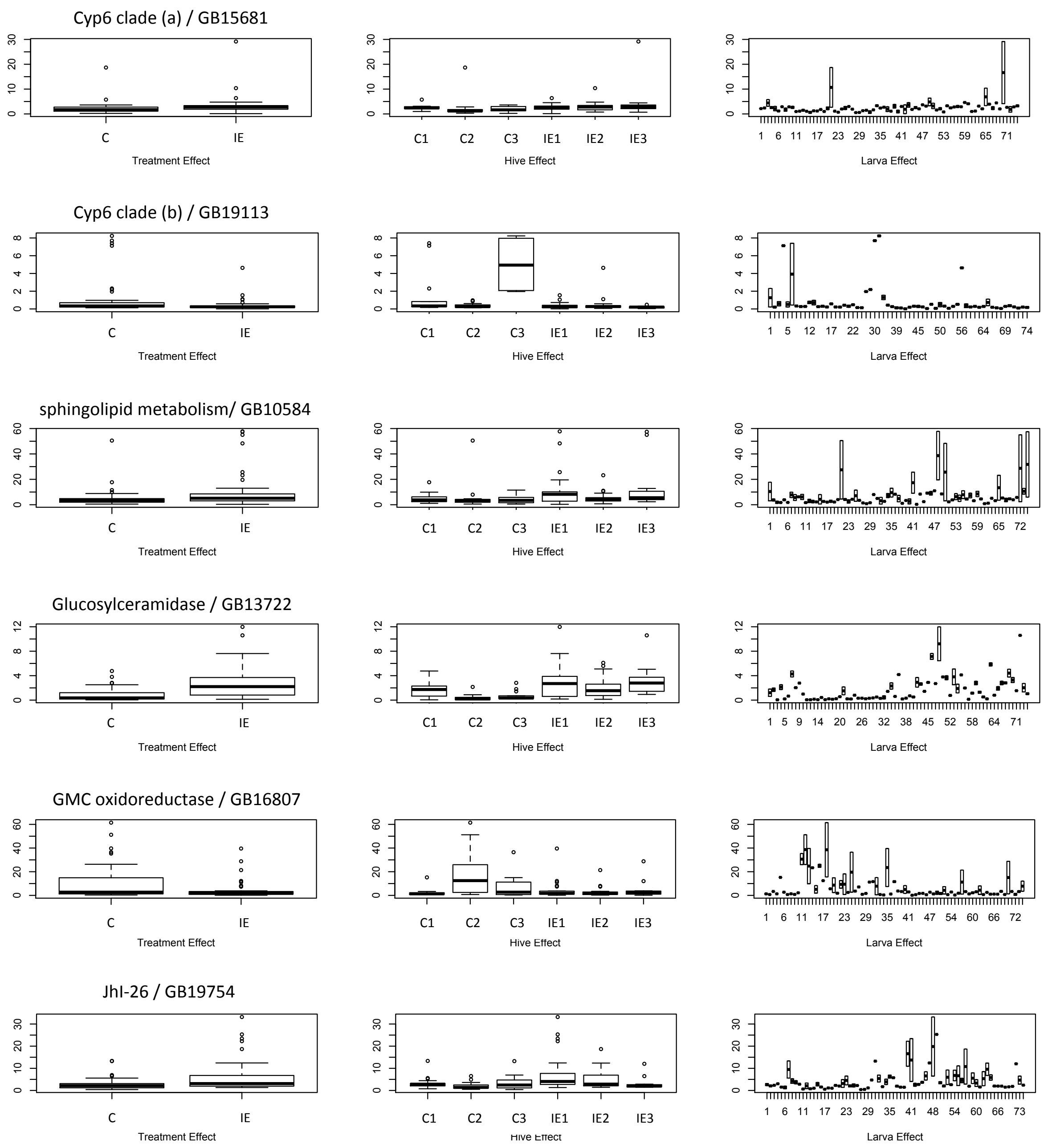
Cyp6 clade (a) / GB15681
Cyp6 clade (b) / GB19113
sphingolipid metabolism/ GB10584
Glucosylceramidase / GB13722
GMC oxidoreductase / GB16807
JhI-26 / GB19754
C
IE
Treatment Effect
C1
C2
C3
IE1
IE2
IE3
Hive Effect
Larva Effect

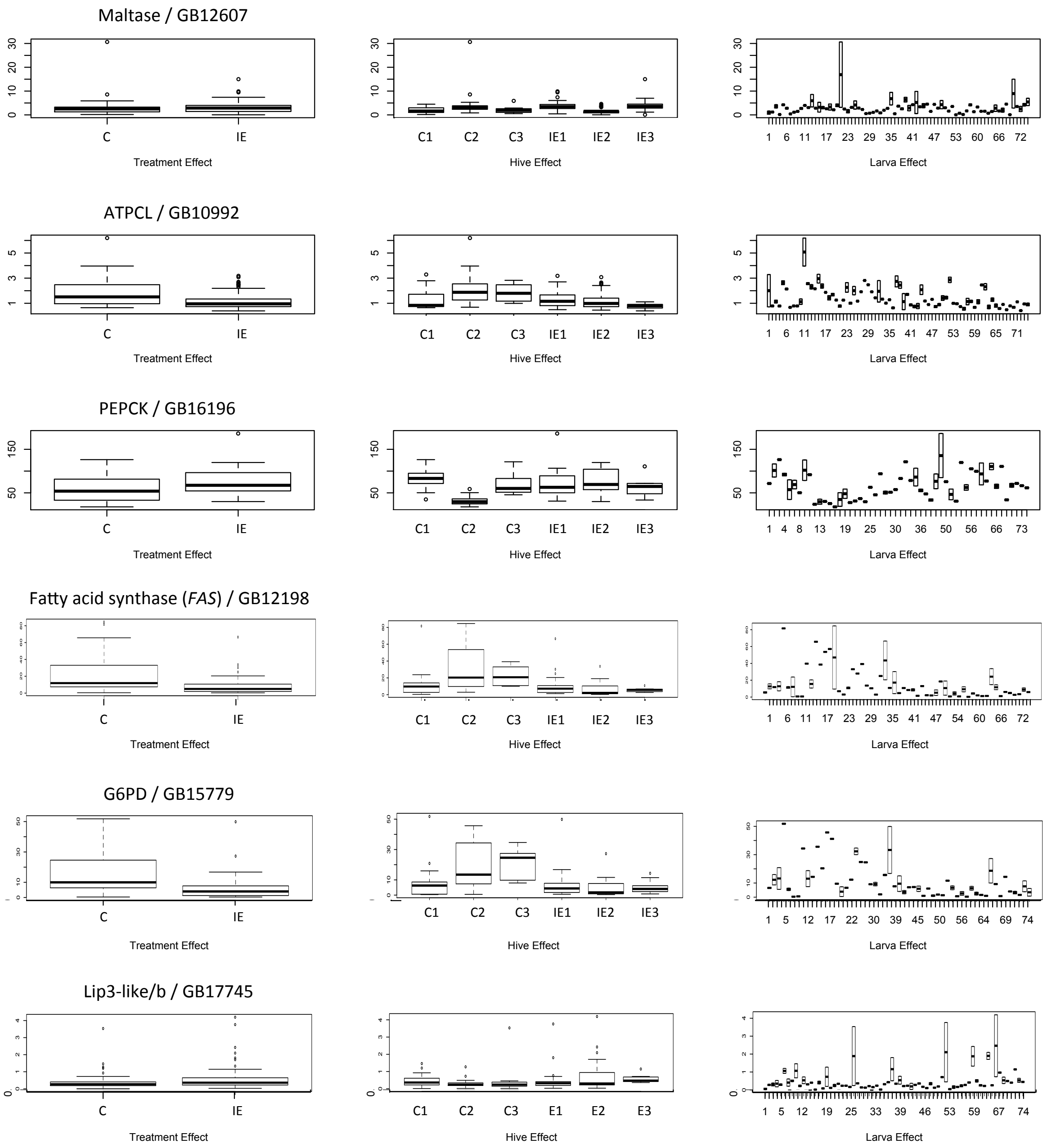

Maltase / GB12607
C
IE
Treatment Effect
C1
C2
C3
IE1
IE2
IE3
Hive Effect
Larva Effect
ATPCL / GB10992
C
IE
Treatment Effect
C1
C2
C3
IE1
IE2
IE3
Hive Effect
Larva Effect
PEPCK / GB16196
C
IE
Treatment Effect
C1
C2
C3
IE1
IE2
IE3
Hive Effect
Larva Effect
Fatty acid synthase (*FAS*) / GB12198
C
IE
Treatment Effect
C1
C2
C3
IE1
IE2
IE3
Hive Effect
Larva Effect
G6PD / GB15779
C
IE
Treatment Effect
C1
C2
C3
IE1
IE2
IE3
Hive Effect
Larva Effect
Lip3-like/b / GB17745
C
IE
Treatment Effect
C1
C2
C3
E1
E2
E3
Hive Effect
Larva Effect

Lip3-like/a / GB17220

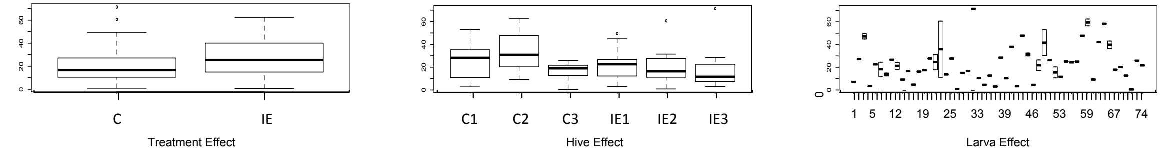

HSP90 / GB14495

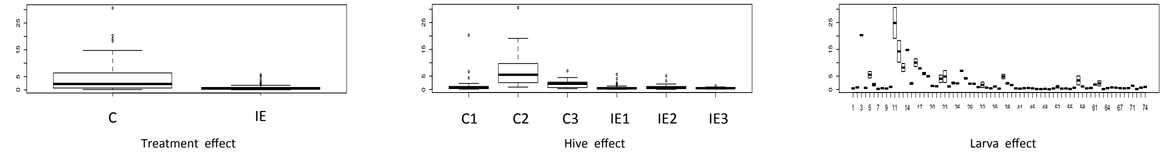

Actin / GB17681

Used for standardisation NOTE: the small y-axis scale

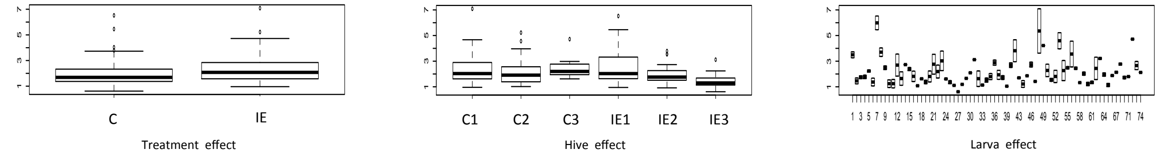

'Neutral' / GB19767

Used for standardisation NOTE: the small y-axis scale

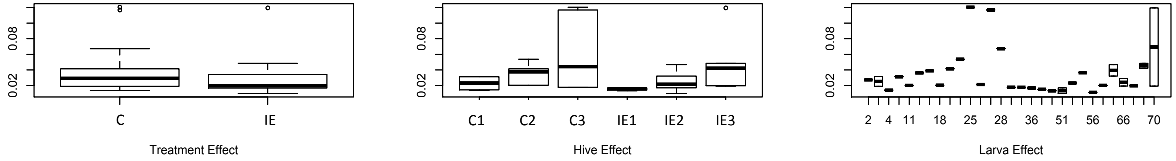

Ubiquitin / GB16469

Used for standardisation NOTE: the small y-axis scale

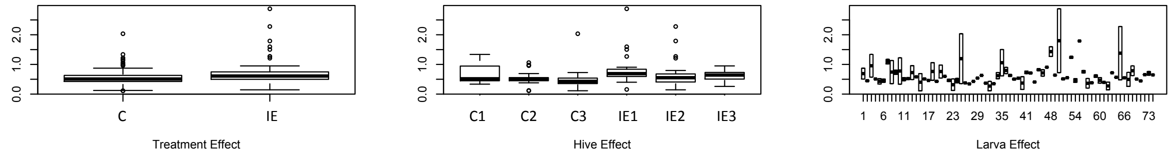

Data S3 Genes and primers used in the GeXP multiplex RT-PCR

Transient exposure to low levels of insecticide affects metabolic network of honeybee larvae.
Derecka et al. (2013)

log2 (Fold_change) normalised in 'IE' samples; determined by RNA-Seq	Name	Accession Number	Left Primer	Right Primer	product length & primer positions
-0.96	*ATPCL*	GB10992	ATCCAGCTGGTCGAATATGG	TACGTTTGCTGTTCACTGGG	(142 bp) (809,20)-(950,20)
0.77	*Cyp6-subtype/a*	GB15681	ATTCGAGGATGATGCTTTCG	ACGGTATCAAAGTTTTGGCG	(170 bp) (1257,20)-(1426,20)
1.01	*Cyp6-subtype/b*	GB19113	GATTAGCAACGCCCTTTACG	CAAGAACGGCAAAGATGGAT	(184 bp) (933,20)-(1116,20)
0.94	*Cyp6-subtype/c*	GB15793	GTCGGACGAGCGTAGTGAAT	ACAAAATCTGGCCTCACGAC	(219 bp) (594,20)-(812,20)
0.74	*Cyp9-subtype/a*	GB16803	CTCGTATCCCGACACGAAAT	GCTCGAAATGCTTAACCGAG	(107 bp) (195,20)-(301,20)
3.35	*Cyp9-subtype/b*	GB14836	CCAAGTGTTCCCACGATTTT	AACTCGTTGTCCGGATGTTC	(163 bp) (476,20)-(638,20)
-0.75	*Fatty acid synthase*	GB12198	TCTCTTGGTTAGCCAGGGTG	ACTTCCCCATTGTCCTCCTT	(205 bp) (4044,20)-(4248,20)
-0.66	*G6PD*	GB15779	ATTTTGTCCGATCATCTCGC	CTGCATGACGTCCCTAATGA	(240 bp) (559,20)-(798,20)
0.70	*GMC oxidoreductase*	GB16807	ATTTCGCCGAGAAGGAAGAT	AGTGTTCCAGGTTGCATTCC	(177 bp) (1025,20)-(1201,20)
-0.8	*Glucosylceramidase*	GB13722	GCGAATGAACGTGTCAGAAG	ACGGCCCAATGTATATCTGC	(226) (210,20)-(435,20)
-1.03	*HSP90*	GB14495	GGGAATCTAGTGCTGGTGGA	TCTTCCTCTGGTGCTTCTTCA	(307 bp) (470,20)-(776,21)
0.68	*JhI-26*	GB19754	TCGATGACGTACGCTACGAG	GCGGTTTCTTGAGAACAAGG	(135 bp) (74,20)-(208,20)
0.76	*Lip3-like/a*	GB17220	ACTTGGTGCACAAATTGCAG	ACCGAATAACGGACAACCTG	(247 bp) (3510,20)-(3756,20)
0.64	*Lip3-like/b*	GB17745	TGAATGCGCGTGGTAATAGA	ATGAAAACTGCTGGAGCCAT	(265 bp) (362,20)-(626,20)
0.82	*Maltase*	GB12607	GCGACAAAGCGAAGAAGAAG	TGGAGTTTACGCTGCTTGTG	(128 bp) (1485,20)-(1612,20)
0.77	*PEPCK*	GB16196	TAATTGGATTTCTCCGGCTG	GTTGAACCAACTGGACCCAT	(114 bp) (411,20)-(524,20)
0.70	*sphingolipid metabo*	GB10584	GTGTTGGGATCATGGGAAAG	AGGTTGGTCCACCATCTTTG	(121 bp) (1138,20)-(1258,20)
	Controls				
-0.08	*Actin*	GB17681	TGCCAACACTGTCCTTTCTG	AGAATTGACCCACCAATCCA	(100 bp) (1077,20)-(1176,20)
-0.07	*Ubiquitin*	GB16469	ATAAAGCAGCTGCCGATCAA	TTCCTGCCACATCACCTTTT	(198 bp) (1413,20)-(1610,20)
0.00	*Neutral*	GB19767	ATTTCAGCCTCGCGTTTTTA	TGACTTGGCCGAAGTAAACC	(272 bp) (2221,20)-(2492,20)
	Kan(r)		ATCATCAGCATTGCATTCGATTCCT(	ATTCCGACTCGTCCAACATC	Kan(r)

(external synthetic reference control transcript Kan)

Data S3 Unprocessed (raw) expression values obtained by GeXP multiplex RT-PCR for individual genes of individual larval samples

Transient exposure to low levels of insecticide affects metabolic network of honeybee larvae. Derecka et al.

hive	treatment	samplie	UBQ GB16469	ACT GB17681	Maltase / GB12607	ATPCL / GB10992	Cyp6 GB15681	b_Cyp6_GB15793
2	ie	050_018	7,929	23,116	35,211	35,972	59,468	25,942
2	ie	050_018	9,878	30,490	50,557	40,891	61,859	32,862
2	ie	050_019	1,903	4,580	15,574	14,109	23,474	5,869
2	ie	050_019	2,595	8,140	30,926	21,570	33,973	7,846
2	ie	050_020	2,470	8,096	337	8,392	7,154	6,103
2	ie	050_020	1,761	7,245	266	6,931	5,244	4,527
6	c	050_078	1,861	7,783	18,942	18,199	31,854	12,943
6	c	050_078	5,452	16,688	35,644	27,981	48,317	27,916
6	c	050_079	ND	ND	ND	ND	ND	ND
6	c	050_079	7,864	23,192	66,714	27,123	81,057	49,664
6	c	050_081	ND	ND	ND	ND	ND	ND
6	c	050_081	12,583	22,508	51,233	43,115	82,560	54,627
6	c	050_082	12,761	34,913	23,861	97,868	56,962	21,756
6	c	050_082	16,161	45,170	24,419	127,730	68,114	25,475
2	ie	060_001	1,807	6,727	3,726	6,145	18,981	9,176
2	ie	060_001,1i	896	5,244	4,321	5,312	18,384	8,733
3	ie	060_038	4,637	13,968	39,757	12,748	37,179	21,787
3	ie	060_038	6,666	17,447	37,753	15,747	39,037	22,732
3	ie	060_039	ND	5,092	23,040	2,467	10,963	7,102
3	ie	060_039	ND	ND	5,984	ND	1,085	1,579
3	ie	060_040	6,935	29,959	ND	24,295	46,682	53,781
3	ie	060_040	10,431	31,213	2,524	24,163	51,357	56,406
4	c	060_051	4,237	15,801	6,887	13,941	29,306	29,744
4	c	060_051	8,085	30,219	8,426	21,649	47,121	44,985
4	c	060_052	6,770	24,383	32,085	18,101	44,393	39,154
4	c	060_052	9,815	28,152	30,333	23,457	55,012	48,285
4	c	060_053	7,493	24,794	67,712	25,390	80,230	63,346
4	c	060_053	9,374	26,660	77,045	33,329	103,269	78,889
4	c	060_054	10,064	32,364	ND	23,607	68,516	54,787
4	c	060_054	10,469	39,180	6,399	32,832	72,353	63,200
4	c	060_055	5,195	21,397	85,696	53,118	66,447	28,059
4	c	060_055	5,553	29,792	119,770	83,181	101,239	39,615
6	c	065_082	7,340	22,597	16,188	55,864	33,041	11,190
6	c	065_083	3,165	10,511	11,015	20,113	26,145	6,860
2	ie	070_002	3,654	10,187	ND	7,415	18,117	15,998
2	ie	070_002,1i	ND	8,145	ND	6,027	16,434	8,604
2	ie	070_003	2,453	14,060	2,578	6,394	17,363	12,551
2	ie	070_003,1i	3,660	13,136	1,848	8,923	25,783	19,929

hive	treatment	sample	UBQ GB16469	ACT GB17681	Maltase / GB12607	ATPCL / GB10992	Cyp6 GB15681	b_Cyp6_GB15793
2	ie	070_004	3,658	17,627	29,827	15,487	40,292	41,748
2	ie	070_004,1i	643	4,259	13,493	5,950	20,444	14,057
1	ie	070_021	3,222	9,696	45,489	12,508	29,986	15,114
1	ie	070_021	4,758	12,105	50,593	15,422	38,678	17,513
3	ie	070_041	8,497	28,313	76,391	20,812	71,953	67,470
3	ie	070_041	6,071	21,898	63,257	13,632	56,133	59,224
3	ie	070_042	ND	ND	ND	ND	ND	ND
3	ie	070_042	5,629	22,777	80,195	25,352	67,341	63,768
4	c	070_056	5,597	11,773	36,509	25,690	35,661	29,120
4	c	070_056	5,015	21,140	52,856	44,252	43,647	39,407
5	c	070_063	6,034	17,085	65,260	67,730	66,549	29,000
5	c	070_063	7,002	14,235	58,702	88,177	82,728	35,779
5	c	070_064	13,319	49,307	144,061	122,837	142,032	67,786
5	c	070_064	4,652	30,038	97,045	79,356	83,629	34,047
6	c	070_084	2,105	17,826	29,328	25,540	37,630	18,746
2	ie	075_005	4,440	16,007	61,485	16,936	48,946	35,087
2	ie	075_005,1i	4,143	13,882	64,014	17,231	49,305	36,588
2	ie	075_006	6,390	21,370	30,518	15,684	70,114	36,472
2	ie	075_006,1i	7,533	25,036	30,811	15,442	73,360	28,792
5	c	075_065	10,410	46,642	146,627	99,781	119,901	59,610
5	c	075_065	14,591	33,289	110,904	82,664	99,416	54,327
5	c	075_066	4,174	19,590	55,283	42,185	44,865	20,219
2	ie	080_007	6,729	22,522	51,354	21,649	73,949	34,083
2	ie	080_007,1i	4,319	12,636	43,187	16,293	62,458	32,878
2	ie	080_008	3,346	10,939	16,209	27,384	25,427	19,851
2	ie	080_008,1i	3,421	12,010	18,555	28,984	30,659	20,311
2	ie	080_009	1,136	4,380	7,385	11,213	12,349	5,706
2	ie	080_009	1,686	10,365	17,520	21,439	19,726	8,970
2	ie	080_010	2,186	9,852	5,602	5,600	18,077	8,190
2	ie	080_010	2,490	11,021	9,228	9,401	28,850	10,571
1	ie	080_022	6,390	19,052	9,923	13,012	33,266	26,547
1	ie	080_022	2,997	13,395	5,856	7,862	22,460	20,527
1	ie	080_023	4,663	12,322	42,382	39,215	38,689	19,411
1	ie	080_023	5,117	20,144	47,347	46,360	44,455	23,658
1	ie	080_024	6,927	22,607	65,950	61,113	67,284	32,925
1	ie	080_024	8,800	31,111	67,755	68,124	75,819	36,841
1	ie	080_026	8,576	16,748	90,794	29,666	70,414	31,509
1	ie	080_026	ND	5,144	38,054	2,530	9,662	1,460
5	c	080_067	1,087	3,935	20,799	12,998	13,290	8,661
5	c	080_067	3,279	9,597	39,674	25,077	23,760	20,990
5	c	080_068	2,061	8,682	30,624	22,094	28,771	13,815
5	c	080_068	5,667	16,242	41,257	35,842	43,121	24,681

hive	treatment	sample	UBQ GB16469	ACT GB17681	Maltase / GB12607	ATPCL / GB10992	Cyp6 GB15681	b_Cyp6_GB15793
6	c	080_085	4,266	11,356	24,972	12,470	27,931	15,711
6	c	080_085	920	4,802	5,562	13,468	17,005	3,115
2	ie	090_011	3,487	12,947	15,486	14,892	28,143	14,925
2	ie	090_011	4,278	13,702	22,683	19,488	43,131	23,819
1	ie	090_027	2,246	10,470	39,309	17,112	26,442	11,831
1	ie	090_027	3,065	13,094	52,262	23,330	35,177	18,531
1	ie	090_028	948	5,701	24,026	4,525	13,997	5,953
1	ie	090_028	1,098	4,709	20,517	4,441	13,022	7,165
1	ie	090_029	ND	1,315	12,994	848	3,093	466
1	ie	090_029	ND	655	7,371	550	2,620	716
1	ie	090_030	5,275	12,587	43,620	12,376	33,364	15,095
1	ie	090_030	5,438	13,053	43,806	7,513	34,061	17,388
4	c	090_057	2,087	13,401	5,598	9,370	26,595	8,045
4	c	090_057	2,888	13,436	5,430	8,695	25,587	9,549
4	c	090_058	3,888	13,853	15,052	11,064	27,971	22,423
4	c	090_058	4,136	14,734	14,316	11,455	26,969	21,886
5	c	090_069	4,609	10,852	28,075	20,267	23,882	20,157
5	c	090_069	ND	ND	ND	ND	ND	ND
5	c	090_070	4,146	16,577	39,810	26,657	41,612	24,001
5	c	090_070	8,884	31,778	64,652	40,084	67,896	42,068
5	c	090_071	9,867	35,700	82,239	63,934	62,064	60,826
5	c	090_071	18,546	51,886	103,077	82,175	82,197	69,793
1	ie	100_031	4,086	15,105	45,469	25,612	47,579	26,568
1	ie	100_031	4,538	10,502	49,811	25,849	45,048	33,190
1	ie	100_032	6,134	15,001	58,950	17,368	36,640	18,762
1	ie	100_032	6,260	14,792	64,315	17,821	37,866	20,520
3	ie	100_044	1,382	9,946	35,535	4,369	12,968	21,429
3	ie	100_044	ND	4,323	30,734	1,702	9,592	8,023
3	ie	100_045	1,309	6,119	22,819	5,574	15,524	15,827
3	ie	100_045	2,366	7,247	34,773	7,120	20,548	30,260
5	c	100_072	8,012	33,076	114,937	41,213	70,347	64,296
5	c	100_072	18,722	53,494	146,283	67,790	113,110	98,599
5	c	100_073	8,325	27,614	86,948	23,957	64,034	56,984
5	c	100_073	ND	2,253	69,040	1,548	28,920	14,402
5	c	100_074	3,479	18,770	48,382	23,918	26,980	19,964
5	c	100_074	5,949	23,607	55,546	28,971	32,249	33,317
5	c	100_075	1,704	10,893	23,087	20,478	15,330	6,666
5	c	100_075	1,369	6,056	13,440	16,020	11,744	5,003
6	c	100_086	6,159	20,017	38,953	26,597	43,445	25,323
6	c	100_087	5,977	15,688	40,697	16,160	58,425	17,573
6	c	100_087	3,359	7,790	22,351	7,728	23,421	9,670
2	ie	120_013	6,868	15,228	40,940	14,373	58,123	32,309

hive	treatment	sample	UBQ GB16469	ACT GB17681	Maltase / GB12607	ATPCL / GB10992	Cyp6 GB15681	b_Cyp6_GB15793
2	ie	120_013	12,367	32,582	47,522	22,321	75,053	43,840
2	ie	120_014	8,880	31,826	53,115	17,032	69,397	32,369
2	ie	120_014	10,522	34,423	55,617	21,698	82,145	39,829
1	ie	120_033	8,734	31,084	49,006	27,893	54,104	44,516
1	ie	120_033	7,780	32,707	44,924	29,347	62,702	49,842
1	ie	120_034	7,154	16,341	65,332	21,449	65,515	22,939
1	ie	120_035	1,677	7,445	20,709	4,859	15,785	4,151
1	ie	120_035	3,347	9,568	27,975	7,869	23,926	12,645
1	ie	120_036	950	6,268	15,993	6,135	20,139	4,744
1	ie	120_036	ND	1,073	6,487	864	5,508	157
1	ie	120_037	3,020	9,094	23,733	11,202	20,869	16,050
1	ie	120_037	3,393	8,402	23,612	13,477	28,671	19,310
3	ie	120_046	3,539	11,617	45,058	11,775	33,690	34,763
3	ie	120_046	ND	2,928	20,456	2,331	8,389	3,569
4	c	120_061	4,948	14,352	24,113	11,906	34,424	22,521
4	c	120_061	4,299	17,006	21,556	12,629	35,720	18,680
4	c	120_062	4,886	13,850	42,532	18,611	41,805	34,666
4	c	120_062	4,465	18,869	34,785	17,163	34,914	28,323
5	c	120_076	1,576	6,790	22,237	7,299	13,345	12,582
5	c	120_076	3,553	10,670	32,326	10,024	21,402	19,551

Data S3	Unprocessed (raw) expression values obtained by GeXP multiplex RT-PCR for individual genes of individual larval samples

Transient exposure to low levels of insecticide affects metabolic network of honeybee larvae. Derecka et al. (2013)

hive	treatment	sample	Cyp9b1_GB14836	b_cyp9b1_GB16803	Cyp6 GB19113	FAS GB12198	G6PD / GB15779	sphingolipid metabo GB10584
2	ie	050_018	9,899	ND	52,937	47,000	29,526	61,491
2	ie	050_018	8,748	ND	55,843	52,959	30,762	77,605
2	ie	050_019	6,529	ND	10,570	11,383	4,729	20,294
2	ie	050_019	11,510	1,908	15,273	13,298	7,673	32,797
2	ie	050_020	1,366	19,944	1,739	4,845	2,490	21,685
2	ie	050_020	892	18,315	1,103	2,875	3,920	15,733
6	c	050_078	6,362	ND	12,313	12,225	11,109	29,651
6	c	050_078	11,506	ND	29,317	35,949	24,594	54,035
6	c	050_079	ND	ND	ND	ND	ND	ND
6	c	050_079	19,854	ND	39,562	29,701	26,400	91,263
6	c	050_081	ND	ND	ND	ND	ND	ND
6	c	050_081	16,191	17,261	37,784	85,962	52,603	75,198
6	c	050_082	20,870	ND	48,817	107,579	71,110	79,086
6	c	050_082	25,130	ND	52,438	136,645	90,459	74,486
2	ie	060_001	5,371	19,903	5,544	7,113	4,599	11,913
2	ie	060_001,1i	6,417	20,883	6,252	3,347	1,963	14,802
3	ie	060_038	7,762	83,018	11,855	11,887	11,975	61,096
3	ie	060_038	7,971	81,756	12,117	15,874	13,712	58,265
3	ie	060_039	2,764	83,823	4,573	ND	ND	37,495
3	ie	060_039	ND	40,076	ND	ND	ND	7,164
3	ie	060_040	3,899	70,098	22,287	22,125	23,516	95,658
3	ie	060_040	5,509	80,097	20,629	26,374	18,530	97,573
4	c	060_051	6,241	52,682	14,378	10,274	13,651	38,507
4	c	060_051	10,575	82,750	18,545	17,691	18,965	58,082
4	c	060_052	12,902	85,223	21,101	19,172	17,005	76,393
4	c	060_052	15,613	109,772	18,249	29,168	29,793	87,834
4	c	060_053	13,786	88,352	43,987	25,725	21,728	38,041
4	c	060_053	19,027	108,267	36,920	30,951	33,209	36,472
4	c	060_054	2,561	ND	38,521	28,449	21,304	43,242
4	c	060_054	5,186	5,976	42,604	31,296	35,864	51,999
4	c	060_055	7,596	30,110	24,028	37,329	20,284	85,903
4	c	060_055	13,239	49,998	24,129	46,845	34,257	118,014
6	c	065_082	13,205	ND	27,949	48,396	30,351	47,848
6	c	065_083	4,471	2,048	15,765	20,093	4,510	36,177
2	ie	070_002	1,507	22,823	9,160	7,142	8,117	27,449
2	ie	070_002,1i	698	13,386	7,732	4,652	3,910	18,683
2	ie	070_003	14,212	1,777	8,227	7,652	4,764	24,547
2	ie	070_003,1i	20,012	ND	12,228	10,516	8,440	32,101

hive	treatment	sample	Cyp9b1_GB14836	b_cyp9b1_GB16803	Cyp6 GB19113	FAS GB12198	G6PD / GB15779	sphingolipid metabolism
2	ie	070_004	59,261	107,638	15,909	16,807	13,989	75,814
2	ie	070_004,1i	24,642	46,693	7,627	3,222	2,891	35,620
1	ie	070_021	19,046	ND	2,983	10,263	6,376	41,921
1	ie	070_021	21,470	ND	4,184	14,802	7,609	46,225
3	ie	070_041	16,054	125,089	39,935	27,619	25,260	77,384
3	ie	070_041	16,602	117,633	31,467	19,339	16,729	65,436
3	ie	070_042	ND	ND	ND	ND	ND	ND
3	ie	070_042	17,165	164,348	37,998	24,973	16,367	94,216
4	c	070_056	5,457	48,626	21,365	29,150	18,511	36,135
4	c	070_056	10,830	3,028	22,418	30,617	17,428	28,947
5	c	070_063	9,388	98,157	30,229	49,172	42,586	87,061
5	c	070_063	11,486	110,978	27,980	68,778	61,127	79,939
5	c	070_064	21,391	68,331	59,333	101,867	93,654	161,021
5	c	070_064	12,756	51,521	31,196	37,237	25,738	111,771
6	c	070_084	4,194	2,956	24,342	15,112	9,432	46,609
2	ie	075_005	70,122	ND	25,092	16,203	15,817	44,546
2	ie	075_005,1i	70,792	ND	30,462	16,446	13,918	48,482
2	ie	075_006	74,511	101,947	18,565	18,664	13,370	85,758
2	ie	075_006,1i	55,018	79,925	21,166	18,956	8,251	87,582
5	c	075_065	19,164	52,070	44,730	81,416	66,408	146,738
5	c	075_065	13,656	40,631	39,472	100,926	72,118	108,423
5	c	075_066	9,918	68,334	17,745	31,569	29,125	64,762
2	ie	080_007	58,555	ND	14,020	21,550	13,152	79,607
2	ie	080_007,1i	51,879	ND	11,988	17,111	16,992	69,358
2	ie	080_008	43,900	52,715	13,320	25,452	12,175	11,593
2	ie	080_008,1i	41,814	52,901	17,413	25,268	19,551	14,308
2	ie	080_009	17,560	ND	4,156	8,942	6,447	8,838
2	ie	080_009	35,332	570	6,663	10,136	6,286	18,285
2	ie	080_010	11,948	31,238	6,238	4,713	3,063	15,610
2	ie	080_010	20,827	44,434	7,419	4,213	3,601	18,189
1	ie	080_022	4,983	19,116	29,529	11,982	7,001	59,110
1	ie	080_022	4,802	17,764	18,560	6,031	4,868	39,331
1	ie	080_023	7,984	59,040	25,344	32,868	21,184	48,522
1	ie	080_023	9,185	80,339	27,755	36,932	26,287	56,042
1	ie	080_024	13,346	95,961	26,703	53,616	43,992	50,298
1	ie	080_024	15,264	109,591	26,465	61,798	46,333	49,781
1	ie	080_026	24,282	99,632	23,346	37,769	26,827	89,860
1	ie	080_026	4,340	53,831	1,725	ND	ND	33,168
5	c	080_067	2,887	10,833	4,208	6,852	2,656	21,131
5	c	080_067	4,658	20,127	8,675	20,225	8,617	39,325
5	c	080_068	3,487	42,121	8,651	14,512	12,788	38,285
5	c	080_068	4,122	58,230	13,926	34,967	24,853	54,469

hive	treatment	sample	Cyp9b1_GB14836	b_cyp9b1_GB16803	Cyp6 GB19113	FAS GB12198	G6PD / GB15779	sphingolipid metabolism
6	c	080_085	12,087	ND	16,479	15,278	11,838	46,410
6	c	080_085	2,768	ND	6,413	7,479	3,225	18,659
2	ie	090_011	21,382	48,243	15,000	14,242	9,720	32,495
2	ie	090_011	40,213	76,896	19,470	17,234	13,935	42,544
1	ie	090_027	18,295	ND	10,521	10,683	3,630	30,084
1	ie	090_027	26,470	ND	12,646	16,156	8,009	36,915
1	ie	090_028	14,716	ND	9,120	2,017	1,125	28,890
1	ie	090_028	13,946	ND	7,973	2,807	2,599	23,437
1	ie	090_029	1,554	17,862	235	ND	ND	4,427
1	ie	090_029	950	10,769	274	ND	ND	2,553
1	ie	090_030	12,446	65,214	13,197	14,459	9,478	79,804
1	ie	090_030	13,699	70,440	12,876	14,813	9,637	80,598
4	c	090_057	4,424	30,517	10,211	5,767	2,603	25,764
4	c	090_057	3,890	30,247	10,029	7,982	4,107	23,394
4	c	090_058	5,893	ND	13,086	8,497	7,276	61,732
4	c	090_058	6,599	ND	13,664	8,439	7,317	58,060
5	c	090_069	3,524	22,078	12,659	27,247	16,555	34,036
5	c	090_069	ND	ND	ND	ND	ND	ND
5	c	090_070	5,648	67,642	9,240	23,577	25,064	44,962
5	c	090_070	13,906	98,084	21,881	47,185	40,199	75,565
5	c	090_071	17,055	134,494	26,090	57,991	49,156	115,524
5	c	090_071	21,240	144,354	33,663	96,933	69,856	135,909
1	ie	100_031	10,339	66,987	17,816	19,526	16,714	42,075
1	ie	100_031	10,627	71,021	24,866	21,812	18,827	43,694
1	ie	100_032	18,336	125,575	25,929	9,836	7,664	80,834
1	ie	100_032	19,608	142,045	24,694	10,439	8,306	81,750
3	ie	100_044	6,684	70,205	11,517	2,114	993	54,668
3	ie	100_044	4,366	69,225	4,339	ND	ND	39,293
3	ie	100_045	3,244	40,591	9,581	3,181	3,380	28,335
3	ie	100_045	5,850	73,407	13,562	5,742	3,181	40,782
5	c	100_072	27,751	60,760	25,511	32,802	32,670	137,507
5	c	100_072	39,663	81,145	41,132	74,364	59,365	156,789
5	c	100_073	12,162	44,408	18,829	22,989	24,246	108,867
5	c	100_073	3,637	24,598	4,547	ND	1,326	66,983
5	c	100_074	7,907	24,391	11,632	16,172	12,004	56,384
5	c	100_074	10,427	31,607	14,811	31,978	18,589	66,156
5	c	100_075	4,710	20,943	5,809	9,952	3,025	32,279
5	c	100_075	2,472	14,965	2,660	8,552	4,691	18,172
6	c	100_086	15,417	ND	32,221	32,177	21,566	57,534
6	c	100_087	24,895	ND	26,629	13,464	14,052	79,288
6	c	100_087	13,588	ND	14,525	6,558	4,840	42,758
2	ie	120_013	66,135	77,116	34,401	22,419	14,277	71,058

hive	treatment	sample	Cyp9b1_GB14836	b_cyp9b1_GB16803	Cyp6 GB19113	FAS GB12198	G6PD / GB15779	sphingolipid metabolism
2	ie	120_013	84,821	108,387	39,842	38,199	24,338	87,790
2	ie	120_014	37,618	94,778	17,340	22,546	16,686	81,919
2	ie	120_014	51,539	119,297	23,211	26,193	16,443	94,322
1	ie	120_033	13,024	138,739	33,712	18,336	10,081	105,225
1	ie	120_033	13,712	156,142	33,237	18,687	13,273	101,064
1	ie	120_034	24,774	ND	6,588	13,750	10,654	91,280
1	ie	120_035	3,570	17,172	8,939	1,794	633	36,563
1	ie	120_035	5,726	27,310	14,448	4,481	2,611	51,089
1	ie	120_036	2,283	27,848	3,148	2,191	2,117	18,008
1	ie	120_036	743	18,514	348	ND	ND	8,013
1	ie	120_037	3,009	41,636	12,443	9,276	6,930	30,581
1	ie	120_037	4,943	48,052	11,535	10,958	9,832	30,057
3	ie	120_046	5,126	77,276	19,897	8,918	9,322	56,211
3	ie	120_046	2,254	34,675	3,104	ND	414	23,802
4	c	120_061	9,073	ND	17,936	13,642	11,257	65,733
4	c	120_061	7,265	ND	12,883	10,862	8,761	57,825
4	c	120_062	8,303	ND	8,704	17,634	18,472	74,927
4	c	120_062	5,771	2,095	5,642	14,326	14,175	59,926
5	c	120_076	2,695	29,258	3,793	4,659	6,013	26,288
5	c	120_076	4,788	44,137	6,451	9,015	9,187	38,739

**Data S3**	Unprocessed (raw) expression values obtained by GeXP multiplex RT-PCR for individual genes of individual larval samples

Transient exposure to low levels of insecticide affects metabolic network of honeybee larvae. Derecka et al. (2013)

**hive**	**treatment**	**sample**	**Glucosylceramidase GB13722**	**GMC oxidored / GB16807**	**HSP90 / GB14494**	**JHIP26 / GB19754**	**Neutral / GB19767**	**Lip3-like_a GB17220**
2	ie	050_018	ND	80,311	25,241	42,292	720	22,090
2	ie	050_018	ND	79,985	22,642	53,608	524	23,812
2	ie	050_019	3,918	29,002	5,657	15,339	ND	9,558
2	ie	050_019	5,458	38,144	3,051	28,137	ND	9,428
2	ie	050_020	12,689	8,759	4,194	10,111	ND	ND
2	ie	050_020	9,887	5,900	3,025	8,556	198	ND
6	c	050_078	3,108	8,498	6,484	19,258	ND	8,517
6	c	050_078	8,531	17,851	23,580	36,839	1,063	21,751
6	c	050_079	ND	ND	ND	ND	ND	ND
6	c	050_079	8,295	30,078	18,927	42,166	899	29,615
6	c	050_081	ND	ND	ND	ND	ND	ND
6	c	050_081	11,440	81,426	79,684	31,204	3,645	55,758
6	c	050_082	23,599	20,044	105,467	43,102	4,063	12,519
6	c	050_082	24,865	22,592	95,341	53,678	3,603	21,744
2	ie	060_001	5,436	16,465	2,606	11,416	ND	6,518
2	ie	060_001,1i	5,122	13,717	1,493	13,696	ND	3,660
3	ie	060_038	35,623	36,481	17,439	34,015	ND	19,073
3	ie	060_038	36,418	40,756	17,883	35,237	1,240	22,165
3	ie	060_039	3,671	14,109	ND	11,675	ND	1,161
3	ie	060_039	566	1,669	ND	2,313	ND	ND
3	ie	060_040	88,950	64,417	36,942	56,293	2,378	ND
3	ie	060_040	93,570	67,526	40,774	59,817	2,903	ND
4	c	060_051	23,328	35,420	14,400	31,033	ND	6,367
4	c	060_051	34,372	57,056	15,278	48,207	ND	9,332
4	c	060_052	33,733	25,453	21,547	51,756	ND	19,513
4	c	060_052	42,652	29,305	38,763	65,820	1,797	33,033
4	c	060_053	1,120	77,537	22,735	55,301	976	29,312
4	c	060_053	ND	93,495	28,436	63,886	1,217	40,430
4	c	060_054	53,592	73,549	29,999	92,089	1,327	5,302
4	c	060_054	62,270	78,089	39,185	116,560	1,596	6,431
4	c	060_055	4,771	72,594	20,694	33,935	ND	3,850
4	c	060_055	5,284	92,823	35,329	49,276	ND	18,477
6	c	065_082	11,022	18,193	23,941	27,648	493	4,276
6	c	065_083	2,283	25,666	4,842	22,877	ND	7,711
2	ie	070_002	33,585	4,664	10,020	22,032	368	ND
2	ie	070_002,1i	16,074	3,887	1,162	12,899	ND	ND
2	ie	070_003	9,722	19,710	5,682	31,147	ND	9,510
2	ie	070_003,1i	16,254	26,784	11,097	39,153	440	15,033

hive	treatment	sample	Glucosylceramidase GB13722	GMC oxidored / GB16807	HSP90 / GB14494	JHIP26 / GB19754	Neutral / GB19767	Lip3-like_a GB17220
2	ie	070_004	1,915	40,904	9,744	28,187	ND	44,678
2	ie	070_004,1i	427	17,190	739	13,803	ND	12,601
1	ie	070_021	3,799	24,981	4,840	27,012	ND	23,666
1	ie	070_021	4,660	34,669	4,362	30,355	ND	28,843
3	ie	070_041	72,541	105,757	36,585	59,884	1,395	35,970
3	ie	070_041	61,939	81,535	31,796	51,845	999	29,290
3	ie	070_042	ND	ND	ND	ND	ND	ND
3	ie	070_042	25,225	83,182	34,920	64,172	ND	18,397
4	c	070_056	12,315	34,462	28,131	21,316	665	12,804
4	c	070_056	11,370	29,700	15,347	30,300	ND	13,186
5	c	070_063	2,993	77,608	57,200	45,008	ND	19,002
5	c	070_063	2,911	102,854	89,128	46,006	926	25,942
5	c	070_064	5,520	144,338	100,843	84,767	ND	21,424
5	c	070_064	1,586	81,230	16,157	54,324	ND	9,739
6	c	070_084	2,710	31,595	2,401	31,802	ND	5,854
2	ie	075_005	16,095	55,415	17,471	35,781	ND	33,558
2	ie	075_005,1i	17,655	52,050	20,359	36,632	ND	34,783
2	ie	075_006	33,719	33,269	19,263	60,718	ND	43,370
2	ie	075_006,1i	25,470	38,087	6,415	50,970	ND	31,699
5	c	075_065	10,580	118,835	70,854	71,589	ND	27,769
5	c	075_065	11,064	109,030	107,431	55,873	2,023	37,852
5	c	075_066	2,021	47,183	29,843	30,369	ND	6,789
2	ie	080_007	19,115	43,941	6,330	30,829	ND	43,945
2	ie	080_007,1i	19,379	32,696	15,095	27,163	549	50,064
2	ie	080_008	7,710	22,189	15,742	22,467	ND	21,995
2	ie	080_008,1i	8,071	26,190	13,398	22,705	ND	21,396
2	ie	080_009	1,097	8,761	3,538	7,624	ND	6,948
2	ie	080_009	1,503	15,819	2,026	17,237	ND	8,187
2	ie	080_010	17,128	11,244	2,882	19,550	ND	11,485
2	ie	080_010	21,960	16,408	2,574	31,823	ND	12,902
1	ie	080_022	28,936	34,211	8,282	49,565	822	4,797
1	ie	080_022	20,591	22,444	6,407	35,437	604	3,683
1	ie	080_023	4,169	46,191	16,771	38,113	ND	27,918
1	ie	080_023	4,770	56,773	26,959	44,415	ND	36,314
1	ie	080_024	13,633	49,454	27,019	45,150	ND	47,650
1	ie	080_024	15,419	58,005	39,548	52,305	ND	55,382
1	ie	080_026	18,824	61,300	30,958	44,872	ND	47,039
1	ie	080_026	ND	3,715	ND	12,045	ND	ND
5	c	080_067	1,208	9,963	2,334	8,419	ND	2,958
5	c	080_067	3,507	21,298	8,973	18,642	ND	10,705
5	c	080_068	924	24,299	7,939	14,790	ND	2,144
5	c	080_068	1,866	44,276	21,425	27,022	ND	19,192

hive	treatment	sample	Glucosylc eramidase GB13722	GMC oxidored / GB16807	HSP90 / GB14494	JHIP26 / GB19754	Neutral / GB19767	Lip3-like_a GB17220
6	c	080_085	8,331	13,709	5,998	22,866	ND	6,347
6	c	080_085	631	9,468	1,739	12,090	ND	3,226
2	ie	090_011	7,278	19,413	3,065	21,602	ND	19,490
2	ie	090_011	11,949	28,571	7,071	32,857	ND	30,262
1	ie	090_027	2,139	23,405	629	13,993	ND	6,579
1	ie	090_027	3,479	28,627	1,816	19,882	ND	12,630
1	ie	090_028	4,163	14,501	515	12,063	ND	3,358
1	ie	090_028	5,527	12,956	2,633	10,701	ND	5,540
1	ie	090_029	ND	ND	ND	1,036	ND	ND
1	ie	090_029	165	438	ND	569	ND	377
1	ie	090_030	23,200	14,863	9,925	32,127	ND	15,959
1	ie	090_030	27,196	16,435	14,862	28,476	ND	18,439
4	c	090_057	12,393	10,052	1,867	24,901	ND	7,430
4	c	090_057	15,597	10,173	4,413	24,659	ND	11,838
4	c	090_058	15,256	18,171	8,598	27,747	ND	13,869
4	c	090_058	14,490	18,192	5,326	28,159	ND	12,609
5	c	090_069	3,499	43,507	27,626	15,196	591	11,434
5	c	090_069	ND	ND	ND	ND	ND	ND
5	c	090_070	3,247	56,796	17,908	22,046	ND	12,478
5	c	090_070	6,233	97,423	39,709	41,336	844	23,756
5	c	090_071	11,560	64,876	59,952	58,306	ND	28,567
5	c	090_071	15,658	86,560	73,618	77,209	3,196	48,300
1	ie	100_031	23,374	40,245	12,220	35,553	ND	19,591
1	ie	100_031	27,785	42,478	17,010	43,262	ND	21,960
1	ie	100_032	21,482	40,697	8,593	34,401	ND	25,762
1	ie	100_032	24,851	41,936	11,248	37,813	ND	28,574
3	ie	100_044	10,520	18,165	1,556	18,722	ND	5,077
3	ie	100_044	2,546	7,208	ND	13,778	ND	783
3	ie	100_045	4,522	17,284	2,132	13,812	ND	5,198
3	ie	100_045	8,648	22,828	7,497	21,403	ND	10,977
5	c	100_072	19,414	46,833	28,512	59,229	ND	24,453
5	c	100_072	31,443	92,760	36,870	91,503	2,223	51,951
5	c	100_073	21,336	33,009	25,441	50,604	ND	27,197
5	c	100_073	2,858	6,053	ND	15,809	ND	1,996
5	c	100_074	1,985	24,805	5,270	25,856	ND	3,546
5	c	100_074	4,557	27,880	24,438	33,681	ND	20,673
5	c	100_075	807	15,856	2,133	13,878	ND	1,802
5	c	100_075	489	8,883	3,461	8,833	ND	3,295
6	c	100_086	30,780	22,097	20,475	35,129	623	17,078
6	c	100_087	25,345	9,839	13,944	37,517	ND	12,359
6	c	100_087	13,660	4,302	4,257	21,686	ND	7,706
2	ie	120_013	31,535	22,039	26,584	47,606	913	51,594

hive	treatment	sample	Glucosylc eramidase GB13722	GMC oxidored / GB16807	HSP90 / GB14494	JHIP26 / GB19754	Neutral / GB19767	Lip3-like_a GB17220
2	ie	120_013	39,736	30,838	28,559	63,013	2,025	72,281
2	ie	120_014	41,975	48,804	30,424	55,300	1,600	65,082
2	ie	120_014	50,888	52,022	30,736	65,409	1,265	78,401
1	ie	120_033	76,811	44,957	10,448	88,190	ND	46,260
1	ie	120_033	88,475	52,373	18,578	87,302	1,327	59,677
1	ie	120_034	28,853	67,395	4,059	50,414	ND	30,529
1	ie	120_035	7,584	10,488	738	24,477	ND	6,270
1	ie	120_035	16,803	18,857	6,411	41,614	ND	18,776
1	ie	120_036	8,026	7,270	647	16,361	ND	3,847
1	ie	120_036	172	622	ND	6,672	ND	ND
1	ie	120_037	27,554	18,262	9,196	36,599	ND	15,430
1	ie	120_037	32,816	23,391	13,386	40,849	538	21,389
3	ie	120_046	10,852	38,424	10,374	24,941	ND	14,544
3	ie	120_046	388	4,674	ND	8,087	ND	ND
4	c	120_061	31,354	23,429	12,103	35,884	ND	16,399
4	c	120_061	24,417	21,984	7,419	32,843	ND	13,146
4	c	120_062	18,518	41,888	16,676	45,569	ND	27,878
4	c	120_062	13,854	30,576	13,973	40,765	ND	22,521
5	c	120_076	4,738	11,905	4,235	10,595	ND	6,896
5	c	120_076	7,562	18,891	10,346	16,825	ND	14,019

Data S3	Unprocessed (raw) expression values obtained by GeXP multiplex RT-PCR for individual genes of individual larval samples

Transient exposure to low levels of insecticide affects metabolic network of honeybee larvae. Derecka et al. (2013)

hive	treatment	sample	PEPCK / GB16196	Lip3-like_b GB17745	Kan
2	ie	050_018	63,917	556	18,057
2	ie	050_018	76,314	862	18,739
2	ie	050_019	18,955	928	6,636
2	ie	050_019	33,962	798	4,497
2	ie	050_020	31,524	ND	4,397
2	ie	050_020	25,919	ND	3,572
6	c	050_078	30,432	1,204	7,104
6	c	050_078	59,201	4,521	11,875
6	c	050_079	ND	ND	ND
6	c	050_079	80,670	5,986	14,581
6	c	050_081	ND	ND	3,216
6	c	050_081	73,336	7,303	22,863
6	c	050_082	82,460	8,409	26,627
6	c	050_082	98,360	8,436	25,198
2	ie	060_001	31,144	599	3,303
2	ie	060_001,1i	27,788	ND	1,018
3	ie	060_038	76,999	7,333	7,692
3	ie	060_038	80,853	9,655	10,150
3	ie	060_039	29,640	ND	4,777
3	ie	060_039	5,636	ND	7,952
3	ie	060_040	112,731	ND	22,454
3	ie	060_040	121,156	ND	22,988
4	c	060_051	62,495	761	8,682
4	c	060_051	94,147	1,044	13,637
4	c	060_052	83,784	2,751	7,975
4	c	060_052	104,396	4,850	12,387
4	c	060_053	88,982	3,258	18,383
4	c	060_053	103,249	4,148	24,760
4	c	060_054	147,507	ND	17,821
4	c	060_054	164,824	ND	20,616
4	c	060_055	34,195	2,373	9,977
4	c	060_055	49,585	3,173	11,010
6	c	065_082	48,412	2,192	6,349
6	c	065_083	34,965	811	9,747
2	ie	070_002	51,839	ND	6,897
2	ie	070_002,1i	34,648	ND	2,087
2	ie	070_003	58,326	809	2,926
2	ie	070_003,1i	65,015	1,564	4,929

hive	treatment	sample	PEPCK / GB16196	Lip3-like_b GB17745	Kan
2	ie	070_004	54,325	2,425	11,442
2	ie	070_004,1i	23,575	ND	3,454
1	ie	070_021	42,104	2,424	6,450
1	ie	070_021	50,090	3,147	8,002
3	ie	070_041	113,366	13,279	20,208
3	ie	070_041	91,314	10,576	19,319
3	ie	070_042	ND	ND	1,935
3	ie	070_042	104,014	8,168	15,107
4	c	070_056	41,739	3,850	4,909
4	c	070_056	37,860	1,638	6,219
5	c	070_063	28,640	4,928	17,572
5	c	070_063	26,271	6,446	26,628
5	c	070_064	55,490	8,057	10,451
5	c	070_064	28,480	1,360	1,003
6	c	070_084	41,276	418	817
2	ie	075_005	69,972	4,547	12,046
2	ie	075_005,1i	73,216	4,514	11,040
2	ie	075_006	110,552	21,387	15,439
2	ie	075_006,1i	111,193	14,878	9,113
5	c	075_065	62,319	6,071	7,256
5	c	075_065	54,156	11,294	9,322
5	c	075_066	25,488	1,629	1,545
2	ie	080_007	72,623	5,177	9,077
2	ie	080_007,1i	60,609	6,205	11,658
2	ie	080_008	26,111	2,082	6,024
2	ie	080_008,1i	29,166	2,050	4,912
2	ie	080_009	8,132	378	2,470
2	ie	080_009	18,982	ND	2,129
2	ie	080_010	48,466	5,203	4,146
2	ie	080_010	61,662	4,563	3,728
1	ie	080_022	82,423	4,359	13,448
1	ie	080_022	57,771	3,439	10,568
1	ie	080_023	37,883	3,437	13,276
1	ie	080_023	48,930	4,684	16,896
1	ie	080_024	52,801	3,741	16,693
1	ie	080_024	57,335	4,375	19,988
1	ie	080_026	94,031	4,699	20,103
1	ie	080_026	22,768	ND	1,388
5	c	080_067	7,367	ND	747
5	c	080_067	14,271	677	3,690
5	c	080_068	17,649	764	1,781
5	c	080_068	30,162	2,933	6,292

hive	treatment	sample	PEPCK / GB16196	Lip3-like_b GB17745	Kan
6	c	080_085	41,027	1,303	5,677
6	c	080_085	15,549	ND	4,515
2	ie	090_011	41,699	1,744	6,847
2	ie	090_011	55,331	2,340	10,406
1	ie	090_027	26,131	413	1,682
1	ie	090_027	33,766	976	3,128
1	ie	090_028	27,314	ND	1,379
1	ie	090_028	22,907	551	3,644
1	ie	090_029	3,345	ND	16,937
1	ie	090_029	1,645	ND	13,057
1	ie	090_030	80,106	3,581	9,862
1	ie	090_030	84,736	4,338	12,427
4	c	090_057	47,315	872	904
4	c	090_057	46,864	1,775	1,752
4	c	090_058	43,762	2,391	4,134
4	c	090_058	44,274	2,113	3,311
5	c	090_069	11,639	1,351	17,407
5	c	090_069	ND	ND	4,900
5	c	090_070	25,943	1,988	7,584
5	c	090_070	43,211	4,497	14,314
5	c	090_071	60,070	2,552	16,715
5	c	090_071	90,200	5,398	12,211
1	ie	100_031	69,238	1,594	12,165
1	ie	100_031	76,080	2,154	13,912
1	ie	100_032	92,030	1,138	30,008
1	ie	100_032	100,240	1,229	32,658
3	ie	100_044	41,680	1,114	1,444
3	ie	100_044	26,909	ND	591
3	ie	100_045	19,986	776	1,412
3	ie	100_045	28,334	1,926	4,028
5	c	100_072	58,481	2,933	24,572
5	c	100_072	101,842	7,174	29,304
5	c	100_073	61,908	4,338	14,192
5	c	100_073	27,635	ND	4,507
5	c	100_074	21,829	461	1,961
5	c	100_074	27,482	2,354	5,072
5	c	100_075	10,101	ND	1,191
5	c	100_075	6,968	301	2,555
6	c	100_086	72,803	4,753	8,098
6	c	100_087	93,129	3,703	10,540
6	c	100_087	46,992	1,536	4,248
2	ie	120_013	91,605	12,794	16,186

hive	treatment	sample	PEPCK / GB16196	Lip3-like_b GB17745	Kan
2	ie	120_013	128,153	19,859	25,653
2	ie	120_014	105,542	19,626	19,359
2	ie	120_014	129,117	22,072	21,126
1	ie	120_033	162,490	4,177	16,098
1	ie	120_033	167,530	5,581	23,834
1	ie	120_034	85,943	1,809	18,546
1	ie	120_035	36,212	ND	2,886
1	ie	120_035	54,087	1,387	10,562
1	ie	120_036	24,206	439	1,336
1	ie	120_036	9,061	ND	ND
1	ie	120_037	55,424	3,293	6,052
1	ie	120_037	61,503	4,290	10,075
3	ie	120_046	41,223	2,818	5,912
3	ie	120_046	9,915	ND	884
4	c	120_061	70,633	3,756	7,373
4	c	120_061	68,068	2,776	5,858
4	c	120_062	78,342	5,947	11,937
4	c	120_062	73,364	5,046	10,630
5	c	120_076	16,156	500	5,423
5	c	120_076	27,635	1,128	10,611
