# Supplementary material for: Transient Exposure to Low Levels of Insecticide Affects Metabolic Networks of Honeybee Larvae
Source: PLoS One. 2013 Jul 2;8(7):e68191. doi: 10.1371/journal.pone.0068191 (PMC3699529; doi:10.1371/journal.pone.0068191)
Supplement: Figure S2 — STRING analysis. (PDF) [file pone.0068191.s002.pdf]

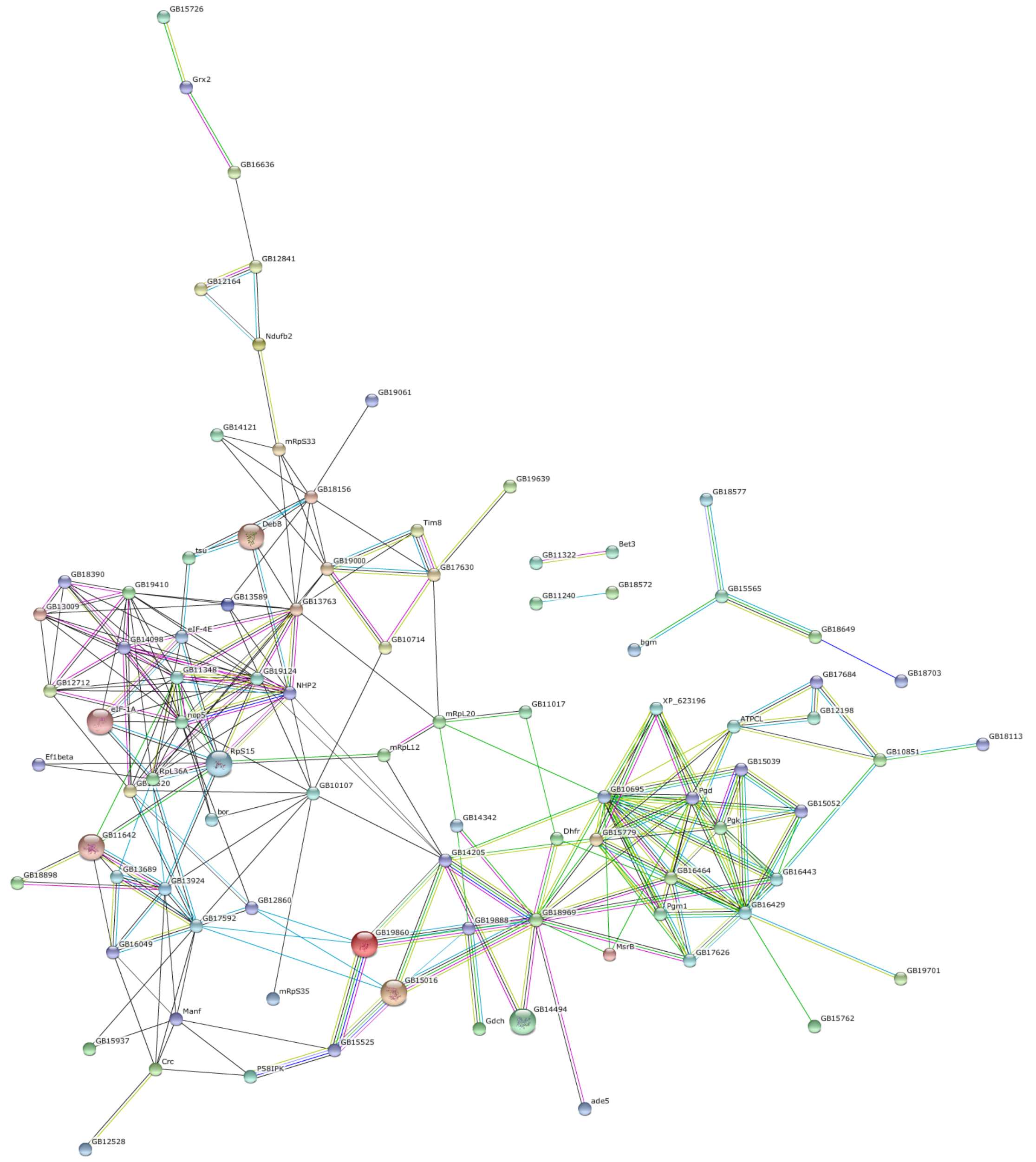

Figure S2

STRING analysis of protein-protein interactions

Shown is a map of known and predicted interactions of gene-products from our list of 195 down-regulated mRNAs in the ‘IE’ data set. The analysis reveals strong links between the down regulation of genes involved in sugar metabolism and translation in both the cell and the mitochondria.

STRING Version 9.O was used for this analysis.

Transient exposure to low levels of insecticide affects metabolic network of honeybee larvae. Derecka et al. 2013
